# Asymmetric Synthesis of Noradamantane Scaffolds via Diphenylprolinol Silyl Ether‐Mediated Domino Michael/Epimerization/Michael (or Aldol)/1,2‐Addition Reactions

**DOI:** 10.1002/anie.202500378

**Published:** 2025-04-11

**Authors:** Konstantinos Daskalakis, Nariyoshi Umekubo, Satrajit Indu, Genki Kawauchi, Tohru Taniguchi, Kenji Monde, Yujiro Hayashi

**Affiliations:** ^1^ Department of Chemistry Graduate School of Science Tohoku University 6‐3 Aramaki Aza‐Aoba, Aoba‐ku Sendai Miyagi 980–8578 Japan; ^2^ Frontier Research Center for Advanced Material and Life Science Faculty of Advanced Life Science Hokkaido University Sapporo 001–0021 Japan; ^3^ Present address: Graduate School of Pharmaceutical Sciences Nagoya University Nagoya 464–8601 Japan

**Keywords:** Asymmetric synthesis, Domino reaction, Noradamantane, Organocatalyst

## Abstract

Topologically unique chiral noradamantanes are synthesized using a diphenylprolinol silyl ether‐mediated domino Michael/epimerization/Michael/1,2‐addition or Michael/epimerization/aldol/1,2‐addition reaction with excellent enantioselectivity in a single reaction vessel. Three carbon–carbon bonds are formed, and six chiral centers, including one all‐carbon quaternary center, are generated, five of which are fully controlled. These functionalized noradamantanes are 3D, cage‐like molecules that can serve as valuable chiral building blocks for drug design.

## Graphical abstract

Topologically unique, 3D cage‐like noradamantanes are synthesized in one pot from simple starting materials. Catalyzed by diphenylprolinol silyl ether, the reaction followed a domino sequence of Michael/epimerization/Michael/1,2‐addition, achieving excellent enantioselectivity. This method formed three C─C bonds and six chiral centers, including a quaternary all‐carbon center, with five fully stereocontrolled.

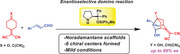

In modern organic synthesis, numerous effective methods have been developed for constructing planar molecules. Beyond planar structures, 3D molecules have garnered significant attention in recent years due to their value as 3D pharmacophores and scaffolds, making them essential tools in the design and development of bioactive molecules.^[^ ^1^, ^2^, ^3^ ^]^ In particular, 3D molecules with functional groups positioned along three almost mutually perpendicular directions, enabling binding to other units, are indispensable in medicinal chemistry.

Polycyclic caged compounds are essential for constructing 3D molecules. They have garnered significant interest from organic chemists not only due to their unique and intriguing molecular architectures but also because of their properties arising from rigid 3D structures. Among these compounds, cubane^[^ ^4^, ^5^, ^6^ ^]^ and adamantane^[^ ^7^, ^8^ ^]^ derivatives are the most prominent classes, with numerous applications in medicinal chemistry and catalysis (Figure 1).

**Figure 1:**
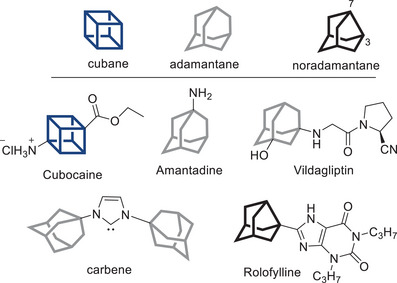
Characteristic polycyclic caged compounds.

Cubane analogs, such as the anesthetic cubocaine,^[^ ^9^ ^]^ can serve as potent bioisosteres for flatter molecules in biological applications. Adamantane derivatives, including amantadine, are used to treat influenza and Parkinson's disease,^[^ ^10^ ^]^ while vildagliptin^[^ ^11^ ^]^ and saxagliptin^[^ ^12^ ^]^ are marketed as anti‐diabetic drugs. The adamantyl moiety enhances lipophilicity, facilitating the transport of these molecules across biological membranes. Additionally, its bulky structure was leveraged by Arduengo and Glorius for the synthesis of thermodynamically stable carbenes, which are extensively applied in synthetic transformations.^[^ ^13^, ^14^ ^]^

Similarly, the methylene‐contracted noradamantane (tricyclo[3.3.1.0^3,7^]nonane) and its derivatives have been explored for potential activities as HDAC inhibitors,^[^ ^15^ ^]^ antivirals,^[^ ^16^ ^]^ and adenosine A1 receptor antagonists, such as rolofylline.^[^ ^17^ ^]^

Despite the rich chemistry of cubane and adamantane, the chemistry of noradamantane has not been well explored, likely due to the lack of efficient synthetic methods. For the synthesis of noradamantane derivatives, only three general methodologies have been reported (Scheme 1). The most widely used approach constructs the noradamantane skeleton by forming a bond between C3 and C7 through reductive coupling reactions using Na,^[^ ^18^, ^19^ ^]^ Zn,^[^ ^20^ ^]^ electrochemical methods,^[^ ^21^ ^]^ and others.^[^ ^22^ ^]^ (Scheme 1 (Eq. 1)). Enolate alkylation is another strategy employed for C3─C7 bond formation (Scheme 1 (Eq. 2)).^[^ ^23^ ^]^ Aside from C3─C7 bond formation, there are only two examples of noradamantane synthesis via acid‐mediated rearrangement (Scheme 1 (Eq. 3))^[^ ^24^ ^]^ and the Wolff rearrangement (Scheme 1 (Eq. 4)).^[^ ^25^ ^]^ All these methods require multiple steps to prepare the starting materials. Consequently, only a few synthetic approaches exist for constructing the noradamantane skeleton, and no established methods are available for synthesizing functionalized noradamantanes or achieving their enantioselective synthesis.

**Scheme 1:**
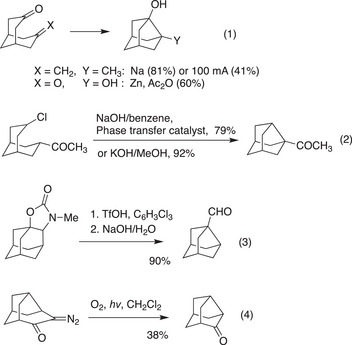
Previous syntheses of the noradamantane framework.

On the other hand, domino reactions represent a highly valued category of modern organic transformations.^[^ ^26^, ^27^, ^28^, ^29^, ^30^, ^31^, ^32^, ^33^, ^34^ ^]^ Their ability to construct complex molecules from simple starting materials in a single sequence significantly reduces the cost, production time, and environmental impact of organic reactions. Developing methods to construct substituted chiral noradamantane derivatives from readily available starting materials via domino reactions is highly desirable.

We recently discovered that non‐activated ketones, such as cyclohexanone, can act as effective nucleophiles in the Michael reaction with α,β‐unsaturated aldehydes, catalyzed by diphenylprolinol silyl ether.^[^ ^35^, ^36^ ^]^ This reaction produces *syn*‐addition products with excellent enantioselectivity (Scheme 2 (Eq. 5)).^[^ ^37^, ^38^ ^]^ Additionally, we identified an enantioselective formal carbo‐[3+3] cycloaddition reaction between isopropylidenemalononitrile and α,β‐unsaturated aldehydes, also catalyzed by diphenylprolinol silyl ether (Scheme 2 (Eq. 6)).^[^ ^39^ ^]^ This reaction involves a domino Michael/addition sequence, followed by Al_2_O_3_‐mediated dehydration, yielding substituted cyclohexene derivatives bearing a methylene‐malononitrile moiety with excellent enantioselectivity.

**Scheme 2:**
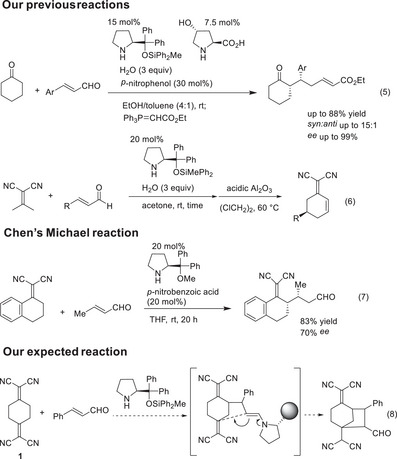
Our previous reactions, Chen's reaction, and the expected reaction.

2‐Methylene‐malononitrile is a synthetically useful electron‐deficient functional group (Figure 2). Both Zanardi's^[^ ^40^, ^41^, ^42^ ^]^ and Chen's^[^ ^43^ ^]^ groups have developed several asymmetric reactions utilizing reagents containing this motif. For example, Chen and coworkers reported the Michael reaction between naphthalenylidenepropanedinitrile and crotonaldehyde, catalyzed by diphenylprolinol methyl ether, producing the *syn*‐product enantioselectively (Scheme 2 (Eq. 7)).^[^ ^44^ ^]^

**Figure 2:**
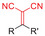
2‐Methylidene‐malononitrile moiety.

Motivated by our interest in domino reactions, we designed 2,2′‐(cyclohexane‐1,4‐diylidene)dimalononitrile (**1**), a reagent containing both nucleophilic and electrophilic moieties, with the aim of developing a domino Michael/Michael reaction to synthesize a bicyclo[3.2.1]octane derivative (Scheme 2 (Eq. 8)). During this study, we unexpectedly obtained a noradamantane derivative, which is detailed in this paper.

We selected the reaction between 2,2′‐(cyclohexane‐1,4‐diylidene)dimalononitrile (**1**) and cinnamaldehyde (**2a**) as a model and investigated it in the presence of diphenylprolinol diphenylmethylsilyl ether^[^ ^45^ ^]^ and benzoic acid in THF (Table 1, entry 1). The reaction proceeded smoothly, yielding noradamantanes **3a** and **3a’** with a total yield of 88% (dr = 6:1). The major isomer, **3a**, exhibited excellent enantioselectivity (97% ee). These products were unexpected: in a single reaction step, three carbon–carbon bonds were formed, and six chiral centers, including one all‐carbon quaternary center, were generated, five of which were fully controlled. Given the lack of synthetic methods for chiral and functionalized noradamantanes, we decided to investigate this reaction in detail.

**Table 1:** Effect of solvent, acid, and additive in the reaction of **1** and cinnamaldehyde (**2a**).

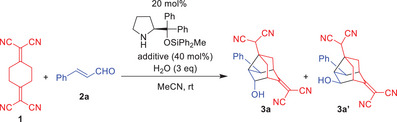						
Entry^a)^	solvent	additive	Time [h]	Yield^b)^ [%]	**3a**:**3a'**	*ee* ^c)^ [%]
1	THF	PhCO_2_H	4	88	6:1	97
2	CH_2_Cl_2_	PhCO_2_H	13	74	2:1	nd
3	MeCN	PhCO_2_H	3	81	5:1	99
4^d)^	MeCN	PhCO_2_H	70	82	8:1	nd
5^e)^	MeCN	PhCO_2_H	75	40	4:1	96
6	MeCN	*p*‐nitrophenol	48	77	nd	nd
7	MeCN	CF_3_CO_2_H	48	0	–	–
8	MeCN	Et_3_N	1.5	28	5:1	nd

^a)^ Unless otherwise shown, the reaction was performed by employing **1** (0.30 mmol), cinnamaldehyde (**2a**) (0.33 mmol), organocatalyst (20 mol%), indicated additive (0.12 mmol), and water (0.90 mmol) in indicated solvent (6.0 mL).

^b)^ Isolated yield of a diastereomer mixture.

^c)^ Determined by HPLC analysis on a chiral column material after conversion of alcohol to acetate using acetic anhydride and a catalytic amount of scandium(III) triflate (See Supporting Information for details).

^d)^ Organocatalyst (10 mol%) was used.

^e)^ Diphenylprolinol trimethylsilyl ether (20 mol%) was used.

To optimize the reaction conditions, we first screened solvents. Due to the poor solubility of **1** in toluene and Et_2_O, low yields were obtained in these solvents. Although the reaction was slow in CH_2_Cl_2_ (entry 2), it proceeded smoothly in MeCN, affording nearly optically pure noradamantane in good yield (entry 3).

Next, we examined the effect of additives. The reaction proceeded with a weak acid, such as *p*‐nitrophenol, but it was slow (entry 6). Trifluoroacetic acid proved too strong and inhibited the reaction (entry 7). Using Et_3_N as an additive allowed the reaction to proceed, with the starting material disappearing within 1.5 h, but the product yield was low (entry 8).

When the catalyst loading was reduced to 10 mol%, the reaction slowed but still afforded the product in good yield (entry 4). However, replacing diphenylprolinol diphenylmethylsilyl ether with diphenylprolinol trimethylsilyl ether as the catalyst reduced the reaction rate and yield (entry 5). Based on these results, we selected diphenylprolinol diphenylmethylsilyl ether as the catalyst and MeCN and benzoic acid as the solvent and additive, respectively, for further studies.

We investigated several α,β‐unsaturated aldehydes (Table 2). Regarding the β‐substituents of α,β‐unsaturated aldehydes, not only phenyl and naphthyl groups but also electron‐rich aryl groups, such as *p*‐methoxyphenyl, *m*‐methoxyphenyl, and *o*‐methoxyphenyl, proved suitable for affording the products in good yields with excellent enantioselectivity. Electron‐deficient substituents, including *p*‐bromophenyl, *p*‐trifluoromethylphenyl, and *p*‐nitrophenyl, were also successfully employed, providing products with excellent enantioselectivity. Heteroaromatic groups, such as furyl, were equally effective. Furthermore, ortho‐substituents on the phenyl ring, such as *o*‐methoxyphenyl, did not interfere with the reaction, yielding the products with excellent selectivity. Aliphatic α,β‐unsaturated aldehydes did not react under these conditions as no reaction was observed with crotonaldehyde even after 3 days.

**Table 2:** The generality of the reaction of **1** and α,β‐unsaturated aldehyde **2** (see footnote).

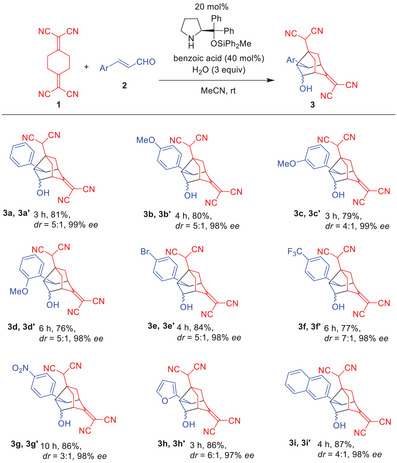

^a)^ Unless otherwise shown, the reaction was conducted using **1** (0.30 mmol), **2** (0.33 mmol), catalyst (0.06 mmol), benzoic acid (0.12 mmol), and water (0.9 mmol) in MeCN (6.0 mL) at room temperature under argon atmosphere for the indicated time. Yields were isolated yield. The diastereomer ratio was determined by ^1^H‐NMR analysis. Enantiomeric excess (*ee*) was determined by HPLC analysis on a chiral column material of the corresponding acetate after separation of the diastereomers. See Supporting Information for more details.

The absolute configuration of the noradamantane derivative **3c** was elucidated using vibrational circular dichroism (VCD)^[^ ^46^, ^47^, ^48^ ^]^ of acetate **4c**. A mixture of **3c** and **3c’** was acetylated with acetic anhydride and scandium(III) triflate^[^ ^49^ ^]^ to afford a mixture of **4c** and **4c’** (Figure 3 (Eq. 9)). Compound **4c** was then separated from the mixture.

**Scheme 3:**
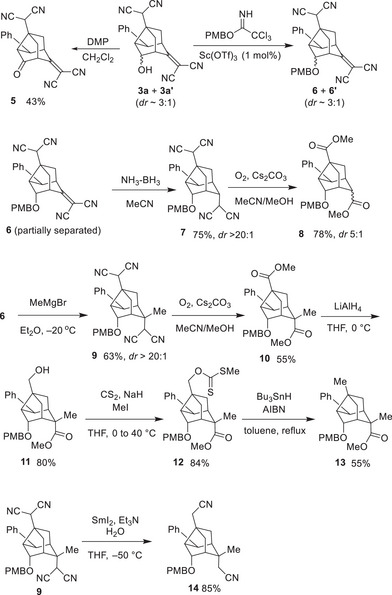
Synthetic transformations to afford derivatized noradamantane compounds.

Figure 3 shows the experimental and theoretical spectra of **4c**. Most of the peaks observed in the VCD spectra corresponded one‐to‐one with those in the calculated spectra, including their signs. The strong overall agreement between the experimental and theoretical spectra unambiguously confirmed the absolute configuration of **4c**. Consequently, the absolute configuration of the parent compound **3c** was determined as shown in Figure 3 (Eq. 9).

**Figure 3:**
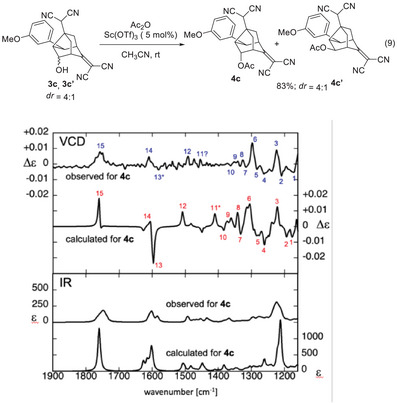
Comparison of the observed and calculated spectra from VCD (top) and IR (bottom) analysis of **4c**.

The obtained noradamantane derivatives possess several functional groups, including an aryl group, a hydroxy group, a malononitrile group, and a methylene malononitrile moiety. Next, derivatization of the product **3a** was investigated (Scheme 3). The diastereomeric mixture of alcohols **3a** and **3a’** was oxidized using Dess‐Martin periodinane (DMP) to yield ketone **5**. The mixture was also converted into the corresponding *para*‐methoxybenzyl (PMB) ethers to obtain the corresponding PMB ethers **6** and **6′** by treatment with a trichloroacetamidate derivative in the presence of a catalytic amount of Sc(OTf)_3_.^[^ ^50^ ^]^ The 2‐methylene‐malononitrile moiety of **6** (obtained in pure form by partial separation) was reduced with NH_3_·BH_3_^[^ ^51^ ^]^ to afford bis‐malononitrile compound **7** in good yield and with excellent diastereoselectivity. The transformation of the malononitrile moiety into the corresponding methoxycarbonyl group was achieved via a reaction with molecular oxygen in the presence of Cs_2_CO_3_, a reaction developed in our group.^[^ ^52^, ^53^ ^]^ This produced bis‐methyl ester **8** in good yield. When **6** was treated with MeMgBr, a stereoselective 1,4‐addition reaction proceeded with excellent diastereoselectivity, generating a new all‐carbon quaternary center to afford **9**. The synthesis of bis‐methyl ester **10** was also successfully achieved using our oxidative method. One of these methyl esters was selectively reduced using LiAlH_4_ to obtain the primary alcohol **11**, which was then converted into the corresponding xanthate **12**. Treatment of **12** under typical Barton‐McCombie conditions provided the deoxygenated product **13**.^[^ ^54^ ^]^ Finally, treatment of **9** with an excess of SmI_2_ resulted in a successful reduction of the two malononitrile units into the primary bis‐nitrile **14**.^[^ ^55^, ^56^ ^]^ This sequence of transformations showcases the functionalization of these molecules in a 3D fashion, making it highly versatile for further derivatization.

Following our success with the reaction of 2,2′‐(cyclohexane‐1,4‐diylidene)dimalononitrile (**1**), we next investigated the reaction of 2‐(4‐oxocyclohexylidene)malononitrile (**15**). When the reaction of **15** and cinnamaldehyde (**2a**) was performed in the presence of diphenylprolinol silyl ether, benzoic acid, and water in diethyl ether at room temperature, a diastereomeric mixture of **16a** and **16a’** (dr = 4:1) was obtained in 93% yield with very high enantioselectivity (96% ee for the major diastereomer) (Eq. 10).^[^ ^57^ ^]^ The substituted noradamantane skeleton was again constructed from simple starting materials in a single operation. Optimization of the reaction conditions, including solvent and additives, indicated that the best solvent and additive were Et_2_O and benzoic acid, respectively.


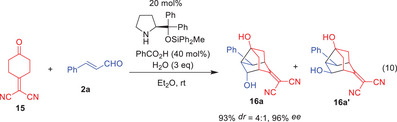


The generality of the reaction was investigated (Table 3). Regarding the β‐substituent of α,β‐unsaturated aldehydes, phenyl groups, as well as electron‐rich phenyl groups such as *para*‐methoxyphenyl, were found to be suitable, affording the products in good yields with moderate diastereoselectivity and excellent enantioselectivity, although the reaction was slow. In reactions of α,β‐unsaturated aldehydes possessing β‐phenyl groups with electron‐deficient substituents, such as *para*‐fluoro, *para*‐chloro, *para*‐bromo, *meta*‐bromo, and *ortho*‐bromo, the reaction was faster, affording the products in good yields with high diastereoselectivity and excellent enantioselectivity. Heteroaryl groups, such as a furyl‐substituted α,β‐unsaturated aldehyde, also provided good enantioselectivity.

**Table 3:** The generality of the reaction of **15** and α,β‐unsaturated aldehyde **2** (See footnote).

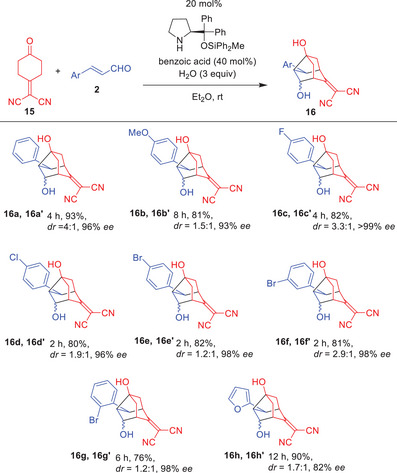

^a)^ Unless otherwise shown, the reaction was conducted using **15** (0.25 mmol), **2** (0.30 mmol), catalyst (0.05 mmol), benzoic acid (0.10 mmol), and water (0.75 mmol) in Et_2_O (0.6 mL) at room temperature under argon atmosphere for the indicated time. Yields were isolated yield. The diastereomer ratio was determined by ^1^H‐NMR analysis. Enantiomeric excess (*ee*) was determined by HPLC analysis on a chiral column material of the corresponding *p*‐bromobenzoate after separation of the diastereomers. See Supporting Information for more details.^[^ ^58^ ^]^

Whereas noradamantane derivatives were obtained with β‐aryl‐substituted α,β‐unsaturated aldehydes, β‐alkyl‐ or silyl‐substituted α,β‐unsaturated aldehydes afforded different products. When we employed 3‐(dimethylphenylsilyl)‐propenal (**2i**) and 5‐phenylpro‐2‐enal (**2j**) as α,β‐unsaturated aldehydes, the reaction proceeded to afford bicyclo[3.2.1]octane derivatives, which were characterized after conversion to α,β‐unsaturated esters **17i** and **17j** using the Wittig reagent (Ph₃P = CHCO₂Et) (Eq. 11).

Compounds **17i** and **17j** were obtained in good yields with excellent enantioselectivity. These compounds are derived from a domino Michael/aldol reaction, as described below. This type of reaction was anticipated when we started this project (cf., Scheme 2 (Eq. 8)). The detailed reaction mechanism will be discussed next.


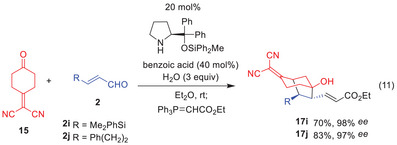


We first explain the reaction of compound **15**. The initial step is a Michael reaction involving an iminium ion, generated by the reaction of an α,β‐unsaturated aldehyde with diphenylprolinol silyl ether (Scheme 4). Both cyclohexanone and cyclohexylidenemalononitrile are known to be good Michael donors in diphenylprolinol ether‐mediated Michael reactions (Scheme 2 (eqs. 5, 7)). The 2‐methylidene‐malononitrile moiety is more reactive than the former, based on previous work by Chen (Scheme 2 (Eq. 7)). The first Michael reaction would yield the *syn*‐adduct *syn*‐**18**, which would undergo an intramolecular aldol reaction in a domino fashion, producing bicyclo[3.2.1]octane derivatives **17**. In fact, when the β‐substituent of acrolein (R in Scheme 4) is dimethylphenylsilyl or 3‐phenylpropyl, the products **17i** and **17j** are obtained (Eq. 11). This is the expected outcome for this reaction, as anticipated in the design of the project.

**Scheme 4:**
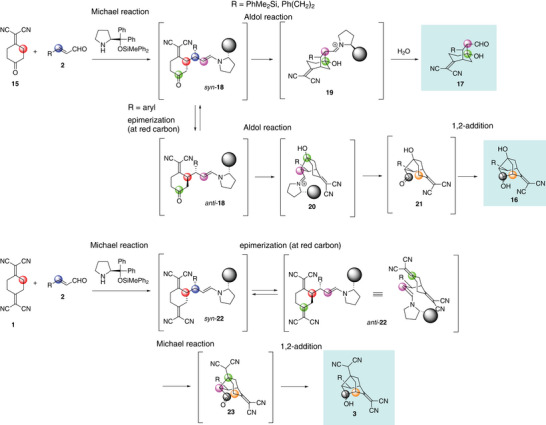
The reaction mechanism of the generation of bicyclo[3.2.1]octane derivatives **17** and noradamantanes **3**, **16**.

However, when the β‐substituent of acrolein is an aryl group (R = aryl in Scheme 4), steric hindrance in the intramolecular aldol reaction (*syn*‐**18** → **19**) prevents it from proceeding. Due to the strongly electron‐withdrawing methylene‐malononitrile moiety, the C2 position of cyclohexylidene undergoes easy epimerization, leading to an equilibration between *syn*‐**18** and *anti*‐**18**. The intramolecular aldol reaction proceeds from *anti*‐**18**, affording iminium ion **20**. After hydrolysis, aldehyde **21** is formed. The proximity of the formyl group at C2 and C1 (noradamantane numbering) facilitates a successive addition reaction, leading to the formation of the noradamantane derivative **16**. Thus, **16** is generated via the domino Michael/epimerization/aldol/1,2‐addition sequence.

Similarly, the reaction of **1** would proceed through a domino Michael/epimerization/Michael/1,2‐addition mechanism. The Michael reaction first generates *syn*‐**22**, from which the subsequent Michael reaction does not proceed due to steric hindrance. Epimerization then occurs, yielding *anti*‐**22**. An intramolecular Michael reaction follows from *anti*‐**22** to provide **23**, and finally, a 1,2‐addition reaction takes place to afford the product **3**.

In summary, we have developed a new synthetic method to produce topologically unique, chiral noradamantanes incorporating several functional groups. These compounds are synthesized from readily available starting materials, including 2,2′‐(cyclohexane‐1,4‐diylidene)dimalononitrile (**1**) and 2‐(4‐oxocyclohexylidene)malononitrile (**15**), via reactions with β‐aryl α,β‐unsaturated aldehydes. The process employs a domino reaction catalyzed by diphenylprolinol silyl ether and follows either an asymmetric catalytic Michael/epimerization/Michael/1,2‐addition sequence or an asymmetric catalytic Michael/epimerization/aldol/1,2‐addition sequence. In a single reaction step, this method enables the synthesis of complex and functionalized noradamantanes with excellent enantioselectivity. It forms three carbon–carbon bonds and generates six chiral centers, including one all‐carbon quaternary center, with five of these chiral centers fully controlled. As 3D, cage‐like molecules with several functional groups, these compounds hold significant potential as valuable chiral building blocks for drug design.

## Conflict of Interests

The authors declare no conflict of interest.

## Supporting information



Supporting Information

## Data Availability

The data that support the findings of this study are available in the supplementary material of this article.
