# Supplementary material for: Asymmetric Synthesis of Noradamantane Scaffolds via Diphenylprolinol Silyl Ether‐Mediated Domino Michael/Epimerization/Michael (or Aldol)/1,2‐Addition Reactions
Source: Angew Chem Int Ed Engl. 2025 Apr 11;64(23):e202500378. doi: 10.1002/anie.202500378 (PMC12124435; doi:10.1002/anie.202500378)
Supplement: Supplementary file 1 — Supporting Information [file ANIE-64-e202500378-s001.pdf]

Asymmetric Synthesis of Noradamantane Scaffolds via Diphenylprolinol Silyl Ether-Mediated Domino Michael/Epimerization/Michael (or Aldol)/1,2-Addition Reactions

Konstantinos Daskalakis,[‡] Nariyoshi Umekubo,[‡ f] Satrajit Indu, Genki Kawauchi, Tohru Taniguchi,[†] Kenji Monde,[†] Yujiro Hayashi*

Department of Chemistry, Graduate School of Science, Tohoku University, 6-3 Aramaki Aza-Aoba, Aoba-ku, Sendai, Miyagi 980-8578, Japan

† Frontier Research Center of Advanced Material and Life Science, Faculty of Advanced Life Science, Hokkaido University, Sapporo 060-0810, Japan

Index

1.	Materials and Methods	SI-3
2.	General Procedure A for the formation of compounds **3**	SI-3
3.	General Procedure B for the formation of compounds **4** and **4'**	SI-9
4.	General Procedure C for the formation of compounds **16**	SI-32
5.	NMR spectral data	SI-45
6.	IR spectral data	SI-114
7.	HPLC data	SI-169

1. Materials and Methods

General Remarks: All reactions were carried out under air atmosphere (unless otherwise is specified) and monitored by thin-layer chromatography using Merck 60 F_{254} precoated silica gel plates (0.25 mm thickness). Specific optical rotations were measured using a JASCO P-1020 polarimeter and a JASCO DIP-370 polarimeter. FT-IR spectra were recorded on a JASCO FT/IR-410. ^{1}H, ^{13}C and ^{19}F NMR spectra were recorded on an Agilent-400 MR (400 MHz for ^{1}H NMR, 100 MHz for ^{13}C NMR, 376 MHz for ^{19}F NMR) instrument. Data for ^{1}H NMR are reported as chemical shift (δ ppm), integration multiplicity (s = singlet, d = doublet, t = triplet, q = quartet, quint = quintet, dd = doublet of doublet, ddd = doublet of doublet of doublet, dt = doublet of triplet, td = triplet of doublet, dquint = doublet of quintet, m = multiplet, br s = broad singlet), coupling constant (Hz). Reference peaks for ^{1}H NMR are: $CDCl_3$ = 7.26 ppm, CD_3CN = 1.96 ppm, $(CD_3)_2CO$ = 2.05 ppm. Data for ^{13}C NMR are reported as chemical shift. Reference peaks for ^{13}C NMR are: $CDCl_3$ = 77.00 ppm, CD_3CN = 118.26, $(CD_3)_2CO$ = 29.84 ppm. Data for ^{19}F NMR are reported as chemical shift. Reference peaks for ^{19}F NMR are: C_6H_5F = -113.15 ppm (added to the sample as internal standard). High resolution ESI-TOF mass spectra were measured by Themo Orbi-trap instrument. HPLC analysis was performed on a HITACHI Elite LaChrom Series HPLC, UV detection monitored at appropriate wavelength respectively, using Chiralpak IB (0.46 cm × 25 cm), Chiralpak IG (0.46 cm × 25 cm) and Chiralpak AD-H (0.46 cm × 25 cm).

2. General procedure A for the formation of compounds 3

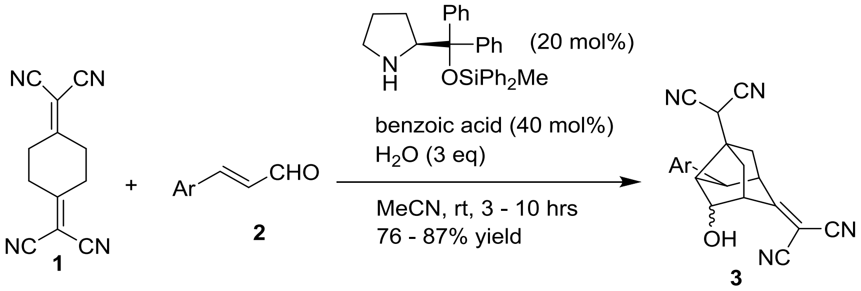

A solution of the respective aldehyde **2** (0.33 mmol, 1.1 equiv.), 2,2'-(cyclohexane-1,4-diylidene)dimalononitrile **1** (62.5 mg, 0.30 mmol), water (16 µL, 0.9 mmol, 3.0 equiv.), benzoic acid (15 mg, 0.12 mmol, 0.4 equiv.) and (*S*)-diphenylprolinoldiphenylmethylsilyl ether (27 mg, 0.06 mmol, 0.2 equiv.) in 6 mL of MeCN (0.05 M) was stirred at room temperature. The progress of the reaction was monitored by both, Thin Layer Chromatography and ^{1}H NMR. After completion, the reaction solvent was removed *in vacuo*. The raw product was then purified using column chromatography (Biotage Isolera One – Sfär HC D Silica, Hexane to Hexane:Ethyl Acetate = 3:1) to obtain the products **3** as an inseparable mixture of two diastereoisomers. (Notice: Partial epimerization from the major to the minor product is possible to occur upon prolonged exposure to silica gel leading to depleted diastereomeric ratios of major : minor 2:1)

2-((2*R*,3*aR*,5*R*,6*S*,6*aR*)-3*a*-(dicyanomethyl)-1-hydroxy-6-phenyloctahydro-2,5-methanopentalen-7-ylidene)malononitrile (3a, 3a')

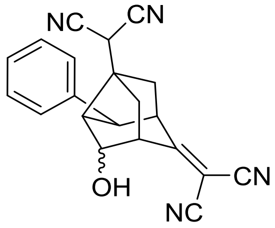

Was prepared following General procedure A. 83 mg (81%, 0.24 mmol) obtained as diastereomeric mixture (dr ~5:1, inseparable). Isolated as a yellow foam. R_f = 0.6 (hexane : ethyl acetate 1:1)

For the gram scale synthesis: A solution of cinnamaldehyde (700 mg, 5.3 mmol, 1.1 equiv.), compound 3 (1.00 g, 4.8 mmol), water (260 µL, 14.4 mmol, 3.0 equiv.), benzoic acid (234 mg, 1.92 mmol, 0.4 equiv.) and (*S*)-diphenylprolinoldiphenylmethylsilyl ether (430 mg, 0.96 mmol, 0.2 equiv.) in 32 mL of MeCN (0.05 M) was stirred at room temperature. After completion (TLC, 3 h), the reaction solvent was removed *in* vacuo. The raw product was then purified using column chromatography (Biotage Isolera One, Hexane to Hexane:Ethyl Acetate = 3:1) to obtain 1.5 g of product (3.8 mmol) as a diastereomeric mixture (*dr* 5:1, inseparable) in 79% of yield.

^{1}H NMR (400 MHz, $CDCl_3$): δ 7.43 – 7.38 (m, 2.4 Hz, 2H), 7.33 – 7.28 (m, 1H), 7.19 – 7.14 (m, 2H), 4.69 (dd, *J* = 6.5, 3.3 Hz, 5/6H), 4.29 (br s, 1/6H), 4.23 (br s, 5/6H), 3.97 (s, 1/6H), 3.93 (s, 5/6H), 3.88 (br s, 5/6H), 3.84 (br s, 1/6H), 3.67 (br s, 1/6H), 3.60 (br s, 5/6H), 3.26 (br s, 1/6H), 3.06 (d, *J* = 6.6 Hz, 5/6H), 2.87 – 2.61 (m, 1H), 2.74 (dt, *J* = 16.6, 3.2 Hz, 1/6H), 2.35 (dt, *J* = 12.3, 3.4 Hz, 5/6H), 2.22 – 2.19 (m, 1/6H), 2.15 (dt, *J* = 12.0, 3.6 Hz, 5/6H), 2.11 – 2.09 (m, 1/6H), 2.06 (br s, 1/6H), 2.02 (d, *J* = 11.8 Hz, 5/6H), 1.96 (d, *J* = 12.0 Hz, 1/6H), 1.88 (d, *J* = 11.6 Hz, 5/6H) ppm.

^{13}C NMR (100 MHz, $CDCl_3$): δ 181.1, 178.2, 138.5, 135.5, 129.4, 129.3, 127.5, 126.2, 126.2, 111.3, 111.2, 110.8, 110.3, 84.6, 81.4, 77.5, 53.3, 50.4, 49.8, 49.6, 49.4, 49.2, 48.8, 47.8, 43.3, 42.4, 40.1, 30.4, 30.1 ppm

HRMS (ESI) *m/z*: calcd for $C_{21}H_{16}N_4NaO$: 363.1216 $[M+Na]^+$; found: 363.1220.

FT-IR (neat): *v* 3498, 3063, 3028, 2968, 2900, 2232, 1600, 1498, 1476, 1451, 1303, 1126, 1094, 910 cm^{-1}.

2-((2*R*,3*aR*,5*R*,6*S*,6*aR*)-3*a*-(dicyanomethyl)-1-hydroxy-6-(4-methoxyphenyl)octahydro-2,5-methanopentalen-7-ylidene)malononitrile (3b, 3b')

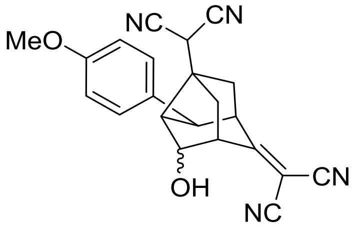

Was prepared following General procedure A. 90 mg (80%, 0.24 mmol) obtained as diastereomeric mixture (*dr* ~5:1, inseparable). Isolated as a yellow oil. R_f = 0.6 (hexane : ethyl acetate 1:1)

^{1}H NMR (400 MHz, $CDCl_3$): δ 7.10 – 7.04 (m, 2H), 6.93 – 6.89 (m, 2H), 4.64 (dd, *J* = 6.7, 3.2 Hz, 5/6H), 4.24 (br s, 1/6H), 4.16 (br s, 5/6H), 3.98 (s, 1/6H), 3.95 (s, 5/6H), 3.80 (s, 3H + 5/6H), 3.75 (br s, 1/6H), 3.62 (br s, 1/6H), 3.56 (br s, 5/6H), 3.19 (br s, 1/6H), 3.04 – 3.02 (m, 1/6H), 2.98 (d, *J* = 6.6 Hz, 1H), 2.31 (dt, *J* = 12.0, 3.3 Hz, 5/6H), 2.17 – 2.09 (m, 1H), 2.03 – 1.98 (m, 1H), 1.92 (d, *J* = 11.7 Hz, 1/6 H), 1.86 (d, *J* = 12.0 Hz, 5/6H) ppm.

^{13}C NMR (100 MHz, $CDCl_3$): δ 181.6, 180.2, 158.9, 158.6, 130.3, 128.4, 127.3, 127.3, 116.3, 114.7, 114.6, 111.4, 111.3, 110.9, 110.4, 84.3, 83.3, 81.3, 77.5, 55.4, 52.9, 52.7, 51.6, 50.7, 49.7, 49.4, 49.1, 49.0, 48.8, 47.9, 43.9, 43.2, 42.3, 40.1, 38.4, 30.4, 30.1 ppm.

HRMS (ESI) *m/z*: calcd for $C_{22}H_{18}N_4NaO_2$: 393.1322 $[M+Na]^+$; found: 393.1316.

FT-IR (neat): ν 3491, 2964, 2904, 2232, 1596, 1511, 1466, 1250, 1179, 1031, 910 cm^{-1}.

<u>2-((2*R*,3*aR*,5*R*,6*S*,6*aR*)-3*a*-(dicyanomethyl)-1-hydroxy-6-(3-methoxyphenyl)octahydro-2,5-methanopentalen-7-ylidene)malononitrile</u> (3c, 3c')

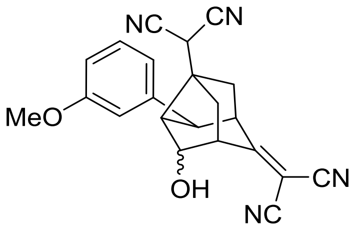

Was prepared following General procedure A. 88 mg (79%, 0.24 mmol) obtained as diastereomeric mixture (*dr* ~4:1, inseparable). Isolated as a yellow oil. R_f = 0.6 (hexane : ethyl acetate 1:1)

^{1}H NMR (400 MHz, $CDCl_3$): δ 7.33 – 7.30 (m, 1H), 6.85 – 6.81 (m, 1H), 6.74 – 6.67 (m, 2H), 4.69 (dd, *J* = 6.8, 3.4 Hz, 4/5 H), 4.27 (br s, 1/5 H), 4.19 (br s, 4/5 H), 3.97 (br s, 1/5 H), 3.94 (br s, 4/5 H), 3.86 (br s, 4/5H), 3.82 (s, 3H), 3.66 (br s, 1/5 H), 3.60 (br s, 4/5 H), 3.22 (br s, 1/5 H), 3.02 (d, *J* = 6.5 Hz, 1H), 2.81 – 2.76 (m, 1/5 H), 2.34 (dt, *J* = 12.1, 3.4 Hz, 1H), 2.16 – 2.12 (m, 1H), 2.03 (d, *J* = 11.6 Hz, 1H), 1.95 (d, *J* = 11.8 Hz, 1/5 H), 1.88 (d, *J* = 12.0 Hz, 4/5 H) ppm.

^{13}C NMR (100 MHz, $CDCl_3$): δ 180.9, 160.3, 140.3, 138.3, 130.5, 130.4, 118.3, 113.2, 116.2, 111.3, 111.2, 110.8, 110.3, 84.7, 81.4, 77.4, 53.3, 53.3, 53.0, 50.5, 49.6, 49.4, 49.1, 47.8, 43.4, 42.3, 40.0, 30.4, 30.2 ppm.

HRMS (ESI) *m/z*: calcd for $C_{22}H_{18}N_4NaO_2$: 393.1322 $[M+Na]^+$; found: 393.1318.

FT-IR (neat): ν 3469, 2236, 1631, 1604, 1490, 1430, 1267, 758 cm^{-1}.

2-((2*R*,3*aR*,5*R*,6*S*,6*aR*)-3*a*-(dicyanomethyl)-1-hydroxy-6-(2-methoxyphenyl)octahydro-2,5-methanopentalen-7-ylidene)malononitrile **(3d, 3d')**

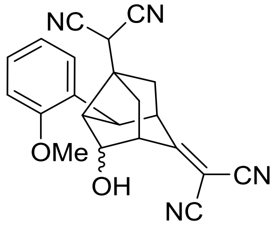

Was prepared following General procedure A. 84 mg (76%, 0.23 mmol) obtained as diastereomeric mixture (*dr* ~5:1, inseparable). Isolated as a light brown foam. R_f = 0.6 (hexane : ethyl acetate 1:1)

^{1}H NMR (400 MHz, $CDCl_3$): δ 7.35 – 7.29 (m, 1H), 7.02 – 6.90 (m, 13/6H), 6.83 (d, *J* = 7.4 Hz, 5/6H), 4.68 (dd, *J* = 6.5, 3.1 Hz, 5/6H), 4.31 (br s, 5/6H), 4.29 (br s, 1/6H), 4.13 (br s, 5/6H), 3.94 (br s, 5/6H), 3.90 (s, 15/6H), 3.89 (s, 3/6H), 3.87 (br s, 1/6H), 3.65 (br s, 1/6H), 3.57 (br s, 5/6H), 3.29 (br s, 1/6H), 3.07 (dt, *J* = 11.8, 3.6 Hz, 1/6H), 2.96 (d, *J* = 6.6 Hz, 1H), 2.74 (br s, 1H), 2.35 (dt, *J* = 12.0, 3.6 Hz, 5/6H), 2.07 – 1.98 (m, 13/6H), 1.92 (d, *J* = 12.0 Hz, 1/6H), 1.85 (d, *J* = 11.5 Hz, 5/6H) ppm.

^{13}C NMR (100 MHz, $CDCl_3$): δ 181.5, 180.3, 157.0, 129.3, 129.0, 126.7, 126.6, 125.1, 120.5, 120.2, 111.5 (2C), 111.1, 110.9, 110.4, 84.2, 82.1, 78.0, 55.2, 50.5, 49.7, 49.7, 49.5, 49.0, 46.9, 46.6, 43.4, 42.1, 39.9, 30.3, 30.1 ppm.

HRMS (ESI) *m/z*: calcd for $C_{22}H_{18}N_4NaO_2$: 393.1322 $[M+Na]^+$; found: 393.1330.

FT-IR (neat): v 3488, 2960, 2907, 2232, 1596, 1490, 1243, 1116, 1027, 906, 730 cm^{-1}.

2-((1*S*,2*R*,3*aR*,5*R*,6*aR*)-1-(4-bromophenyl)-3a-(dicyanomethyl)-6-hydroxyoctahydro-2,5-methanopentalen-7-ylidene)malononitrile **(3e, 3e')**

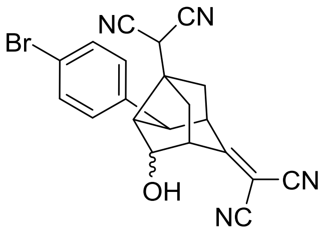

Was prepared following General procedure A. 106 mg (84%, 0.25 mmol) obtained as diastereomeric mixture (*dr* ~5:1, inseparable). Isolated as a yellow foam. R_f = 0.6 (hexane : ethyl acetate 1:1)

^{1}H NMR (400 MHz, $CDCl_3$): δ 7.53 (d, *J* = 8.3 Hz, 2H), 7.06 (d, *J* = 8.2 Hz, 2H), 4.70 (dd, *J* = 6.6, 3.1 Hz, 5/6H), 4.30 (br s, 1/6H), 4.17 (br s, 5/6H), 3.93 (br s, 1/6H), 3.87 (br s, 10/6H), 3.83 (br s, 1/6H), 3.67 (br s, 1/6H), 3.61 (br s, 5/6H), 3.19 (br s, 1/6H), 3.06 – 2.99 (m, 1H), 2.73 (br s, 5/6H), 2.51 (br s, 1/6H),

2.34 (dt, J = 12.0, 3.4 Hz, 1H), 2.14 (dt, J = 11.8, 3.6 Hz, 1H), 2.07 – 1.97 (m, 7/6H), 1.91 (d, J = 12.2 Hz, 5/6H) ppm.

^{13}C NMR (100 MHz, $CDCl_3$): δ 180.4, 137.5, 132.5, 132.4, 127.9, 121.4, 111.2, 111.1, 110.8, 110.3, 84.9, 77.3, 50.0, 49.9, 49.6, 49.1, 48.8, 48.1, 43.3, 40.1, 30.4 ppm.

HRMS (ESI) *m/z*: calcd for $C_{21}H_{15}BrN_4NaO$: 441.0321, 443.0301 $[M+Na]^+$; found: 441.0331, 443.0301.

FT-IR (neat): ν 3491, 2971, 2897, 2236, 1596, 1494, 1010, 906, 730 cm^{-1}.

<u>2-((2*R*,3*aR*,5*R*,6*S*,6*aR*)-3*a*-(dicyanomethyl)-1-hydroxy-6-(4-(trifluoromethyl)phenyl)octahydro-2,5-methanopentalen-7-ylidene)malononitrile</u> (3f, 3f')

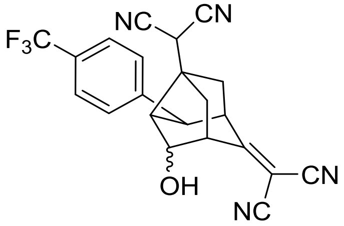

Was prepared following General procedure A. 95 mg (77%, 0.23 mmol) obtained as diastereomeric mixture (*dr* ~7:1, inseparable). Isolated as a yellow oil. R_f = 0.6 (hexane : ethyl acetate 1:1)

^{1}H NMR (400 MHz, $CDCl_3$): δ 7.67 (δ, J = 8.1 Hz, 2H), 7.32 (d, J = 8.0 Hz, 2H), 4.72 (dd, J = 6.2, 3.1 Hz, 7/8 H), 4.33 (br s, 1/8 H), 4.28 (br s, 7/8 H), 3.96 (br s, 1/8H), 3.94 (br s, 7/8 H), 3.88 (s, 1H), 3.69 (br s, 1/8H), 3.63 (br s, 7/8H), 3.28 (br s, 1/8H), 3.12 – 3.02 (m, 7/8H + 2/8H), 2.84 (br s, 1H), 2.36 (dt, J = 12.1, 3.2 Hz, 7/8H), 2.14 (dt, J = 12.0, 3,3 Hz, 7/8H), 2.09 – 1.99 (m, 7/8H + 3/8H), 1.93 (d, J = 12.0 Hz, 7/8H) ppm.

^{13}C NMR (100 MHz, $CDCl_3$): δ 180.1, 178.8, 142.6, 140.5, 129.9 (q, J = 32.7 Hz), 127.8, 126.7, 126.7, 126.3 (J = 3.7 Hz), 123.8 (q, J = 273.3 Hz), 111.1, 111.1, 110.7, 110.3, 85.0, 81.4, 77.3, 77.2, 53.1, 53.0, 51.6, 50.0, 49.7, 49.7, 49.2, 49.1, 48.3, 48.2, 43.3, 43.1, 42.6, 40.2, 30.4, 30.1 ppm.

^{19}F NMR (376 MHz, $CDCl_3$): δ -62.7 (s, 21/8F), -62.7 (s, 3/8F) ppm.

HRMS (ESI) *m/z*: calcd for $C_{22}H_{15}F_3N_4NaO$: 431.1090 $[M+Na]^+$; found: 431.1075.

FT-IR (neat): ν 3494, 2968, 2904, 2236, 1617, 1600, 1415, 1328, 1169, 1122, 1069, 914 cm^{-1}.

<u>2-((2*R*,3*aR*,5*R*,6*S*,6*aR*)-3*a*-(dicyanomethyl)-1-hydroxy-6-(4-nitrophenyl)octahydro-2,5-methanopentalen-7-ylidene)malononitrile</u> (3g, 3g')

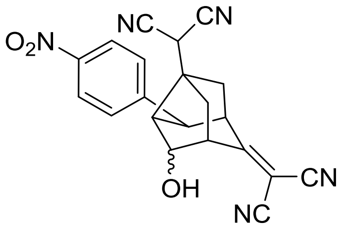

Was prepared following General procedure A. 101 mg (86%, 0.26 mmol) obtained as diastereomeric mixture (*dr* ~3:1, inseparable). Isolated as a yellow oil. R_f = 0.5 (hexane : ethyl acetate 1:1)

^{1}H NMR (400 MHz, $CDCl_3$): δ 8.22 – 8.16 (m, 2H), 7.55 – 7.50 (m, 2H), 4.60 – 4.55 (m, 3/4H), 4.39 (s, 1H), 4.26 (br s, 1/4H), 4.23 (br s, 3/4H), 4.13 (br s, 3/4H), 3.94 (br s, 3/4H), 3.83 (br s, 1/4H), 3.80 (br s, 1/4H), 3.49 (br s, 1H), 3.42 (s, 1/4H), 3.05 (d, *J*= 6.4 Hz, 3/4H), 3.02 (br s, 1/4H), 2.90 (dt, *J* = 11.6, 3.7 Hz, 1/4H), 2.25 (dt, *J* = 12.1, 3.5 Hz, 3/4H), 2.17 (dt, *J* = 12.1, 3.4 Hz, 3/4H), 2.01 – 1.93 (m, 6/4H + 3/4H) ppm.

^{13}C NMR (100 MHz, $CDCl_3$): δ 182.8, 180.7, 148.5, 147.6, 146.8, 129.2, 129.1, 124.6, 124.6, 113.1, 112.9, 112.6, 112.2, 84.7, 81.7, 77.9, 54.2, 53.5, 54.2, 53.5, 52.7, 50.4, 50.3, 50.0, 49.8, 48.9, 43.5, 42.9, 41.1, 30.8, 30.7 ppm.

HRMS (ESI) *m/z*: calcd for $C_{21}H_{15}N_5NaO_3$: 408.1067 $[M+Na]^+$; found: 408.1071.

FT-IR (neat): ν 3494, 3021, 2971, 2904, 2236, 1635, 1604, 1519, 1345, 1214, 850, 755 cm^{-1}.

<u>2-((1*S*,2*R*,3*aR*,5*R*,6*aR*)-3*a*-(dicyanomethyl)-1-(furan-2-yl)-6-hydroxyoctahydro-2,5-methanopentalen-7-ylidene)malononitrile</u> (3h, 3h')

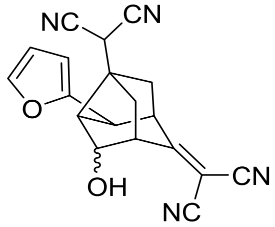

Was prepared following General procedure A. 85 mg (86%, 0.26 mmol) obtained as diastereomeric mixture (*dr* ~6:1, inseparable). Isolated as a yellow foam. R_f = 0.5 (hexane : ethyl acetate 1:1)

^{1}H NMR (400 MHz, $CDCl_3$): δ 7.40 – 7.38 (m, 1H), 6.43 – 6.38 (m, 1H), 6.24 – 6.19 (m, 1H), 4.64 (dd, *J* = 6.6, 3.3 Hz, 6/7 H), 4.24 (br s, 1/7 H), 4.21 (s, 6/7 H), 4.20 (br s, 6/7 H), 4.15 (br s, 1/7 H), 3.72 (br s, 6/7 H), 3.64 (br s, 1/7 H), 3.58 (br s, 6/7 H), 3.22 (br s, 1/7 H), 3.05 (dt, *J* = 11.6, 4.0 Hz, 1/7 H), 2.93 (br s, 1/7 H), 2.88 (d, *J* = 6.6 Hz, 6/7 H), 2.36 (dt, *J* = 12.3, 3.6 Hz, 1H), 2.36 (dt, *J* = 12.2, 3.3 Hz, 1H), 2.26 (dt, *J* = 12.2, 3.3 Hz, 1H), 2.06 (d, *J* = 13.1 Hz, 1H), 1.94 (d, *J* = 12.1 Hz, 1H) ppm.

^{13}C NMR (100 MHz, $CDCl_3$): δ 180.4, 179.0, 152.8, 150.6, 142.5, 142.5, 128.1, 127.8, 111.5, 111.2, 111.2, 111.1, 110.8, 110.7, 110.7, 110.6, 107.4, 84.8, 83.8, 80.7, 76.8, 53.0, 51.7, 50.7, 49.7, 49.6, 49.2, 48.6, 48.3, 48.2, 44.3, 44.2, 43.9, 42.3, 40.0, 29.8, 29.8 ppm.

HRMS (ESI) *m/z*: calcd for $C_{19}H_{14}N_4NaO_2$: 353.1009 $[M+Na]^+$; found: 353.1013.

FT-IR (neat): ν 3491, 2971, 2904, 2236, 1600, 1508, 1476, 1381, 914, 737 cm^{-1}.

2-((2*R*,3*aR*,5*R*,6*S*,6*aR*)-3*a*-(dicyanomethyl)-1-hydroxy-6-(naphthalen-2-yl)octahydro-2,5-methanopentalen-7-ylidene)malononitrile (3i, 3i')

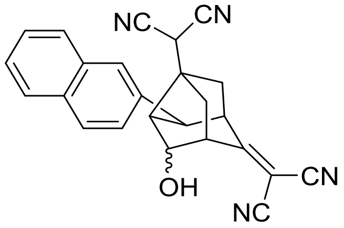

Was prepared following General procedure A. 102 mg (87%, 0.26 mmol) obtained as diastereomeric mixture (*dr* ~4:1, inseparable). Isolated as a pale green foam. R_f = 0.6 (hexane : ethyl acetate 1:1)

^{1}H NMR (400 MHz, $CDCl_3$): δ 7.90-7.81 (m, 3H), 7.59-7.48 (m, 3H), 7.33-7.27 (m, 1H), 4.68 (dd, *J* = 6.6, 3.2 Hz, 4/5 H), 4.35 (br s, 4/5 H), 4.28 (br s, 1/5 H), 4.00 (s, 1/5 H), 3.98 (br s, 4/5 H), 3.95 (s, 4/5 H), 3.91 (br s, 1/5 H), 3.66 (br s, 1/5 H), 3.59 (br s, 4/5 H), 3.34 (br s, 1/5 H), 3.17 (d, *J* = 6.1 Hz, 1H), 3.04 (dt, *J* = 11.8, 3.5 Hz, 1/5 H), 2.33 (dt, *J* = 12.0, 3.3 Hz, 4/5 H), 2.20 – 2.14 (m, 4/5 H), 2.04 (d, *J* = 8.2 Hz, 1/5 H), 1.98 (d, *J* = 12.0 Hz, 1H), 1.93 (d, *J* = 12.1Hz, 1/5 H), 1.86 (d, *J* = 11.9 Hz, 4/5 H) ppm.

^{13}C NMR (100 MHz, $CDCl_3$): δ 181.3, 179.9, 135.9, 133.8, 133.1, 132.3, 129.5, 129.4, 127.7, 127.7, 127.6, 127.0, 126.9, 126.7, 126.5, 124.9, 124.6, 124.4, 124.1, 111.4, 111.3, 110.9, 110.4, 84.6, 83.5, 81.4, 77.5, 53.6, 52.7, 51.6, 50.2, 49.8, 49.6, 49.5, 49.2, 48.7, 48.3, 47.8, 43.3, 43.1, 42.4, 40.1, 30.4, 30.2 ppm.

HRMS (ESI) *m/z*: calcd for $C_{25}H_{18}N_4NaO$: 413.1373 $[M+Na]^+$; found: 413.1364.

FT-IR (neat): v 3505, 3060, 2971, 2904, 2236, 1600, 1476, 1133, 1087, 910, 737 cm^{-1}.

3. General procedure B for the formation of compounds 4 and 4'

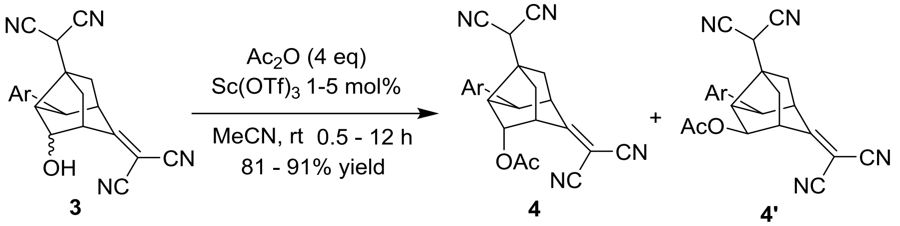

To the diastereomeric mixture of **3** in acetonitrile (0.1 M), acetic anhydride (4 equiv.) and scandium triflate (1-5 mol%) were added. The solution was left stirring at room temperature until completion (TLC) and quenched with saturated aqueous $NaHCO_3$. The aqueous mixture was extracted with dichloromethane and the combined organic layers were dried with Na_2SO_4 and evaporated *in vacuo*. Purification by column chromatography (Biotage Isolera One, hexane to hexane:ethyl acetate 3:1) followed to afford a diastereomeric mixture of **5** and **6**, which could be partially separated.

(1*S*,2*R*,3*aR*,5*R*,6*S*,6*aR*)-3*a*-(dicyanomethyl)-7-(dicyanomethylene)-6-phenyloctahydro-2,5-methanopentalen-1-yl acetate **(4a)**

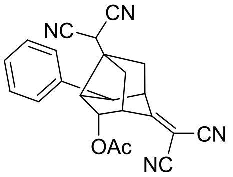

Was prepared following general procedure B from 83 mg diastereomeric mixture of alcohol (0.24 mmol, *dr* ~4:1) and 2 mg (2 mol%) $Sc(OTf)_3$ for 8 h to afford 74 mg (81%, 0.19 mmol) as a mixture of diastereoisomers (*dr* ~4:1).

4a was isolated as a colorless oil (R_f = 0.7, hexane:ethyl acetate 1:1)

^{1}H NMR (400 MHz, $CDCl_3$): δ 7.42 (t, *J* = 7.3 Hz, 2H), 7.34 – 7.30 (m, 1H), 7.16 (d, *J* = 8.0 Hz, 2H), 5.31 (dd, *J* = 6.6, 3.3 Hz, 1H), 3.97 (br s, 1H), 3.92 (s, 1H), 3.83 (br s, 1H), 3.81 (br s, 1H), 3.35 (d, *J* = 6.5 Hz, 1H), 2.42 (dt, *J* = 12.2, 3.5 Hz, 1H), 2.24 (dt, *J* = 12.1, 3.5 Hz, 1H), 2.14 (s, 3H), 2.10 (d, J = 12.2 Hz, 1H), 1.95 (d, J = 12.1 Hz, 1H) ppm.

^{13}C NMR (100 MHz, $CDCl_3$): δ 179.2, 169.3, 137.5, 129.5, 127.7, 126.0, 110.9, 110.7, 110.5, 110.2, 85.1, 77.7, 49.9, 49.7, 49.6, 46.4, 46.2, 43.4, 40.2, 30.2, 20.8 ppm.

HRMS (ESI) *m/z*: calcd for $C_{23}H_{18}N_4NaO_2$: 405.1322 $[M+Na]^+$; found: 405.1322.

FT-IR (neat): ν 2975, 2235, 1743, 1602, 1498, 1449, 1367, 1117, 1071, 737, 702 cm^{-1}.

$[\alpha]_D^{25}$ = -53.7 (*c* 1.0, $CHCl_3$)

HPLC (Daicel Chiralpak IG column, *n*-hexane/isopropanol = 9/1, flow 1.0 mL/min, detection at 233 nm) retention time = 12.2 min (minor) and 17.3 min (major), 98.7% *ee*.

(1*R*,2*R*,3*aR*,5*R*,6*S*,6*aR*)-3*a*-(dicyanomethyl)-7-(dicyanomethylene)-6-phenyloctahydro-2,5-methanopentalen-1-yl acetate **(4a')**

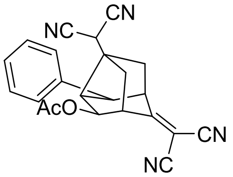

4a' was isolated as a yellow oil

^{1}H NMR (400 MHz, $CDCl_3$): δ 7.42 (t, J = 7.0 Hz, 2H), 7.35 – 7.31 (m, 1H), 7.15 (d, J = 7.9 Hz, 2H), 5.00 (br s, 1H), 3.89 (s, 1H), 3.88 (br s, 1H), 3.79 (br s, 1H), 3.42 (br s, 1H), 3.26 (br s, 1H), 2.97 (dt, *J* = 12.0, 3.7 Hz, 1H), 2.17 (s, 3H), 2.13 – 2.11 (m, 2H), 1.98 (d, *J* = 12.0 Hz, 1H) ppm.

^{13}C NMR (100 MHz, $CDCl_3$): δ 177.8, 170.0, 136.4, 129.5, 128.0, 126.1, 111.1, 110.6, 110.4, 84.6, 81.9, 53.7, 50.9, 50.1, 49.1, 48.6, 43.7, 43.0, 30.0, 20.9 ppm.

HRMS (ESI) *m/z***:** calcd for $C_{23}H_{18}N_4NaO_2$: 405.1322 $[M+Na]^+$; found: 405.1320.

FT-IR (neat): *v* 2916, 2235, 1744, 1602, 1449, 1365, 1231, 1051, 737 cm^{-1}.

HPLC (Daicel Chiralpak IB column, *n*-hexane/isopropanol = 9/1, flow 1.0 mL/min, detection at 233 nm) retention time = 25.6 min (minor) and 46.0 min (major), 98.6% *ee*.

$[\alpha]_D^{25}$ = -20.3 (*c* 0.2, $CHCl_3$)

(1*S*,2*R*,3*aR*,5*R*,6*S*,6*aR*)-3*a*-(dicyanomethyl)-7-(dicyanomethylene)-6-(4-methoxyphenyl)octahydro-2,5-methanopentalen-1-yl acetate (4b)

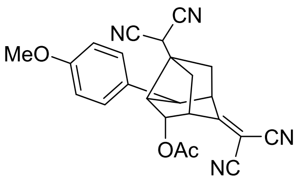

Was prepared following general procedure B from 81 mg diastereomeric mixture of alcohol (0.22 mmol, *dr* ~5:1) and 2.5 mg (2 mol%) $Sc(OTf)_3$ for 12 h to afford 75 mg (83%, 0.18 mmol) as a mixture of diastereoisomers (*dr* ~5:1).

4b was isolated as a yellow oil

^{1}H NMR (400 MHz, $CDCl_3$): δ 7.07 (d, *J* = 8.6 Hz, 2H), 6.93 (d, *J* = 8.6 Hz, 2H), 5.29 (dd, *J* = 6.5, 3.2 Hz, 1H), 3.92 (s, 1H), 3.90 (br s, 1H), 3.81 (s, 3H), 3.80 – 3.76 (m, 2H), 3.28 (d, *J* = 6.6 Hz, 1H), 2.41 (dt, *J* = 12.1 Hz , 3.5 Hz, 1H), 2.24 (dt, *J* = 11.9, 3.5 Hz, 1H), 2.13 (s, 3H), 2.09 (d, *J* = 12.2 Hz, 1H), 1.93 (d, *J* = 11.6 Hz, 1H) ppm.

^{13}C NMR (100 MHz, $CDCl_3$): δ 179.4, 169.3, 158.9, 129.2, 127.1, 114.8, 110.9, 110.7, 110.6, 110.2, 85.0, 77.7, 55.4, 49.9, 49.7, 49.3, 46.3, 46.2, 43.4, 40.1, 30.3, 20.8 ppm.

HRMS (ESI) *m/z*: calcd for $C_{24}H_{20}N_4NaO_3$: 435.1428 $[M+Na]^+$; found 435.1434.

FT-IR (neat): *v* 2964, 2907, 2232, 1740, 1606, 1515, 1360, 1228, 1179, 1073, 1034, 840 cm^{-1}.

$[\alpha]_D^{24}$ = -77.4 (*c* 1.0, $CHCl_3$)

HPLC (Daicel Chiralpak IG column, *n*-hexane/isopropanol = 9/1, flow 1.0 mL/min, detection at 233 nm) retention time = 15.4 min (minor) and 24.3 min (major), 98.3% *ee*.

(1*R*,2*R*,3*aR*,5*R*,6*S*,6*aR*)-3*a*-(dicyanomethyl)-7-(dicyanomethylene)-6-(4-methoxyphenyl)octahydro-2,5-methanopentalen-1-yl acetate (4b')

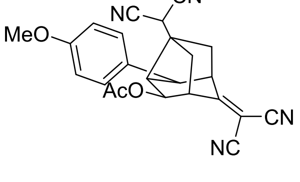

4b' was isolated as a yellow solid (m.p. 117 – 120 ^{o}C)

^{1}H NMR (400 MHz, $CDCl_3$): δ 7.05 (d, *J* = 8.7 Hz, 2H), 6.95 – 6.92 (m, 2H), 4.99 (s, 1H), 3.87 (s, 1H), 3.84 – 3.80 (m, 4H), 3.78 (br s, 1H), 3.36 (br s, 1H), 3.20 (br s, 1H), 2.96 (dt, *J* = 12.0 Hz, 3.7 Hz, 1H), 2.17 (s, 3H), 2.14 – 2.07 (m, 2H), 1.97 (d, *J* = 11.9 Hz, 1H) ppm.

^{13}C NMR (100 MHz, $CDCl_3$): δ 178.0, 170.0, 159.1, 128.1, 127.2, 114.8, 111.2, 110.6, 110.6, 110.4, 84.5, 81.9, 53.4, 53.1, 51.1, 50.0, 49.3, 48.5, 43.6, 43.0, 30.1, 20.9 ppm.

HRMS (ESI) *m/z*: calcd for $C_{24}H_{20}N_4O_3Na$: 435.1428 $[M+Na]^+$; found 435.1439.

FT-IR (neat): *v* 2968, 2907, 2232, 1744, 1606, 1519, 1466, 1362, 1228, 1179, 1052 cm^{-1}.

$[a]_D^{25}$ = -42.1 (*c* 0.9, $CHCl_3$)

<u>(1*S*,2*R*,3*aR*,5*R*,6*S*,6*aR*)-3a-(dicyanomethyl)-7-(dicyanomethylene)-6-(3-methoxyphenyl)octahydro-2,5-methanopentalen-1-yl acetate</u> (4c)

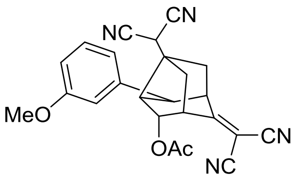

Was prepared following general procedure B from 88 mg diastereomeric mixture of alcohol (0.24 mmol, *dr* ~4:1) and 6 mg (5 mol%) $Sc(OTf)_3$ for 12 h to afford 82 mg (83%, 0.20 mmol) as a mixture of diastereoisomers (*dr* ~4:1).

4c was isolated as a colorless oil

^{1}H NMR (400 MHz, $CDCl_3$): δ 7.33 (t, *J* = 8.3 Hz, 1H), 6.83 (dd, *J* = 8.2, 2.5 Hz, 1H), 6.75 – 6.71 (m, 1H), 6.68 (br s, 1H), 5.31 (dd, *J* = 6.5 Hz, 3.3 Hz, 1H), 3.95 (s, 1H), 3.93 (br s, 1H), 3.82 (s, 3H), 3.79 (br s, 2H), 3.30 (d, *J* = 6.5 Hz, 1H), 2.41 (dt, *J* = 12.2 , 3.4 Hz, 1H), 2.22 (dt, *J* = 12.2, 3.4 Hz, 1H), 2.13 (s, 3H), 2.09 (d, *J* = 12.3 Hz, 1H), 1.94 (d, *J* = 11.7 Hz, 1H) ppm.

^{13}C NMR (100 MHz, $CDCl_3$): δ 179.3, 169.3, 160.3, 139.2, 130.5, 118.1, 113.0, 111.8, 111.0, 110.7, 110.5, 110.3, 85.0, 77.6, 55.3, 49.9, 49.7, 49.6, 46.4, 46.0, 43.4, 40.1, 30.2, 20.7 ppm.

HRMS (ESI) *m/z*: calcd for $C_{24}H_{20}N_4NaO_3$: 435.1428 $[M+Na]^+$; found 435.1425.

FT-IR (neat): *v* 3024, 2968, 2911, 2232, 1740, 1604, 1490, 1225, 1069, 1044, 758 cm^{-1}.

$[\alpha]_D^{25}$ = -70.9 (*c* 1.2, $CHCl_3$)

HPLC (Daicel Chiralpak IG column, *n*-hexane/isopropanol = 9/1, flow 1.0 mL/min, detection at 233 nm) retention time = 15.0 min (minor), 18.9 min (major), 99.0% *ee*.

VCD Analysis

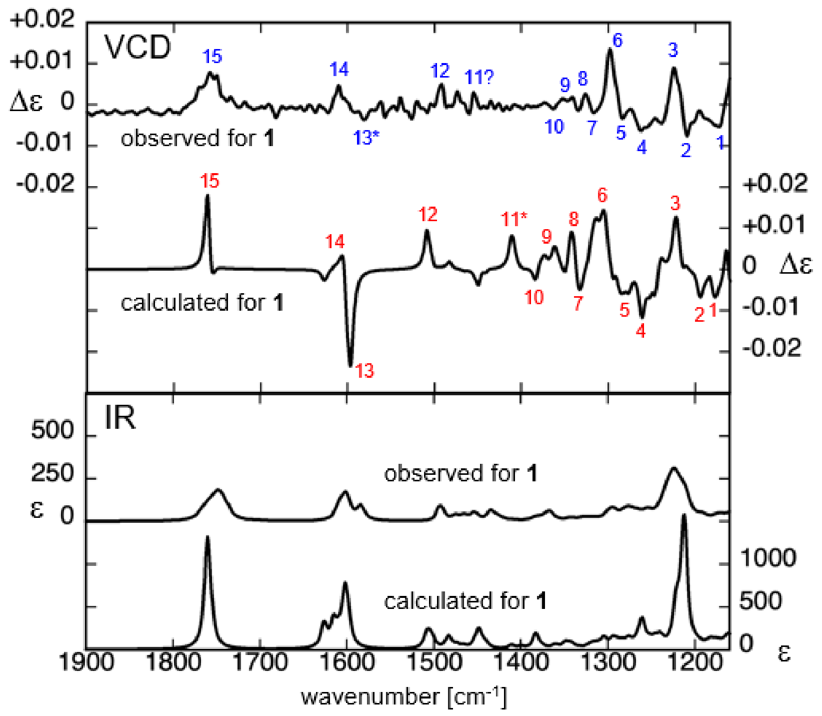

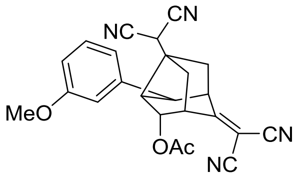

<u>**Measurement condition:**</u>

Instrument: JASCO FVS-6000 spectrometer
c = 0.5 M
$CDCl_3$ solution, measured at ambient temperature
l = 50 µm
IR: 16 scans, VCD: 3000 scans
All spectral data were corrected by a solvent spectrum obtained under the same experimental condition, and presented as De and e (both in M^{-1} cm^{-1}).

<u>**Calculation condition:**</u>

Preliminary MMFF conformational search of the arbitrarily chosen enantiomer shown above was carried out by CONFLEX 7 program. The obtained geometries within 3.0 kcal/mol from the most stable were optimized by DFT/B3LYP/6-311+G(d,p) using PCM for chloroform. Calculation was done on a Gaussian16 software, which led to 15 stable conformers in 1.75 kcal/mol energy window.

The IR and VCD spectra of the resultant stable conformers were calculated at the same level of theory, and then averaged based on their Boltzmann population at 298 K. The calculated frequencies, dipole strengths and rotational strengths were converted to IR and VCD spectra on GaussView 6 software using a peak half-width at half height of 4 cm^{-1} width. The calculated frequencies n were scaled with a factor of 0.99.

(1*R*,2*R*,3*aR*,5*R*,6*S*,6*aR*)-3*a*-(dicyanomethyl)-7-(dicyanomethylene)-6-(3-methoxyphenyl)octahydro-2,5-methanopentalen-1-yl acetate **(4c')**

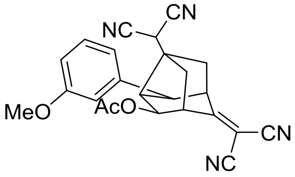

4c' was isolated as a yellow oil

^{1}H NMR (400 MHz, $CDCl_3$): δ 7.33 (t, *J* = 8.0 Hz, 1H), 6.85 (dd, *J* = 8.3 Hz, 2.4 Hz, 1H), 6.71 (d, *J* = 7.8 Hz, 1H), 6.68 – 6.63 (m, 1H), 4.98 (s, 1H), 3.91 (s, 1H), 3.85 (br s, 1H), 3.82 (s, 3H), 3.78 (m, 1H), 3.38 (br s, 1H), 3.22 (br s, 1H), 2.96 (dt, *J*= 12.0, 3.8 Hz, 1H), 2.17 (s, 3H), 2.14 – 2.08 (m, 2H), 1.97 (d, *J* = 11.7 Hz, 1H) ppm.

^{13}C NMR (100 MHz, $CDCl_3$): δ 177.8, 169.9, 160.4, 138.1, 130.6, 118.2, 113.2, 112.1, 111.2, 110.6, 110.6, 110.4, 84.6, 81.9, 55.4, 53.6, 50.9, 50.0, 49.3, 48.6, 43.7, 42.9, 30.0, 20.9 ppm.

HRMS (ESI) *m/z*: calcd for $C_{24}H_{20}N_4NaO_3$: 435.1428 $[M+Na]^+$; found 435.1428.

FT-IR (neat): *v* 2971, 2914, 2373, 2232, 1744, 1600, 1495, 1234, 1048, 757 cm^{-1}.

$[\alpha]_D^{25}$ = -19.7 (*c* 0.7, $CHCl_3$)

(1*S*,2*R*,3*aR*,5*R*,6*S*,6*aR*)-3*a*-(dicyanomethyl)-7-(dicyanomethylene)-6-(2-methoxyphenyl)octahydro-2,5-methanopentalen-1-yl acetate **(4d)**

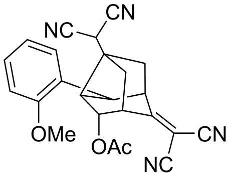

Was prepared following general procedure B from 73 mg diastereomeric mixture of alcohol (0.20 mmol, *dr* ~5:1) and 5 mg (5 mol%) $Sc(OTf)_3$ for 12 h to afford 75 mg (91%, 0.18 mmol) as a mixture of diastereoisomers (*dr* ~5:1).

4d was isolated as a colorless oil

^{1}H NMR (400 MHz, $CDCl_3$): δ 7.35 – 7.29 (m, 1H), 6.98 (t, *J* = 7.3 Hz, 1H), 6.93 (d, *J* = 8.3 Hz, 1H), 6.88 (d, *J* = 7.2 Hz ,1H), 5.34 (dd, *J* = 6.6, 3.3 Hz, 1H), 4.08 (s, 1H), 4.03 (br s, 1H), 3.91 (br s, 1H), 3.90 (s, 3H), 3.77 (br s, 1H), 3.23 (d, *J* = 6.4 Hz, 1H), 2.42 (dt, *J* = 12.1, 3.6 Hz, 1H), 2.20 – 2.15 (m, 1H), 2.14 (s, 3H), 2.10 (d, *J* = 12.0 Hz, 1H), 1.92 (d, *J* = 11.8 Hz, 1H) ppm.

^{13}**C NMR** (100 MHz, $CDCl_3$): δ 179.8, 169.4, 156.9, 129.2, 126.5, 125.8, 120.5, 111.2, 111.1, 110.8, 110.4, 84.7, 77.9, 55.1, 49.7, 49.0, 47.4, 46.3, 45.1, 43.5, 40.1, 30.2, 20.9 ppm.

HRMS (ESI) *m/z*: calcd for $C_{24}H_{20}N_4NaO_3$: 435.1428 $[M+Na]^+$; found 435.1429.

FT-IR (neat): *v* 2968, 2907, 2366, 2232, 1744, 1604, 1490, 1462, 1235, 1069, 762 cm^{-1}.

$[\mathbf{a}]_D^{24}$ = -7.6 (*c* 1.0, $CHCl_3$)

HPLC (Daicel Chiralpak IG column, *n*-hexane/isopropanol = 9/1, flow 1.0 mL/min, detection at 233 nm) retention time = 14.9 min (minor) and 25.3 min (major), 97.5% *ee*.

<u>(1*R*,2*R*,3*aR*,5*R*,6*S*,6*aR*)-3*a*-(dicyanomethyl)-7-(dicyanomethylene)-6-(2-methoxyphenyl)octahydro-2,5-methanopentalen-1-yl acetate</u> (4d')

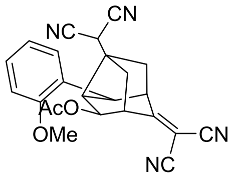

4d' was isolated as a colorless oil

^{1}**H NMR** (400 MHz, $CDCl_3$): δ 7.36 – 7.30 (m, 1H), 6.98 (t, *J* = 7.5Hz, 1H), 6.93 (d, *J* = 8.2 Hz, 1H), 6.82 (d, *J* = 7.4 Hz, 1H), 5.02 (s, 1H), 4.09 (s, 1H), 3.91 (br s, 1H), 3.89 (s, 3H), 3.76 (br s, 1H), 3.46 (br s, 1H), 3.15 (br s, 1H), 2.98 (dt, *J* = 11.9, 3.7 Hz, 1H), 2.15 (s, 3H), 2.14 – 2.09 (m, 1H), 2.04 – 1.99 (m, 1H), 1.95 (d, *J* = 12.2 Hz, 1H) ppm.

^{13}**C NMR** (100 MHz, $CDCl_3$): δ 178.5, 169.9, 156.7, 129.4, 126.6, 124.8, 120.5, 111.4, 111.2, 110.7, 110.7, 110.6, 84.1, 82.6, 55.2, 50.9, 50.0, 49.8, 48.5, 48.3, 43.8, 42.8, 29.9, 20.9 ppm.

HRMS (ESI) *m/z*: calcd for $C_{24}H_{20}N_4NaO_3$: 435.1428 $[M+Na]^+$; found 435.1426.

FT-IR (neat): *v* 3021, 2964, 2911, 2236, 1740, 1606, 1490, 1466, 1370, 1235, 1055, 762 cm^{-1}.

$[\boldsymbol{\alpha}]_D^{24}$ = + 26.6 (*c* 1.0, $CHCl_3$)

<u>(1*S*,2*R*,3*aR*,5*R*,6*S*,6*aR*)-6-(4-bromophenyl)-3*a*-(dicyanomethyl)-7-(dicyanomethylene)octahydro-2,5-methanopentalen-1-yl acetate</u> (4e)

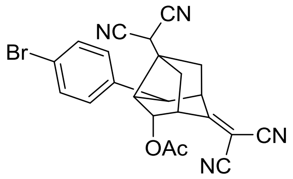

Was prepared following general procedure B from 64 mg diastereomeric mixture of alcohol (0.15 mmol, *dr* ~5:1) and 1.5 mg (2 mol%) $Sc(OTf)_3$ for 4 h to afford 59 mg (85%, 0.13 mmol) as a mixture of diastereoisomers (*dr* ~5:1).

4e was isolated as a yellow oil.

^{1}H NMR (400 MHz, $CDCl_3$) δ 7.54 (d, *J* = 8.5 Hz, 2H), 7.05 (d, *J* = 8.5 Hz, 2H), 5.29 (dd, *J* = 6.8, 3.2 Hz, 1H), 3.95 (br s, 1H), 3.87 (s, 1H), 3.81 (br s, 1H), 3.74 (br s, 1H), 3.29 (d, *J* = 6.4 Hz, 1H), 2.40 (dt, *J* = 12.2, 3.5 Hz, 1H), 2.23 (dt, *J* = 12.1, 3.5 Hz, 1H), 2.14 (s, 3H), 2.09 (d, *J* = 12.2 Hz, 1H) , 1.97 (d, *J* = 12.2 Hz, 1H) ppm.

^{13}C NMR (100 MHz, $CDCl_3$): δ 178.6, 169.4, 136.4, 132.5, 127.8, 121.7, 110.8, 110.6, 110.4, 110.2, 85.3, 77.6, 49.8, 49.3, 49.1, 46.5, 46.3, 43.3, 40.2, 30.2, 20.8 ppm.

HRMS (ESI) *m/z*: calcd for $C_{23}H_{17}BrN_4NaO_2$: 483.0427, 485.0407 $[M+Na]^+$; found: 483.0433, 485.0412.

FT-IR (neat): *v* 2975, 2900, 2232, 1742, 1603, 1491, 1366, 1227, 1073, 1010, 911, 732 cm^{-1}.

$[\alpha]_D^{25}$ = -95.2 (*c* 0.2, $CHCl_3$)

HPLC (Daicel Chiralpak IG column, *n*-hexane/isopropanol = 9/1, flow 1.0 mL/min, detection at 233 nm) retention time = 12.5 min (minor) and 19.8 min (major), 98.5% *ee*.

(1*R*,2*R*,3*aR*,5*R*,6*S*,6*aR*)-6-(4-bromophenyl)-3*a*-(dicyanomethyl)-7-(dicyanomethylene)octahydro-2,5-methanopentalen-1-yl acetate (4e')

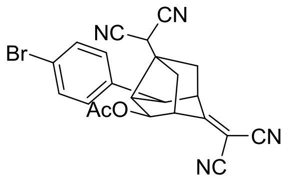

4e' was isolated as a yellow oil.

^{1}H NMR (400 MHz, $CDCl_3$): δ 7.54 (d, *J* = 8.6 Hz, 2H), 7.04 (d, *J* = 8.6 Hz, 2H), 4.98 (s, 1H), 3.86 (br s, 1H), 3.84 (s, 1H), 3.79 (br s, 1H), 3.34 (br s, 1H), 3.20 (br s, 1H), 2.96 (dt, *J* = 11.9, 3.6 Hz, 1H), 2.17 (s, 3H), 2.14 – 2.09 (m, 2H), 2.00 (d, J = 12.0 Hz, 1H) ppm.

^{13}C NMR (100 MHz, $CDCl_3$): δ 177.2, 170.0, 153.3, 132.6, 127.8, 122.0, 111.0, 110.5, 110.5, 110.3, 84.8, 81.8, 53.1, 51.1, 50.1, 48.6, 48.5, 43.6, 43.1, 30.0, 20.9 ppm.

HRMS (ESI) *m/z*: calcd for $C_{23}H_{17}BrN_4NaO_2$: 483.0427, 485.0407 $[M+Na]^+$; found: 483.0424, 485.0409.

FT-IR (neat): *v* 2975, 2904, 2236, 1744, 1604, 1490, 1366, 1228, 1052, 1010, 910, 730 cm^{-1}.

$[\alpha]_D^{24}$ = -31.6 (*c* 0.4, $CHCl_3$)

(1*S*,2*R*,3*aR*,5*R*,6*S*,6*aR*)-3*a*-(dicyanomethyl)-7-(dicyanomethylene)-6-(4-(trifluoromethyl)phenyl)octahydro-2,5-methanopentalen-1-yl acetate (4f)

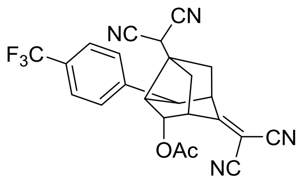

4f was isolated as a yellow oil

Was prepared following general procedure B from 100 mg diastereomeric mixture of alcohol (0.24 mmol, *dr* ~4:1) and 2.5 mg (2 mol%) $Sc(OTf)_3$ for 12 h to afford 90 mg (82%, 0.20 mmol) as a mixture of diastereoisomers (*dr* ~4:1).

^{1}H NMR (400 MHz, $CDCl_3$): δ 7.68 (d, *J* = 8.3Hz, 2H), 7.32 (d, *J* = 8.3 Hz, 2H), 5.31 (dd, *J* = 6.6, 3.2 Hz, 1H), 4.03 (br s, 1H), 3.89 (s, 1H), 3.86 – 3.80 (m, 2H), 3.37 (d, *J* = 6.3 Hz, 1H), 2.42 (dt, *J* = 12.4 , 3.5 Hz, 1H), 2.22 (dt, *J* = 12.3, 3.5 Hz, 1H), 2.14 (s, 3H), 2.10 (d, *J* = 12.3 Hz, 1H), 2.00 (d, *J* = 12.0 Hz, 1H) ppm.

^{13}C NMR (100 MHz, $CDCl_3$): δ 178.3, 169.4, 141.5 (d, *J* = 1 Hz), 130.1 (q, *J* = 33.0 Hz), 126.6, 126.3 (q, *J* = 4.0 Hz), 123.7 (q, *J* = 272.0 Hz), 110.7, 110.6, 110.4, 110.2, 85.4, 77.6, 49.9, 49.6, 48.9, 46.7, 46.4, 43.3, 40.3, 30.2, 20.8 ppm.

^{19}F NMR (376 MHz, $CDCl_3$): δ -62.8 (s, 3F) ppm.

HRMS (ESI) *m/z*: calcd for $C_{23}H_{17}F_3N_4NaO_2$: 473.1196 $[M+Na]^+$; found: 473.1195.

FT-IR (neat): *v* 2979, 2897, 2370, 2236, 1748, 1606, 1331, 1232, 1161, 1122, 1069 cm^{-1}.

$[\alpha]_D^{22}$ = -67.2 (*c* 1.0, $CHCl_3$)

HPLC (Daicel Chiralpak IG column, *n*-hexane/isopropanol = 9/1, flow 1.0 mL/min, detection at 233 nm) retention time = 8.2 min (minor) and 11.5 min (major), 98.4% *ee*.

(1*R*,2*R*,3*aR*,5*R*,6*S*,6*aR*)-3*a*-(dicyanomethyl)-7-(dicyanomethylene)-6-(4-(trifluoromethyl)phenyl)octahydro-2,5-methanopentalen-1-yl acetate **(4f')**

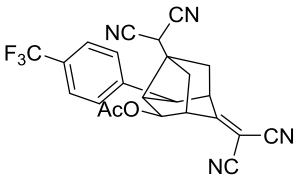

4f' was isolated as a yellow oil

^{1}H NMR (400 MHz, $CDCl_3$): δ 7.69 (d, *J* = 8.1 Hz, 2H), 7.32 (d, *J* = 8.1 Hz, 2H), 5.01 (s, 1H), 3.94 (br s, 1H), 3.85 (s, 1H), 3.81 (br s, 1H), 3.44 (br s, 1H), 3.27 (br s, 1H), 2.98 (dt, *J* = 12.1 Hz, 3.6 Hz, 1H), 2.18 (s, 3H), 2.14 – 2.08 (m, 2H), 2.03 (d, *J* = 11.8 Hz, 1H) ppm.

^{13}C NMR (100 MHz, $CDCl_3$): δ 176.9, 170.0, 140.4 (d, *J* = 1.0 Hz), 126.7, 126.5 (q, *J* = 4.0 Hz), 111.0, 110.5, 110.3, 85.0, 81.9, 53.3, 51.2, 50.2, 48.6, 48.5, 43.7, 43.1, 30.0, 20.9 ppm.

^{19}F NMR (376 MHz, $CDCl_3$): δ -62.8 (s, 3F) ppm.

HRMS (ESI) *m/z*: calcd for $C_{23}H_{17}F_3N_4NaO_2$: 473.1196 $[M+Na]^+$; found: 473.1193.

FT-IR (neat): *v* 2975, 2897, 2366, 2236, 1744, 1617, 1604, 1328, 1232, 1122, 1073 cm^{-1}.

$[\alpha]_D^{24}$ = -26.5 (*c* 1.0, $CHCl_3$)

(1*S*,2*R*,3*aR*,5*R*,6*S*,6*aR*)-3a-(dicyanomethyl)-7-(dicyanomethylene)-6-(4-nitrophenyl)octahydro-2,5-methanopentalen-1-yl acetate (4g)

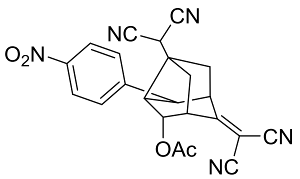

Was prepared following general procedure B from 78mg diastereomeric mixture of alcohol (0.20 mmol, *dr* ~1.5:1) and 2 mg (2 mol%) $Sc(OTf)_3$ for 12 h to afford 69 mg (81%, 0.16 mmol) as a mixture of diastereoisomers (*dr* ~1.5:1).

4g was isolated as a yellow oil

^{1}H NMR (400 MHz, $CDCl_3$): δ 8.28 – 8.20 (m, 2H), 7.39 (d, *J* = 8.4 Hz, 2H), 5.30 (dd, *J* = 6.7, 3.3 Hz, 1H), 4.09 (br s, 1H), 3.94 (s, 1H), 3.85 (br s, 2H), 3.39 (d, *J* = 6.6 Hz, 1H), 2.40 (dt, *J* = 12.3 Hz, 3.6 Hz, 1H), 2.30 (dt, *J* = 12.3, 3.4 Hz, 1H), 2.17 (s, 3H), 2.11 (d, *J* = 12.4 Hz, 1H), 2.03 (d, *J* = 12.4 Hz, 1H) ppm.

^{13}C NMR (100 MHz, $CDCl_3$): δ 177.9, 169.5, 147.1, 144.8, 127.3, 124.4, 110.7, 110.5, 110.3, 110.3, 85.7, 77.6, 50.0, 49.5, 48.3, 47.4, 46.4, 43.3, 40.4, 30.1, 20.8 ppm.

HRMS (ESI) *m/z*: calcd for $C_{23}H_{17}N_5NaO_4$: 450.1173 $[M+Na]^+$; found 450.1176.

FT-IR (neat): *v* 2968, 2907, 2232, 1738, 1606, 1519, 1466, 1366, 1228, 1186, 1069, 1034, 984 cm^{-1}.

$[\alpha]_D^{21}$ = -104.7 (*c* 1.0, $CHCl_3$)

HPLC (Daicel Chiralpak IG column, *n*-hexane/isopropanol = 8/1, flow 1.0 mL/min, detection at 249 nm) retention time = 25.2 min (minor) and 42.6 min (major), 98.4% *ee*.

(1*R*,2*R*,3*aR*,5*R*,6*S*,6*aR*)-3*a*-(dicyanomethyl)-7-(dicyanomethylene)-6-(4-nitrophenyl)octahydro-2,5-methanopentalen-1-yl acetate (4g')

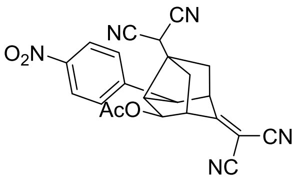

4g' was isolated as a yellow oil

^{1}H NMR (400 MHz, $CDCl_3$): δ 8.25 (d, *J* = 8.7 Hz, 2H), 7.41 (d, *J* = 8.7 Hz, 2H), 5.03 (s, 1H), 4.00 (br s, 1H), 3.94 (s, 1H), 3.82 (br s, 1H), 3.47 (br s, 1H), 3.29 (br s, 1H), 2.97 (dt, *J* = 12.4, 3.7 Hz, 1H), 2.18 (s, 3H), 2.17 - 2.04 (m, 3H) ppm.

^{13}C NMR (100 MHz, $CDCl_3$): δ 176.4, 170.1, 147.3, 143.6, 127.4, 124.5, 110.9, 110.5, 110.4, 110.2, 85.2, 81.8, 53.2, 51.7, 50.3, 48.5, 48.0, 43.6, 43.2, 29.9, 20.9 ppm.

HRMS (ESI) *m/z*: calcd for $C_{23}H_{17}N_5NaO_4$: 450.1173 $[M+Na]^+$; found 450.1171.

FT-IR (neat): *v* 2968, 2907, 2236, 1740, 1604, 1515, 1370, 1235, 1182, 1119, 1073, 1034, 984 cm^{-1}.

$[\alpha]_D^{25}$ = - 49.6 (*c* 1.0, $CHCl_3$)

(1*S*,2*R*,3*aR*,5*R*,6*S*,6*aR*)-3*a*-(dicyanomethyl)-7-(dicyanomethylene)-6-(furan-2-yl)octahydro-2,5-methanopentalen-1-yl acetate (4h)

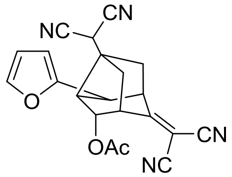

Was prepared following general procedure B from 85mg diastereomeric mixture of alcohol (0.26 mmol, *dr* ~6:1) and 5 mg (5 mol%) $Sc(OTf)_3$ for 12 h to afford 80 mg (82%, 0.22 mmol) as a mixture of diastereoisomers (*dr* ~6:1).

4h was isolated as a yellow oil. Partial decomposition was observed after 2 months storage at -20 ^{o}C.

^{1}H NMR (400 MHz, $CDCl_3$): δ 7.42 – 7.36 (m, 1H), 6.42 – 6.37 (m, 1H), 6.25 – 6.20 (m, 1H), 5.28 (dd, *J* = 6.5, 3.3 Hz, 1H), 4.20 (s, 1H), 3.83 (br s, 1H), 3.80 – 3.74 (m, 2H), 3.11 (d, *J* = 6.8 Hz, 1H), 2.44 (dt, *J* = 12.3, 3.6 Hz, 1H), 2.26 (dt, *J* = 12.3, 3.6 Hz, 1H), 2.17 – 2.08 (m, 4H), 1.99 (d, *J* = 11.9 Hz, 1H) ppm.

^{13}C NMR (100 MHz, $CDCl_3$): δ 178.3, 169.2, 151.6, 142.4, 111.2, 111.1, 110.6, 110.4, 110.4, 107.6, 85.3, 76.9, 50.1, 49.7, 46.4, 46.1, 45.2, 44.2, 40.2, 29.7, 20.8 ppm.

HRMS (ESI) *m/z*: calcd for $C_{21}H_{16}N_4NaO_3$: 395.1115 $[M+Na]^+$; found 395.1114.

FT-IR (neat): *v* 2979, 2907, 2232, 1797, 1744, 1604, 1370, 1232, 1065, 758 cm^{-1}.

$[\alpha]_D^{22}$ = -48.1 (*c* 1.0, $CHCl_3$)

HPLC (Daicel Chiralpak IG column, *n*-hexane/isopropanol = 9/1, flow 1.0 mL/min, detection at 233 nm) retention time 11.9 min (minor) and 18.2 min (major), 97.2% *ee*.

(1*R*,2*R*,3*aR*,5*R*,6*S*,6*aR*)-3*a*-(dicyanomethyl)-7-(dicyanomethylene)-6-(furan-2-yl)octahydro-2,5-methanopentalen-1-yl acetate (4h')

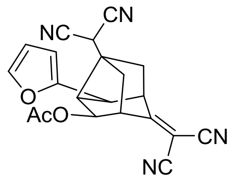

9b was isolated as a yellow oil. Partial decomposition was observed after 2 months storage at -20 °C.

^{1}H NMR (400 MHz, $CDCl_3$): δ 7.39 (d, J = 1.7 Hz, 1H), 6.41 (dd, J = 3.2 Hz, 1.9 Hz, 1H), 6.22 (d, J = 3.4 Hz, 1H), 4.94 (br s, 1H), 4.13 (s, 1H), 3.77 – 3.72 (m, 2H), 3.38 (br s, 1H), 3.10 – 3.07 (m, 1H), 2.98 (dt, J = 12.0, 3.7 Hz, 1H), 2.21 – 2.13 (m, 5H), 2.03 (d, J = 11.9 Hz, 1H) ppm.

^{13}C NMR (100 MHz, $CDCl_3$): δ 177.0, 169.9, 150.4, 142.6, 111.4, 111.2, 110.8, 110.4, 110.4, 107.9, 84.9, 81.1, 51.1, 50.1, 49.5, 48.8, 48.6, 44.4, 42.9, 29.6, 20.8 ppm.

HRMS (ESI) *m/z*: calcd for $C_{21}H_{16}N_4O_3Na$: 395.1115 $[M+Na]^+$; found 395.1115.

FT-IR: ν 2971, 2922, 2239, 1804, 1744, 1606, 1370, 1228, 1108, 1059, 1020, 762 cm^{-1}.

$[\alpha]_D^{23}$ = - 20.6 (*c* 0.4, $CHCl_3$)

(1*S*,2*R*,3*aR*,5*R*,6*S*,6*aR*)-3*a*-(dicyanomethyl)-7-(dicyanomethylene)-6-(naphthalen-2-yl)octahydro-2,5-methanopentalen-1-yl acetate (4i)

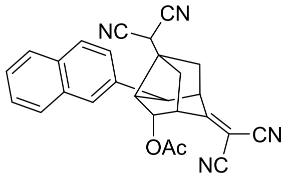

Was prepared following general procedure B from 102 mg diastereomeric mixture of alcohol (0.26 mmol, *dr* ~4:1) and 6.5 mg (5 mol%) $Sc(OTf)_3$ for 12 h to afford 95 mg (84%, 0.22 mmol) as a mixture of diastereoisomers (*dr* ~4:1).

4i was isolated as a pale orange amorphous solid

^{1}H NMR (400 MHz, $CDCl_3$): δ 7.90 (d, J = 8.6 Hz, 1H), 7.87 – 7.81 (m, 2H), 7.60 – 7.50 (m, 3H), 7.29 (dd, J = 8.6, 1.8 Hz, 1H), 5.34 (dd, J = 6.5, 3.3 Hz, 1H), 4.09 (br s, 1H), 3.97 (s, 1H), 3.93 (s, 1H), 3.81 (br s, 1H), 3.49 (d, J = 6.5 Hz, 1H), 2.42 (dt, J = 12.2, 3.5 Hz, 1H), 2.28 (dt, J = 12.2, 3.5 Hz, 1H), 2.14 (s, 3H), 2.09 (d, J = 11.9 Hz, 1H), 1.95 (d, J = 11.6 Hz, 1H) ppm.

^{13}C NMR (100 MHz, $CDCl_3$): δ 179.3, 169.4, 134.7, 133.1, 132.4, 129.6, 127.7, 127.7, 127.0, 126.7, 124.6, 123.9, 111.0, 110.8, 110.6, 110.2, 85.1, 77.8, 50.1, 49.8, 49.5, 46.4, 46.2, 43.5, 40.2, 30.3, 20.8 ppm.

HRMS (ESI) *m/z*: calcd for $C_{27}H_{20}N_4NaO_2$: 455.1478 $[M+Na]^+$; found 455.1485.

FT-IR (neat): ν 3021, 2971, 2911, 2236, 1744, 1600, 1366, 1225, 1065, 819, 751 cm^{-1}.

$[\alpha]_D^{25}$ = -103.7 (c 1.0, $CHCl_3$)

HPLC (Daicel Chiralpak IG column, *n*-hexane/isopropanol 9/1, flow 1.0 mL/min, detection at 226 nm) retention time = 20.3 min (minor) and 14.7 min (major), 98.2% *ee*.

(1*R*,2*R*,3*aR*,5*R*,6*S*,6*aR*)-3*a*-(dicyanomethyl)-7-(dicyanomethylene)-6-(naphthalen-2-yl)octahydro-2,5-methanopentalen-1-yl acetate (4i')

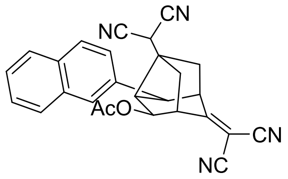

4i' was isolated as a pale yellow amorphous solid

^{1}H NMR (400 MHz, $CDCl_3$): δ 7.90 (d, *J* = 8.6 Hz, 1H), 7.86 – 7.82 (m, 2H), 7.58 – 7.51 (m, 3H), 7.26 (dd, *J* = 8.6, 1.8 Hz, 1H), 5.05 (s, 1H), 4.00 (br s, 1H), 3.90 (s, 1H), 3.82 (br s, 1H), 3.52 (br s, 1H), 3.40 (br s, 1H), 3.00 (dt, *J* = 12, 3.7 Hz, 1H), 2.20 (s, 3H), 2.18 – 2.08 (m, 2H), 2.00 (d, *J* = 12.2 Hz, 1H) ppm.

^{13}C NMR (100 MHz, $CDCl_3$): δ 177.8, 170.1, 133.6, 133.1, 132.5, 129.8, 127.8, 127.7, 127.2, 126.8, 124.8, 123.9, 111.2, 110.6. 110.6, 110.4, 84.6, 82.0, 53.8, 51.0, 50.1, 48.9, 48.7, 43.7, 43.0, 30.1, 20.9 ppm.

HRMS (ESI) *m/z* : calcd for $C_{27}H_{20}N_4O_2Na$: 455.1478 $[M+Na]^+$; found 455.1472.

FT-IR (neat): *v* 3024, 2968, 2915, 2232, 1740, 1606, 1366, 1228, 1055, 755 cm^{-1}.

$[\alpha]_D^{22}$ = - 53.2 (*c* 0.4, $CHCl_3$)

Formation of compound 5

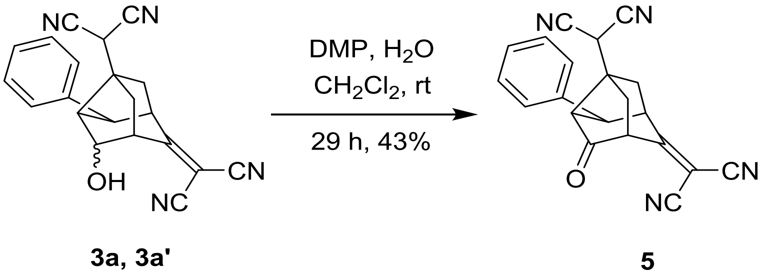

To a solution of noradamantyl alcohols **3a**, **3a'** (35 mg, 0.103 mmol, *dr* ~4:1) in dichloromethane (2 mL) was added Dess Martin periodinane (46 mg, 0.11 mmol, 1.1 equiv.). After stirring for 2 minutes, H_2O (2 μL, 0.11 mmol, 1.1 equiv.) was added. The mixture was left stirring at rt until starting material was completely consumed (TLC, 29 h) and then diluted with dichloromethane (2 mL). An aquatic solution of 10% w/v $Na_2S_2O_3$ (1 mL) and 10% $NaHCO_3$ (1 mL) were added. The organic layer was collected and washed with brine (1 mL). The solvent was removed *in vacuo* and the crude product was purified with

column chromatography (hexane to hexane : ethyl acetate 4:1) to afford ketone **5** (15mg, 0.044 mmol) as a colorless oil with 43% yield. (**Notice**: Partial decomposition of the product was observed in silica gel. Product was also found to decompose in alumina and upon prolonged storage at room temperature)

2-((2*R*,3*aR*,5*R*,6*S*,6*aR*)-3*a*-(dicyanomethyl)-1-oxo-6-phenyloctahydro-2,5-methanopentalen-7-ylidene)malononitrile (5)

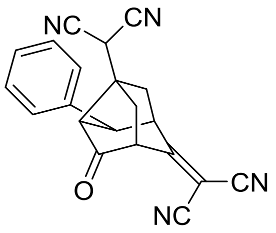

R$_f$ = 0.6 (5:3 hexane : ethyl acetate)

^{1}H NMR (400 MHz, $CDCl_3$): δ 7.46 (t, *J* = 7.6 Hz, 2H), 7.40 – 7.35 (m, 1H), 7.18 (d, *J* = 7.6 Hz, 2H), 4.11 – 4.09 (m, 1H), 4.08 – 4.05 (m, 1H), 4.02 (s, 1H), 3.53 (br s, 1H), 3.36 (br s, 1H), 2.47-2.39 (m, 2H), 2.36 (dt, *J* = 12.1, 3.3 Hz, 1H), 2.10 (d, *J* = 12.1 Hz, 1H) ppm.

^{13}C NMR (100 MHz, $CDCl_3$): δ 199.1, 174.6, 134.7, 129.7, 128.5, 126.3, 110.5, 110.2, 110.1, 109.7, 83.9, 56.1, 55.7, 53.6, 49.4, 47.6, 41.4, 41.2, 30.0 ppm.

IR (neat): v 2904, 2236, 1776, 1600, 1497, 1451, 1158, 762 cm^{-1}.

HRMS (ESI) *m/z*: calcd for $C_{21}H_{15}N_4O$: 339.1240 $[M+H]^+$; found: 339.1250.

$[a]_D^{25}$: -27.2 (c = 0.7, $CHCl_3$)

Formation of compound 6

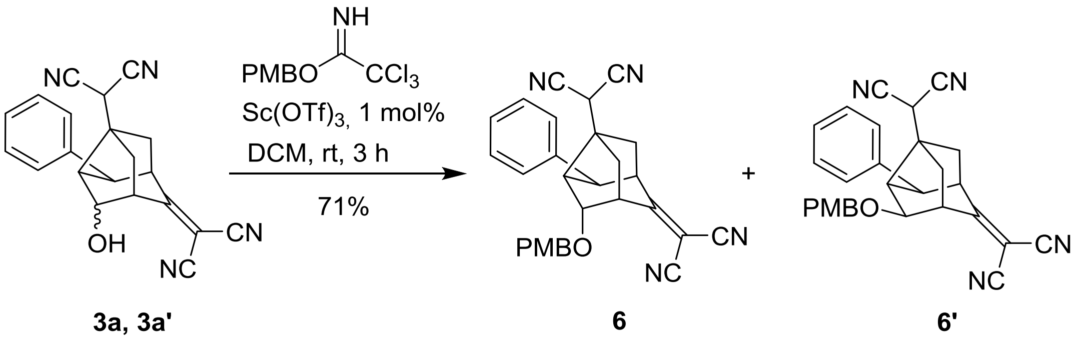

To a solution of a 3:1 diastereomeric mixture of alcohols **3a**, **3a’** (208 mg, 0.61 mmol) in dichloromethane (2 mL, 0.3 M), 4-methoxybenzyl 2,2,2-trichloroacetimidate (525 mg, 1.8 mmol) and $Sc(OTf)_3$ (3 mg, 1 mol%) are added. The solution is left stirring at room temperature for 3 h, after which a saturated solution of $NaHCO_3$ was added. The organic layer was removed and the aqueous layer was washed with dichloromethane. The combined organic layers were dried over Na_2SO_4, filtered and evaporated *in vacuo*. Column chromatography (hexane to hexane : ethyl acetate 4:1) followed to afford a

199 mg (71%) diastereomeric mixture (*dr* ~3:1) of compounds **6** and **6'**, from which **6** can be partially isolated as a yellow oil.

<u>2-((1*S*,2*R*,3*aR*,5*R*,6*S*,6a*R*)-3*a*-(dicyanomethyl)-1-((4-methoxybenzyl)oxy)-6-phenyloctahydro-2,5-methanopentalen-7-ylidene)malononitrile</u> (6)

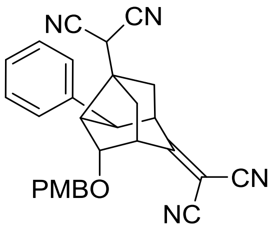

Isolated as a yellow oil. R_f = 0.7 (hexane : ethyl acetate 4:3)

^{1}H NMR (400 MHz, $CDCl_3$): δ 7.40 – 7.35 (m, 2H), 7.29 (d, *J* = 7.6 Hz, 1H), 7.24 (d, *J* = 8.3 Hz, 2H), 7.05 (d, *J* = 8.3 Hz, 2H), 6.86 (d, *J* = 8.7 Hz, 2H), 4.62 (d, *J* = 11.1 Hz, 1H), 4.49 (d, *J* = 11.1 Hz, 1H), 4.30 (dd, *J* = 6.4, 3.3 Hz, 1H), 4.10 (br s, 1H), 3.86 (br s, 2H), 3.79 (s, 3H), 3.75 (br s, 1H), 2.95 (d, *J* = 6.4 Hz, 1H), 2.29 (dt, *J* = 12.0, 3.7 Hz, 1H), 2.11 (dt, *J* = 12.0, 3.5 Hz, 1H), 2.02 (d, *J* = 12.3 Hz, 1H), 1.86 (d, *J* = 11.9 Hz, 1H) ppm.

^{13}C NMR (100 MHz, $CDCl_3$): δ 180.9, 159.8, 138.4, 129.8, 129.2, 128.5, 127.4, 126.1, 114.1, 111.2, 111.0, 110.9, 110.3, 84.5, 84.3, 72.6, 55.3, 50.1, 50.1, 49.4, 46.7, 46.6, 43.5, 40.4, 30.4 ppm.

HRMS (ESI) *m/z*: calcd for $C_{29}H_{24}N_4NaO_2$: 483.1791 $[M+Na]^+$; found: 483.1791.

FT-IR (neat): *v* 2960, 2915, 2236, 1606, 1596, 1515, 1469, 1250, 1129, 1091, 1038 cm^{-1}.

$[\alpha]_D^{24}$ = -32.1 (*c* 1.0, $CHCl_3$)

Formation of compound 7

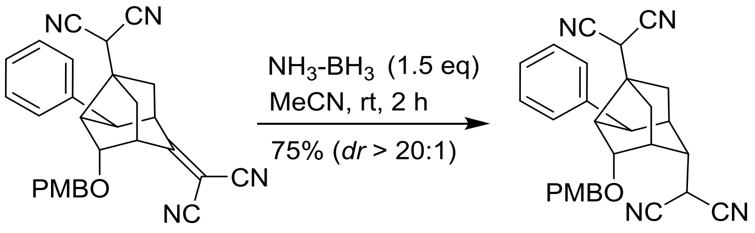

6 **7**

To a solution of the PMB ether **6** (60 mg, 0.13 mmol) in acetonitrile (1.3 mL, 0.1 M), was added ammonia-borane (6 mg, 0.2 mmol). The solution was left stirring for 2 hours and then filtered through a pad of silica. Solvent was removed *in vacuo* followed by purification with column chromatography (silica gel, hexane to hexane : ethyl acetate 3:1) to obtain 45 mg (75%) of compound **7** as a colorless oil.

<u>2,2'-((1*S*,2*R*,3*aR*,5*S*,6*S*,6*aR*,7*R*)-1-((4-methoxybenzyl)oxy)-6-phenylhexahydro-2,5-methanopentalene-3*a*,7(1*H*)-diyl)dimalononitrile</u> (7)

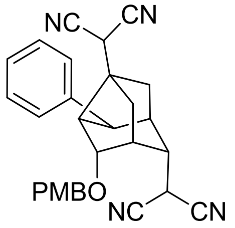

7 was isolated as a colorless oil. R_f = 0.7 (4:3 hexane : ethyl acetate)

^{1}H NMR (400 MHz, $CDCl_3$): δ 7.38 – 7.33 (m, 2H), 7.29 – 7.22 (m, 3H), 7.03 (d, *J* = 7.8 Hz, 2H), 6.86 (d, *J* = 8.6 Hz, 2H), 4.94 (d, *J* = 11.4 Hz, 1H), 4.57 (d, *J* = 11.1 Hz, 1H), 4.49 (d, *J* = 11.1 Hz, 1H), 4.16 – 4.13 (m, 1H), 3.83 (br s, 1H), 3.80 (s, 3H), 3.76 (s, 1H), 3.01 (br s, 1H), 2.81 (br s, 1H), 2.79 (br s, 1H), 2.52 (d, *J* = 11.6 Hz, 1H), 2.11 (dt, *J* = 12.1, 3.5 Hz, 1H), 1.99 (dt, *J* = 11.9, 3.4 Hz, 1H), 1.86 (d, *J* = 12.1 Hz, 1H), 1.79 (d, *J* = 11.5 Hz, 1H) ppm.

^{13}C NMR (100 MHz, $CDCl_3$): δ 159.8, 140.1, 129.7, 129.1, 128.5, 126.9, 125.8, 114.2, 112.7, 112.3, 111.5, 110.7, 83.7, 72.8, 55.3, 49.7, 46.5, 44.4, 44.3, 43.2, 42.9, 39.4, 38.5, 30.9, 25.0 ppm.

HRMS (ESI) *m/z*: calcd for $C_{29}H_{26}N_4NaO_2$: 485.1948 $[M+Na]^+$; found: 485.1941.

FT-IR (neat): *v* 2953, 2911, 1610, 1511, 1250, 1091, 1031 cm^{-1}.

$[\alpha]^{25}_D$ = + 17.9 (*c* 0.9, $CHCl_3$)

Formation of compound 8

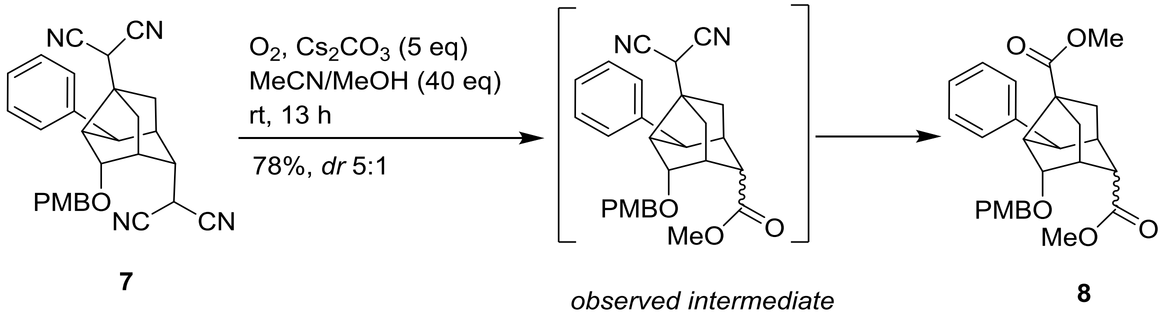

7 *observed intermediate* **8**

To a 4 mL vial with a rubber septum containing a solution of **7** (42 mg, 0.09 mmol) in acetonitrile (1 mL, 0.1 M) and methanol (150 μL, 3.64 mmol) was added Cs_2CO_3 (150 mg, 0.46 mmol). A flow of oxygen was provided through an oxygen balloon. The mixture was left stirring for 13 h, after which it was filtered through a pad of silica. The solvent was removed *in vacuo* to afford without further purification 32 mg (78%) diastereomeric mixture (*dr* ~5:1) of **8** (78%) as a yellow oil.

<u>dimethyl (1*S*,2*R*,3*aS*,5*R*,6*S*,6*aR*)-1-((4-methoxybenzyl)oxy)-6-phenylhexahydro-2,5-methanopentalene-3*a*,7(1*H*)-dicarboxylate</u> (8)

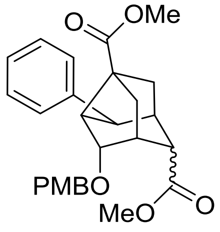

8 was isolated as a diastereomeric mixture (*dr* ~5:1, inseparable). Colorless oil. R_f = 0.7 (hexane : ethyl acetate 4:3).

^{1}H NMR (400 MHz, $CDCl_3$): δ 7.36 – 7.26 (m, 4H), 7.23 – 7.14 (m, 3H), 6.87 – 6.82 (m, 2H), 4.49 – 4.47 (m, 2/6 H), 4.41 (q, *J* = 12.0 Hz, 10/6 H), 4.18 (br s, 5/6 H), 4.08 (d, *J* = 3.3 Hz, 1/6 H), 4.05 (dd, *J* = 6.9, 3.0 Hz, 5/6 H), 3.91 (br s, 1/6 H), 3.79 (s, 3/6 H), 3.78 (s, 15/6 H), 3.75 (s, 15/6 H), 3.72 (s, 3/6 H), 3.69 (s, 3/6 H), 3.61 (s, 15/6 H), 3.26 (d, *J* = 6.5 Hz, 1/6 H), 3.22 (d, *J* = 6.8 Hz, 5/6 H), 3.17 (br s, 1/6 H), 3.03 (br s, 5/6 H), 2.85 – 2.79 (m, 1H), 2.72 – 2.69 (m, 1/6 H), 2.63 (t, *J* = 2.5 Hz, 5/6 H), 2.19 – 2.09 (m, 10/6 H), 1.97 (br s, 2/6 H), 1.82 (d, *J* = 11.8 Hz, 5/6 H), 1.67 (d, *J* = 11.0 Hz, 2/6 H), 1.52 (d, *J* = 11.6 Hz, 5/6 H) ppm.

^{13}C NMR (100 MHz, $CDCl_3$): δ 176.4, 176.2, 174.2, 173.7, 159.2, 158.9, 144.0, 144.3, 130.4, 129.8, 129.1, 128.6, 128.2, 128.1, 127.4, 127.3, 126.1, 125.6, 113.8, 113.6, 83.4, 81.8, 70.6, 70.4, 55.2, 55.2, 53.1, 53.0, 52.0, 51.9, 51.6, 51.5, 47.0, 46.7, 45.7, 45.6, 45.3, 44.6, 44.2, 40.6, 40.4, 40.1, 40.0, 39.0, 38.8 ppm.

HRMS (ESI) *m/z*: calcd for $C_{27}H_{30}NaO_6$: 473.1935 $[M+Na]^+$; found: 473.1938.

FT-IR (neat): *v* 2953, 2879, 1730, 1614, 1511, 1437, 1278, 1246, 1091, 1031 cm^{-1}.

Formation of compound 9

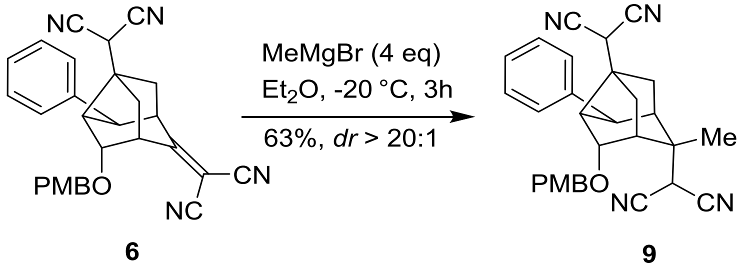

6 **9**

To a solution of **6** (50 mg, 0.11 mmol) in diethyl ether (0.1 M) under nitrogen atmosphere was added dropwise a 3 M solution of MeMgBr in diethyl ether (0.15 mL, 0.45 mmol) at -20 °C. The mixture was

slowly warmed to room temperature and was left stirring for 3 h until completion (TLC). An equal volume of 1 M aqueous HCl solution was added. The organic layer was removed and the aqueous one was further extracted with diethyl ether. The combined organic layers were dried with Na_2SO_4, filtered and evaporated *in vacuo*. Column chromatography (hexane to hexane : ethyl acetate 3:1) followed to obtain 33 mg (63%) of compound **9** as a colorless oil.

2,2'-((1*S*,2*R*,3*aR*,5*R*,6*S*,6*aR*,7*R*)-1-((4-methoxybenzyl)oxy)-7-methyl-6-phenylhexahydro-2,5-methanopentalene-3*a*,7(1*H*)-diyl)dimalononitrile (9)

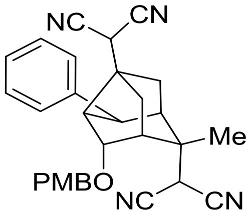

Was isolated as a colorless oil. R_f = 0.7 (hexane : ethyl acetate 4:3)

^{1}H NMR (400 MHz, $CDCl_3$): δ 7.37 – 7.32 (m, 2H), 7.28 – 7.22 (m, 3H), 7.02 (d, *J* = 7.7 Hz, 2H), 6.86 (d, *J* = 8.6 Hz, 2H), 5.58 (s, 1H), 4.63 (d, *J* = 11.3 Hz, 1H), 4.50 (d, *J* = 11.1 Hz, 1H), 4.13 (dd, *J* = 6.3, 3.1 Hz, 1H), 3.79 (s, 3H), 3.75 (s, 1H), 3.74 (br s, 1H), 2.75 (d, *J* = 6.6 Hz, 1H), 2.67 (br s, 1H), 2.49 (br s, 1H), 2.03 (d, *J* = 14.2 Hz, 1H), 1.95 – 1.89 (m, 2H), 1.79 – 1.73 (m, 1H), 1.46 (s, 3H) ppm.

^{13}C NMR (100 MHz, $CDCl_3$): δ 159.8, 140.3, 129.7, 129.0, 128.4, 126.9, 125.8, 114.2, 112.5, 111.9, 111.5, 110.7, 83.1, 73.2, 55.3, 49.3, 49.2, 44.7, 43.9, 43.4, 43.4, 38.7, 35.0, 31.1, 30.9, 21.9 ppm.

HRMS (ESI) *m/z*: calcd for $C_{30}H_{28}N_4NaO_2$: 499.2104 $[M+Na]^+$; found: 499.2108.

FT-IR (neat): ν 2936, 2366, 1614, 1508, 1250, 1119, 1034 cm^{-1}.

$[\alpha]^{25}_D$ = + 20.5 (*c* 1.0, $CHCl_3$)

Synthesis of bis-methyl ester 10

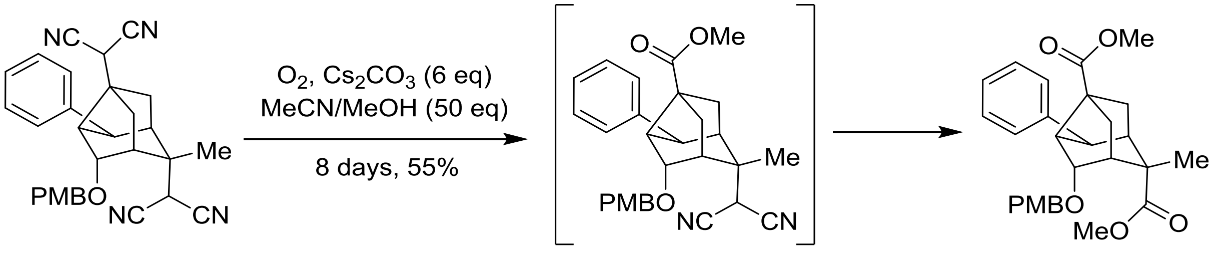

9 *observed intermediate* **10**

To a 4 mL vial with a rubber septum containing a solution of **9** (41 mg, 0.086 mmol) in acetonitrile (0.1 M) and methanol (170 μL, 4.3 mmol) was added Cs_2CO_3 (168 mg, 0.52 mmol). A flow of oxygen was

provided through an oxygen balloon. The mixture was left stirring at room temperature for 8 days, after which it was filtered through a pad of silica. The solvent was removed *in vacuo* and the crude product was purified with column chromatography (hexane to hexane : ethyl acetate 4:1) to afford 22 mg (55%) of bis-methyl ester **10** as a colorless oil.

dimethyl (1*S*,2*R*,3*aS*,5*R*,6*S*,6a*R*,7*S*)-1-((4-methoxybenzyl)oxy)-7-methyl-6-phenylhexahydro-2,5-methanopentalene-3*a*,7(1*H*)-dicarboxylate (10)

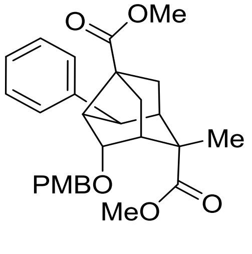

10 was isolated as a colorless oil. R_f = 0.7 (hexane : ethyl acetate 4:3)

^{1}H NMR (400 MHz, $CDCl_3$): δ 7.33 – 7.26 (m, 4H), 7.21 – 7.14 (m, 3H), 6.83 (d, *J* = 8.3 Hz, 2H), 4.38 (q, *J* = 11.2 Hz, 2H), 4.14 (br s, 1H), 4.02 (dd, *J* = 6.8, 3.1 Hz, 1H), 3.78 (s, 3H), 3.74 (s, 3H), 3.53 (s, 3H), 3.17 (d, *J* = 6.7 Hz, 1H), 2.73 (br s, 1H), 2.53 (br s, 1H), 1.99 – 1.88 (m, 3H), 1.69 – 1.64 (m, 1H), 1.26 (s, 3H) ppm.

^{13}C NMR (100 MHz, $CDCl_3$): δ 177.6, 176.5, 158.9, 144.3, 130.4, 128.8, 128.1, 127.3, 125.6, 113.6, 82.8, 70.5, 55.2, 53.0, 52.0, 51.6, 49.9, 46.5, 45.9, 44.2, 44.2, 36.3, 36.2, 23.5 ppm.

HRMS (ESI) *m/z*: calcd for $C_{28}H_{32}NaO_6$: 487.2091 $[M+Na]^+$; found: 487.2094.

FT-IR (neat): *v* 2953, 2922, 1727, 1614, 1511, 1264, 1116, 1034 cm^{-1}.

$[\alpha]^{27}_{D}$ = +31.4 (*c* 0.2, $CHCl_3$)

Formation of compound 11

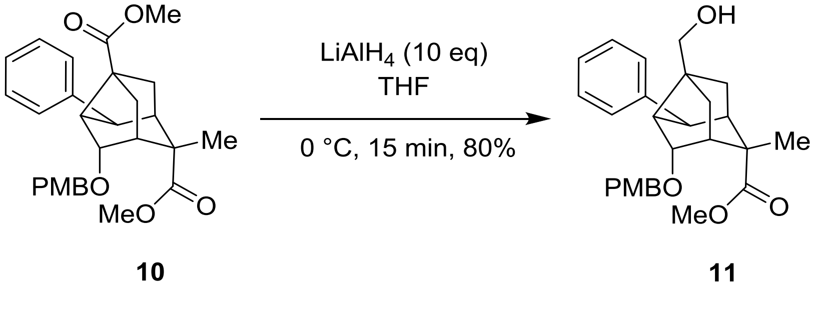

To a solution of the ester **10** (4.0 mg, 8.6 μmol) in THF (0.17 mL, 0.05 M), was added $LiAlH_4$ (3.3 mg, 0.086 mmol) at 0 ℃. The solution is left stirring at room temperature for 15 min, after which a saturated

solution of Rochelle's salt was added. The organic layer was removed and the aqueous layer was washed with ethyl acetate. The combined organic layers were dried with Na_2SO_4, filtered and evaporated *in vacuo*. Column chromatography (hexane : ethyl acetate = 2:1) followed to obtain 2.8 mg (80%) of compound **11** as a pale yellow oil.

methyl (1*S*,2*R*,3*aS*,5*R*,6*S*,6*aR*,7*S*)-3*a*-(hydroxymethyl)-1-((4-methoxybenzyl)oxy)-7-methyl-6-phenyloctahydro-2,5-methanopentalene-7-carboxylate (11)

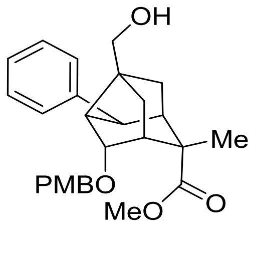

Was isolated as a colorless oil. R_f = 0.2 (hexane : ethyl acetate 2:1)

^{1}H NMR (400 MHz, $CDCl_3$): δ 7.34 – 7.25 (m, 4H), 7.21 – 7.14 (m, 3H), 6.85 – 6.80 (m, 2H), 4.38 (dd, *J* = 11.2, 24.0 Hz, 2H), 4.11 (br s, 1H), 3.91 (dd, *J* = 3.2, 6.4 Hz, 1H), 3.77 (s, 3H), 3.70 – 3.69 (m, 2H), 3.52 (s, 3H), 2.71 – 2.68 (m, 2 H), 2.62 (br s, 1H), 1.65 (br s, 2H), 1.49 (br d, *J* = 12.4 Hz, 1H), 1.42 (br d, *J* = 12.4 Hz, 1H), 1.28 (s, 3H) ppm.

^{13}C NMR (100 MHz, $CDCl_3$): δ 178.0, 158.9, 144.8, 130.7, 128.7, 128.1, 126.9, 125.4, 113.5, 83.4, 70.3, 68.1, 55.2, 51.5, 50.4, 49.1, 47.1, 45.7, 44.2, 42.4, 35.8, 34.2, 23.7 ppm.

HRMS (ESI) *m/z*: calcd for $C_{27}H_{32}NaO_5$: 459.2142 $[M+Na]^+$; found: 459.2190.

FT-IR (neat): ν 3463, 2948, 2877, 1727, 1613, 1514, 1498, 1481, 1464, 1450, 1373, 1360, 1301, 1248, 1198, 1174, 1116, 1081, 1035, 821, 744, 704 cm^{-1}.

[α]$^{22}_{D}$ = + 5.7 (*c* 0.14, $CHCl_3$)

Formation of compound 13

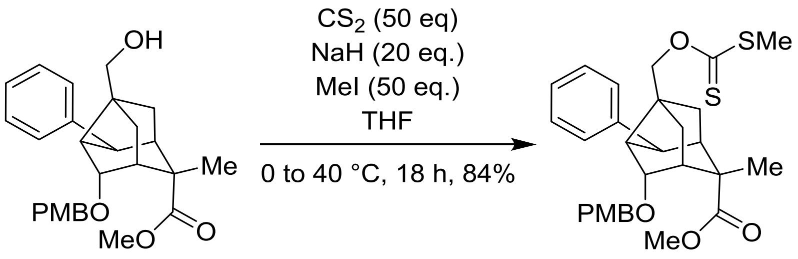

11 **12**

To a solution of the alcohol **11** (10 mg, 23 μmol) in THF (0.76 mL, 0.03 M), was added CS_2 (69 μL, 1.2 mmol) and NaH (60% in oil, 18 mg, 0.46 mmol) at 0 °C. After stirring for 0.5 h at this temperature, MeI (36 μL, 0.57 mmol) was added to this mixture. The solution is left stirring at 40 °C for 18 hours, after which this reaction mixture was cooled to 0 °C then a saturated solution of NH_4Cl was added. The organic layer was removed and the aqueous layer was washed with ethyl acetate 2 times. The combined organic layers were dried with Na_2SO_4, filtered and evaporated *in vacuo*. Column chromatography (hexane to hexane : ethyl acetate = 6:1) followed to obtain 10.2 mg (84%) of compound **12** as a pale yellow oil.

methyl (1*S*,2*R*,3*aS*,5*R*,6*S*,6*aR*,7*S*)-1-((4-methoxybenzyl)oxy)-7-methyl-3*a*-((((methylthio)carbonothioyl)oxy)methyl)-6-phenyloctahydro-2,5-methanopentalene-7-carboxylate (12)

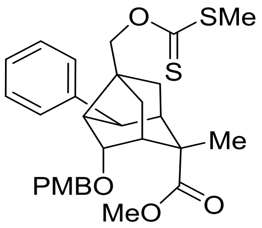

Was isolated as a pale yellow oil. R_f = 0.4 (hexane : ethyl acetate 6:1)

^{1}H NMR (400 MHz, $CDCl_3$): δ 7.31 (d, *J* = 4.3 Hz, 4H), 7.21 – 7.16 (m, 3H), 6.84 – 6.82 (m, 2H), 4.67 (d, *J* = 11.0 Hz, 1H), 4.62 (d, *J* = 11.0 Hz, 1H), 4.43 (d, *J* = 11.3 Hz, 1H), 4.36 (d, *J* = 11.3 Hz, 1H), 4.14 (br s, 1H), 3.92 (dd, *J* = 6.7, 3.2 Hz, 1H), 3.78 (s, 3H), 3.54 (s, 3H), 2.76 – 2.72 (m, 2H), 2.65 (br s, 1H), 2.50 (s, 3H), 1.74 (br d, *J* = 12.2 Hz, 1H), 1.68 – 1.58 (m, 2H), 1.50 (d, *J* = 12.2 Hz, 1H), 1.29 (s, 3H) ppm.

^{13}C NMR (100 MHz, $CDCl_3$): δ 177.8, 158.9, 144.3, 130.5, 128.8, 128.2, 126.9, 125.5, 113.6, 82.9, 78.3, 70.4, 55.2, 51.6, 49.1, 48.1, 47.0, 45.8, 43.9, 43.1, 36.1, 34.9, 23.6, 18.8 ppm.

HRMS (ESI) *m/z*: calcd for $C_{29}H_{34}NaO_5S_2$: 549.1740 $[M+Na]^+$; found: 549.1755.

FT-IR (neat): ν 2950, 2879, 1727, 1612, 1513, 1450, 1302, 1247, 1233, 1219, 1186, 1179, 1119, 1096, 1068, 1048, 1036, 1000, 738, 704 cm^{-1}.

[α]$^{27}_{D}$ = +10.6 (*c* 0.06, $CHCl_3$)

Formation of compound 13

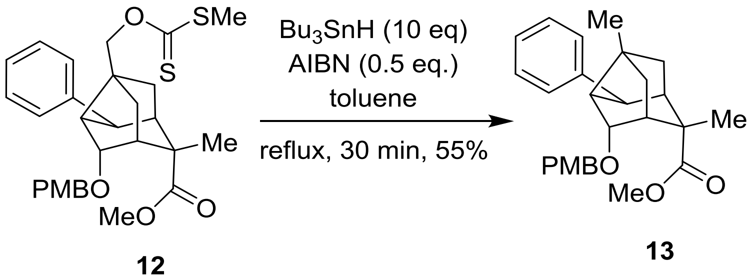

12 **13**

To a solution of the xanthate **12** (8.0 mg, 15 μmol) in toluene (0.30 mL, 0.05 M), was added Bu_3SnH (41 μL, 0.15 mmol) and AIBN (1.2 mg, 7.5 μmol) at room temperature. The solution is heated and left stirring at reflux for 30 minutes, after which this reaction mixture was cooled to room temperature and the solvent was removed in *vacuo*. Column chromatography (hexane to hexane : ethyl acetate = 9:1) was followed to obtain 3.5 mg (55%) of compound **13** as a colorless oil.

<u>methyl (1*S*,2*R*,3*aR*,5*R*,6*S*,6*aR*,7*S*)-1-((4-methoxybenzyl)oxy)-3*a*,7-dimethyl-6-phenyloctahydro-2,5-methanopentalene-7-carboxylate</u> (13)

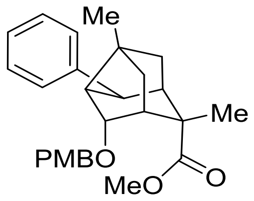

Was isolated as a colorless oil. R_f = 0.5 (hexane : ethyl acetate 6:1)

^{1}H NMR (400 MHz, $CDCl_3$): δ 7.36 – 7.27 (m, 4H), 7.23 – 7.12 (m, 3H), 6.87 – 6.79 (m, 2H), 4.40 (d, *J* = 11.3 Hz, 1H), 4.33 (d, *J* = 11.3 Hz, 1H), 4.08 (br s, 1H), 3.93 (dd, *J* = 6.6, 3.2 Hz, 1H), 3.78 (s, 3H), 3.53 (s, 3H), 2.64 (br s, 1H), 2.51 – 2.42 (m, 2H), 1.68 (br d, *J* = 12.0 Hz, 1H), 1.48 (dt, *J* = 3.2, 12.0 Hz, 1H), 1.40 (dt, 1H, J = 3.2, 12.0 Hz), 1.36 (br d, *J* = 12.0 Hz, 1H), 1.26 (s, 3H), 1.24 (s, 3H) ppm.

^{13}C NMR (100 MHz, $CDCl_3$): δ 178.2, 158.8, 145.5, 130.9, 128.7, 127.9, 127.3, 125.3, 113.5, 83.3, 70.2, 55.2, 51.4, 50.3, 46.7, 46.5, 46.0, 44.6, 44.0, 40.2, 39.6, 24.9, 23.7 ppm.

HRMS (ESI) *m/z*: calcd for $C_{27}H_{32}NaO_4$: 443.2193 $[M+Na]^+$; found: 443.2200.

FT-IR (neat): ν 2947, 2874, 1728, 1613, 1514, 1452, 1248, 1203, 1173, 1127, 1111, 1035, 821, 703 cm^{-1}.

[α]$^{22}_{D}$ = –13.3 (*c* 0.02, $CHCl_3$)

Formation of compound 14

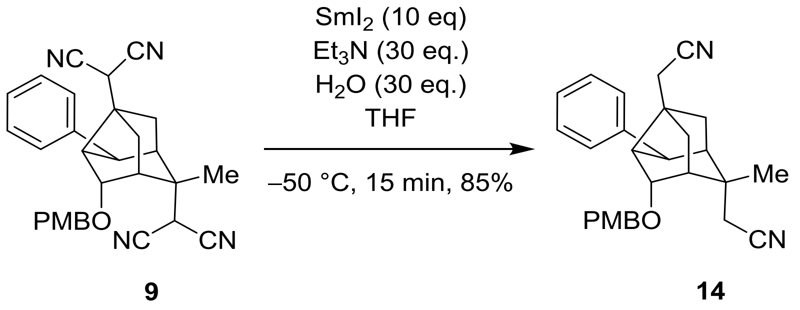

All reagents were used after freeze-pump-thaw cycling, and this reaction was conducted in the Schlenk flask under argon.

To a THF solution of SmI_2 (0.1 M, 2.5 mL, 0.25 mmol) was added **9** (12 mg, 0.025 mmol) in THF (0.5 mL) under argon. Et_3N (105 μL, 0.76 mmol) and H_2O (105 μL, 0.76 mmol) was then added in dropwisem manner into the reaction mixture at -50 ^{o}C. The mixture was stirred for 15 min until completion (TLC). After the reaction mixture was opened to air, an equal volume of water was added. The organic layer was removed and the aqueous one was further extracted with EtOAc. The combined organic layers were dried with Na_2SO_4, filtered and evaporated *in vacuo*. Column chromatography (hexane : ethyl acetate 2:1) followed to obtain 9.2 mg (85%) of compound **14** as a pale yellow oil.

2,2'-((1*S*,2*R*,3*aR*,5*R*,6*S*,6*aR*,7*S*)-1-((4-methoxybenzyl)oxy)-7-methyl-6-phenylhexahydro-2,5-methanopentalene-3*a*,7(1H)-diyl)diacetonitrile (14)

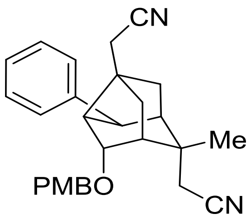

Was isolated as a pale yellow oil. R_f = 0.6 (hexane : ethyl acetate 2:1)

^{1}H NMR (400 MHz, $CDCl_3$): δ 7.36 – 7.29 (m, 2H), 7.27 – 7.20 (m, 3H), 7.10 – 7.08 (m, 2H), 6.88 – 6.84 (m, 2H), 5.64 (s, 1H), 4.57 (d, *J* = 11.2 Hz, 1H), 4.49 (d, *J* = 11.2 Hz, 1H), 4.11 – 4.10 (m, 1H), 3.80 (s, 3H), 3.79 (s, 1H), 3.70 (br s, 1H), 2.73 (d, *J* = 6.8 Hz, 1H), 2.57 (d, *J* = 2.8 Hz, 2H), 2.52 – 2.51 (m, 1H), 2.41 (br s, 1H), 1.85 (d, *J* = 12.4 Hz, 1H), 1.79 (dt, *J* = 3.2, 15.6 Hz, 1H), 1.71 (d, J = 12.4 Hz, 1H), 1.63 (dt, *J* = 3.2, 15.6 Hz, 1H), 1.42 (s, 3H) ppm.

^{13}C NMR (100 MHz, $CDCl_3$): δ 159.6, 141.5, 129.4, 128.9, 128.8, 126.6, 126.3, 117.6, 114.1, 112.8, 112.2, 83.5, 72.7, 55.3, 50.1, 45.0, 44.4, 44.3, 43.5, 39.7, 37.2, 31.1, 26.3, 22.0 ppm.

HRMS (ESI) *m/z*: calcd for $C_{28}H_{30}N_2NaO_2$: 449.2199 $[M+Na]^+$; found: 449.2200.

FT-IR (neat): v 2925, 2854, 2358, 1732, 1612, 1513, 1456, 1247, 1174, 1083, 1031, 821, 732, 701, 642, 628, 574 cm^{-1}.

$[\alpha]^{28}_{D}$ = –13.4 (*c* 0.07, $CHCl_3$)

General procedure C for the formation of compounds 16

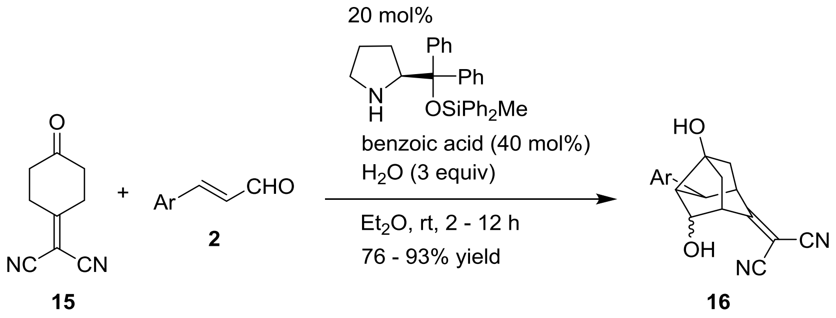

To a solution of ketone **15** (40.0 mg, 0.25 mmol) and aldehyde **2** (40.0 mg, 0.30 mmol) in Et_2O (600 μL), H_2O (13.5 μL, 0.75 mmol), diphenylprolinol silyl ether (22.4 mg, 0.050 mmol) and benzoic acid (12.2 mg, 0.10 mmol) were added at room temperature. After stirring the reaction mixture at this temperature, the solvent was removed under reduced pressure. The residue was directly purified by column chromatography on silica gel (*n*-Hexane: EtOAc = 3:1~1/1) to give compounds **16** as an inseparable mixture of two diastereoisomers.

2-((2*R*,3*aS*,5*R*,6*S*,6*aS*)-1,3a-dihydroxy-6-phenyloctahydro-2,5-methanopentalen-7-ylidene)malononitrile (16a, 16a')

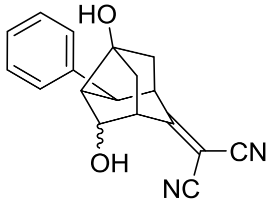

Was prepared following General procedure C. 71.3 mg (93%) obtained as diastereomeric mixture (*dr* ~4:1, inseparable). Isolated as a yellow solid. R_f = 0.2 (hexane : ethyl acetate 1:1)

For major isomer:

^{1}H NMR (CD_3CN, 400 MHz) δ 7.42-7.34 (m, 5H), 4.65 (quint, *J* = 3.2 Hz, 1H), 4.01 (br s, 1H), 3.76 (d, *J* = 3.6 Hz, 1H), 3.67 (br s, 1H), 3.39 (q, *J* = 1.6 Hz, 1H), 3.36 (br s, 1H), 2.69 (td, *J* = 2.0, 6.8 Hz, 1H), 2.22-2.18 (m,1H), 1.69 (d, *J* = 11.2 Hz, 1H) ppm.

^{13}C NMR (CD_3CN, 100 MHz) δ 186.6, 142.7, 129.3, 129.3, 129.2, 129.2, 129.0, 127.5, 113.0, 112.6, 82.1, 77.5, 53.1, 51.5, 50.6, 50.5, 45.8, 45.6 ppm.

HRMS (ESI): $[M+Na]^+$ calcd for $C_{18}H_{16}N_2O_2Na$: 315.1104 $[M+Na]^+$; found: 315.1109.

IR(neat) ν 3431, 3060, 2968, 2236, 1592, 1233, 1497, 1451, 1415, 1308, 1265, 1231, 1129, 1086, 980, 930, 737, 704, 652, 610 cm^{-1}.

<u>2-((2*R*,3*aS*,5*R*,6*S*,6*aS*)-1,3*a*-dihydroxy-6-(4-methoxyphenyl)octahydro-2,5-methanopentalen-7-ylidene)malononitrile</u> (16b, 16b')

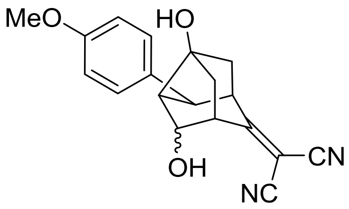

Was prepared following General procedure C. 71.0 mg (81%) obtained as diastereomeric mixture (*dr* ~1.5:1, inseparable). Isolated as a yellow solid. R_f = 0.1 (hexane : ethyl acetate 1:1)

For the major isomer:

^{1}H NMR (CD_3CN, 400 MHz) δ 7.30 (d, *J* = 8.4 Hz, 2H), 6.94 (d, *J* = 8.8 Hz, 2H), 4.63 (quint, *J* = 3.6 Hz, 1H), 3.95 (br s, 1H), 3.80 (s, 3H), 3.73 (d, *J* = 3.6 Hz, 1H), 3.65 (br s, 1H), 3.3.8 (br s, 1H), 3.30 (br s, 1H), 2.84 (td, *J* = 3.6, 10.8 Hz, 1H), 2.63 (td, *J* = 1.6, 6.4 Hz, 1H), 1.68 (d, *J* = 11.2 Hz, 1H) ppm.

^{13}C NMR (CD_3CN, 100 MHz) δ 187.0, 159.3, 134.6, 133.3, 130.3, 130.1, 114.6, 113.0, 112.6, 82.6, 82.1, 77.5, 55.8, 53.3, 51.5, 50.7, 49.9, 45.9, 45.6 ppm.

HRMS (ESI): $[M+Na]^+$ calcd for $C_{19}H_{18}N_2O_3Na^+$: 345.1210, found: 345.1210.

IR (neat): I3439, 2965, 2235, 1591, 1513, 1464, 1307, 1252, 1181, 1116, 1082, 1034, 835, 735, 569, 532 cm^{-1}.

<u>2-((1*S*,2*R*,3*aS*,5*R*,6*aS*)-1-(4-fluorophenyl)-3*a*,6-dihydroxyoctahydro-2,5-methanopentalen-7-ylidene)malononitrile</u> (16c, 16c')

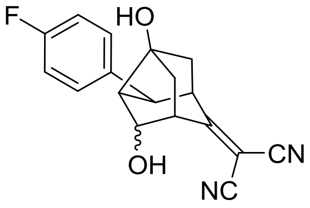

Was prepared following General procedure C. 63.3 mg (82%) obtained as diastereomeric mixture (*dr* ~3.3:1, inseparable). Isolated as a yellow solid. R_f = 0.2 (hexane : ethyl acetate 1:1)

For the major isomer:

^{1}H NMR (CD_3CN, 400 MHz) δ 7.40 (dd, *J* = 5.2, 8.0 Hz, 2H), 7.13 (t, *J* = 9.2 Hz, 2H), 4.63 (quint, *J* = 3.6 Hz, 1H), 4.00 (br s, 1H), 3.76 (d, *J* = 3.6 Hz, 1H), 3.68 (br s, 1H), 3.40-3.38 (m, 1H), 3.33 (br s, 1H), 2.65 (td, *J* = 2.0, 6.4 Hz, 1H), 2.15-2.13 (m, 1H), 1.70 (d, *J* = 11.2 Hz, 1H) ppm.

^{13}C NMR (CD_3CN, 100 MHz) δ 185.6, 161.5 (J_{C-F} = 242 Hz), 137.8 (J_{C-F} = 3.1 Hz), 130.2 (J_{C-F} = 7.0 Hz), 130.2 (J_{C-F} = 7.0 Hz), 114.9(J_{C-F} = 21.3 Hz), 114.9(J_{C-F} = 21.3 Hz), 113.0, 112.5, 83.4, 82.0, 77.3, 53.0, 51.4, 50.7, 49.8, 45.8, 45.5 ppm.

HRMS (ESI): $[M+Na]^+$ calcd for $C_{18}H_{15}FN_2O_2Na^+$: 333.1010, found: 333.1005.

IR (neat): *v* 3423, 2966, 2235, 1714, 1681, 1595, 1510, 1228, 1160, 1082, 838, 734, 518 cm^{-1}.

2-((1*S*,2*R*,3*aS*,5*R*,6*aS*)-1-(4-chlorophenyl)-3*a*,6-dihydroxyoctahydro-2,5-methanopentalen-7-ylidene)malononitrile (16d, 16d')

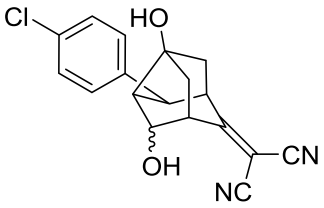

Was prepared following General procedure C. 65.5 mg (80%) obtained as diastereomeric mixture (*dr* ~1.9:1, inseparable). Isolated as a yellow solid. R_f = 0.2 (hexane : ethyl acetate 1:1)

For the major isomer:

^{1}H NMR (CD_3CN, 400 MHz) δ 7.42-7.30 (m, 4H), 4.63 (dd, *J* = 3.6, 6.8 Hz, 1H), 4.00 (br s, 1H), 3.77 (br s, 1H), 3.68 (br s, 1H), 3.40-3.38 (m, 1H), 3.35 (br s, 1H), 2.65 (td, *J* = 2.0, 6.8 Hz, 1H), 2.14-2.12 (m, 1H) 1.70 (d, *J* = 11.6 Hz, 1H) ppm.

^{13}C NMR (CD_3CN, 100 MHz) δ 186.3, 141.6, 132.8, 130.1, 130.8, 129.3, 129.2, 113.0, 112.6, 83.5, 82.0, 77.3, 52.8, 51.4, 50.6, 50.0, 45.8, 45.6 ppm.

HRMS (ESI): $[M+Na]^+$ calcd for $C_{18}H_{15}ClN_2O_2Na^+$: 349.0714, found: 349.0717.

IR(neat) ν 3399, 2968, 2236, 1592, 1492, 1309, 1232, 1086, 1012, 833, 737 cm^{-1}.

2-((1*S*,2*R*,3*aS*,5*R*,6*aS*)-1-(4-bromophenyl)-3*a*,6-dihydroxyoctahydro-2,5-methanopentalen-7-ylidene)malononitrile (16e, 16e')

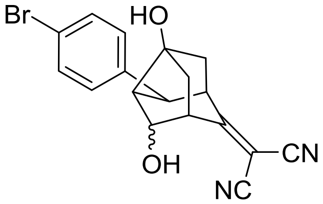

Was prepared following General procedure C. 76.5 mg (82%) obtained as diastereomeric mixture (*dr* ~1.2:1, inseparable). Isolated as a yellow solid. R_f = 0.2 (hexane : ethyl acetate 1:1)

^{1}H NMR (CD_3CN, 400 MHz) δ 7.54 (d, *J* = 8.4 Hz, 2H), 7.32 (d, *J* = 8.0 Hz, 2H), 4.63 (dd, *J* = 3.2, 6.8 Hz, 1H), 3.96 (br s, 1H), 3.96-3.38 (m, 1H), 3.35 (br s, 1H), 3.15 (br s, 1H), 2.85 (td, *J* = 4.0, 11.2 Hz, 1H), 2.64 (td, *J* = 1.6, 6.4 Hz, 1H), 2.05 (td, *J* = 4.0, 12.0 Hz, 1H), 1.70 (d, *J* = 11.6 Hz, 1H) ppm.

^{13}C NMR (CD_3CN, 100 MHz) δ 186.3, 142.1, 132.3, 132.2, 131.4, 131.1, 120.9, 113.0, 112.6, 83.5, 82.0, 81.4, 53.8, 52.7, 51.4, 50.6, 45.8, 45.6 ppm.

HRMS (ESI): $[M+Na]^+$ calcd for $C_{18}H_{15}BrN_2O_2Na^+$: 393.0209, found: 393.0211.

IR(neat) *ν* 3398, 2968, 2235, 1712, 1593, 1489, 1404, 1309, 1232, 1113, 1080, 1009, 946, 830, 793, 737 cm^{-1}.

<u>2-((1*S*,2*R*,3*aS*,5*R*,6*aS*)-1-(3-bromophenyl)-3*a*,6-dihydroxyoctahydro-2,5-methanopentalen-7-ylidene)malononitrile</u> (16f, 16f')

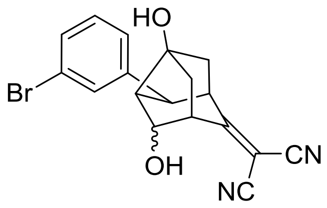

Was prepared following General procedure C. 75.0 mg (81%) obtained as diastereomeric mixture (*dr* ~2.9:1, inseparable). Isolated as a yellow solid. R_f = 0.2 (hexane : ethyl acetate 1:1)

^{1}H NMR (CD_3CN, 400 MHz) δ 7.58 (br s, 1H), 7.45 (d, *J* = 8.0 Hz, 1H), 7.38 (d, *J* = 8.0 Hz, 1H), 7.32 (d, *J* = 8.0 Hz, 1H), 7.26 (br s, 1H), 4.63 (quint, 3.6 Hz, 1H), 4.01 (br s, 1H), 3.78 (dd, *J* = 1.2, 7.6 Hz, 1H), 3.71 (d, *J* = 1.2 Hz, 1H), 3.40-3.36 (m 2H), 2.66 (td, *J* = 1.6, 3.6 Hz, 1H), 1.71 (d, *J* = 11.6 Hz, 1H) ppm.

^{13}C NMR (CD_3CN, 100 MHz) δ 186.1, 145.4, 132.2, 131.2, 130.5, 128.2, 122.9, 112.9, 112.5, 83.6, 82.0, 77.2, 52.7, 51.4, 50.5, 50.2, 45.8, 45.6 ppm.

HRMS (ESI): $[M+Na]^+$ calcd for $C_{18}H_{15}BrN_2O_2Na^+$: 393.0209, found: 393.0209.

IR(neat) *ν* 3439, 2967, 2236, 1593, 1474, 1422, 1308, 1230, 1166, 1081, 981, 926, 782, 727, 705 cm^{-1}.

2-((1*S*,2*R*,3*aS*,5*R*,6*aS*)-1-(2-bromophenyl)-3*a*,6-dihydroxyoctahydro-2,5-methanopentalen-7-ylidene)malononitrile (16g, 16g')

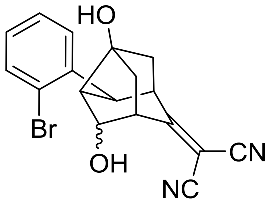

Was prepared following General procedure C. 70.5 mg (76%) obtained as diastereomeric mixture (*dr* ~1.2:1, inseparable). Isolated as a yellow solid. R_f = 0.2 (hexane : ethyl acetate 1:1)

^{1}H NMR (CD_3CN, 400 MHz) δ 7.64 (dd, *J* = 1.2, 8.0 Hz, 1H), 7.56 (d, *J* = 8.0 Hz, 1H), 7.43 (dt, *J* = 1.2, 8.0 Hz, 1H), 7.24 (t, *J* = 8.0 Hz, 1H), 4.65 (dd, *J* = 3.6, 6.8 Hz, 1H), 4.21 (br s, 1H), 4.10 (br s, 1H), 3.41 (br s, 1H), 3.30 (br s, 1H), 2.87 (td, *J* = 4.0, 7.2 Hz, 1H), 2.59 (br s, 1H), 2.07 (td, *J* = 4.0, 12.0 Hz, 1H), 1.80 (d, *J* = 11.6 Hz, 1H) ppm.

^{13}C NMR (CD_3CN, 100 MHz) δ 183.8, 140.1, 133.9, 132.0, 129.8, 128.5, 124.8, 112.8, 112.7, 82.8, 81.5, 77.3, 55.6, 55.3, 53.4, 49.6, 46.5, 45.6 ppm.

HRMS (ESI): $[M+Na]^+$ calcd for $C_{18}H_{15}BrN_2O_2Na^+$: 393.0209, found: 393.0209.

IR(neat) ν 3414, 2968, 2236, 1712, 1594, 1470, 1438, 1310, 1266, 1232, 1084, 738, 669, 571 cm^{-1}.

2-((1*S*,2*R*,3*aS*,5*R*,6*aS*)-1-(furan-2-yl)-3a,6-dihydroxyoctahydro-2,5-methanopentalen-7-ylidene)malononitrile (16h, 16h')

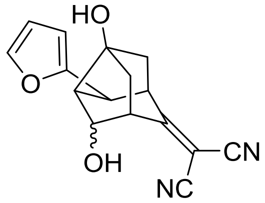

Was prepared following General procedure C. 63.3 mg (90%) obtained as diastereomeric mixture (*dr* ~1.7:1, inseparable). Isolated as a papule solid. R_f = 0.3 (hexane : ethyl acetate 1:1)

^{1}H NMR (CD_3CN, 400 MHz) δ 7.47 (t, *J* = 1.2 Hz, 1H), 6.41 (d, *J* =2.0 Hz, 1H), 6.29 (d, *J* = 3.2 Hz, 1H), 4.59 (quint, *J* = 3.2 Hz, 1H), 3.91 (br s, 1H), 3.81 (br s, 1H), 3.57-3.54 (m, 2H), 3.37 (br s, 1H), 2.54 (td, *J* = 2.0, 6.8 Hz, 1H), 2.16-2.12 (m, 1H), 1.78 (d, *J* = 11.2 Hz, 1H) ppm.

^{13}C NMR (CD_3CN, 100 MHz) δ 185.7, 155.6, 142.9, 112.8, 112.6, 111.3, 108.5, 83.9, 81.8, 77.0, 51.6, 51.0, 50.0, 46.7, 45.6, 44.5 ppm.

HRMS (ESI): $[M+Na]^+$ calcd for $C_{16}H_{14}N_2O_3Na^+$: 305.0897, found: 305.0898.

IR(neat) ν 3367, 2968, 2235, 1595, 1503, 1308, 1230, 1143, 1080, 1015, 944, 738 cm^{-1}.

General procedure D for the benzoylation of secondary alcohol to determine the enantiomeric excess

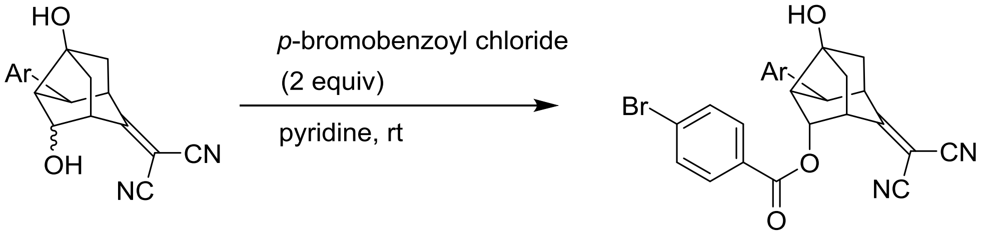

To a solution of secondary alcohol **16** (0.10 mmol) in pyridine (0.3 M), *p*-bromobenzoyl choride (2 equiv.) was added. After the consumption of starting material, the reaction mixture was quenched by 2N HCl. The residue was diluted by EtOAc. After separation, the organic layer was washed with 2N HCl and aq. sat. $NaHCO_3$ for two times. The combined organic layers were evaporated under reduced pressure. The residue was purified by column chromatography on silica gel (*n*-Hexane: EtOAc = 12:1) to give corresponding benzoylated compounds. The major diastereoisomer was isolated to determine the *ee*.

<u>(1*S*,2*R*,3*aS*,5*R*,6*S*,6*aS*)-7-(dicyanomethylene)-3*a*-hydroxy-6-phenyloctahydro-2,5-methanopentalen-1-yl 4-bromobenzoate</u> (24a)

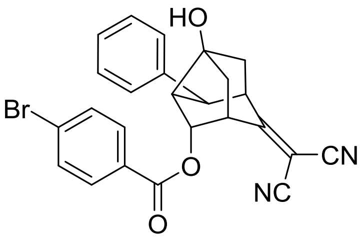

Was prepared following General procedure D. 20.0 mg (42%) obtained as a white solid (m.p. 108-110 °C). R_f = 0.55 (hexane : ethyl acetate 3:1)

^{1}H NMR (acetone-d$_6$, 400 MHz): δ 7.99 (d, *J* = 8.8 Hz, 2H), 7.71 (d, *J* = 8.6 Hz, 2H), 7.46 (d, *J* = 7.8 Hz, 2H), 7.37 (t, *J* = 7.5 Hz, 2H), 7.26 (t, *J* = 7.2 Hz, 1H), 5.70 (dd, *J* = 6.8, 3.4 Hz, 1H), 3.95 (br s, 1H), 3.85 – 3.79 (m, 1H), 3.51 (br s, 1H), 3.18 (d, *J* = 7.1 Hz, 1H), 2.50 (td, *J* = 11.7, 3.6 Hz, 1H), 2.37 (td, *J* = 11.8, 3.7 Hz, 1H), 2.29 (d, *J* = 11.8 Hz, 1H), 1.92 (d, *J* = 11.8 Hz, 1H) ppm.

^{13}C NMR (acetone-d$_6$, 100 MHz): δ 184.0, 165.1, 141.9, 132.9, 132.2, 129.6, 129.3, 129.2, 128.9, 127.7, 112.4, 112.4, 84.1, 82.2, 79.7, 53.0, 51.5, 49.1, 48.7, 45.9, 45.7 ppm.

HRMS (ESI): $[M+Na]^+$ calcd for $C_{25}H_{19}BrN_2O_3Na^+$: 497.0471, found: 497.0466.

IR(neat) ν 3477, 2975, 2235, 1724, 1593, 1481, 1396, 1269, 1173, 1097, 848, 754, 703, 549 cm^{-1}.

The enantiomeric ratio was determined by HPLC using CHIRALPACK® IG (*n*-Hexane: *i*-PrOH = 9:1; flow rate 1.0 mL/min, major isomer t_R = 12.5 min, minor isomer t_R = 17.7 min) (96% *ee*).

$[\alpha]_D^{26}$ -46.7 (*c* 2.0, MeCN)

(1*S*,2*R*,3*aS*,5*R*,6*S*,6*aS*)-7-(dicyanomethylene)-3*a*-hydroxy-6-(4-methoxyphenyl)octahydro-2,5-methanopentalen-1-yl 4-bromobenzoate (24b)

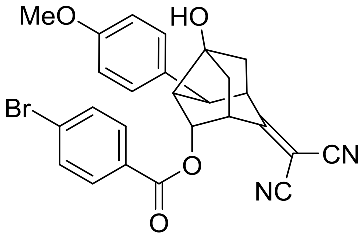

Was prepared following General procedure D. 10.5 mg (21%) obtained as a white solid (m.p. 112-115 °C). R_f = 0.45 (hexane : ethyl acetate 3:1)

^{1}H NMR (CD_3CN, 400 MHz) δ 7.93 (d, *J* = 8.8 Hz, 2H), 7.69 (d, *J* = 8.4 Hz, 2H), 7.30 (d, *J* = 8.4 Hz, 2H), 6.93 (d, *J* = 8.8 Hz, 2H), 5.60 (dd, *J* = 3.2, 6.8 Hz, 1H), 3.78 (s, 3H), 3.76 (br s, 1H), 3.73 (dt, *J* = 1.6, 3.2 Hz, 1H), 3.42 (br s, 1H), 3.03 (td, *J* = 1.6, 4.4 Hz, 1H), 2.34 (td, *J* = 3.6, 11.6 Hz, 1H), 2.25 (td, *J* = 4.0, 11.6 Hz, 1H), 2.14 (dd, *J* = 1.2, 11.6 Hz, 1H), 1.99 (s, 1H), 1.79 (d, *J* = 11.6 Hz, 1H) ppm.

^{13}C NMR (CD_3CN, 100 MHz) δ 184.3, 165.4, 159.4, 133.6, 132.9, 132.9, 132.3,132.3, 130.2, 130.2, 129.5, 129.0, 114.6, 114.6, 112.6, 112.5, 84.2, 82.1, 79.6, 55.8, 53.0, 50.6, 49.1, 48.6, 45.6, 45.5 ppm.

HRMS (ESI): $[M+Na]^+$ calcd for $C_{26}H_{21}BrN_2O_4Na^+$: 527.0577 found: 527.0575.

IR(neat) ν 3484, 2968, 2235, 1724, 1592, 1514, 1480, 1396, 1266, 1176, 1095, 1037, 1011,841, 755, 524 cm^{-1}.

The enantiomeric ratio was determined by HPLC using CHIRALPACK® IG (*n*-Hexane: *i*-PrOH = 9:1; flow rate 1.0 mL/min, major isomer t_R = 24.4 min, minor isomer t_R = 16.4 min) (93% *ee*).

$[\alpha]_D^{26}$ -31.3 (*c* 1.0, MeCN)

(1*S*,2*R*,3*aS*,5*R*,6*S*,6*aS*)-7-(dicyanomethylene)-6-(4-fluorophenyl)-3*a*-hydroxyoctahydro-2,5-methanopentalen-1-yl 4-bromobenzoate (24c)

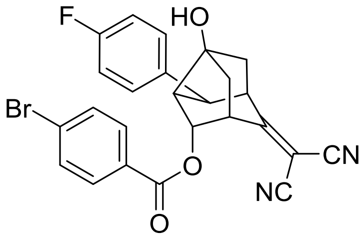

Was prepared following General procedure D. 25.4 mg (52%) obtained as a white solid (m.p. 107-109 °C). R_f = 0.55 (hexane : ethyl acetate 3:1)

^{1}H NMR (CD_3CN, 400 MHz) δ 7.92 (d, *J* = 8.4 Hz, 2H), 7.69 (d, *J* = 8.4 Hz, 2H), 7.41 (dd, *J* = 5.2, 8.4 Hz, 2H), 7.12 (t, *J* = 8.8 Hz, 2H), 5.60 (dd, *J* = 3.6, 10.8 Hz, 1H), 3.81 (br s, 1H), 3.74 (dt, *J* = 1.6, 3.6 Hz, 1H), 3.45 (t, *J* = 1.2 Hz, 1H), 3.05 (td, *J* = 2.0, 6.8 Hz, 1H), 2.35 (td, *J* = 4.0, 12.0 Hz, 1H), 2.21 (dd, *J* = 4.0, 12.0 Hz, 1H), 2.15 (dd, *J* = 1.2, 12.0 Hz, 1H), 1.81 (d, *J* = 11.6 Hz, 1H) ppm.

^{13}C NMR (CD_3CN, 100 MHz) δ 183.8, 165.4, 162.5 (J_{C-F} = 242 Hz), 137.8 (J_{C-F} = 3.1 Hz), 132.9, 132.9, 132.2, 132.2, 131.0 (J_{C-F} = 8.1 Hz), 131.0 (J_{C-F} = 8.1 Hz), 129.4, 129.0, 115.9 (J_{C-F} = 21.4 Hz), 115.9 (J_{C-F} = 21.4 Hz), 112.5, 112.4, 84.5, 82.1, 79.4, 52.8, 50.5, 49.1, 48.6, 45.5, 45.5 ppm.

HRMS (ESI): $[M+Na]^+$ calcd for $C_{25}H_{18}BrFN_2O_3Na^+$: 515.0377, found: 515.0378.

IR(neat) ν 3477, 2975, 2235, 1724, 1593, 1512, 1481, 1396, 1268, 1168, 1102, 1049, 1011, 843 cm^{-1}.

The enantiomeric ratio was determined by HPLC using CHIRALPACK® IG (*n*-Hexane: *i*-PrOH = 9:1; flow rate 0.50 mL/min, major isomer t_R = 31.5 min, minor isomer t_R = 29.1 min) (>99% *ee*).

$[\alpha]_D^{26}$ -53.4 (*c* 2.2, MeCN)

(1*S*,2*R*,3*aS*,5*R*,6*S*,6*aS*)-6-(4-chlorophenyl)-7-(dicyanomethylene)-3*a*-hydroxyoctahydro-2,5-methanopentalen-1-yl 4-bromobenzoate (24d)

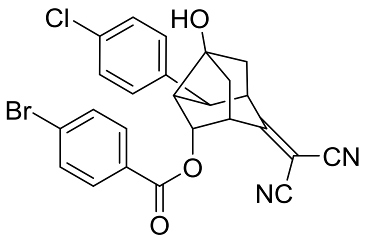

Was prepared following General procedure D. 28.5 mg (56%) obtained as a white solid (m.p. 115-118 °C). R_f = 0.55 (hexane : ethyl acetate 3:1)

^{1}H NMR (CD_3CN, 400 MHz) δ 7.92 (d, *J* = 8.8 Hz, 2H), 7.68 (d, *J* = 8.8 Hz, 2H), 7.38 (s, 4H), 5.60 (dd, *J* = 3.2, 6.8 Hz, 1H), 3.80 (br s, 1H), 3.74 (d, *J* = 1.2 Hz, 1H), 3.47 (br s, 1H), 3.05 (td, *J* = 1.6, 6.4 Hz, 1H), 2.35 (td, *J* = 3.6, 12.0 Hz, 1H), 2.20 (td, *J* = 3.6, 11.6 Hz, 1H), 2.15 (d, *J* = 11.6 Hz, 1H), 1.81 (d, *J* = 12.0 Hz, 1H) ppm.

^{13}C NMR (CD_3CN, 100 MHz) δ 183.6, 165.4, 140.7, 133.0, 132.8, 132.8, 132.3, 132.3, 130.9, 130.9, 129.4, 129.3, 129.3, 129.0, 112.5, 112.4, 84.5, 82.1, 79.4, 52.6, 50.6, 50.0, 48.6, 45.5, 45.5 ppm.

HRMS (ESI): $[M+Na]^+$ calcd for $C_{25}H_{18}BrClN_2O_3Na^+$: 531.0082, found: 531.0083.

IR(neat) ν 3491, 2970, 2236, 1722, 1592, 1490, 1398, 1272, 1173, 1094, 1013, 927, 846, 815, 754, 738 cm^{-1}.

The enantiomeric ratio was determined by HPLC using CHIRALPACK® IG (*n*-Hexane: *i*-PrOH = 9:1; flow rate 1.0 mL/min, major isomer t_R = 15.8 min, minor isomer t_R = 10.4 min) (96% *ee*).

$[\alpha]_D^{26}$ -42.4 (*c* 2.8, MeCN)

(1*S*,2*R*,3*aS*,5*R*,6*S*,6*aS*)-6-(4-bromophenyl)-7-(dicyanomethylene)-3*a*-hydroxyoctahydro-2,5-methanopentalen-1-yl 4-bromobenzoate (24e)

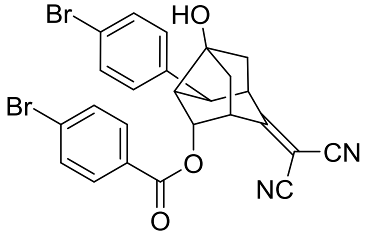

Was prepared following General procedure D. 33.3 mg (60%) obtained as a white solid (m.p. 118-120 °C). R_f = 0.55 (hexane : ethyl acetate 3:1)

^{1}H NMR (CD_3CN, 400 MHz) δ 7.92 (d, *J* = 8.8 Hz, 2H), 7.67 (d, *J* = 8.8 Hz, 2H), 7.52 (d, *J* = 8.4 Hz, 2H), 7.31 (d, *J* = 8.0 Hz, 2H), 5.60 (dd, *J* = 3.6, 6.8 Hz, 1H), 3.78 (br s, 1H), 3.74 (dt, *J* = 1.2, 3.2 Hz, 1H), 3.47 (t, *J* = 1.2 Hz, 1H), 3.04 (td, *J* = 2.0, 6.8 Hz, 1H), 2.34 (td, *J* = 3.6, 11.6 Hz, 1H), 2.19 (td, *J* = 3.6, 12.0 Hz, 1H), 2.14 (dd, *J* = 0.80, 11.6 Hz, 1H), 1.81 (d, *J* = 11.6 Hz, 1H) ppm.

^{13}C NMR (CD_3CN, 100 MHz) δ 183.6, 165.4, 141.1, 132.8, 132.8, 132.3, 132.3, 132.3, 132.3, 131.2, 131.2, 129.4, 129.0, 121.2, 112.5, 112.4, 84.5, 82.1, 79.4, 52.5, 50.7, 48.9, 48.6, 45.5, 45.5 ppm.

HRMS (ESI): $[M+Na]^+$ calcd for $C_{25}H_{18}Br_2N_2O_3Na^+$: 574.9576, found: 574.9576.

IR(neat) ν 3483, 2974, 2235, 1724, 1593, 1489, 1397, 1270, 1173, 1095, 1011, 845, 816, 754, 568 cm^{-1}.

The enantiomeric ratio was determined by HPLC using CHIRALPACK® IE (*n*-Hexane: *i*-PrOH = 9:1; flow rate 1.0 mL/min, major isomer t_R = 9.61 min, minor isomer t_R = 8.71 min) (98% *ee*).

$[\alpha]_D^{26}$ -30.3 (*c* 2.6, MeCN)

(1*S*,2*R*,3*aS*,5*R*,6*S*,6*aS*)-6-(3-bromophenyl)-7-(dicyanomethylene)-3*a*-hydroxyoctahydro-2,5-methanopentalen-1-yl 4-bromobenzoate (24f)

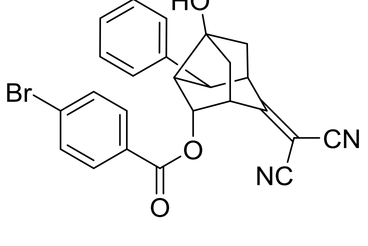

Was prepared following General procedure D. 32.5 mg (59%) obtained as a white solid (m.p. 103-106 ^{o}C). R_f = 0.55 (hexane : ethyl acetate 3:1)

^{1}H NMR (CD$_3$CN, 400 MHz) δ 7.93 (d, *J* = 8.8 Hz, 2H), 7.70 (d, *J* = 8.8 Hz, 2H), 7.58 (s, 1H), 7.45 (d, *J* = 8.0 Hz, 1H), 7.39 (d, *J* = 8.0 Hz, 1H), 7.31 (t, *J* = 8.0 Hz, 1H), 5.61 (dd, *J* = 3.6, 6.8 Hz, 1H), 3.83 (br s, 1H), 3.74 (dd, *J* = 1.6, 2.8 Hz, 1H), 3.49 (br s, 1H), 3.05 (td, *J* = 2.0, 6.8 Hz, 1H), 2.35 (td, *J* = 3.6, 11.6 Hz, 1H), 2.23-2.13 (m, 2H), 1.83 (d, *J* = 12.0 Hz, 1H) ppm.

^{13}C NMR (CD$_3$CN, 100 MHz) δ 183.5, 165.4, 144.5, 132.8, 132.8, 132.3, 132.3, 132.1, 131.2, 130.7, 129.4, 129.0, 128.1, 122.9, 112.5, 112.4, 84.6, 82.1, 79.3, 52.5, 50.9, 48.8, 48.6, 45.5, 45.5 ppm.

HRMS (ESI): $[M+Na]^+$ calcd for $C_{25}H_{18}Br_2N_2O_3Na^+$: 574.9576, found: 574.9576.

IR(neat) ν 3484, 2975, 2235, 1724, 1593, 1479, 1425, 1396, 1374, 1268, 1173, 1095, 1049, 1011, 954, 906, 848, 782, 755, 738, 706, 683, 642, 576 cm^{-1}.

The enantiomeric ratio was determined by HPLC using CHIRALPACK® IH (*n*-Hexane: *i*-PrOH = 9:1; flow rate 1.0 mL/min, major isomer t_R = 17.4 min, minor isomer t_R = 15.6 min) (98% *ee*).

$[\alpha]_D^{26}$ -30.8 (*c* 3.3, MeCN)

(1*S*,2*R*,3*aS*,5*R*,6*S*,6*aS*)-6-(2-bromophenyl)-7-(dicyanomethylene)-3*a*-hydroxyoctahydro-2,5-methanopentalen-1-yl 4-bromobenzoate (24g)

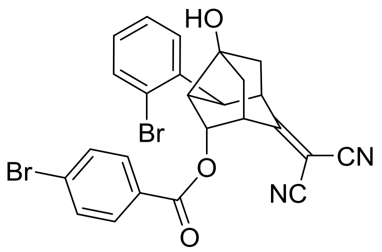

Was prepared following General procedure D. 14.8 mg (27%) obtained as a white solid (m.p. 124-127 ^{o}C). R_f = 0.55 (hexane : ethyl acetate 3:1)

Yield: 27% (14.8 mg)

Physical state : White solid (m.p. 124~127 ºC)

^{1}H NMR (CD$_3$CN, 400 MHz) δ 7.91 (d, *J* = 8.4 Hz, 2H), 7.68 (d, *J* = 8.4 Hz, 2H), 7.61 (dd, *J* = 1.2, 8.0 Hz, 1H), 7.56 (dd, *J* = 1.6, 7.6 Hz, 1H), 7.42 (dt, *J* = 1.6, 8.0 Hz, 1H), 7.21 (dt, *J* = 1.6, 7.6 Hz, 1H), 5.65 (dd, *J* = 3.2, 6.8 Hz, 1H), 4.06 (br s, 1H), 3.76 (dd, *J* = 1.6, 3.2 Hz, 1H), 3.64 (br s, 1H), 3.01 (td, *J* = 1.6, 6.4 Hz, 1H), 2.38 (td, *J* = 3.6, 12.0 Hz, 1H), 2.25 (td, *J* = 4.0, 12.0 Hz, 1H), 2.18 (dd, *J* = 0.40, 11.6 Hz, 1H), 1.85 (d, *J* = 12.4 Hz, 1H) ppm.

^{13}C NMR (CD$_3$CN, 100 MHz) δ 183.1, 165.2, 140.1, 133.8, 133.0, 133.0, 132.6, 132.3, 132.1, 132.1, 131.9, 130.0, 129.5, 128.6, 125.3, 112.5, 112.3, 84.6, 82.4, 79.1, 52.4, 50.7, 49.3, 48.6, 45.4 ppm.

HRMS (ESI): $[M+Na]^+$ calcd for $C_{25}H_{18}Br_2N_2O_3Na^+$: 574.9576, found: 574.9575.

IR(neat) ν 3492, 2976, 2236, 1787, 1725, 1592, 1476, 1437, 1396, 1269, 1173, 1094, 1051, 1012, 954, 926, 847, 754, 677, 568 cm^{-1}.

The enantiomeric ratio was determined by HPLC using CHIRALPACK® IH (*n*-Hexane: *i*-PrOH = 9:1; flow rate 1.0 mL/min, major isomer t_R = 18.9 min, minor isomer t_R = 17.7 min) (98% *ee*).

$[\alpha]_D^{26}$ +114 (*c* 1.4, MeCN)

(1*S*,2*R*,3*aS*,5*R*,6*S*,6*aS*)-7-(dicyanomethylene)-6-(furan-2-yl)-3*a*-hydroxyoctahydro-2,5-methanopentalen-1-yl 4-bromobenzoate (24h)

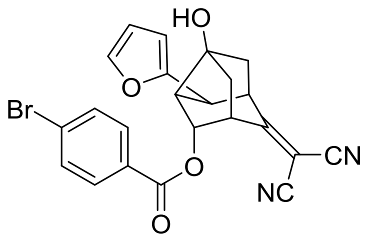

Was prepared following General procedure D. 11.0 mg (24%) obtained as a white solid (m.p. 92.5-95.5 °C). R_f = 0.55 (hexane : ethyl acetate 3:1)

^{1}H NMR (CD_3CN, 400 MHz) δ 7.94 – 7.89 (m, 2H), 7.71 – 7.67 (m, 2H), 7.50 – 7.43 (m, 1H), 6.42 – 6.37 (m, 1H), 6.32 (d, *J* = 3.4 Hz, 1H), 5.57 (dd, *J* = 6.8, 3.5 Hz, 1H), 3.75 (br s, 1H), 3.74 – 3.72 (m, 1H), 3.67 (br s, 1H), 2.93 (dt, *J* = 6.7, 1.6 Hz, 1H), 2.31 (dt, *J* = 11.9, 3.8 Hz, 1H), 2.22 (dt, *J* = 11.8, 3.8 Hz, 1H), 2.13 (d, *J* = 11.6 Hz, 1H), 1.89 (d, *J* = 11.6 Hz, 1H) ppm.

^{13}C NMR (CD_3CN, 100 MHz) δ 182.9, 165.4, 154.5, 143.1, 132.9, 132.6, 132.3, 129.4, 129.0, 112.4, 111.3, 108.8, 85.0, 81.9, 79.1, 49.9, 49.2, 48.7, 46.6, 45.5, 45.4 ppm.

HRMS (ESI): $[M+Na]^+$ calcd for $C_{23}H_{17}BrN_2O_4Na^+$: 487.0264, found: 487.0267.

IR(neat) ν 3487, 2974, 2235, 1787, 1723, 1592, 1481, 1396, 1267, 1173, 1094, 1011, 937, 846, 811, 738, 600, 549 cm^{-1}.

The enantiomeric ratio was determined by HPLC using CHIRALPACK® IG (*n*-Hexane: *i*-PrOH = 9:1; flow rate 0.50 mL/min, major isomer t_R = 27.0 min, minor isomer t_R = 35.4 min) (82% *ee*).

$[\alpha]_D^{26}$ -50.9 (*c* 1.1, MeCN)

Synthesis of compounds 17i and 17j

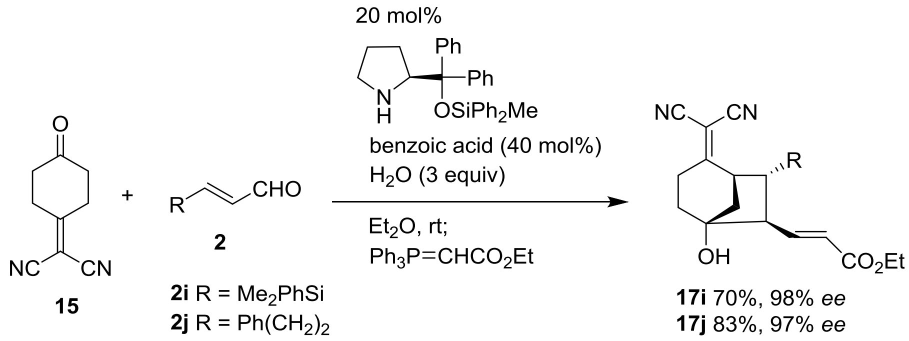

ethyl (*E*)-3-((1*S*,5*S*,6*S*,7*S*)-2-(dicyanomethylene)-7-(dimethyl(phenyl)silyl)-5-hydroxybicyclo[3.2.1]octan-6-yl)acrylate (17i)

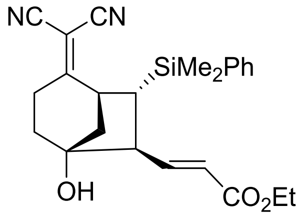

To a solution of ketone (40.0 mg, 0.25 mmol) and (*E*)-3-(dimethyl(phenyl)silyl)acrylaldehyde (57.0 mg, 0.30 mmol) in Et_2O (600 μL), H_2O (13.5 μL, 0.75 mmol), diphenylprolinol silyl ether (22.4 mg, 0.050 mmol) and benzoic acid (12.2 mg, 0.10 mmol) were added at room temperature. After stirring the reaction mixture at this temperature for 3 hours, the solvent was removed under reduced pressure. To a solution of the residue in toluene (600 μL), Wittig reagent (104.5 mg, 0.30 mmol) was added. The residue was directly purified by column chromatography on silica gel (*n*-Hexane: EtOAc = 10/1) to give the target compound (73.1 mg, 0.174 mmol) in 70% yield.

^{1}H NMR (400 MHz, $CDCl_3$): δ 7.49 – 7.39 (m, 5H), 6.79 (dd, *J* = 15.5, 10.1 Hz, 1H), 5.82 (d, *J* = 15.5 Hz, 1H), 4.20 (q, *J* = 6.9 Hz, 2H), 3.50 (t, *J* = 4.7 Hz, 1H), 2.67 (t, *J* = 8.9 Hz, 1H), 2.52 – 2.45 (m, 1H), 2.40 (br s, 1H), 2.24 – 2.17 (m, 1H), 1.77 – 1.69 (m, 3H), 1.65 – 1.55 (m, 2H), 1.31 (t, *J* = 7.1 Hz, 3H), 0.42 (s, 3H), 0.31 (s, 3H) ppm.

^{13}C NMR (100 MHz, $CDCl_3$): δ 184.2, 166.1, 149.2, 135.3, 133.7, 130.2, 128.4, 122.7, 111.3, 111.2, 83.3, 79.3, 60.6, 48.8, 45.0, 44.9, 40.1, 39.0, 28.1, 14.2, -2.5, -3.8 ppm.

IR (neat): ν 2979, 2236, 1709, 1649, 1589, 1426, 1370, 1275, 1119, 832 cm^{-1}

HRMS (ESI) *m/z*: calcd for $C_{24}H_{29}N_2O_3Si$: 421.1942 $[M+H]^+$; found: 421.1938.

$[a]_D^{23}$: -78.1 (c = 1.0, $CHCl_3$)

HPLC (Daicel Chiralpak AD-H column, *n*-hexane/isopropanol = 9/1, flow 1.0 mL/min, detection at 230 nm) retention time = 9.6 min (minor) and 10.5 min (major), 98% *ee*.

<u>ethyl (*E*)-3-((1*R*,5*S*,6*R*,7*S*)-2-(dicyanomethylene)-5-hydroxy-7-phenethylbicyclo[3.2.1]octan-6-yl)acrylate</u> (17j)

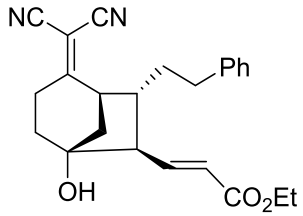

To a solution of ketone (40.0 mg, 0.25 mmol) and (*E*)-5-phenylpent-2-enal (48 mg, 0.30 mmol) in Et_2O (600 μL), H_2O (13.5 μL, 0.75 mmol), diphenylprolinol silyl ether (22.4 mg, 0.050 mmol) and benzoic acid (12.2 mg, 0.10 mmol) were added at room temperature. After stirring the reaction mixture at this temperature for 3 hours, the solvent was removed under reduced pressure. To a solution of the residue in toluene (600 μL), Wittig reagent (104.5 mg, 0.30 mmol) was added. The residue was directly purified by column chromatography on silica gel (*n*-Hexane: EtOAc = 10/1) to give the target compound (97 mg, 0.25 mmol) as a yellow oil in 83% yield.

^{1}H NMR (400 MHz, $CDCl_3$): δ 7.31 – 7.26 (m, 2H), 7.24 – 7.20 (m, 1H), 7.13 (d, *J* = 7.1 Hz, 2H), 6.89 (dd, *J* = 15.6, 9.8 Hz, 1H), 5.85 (d, *J* = 15.6 Hz, 1H), 4.25 – 4.17 (m, 2H), 3.52 (t, *J* = 4.9 Hz, 1H), 3.16 (dd, *J* = 18.1, 6.6 Hz, 1H), 2.94 (br s, 1H), 2.64 – 2.50 (m, 3H), 2.40 – 2.28 (m, 2H), 2.26 – 2.18 (m, 1H), 2.04 – 1.87 (m, 2H), 1.71 (d, *J* = 11.7 Hz, 1H), 1.69 – 1.61 (m, 1H), 1.53 – 1.42 (m, 1H), 1.31 (t, *J* = 7.2 Hz, 3H) ppm.

^{13}C NMR (100 MHz, $CDCl_3$): δ 182.6, 166.1, 148.0, 140.4, 128.6, 128.1, 126.3, 123.0, 111.3, 111.3, 84.7, 77.8, 60.7, 53.2, 49.2, 46.0, 42.9, 39.6, 34.0, 31.9, 28.9, 14.2 ppm.

IR (neat): v 3463, 2939, 2236, 1709, 1653, 1589, 1451, 1303, 1275, 1126, 1031 cm^{-1}.

HRMS (ESI) *m/z*: calcd for $C_{24}H_{26}N_2NaO_3$: 413.1836 $[M+Na]^+$; found: 413.1839.

$[a]_D^{24}$: -43.4 (c = 2.0, $CHCl_3$)

HPLC (Daicel Chiralpak OJ-H column, *n*-hexane/isopropanol = 9/1, flow 1.0 mL/min, detection at 214 nm) retention time = 33.7 min (minor) and 42.1 min (major), 97% *ee*.

5. NMR spectral data

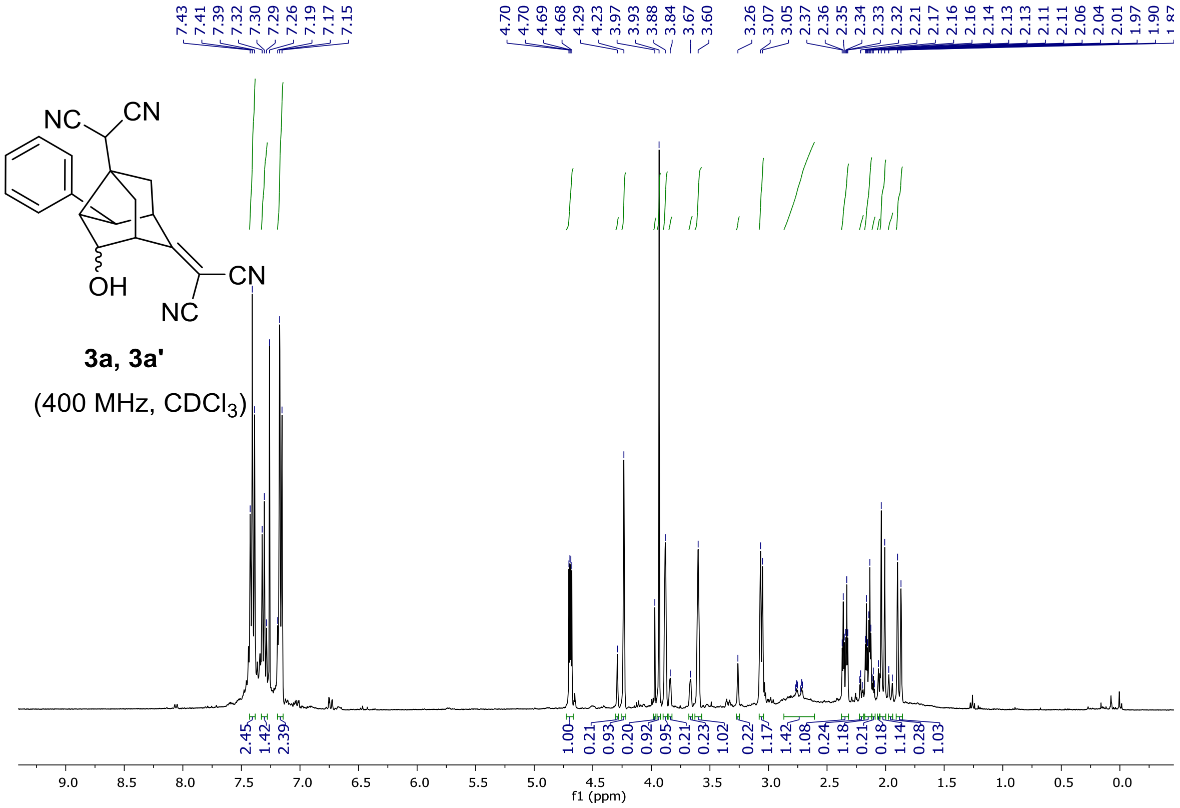

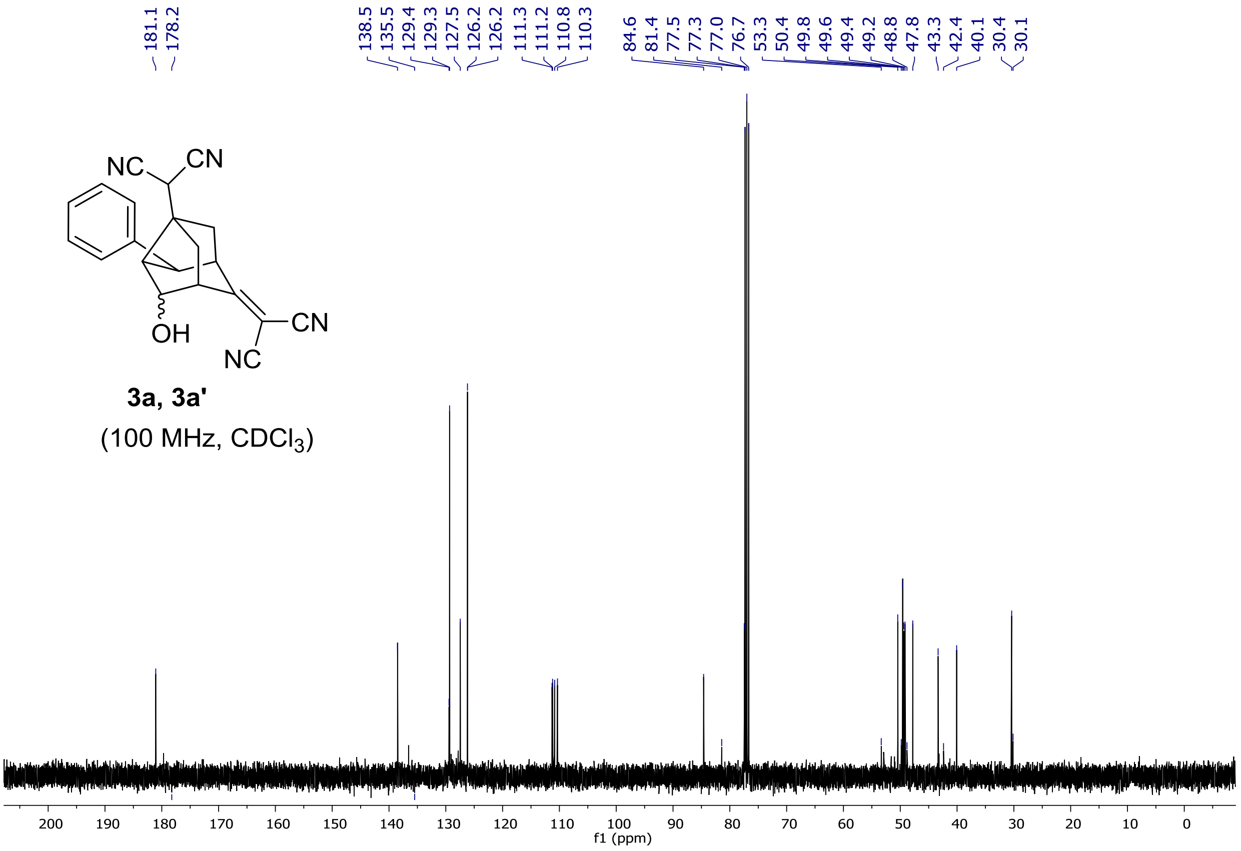

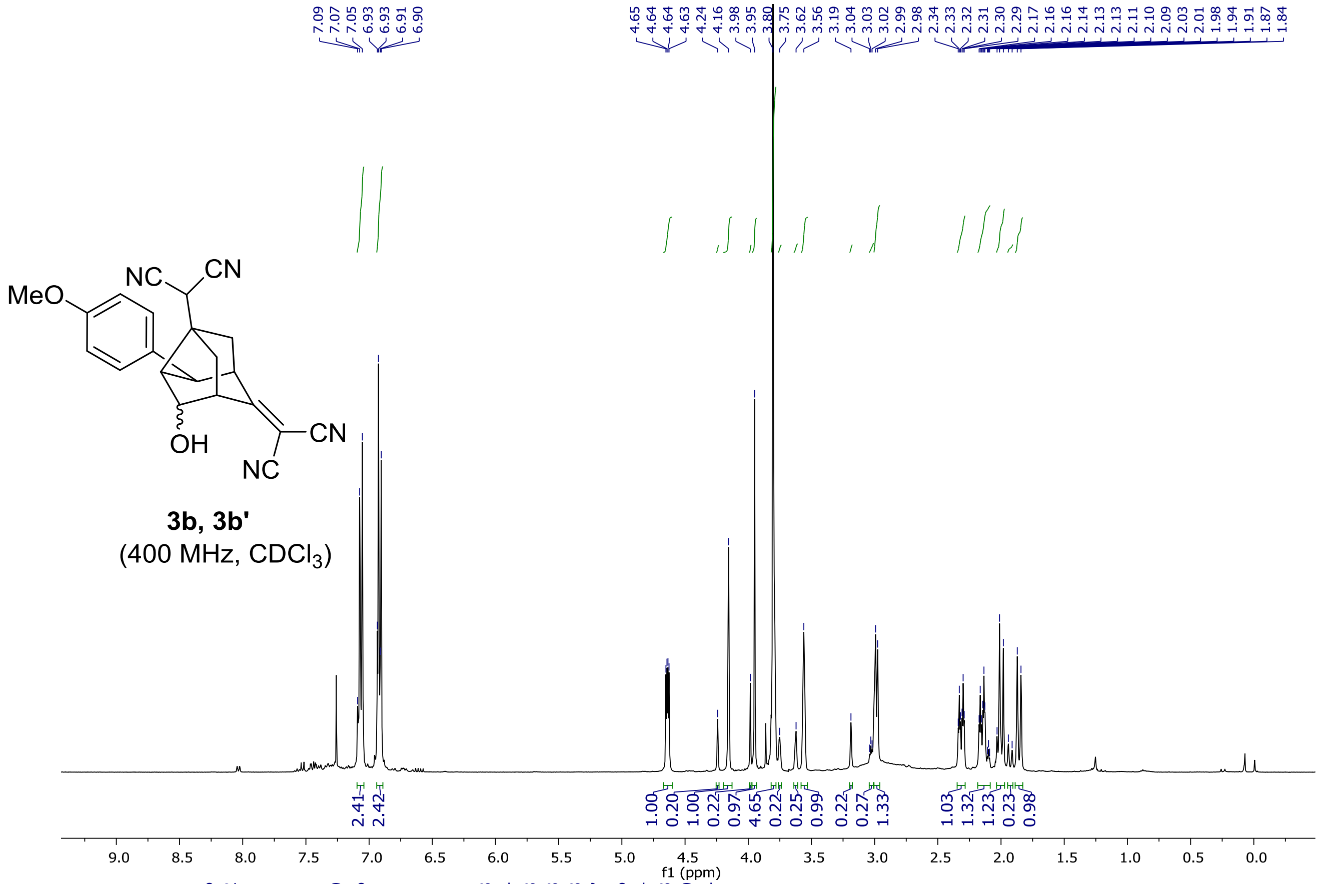
7.09 7.07 7.05 6.93 6.93 6.91 6.90
4.65 4.64 4.64 4.63 4.24 4.16 3.98 3.95 3.80 3.75 3.62 3.56 3.19 3.04 3.03 3.02 2.99 2.98 2.34 2.33 2.32 2.31 2.30 2.29 2.17 2.16 2.14 2.13 2.13 2.11 2.10 2.09 2.03 2.01 1.98 1.94 1.91 1.87 1.84
MeO
NC
CN
OH
NC
CN
3b, 3b'
(400 MHz, CDCl3)
2.41 2.42
1.00 0.20 1.00 0.22 0.97 4.65 0.22 0.25 0.99 0.22 0.27 1.33
1.03 1.32 1.23 0.23 0.98
9.0 8.5 8.0 7.5 7.0 6.5 6.0 5.5 5.0 4.5 4.0 3.5 3.0 2.5 2.0 1.5 1.0 0.5 0.0
f1 (ppm)

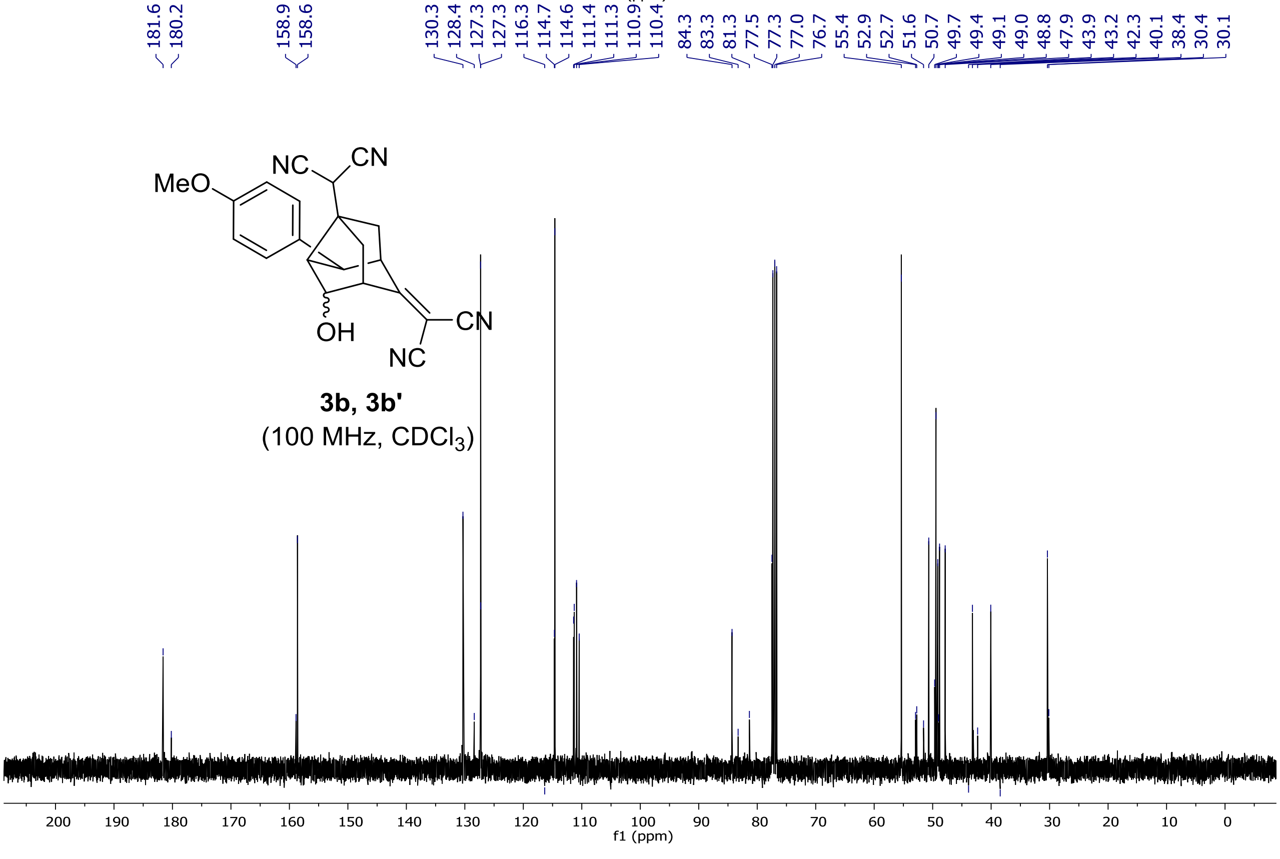
181.6 180.2 158.9 158.6 130.3 128.4 127.3 127.3 116.3 114.7 114.6 111.4 111.3 110.9 110.4 84.3 83.3 81.3 77.5 77.3 77.0 76.7 55.4 52.9 52.7 51.6 50.7 49.7 49.4 49.1 49.0 48.8 47.9 43.9 43.2 42.3 40.1 38.4 30.4 30.1
MeO
NC
CN
OH
NC
CN
3b, 3b'
(100 MHz, CDCl3)
200 190 180 170 160 150 140 130 120 110 100 90 80 70 60 50 40 30 20 10 0
f1 (ppm)

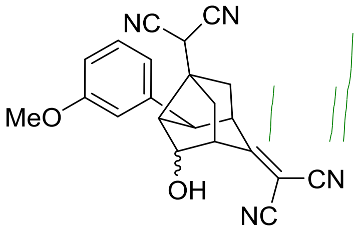

3c, 3c'

(400 MHz, $CDCl_3$)

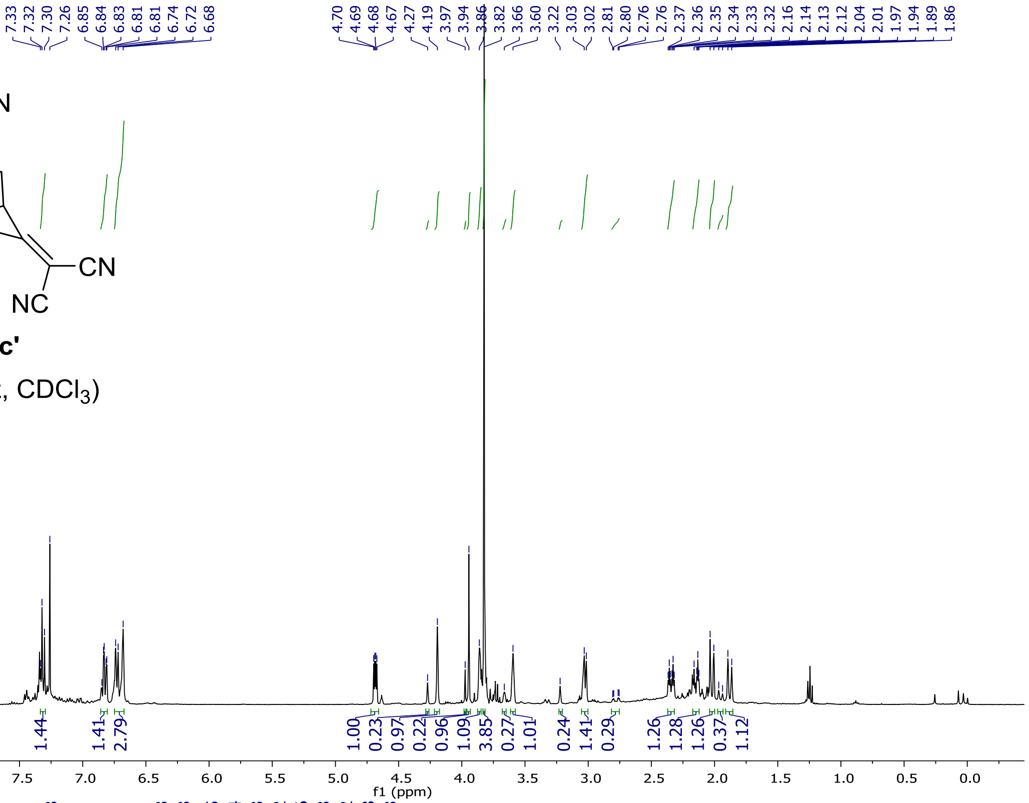

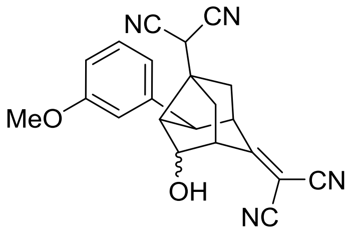

3c, 3c'

(100 MHz, $CDCl_3$)

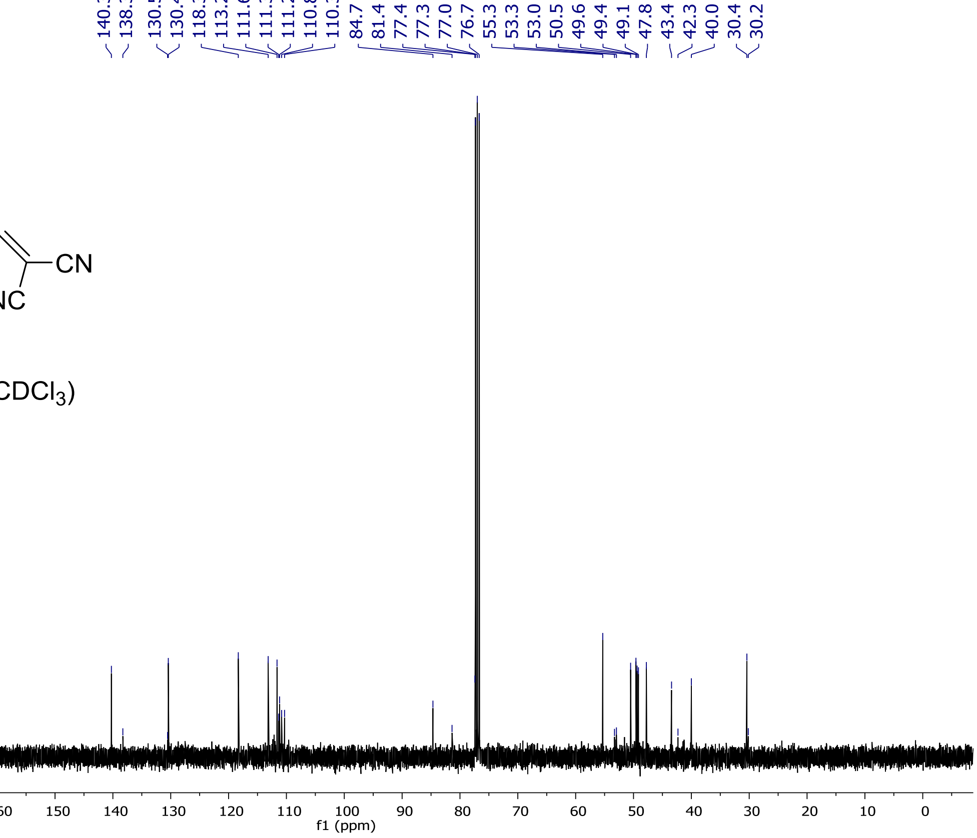

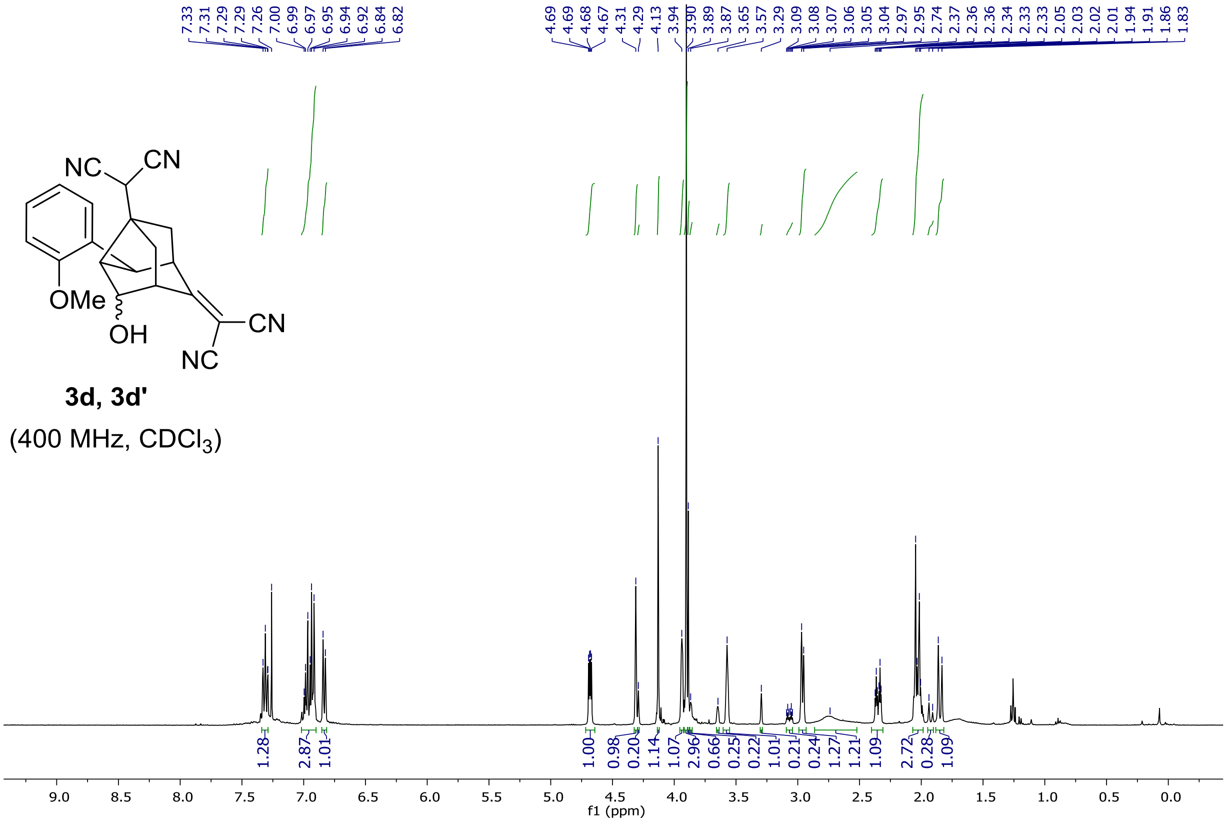

7.33 7.31 7.29 7.29 7.26 7.00 6.99 6.97 6.95 6.94 6.92 6.84 6.82
4.69 4.69 4.68 4.67 4.31 4.29 4.13 3.94 3.90 3.89 3.87 3.65 3.57 3.29 3.09 3.08 3.07 3.06 3.05 3.04 2.97 2.95 2.74 2.37 2.36 2.36 2.34 2.33 2.33 2.05 2.03 2.02 2.01 1.94 1.91 1.86 1.83
NC
CN
OMe
OH
CN
NC
3d, 3d'
(400 MHz, CDCl$_3$)
1.28 2.87 1.01 1.00 0.98 0.20 1.14 1.07 2.96 0.66 0.25 0.22 1.01 0.21 0.24 1.27 1.21 1.09 2.72 0.28 1.09
f1 (ppm)

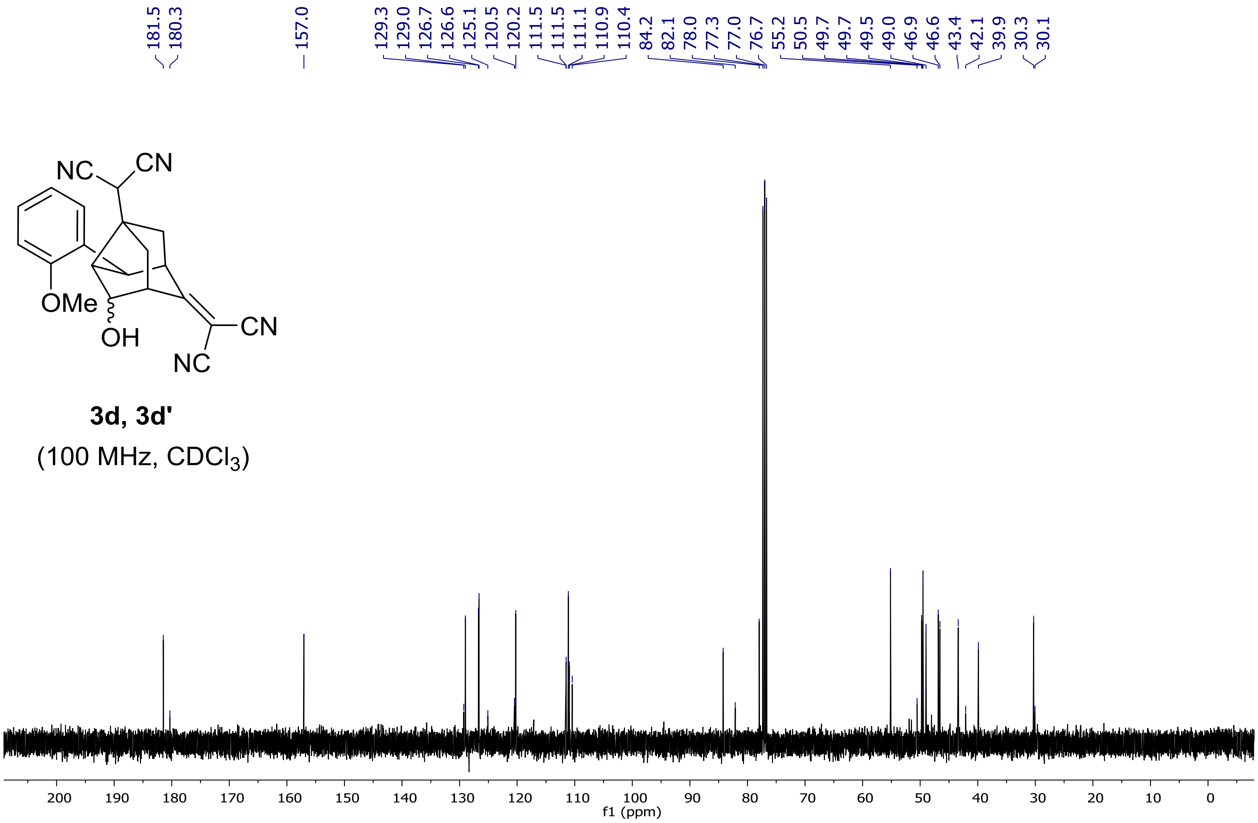

181.5 180.3 157.0 129.3 129.0 126.7 126.6 125.1 120.5 120.2 111.5 111.5 111.1 110.9 110.4 84.2 82.1 78.0 77.3 77.0 76.7 55.2 50.5 49.7 49.7 49.5 49.0 46.9 46.6 43.4 42.1 39.9 30.3 30.1
NC
CN
OMe
OH
CN
NC
3d, 3d'
(100 MHz, CDCl$_3$)
f1 (ppm)

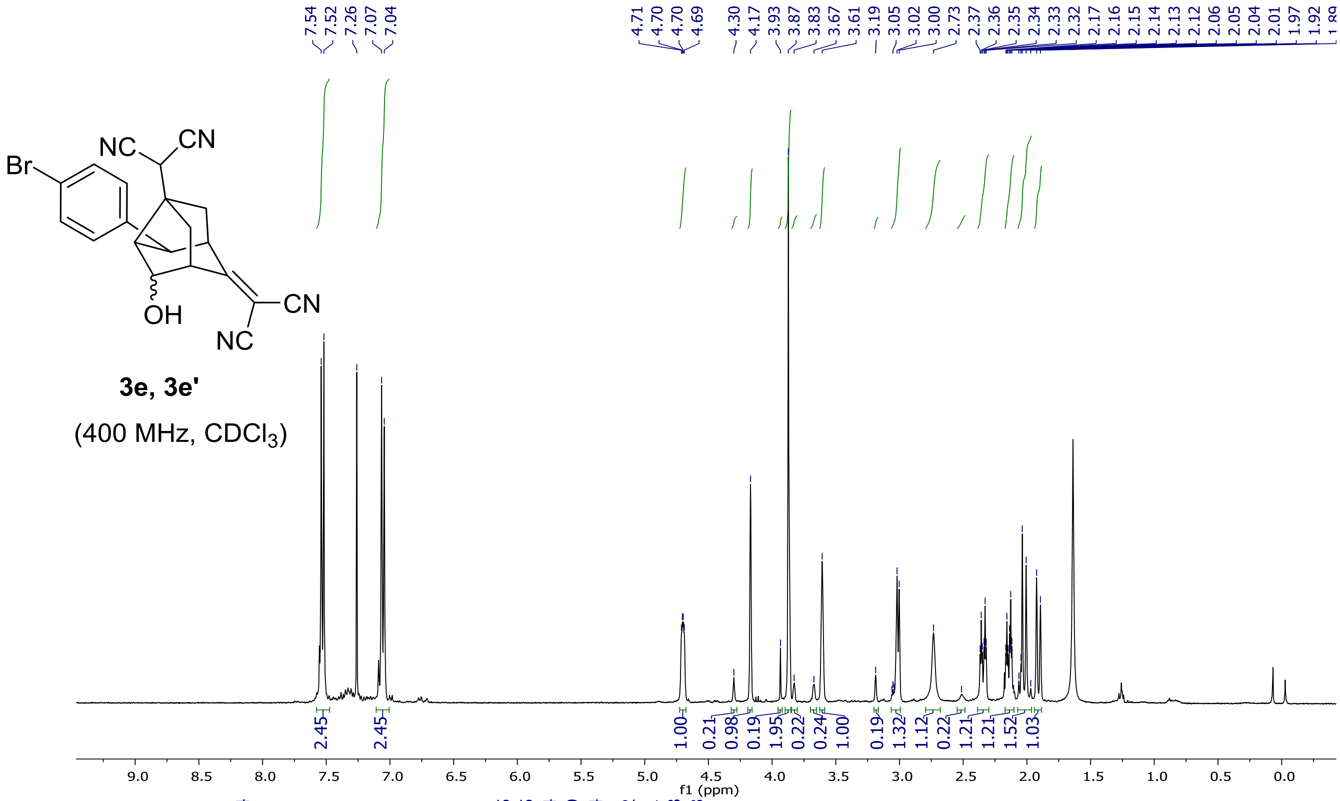
NC
CN
Br
OH
CN
NC
3e, 3e'
(400 MHz, CDCl3)
7.54
7.52
7.26
7.07
7.04
4.71
4.70
4.70
4.69
4.30
4.17
3.93
3.87
3.83
3.67
3.61
3.19
3.05
3.02
3.00
2.73
2.37
2.36
2.35
2.34
2.33
2.32
2.17
2.16
2.15
2.14
2.13
2.12
2.06
2.05
2.04
2.01
1.97
1.92
2.45
2.45
1.00
0.21
0.98
0.19
1.95
0.22
0.24
1.00
0.19
1.32
1.12
0.22
1.21
1.21
1.52
1.03
f1 (ppm)

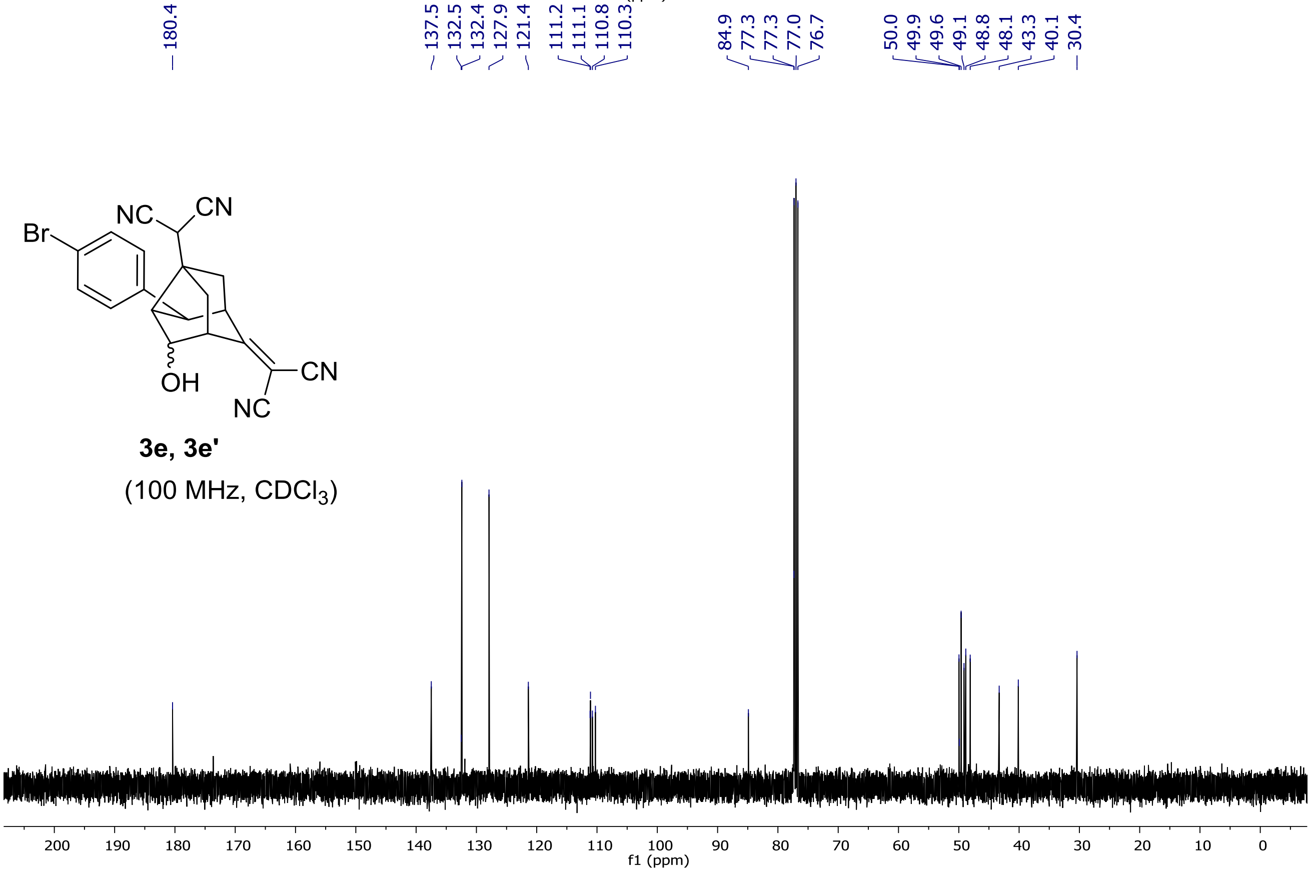
180.4
137.5
132.5
132.4
127.9
121.4
111.2
111.1
110.8
110.3
84.9
77.3
77.3
77.0
76.7
50.0
49.9
49.6
49.1
48.8
48.1
43.3
40.1
30.4
NC
CN
Br
OH
CN
NC
3e, 3e'
(100 MHz, CDCl3)
f1 (ppm)

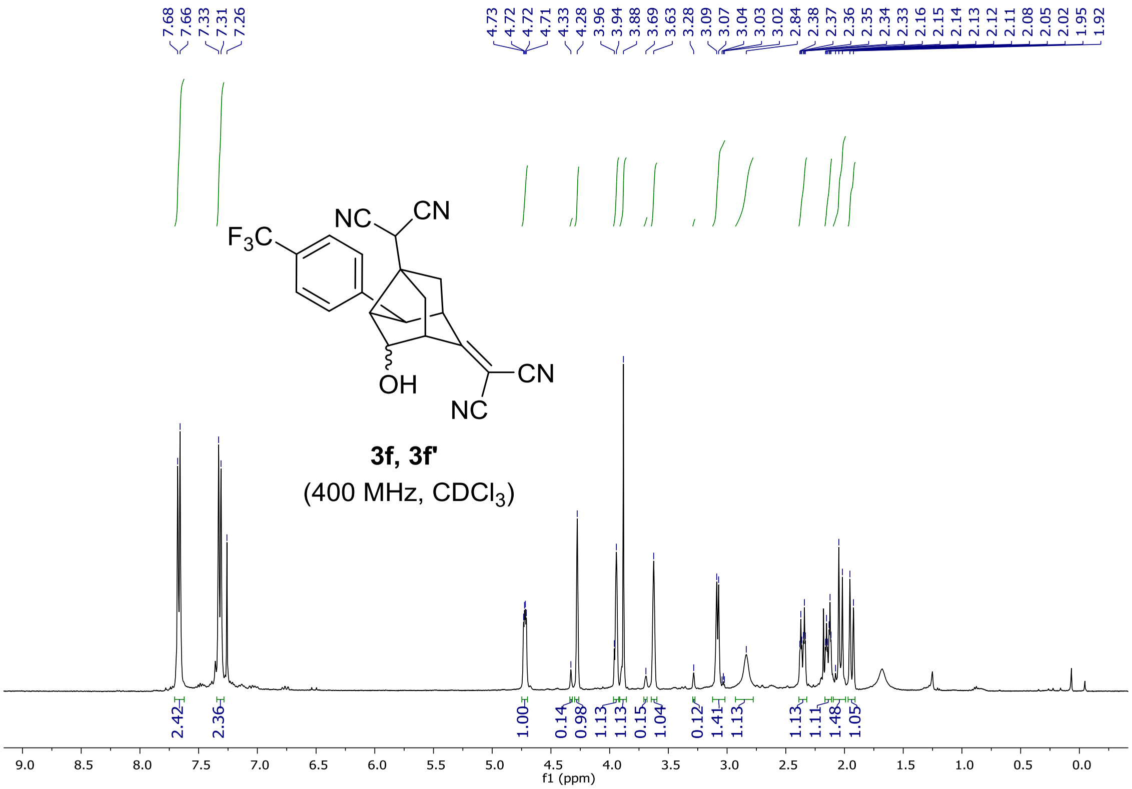

3f, 3f'

(400 MHz, $CDCl_3$)

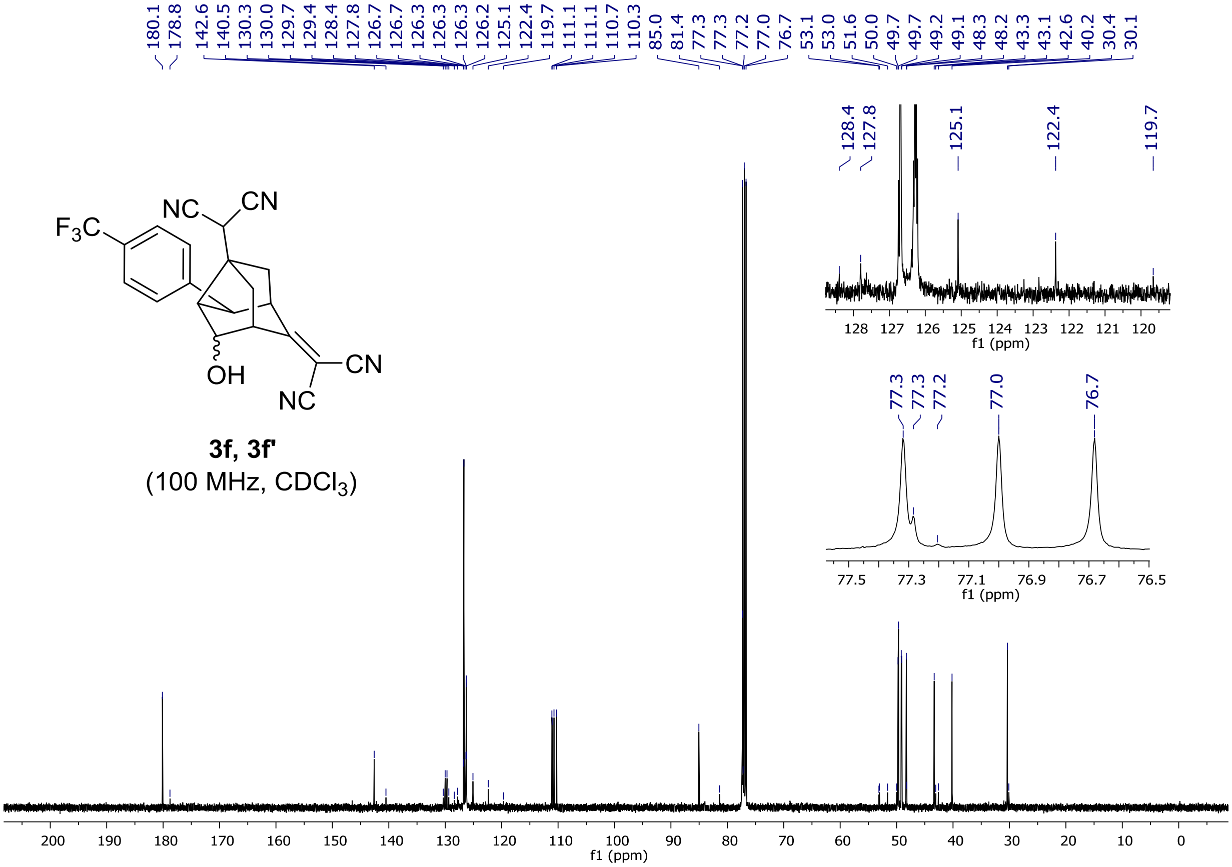

3f, 3f'

(100 MHz, $CDCl_3$)

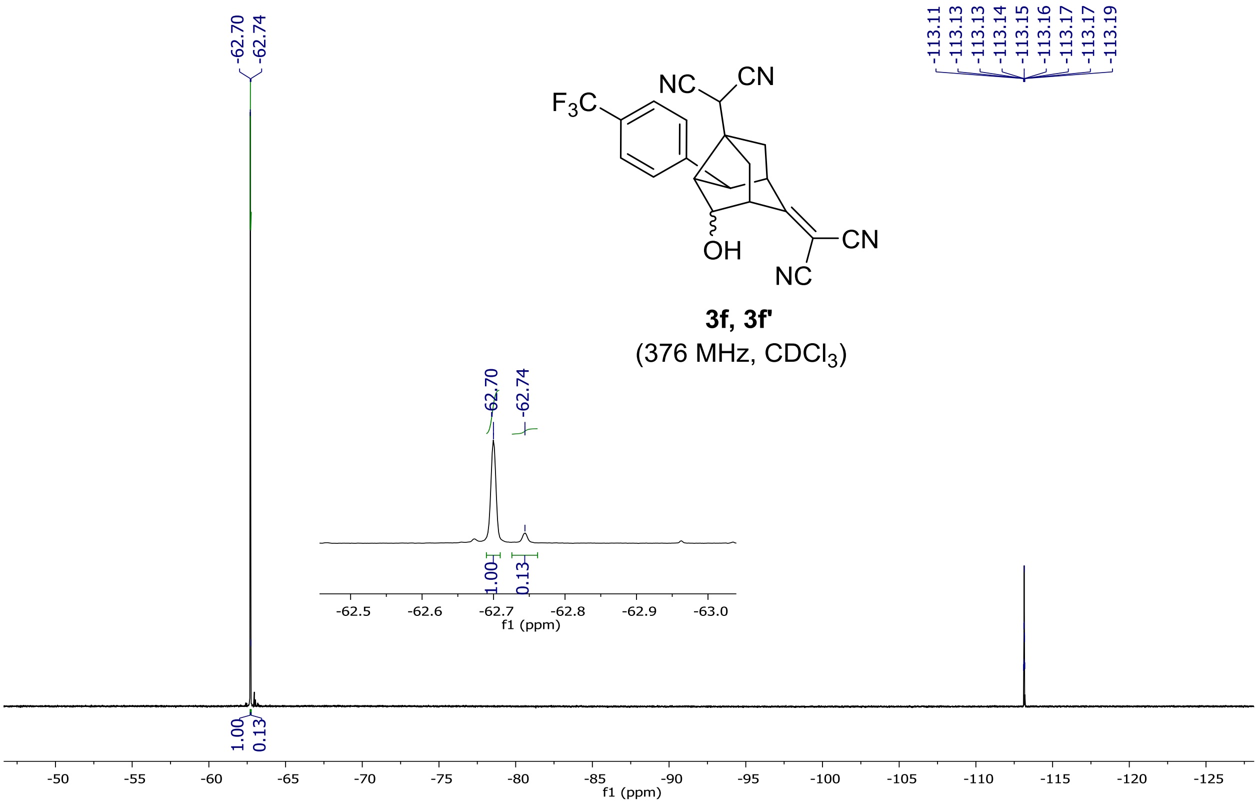
-62.70
-62.74
-113.11
-113.13
-113.13
-113.14
-113.15
-113.16
-113.17
-113.17
-113.19
F_3C
NC
CN
OH
NC
CN
3f, 3f'
(376 MHz, $CDCl_3$)
1.00
0.13
-62.5
-62.6
-62.7
-62.8
-62.9
-63.0
f1 (ppm)
-50
-55
-60
-65
-70
-75
-80
-85
-90
-95
-100
-105
-110
-115
-120
-125
f1 (ppm)

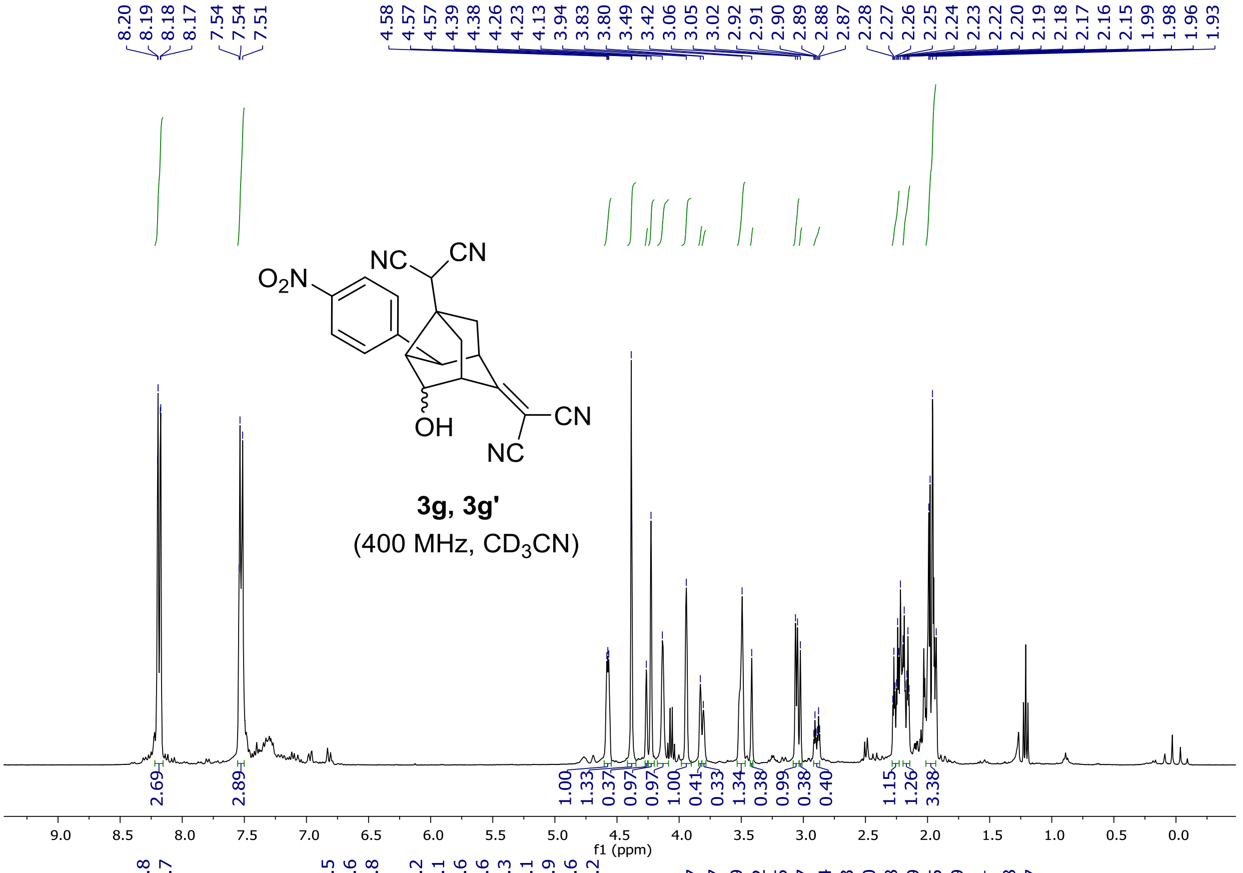
8.20 8.19 8.18 8.17 7.54 7.54 7.51
4.58 4.57 4.57 4.39 4.38 4.26 4.23 4.13 3.94 3.83 3.80 3.49 3.42 3.06 3.05 3.02 2.92 2.91 2.90 2.89 2.88 2.87 2.28 2.27 2.26 2.25 2.24 2.23 2.22 2.20 2.19 2.18 2.17 2.16 2.15 1.99 1.98 1.96 1.93
O_2N
NC
CN
OH
CN
NC
3g, 3g'
(400 MHz, CD_3CN)
2.69 2.89 1.00 1.33 0.37 0.97 0.97 1.00 0.41 0.33 1.34 0.38 0.99 0.38 0.40 1.15 1.26 3.38
f1 (ppm)

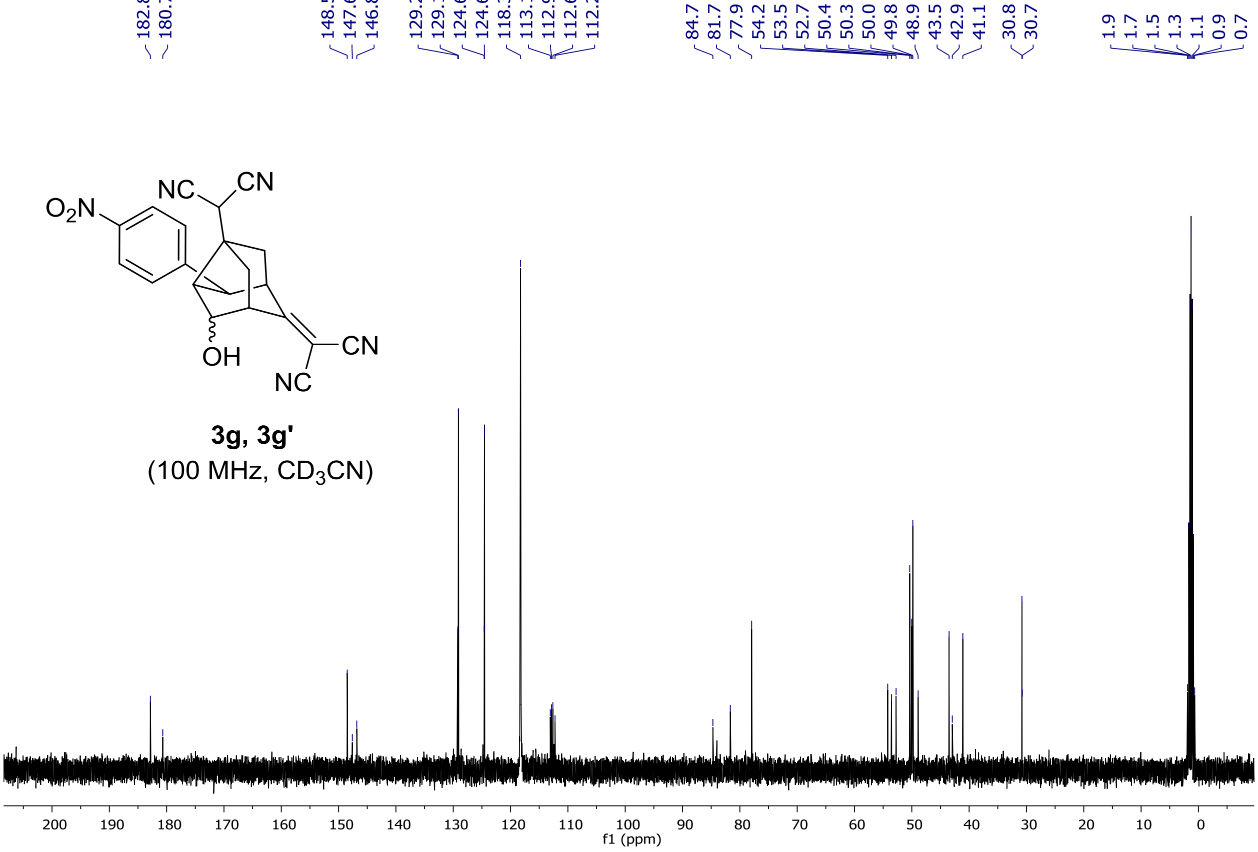
182.8 180.7 148.5 147.6 146.8 129.2 129.1 124.6 124.6 118.3 113.1 112.9 112.6 112.2 84.7 81.7 77.9 54.2 53.5 52.7 50.4 50.3 50.0 49.8 48.9 43.5 42.9 41.1 30.8 30.7 1.9 1.7 1.5 1.3 1.1 0.9 0.7
O_2N
NC
CN
OH
CN
NC
3g, 3g'
(100 MHz, CD_3CN)
f1 (ppm)

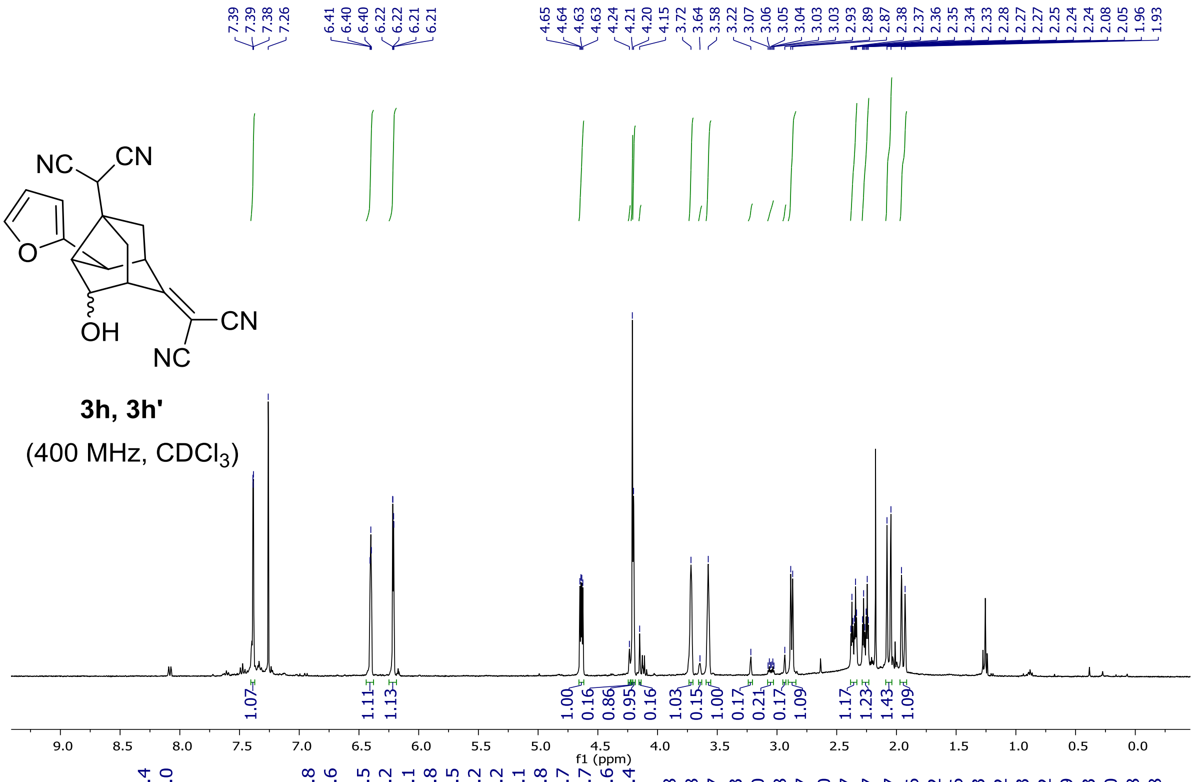
7.39 7.39 7.38 7.26 6.41 6.40 6.40 6.22 6.22 6.21 6.21 4.65 4.64 4.63 4.63 4.24 4.21 4.20 4.15 3.72 3.64 3.58 3.22 3.07 3.06 3.05 3.04 3.03 3.03 2.93 2.89 2.87 2.38 2.37 2.36 2.35 2.34 2.33 2.28 2.27 2.27 2.25 2.24 2.24 2.08 2.05 1.96 1.93
NC
CN
O
OH
CN
NC
3h, 3h'
(400 MHz, CDCl3)
1.07 1.11 1.13 1.00 0.16 0.86 0.95 0.16 1.03 0.15 1.00 0.17 0.21 0.17 1.09 1.17 1.23 1.43 1.09
9.0 8.5 8.0 7.5 7.0 6.5 6.0 5.5 5.0 4.5 4.0 3.5 3.0 2.5 2.0 1.5 1.0 0.5 0.0
f1 (ppm)

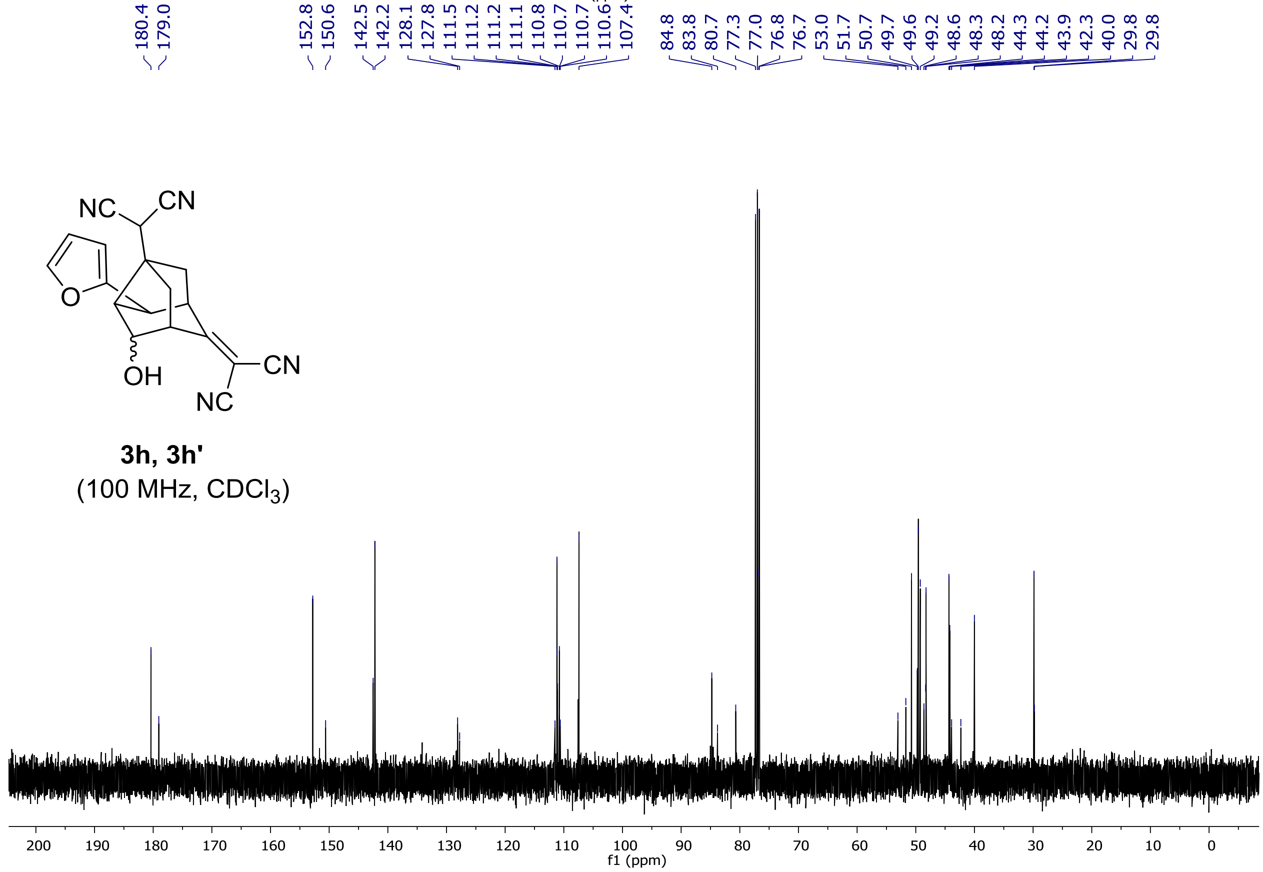
180.4 179.0 152.8 150.6 142.5 142.2 128.1 127.8 111.5 111.2 111.2 111.1 110.8 110.7 110.7 110.6 107.4 84.8 83.8 80.7 77.3 77.0 76.8 76.7 53.0 51.7 50.7 49.7 49.6 49.2 48.6 48.3 48.2 44.3 44.2 43.9 42.3 40.0 29.8 29.8
NC
CN
O
OH
CN
NC
3h, 3h'
(100 MHz, CDCl3)
200 190 180 170 160 150 140 130 120 110 100 90 80 70 60 50 40 30 20 10 0
f1 (ppm)

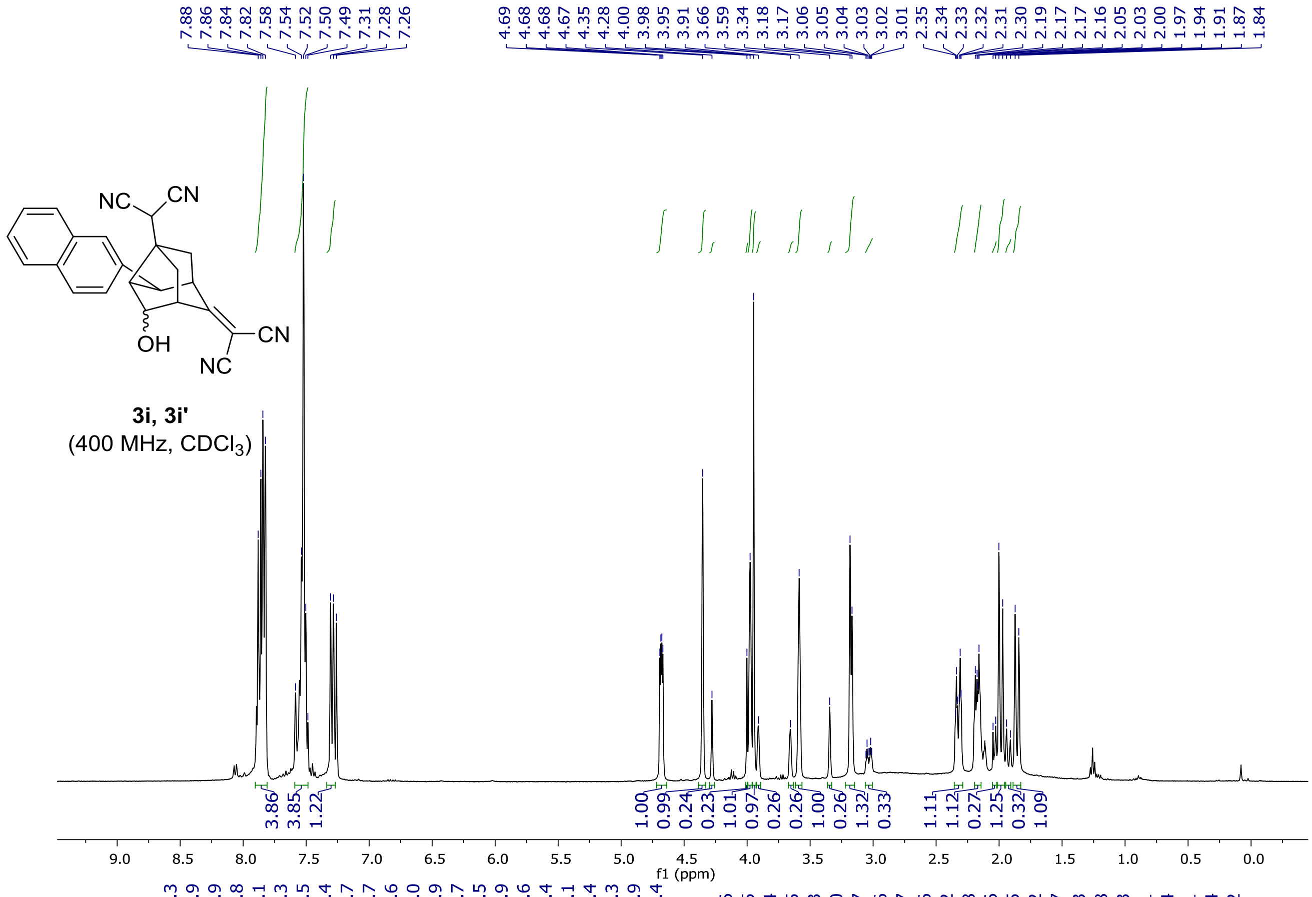

3i, 3i'
(400 MHz, $CDCl_3$)
7.88 7.86 7.84 7.82 7.58 7.54 7.52 7.50 7.49 7.31 7.28 7.26
4.69 4.68 4.68 4.67 4.35 4.28 4.00 3.98 3.95 3.91 3.66 3.59 3.34 3.18 3.17 3.06 3.05 3.04 3.03 3.02 3.01
2.35 2.34 2.33 2.32 2.31 2.30 2.19 2.17 2.17 2.16 2.05 2.03 2.00 1.97 1.94 1.91 1.87 1.84
3.86 3.85 1.22
1.00 0.99 0.24 0.23 1.01 0.97 0.26 0.26 1.00 0.26 1.32 0.33
1.11 1.12 0.27 1.25 0.32 1.09
f1 (ppm)

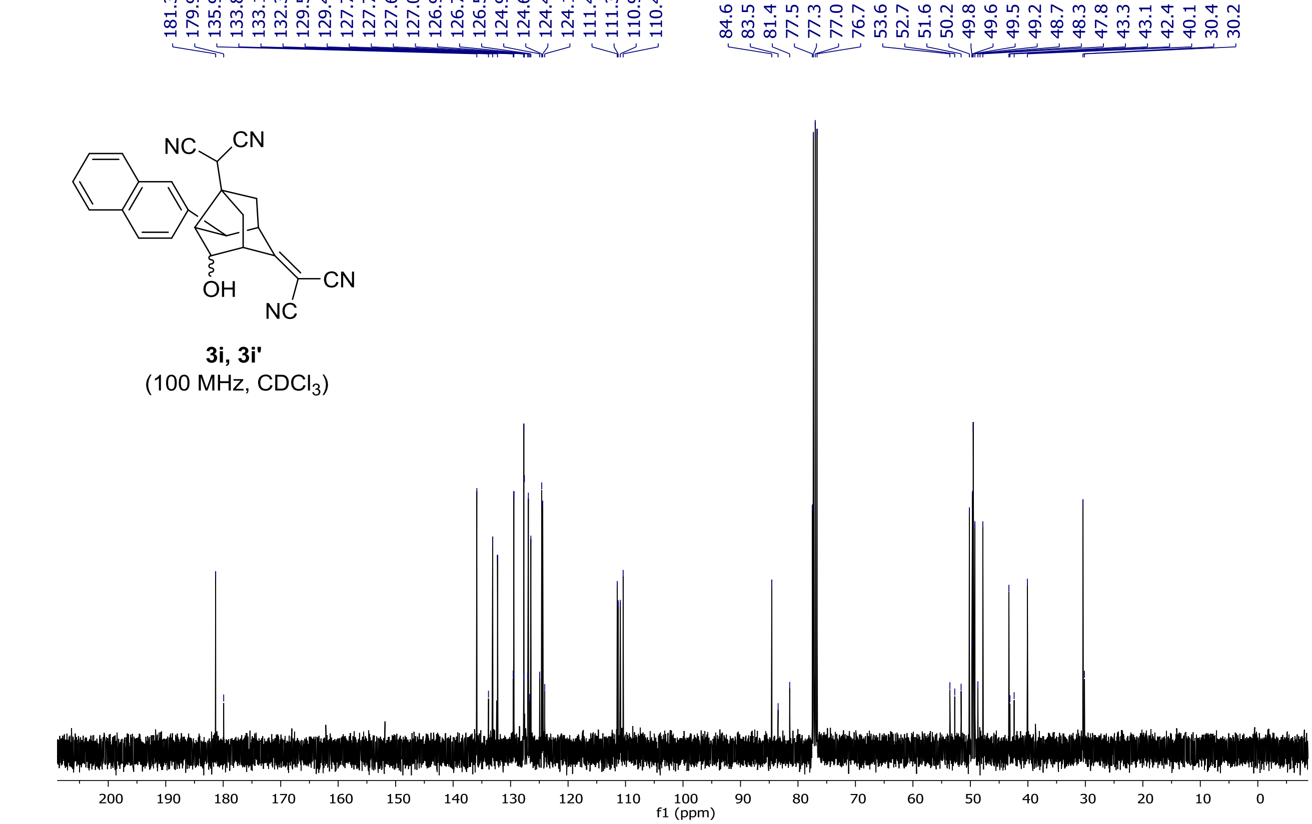

3i, 3i'
(100 MHz, $CDCl_3$)
181.3 179.9 135.9 133.8 133.1 132.3 129.5 129.4 127.7 127.6 127.0 126.9 126.7 126.5 124.9 124.6 124.4 124.1 111.4 111.3 110.9 110.4
84.6 83.5 81.4 77.5 77.3 77.0 76.7 53.6 52.7 51.6 50.2 49.8 49.6 49.5 49.2 48.7 48.3 47.8 43.3 43.1 42.4 40.1 30.4 30.2
f1 (ppm)

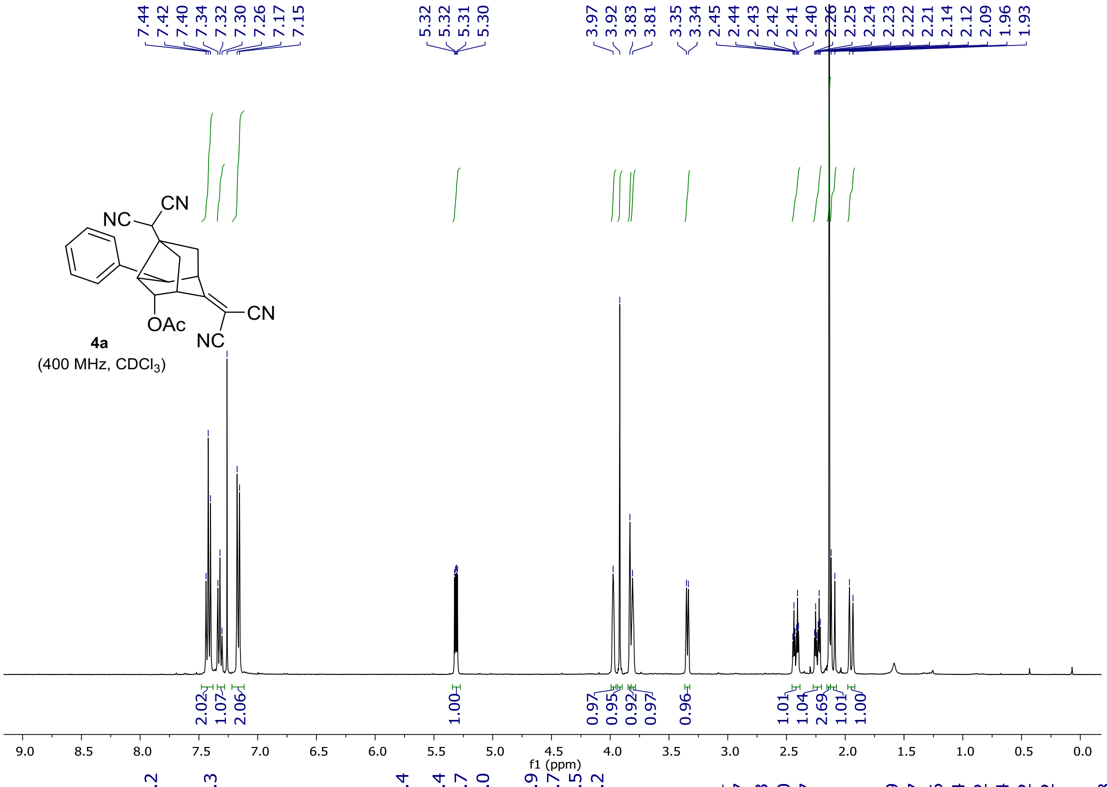
7.44 7.42 7.40 7.34 7.32 7.30 7.26 7.17 7.15
5.32 5.32 5.31 5.30
3.97 3.92 3.83 3.81 3.35 3.34 2.45 2.44 2.43 2.42 2.41 2.40 2.26 2.25 2.24 2.23 2.22 2.21 2.14 2.12 2.09 1.96 1.93
NC
CN
OAc
CN
NC
4a
(400 MHz, CDCl$_3$)
2.02 1.07 2.06
1.00
0.97 0.95 0.92 0.97
0.96
1.01 1.04 2.69 1.01 1.00
9.0 8.5 8.0 7.5 7.0 6.5 6.0 5.5 5.0 4.5 4.0 3.5 3.0 2.5 2.0 1.5 1.0 0.5 0.0
f1 (ppm)

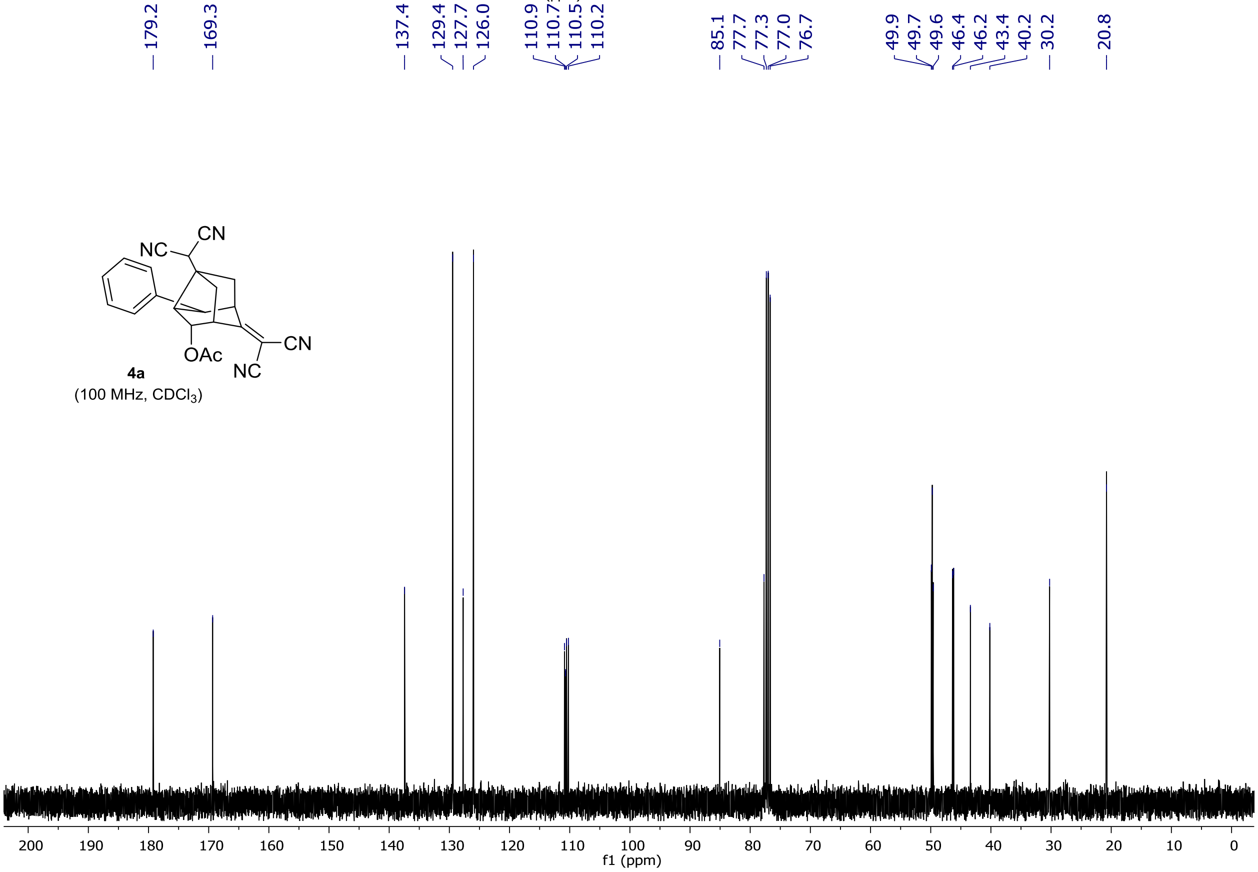
179.2
169.3
137.4
129.4 127.7 126.0
110.9 110.7 110.5 110.2
85.1
77.7 77.3 77.0 76.7
49.9 49.7 49.6 46.4 46.2 43.4 40.2
30.2
20.8
NC
CN
OAc
CN
NC
4a
(100 MHz, CDCl$_3$)
200 190 180 170 160 150 140 130 120 110 100 90 80 70 60 50 40 30 20 10 0
f1 (ppm)

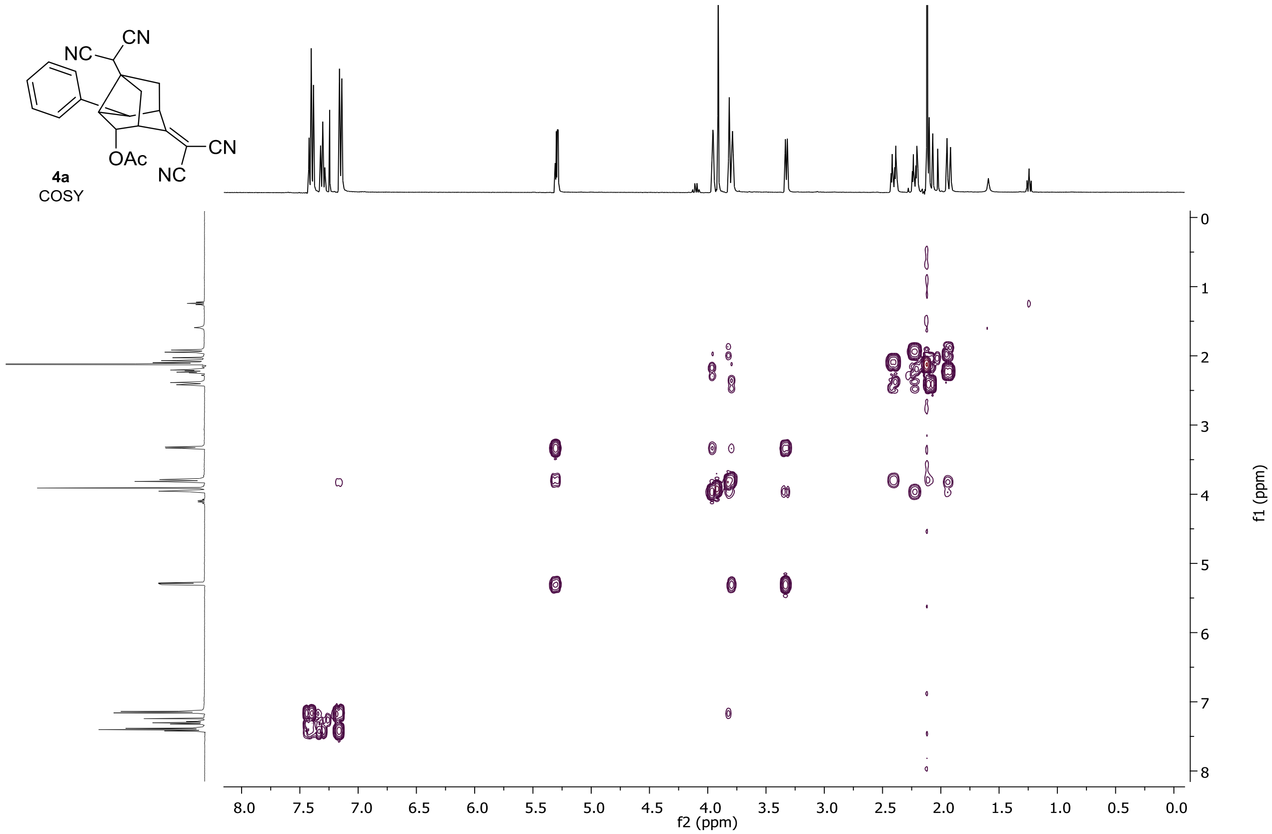
NC
CN
OAc
CN
NC
4a
COSY
f1 (ppm)
f2 (ppm)

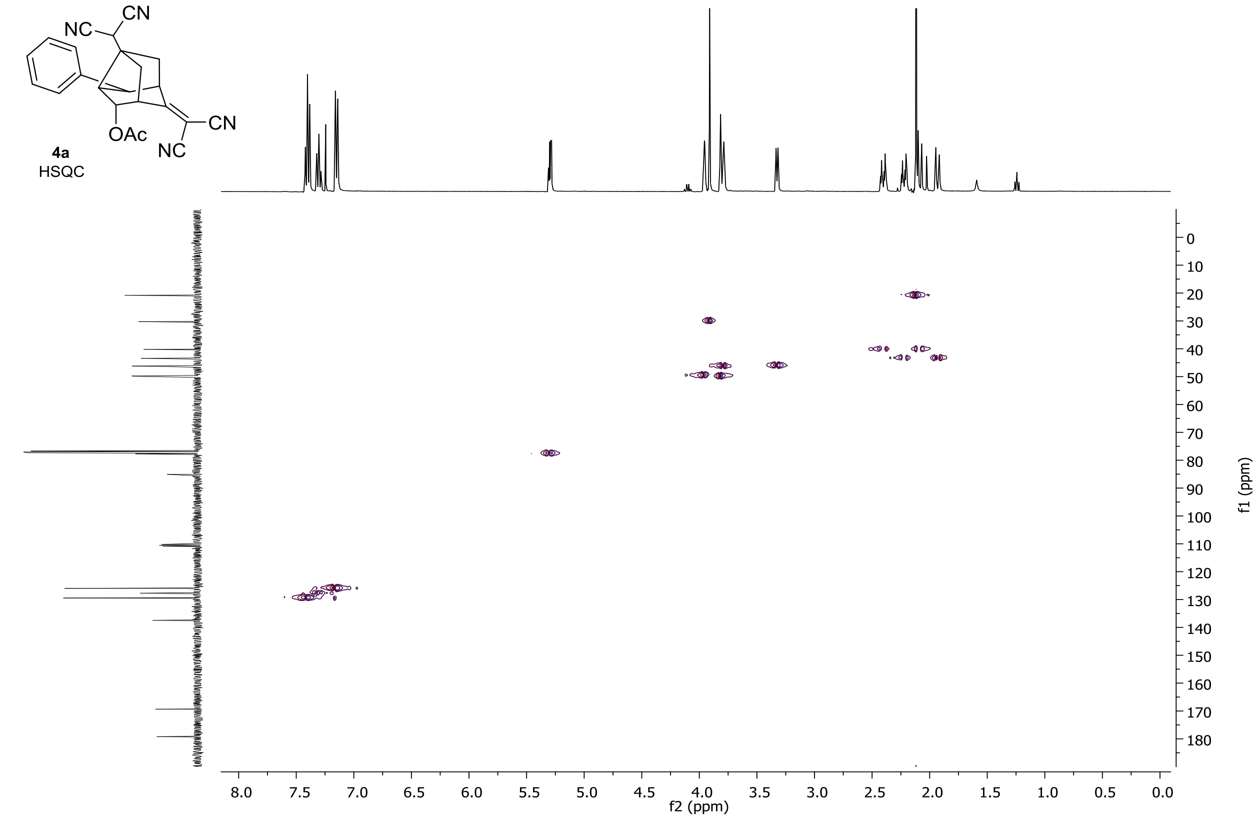
NC
CN
OAc
CN
NC
4a
HSQC
f1 (ppm)
f2 (ppm)

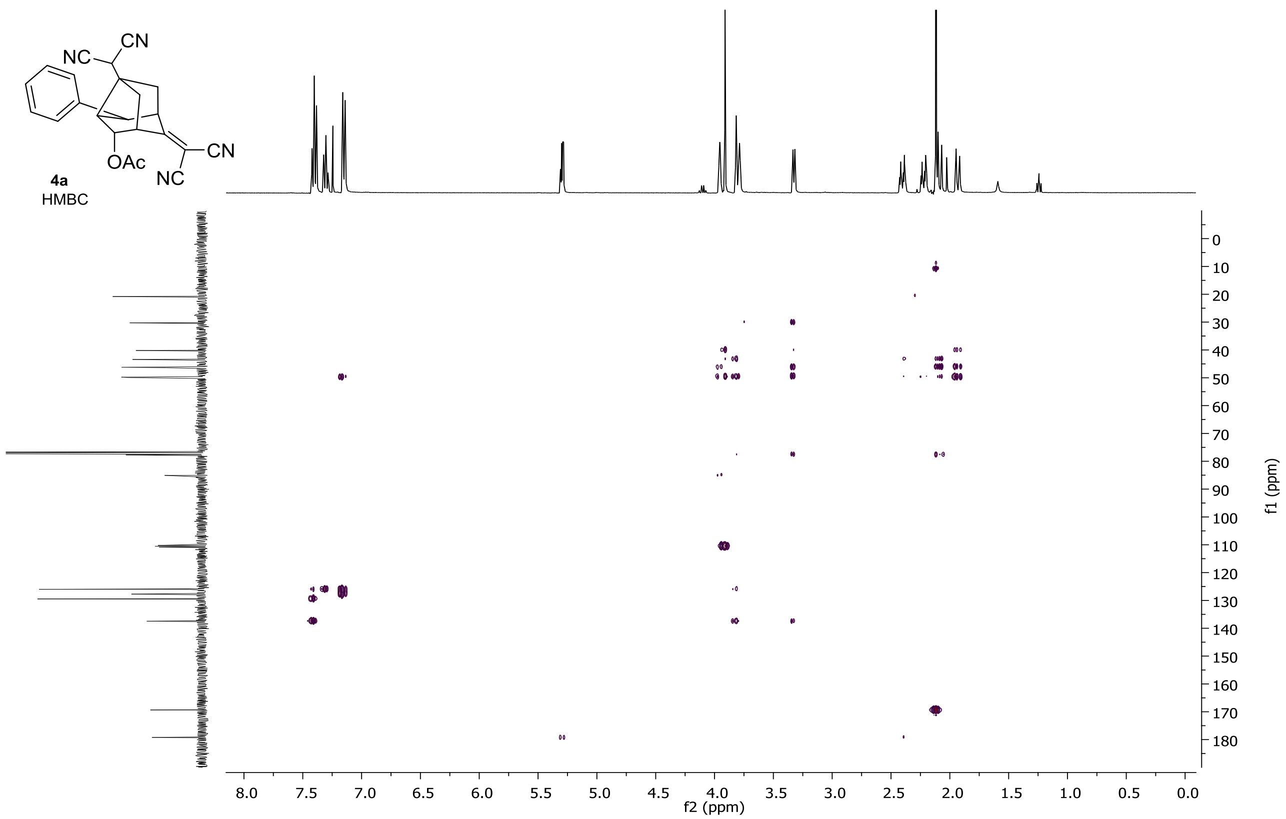

NC
CN
OAc
CN
NC
4a
HMBC
f2 (ppm)
f1 (ppm)

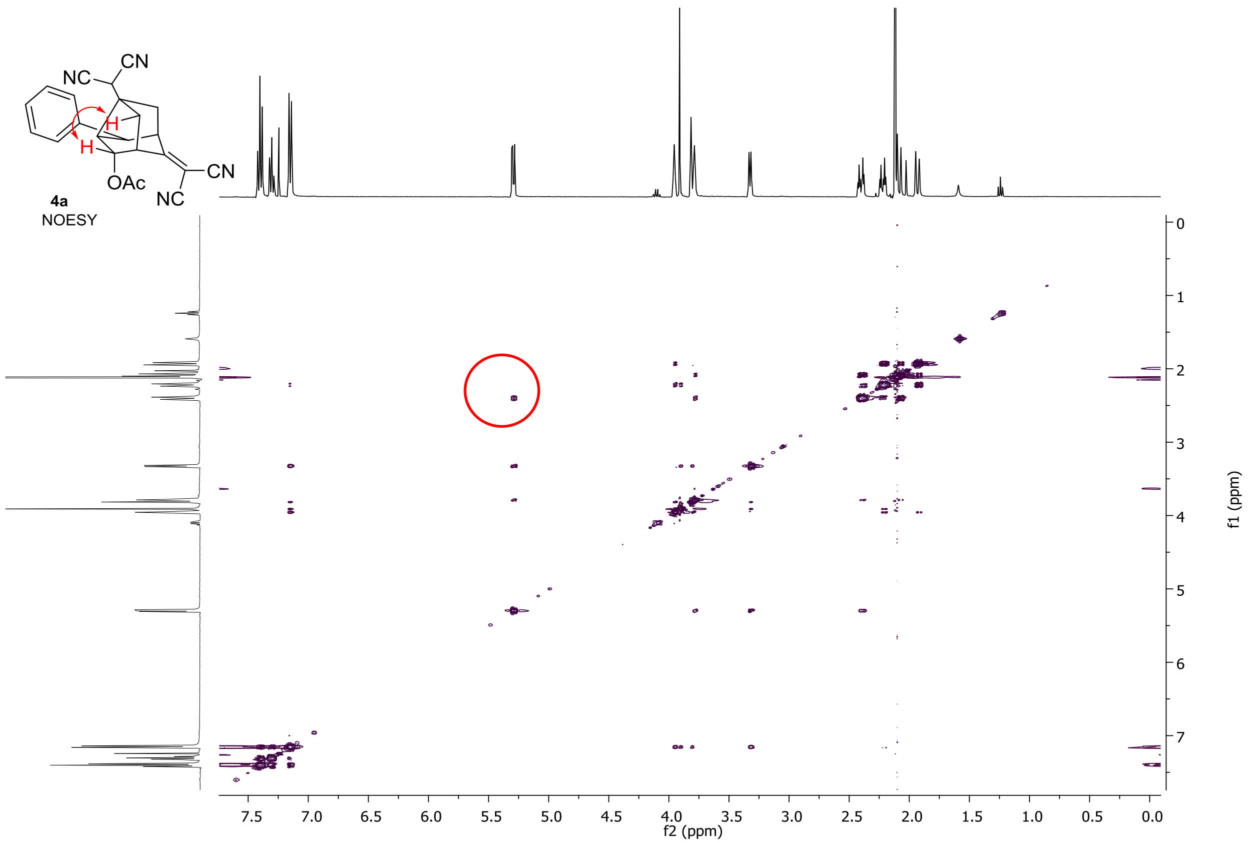

NC
CN
H
H
OAc
CN
NC
4a
NOESY
f2 (ppm)
f1 (ppm)

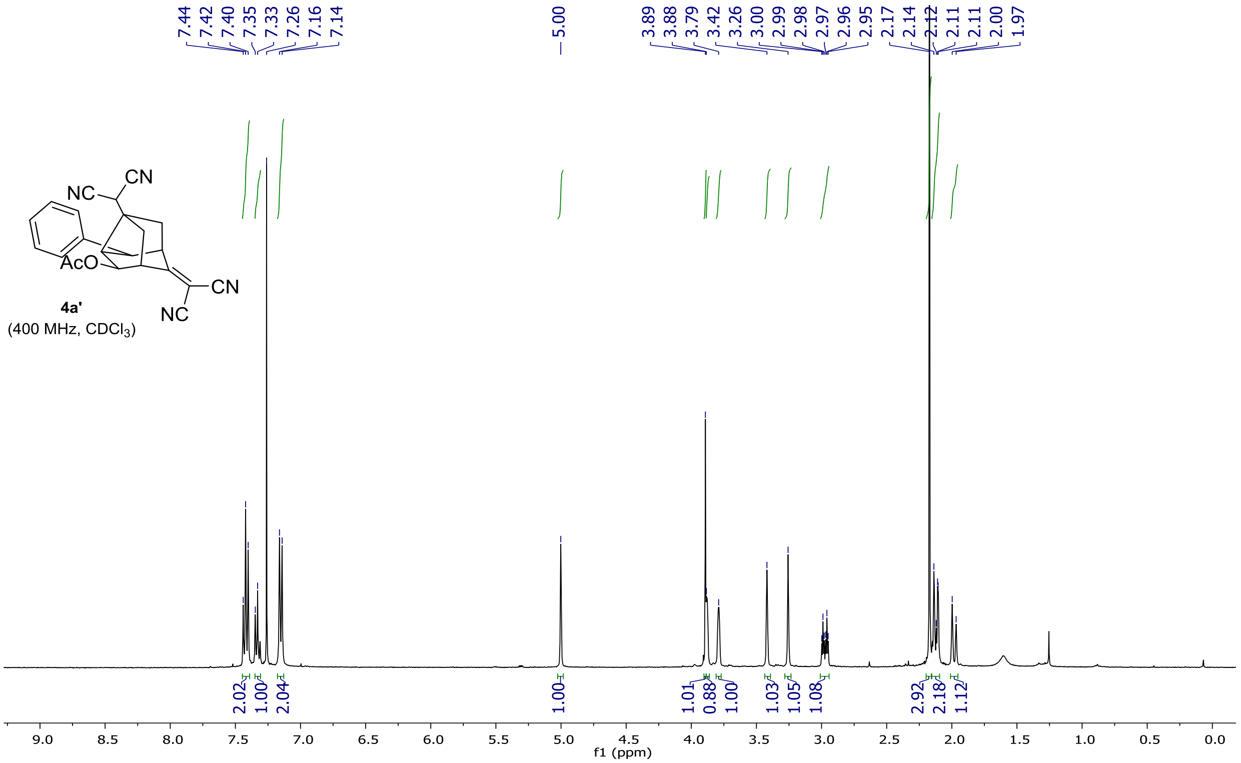
7.44
7.42
7.40
7.35
7.33
7.26
7.16
7.14
5.00
3.89
3.88
3.79
3.42
3.26
3.00
2.99
2.98
2.97
2.96
2.95
2.17
2.14
2.12
2.11
2.11
2.00
1.97
NC
CN
AcO
CN
NC
4a'
(400 MHz, CDCl3)
2.02
1.00
2.04
1.00
1.01
0.88
1.00
1.03
1.05
1.08
2.92
2.18
1.12
9.0
8.5
8.0
7.5
7.0
6.5
6.0
5.5
5.0
4.5
4.0
3.5
3.0
2.5
2.0
1.5
1.0
0.5
0.0
f1 (ppm)

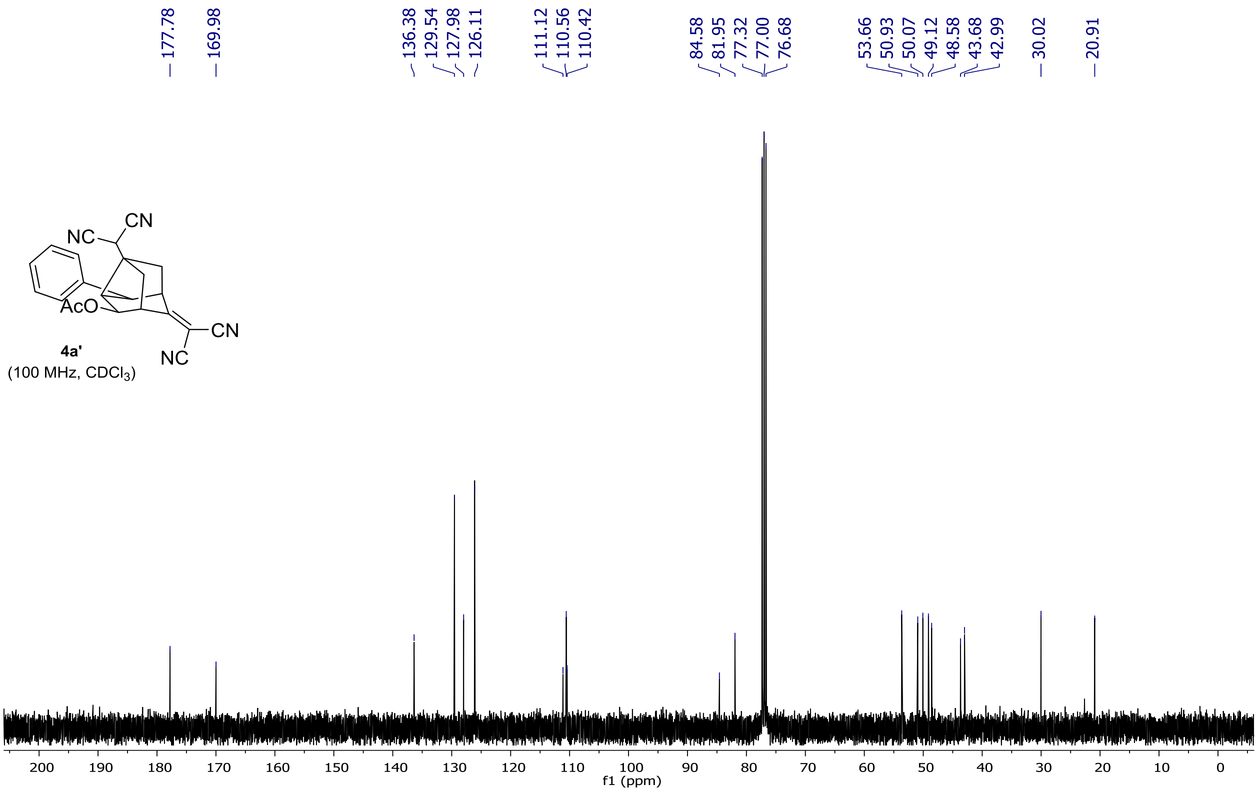
177.78
169.98
136.38
129.54
127.98
126.11
111.12
110.56
110.42
84.58
81.95
77.32
77.00
76.68
53.66
50.93
50.07
49.12
48.58
43.68
42.99
30.02
20.91
NC
CN
AcO
CN
NC
4a'
(100 MHz, CDCl3)
200
190
180
170
160
150
140
130
120
110
100
90
80
70
60
50
40
30
20
10
0
f1 (ppm)

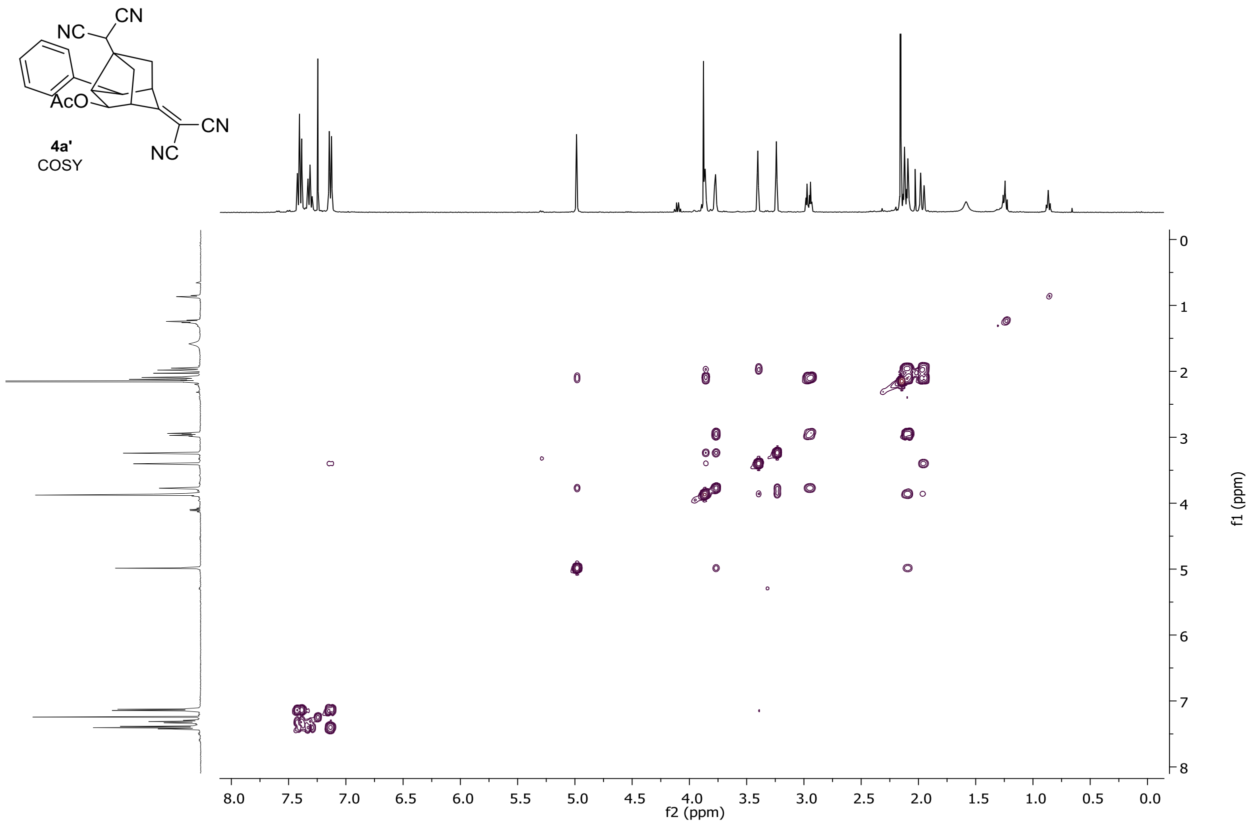
4a'
COSY
f1 (ppm)
f2 (ppm)

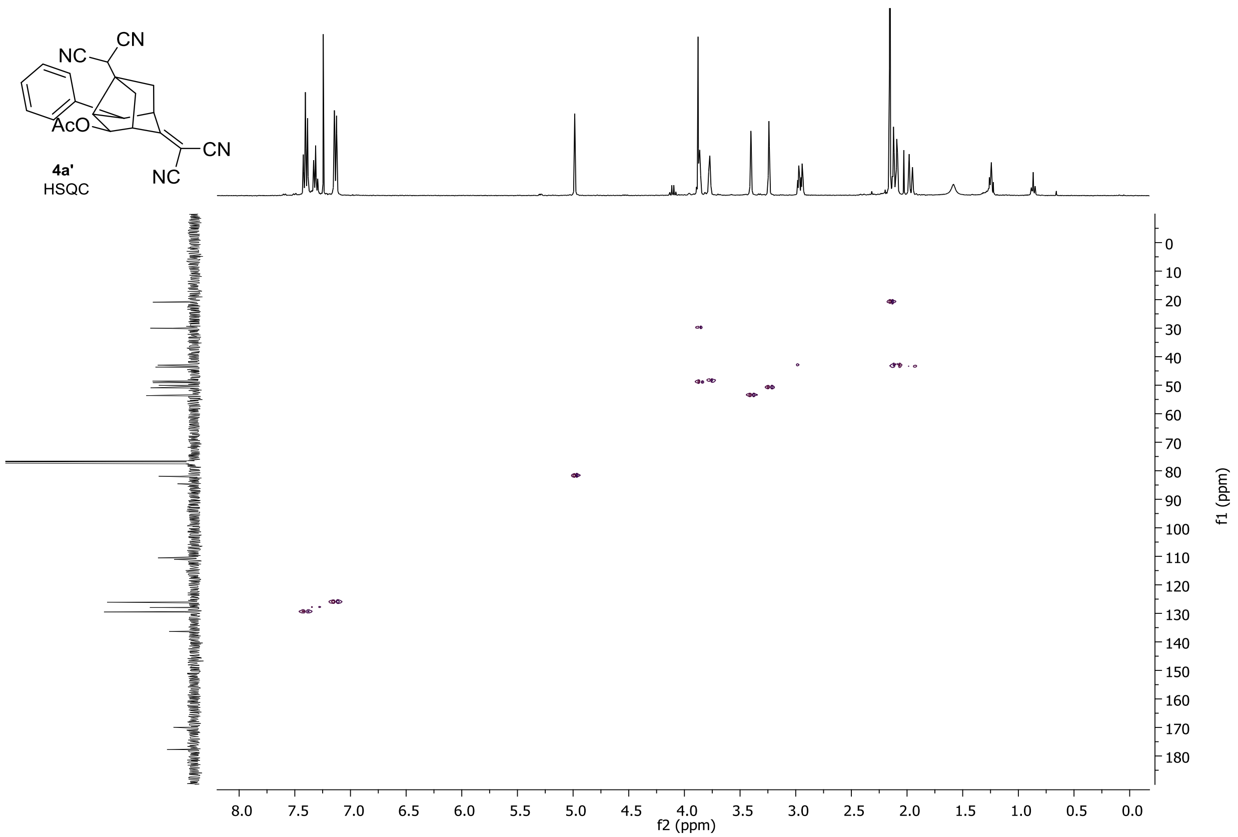
4a'
HSQC
f1 (ppm)
f2 (ppm)

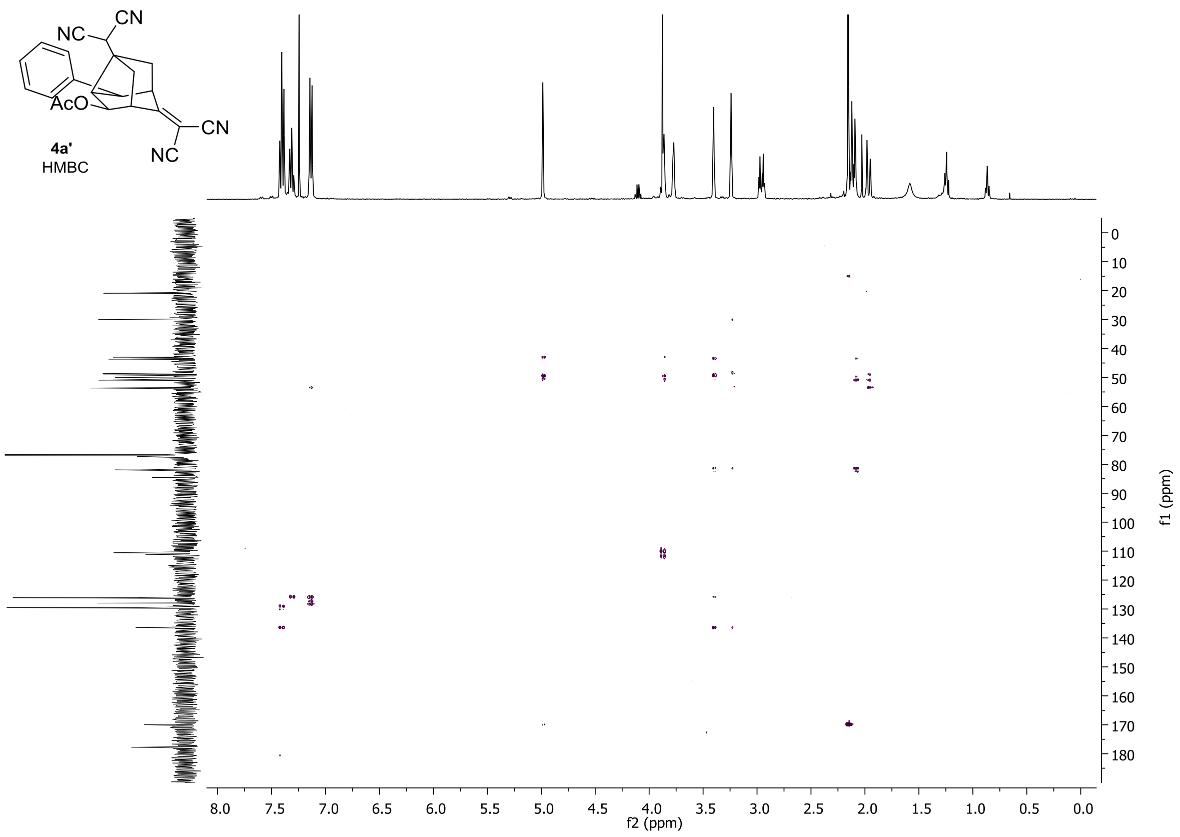

NC
CN
AcO
CN
NC
4a'
HMBC
f2 (ppm)
f1 (ppm)

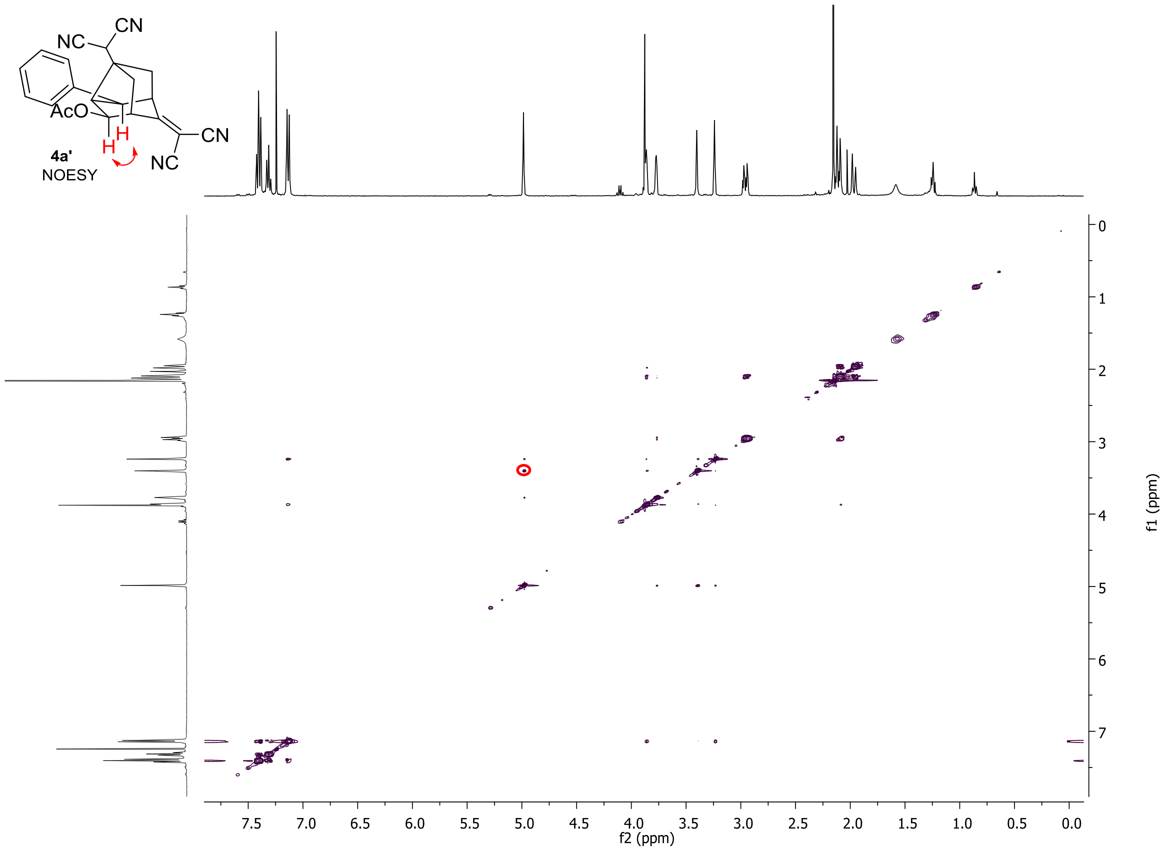

NC
CN
AcO
H
H
CN
NC
4a'
NOESY
f2 (ppm)
f1 (ppm)

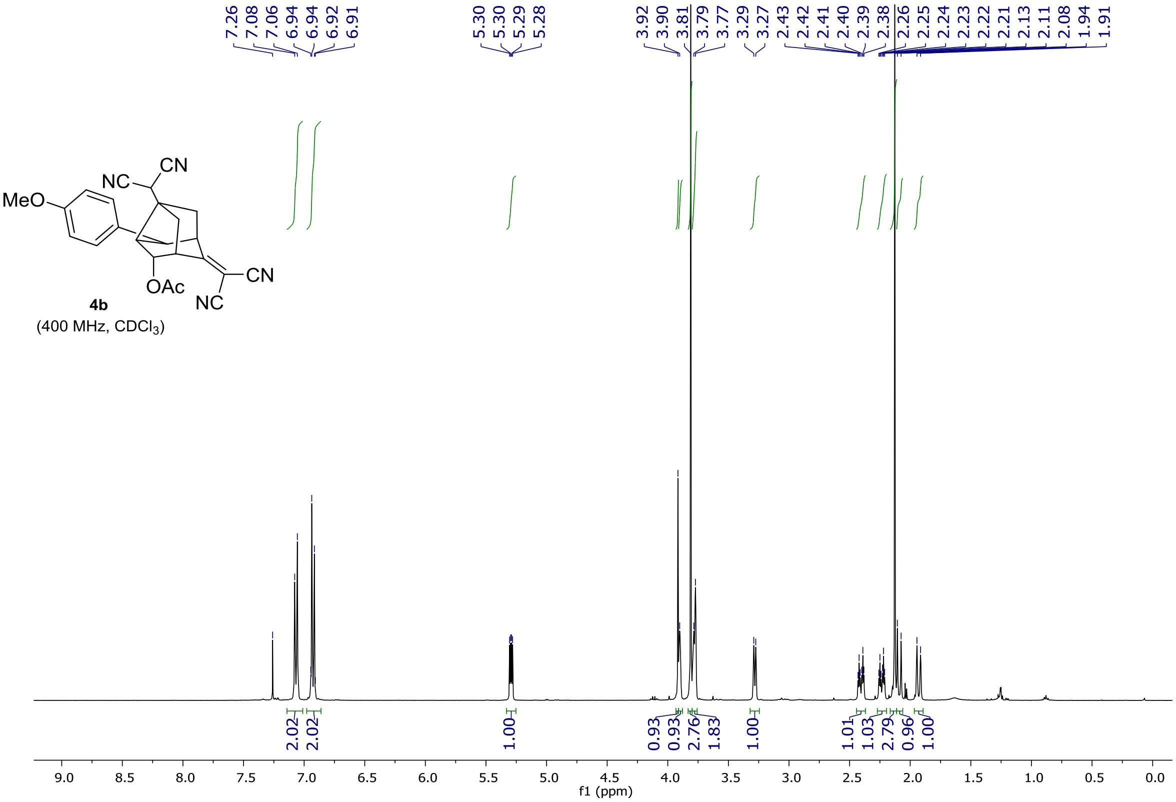

4b
(400 MHz, $CDCl_3$)

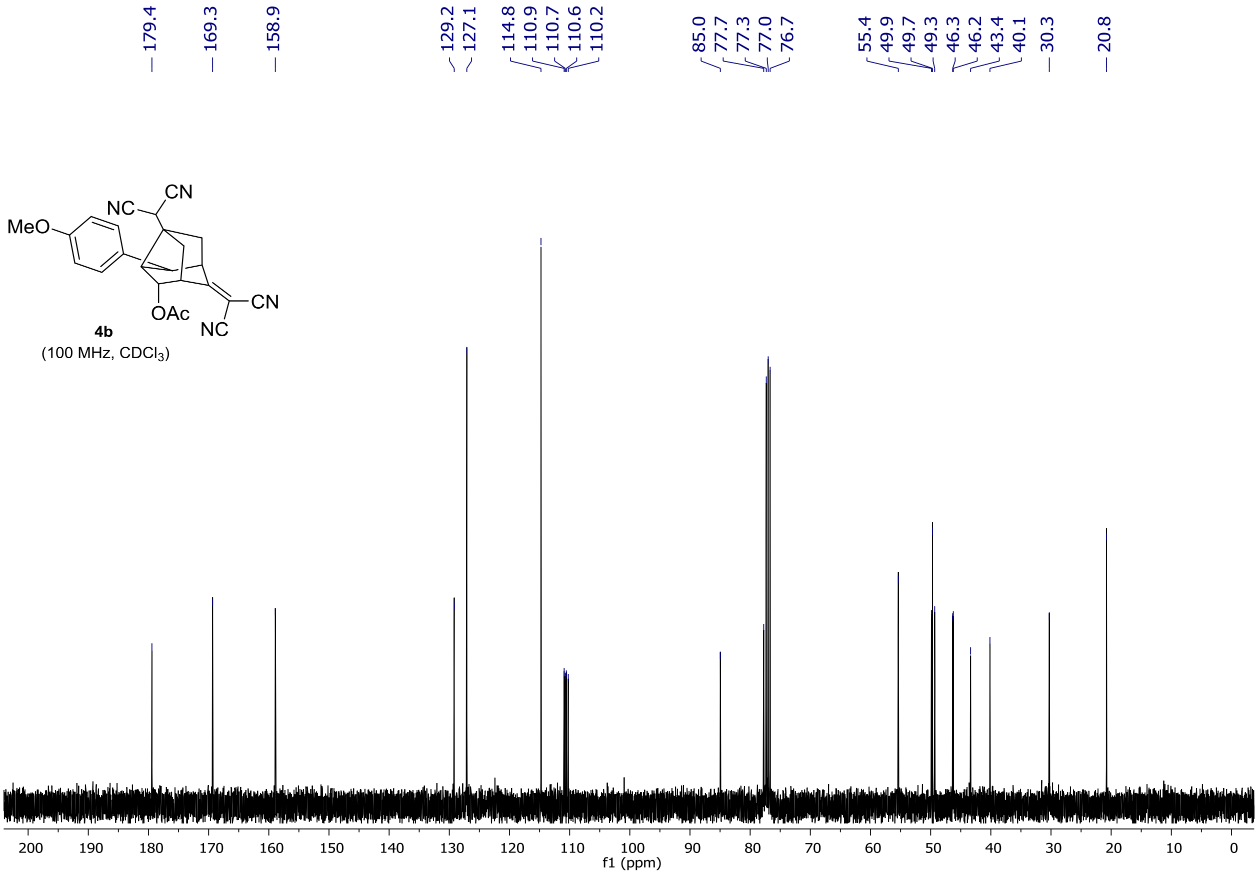

4b
(100 MHz, $CDCl_3$)

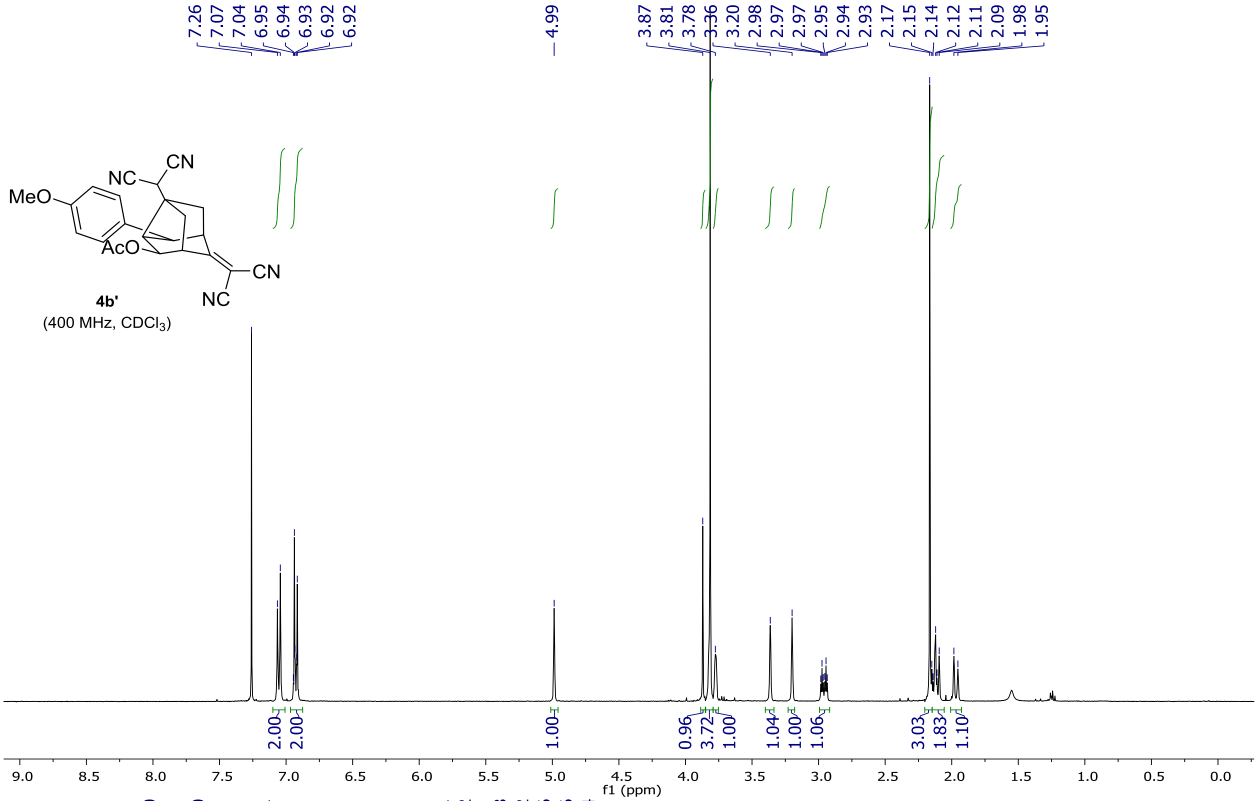
4b'
(400 MHz, CDCl3)
7.26 7.07 7.04 6.95 6.94 6.93 6.92 6.92
4.99
3.87 3.81 3.78 3.36 3.20 2.98 2.97 2.97 2.95 2.94 2.93 2.17 2.15 2.14 2.12 2.11 2.09 1.98 1.95
2.00 2.00 1.00 0.96 3.72 1.00 1.04 1.00 1.06 3.03 1.83 1.10
f1 (ppm)

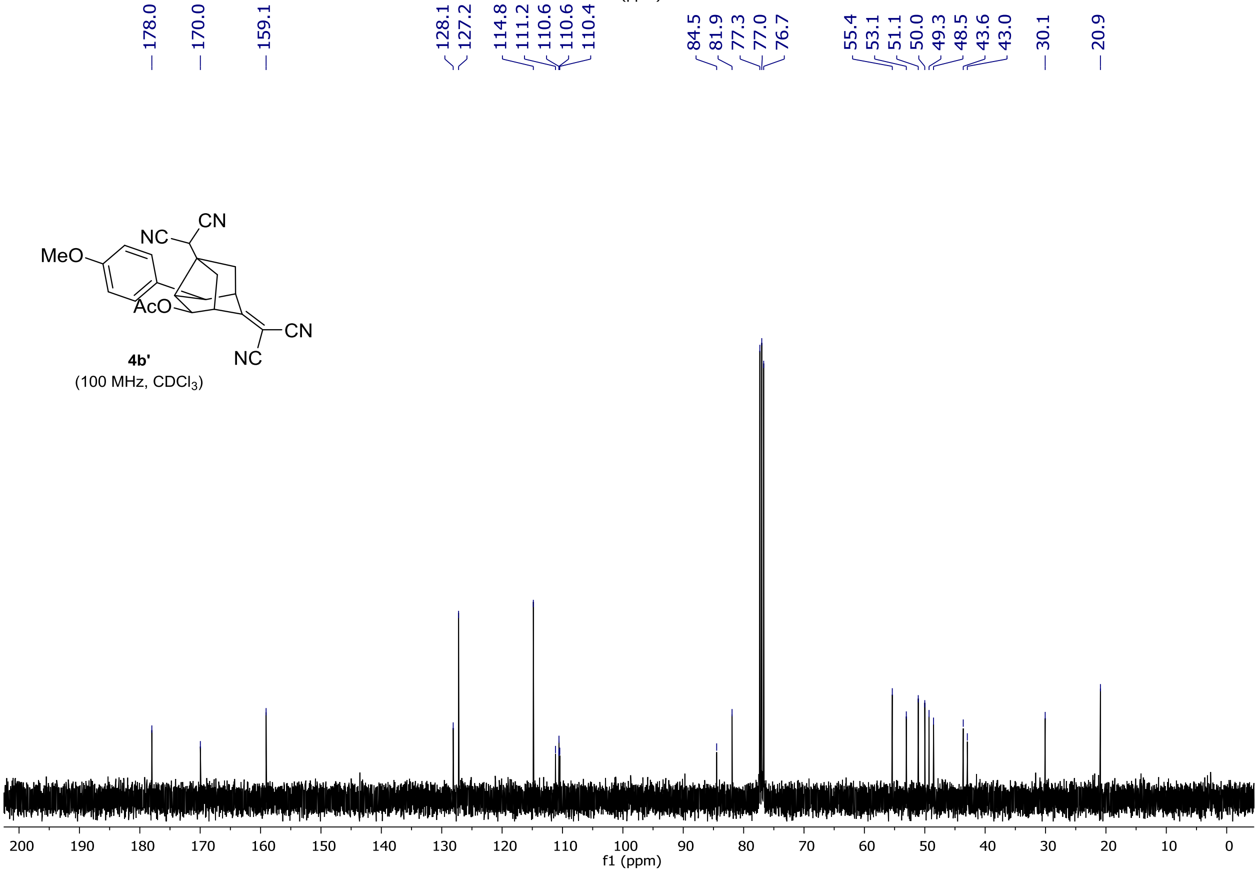
4b'
(100 MHz, CDCl3)
178.0 170.0 159.1 128.1 127.2 114.8 111.2 110.6 110.6 110.4 84.5 81.9 77.3 77.0 76.7 55.4 53.1 51.1 50.0 49.3 48.5 43.6 43.0 30.1 20.9
f1 (ppm)

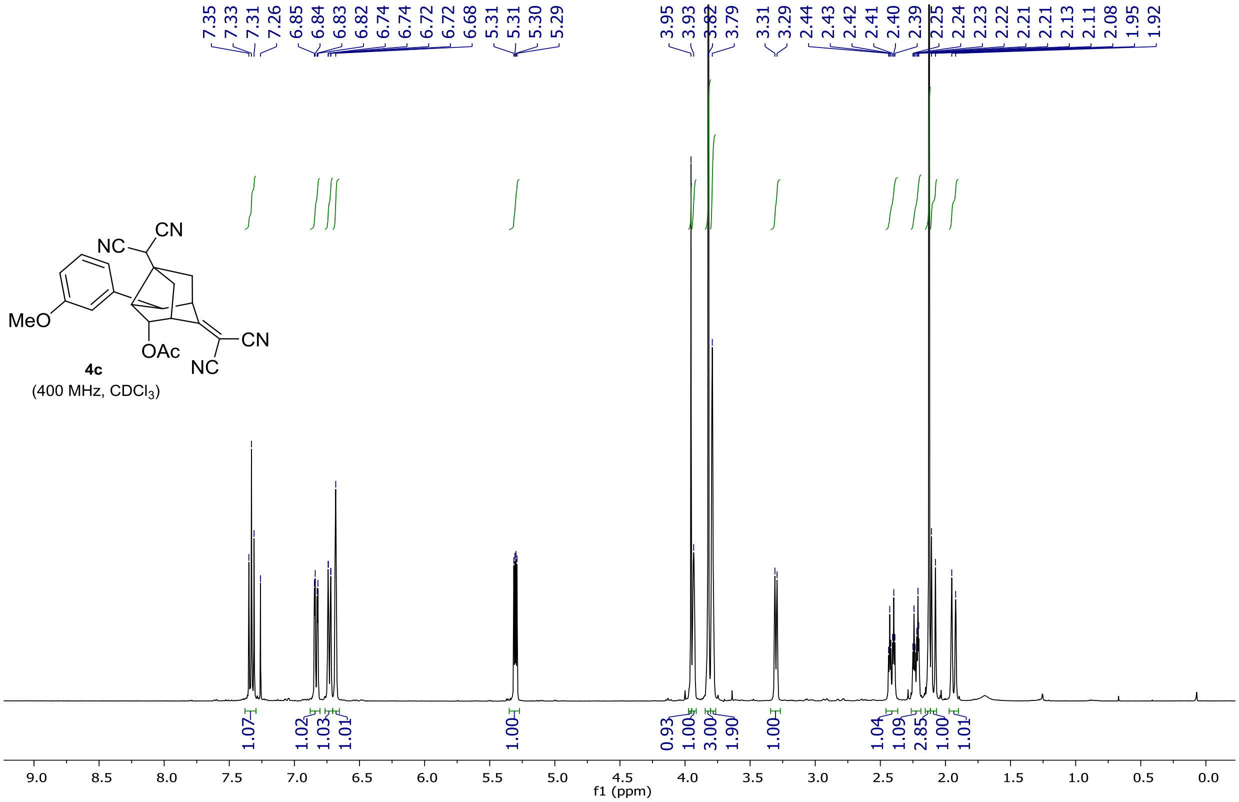

7.35 7.33 7.31 7.26 6.85 6.84 6.83 6.82 6.74 6.74 6.72 6.72 6.68 5.31 5.31 5.30 5.29
3.95 3.93 3.82 3.79 3.31 3.29 2.44 2.43 2.42 2.41 2.40 2.39 2.25 2.24 2.23 2.22 2.21 2.21 2.13 2.11 2.08 1.95 1.92
NC
CN
MeO
OAc
CN
NC
4c
(400 MHz, $CDCl_3$)
1.07 1.02 1.03 1.01 1.00 0.93 1.00 3.00 1.90 1.00 1.04 1.09 2.85 1.00 1.01
9.0 8.5 8.0 7.5 7.0 6.5 6.0 5.5 5.0 4.5 4.0 3.5 3.0 2.5 2.0 1.5 1.0 0.5 0.0
f1 (ppm)

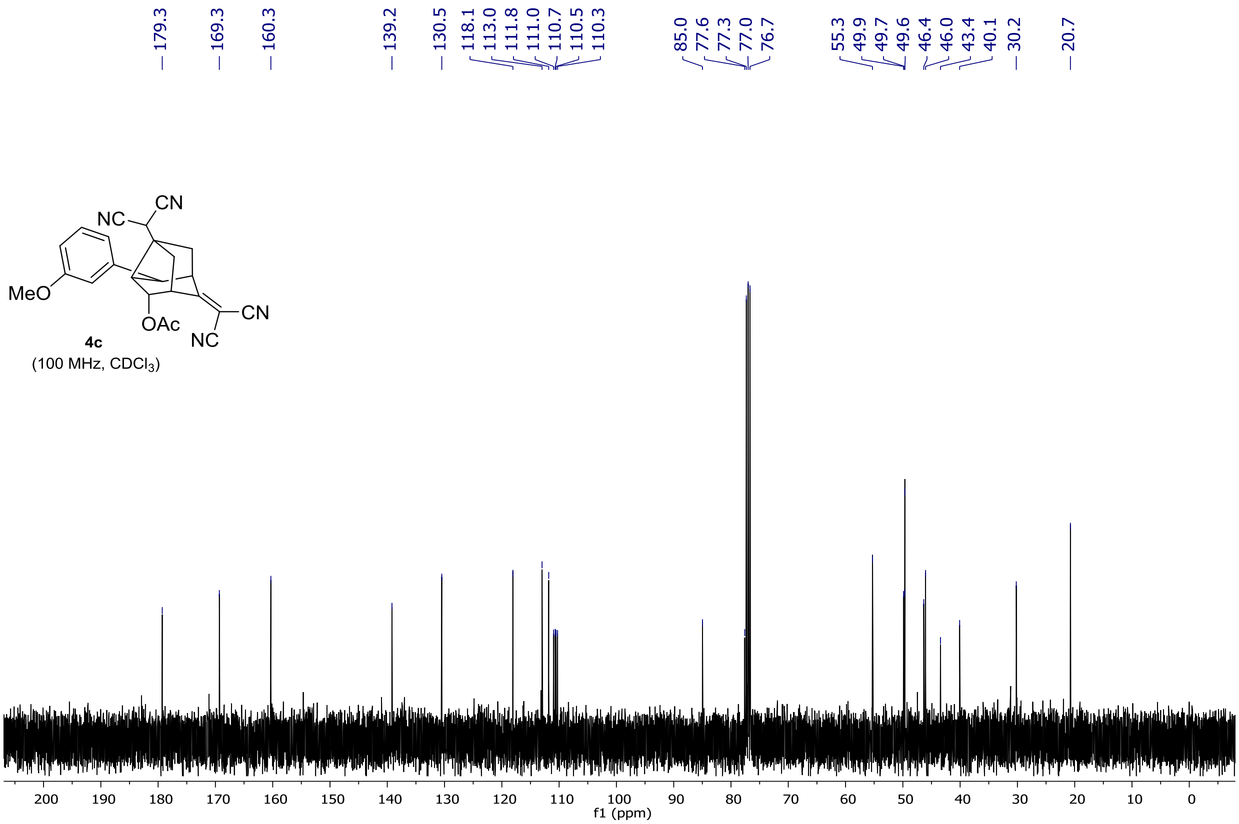

179.3 169.3 160.3 139.2 130.5 118.1 113.0 111.8 111.0 110.7 110.5 110.3
85.0 77.6 77.3 77.0 76.7 55.3 49.9 49.7 49.6 46.4 46.0 43.4 40.1 30.2 20.7
NC
CN
MeO
OAc
CN
NC
4c
(100 MHz, $CDCl_3$)
200 190 180 170 160 150 140 130 120 110 100 90 80 70 60 50 40 30 20 10 0
f1 (ppm)

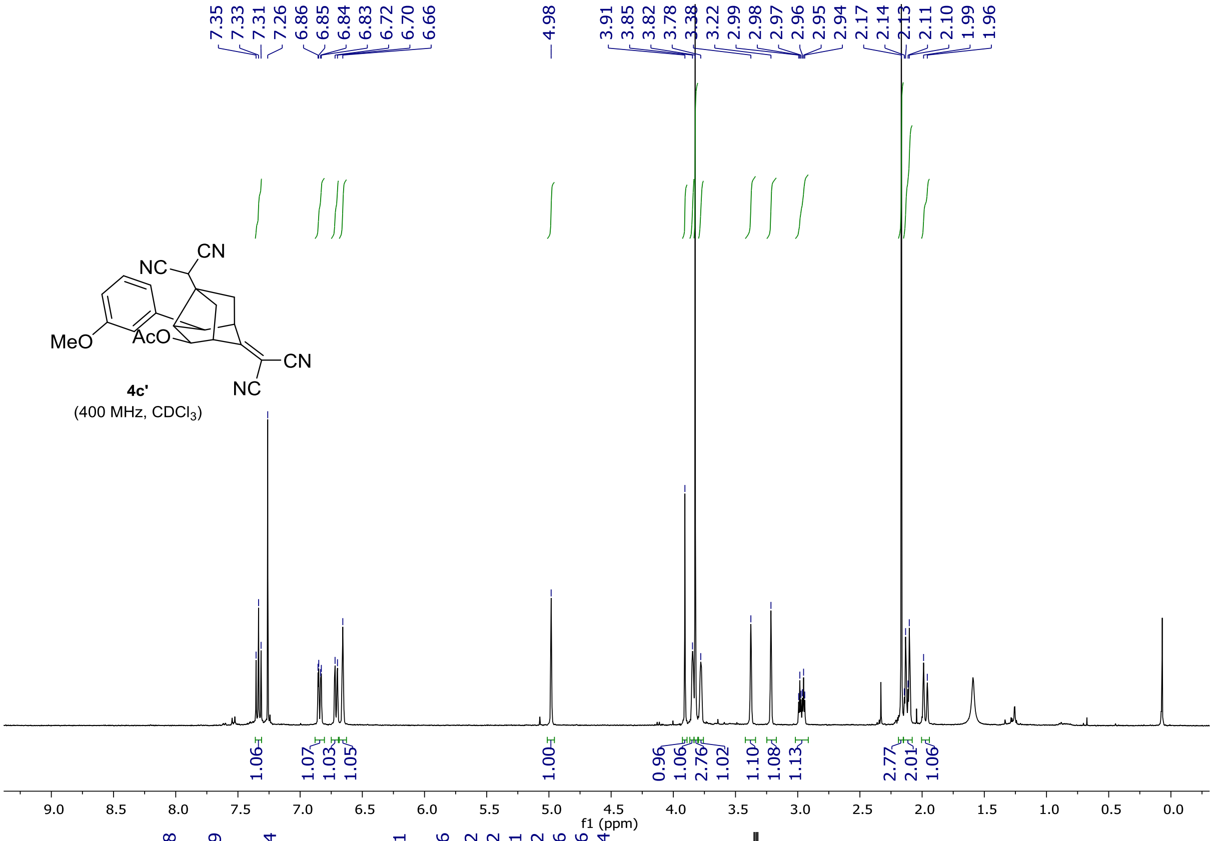
7.35 7.33 7.31 7.26 6.86 6.85 6.84 6.83 6.72 6.70 6.66
4.98
3.91 3.85 3.82 3.78 3.38 3.22 2.99 2.98 2.97 2.96 2.95 2.94 2.17 2.14 2.13 2.11 2.10 1.99 1.96
NC CN
MeO AcO
CN
NC
4c'
(400 MHz, CDCl3)
1.06 1.07 1.03 1.05 1.00 0.96 1.06 2.76 1.02 1.10 1.08 1.13 2.77 2.01 1.06
f1 (ppm)

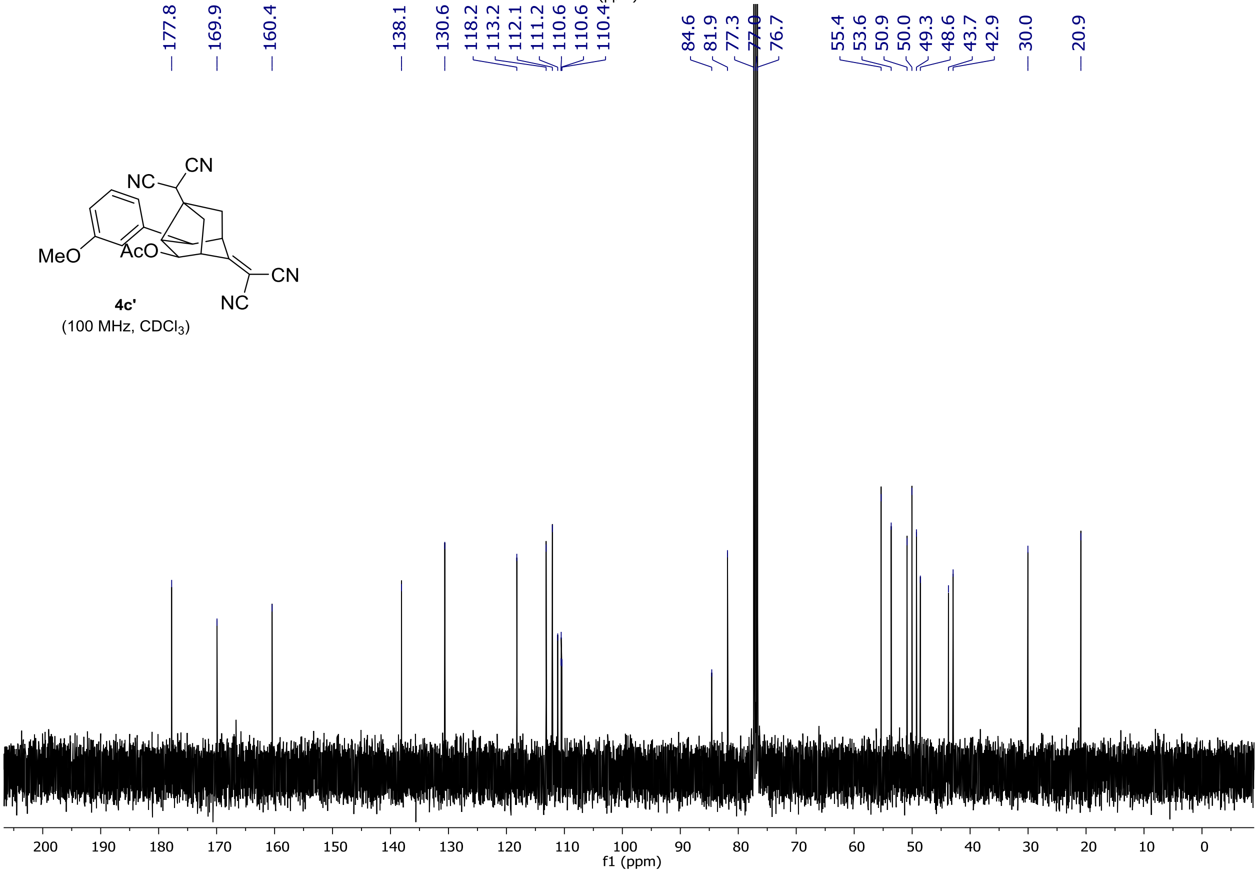
177.8 169.9 160.4 138.1 130.6 118.2 113.2 112.1 111.2 110.6 110.6 110.4
84.6 81.9 77.3 77.0 76.7
55.4 53.6 50.9 50.0 49.3 48.6 43.7 42.9 30.0 20.9
NC CN
MeO AcO
CN
NC
4c'
(100 MHz, CDCl3)
f1 (ppm)

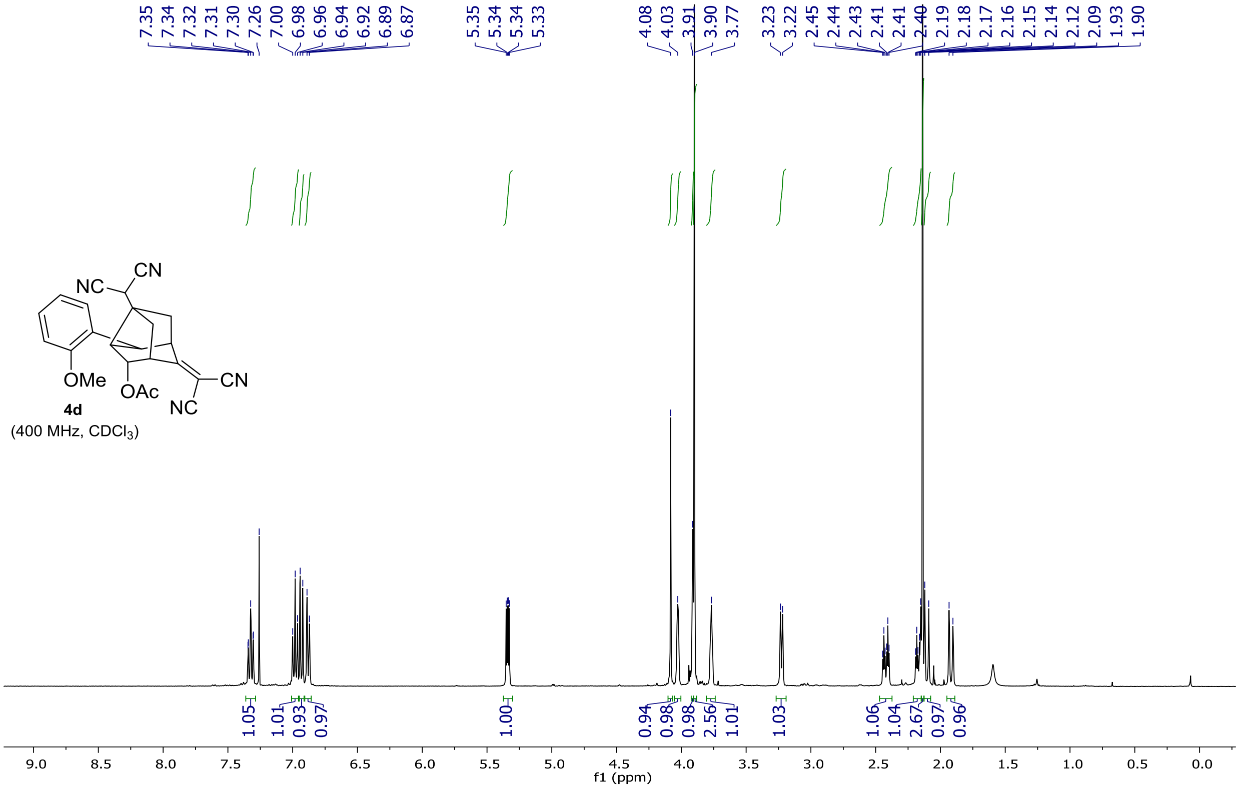
7.35 7.34 7.32 7.31 7.30 7.26 7.00 6.98 6.96 6.94 6.92 6.89 6.87
5.35 5.34 5.34 5.33
4.08 4.03 3.91 3.90 3.77
3.23 3.22
2.45 2.44 2.43 2.41 2.41 2.40 2.19 2.18 2.17 2.16 2.15 2.14 2.12 2.09 1.93 1.90
NC
CN
OMe
OAc
CN
NC
4d
(400 MHz, CDCl3)
1.05 1.01 0.93 0.97 1.00 0.94 0.98 0.98 2.56 1.01 1.03 1.06 1.04 2.67 0.97 0.96
9.0 8.5 8.0 7.5 7.0 6.5 6.0 5.5 5.0 4.5 4.0 3.5 3.0 2.5 2.0 1.5 1.0 0.5 0.0
f1 (ppm)

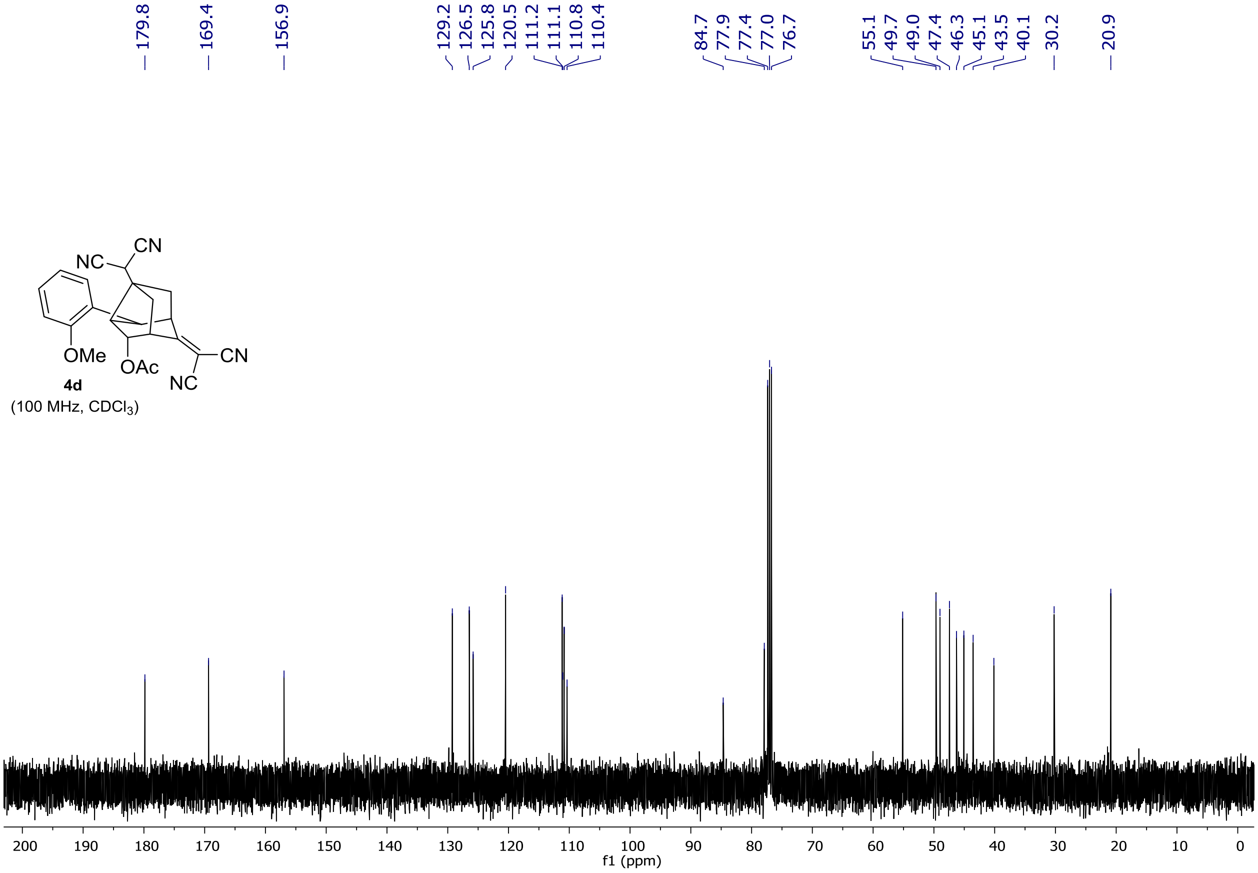
179.8 169.4 156.9
129.2 126.5 125.8 120.5 111.2 111.1 110.8 110.4
84.7 77.9 77.4 77.0 76.7
55.1 49.7 49.0 47.4 46.3 45.1 43.5 40.1 30.2 20.9
NC
CN
OMe
OAc
CN
NC
4d
(100 MHz, CDCl3)
200 190 180 170 160 150 140 130 120 110 100 90 80 70 60 50 40 30 20 10 0
f1 (ppm)

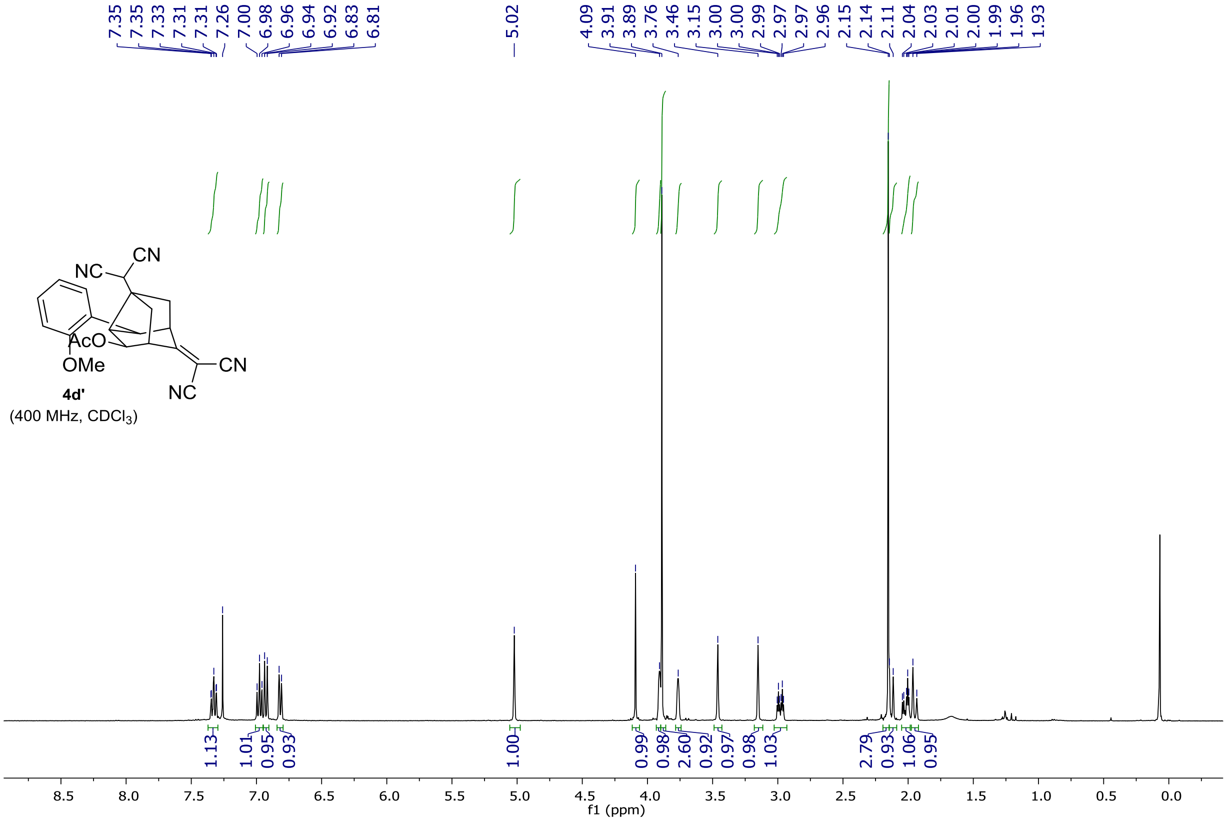
7.35 7.35 7.33 7.31 7.31 7.26 7.00 6.98 6.96 6.94 6.92 6.83 6.81
5.02
4.09 3.91 3.89 3.76 3.46 3.15 3.00 3.00 2.99 2.97 2.97 2.96 2.15 2.14 2.11 2.04 2.03 2.01 2.00 1.99 1.96 1.93
NC
CN
AcO
OMe
CN
NC
4d'
(400 MHz, $CDCl_3$)
1.13 1.01 0.95 0.93 1.00 0.99 0.98 2.60 0.92 0.97 0.98 1.03 2.79 0.93 1.06 0.95
f1 (ppm)

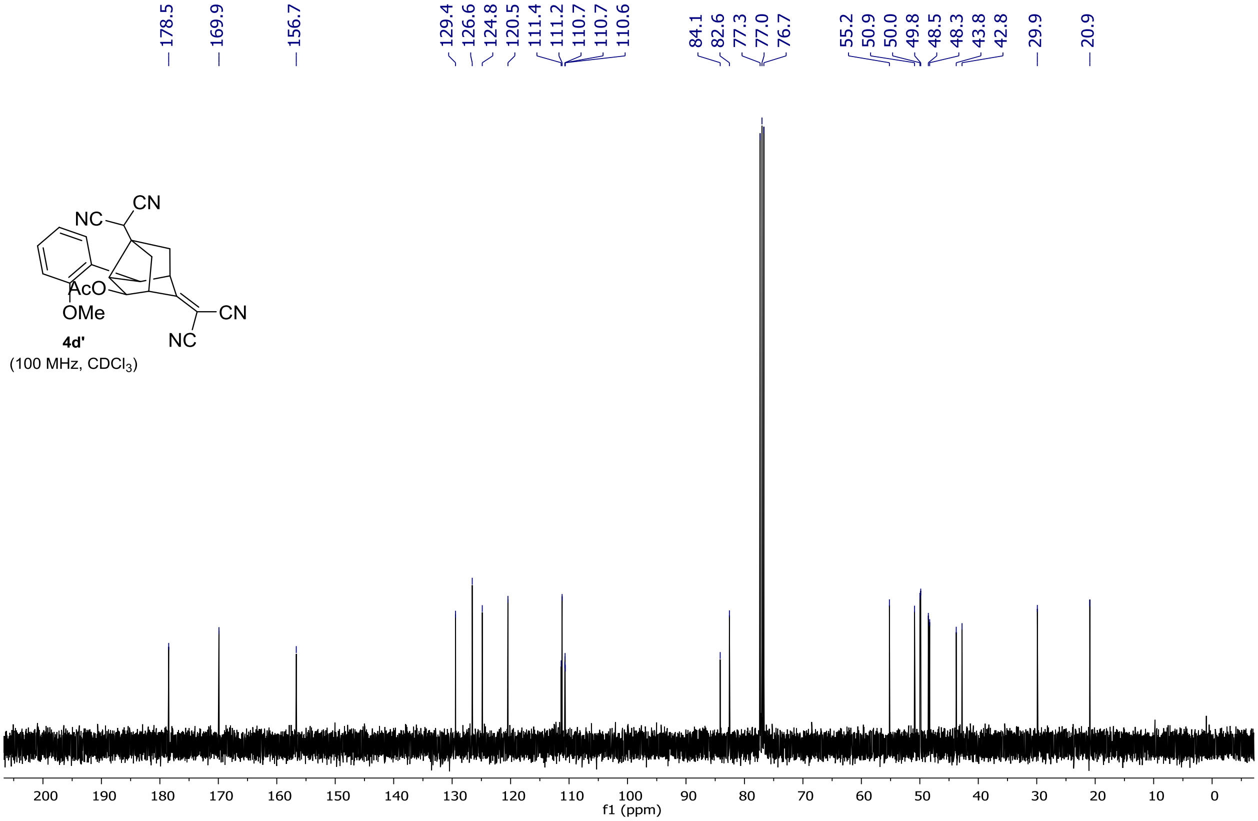
178.5 169.9 156.7 129.4 126.6 124.8 120.5 111.4 111.2 110.7 110.7 110.6 84.1 82.6 77.3 77.0 76.7 55.2 50.9 50.0 49.8 48.5 48.3 43.8 42.8 29.9 20.9
NC
CN
AcO
OMe
CN
NC
4d'
(100 MHz, $CDCl_3$)
f1 (ppm)

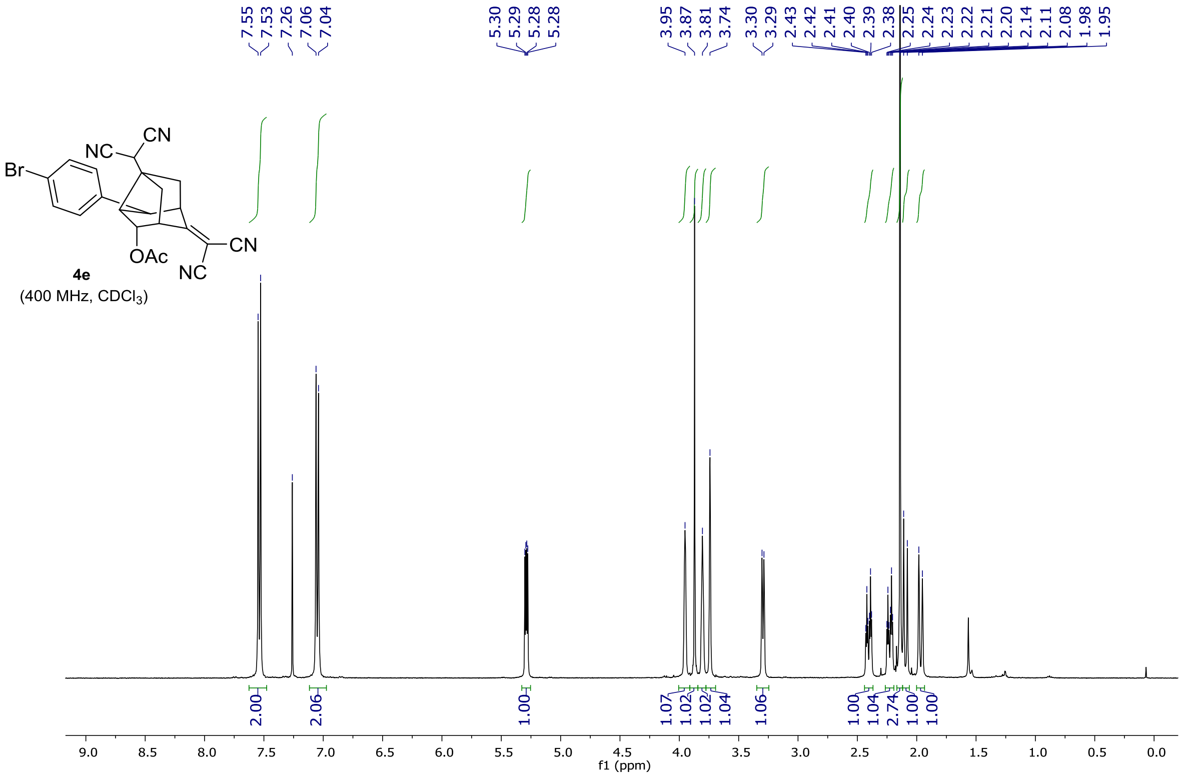
7.55
7.53
7.26
7.06
7.04
5.30
5.29
5.28
5.28
3.95
3.87
3.81
3.74
3.30
3.29
2.43
2.42
2.41
2.40
2.39
2.38
2.25
2.24
2.23
2.22
2.21
2.20
2.14
2.11
2.08
1.98
1.95
Br
NC
CN
OAc
CN
NC
4e
(400 MHz, $CDCl_3$)
2.00
2.06
1.00
1.07
1.02
1.02
1.04
1.06
1.00
1.04
2.74
1.00
1.00
9.0 8.5 8.0 7.5 7.0 6.5 6.0 5.5 5.0 4.5 4.0 3.5 3.0 2.5 2.0 1.5 1.0 0.5 0.0
f1 (ppm)

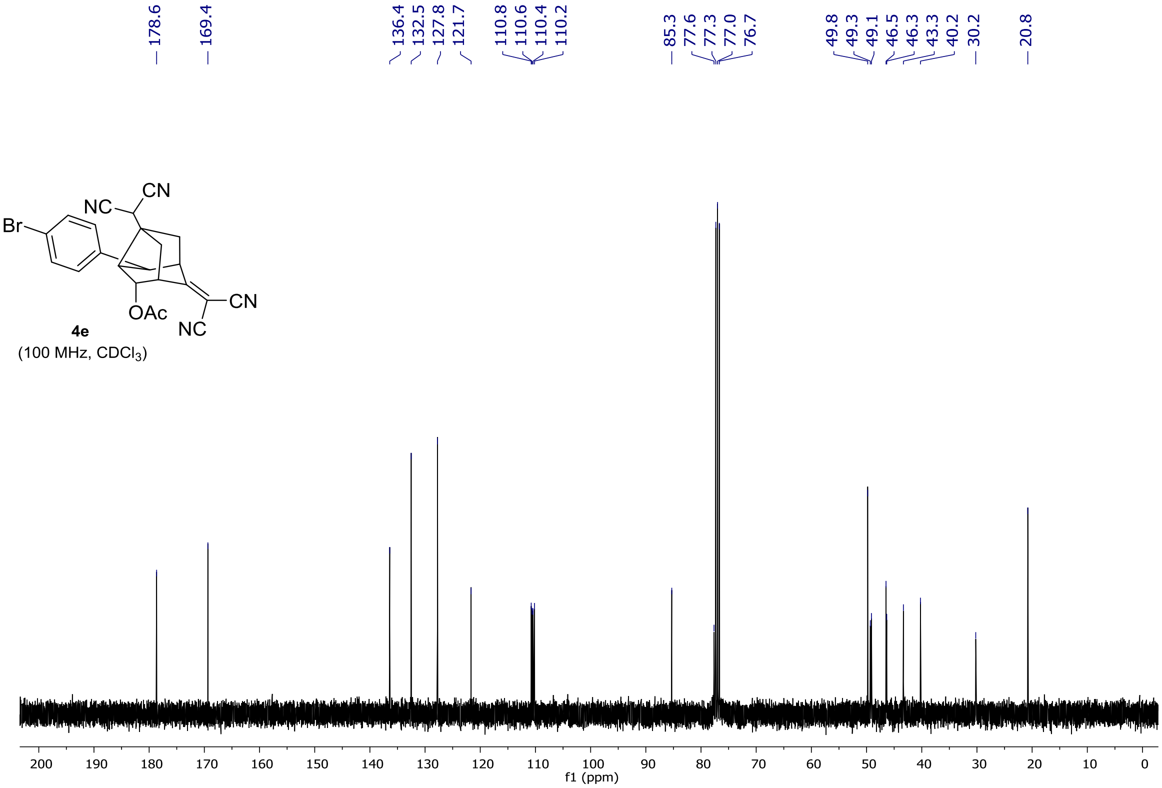
178.6
169.4
136.4
132.5
127.8
121.7
110.8
110.6
110.4
110.2
85.3
77.6
77.3
77.0
76.7
49.8
49.3
49.1
46.5
46.3
43.3
40.2
30.2
20.8
Br
NC
CN
OAc
CN
NC
4e
(100 MHz, $CDCl_3$)
200 190 180 170 160 150 140 130 120 110 100 90 80 70 60 50 40 30 20 10 0
f1 (ppm)

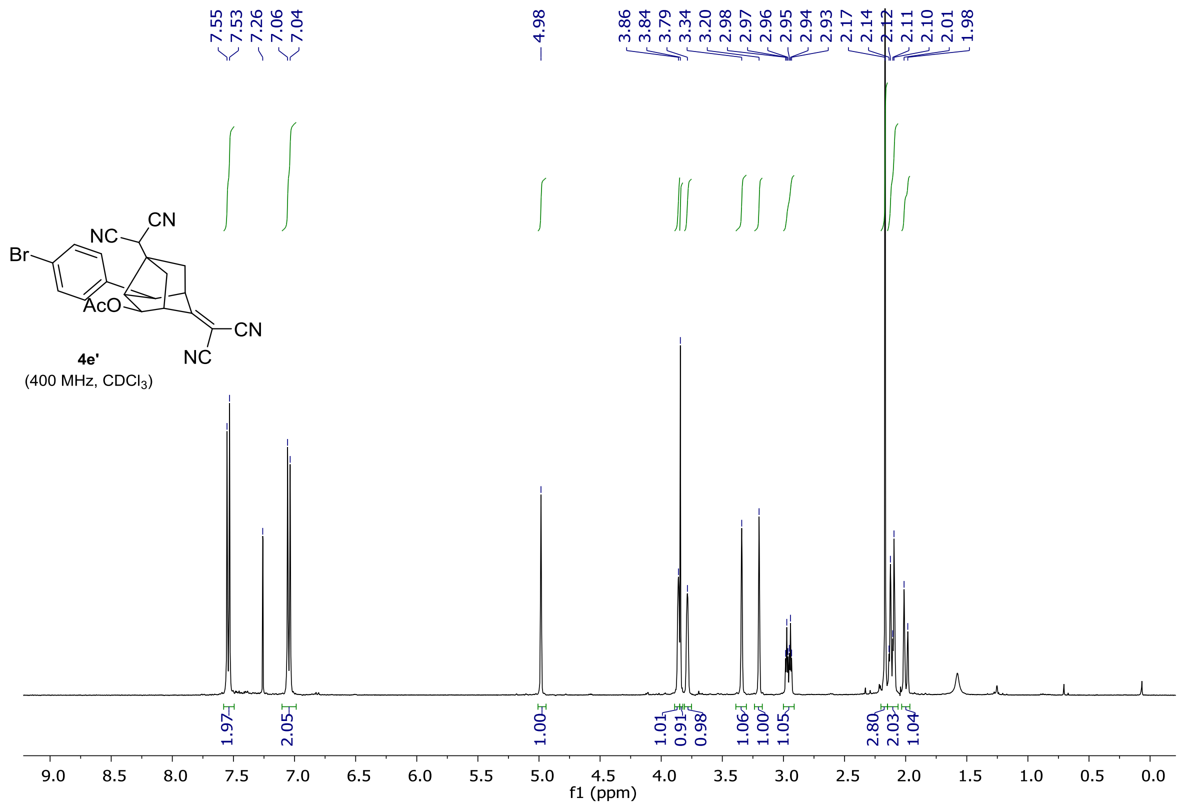

7.55
7.53
7.26
7.06
7.04
4.98
3.86
3.84
3.79
3.34
3.20
2.98
2.97
2.96
2.95
2.94
2.93
2.17
2.14
2.12
2.11
2.10
2.01
1.98
CN
NC
Br
AcO
CN
NC
4e'
(400 MHz, CDCl3)
1.97
2.05
1.00
1.01
0.91
0.98
1.06
1.00
1.05
2.80
2.03
1.04
f1 (ppm)

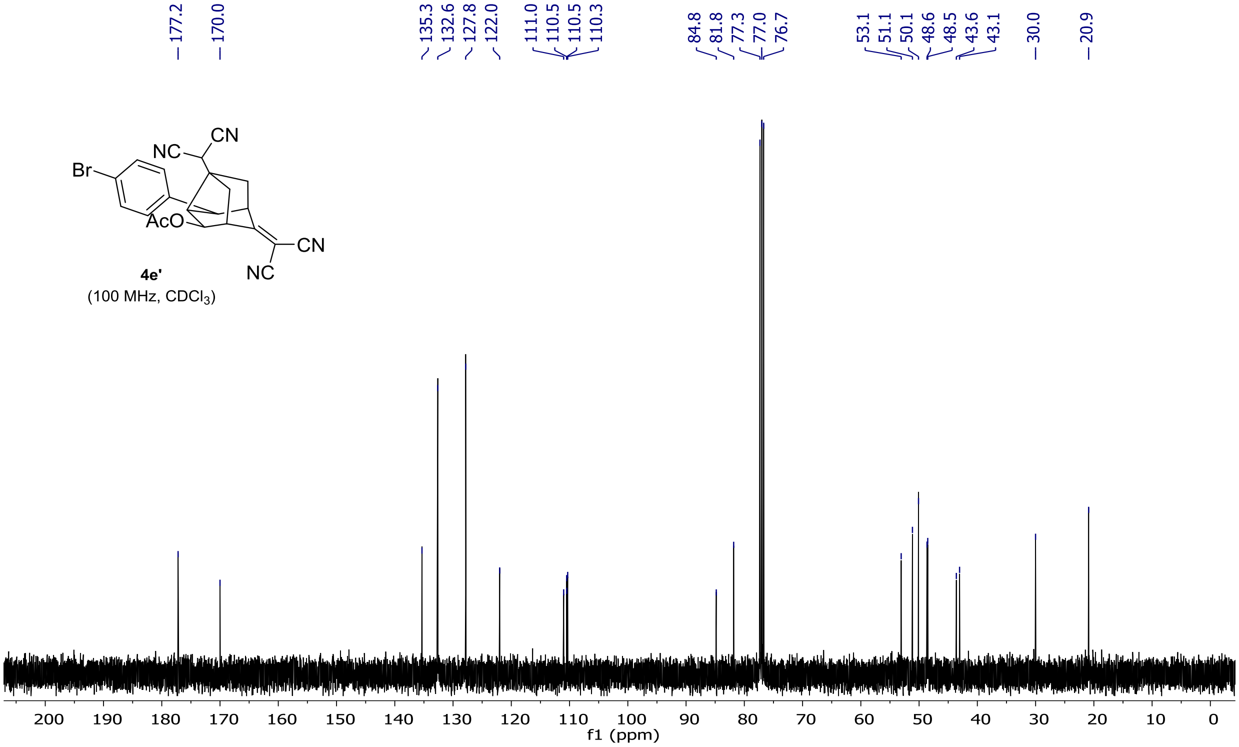

177.2
170.0
135.3
132.6
127.8
122.0
111.0
110.5
110.5
110.3
84.8
81.8
77.3
77.0
76.7
53.1
51.1
50.1
48.6
48.5
43.6
43.1
30.0
20.9
CN
NC
Br
AcO
CN
NC
4e'
(100 MHz, CDCl3)
f1 (ppm)

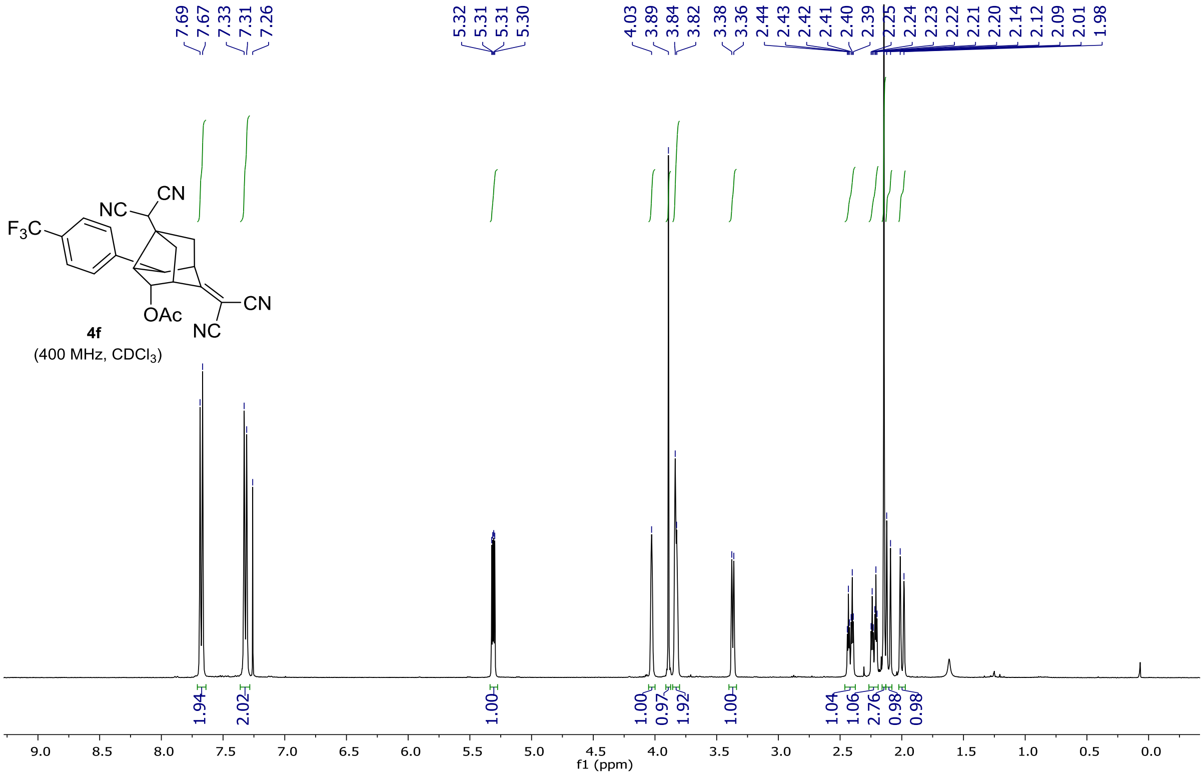
7.69 7.67 7.33 7.31 7.26
5.32 5.31 5.31 5.30
4.03 3.89 3.84 3.82 3.38 3.36 2.44 2.43 2.42 2.41 2.40 2.39 2.25 2.24 2.23 2.22 2.21 2.20 2.14 2.12 2.09 2.01 1.98
NC
CN
F3C
CN
OAc
NC
4f
(400 MHz, CDCl3)
1.94 2.02 1.00 1.00 0.97 1.92 1.00 1.04 1.06 2.76 0.98 0.98
f1 (ppm)

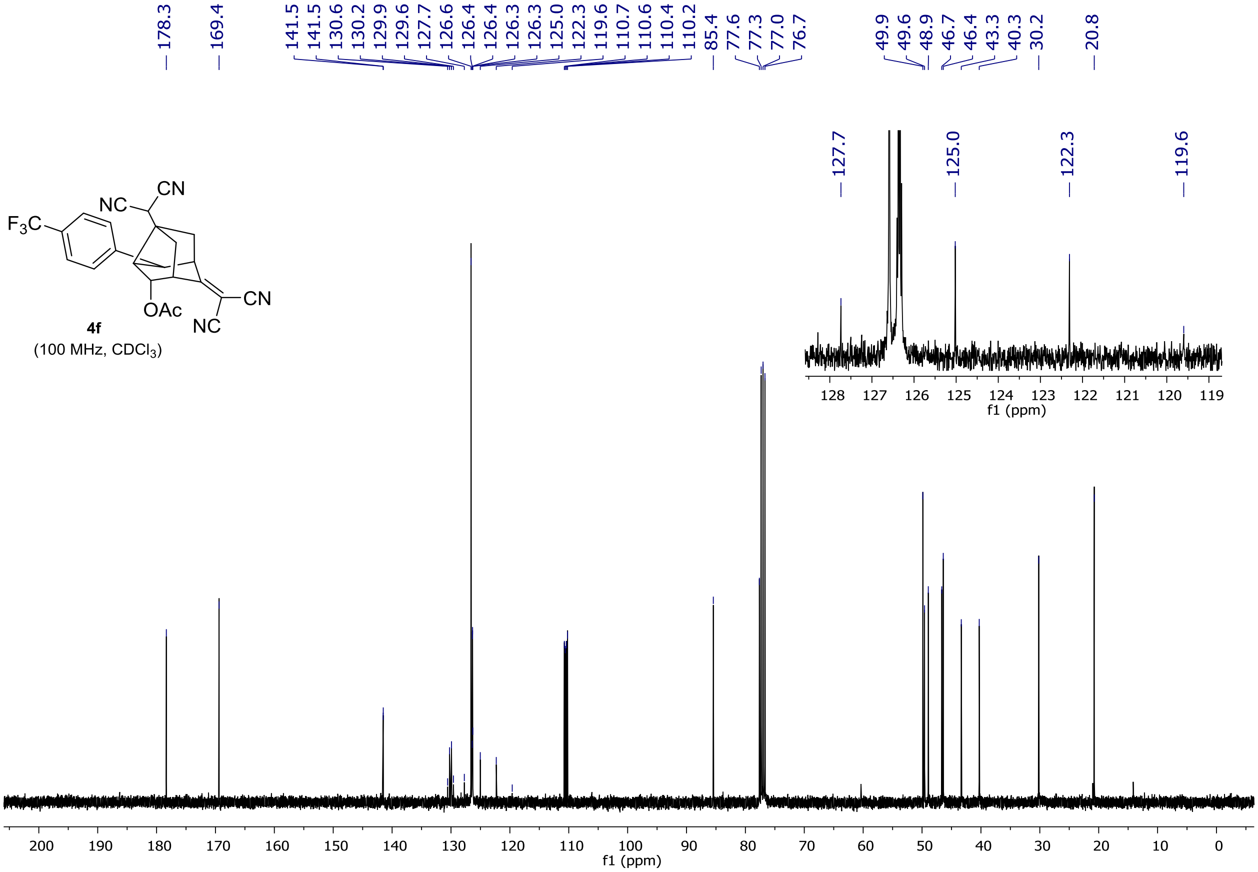
178.3 169.4
141.5 141.5 130.6 130.2 129.9 129.6 127.7 126.6 126.4 126.4 126.3 126.3 125.0 122.3 119.6 110.7 110.6 110.4 110.2
85.4 77.6 77.3 77.0 76.7
49.9 49.6 48.9 46.7 46.4 43.3 40.3 30.2 20.8
127.7 125.0 122.3 119.6
NC
CN
F3C
CN
OAc
NC
4f
(100 MHz, CDCl3)
f1 (ppm)

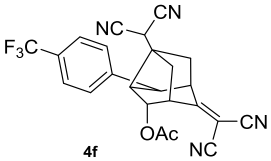

4f

(376 MHz, $CDCl_3$)

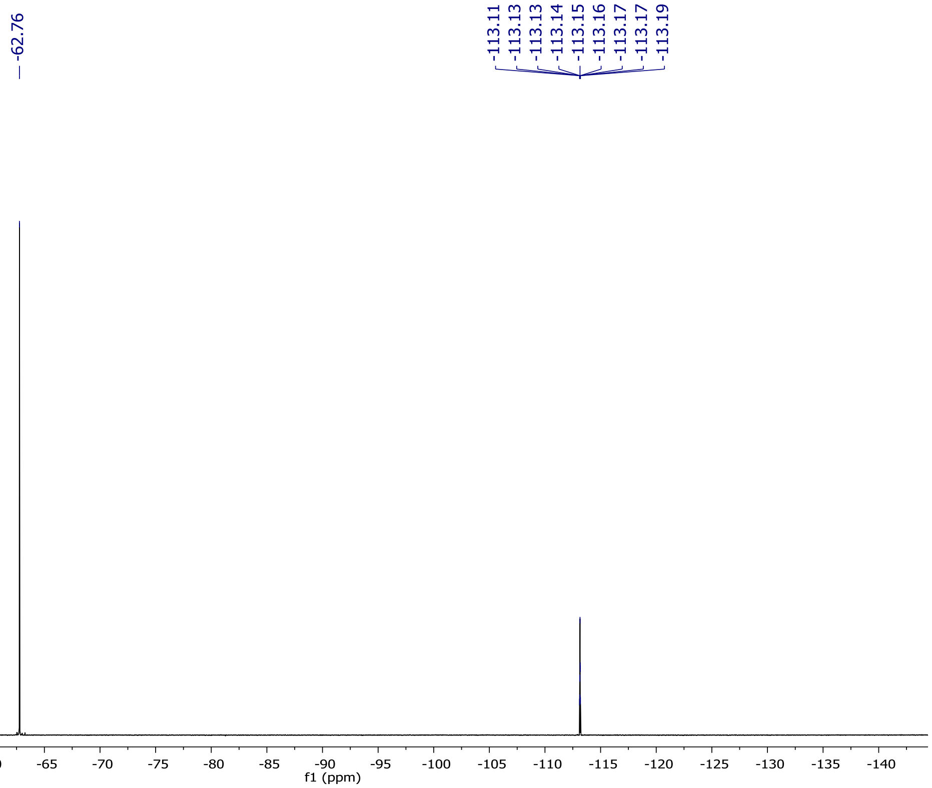

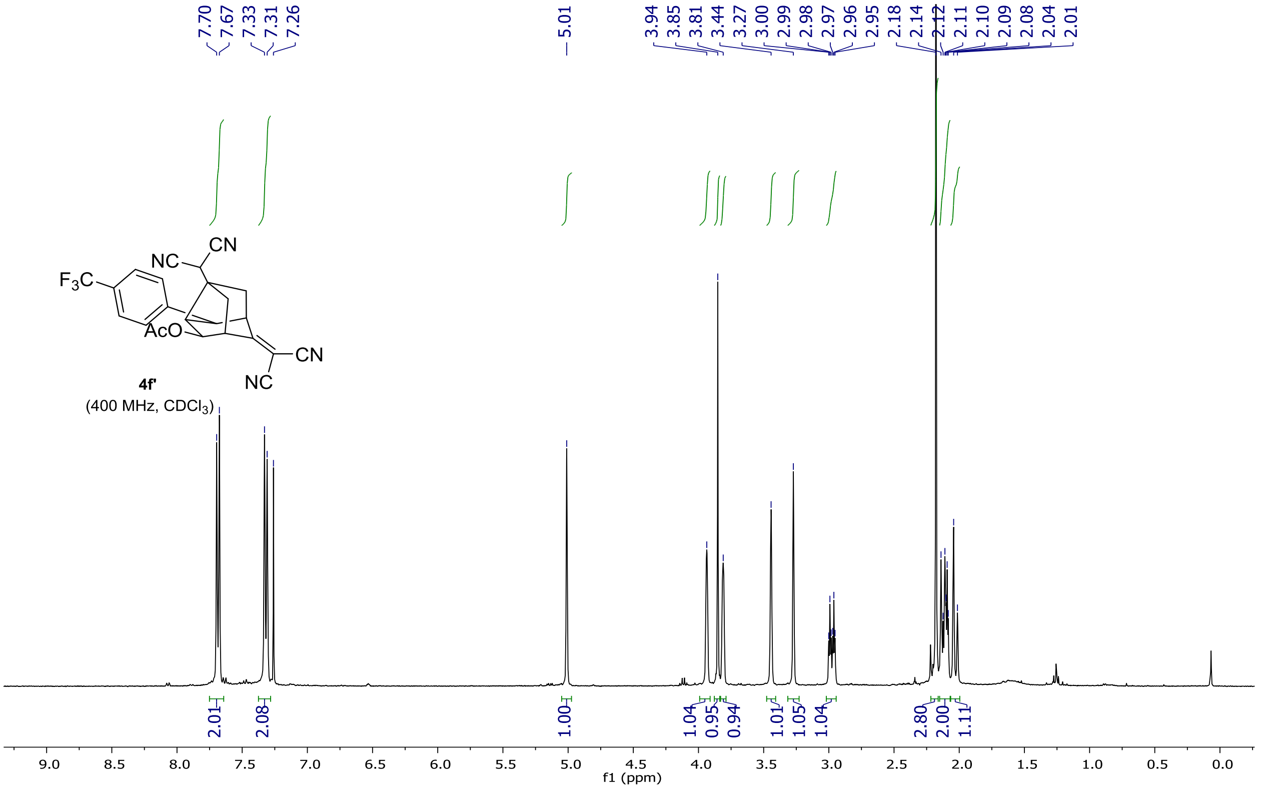
7.70 7.67 7.33 7.31 7.26
5.01
3.94 3.85 3.81 3.44 3.27 3.00 2.99 2.98 2.97 2.96 2.95 2.18 2.14 2.12 2.11 2.10 2.09 2.08 2.04 2.01
F_3C
NC
CN
AcO
CN
NC
4f'
(400 MHz, $CDCl_3$)
2.01 2.08 1.00 1.04 0.95 0.94 1.01 1.05 1.04 2.80 2.00 1.11
9.0 8.5 8.0 7.5 7.0 6.5 6.0 5.5 5.0 4.5 4.0 3.5 3.0 2.5 2.0 1.5 1.0 0.5 0.0
f1 (ppm)

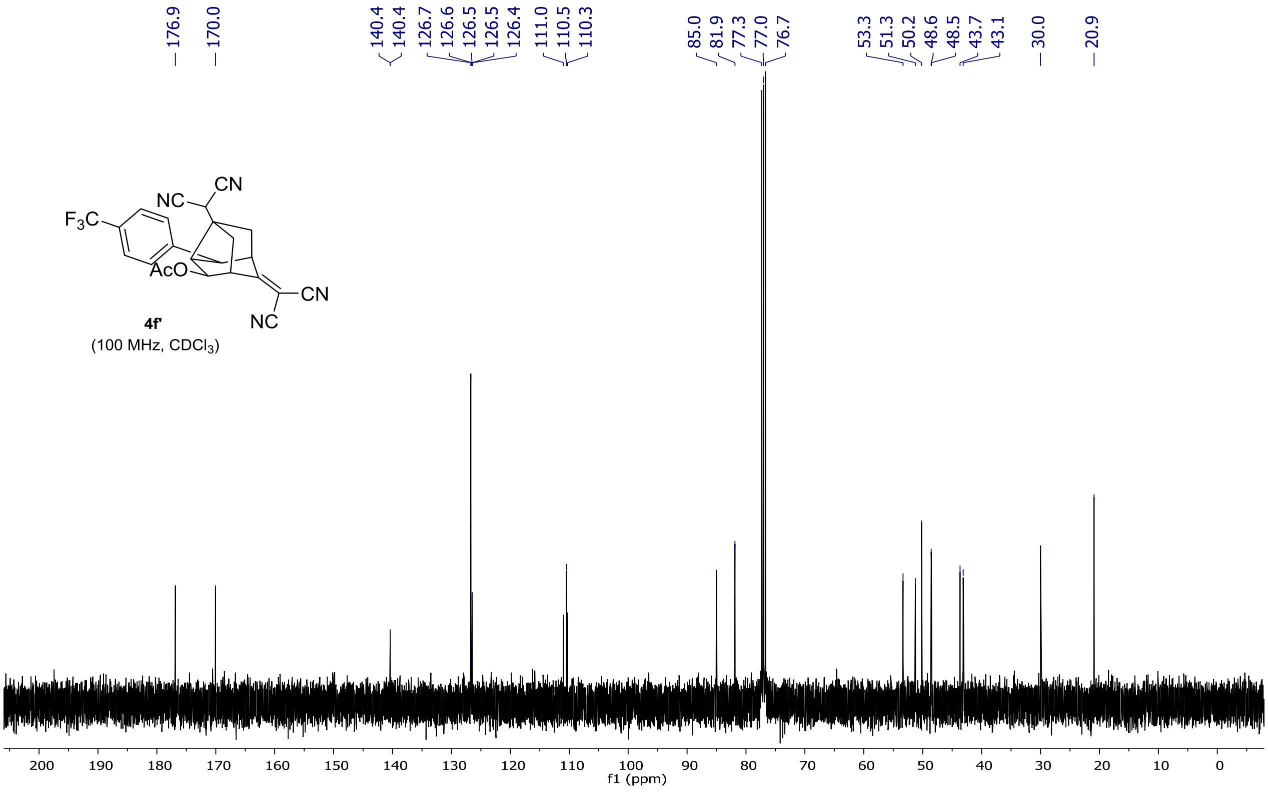
176.9 170.0 140.4 140.4 126.7 126.6 126.5 126.5 126.4 111.0 110.5 110.3 85.0 81.9 77.3 77.0 76.7 53.3 51.3 50.2 48.6 48.5 43.7 43.1 30.0 20.9
F_3C
NC
CN
AcO
CN
NC
4f'
(100 MHz, $CDCl_3$)
200 190 180 170 160 150 140 130 120 110 100 90 80 70 60 50 40 30 20 10 0
f1 (ppm)

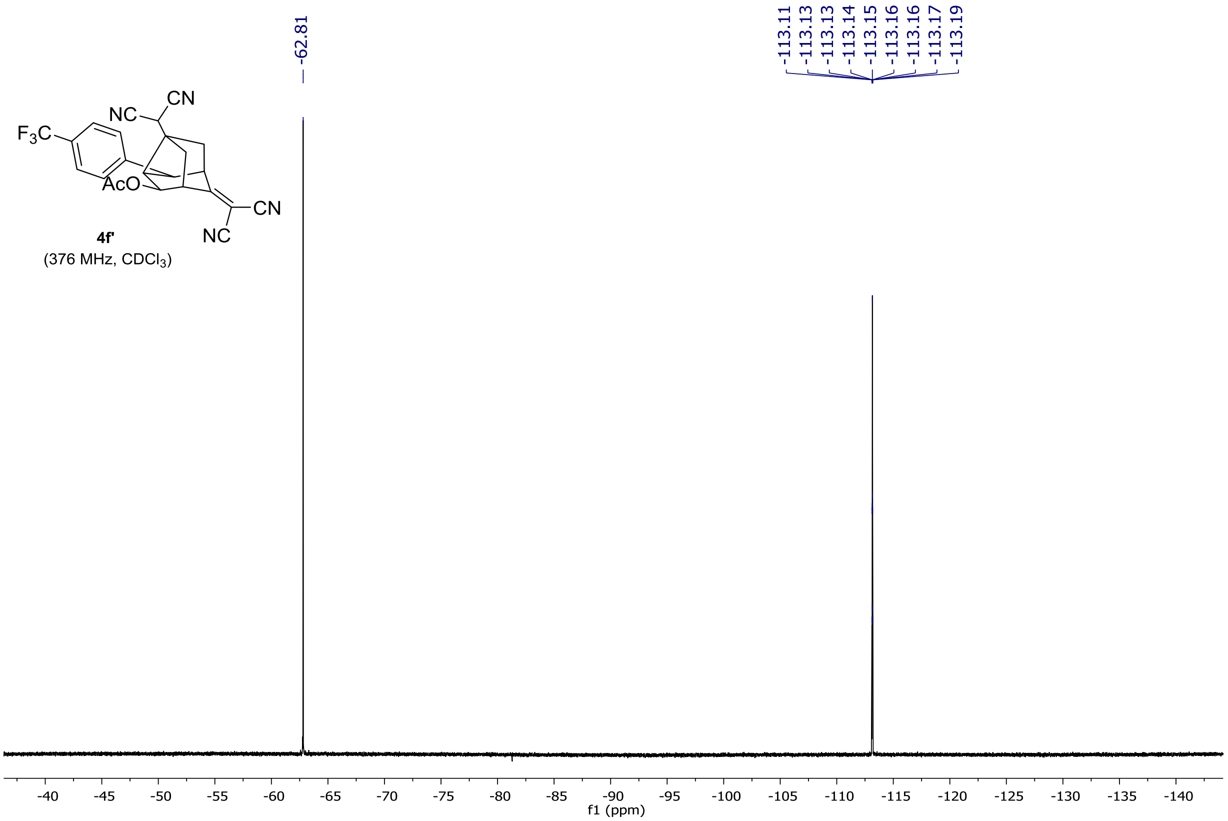

-62.81
-113.11
-113.13
-113.13
-113.14
-113.15
-113.16
-113.16
-113.17
-113.19
NC
CN
F3C
AcO
CN
NC
4f'
(376 MHz, CDCl3)
-40 -45 -50 -55 -60 -65 -70 -75 -80 -85 -90 -95 -100 -105 -110 -115 -120 -125 -130 -135 -140
f1 (ppm)

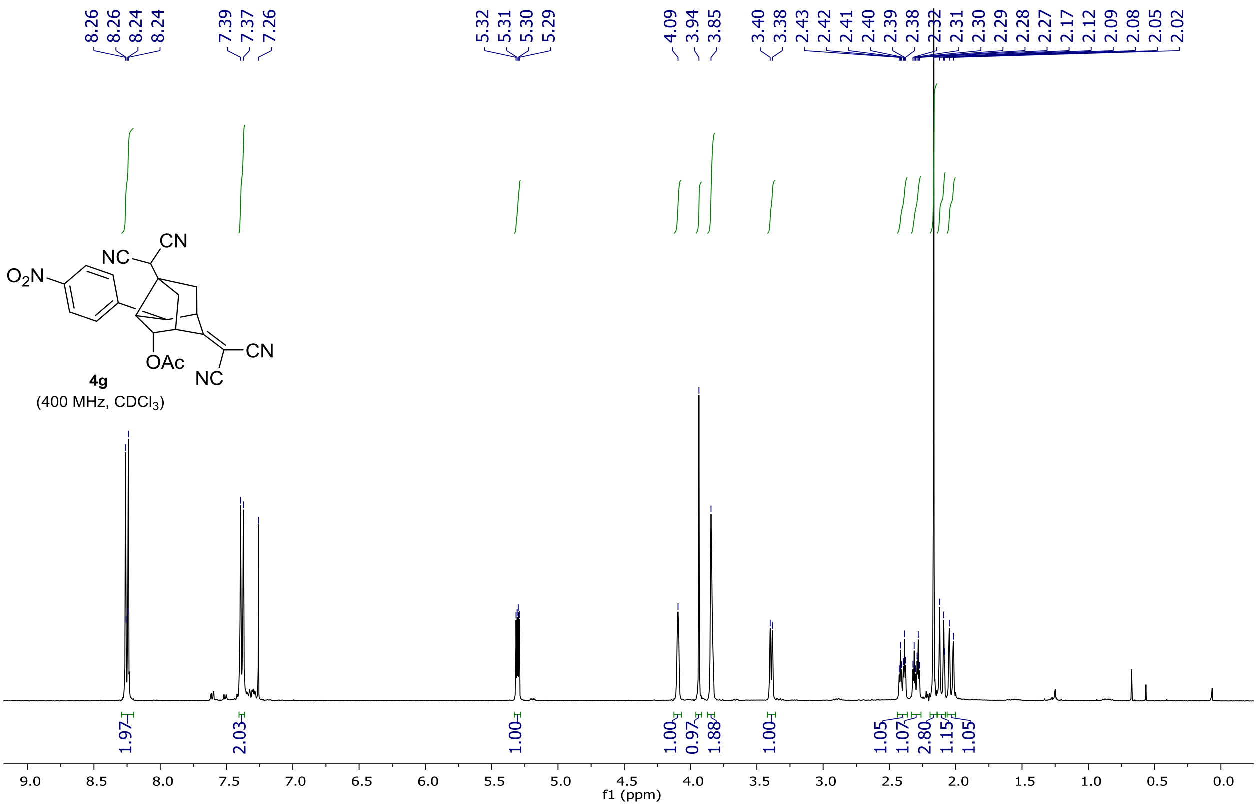

4g
(400 MHz, CDCl3)
f1 (ppm)

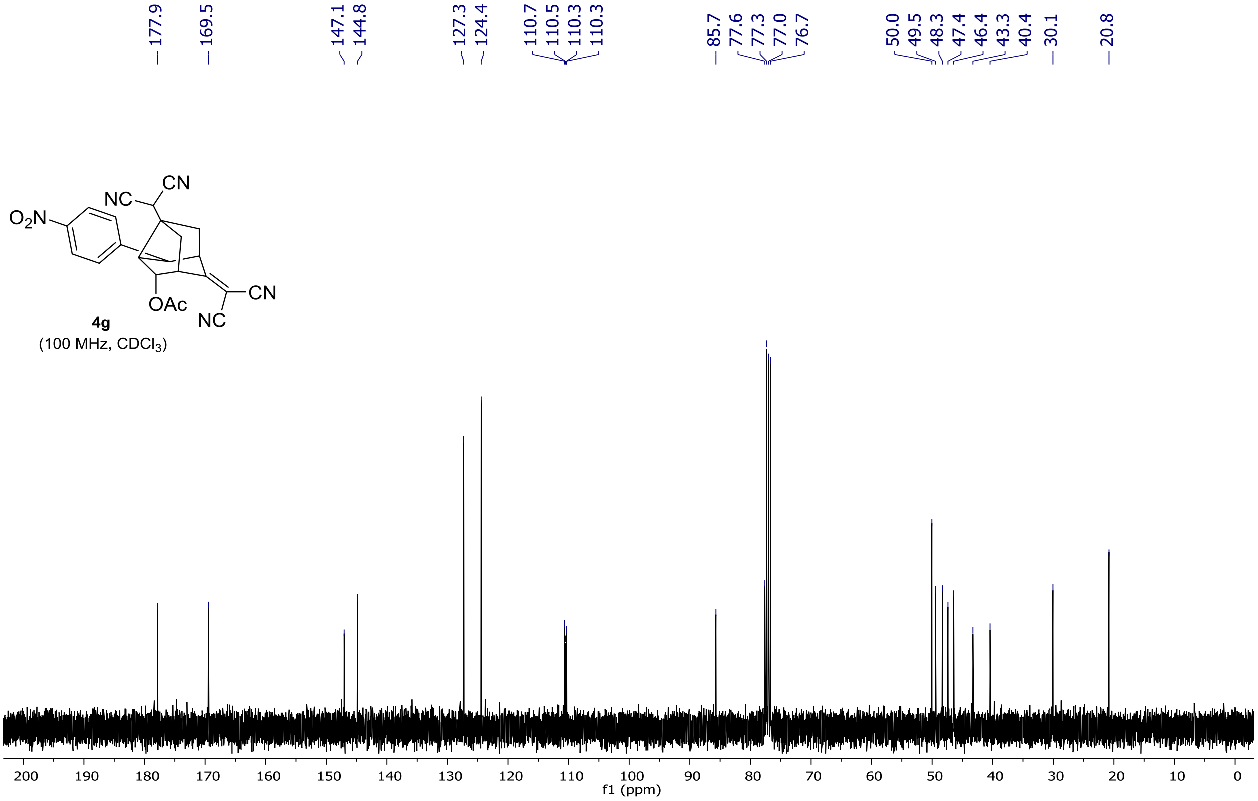

4g
(100 MHz, CDCl3)
f1 (ppm)

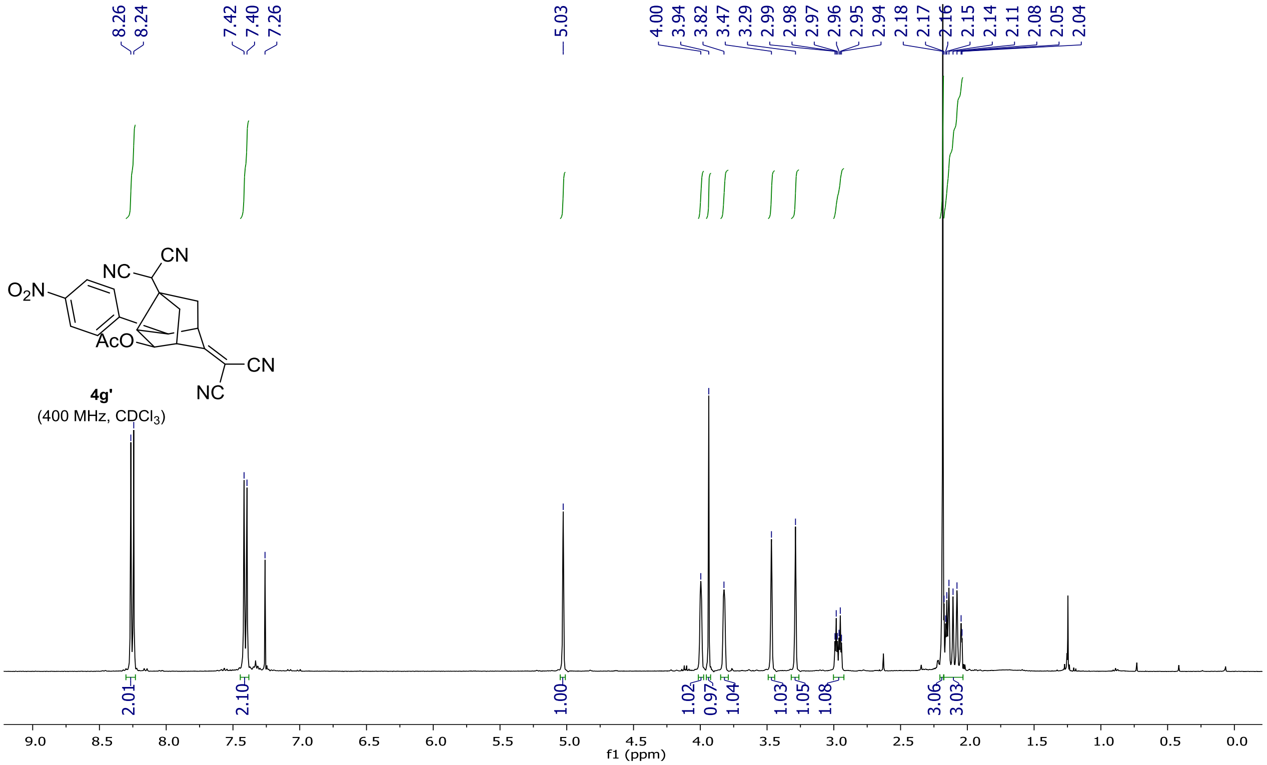
8.26
8.24
7.42
7.40
7.26
5.03
4.00
3.94
3.82
3.47
3.29
2.99
2.98
2.97
2.96
2.95
2.94
2.18
2.17
2.16
2.15
2.14
2.11
2.08
2.05
2.04
O_2N
NC
CN
AcO
CN
NC
4g'
(400 MHz, $CDCl_3$)
2.01
2.10
1.00
1.02
0.97
1.04
1.03
1.05
1.08
3.06
3.03
9.0
8.5
8.0
7.5
7.0
6.5
6.0
5.5
5.0
4.5
4.0
3.5
3.0
2.5
2.0
1.5
1.0
0.5
0.0
f1 (ppm)

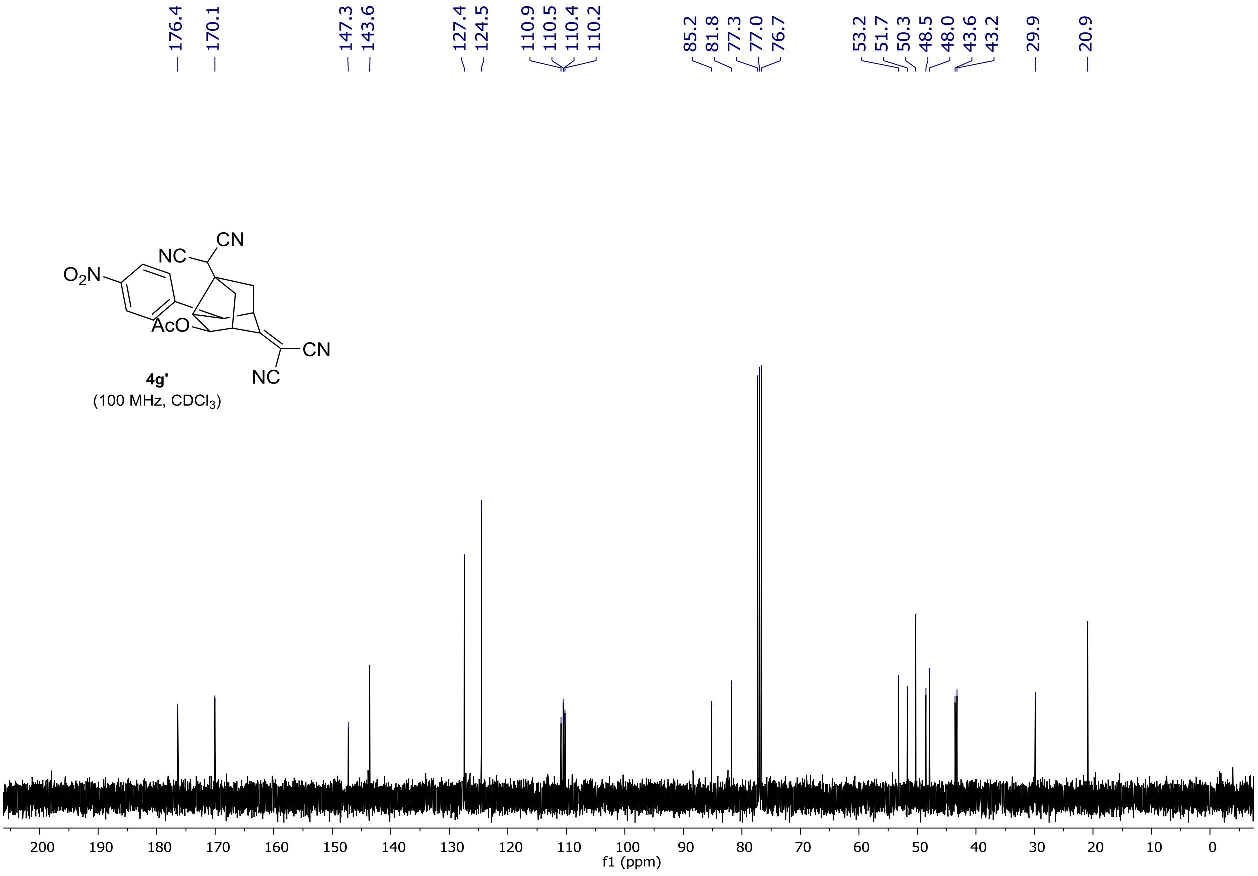
176.4
170.1
147.3
143.6
127.4
124.5
110.9
110.5
110.4
110.2
85.2
81.8
77.3
77.0
76.7
53.2
51.7
50.3
48.5
48.0
43.6
43.2
29.9
20.9
O_2N
NC
CN
AcO
CN
NC
4g'
(100 MHz, $CDCl_3$)
200
190
180
170
160
150
140
130
120
110
100
90
80
70
60
50
40
30
20
10
0
f1 (ppm)

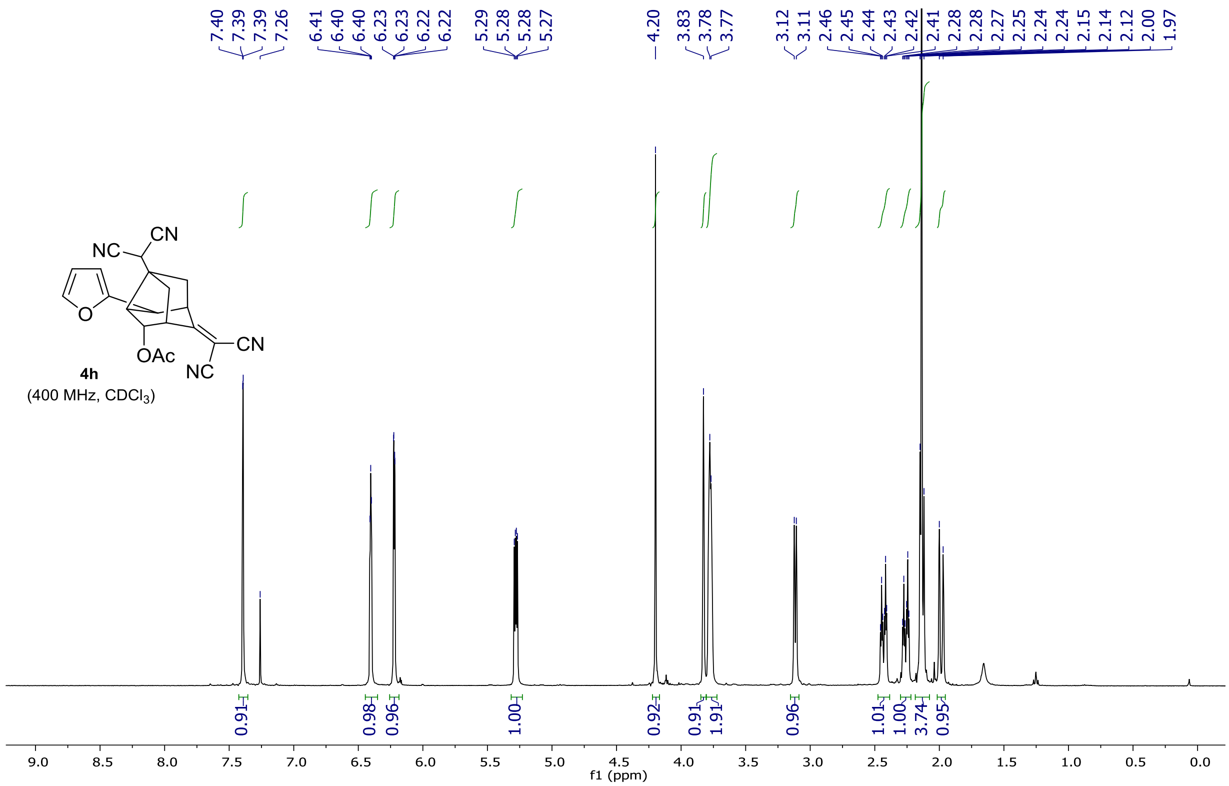
7.40 7.39 7.39 7.26 6.41 6.40 6.40 6.23 6.23 6.22 6.22 5.29 5.28 5.28 5.27 4.20 3.83 3.78 3.77 3.12 3.11 2.46 2.45 2.44 2.43 2.42 2.41 2.28 2.28 2.27 2.25 2.24 2.24 2.15 2.14 2.12 2.00 1.97
NC
CN
O
OAc
CN
NC
4h
(400 MHz, $CDCl_3$)
0.91 0.98 0.96 1.00 0.92 0.91 1.91 0.96 1.01 1.00 3.74 0.95
f1 (ppm)

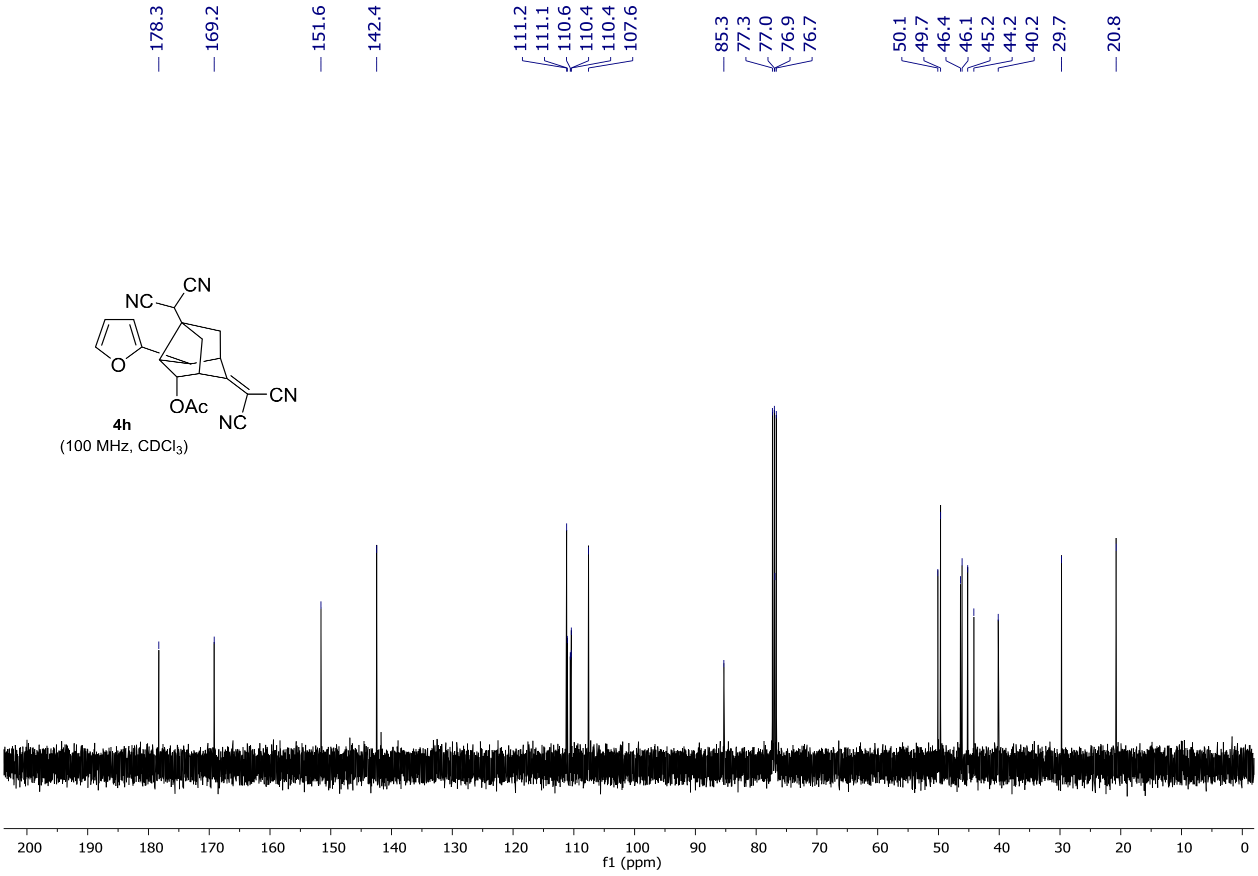
178.3 169.2 151.6 142.4 111.2 111.1 110.6 110.4 110.4 107.6 85.3 77.3 77.0 76.9 76.7 50.1 49.7 46.4 46.1 45.2 44.2 40.2 29.7 20.8
NC
CN
O
OAc
CN
NC
4h
(100 MHz, $CDCl_3$)
f1 (ppm)

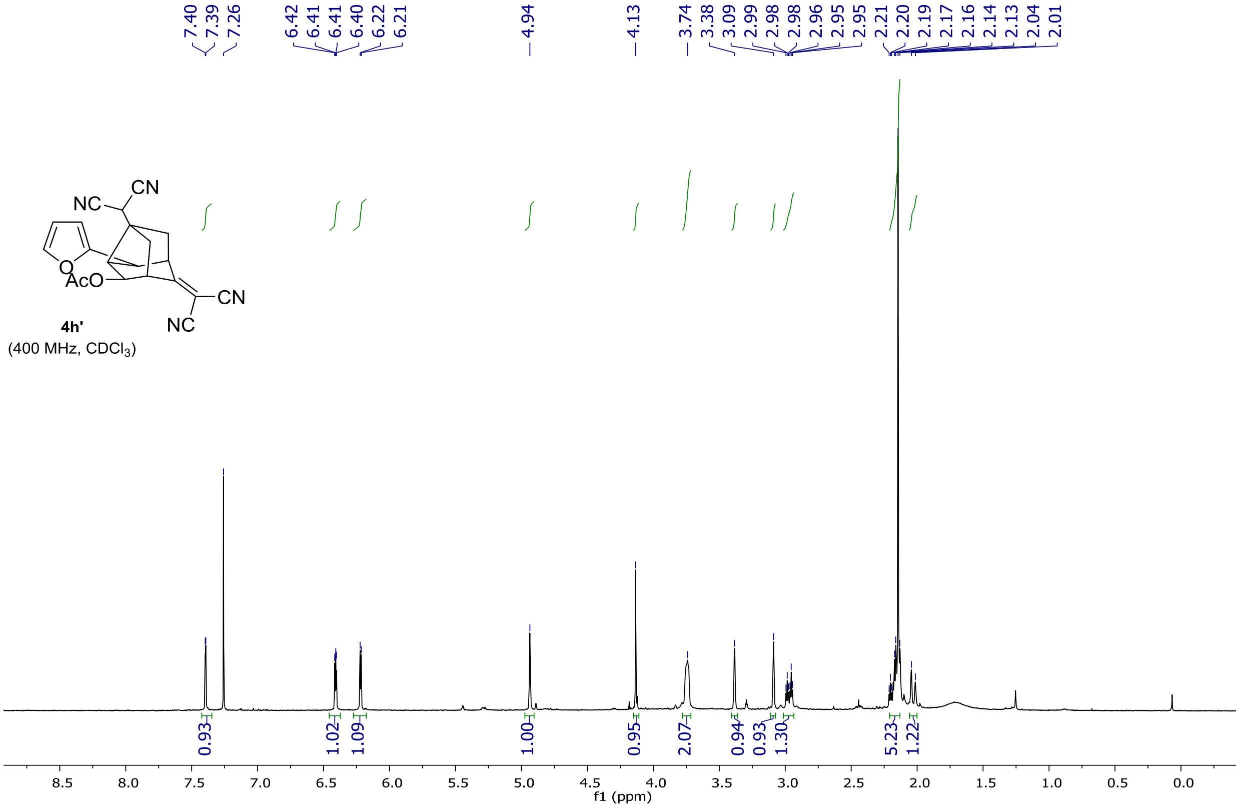
7.40
7.39
7.26
6.42
6.41
6.41
6.40
6.22
6.21
4.94
4.13
3.74
3.38
3.09
2.99
2.98
2.98
2.96
2.95
2.95
2.21
2.20
2.19
2.17
2.16
2.14
2.13
2.04
2.01
NC
CN
AcO
O
CN
NC
4h'
(400 MHz, CDCl3)
0.93
1.02
1.09
1.00
0.95
2.07
0.94
0.93
1.30
5.23
1.22
8.5 8.0 7.5 7.0 6.5 6.0 5.5 5.0 4.5 4.0 3.5 3.0 2.5 2.0 1.5 1.0 0.5 0.0
f1 (ppm)

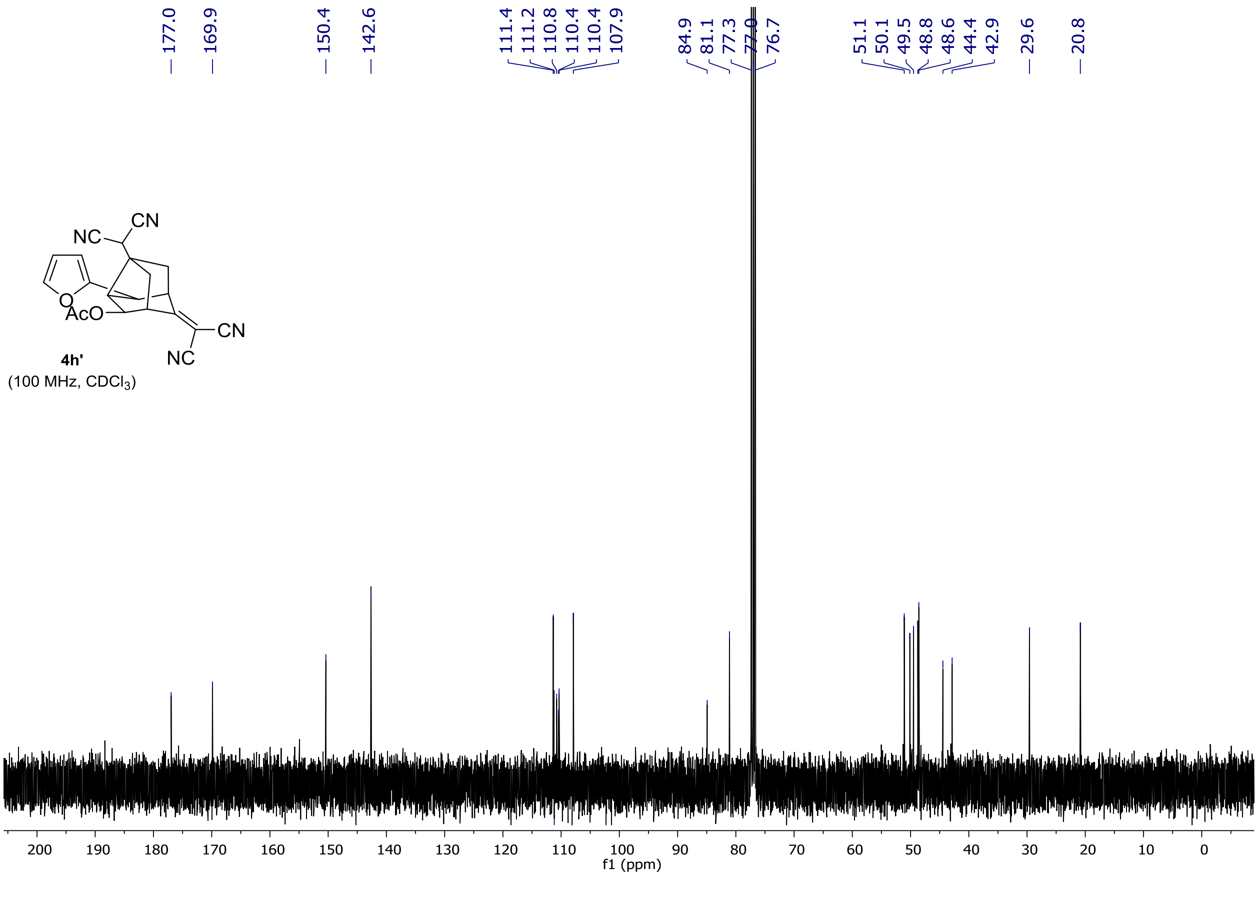
177.0
169.9
150.4
142.6
111.4
111.2
110.8
110.4
110.4
107.9
84.9
81.1
77.3
77.0
76.7
51.1
50.1
49.5
48.8
48.6
44.4
42.9
29.6
20.8
NC
CN
AcO
O
CN
NC
4h'
(100 MHz, CDCl3)
200 190 180 170 160 150 140 130 120 110 100 90 80 70 60 50 40 30 20 10 0
f1 (ppm)

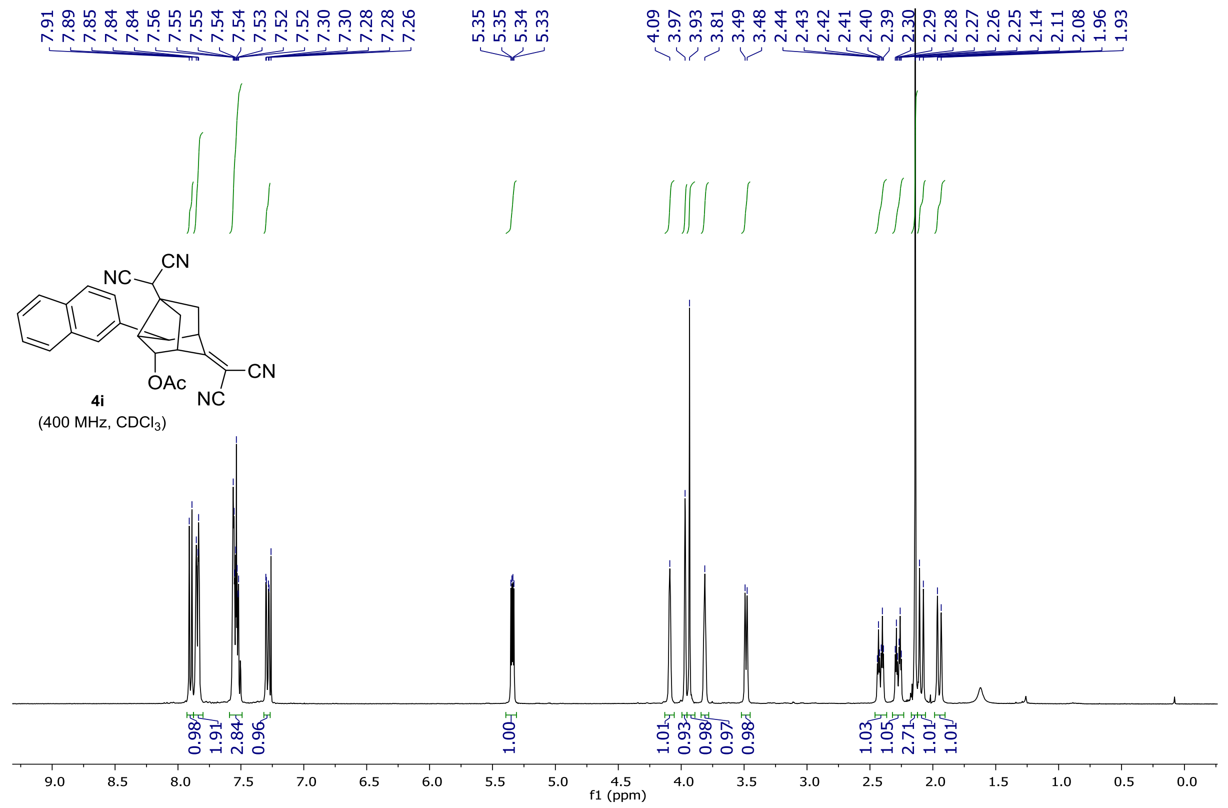
7.91 7.89 7.85 7.84 7.84 7.56 7.55 7.55 7.54 7.54 7.53 7.52 7.52 7.30 7.30 7.28 7.28 7.26
5.35 5.35 5.34 5.33
4.09 3.97 3.93 3.81 3.49 3.48 2.44 2.43 2.42 2.41 2.40 2.39 2.30 2.29 2.28 2.27 2.26 2.25 2.14 2.11 2.08 1.96 1.93
NC
CN
CN
OAc
NC
4i
(400 MHz, CDCl3)
0.98 1.91 2.84 0.96 1.00 1.01 0.93 0.98 0.97 0.98 1.03 1.05 2.71 1.01 1.01
f1 (ppm)

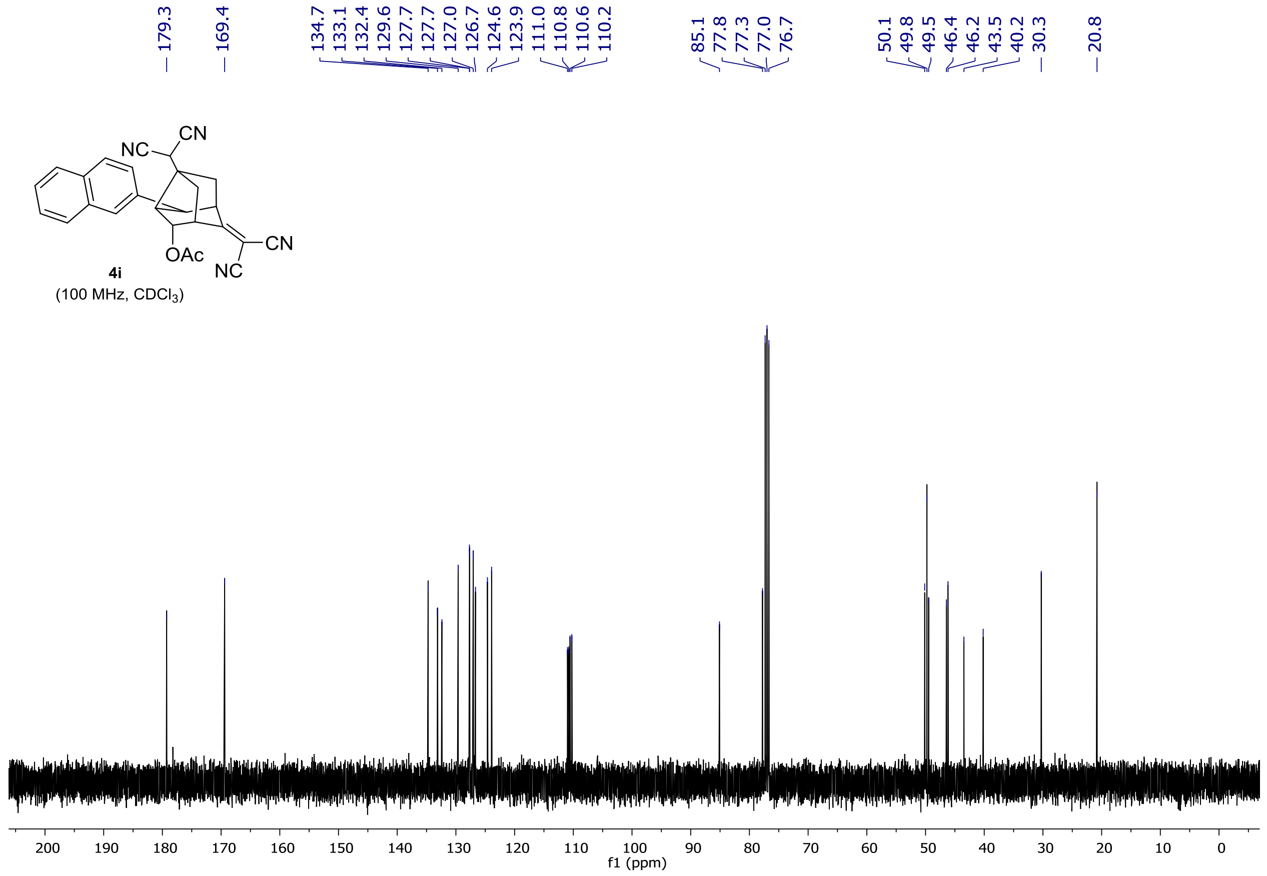
179.3 169.4 134.7 133.1 132.4 129.6 127.7 127.7 127.0 126.7 124.6 123.9 111.0 110.8 110.6 110.2 85.1 77.8 77.3 77.0 76.7 50.1 49.8 49.5 46.4 46.2 43.5 40.2 30.3 20.8
NC
CN
CN
OAc
NC
4i
(100 MHz, CDCl3)
f1 (ppm)

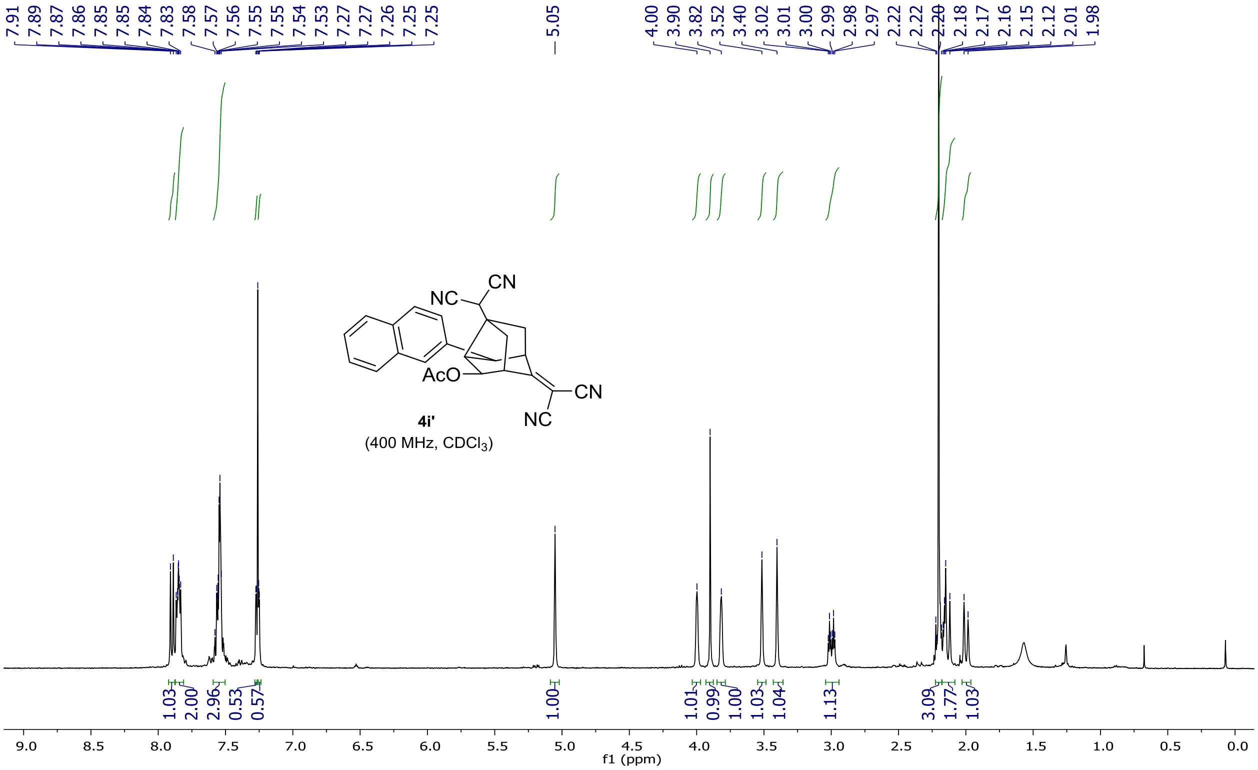
7.91 7.89 7.87 7.86 7.85 7.85 7.84 7.83 7.58 7.57 7.56 7.55 7.55 7.54 7.53 7.27 7.27 7.26 7.25 7.25
5.05
4.00 3.90 3.82 3.52 3.40 3.02 3.01 3.00 2.99 2.98 2.97 2.22 2.22 2.20 2.18 2.17 2.16 2.15 2.12 2.01 1.98
NC
CN
AcO
CN
NC
4i'
(400 MHz, CDCl3)
1.03 2.00 2.96 0.53 0.57 1.00 1.01 0.99 1.00 1.03 1.04 1.13 3.09 1.77 1.03
f1 (ppm)

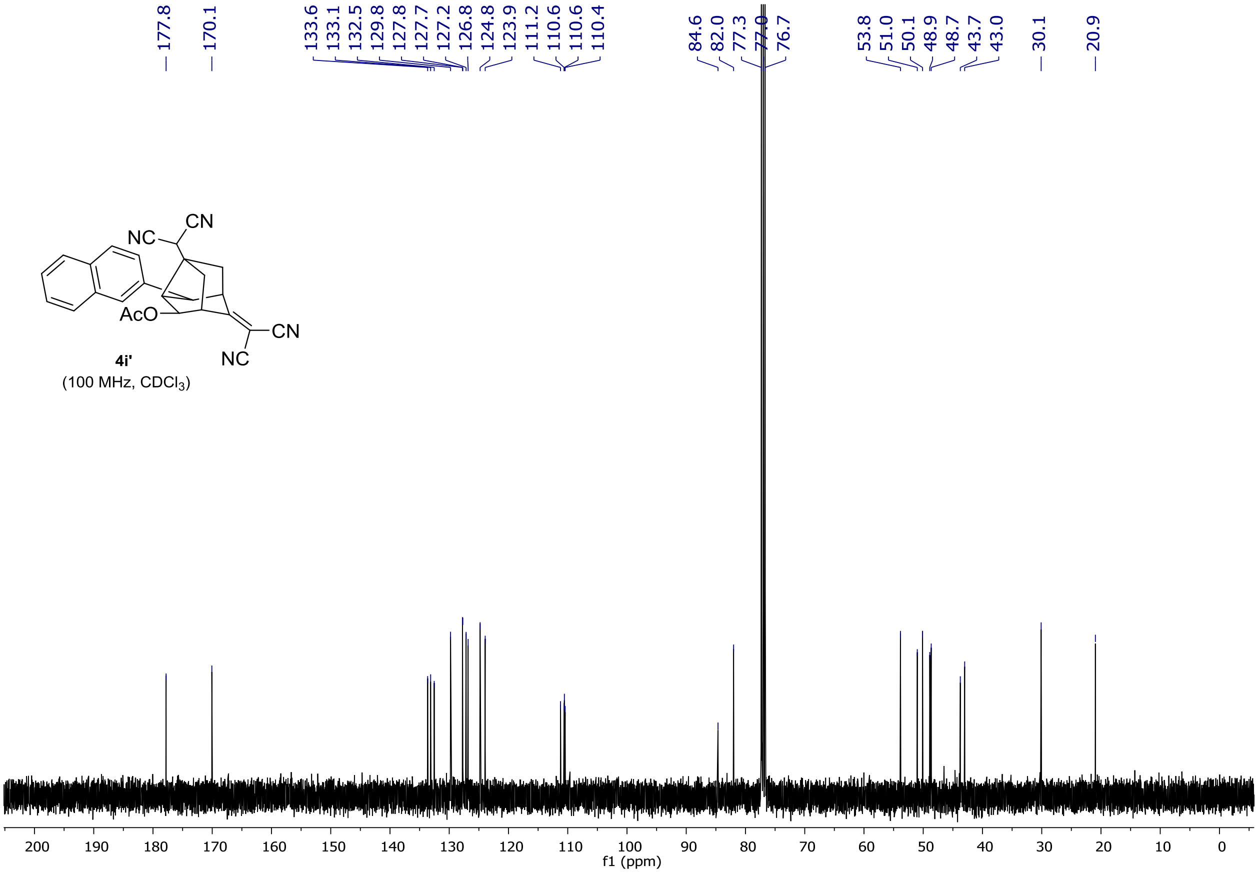
177.8 170.1 133.6 133.1 132.5 129.8 127.8 127.7 127.2 126.8 124.8 123.9 111.2 110.6 110.6 110.4 84.6 82.0 77.3 77.0 76.7 53.8 51.0 50.1 48.9 48.7 43.7 43.0 30.1 20.9
NC
CN
AcO
CN
NC
4i'
(100 MHz, CDCl3)
f1 (ppm)

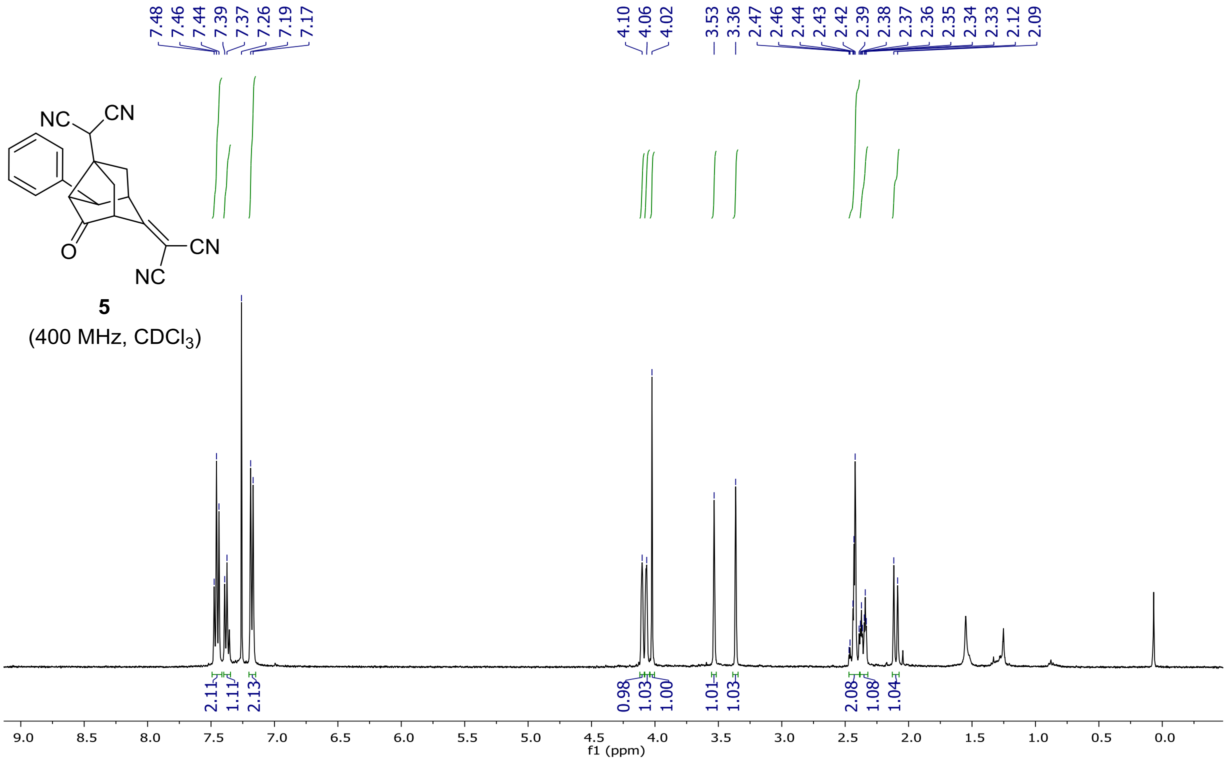
7.48 7.46 7.44 7.39 7.37 7.26 7.19 7.17
4.10 4.06 4.02
3.53 3.36
2.47 2.46 2.44 2.43 2.42 2.39 2.38 2.37 2.36 2.35 2.34 2.33 2.12 2.09
NC
CN
O
CN
NC
5
(400 MHz, CDCl3)
2.11 1.11 2.13
0.98 1.03 1.00
1.01 1.03
2.08 1.08 1.04
9.0 8.5 8.0 7.5 7.0 6.5 6.0 5.5 5.0 4.5 4.0 3.5 3.0 2.5 2.0 1.5 1.0 0.5 0.0
f1 (ppm)

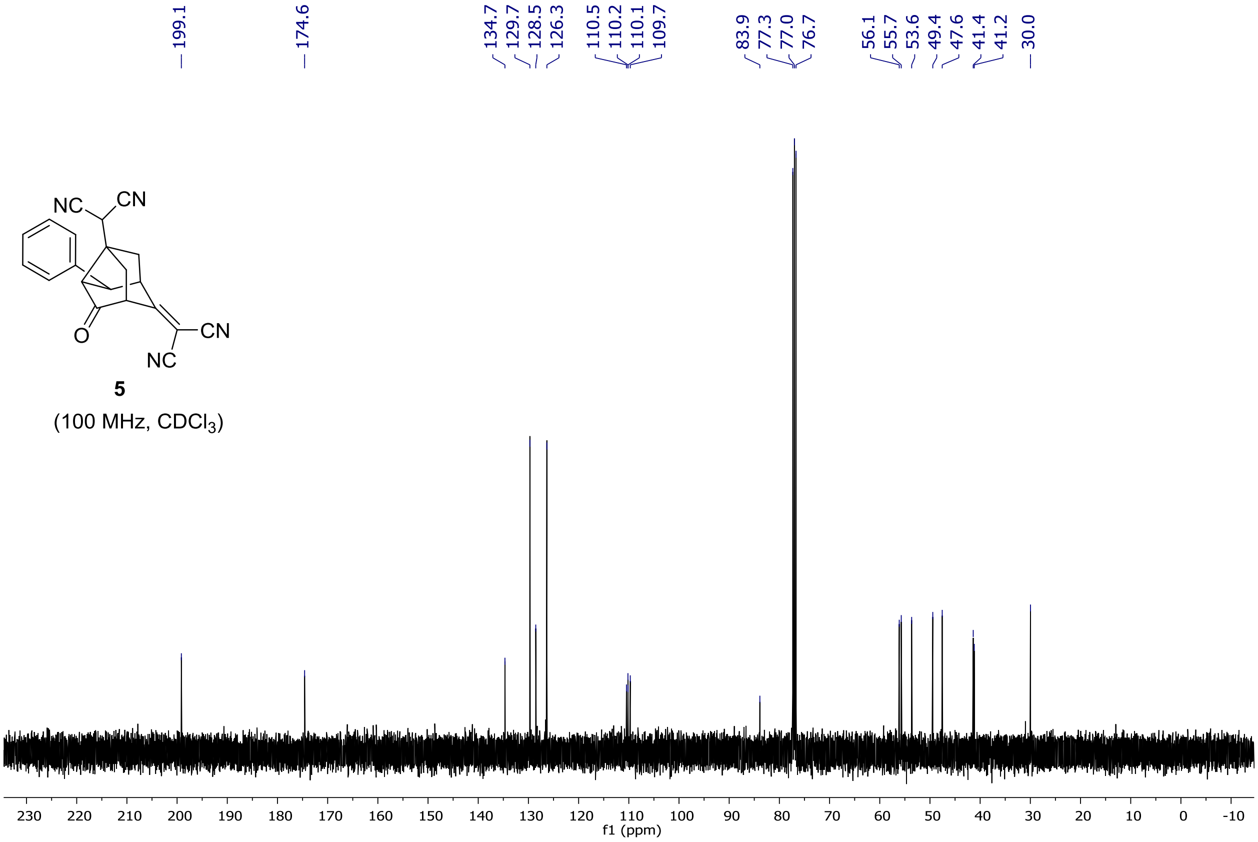
199.1
174.6
134.7 129.7 128.5 126.3
110.5 110.2 110.1 109.7
83.9 77.3 77.0 76.7
56.1 55.7 53.6 49.4 47.6 41.4 41.2 30.0
NC
CN
O
CN
NC
5
(100 MHz, CDCl3)
230 220 210 200 190 180 170 160 150 140 130 120 110 100 90 80 70 60 50 40 30 20 10 0 -10
f1 (ppm)

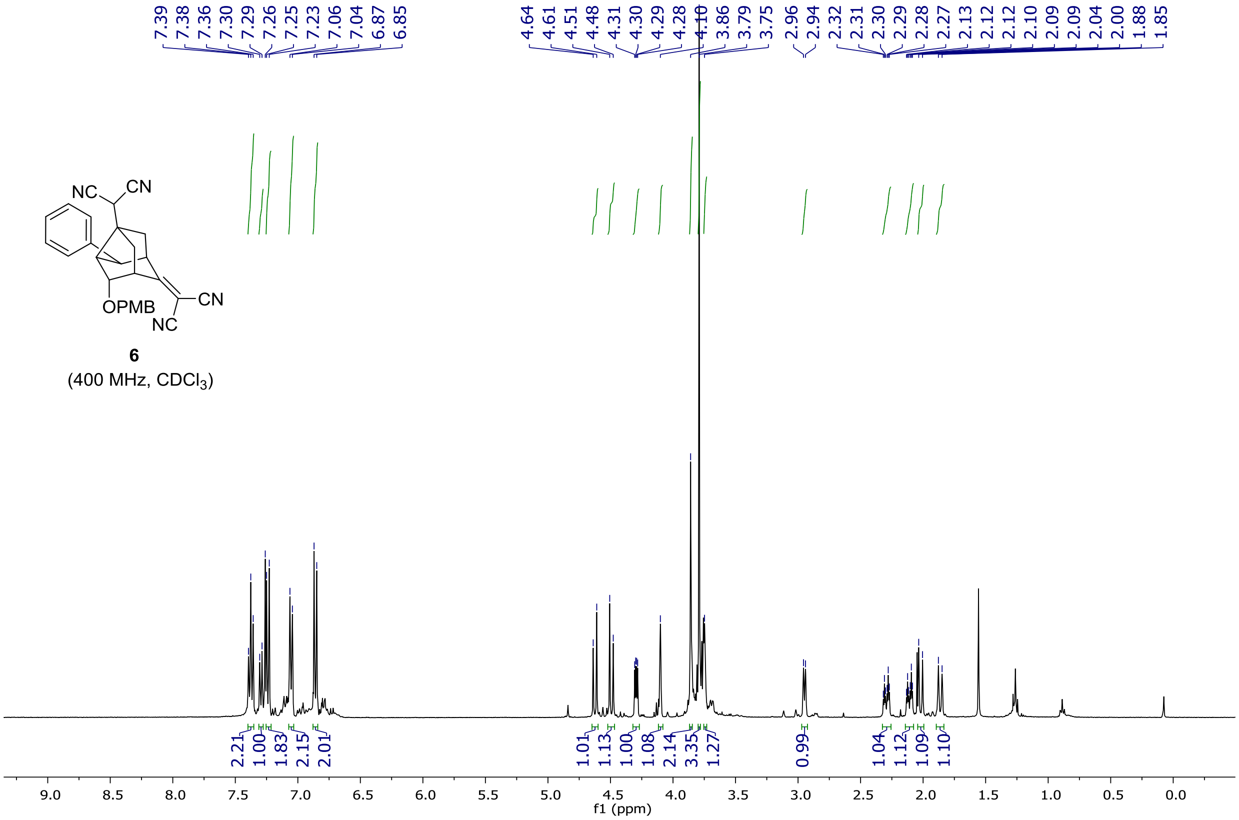

6
(400 MHz, $CDCl_3$)

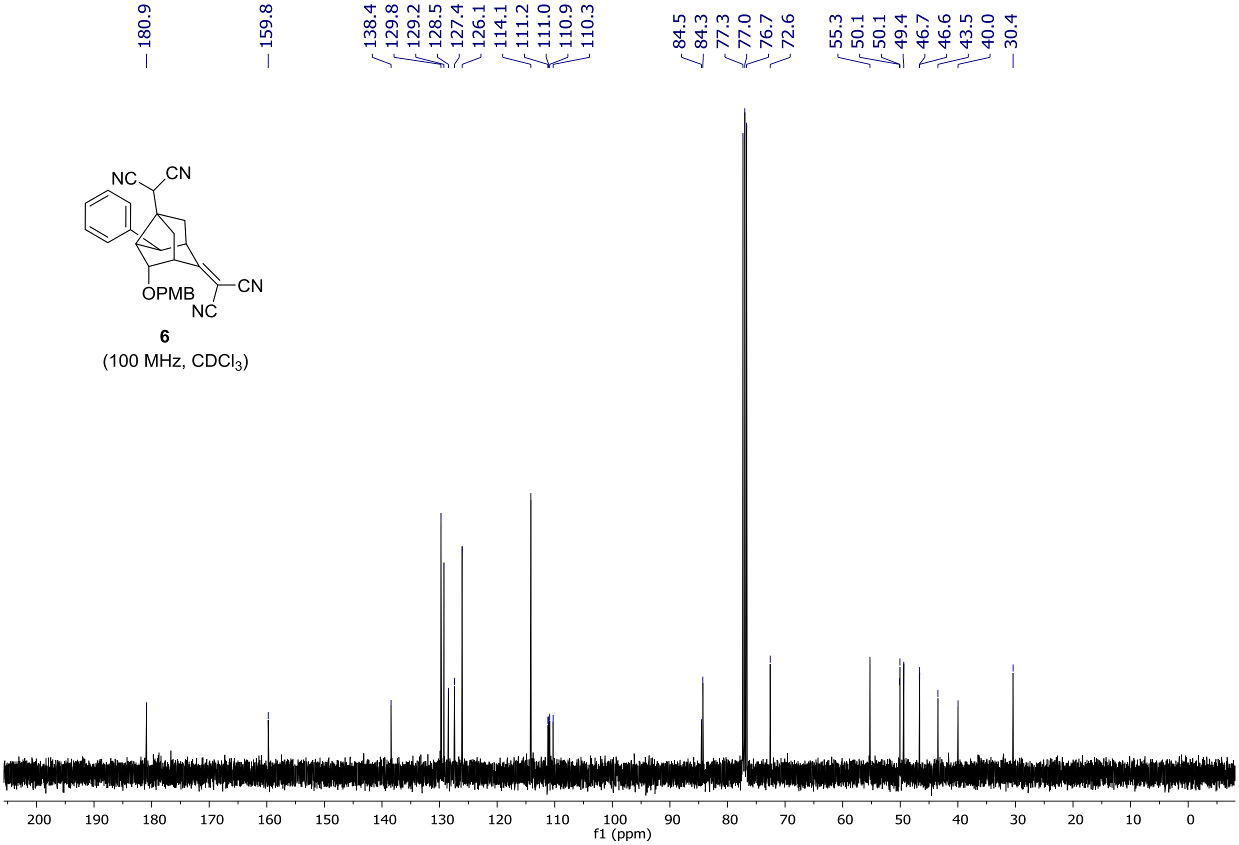

6
(100 MHz, $CDCl_3$)

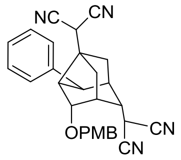

7
(400 MHz, $CDCl_3$)

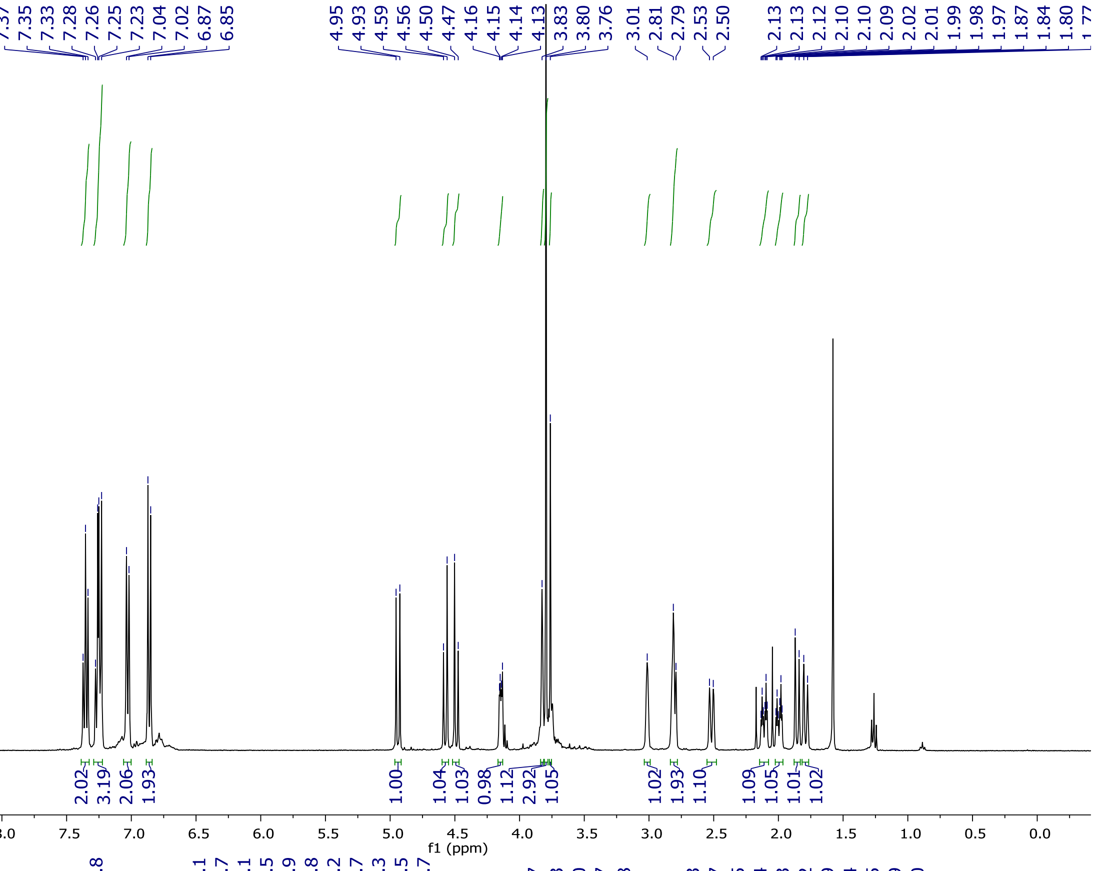

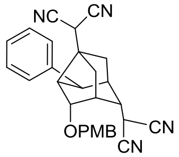

7
(100 MHz, $CDCl_3$)

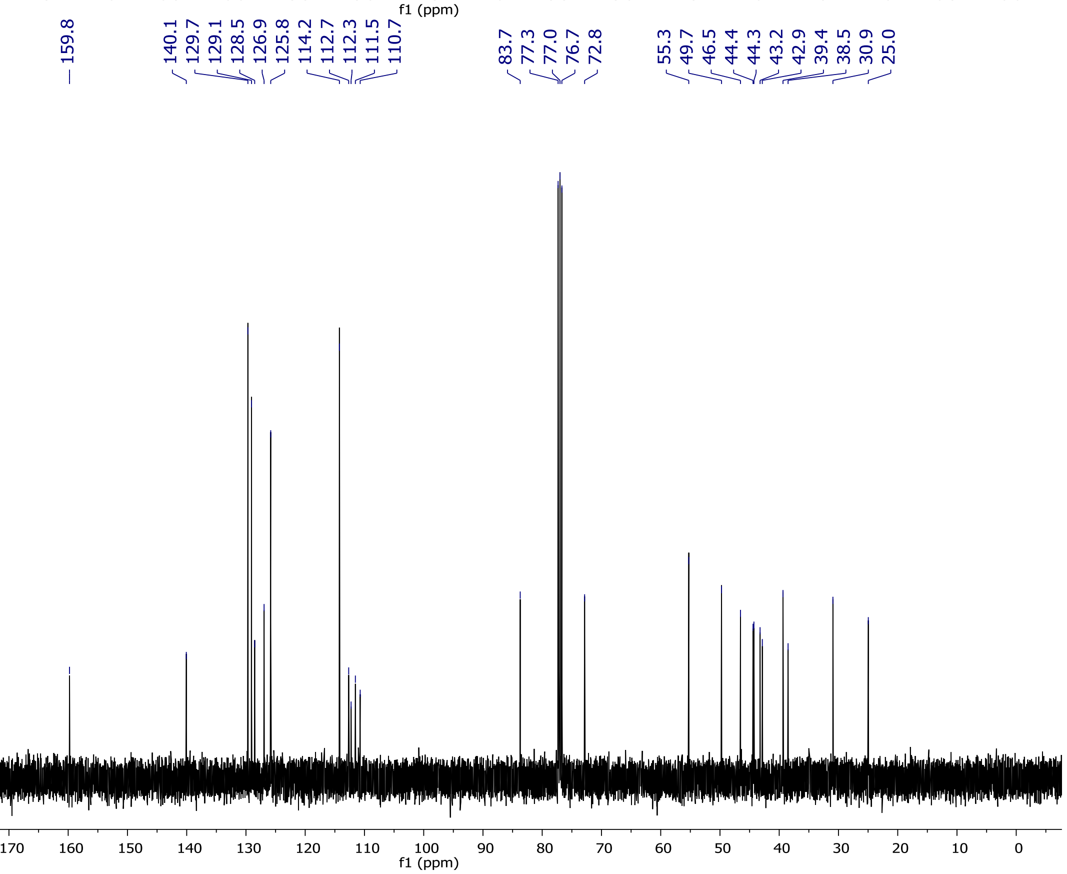

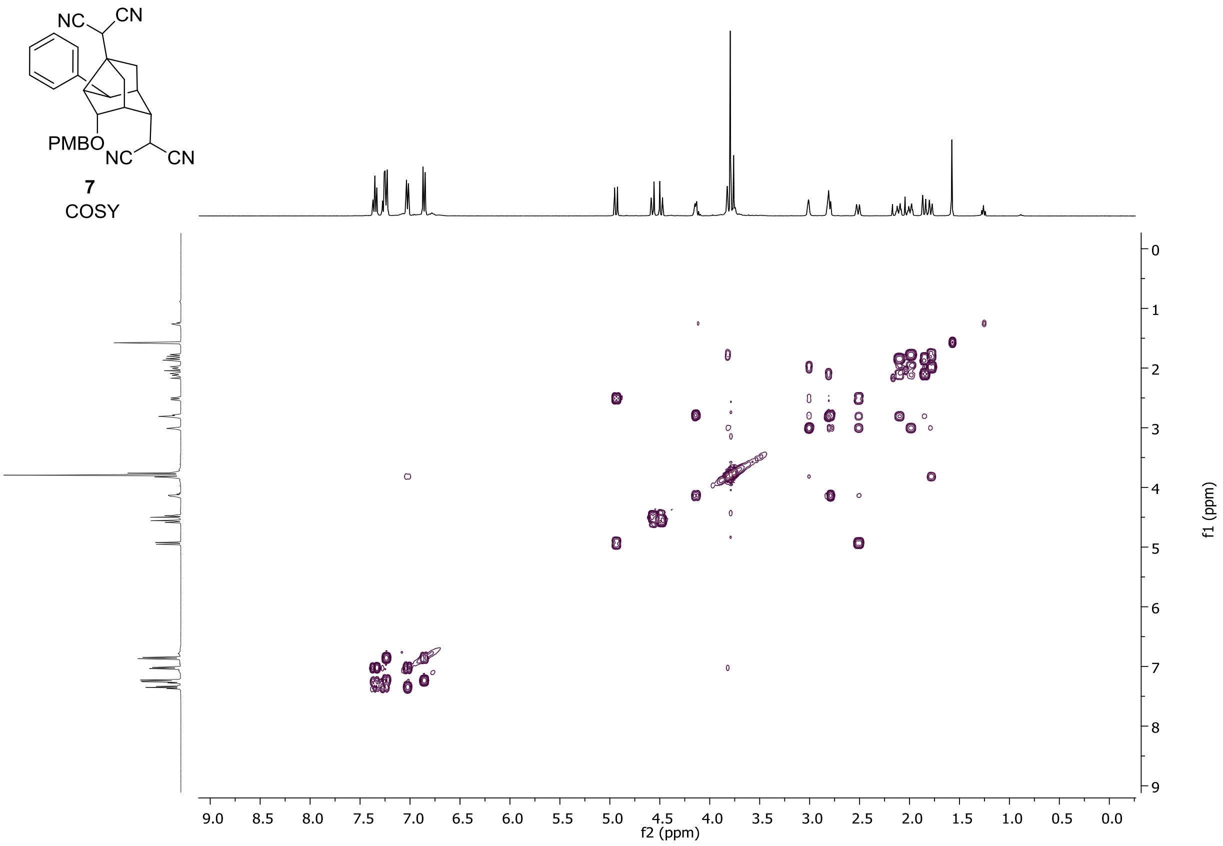
NC
CN
PMBO
NC
CN
7
COSY
f1 (ppm)
f2 (ppm)

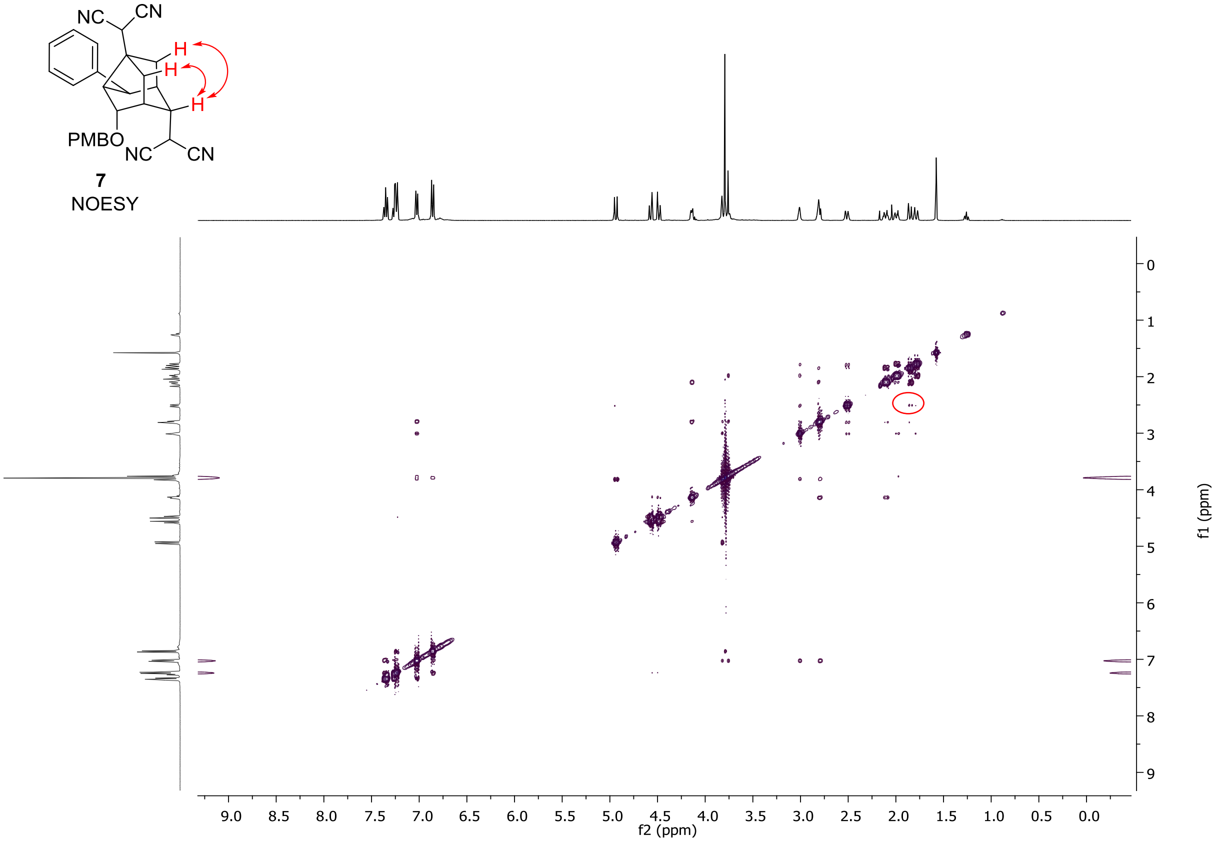
NC
CN
H
H
H
PMBO
NC
CN
7
NOESY
f1 (ppm)
f2 (ppm)

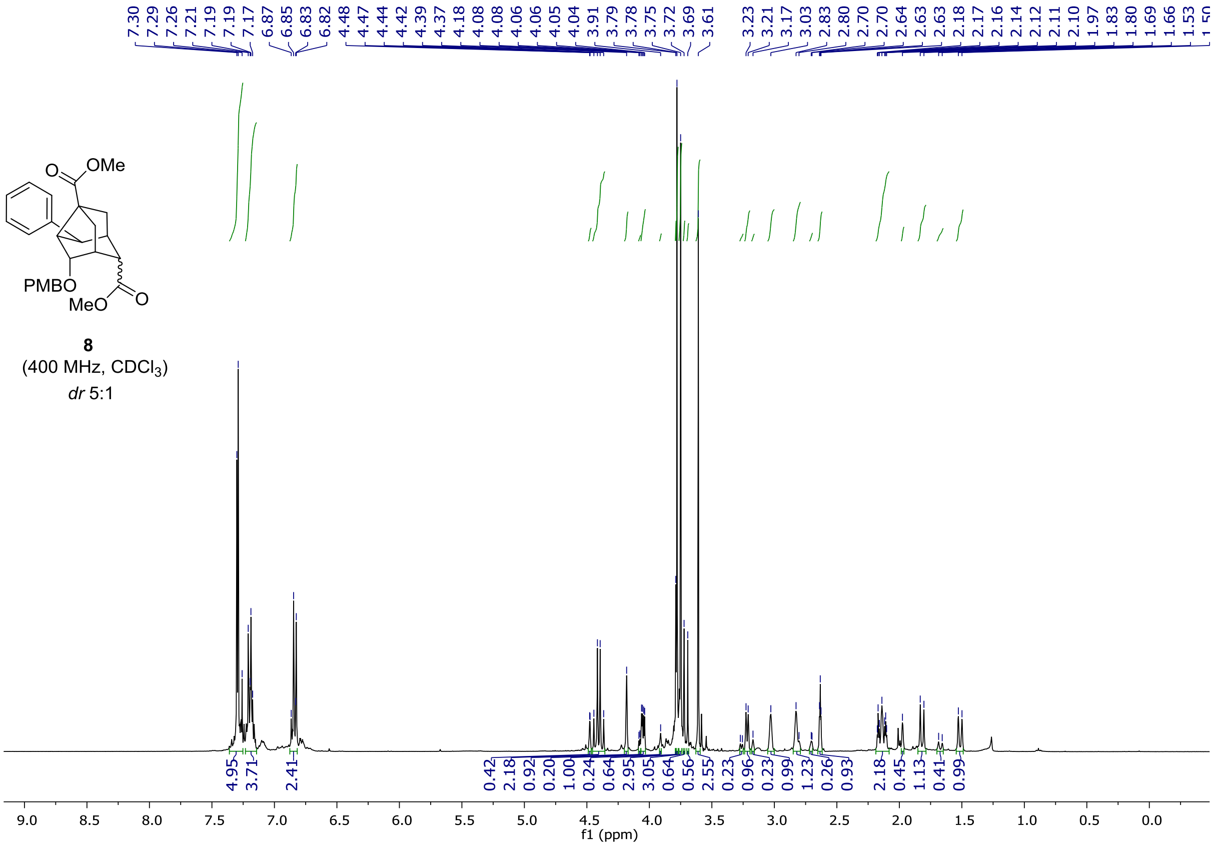

8
(400 MHz, $CDCl_3$)
dr 5:1

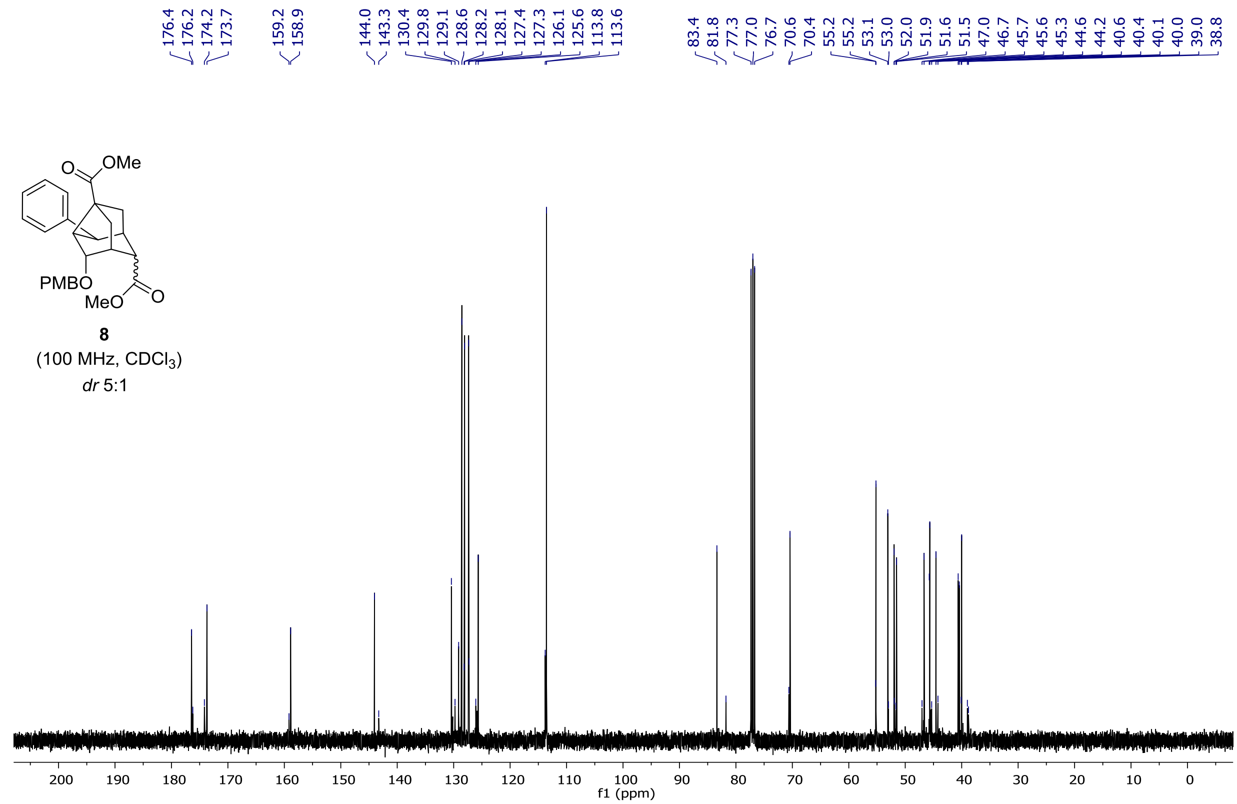

8
(100 MHz, $CDCl_3$)
dr 5:1

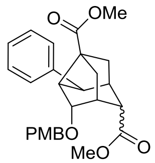

8

COSY

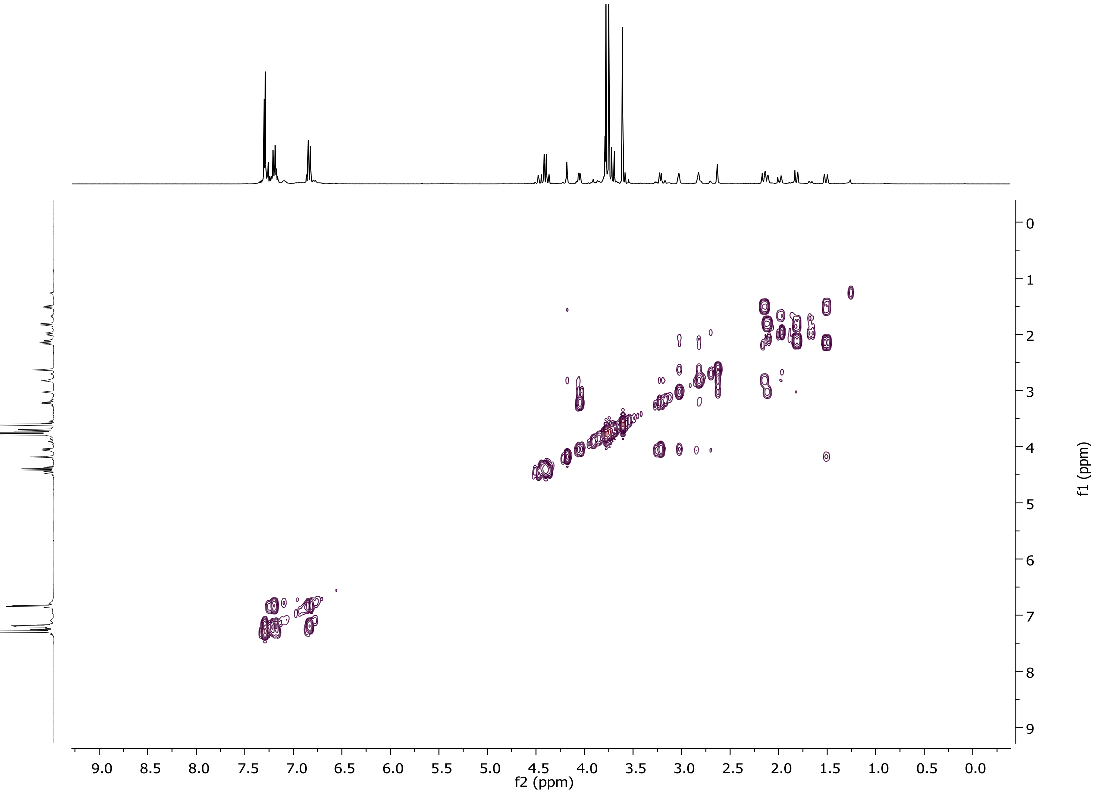

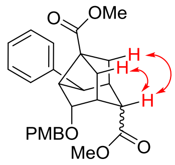

8

NOESY

(for major)

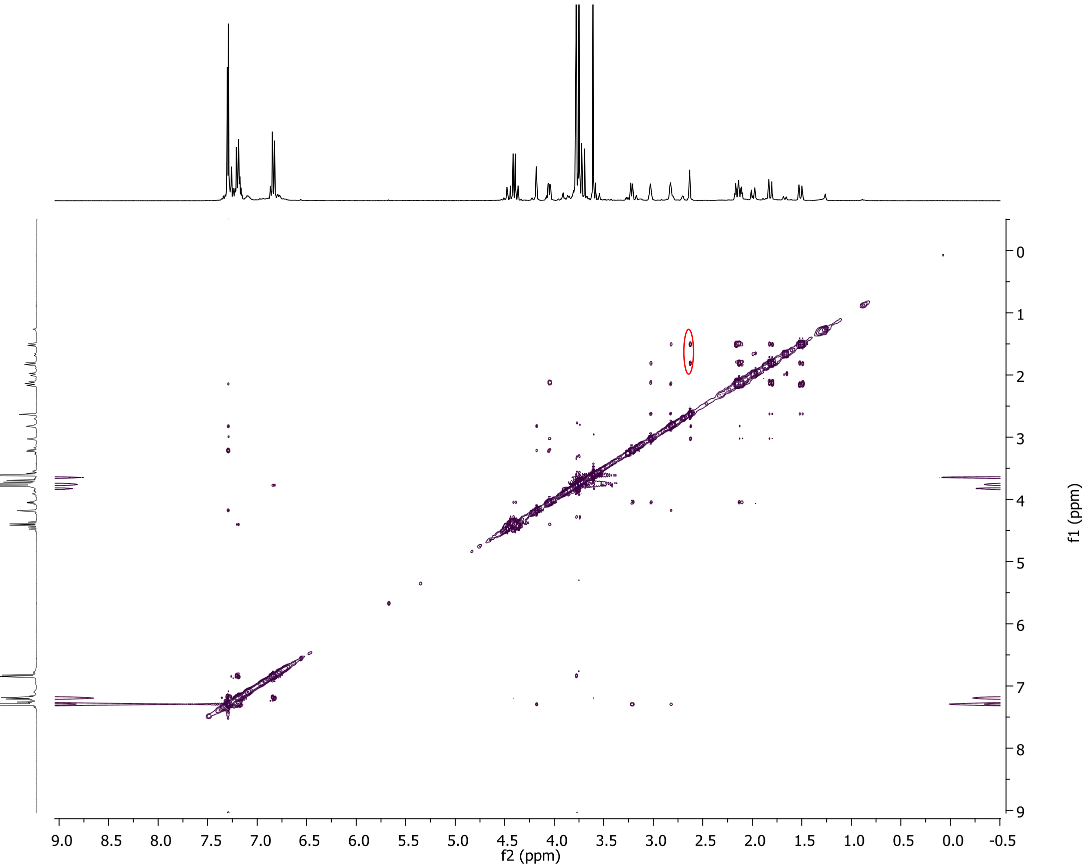

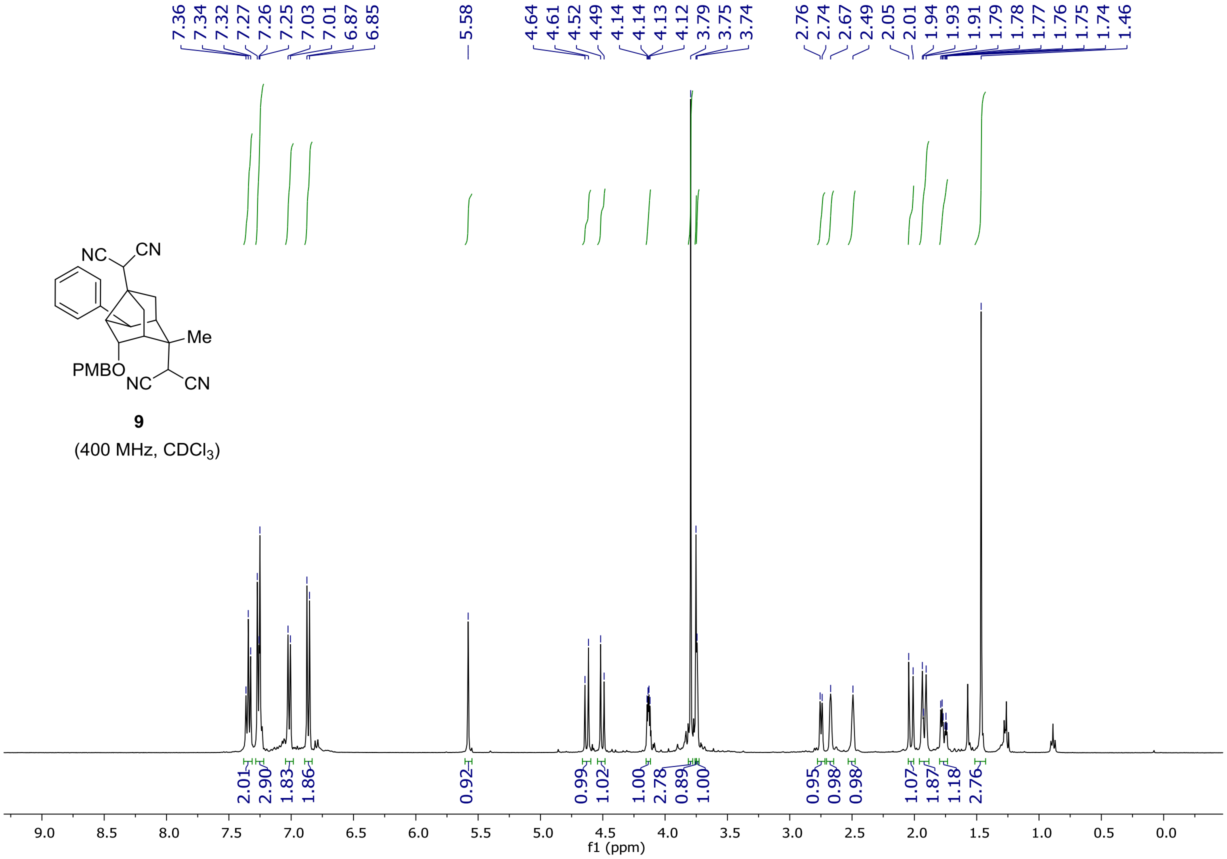
7.36 7.34 7.32 7.27 7.26 7.25 7.03 7.01 6.87 6.85
5.58
4.64 4.61 4.52 4.49 4.14 4.14 4.13 4.12 3.79 3.75 3.74
2.76 2.74 2.67 2.49 2.05 2.01 1.94 1.93 1.91 1.79 1.78 1.77 1.76 1.75 1.74 1.46
NC CN
Me
PMBO
NC CN
9
(400 MHz, CDCl3)
2.01 2.90 1.83 1.86 0.92 0.99 1.02 1.00 2.78 0.89 1.00 0.95 0.98 0.98 1.07 1.87 1.18 2.76
f1 (ppm)

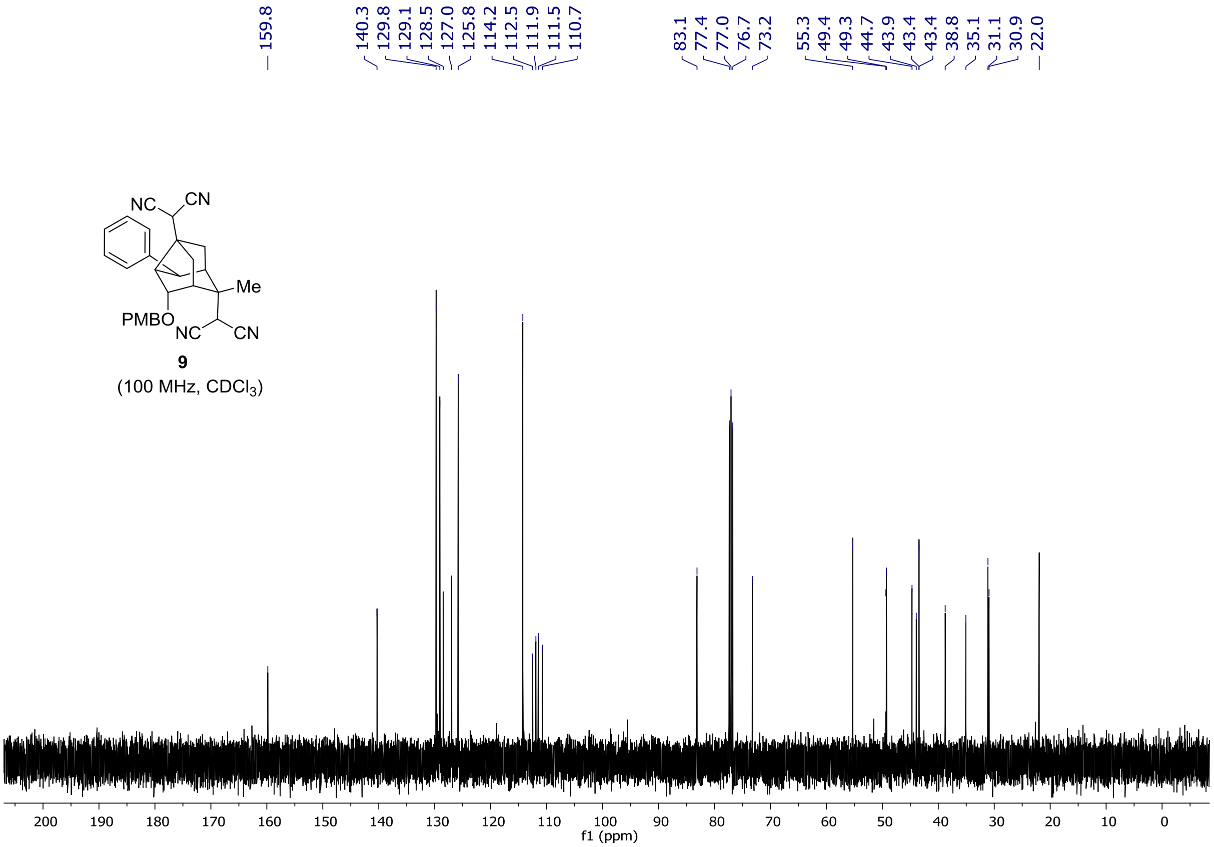
159.8
140.3 129.8 129.1 128.5 127.0 125.8 114.2 112.5 111.9 111.5 110.7
83.1 77.4 77.0 76.7 73.2
55.3 49.4 49.3 44.7 43.9 43.4 43.4 38.8 35.1 31.1 30.9 22.0
NC CN
Me
PMBO
NC CN
9
(100 MHz, CDCl3)
f1 (ppm)

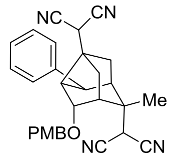

9
COSY

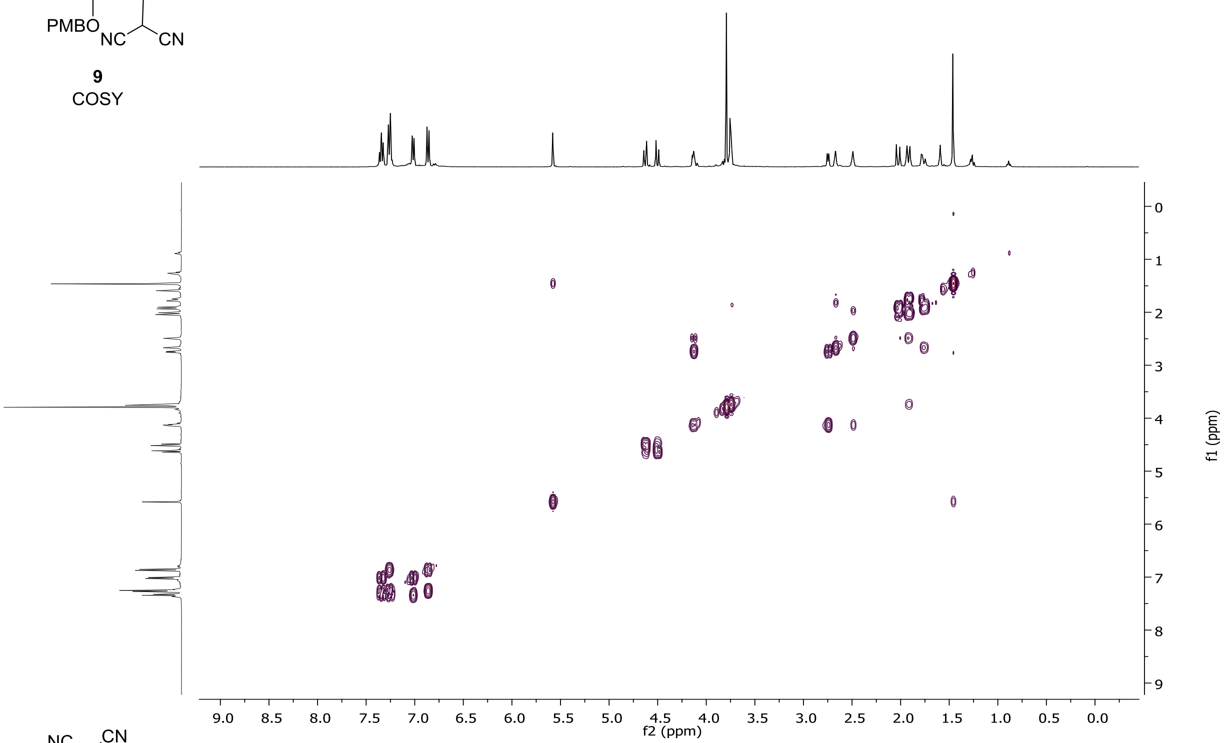

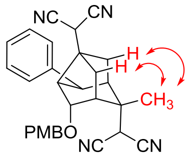

9
NOESY

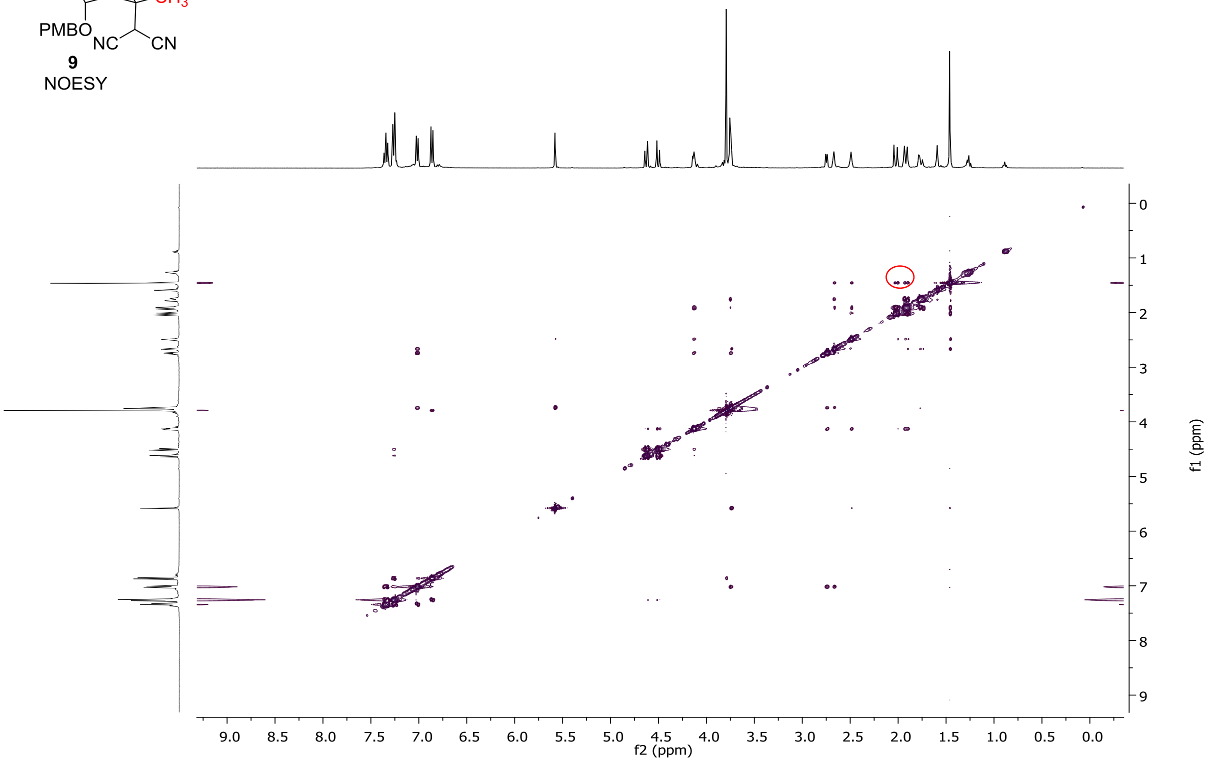

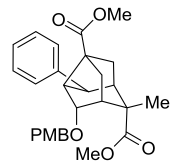

10
(400 MHz, $CDCl_3$)

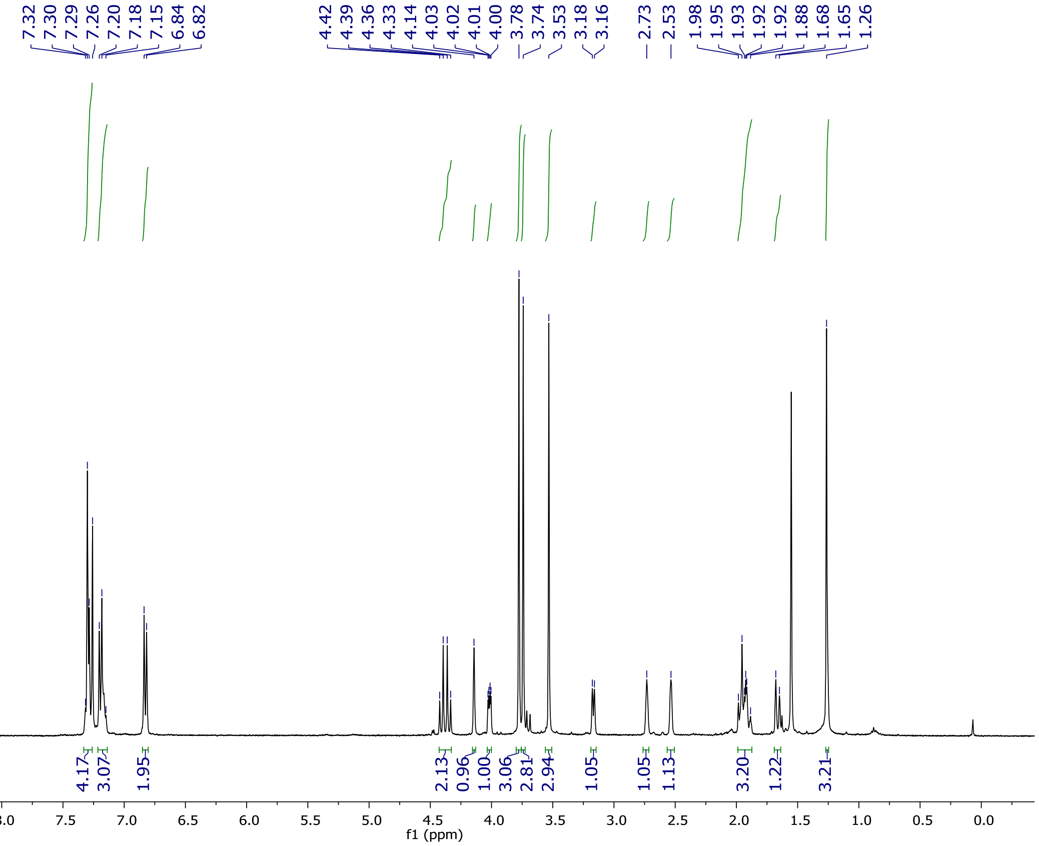

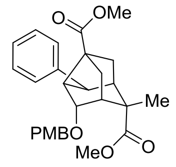

10
(100 MHz, $CDCl_3$)

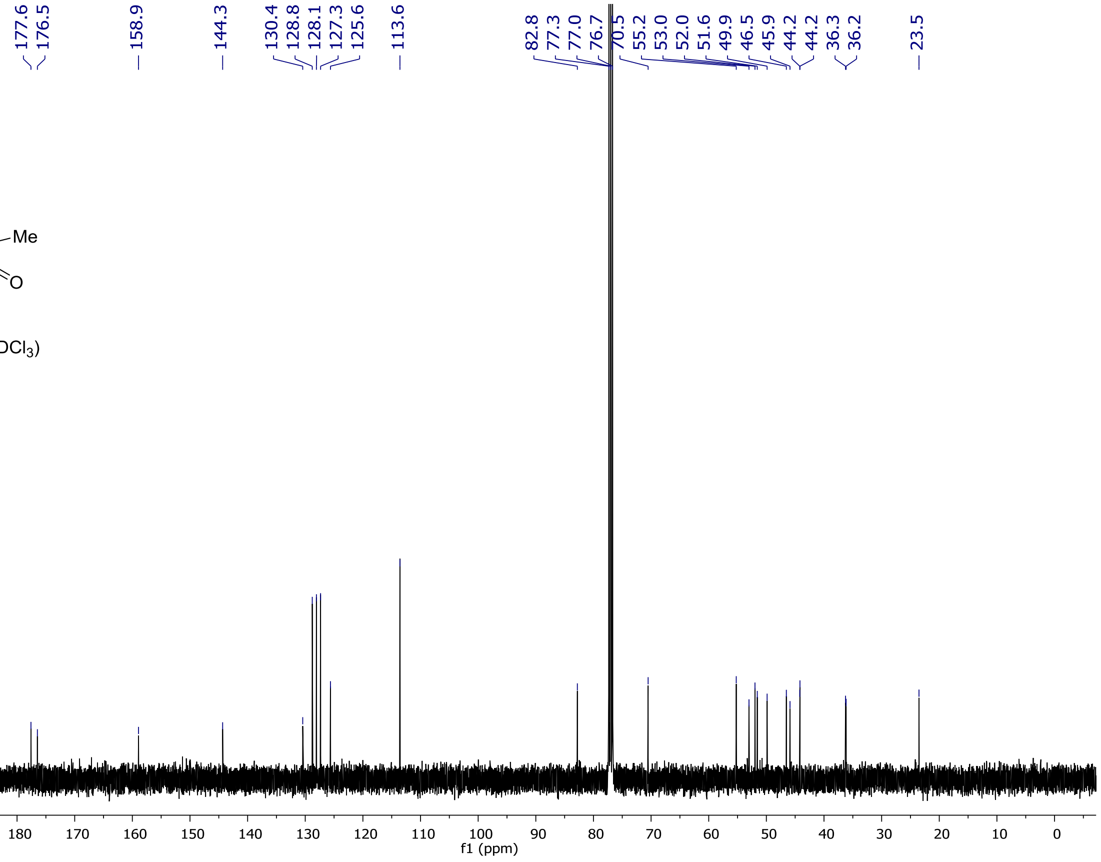

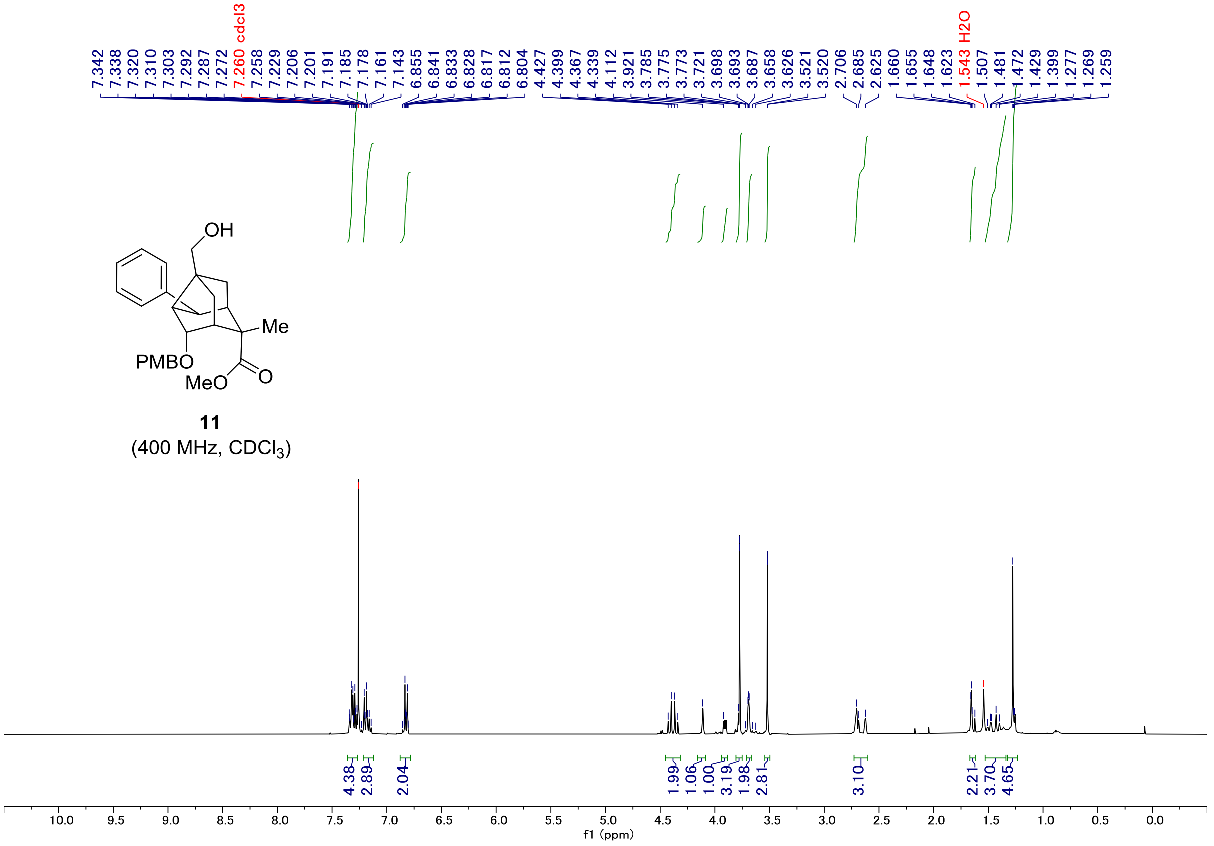
7.342 7.338 7.320 7.310 7.303 7.292 7.287 7.272 7.260 cdcl3 7.258 7.229 7.206 7.201 7.191 7.185 7.178 7.161 7.143 6.855 6.841 6.833 6.828 6.817 6.812 6.804 4.427 4.399 4.367 4.339 4.112 3.921 3.785 3.775 3.773 3.721 3.698 3.693 3.687 3.658 3.626 3.521 3.520 2.706 2.685 2.625 1.660 1.655 1.648 1.623 1.543 H2O 1.507 1.481 1.472 1.429 1.399 1.277 1.269 1.259
OH
Me
PMBO
MeO
O
11
(400 MHz, CDCl3)
4.38 2.89 2.04 1.99 1.06 1.00 3.19 1.98 2.81 3.10 2.21 3.70 4.65
10.0 9.5 9.0 8.5 8.0 7.5 7.0 6.5 6.0 5.5 5.0 4.5 4.0 3.5 3.0 2.5 2.0 1.5 1.0 0.5 0.0
f1 (ppm)

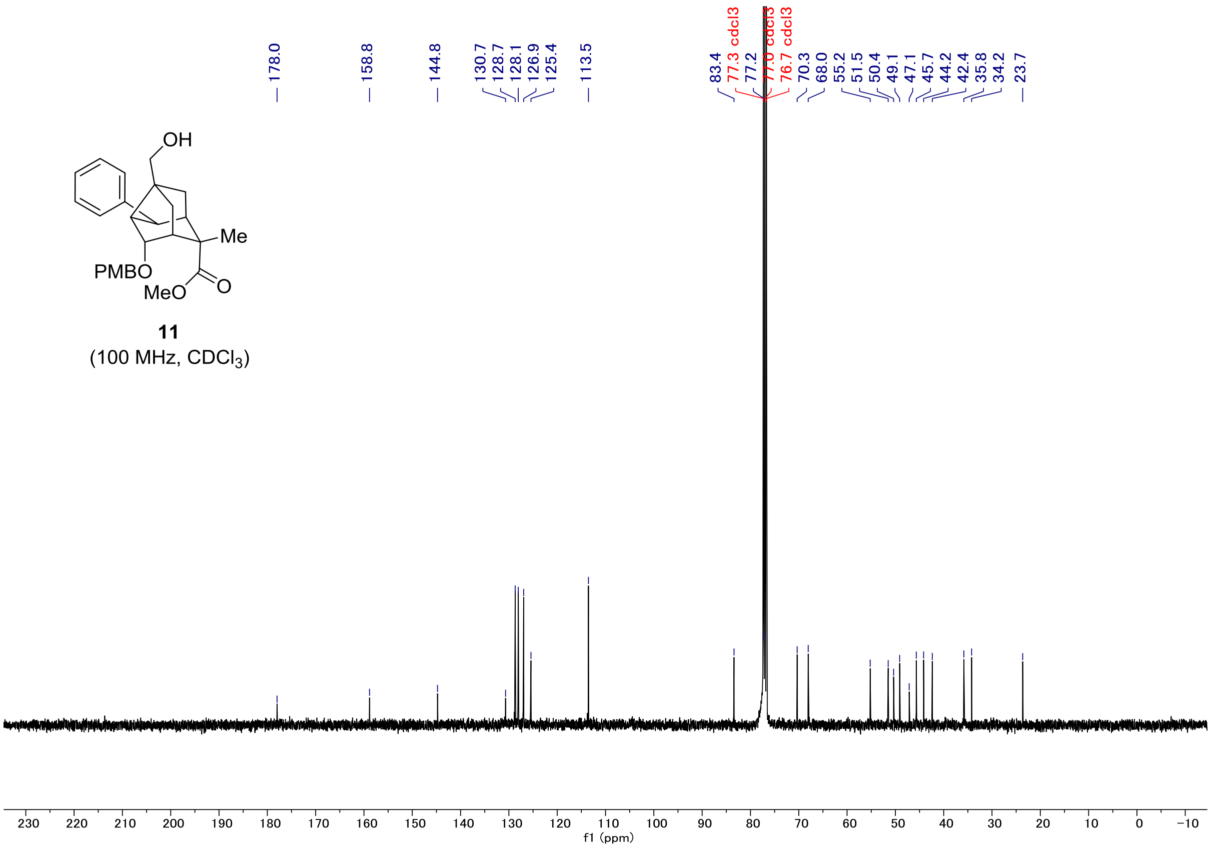
178.0 158.8 144.8 130.7 128.7 128.1 126.9 125.4 113.5 83.4 77.3 cdcl3 77.2 77.0 cdcl3 76.7 cdcl3 70.3 68.0 55.2 51.5 50.4 49.1 47.1 45.7 44.2 42.4 35.8 34.2 23.7
OH
Me
PMBO
MeO
O
11
(100 MHz, CDCl3)
230 220 210 200 190 180 170 160 150 140 130 120 110 100 90 80 70 60 50 40 30 20 10 0 −10
f1 (ppm)

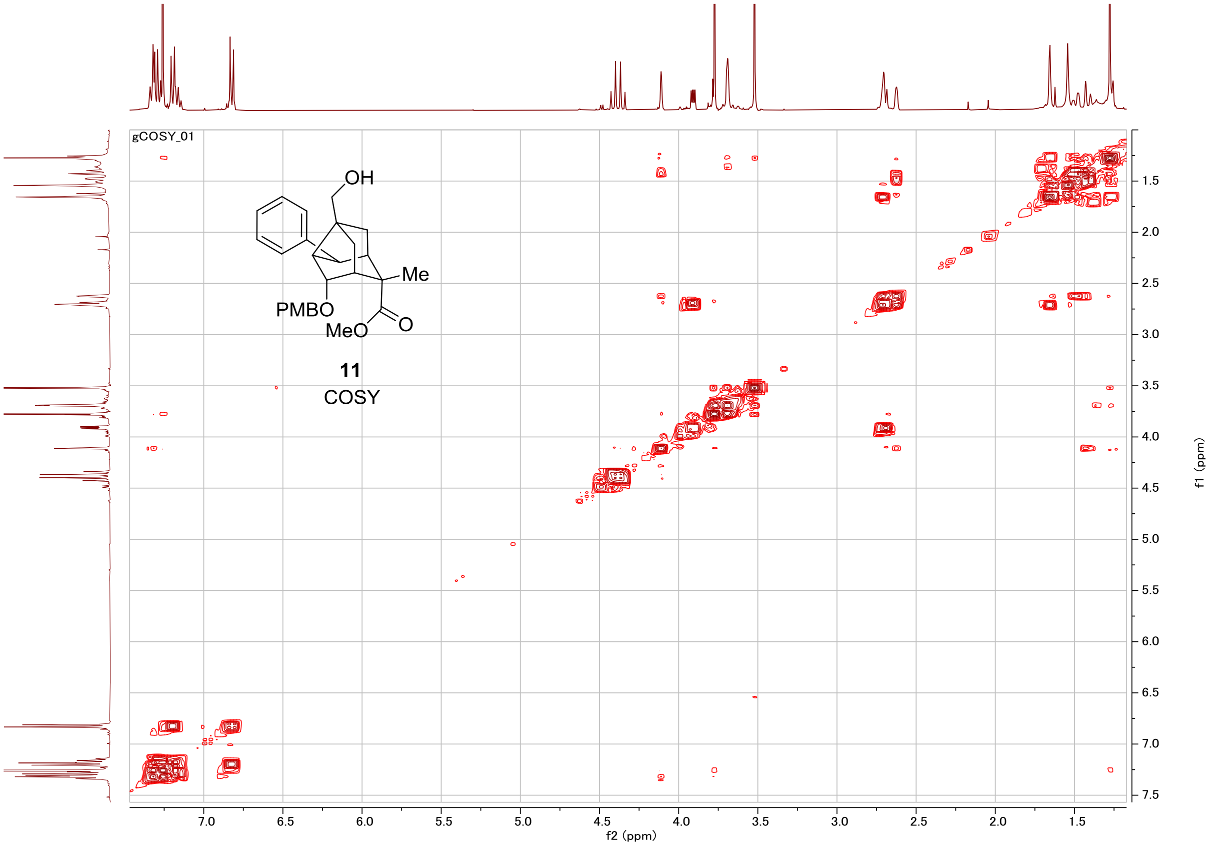
gCOSY_01
OH
Me
PMBO
MeO
O
11
COSY
f2 (ppm)
f1 (ppm)

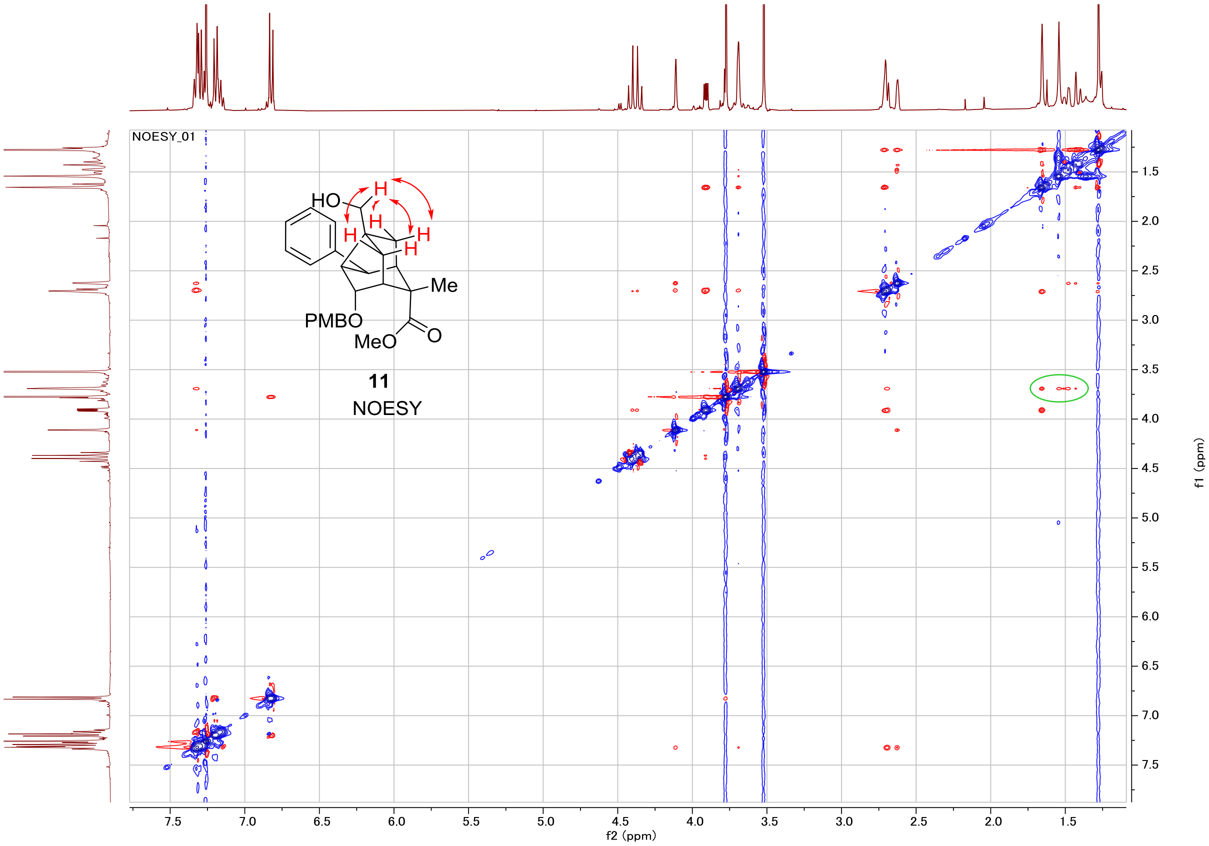
NOESY_01
HO
H
Me
PMBO
MeO
O
11
NOESY
f2 (ppm)
f1 (ppm)

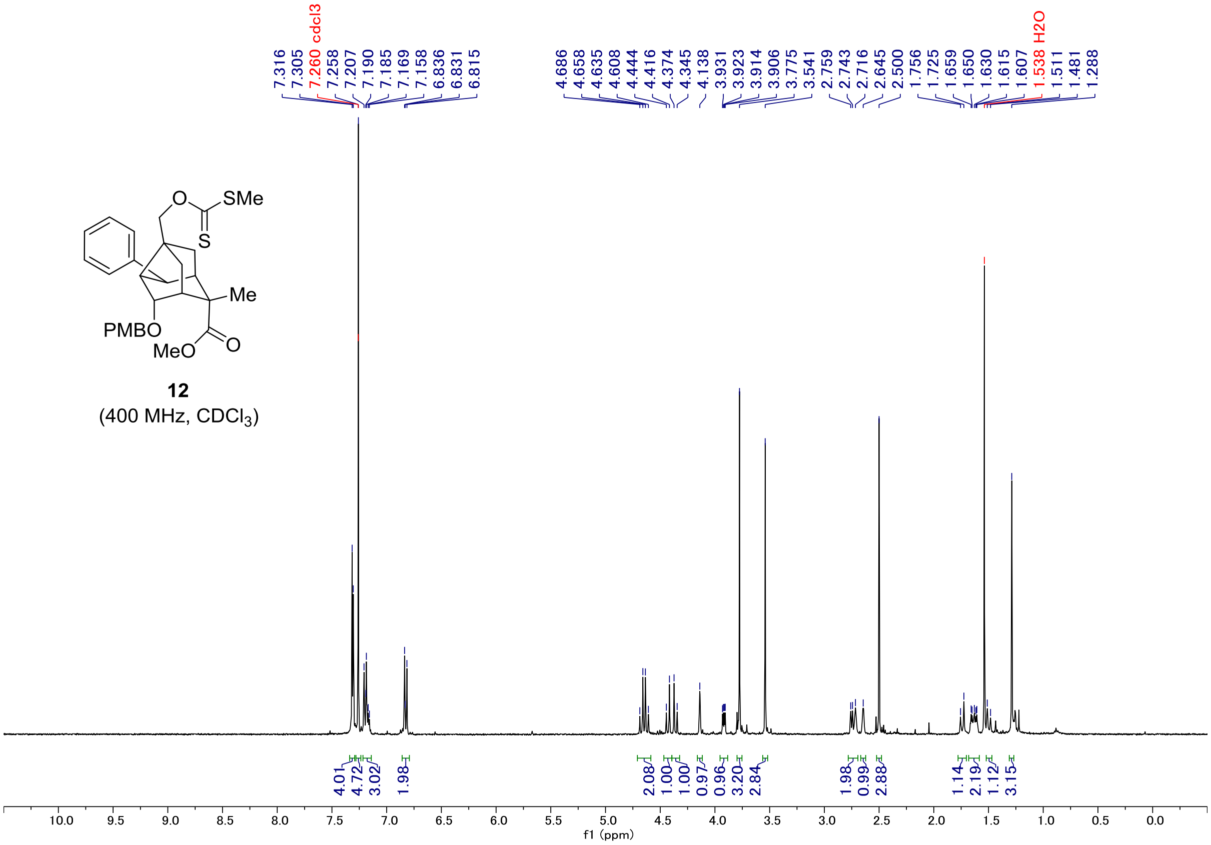

12
(400 MHz, $CDCl_3$)

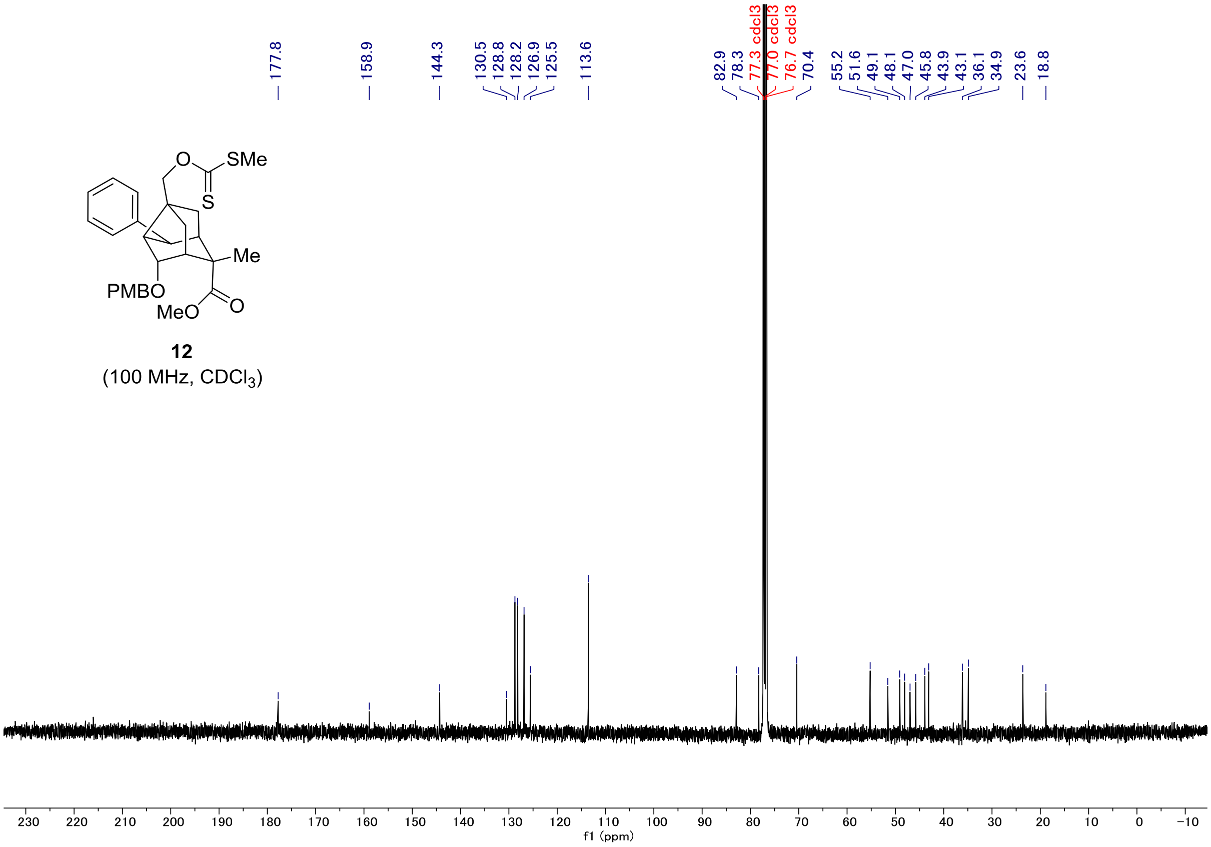

12
(100 MHz, $CDCl_3$)

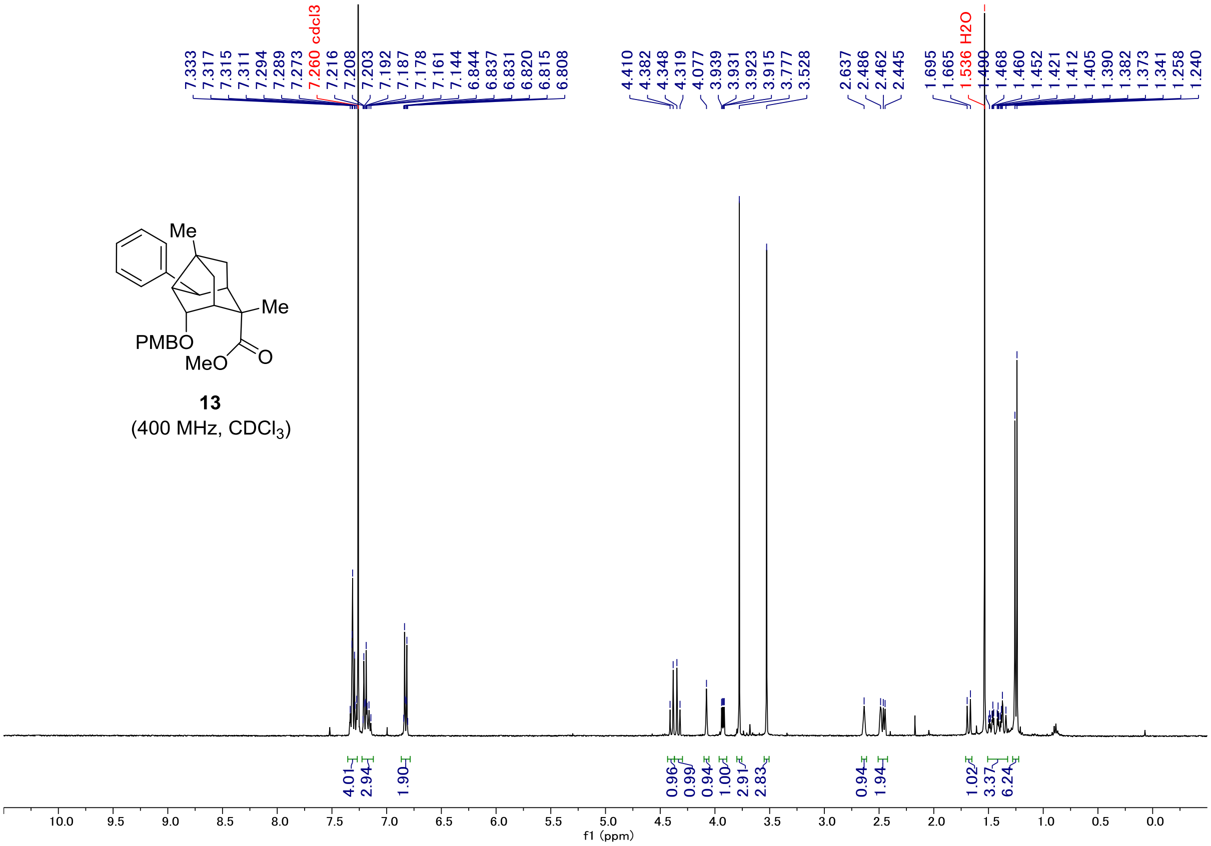

13
(400 MHz, $CDCl_3$)

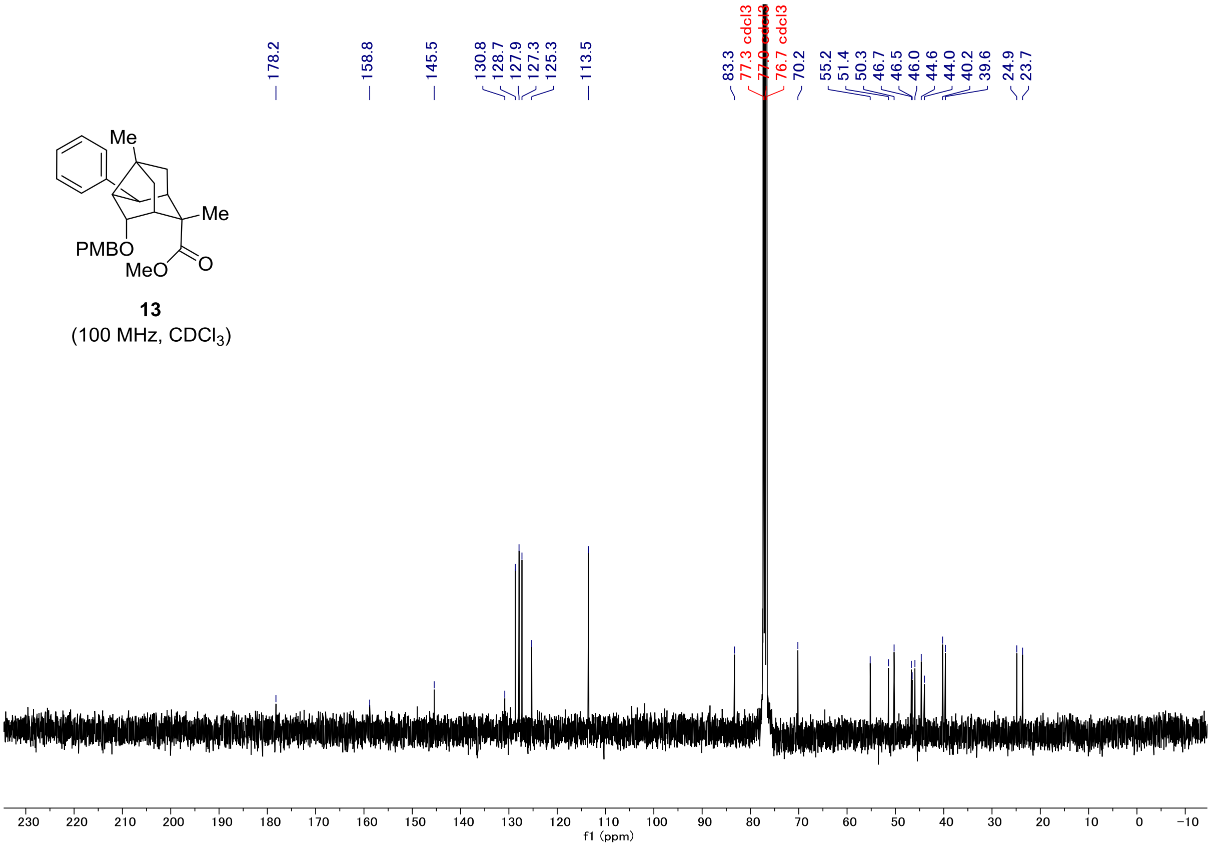

13
(100 MHz, $CDCl_3$)

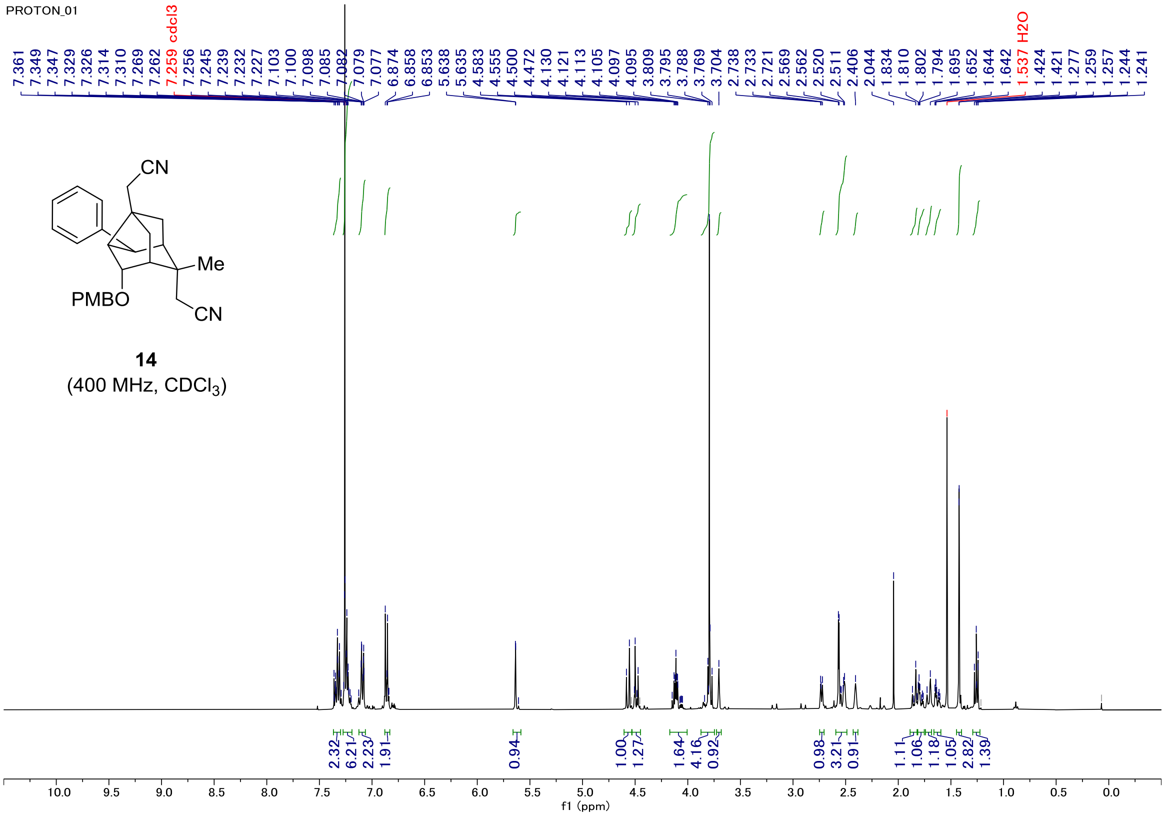
PROTON_01
7.361 7.349 7.347 7.329 7.326 7.314 7.310 7.269 7.262 7.259 cdcl3 7.256 7.245 7.239 7.232 7.227 7.103 7.100 7.098 7.085 7.082 7.079 7.077 6.874 6.858 6.853 5.638 5.635 4.583 4.555 4.500 4.472 4.130 4.121 4.113 4.105 4.097 4.095 3.809 3.795 3.788 3.769 3.704 2.738 2.733 2.721 2.569 2.562 2.520 2.511 2.406 2.044 1.834 1.810 1.802 1.794 1.695 1.652 1.644 1.642 1.537 H2O 1.424 1.421 1.277 1.259 1.257 1.244 1.241
CN
Me
PMBO
CN
14
(400 MHz, CDCl3)
2.32 6.21 2.23 1.91 0.94 1.00 1.27 1.64 4.16 0.92 0.98 3.21 0.91 1.11 1.06 1.18 1.05 2.82 1.39
f1 (ppm)

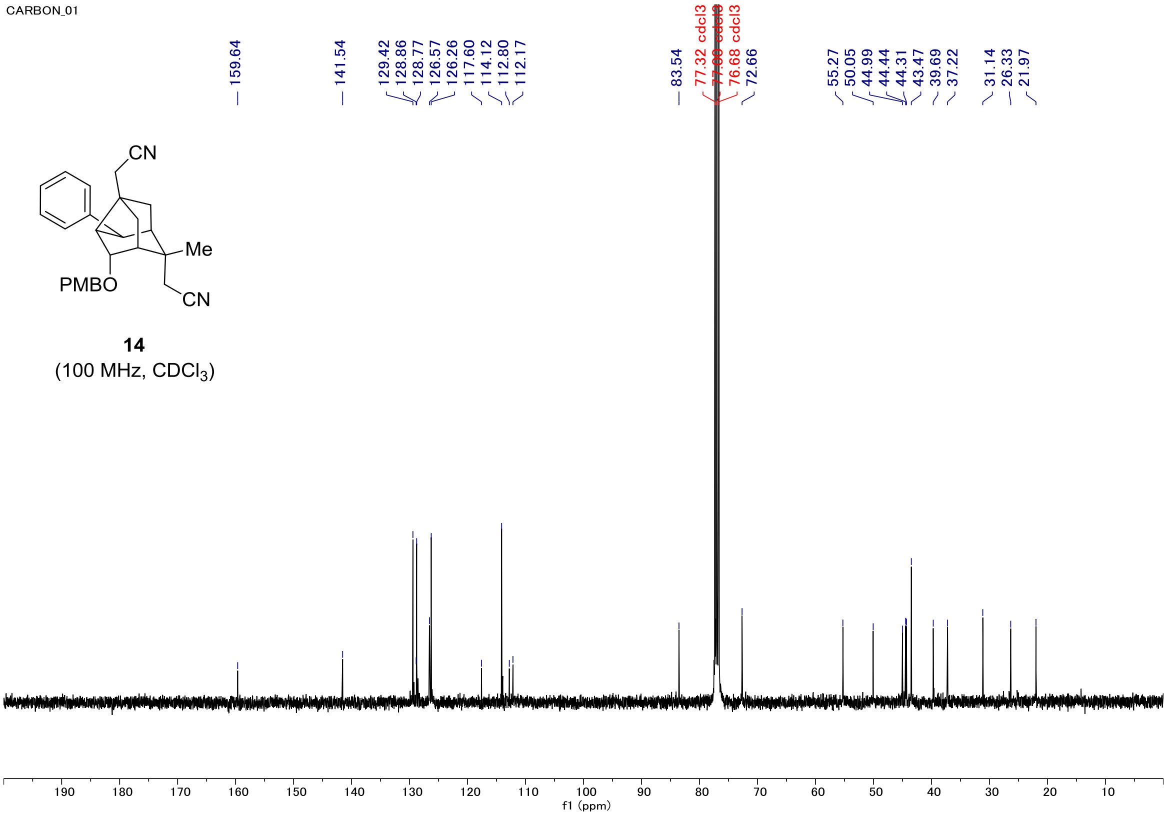
CARBON_01
159.64 141.54 129.42 128.86 128.77 126.57 126.26 117.60 114.12 112.80 112.17 83.54 77.32 cdcl3 77.00 cdcl3 76.68 cdcl3 72.66 55.27 50.05 44.99 44.44 44.31 43.47 39.69 37.22 31.14 26.33 21.97
CN
Me
PMBO
CN
14
(100 MHz, CDCl3)
f1 (ppm)

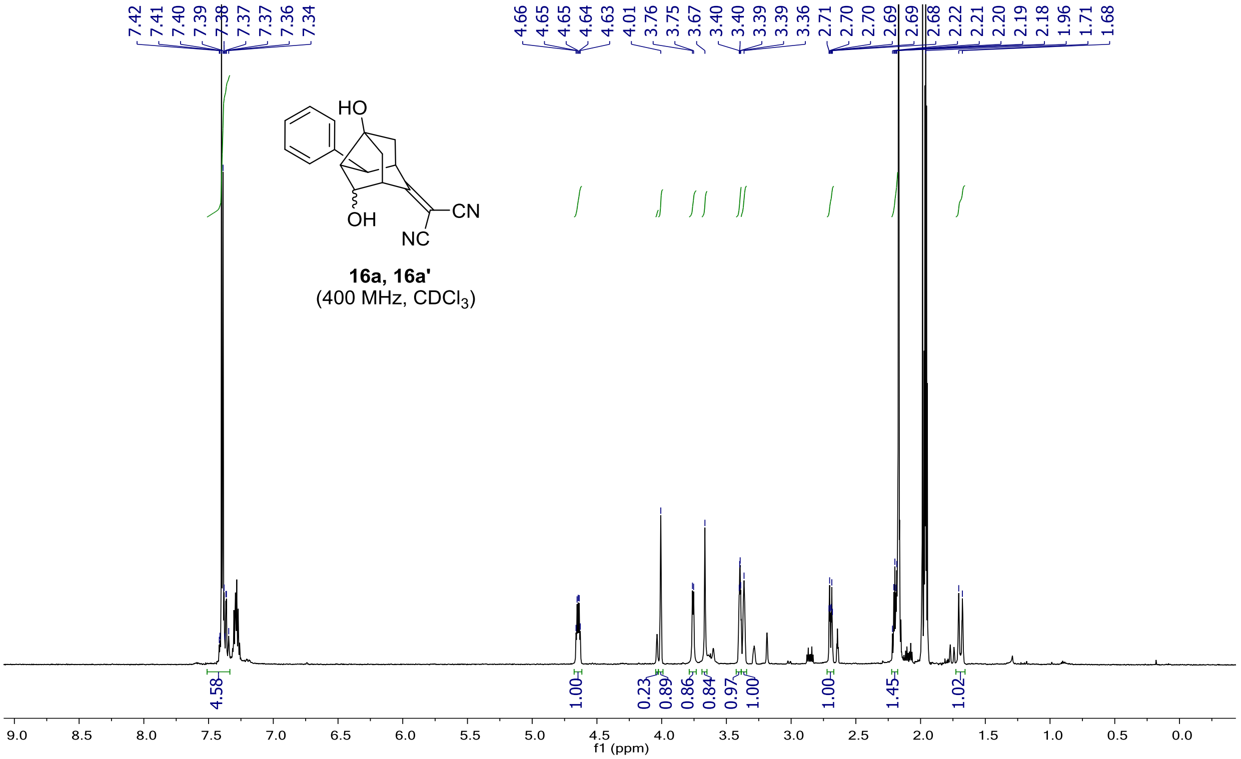
7.42 7.41 7.40 7.39 7.38 7.37 7.37 7.36 7.34
4.66 4.65 4.65 4.64 4.63 4.01 3.76 3.75 3.67 3.40 3.40 3.39 3.39 3.36 2.71 2.70 2.70 2.69 2.69 2.68 2.22 2.21 2.20 2.19 2.18 1.96 1.71 1.68
HO
CN
OH
NC
16a, 16a'
(400 MHz, CDCl3)
4.58 1.00 0.23 0.89 0.86 0.84 0.97 1.00 1.00 1.45 1.02
f1 (ppm)

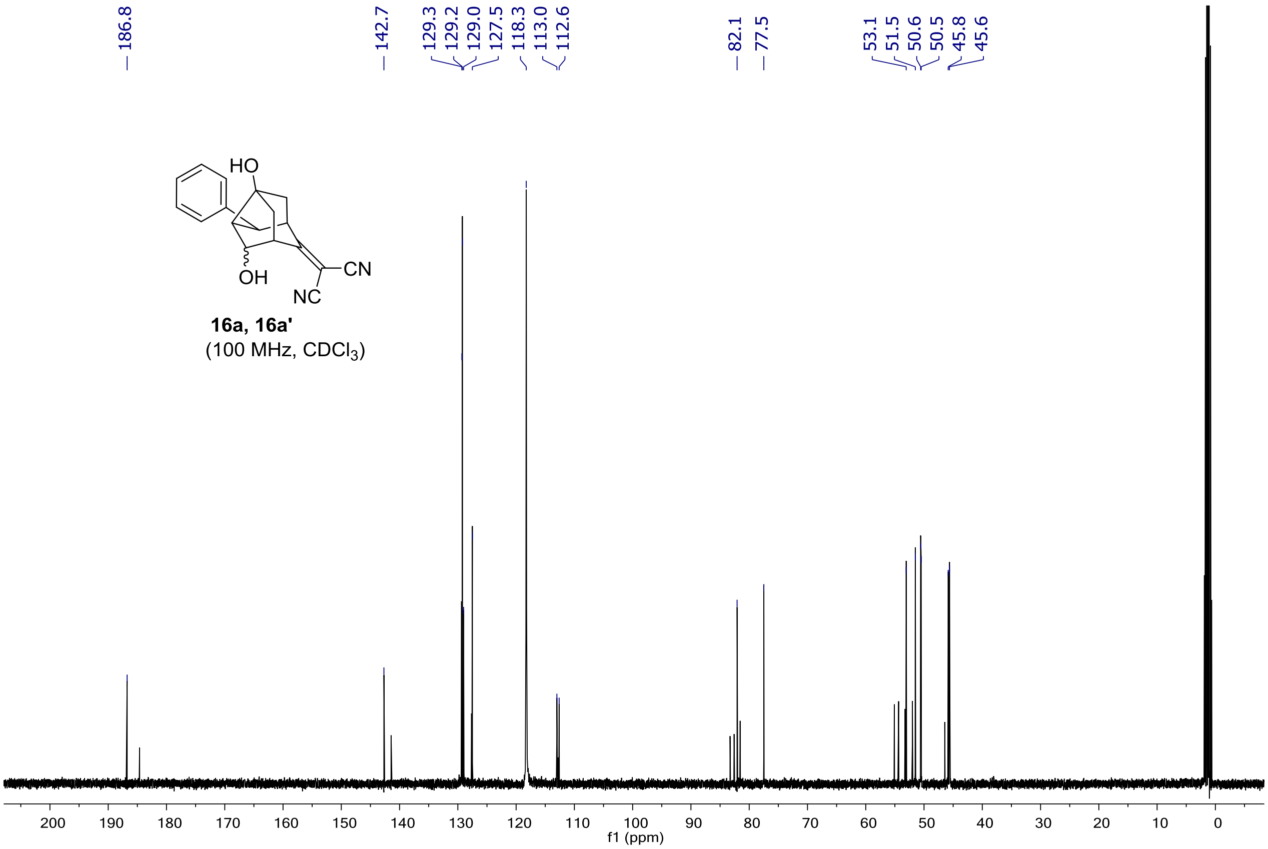
186.8
142.7
129.3 129.2 129.0 127.5 118.3 113.0 112.6
82.1 77.5
53.1 51.5 50.6 50.5 45.8 45.6
HO
CN
OH
NC
16a, 16a'
(100 MHz, CDCl3)
f1 (ppm)

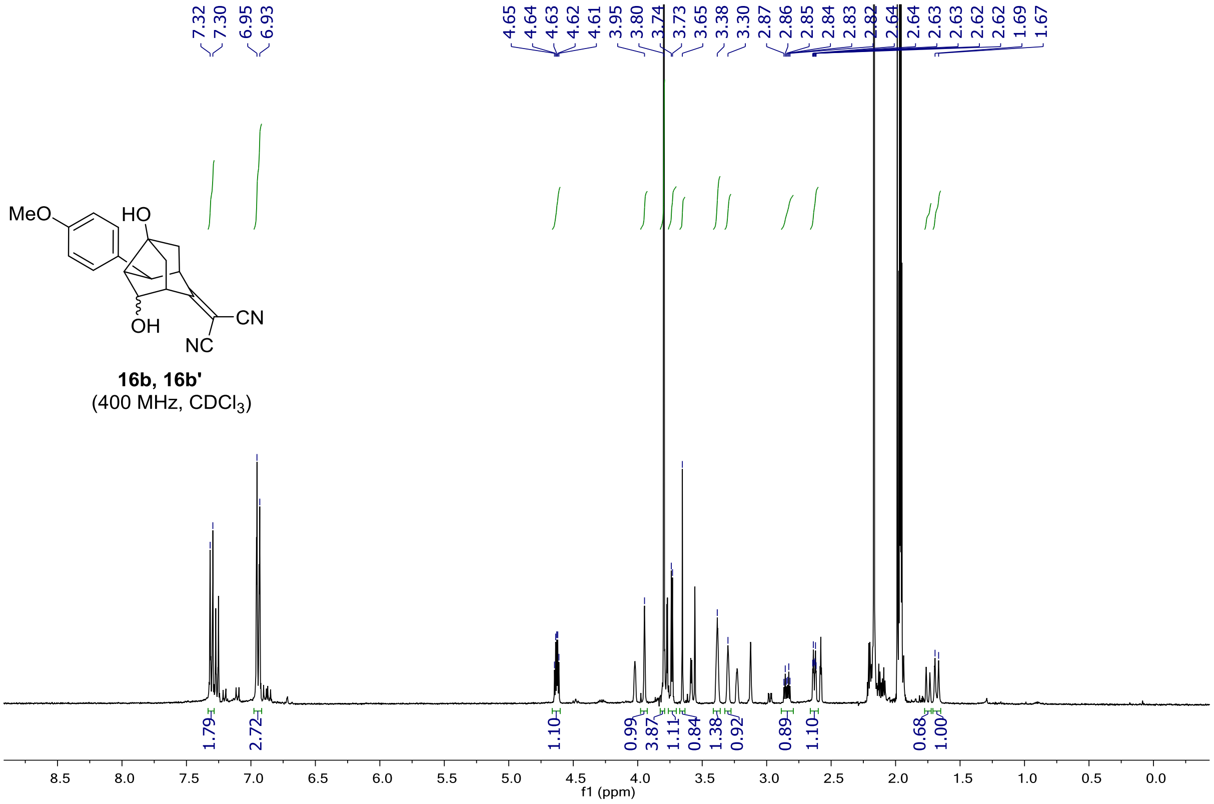

16b, 16b'
(400 MHz, $CDCl_3$)

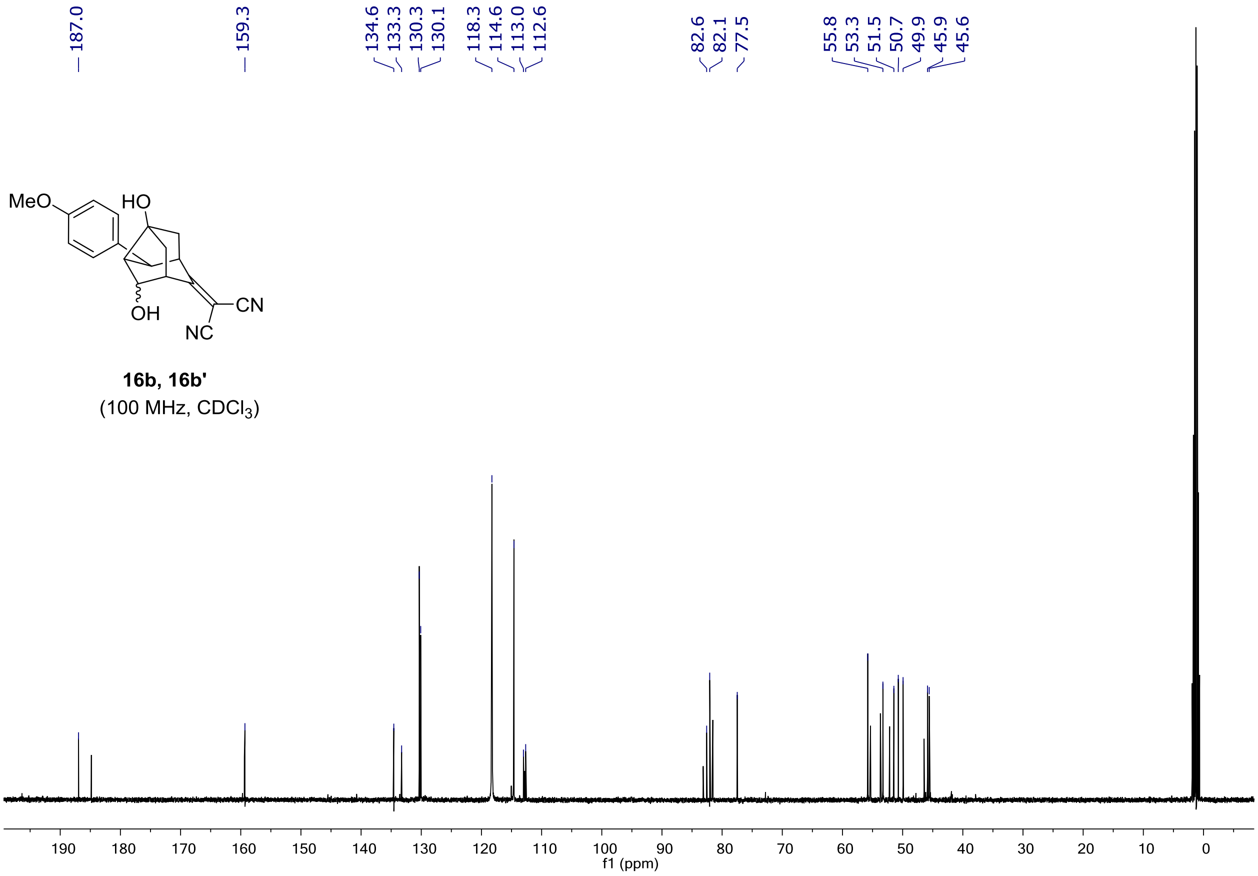

16b, 16b'
(100 MHz, $CDCl_3$)

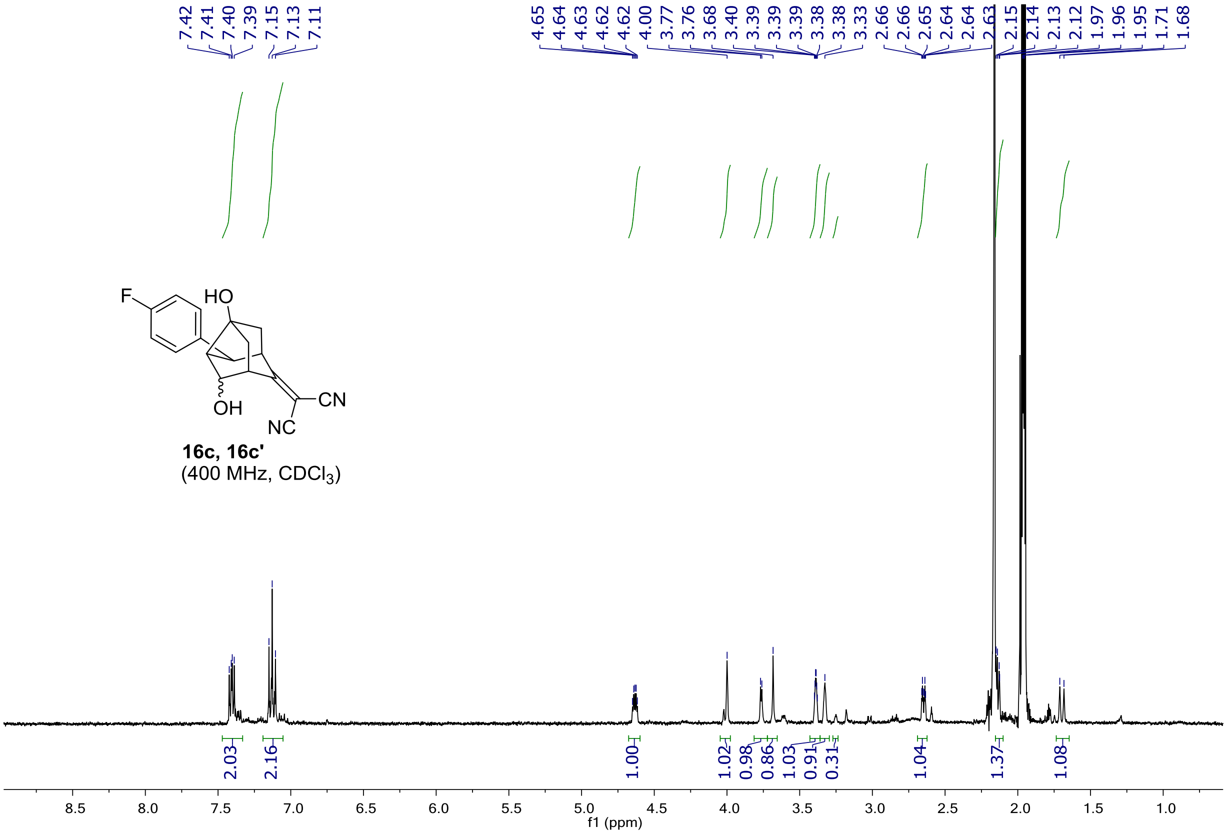
7.42
7.41
7.40
7.39
7.15
7.13
7.11
4.65
4.64
4.63
4.62
4.62
4.00
3.77
3.76
3.68
3.40
3.39
3.39
3.38
3.38
3.33
2.66
2.66
2.65
2.64
2.64
2.63
2.15
2.14
2.13
2.12
1.97
1.96
1.95
1.71
1.68
F
HO
OH
CN
NC
16c, 16c'
(400 MHz, CDCl3)
2.03
2.16
1.00
1.02
0.98
0.86
1.03
0.91
0.31
1.04
1.37
1.08
8.5
8.0
7.5
7.0
6.5
6.0
5.5
5.0
4.5
4.0
3.5
3.0
2.5
2.0
1.5
1.0
f1 (ppm)

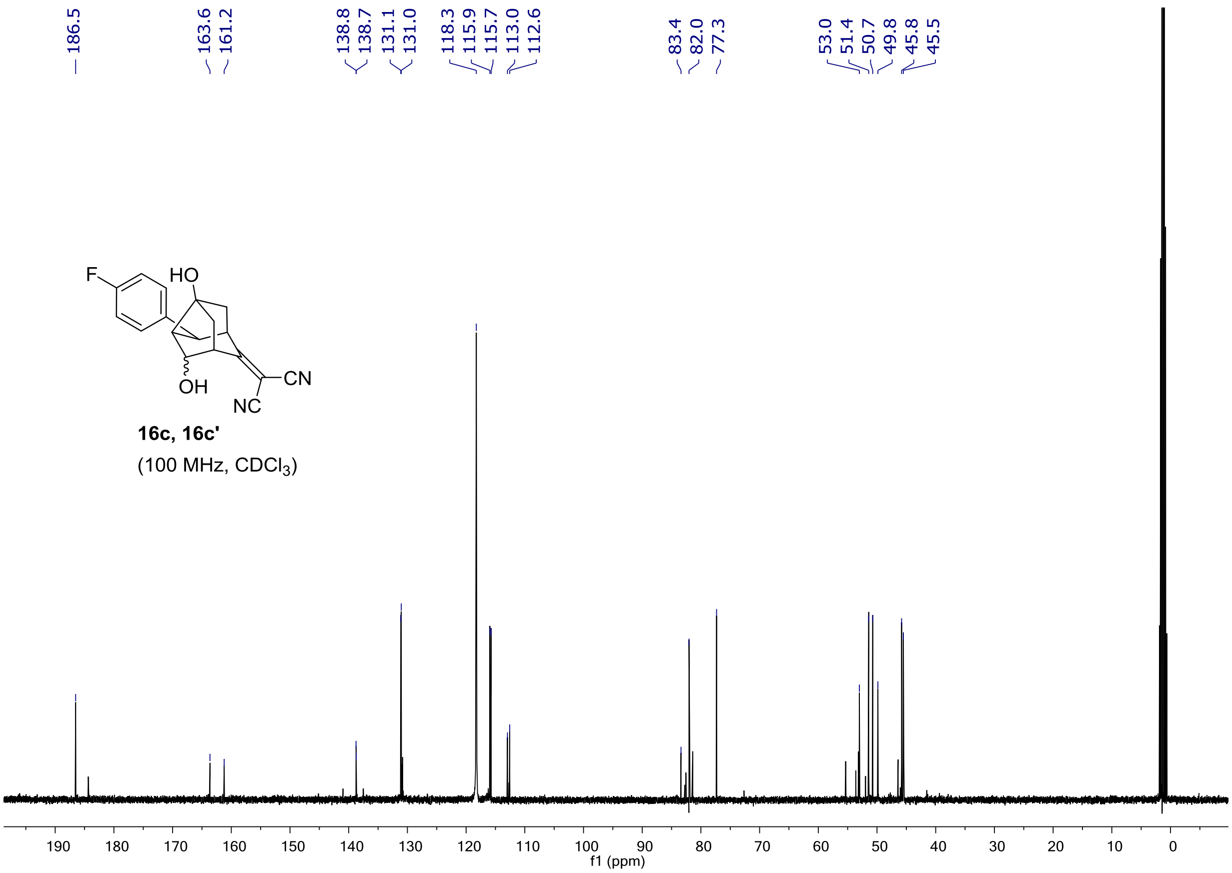
186.5
163.6
161.2
138.8
138.7
131.1
131.0
118.3
115.9
115.7
113.0
112.6
83.4
82.0
77.3
53.0
51.4
50.7
49.8
45.8
45.5
F
HO
OH
CN
NC
16c, 16c'
(100 MHz, CDCl3)
190
180
170
160
150
140
130
120
110
100
90
80
70
60
50
40
30
20
10
0
f1 (ppm)

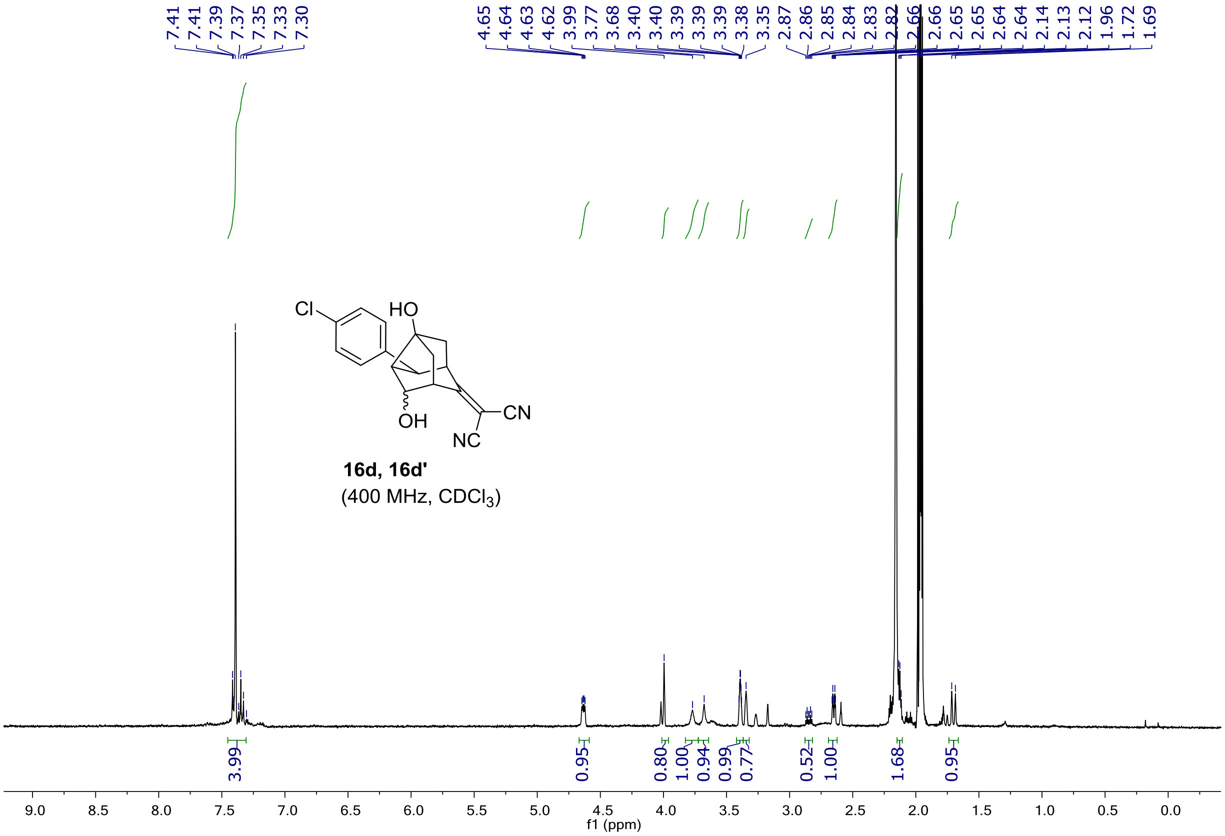

16d, 16d'
(400 MHz, $CDCl_3$)

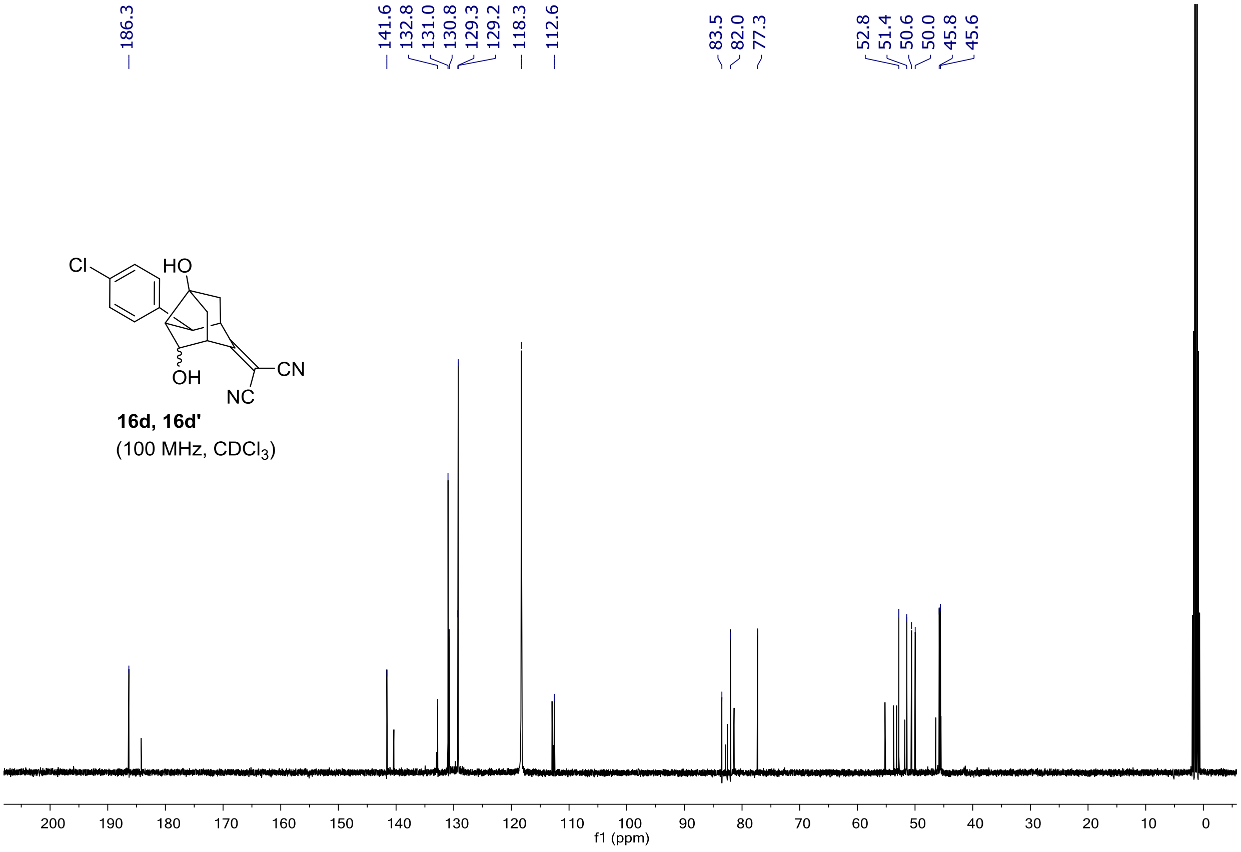

16d, 16d'
(100 MHz, $CDCl_3$)

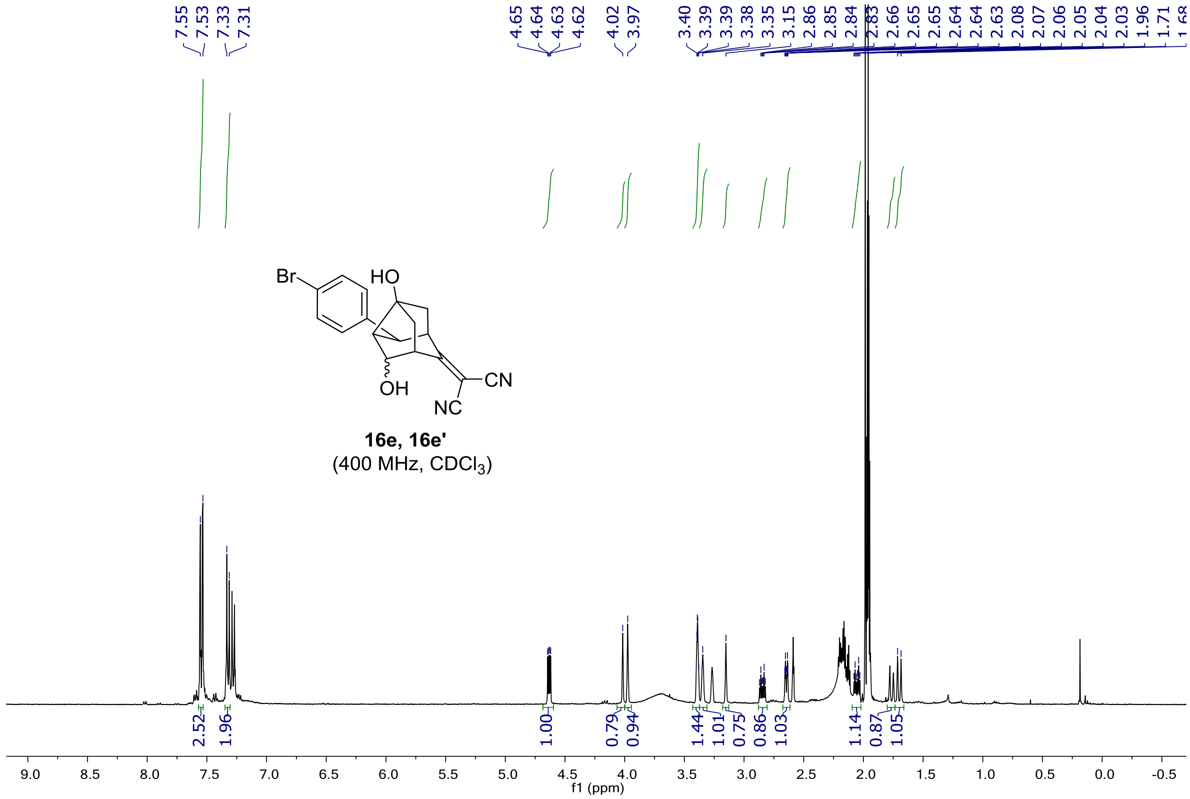
7.55 7.53 7.33 7.31
4.65 4.64 4.63 4.62 4.02 3.97
3.40 3.39 3.39 3.38 3.35 3.15 2.86 2.85 2.84 2.83 2.66 2.65 2.65 2.64 2.64 2.63 2.08 2.07 2.06 2.05 2.04 2.03 1.96 1.71
Br
HO
OH
CN
NC
16e, 16e'
(400 MHz, CDCl3)
2.52 1.96 1.00 0.79 0.94 1.44 1.01 0.75 0.86 1.03 1.14 0.87 1.05
9.0 8.5 8.0 7.5 7.0 6.5 6.0 5.5 5.0 4.5 4.0 3.5 3.0 2.5 2.0 1.5 1.0 0.5 0.0 -0.5
f1 (ppm)
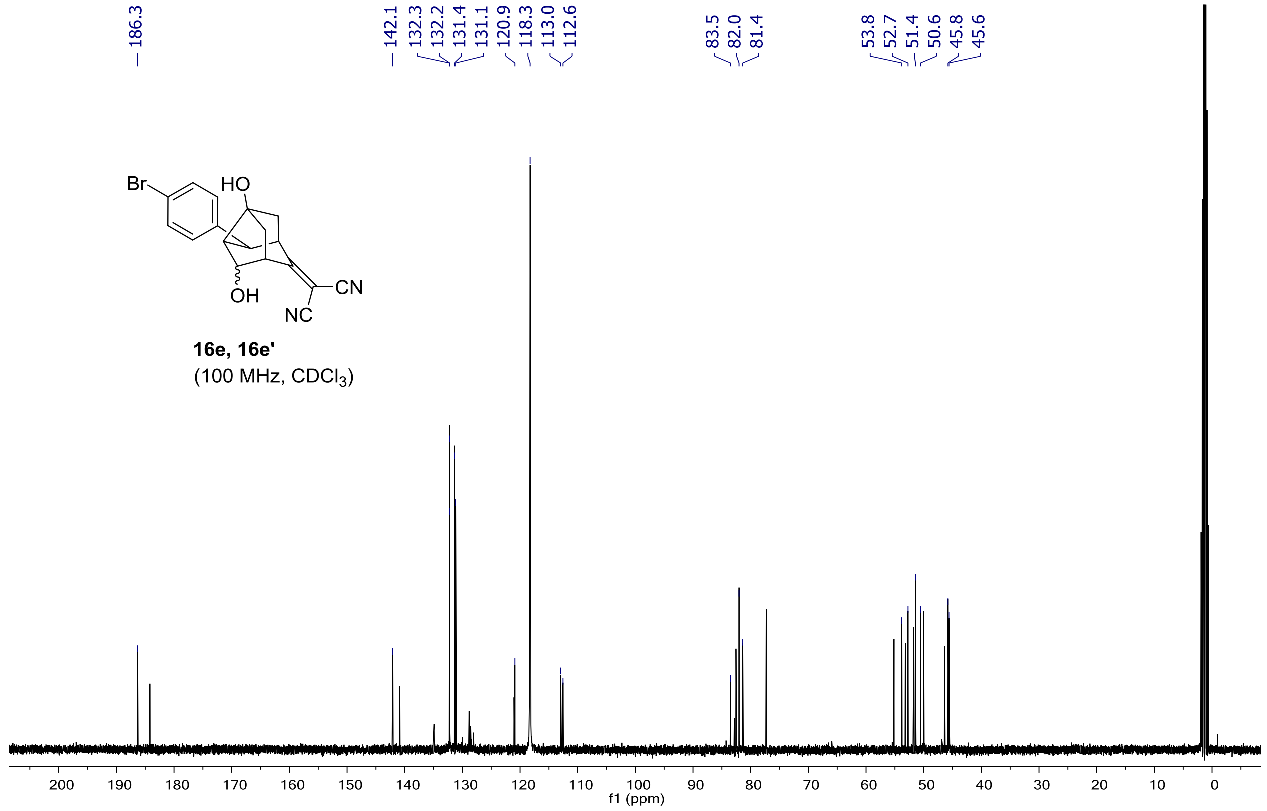
186.3
142.1 132.3 132.2 131.4 131.1 120.9 118.3 113.0 112.6
83.5 82.0 81.4
53.8 52.7 51.4 50.6 45.8 45.6
Br
HO
OH
CN
NC
16e, 16e'
(100 MHz, CDCl3)
200 190 180 170 160 150 140 130 120 110 100 90 80 70 60 50 40 30 20 10 0
f1 (ppm)

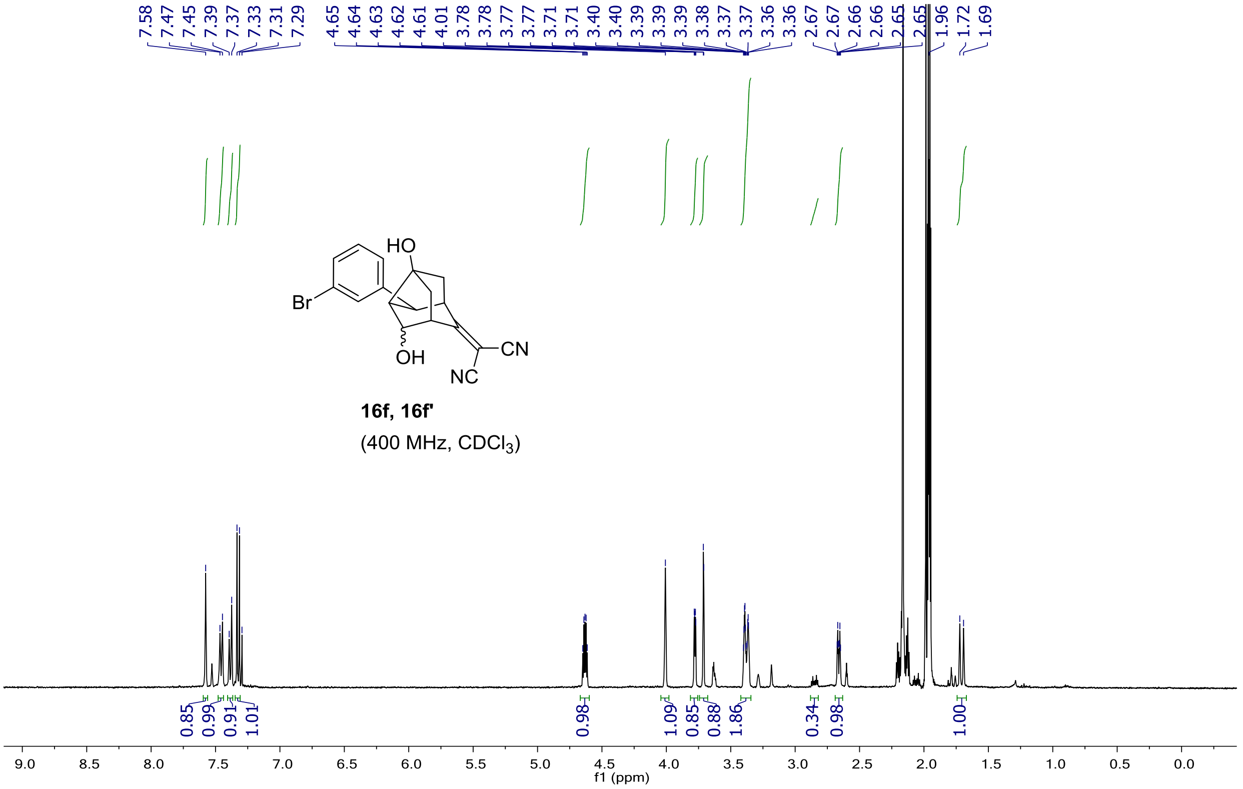
7.58 7.47 7.45 7.39 7.37 7.33 7.31 7.29 4.65 4.64 4.63 4.62 4.61 4.01 3.78 3.78 3.77 3.77 3.71 3.71 3.40 3.40 3.39 3.39 3.39 3.38 3.37 3.37 3.36 3.36 2.67 2.67 2.66 2.66 2.65 2.65 1.96 1.72 1.69
HO
Br
OH
CN
NC
16f, 16f'
(400 MHz, CDCl3)
0.85 0.99 0.91 1.01 0.98 1.09 0.85 0.88 1.86 0.34 0.98 1.00
f1 (ppm)

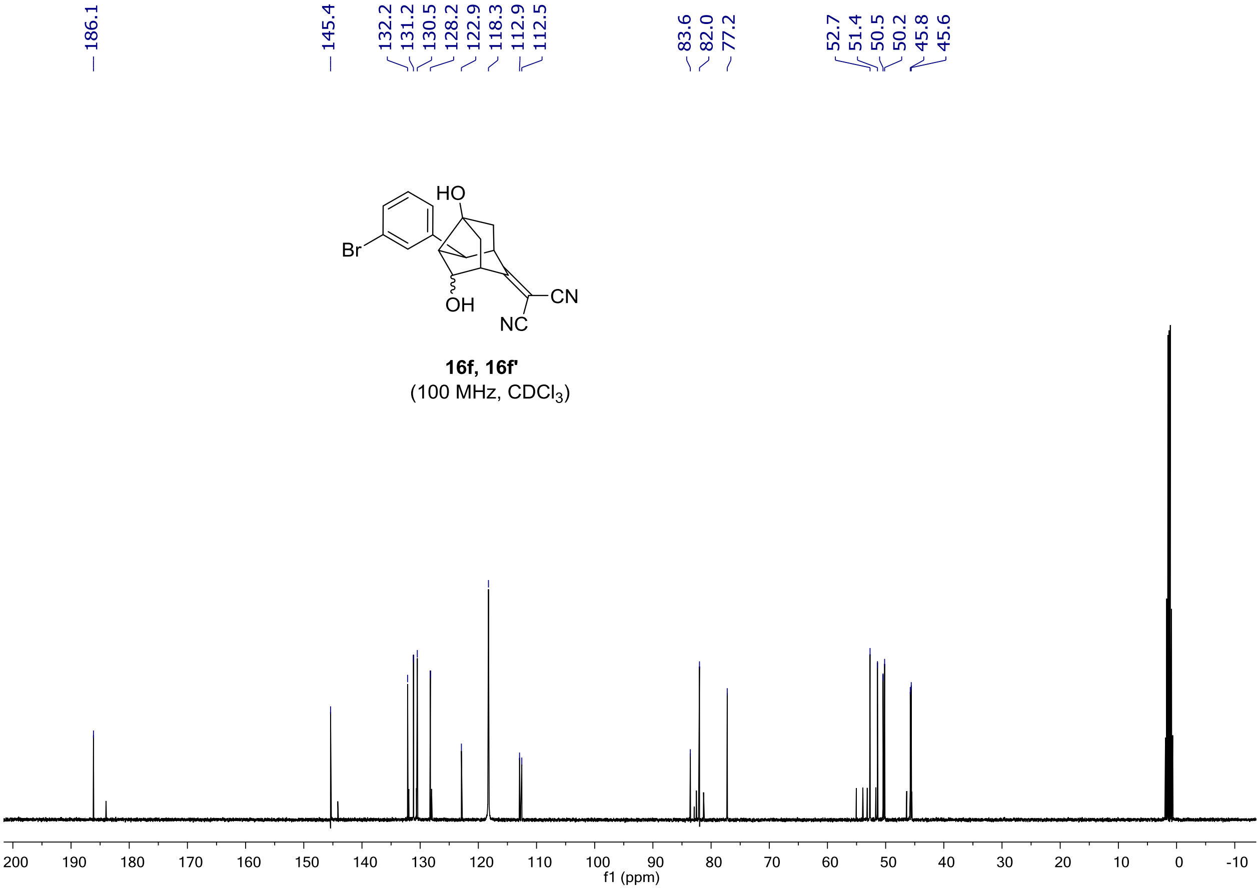
186.1 145.4 132.2 131.2 130.5 128.2 122.9 118.3 112.9 112.5 83.6 82.0 77.2 52.7 51.4 50.5 50.2 45.8 45.6
HO
Br
OH
CN
NC
16f, 16f'
(100 MHz, CDCl3)
f1 (ppm)

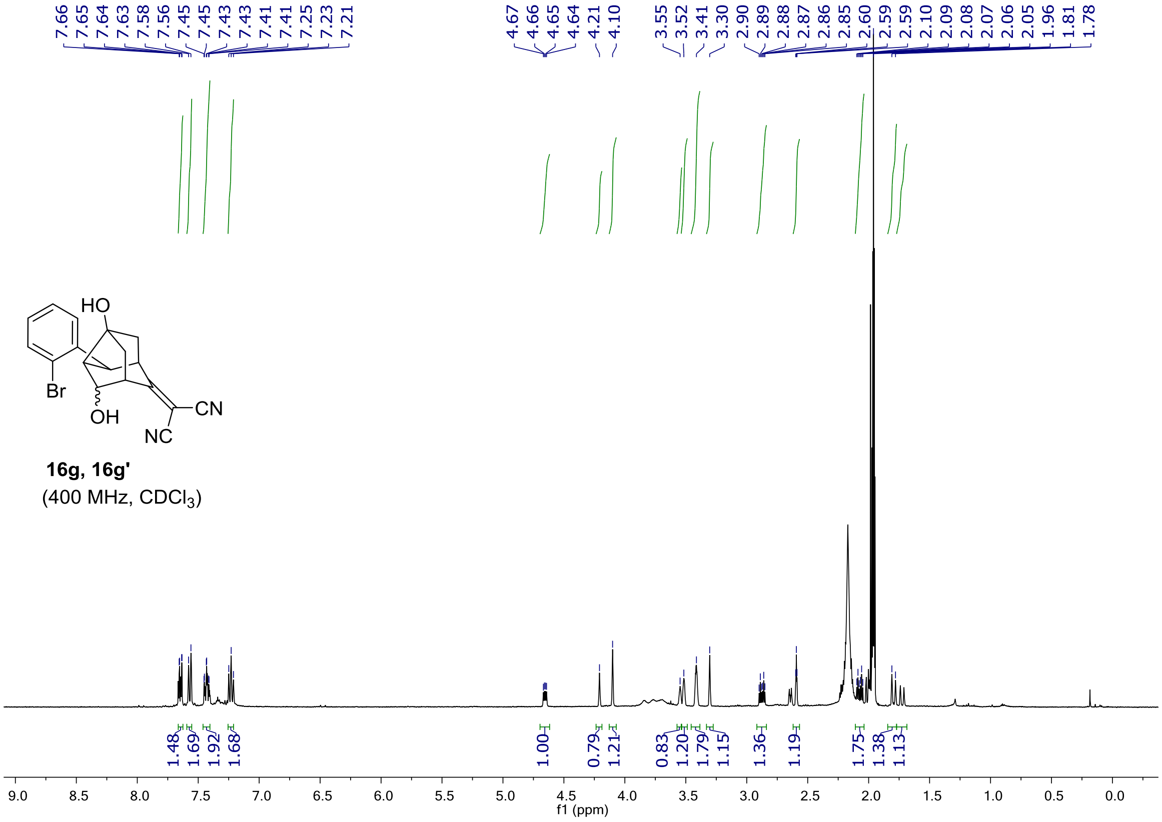

16g, 16g'
(400 MHz, $CDCl_3$)

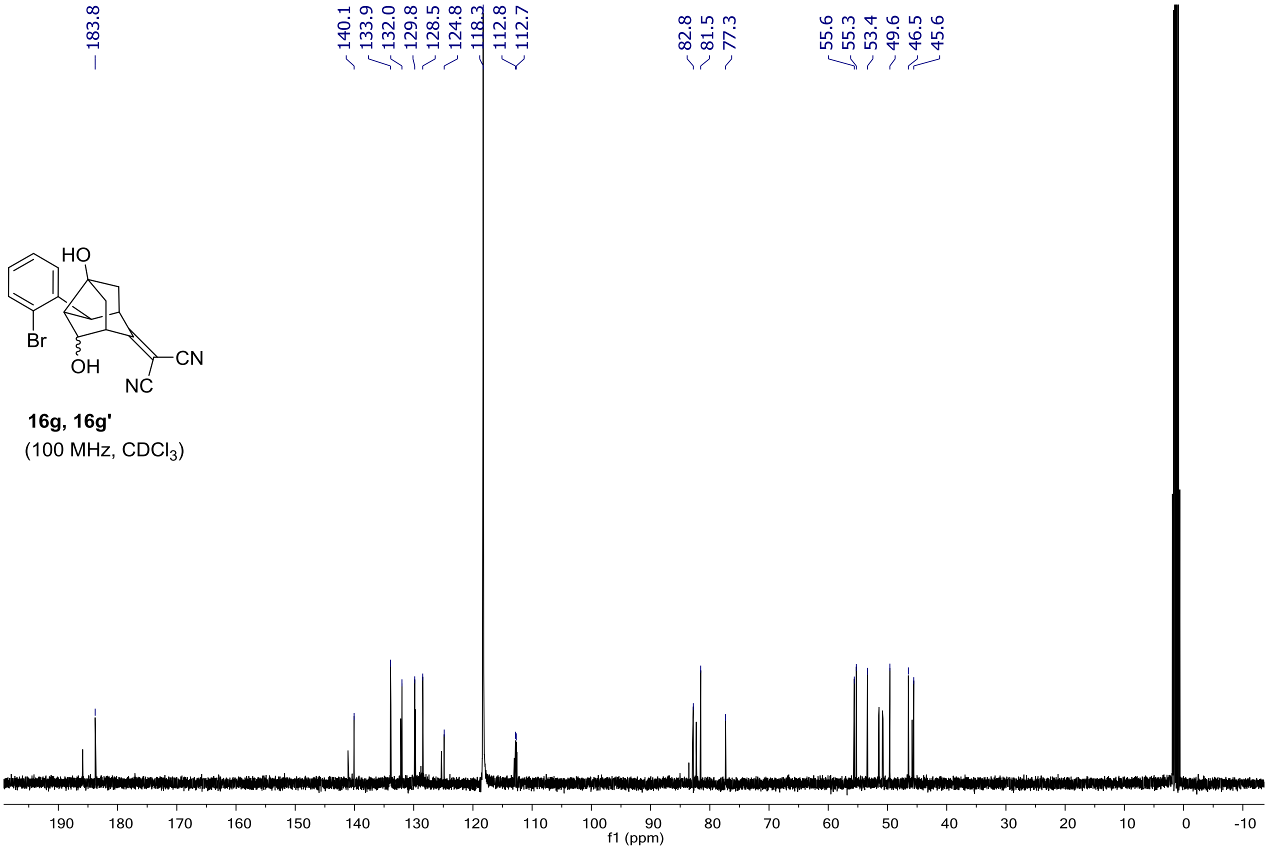

16g, 16g'
(100 MHz, $CDCl_3$)

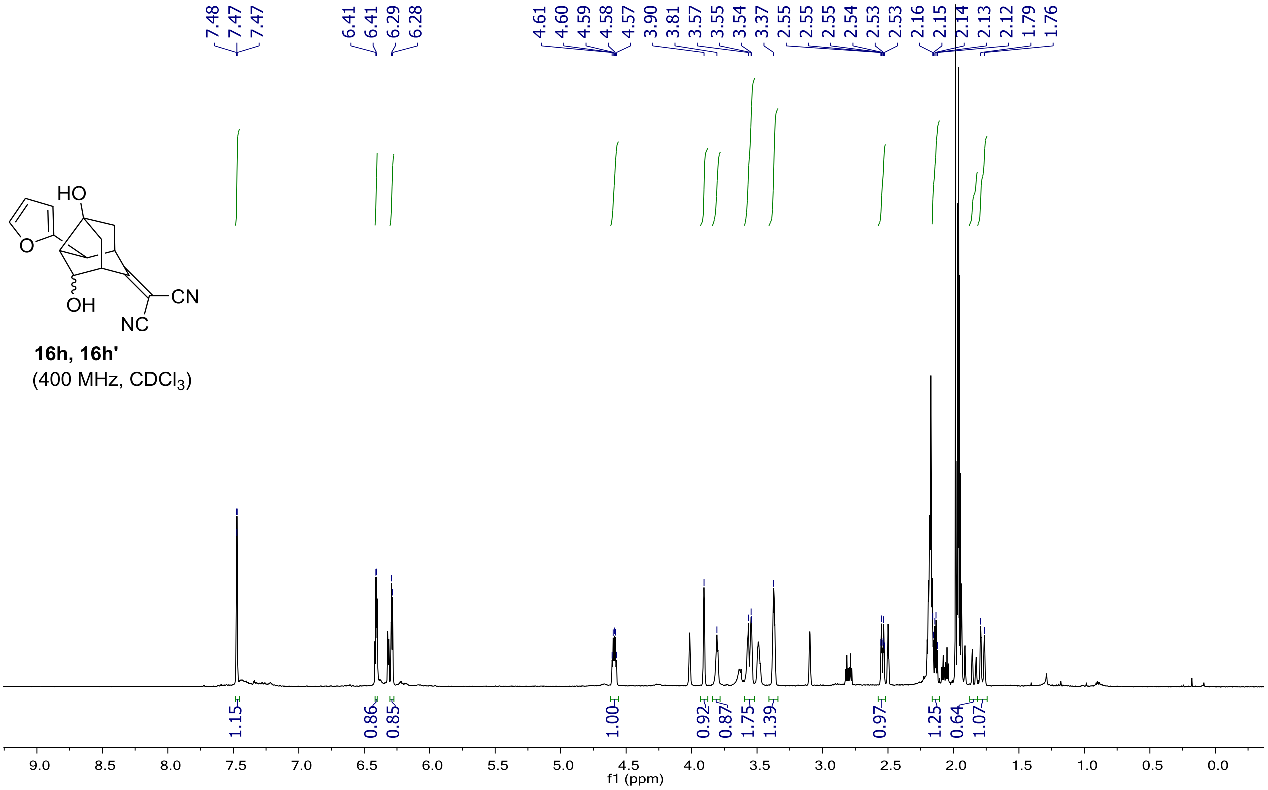
HO
O
OH
CN
NC
16h, 16h'
(400 MHz, $CDCl_3$)
7.48
7.47
7.47
6.41
6.41
6.29
6.28
4.61
4.60
4.59
4.58
4.57
3.90
3.81
3.57
3.55
3.54
3.37
2.55
2.55
2.55
2.54
2.53
2.53
2.16
2.15
2.14
2.13
2.12
1.79
1.76
1.15
0.86
0.85
1.00
0.92
0.87
1.75
1.39
0.97
1.25
0.64
1.07
9.0 8.5 8.0 7.5 7.0 6.5 6.0 5.5 5.0 4.5 4.0 3.5 3.0 2.5 2.0 1.5 1.0 0.5 0.0
f1 (ppm)

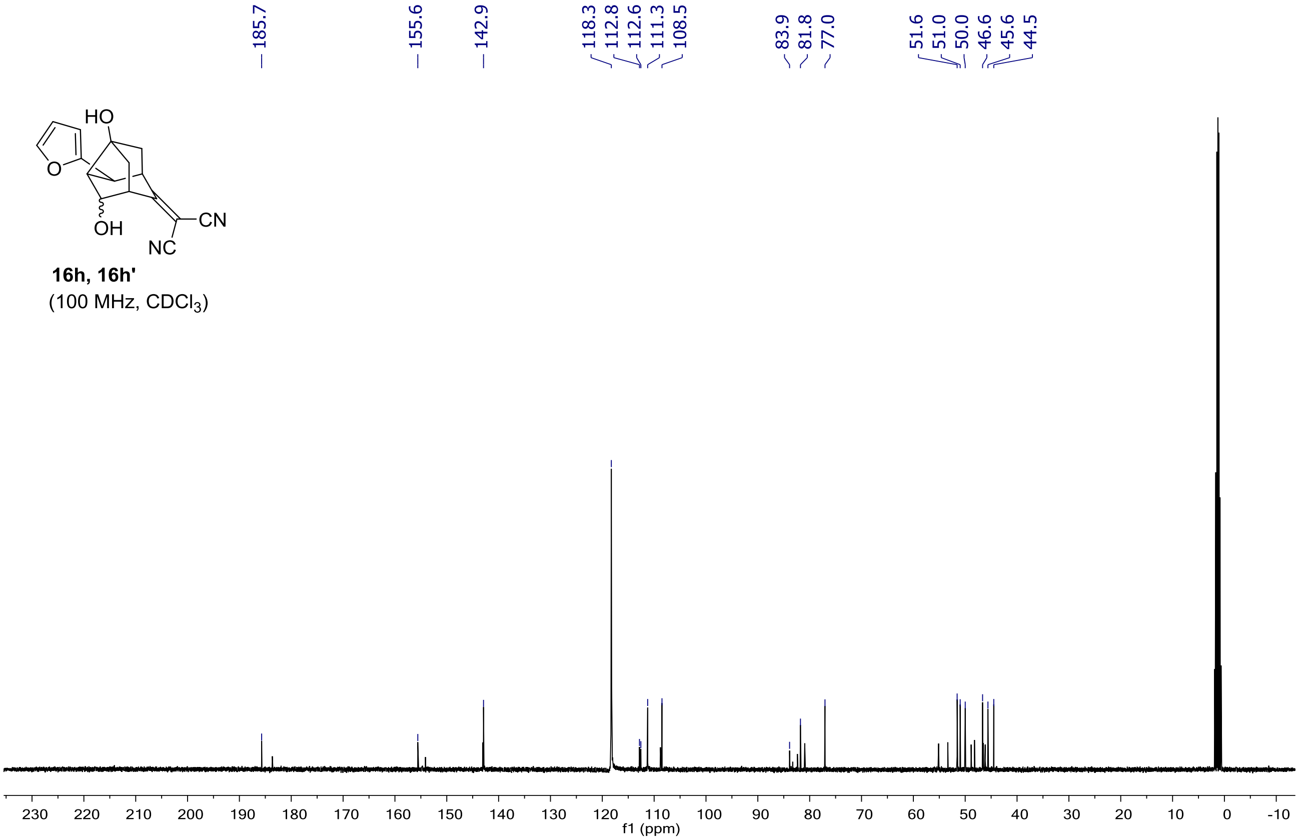
HO
O
OH
CN
NC
16h, 16h'
(100 MHz, $CDCl_3$)
185.7
155.6
142.9
118.3
112.8
112.6
111.3
108.5
83.9
81.8
77.0
51.6
51.0
50.0
46.6
45.6
44.5
230 220 210 200 190 180 170 160 150 140 130 120 110 100 90 80 70 60 50 40 30 20 10 0 -10
f1 (ppm)

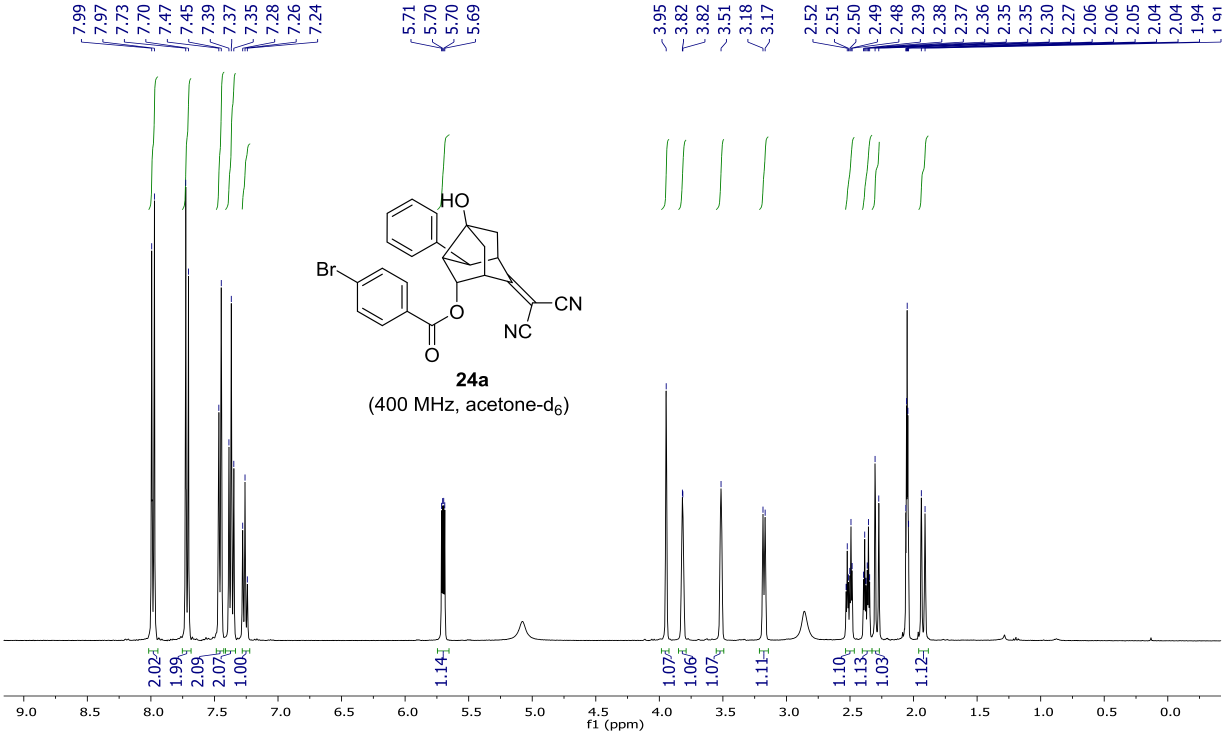

24a
(400 MHz, acetone-d$_6$)

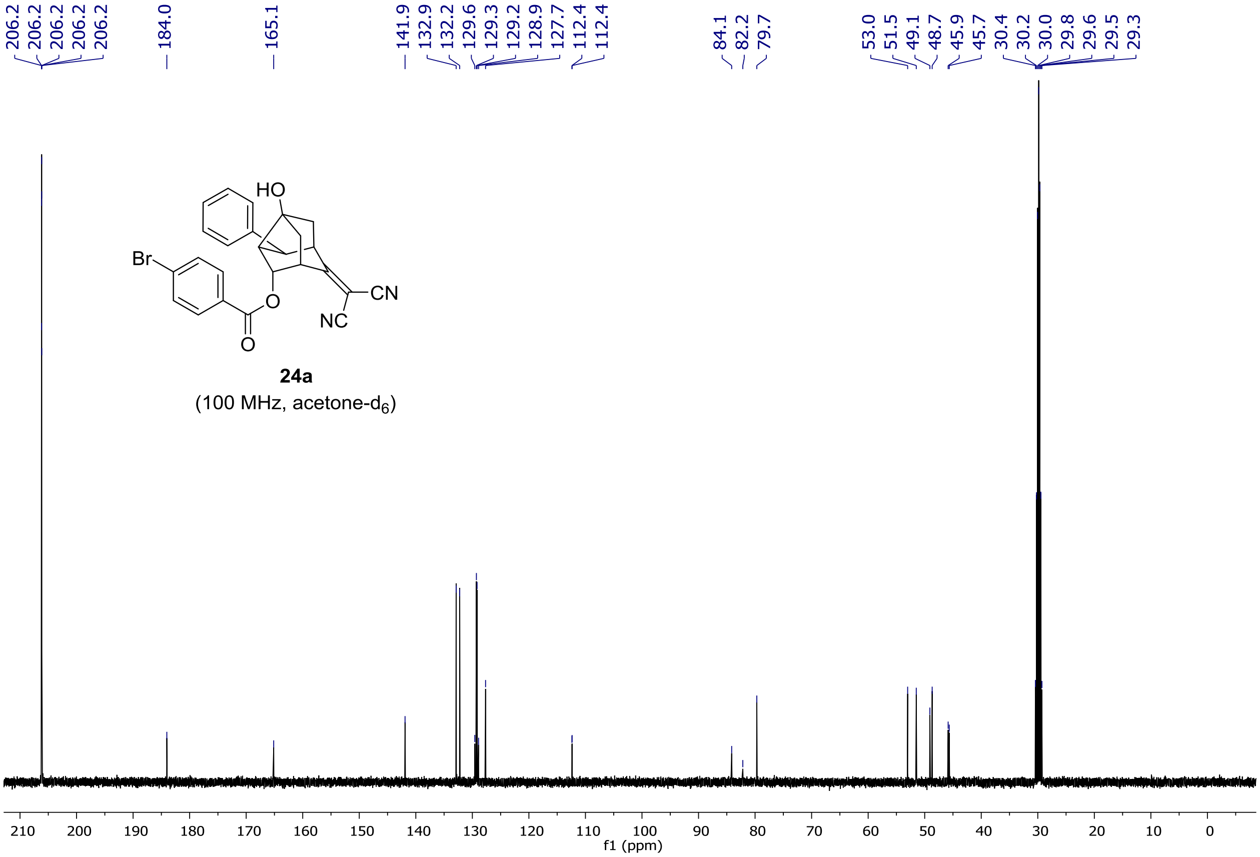

24a
(100 MHz, acetone-d$_6$)

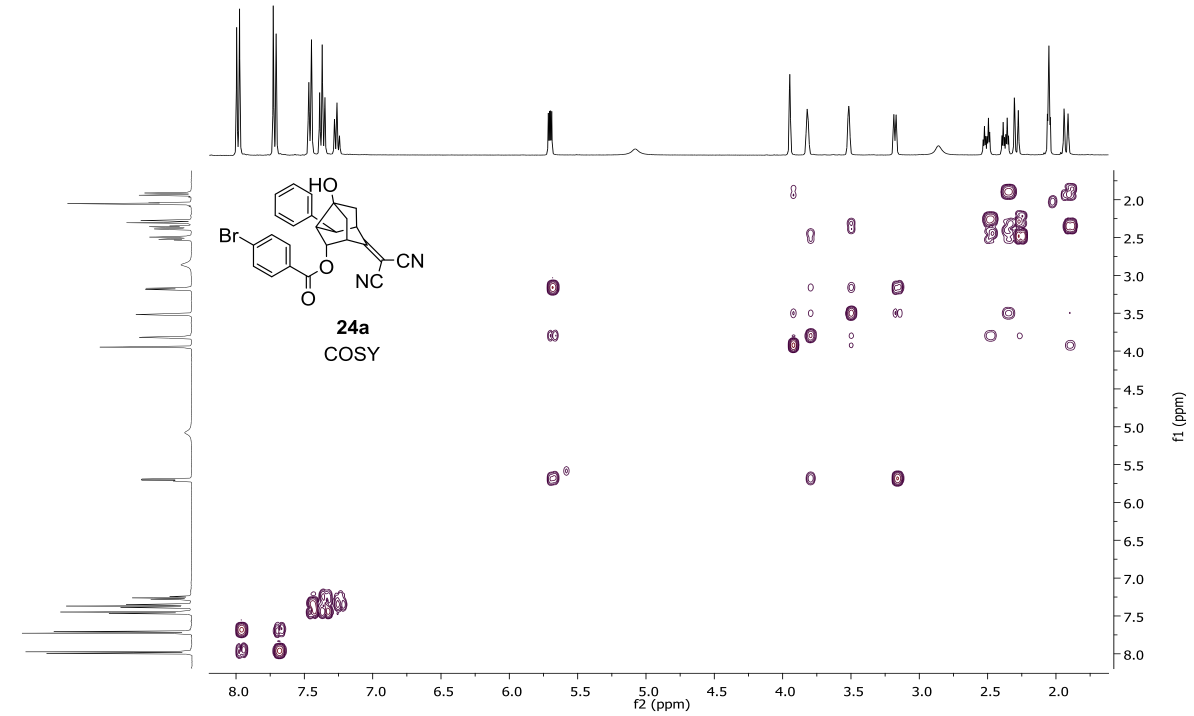
HO
Br
CN
NC
O
24a
COSY
f2 (ppm)
f1 (ppm)

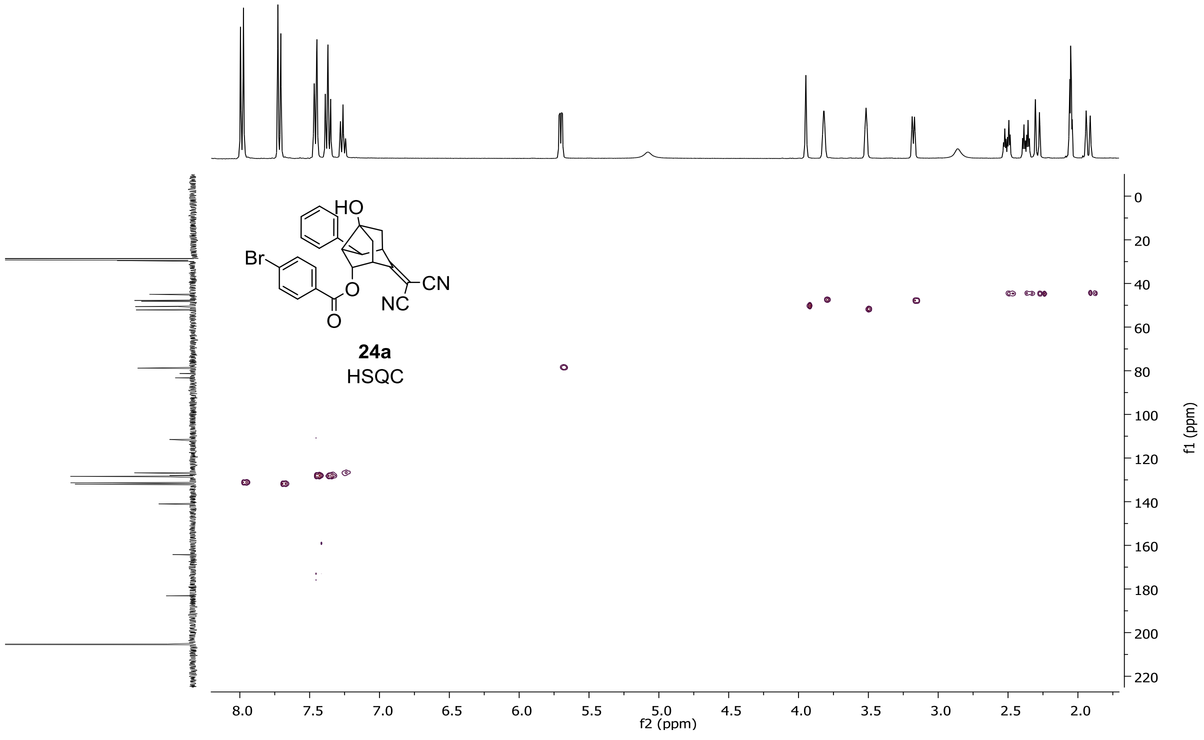
HO
Br
CN
NC
O
24a
HSQC
f2 (ppm)
f1 (ppm)

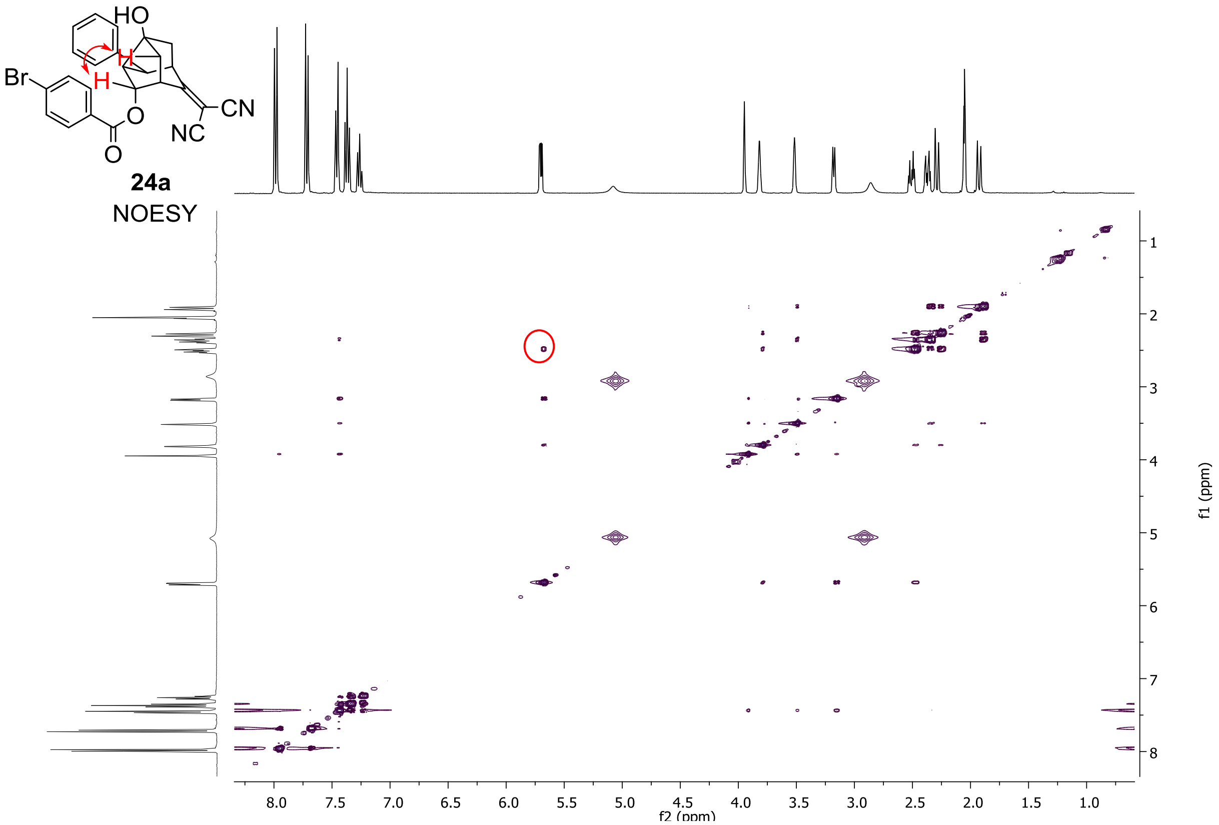
HO
Br
H
H
CN
NC
O
O
24a
NOESY
f2 (ppm)
f1 (ppm)

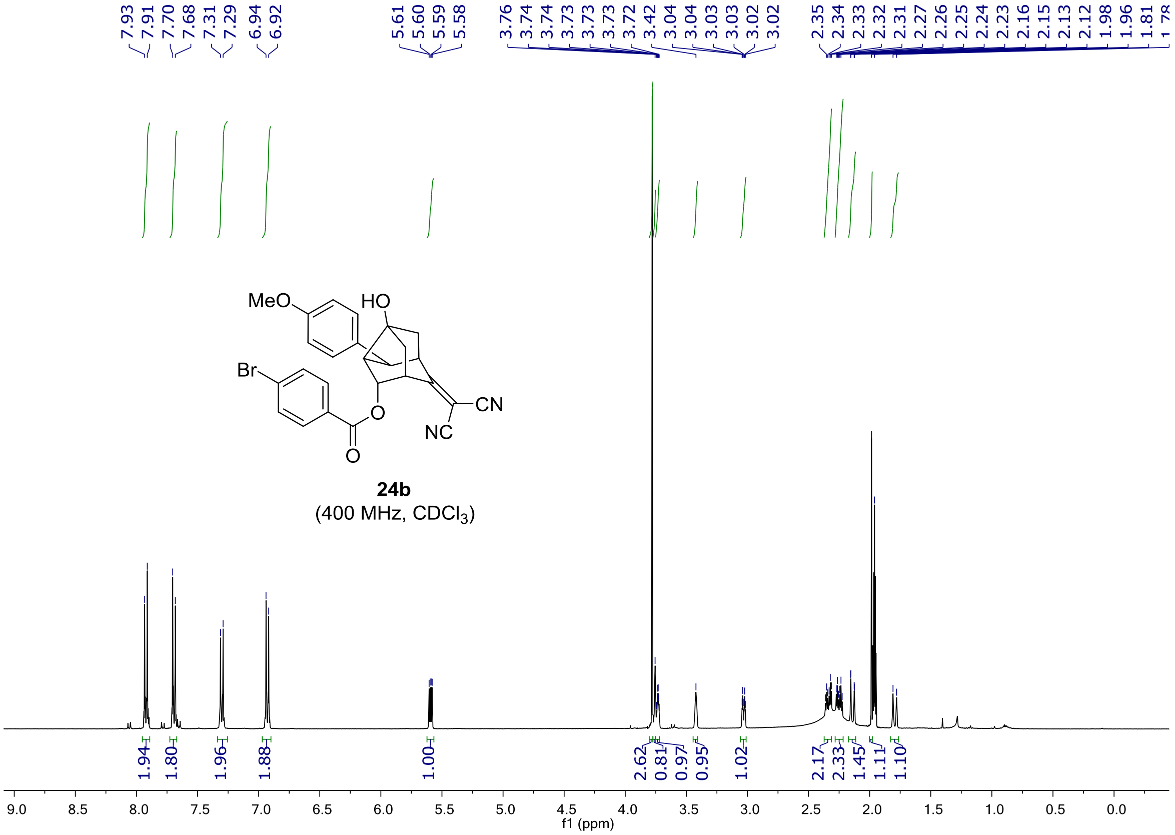

24b
(400 MHz, $CDCl_3$)

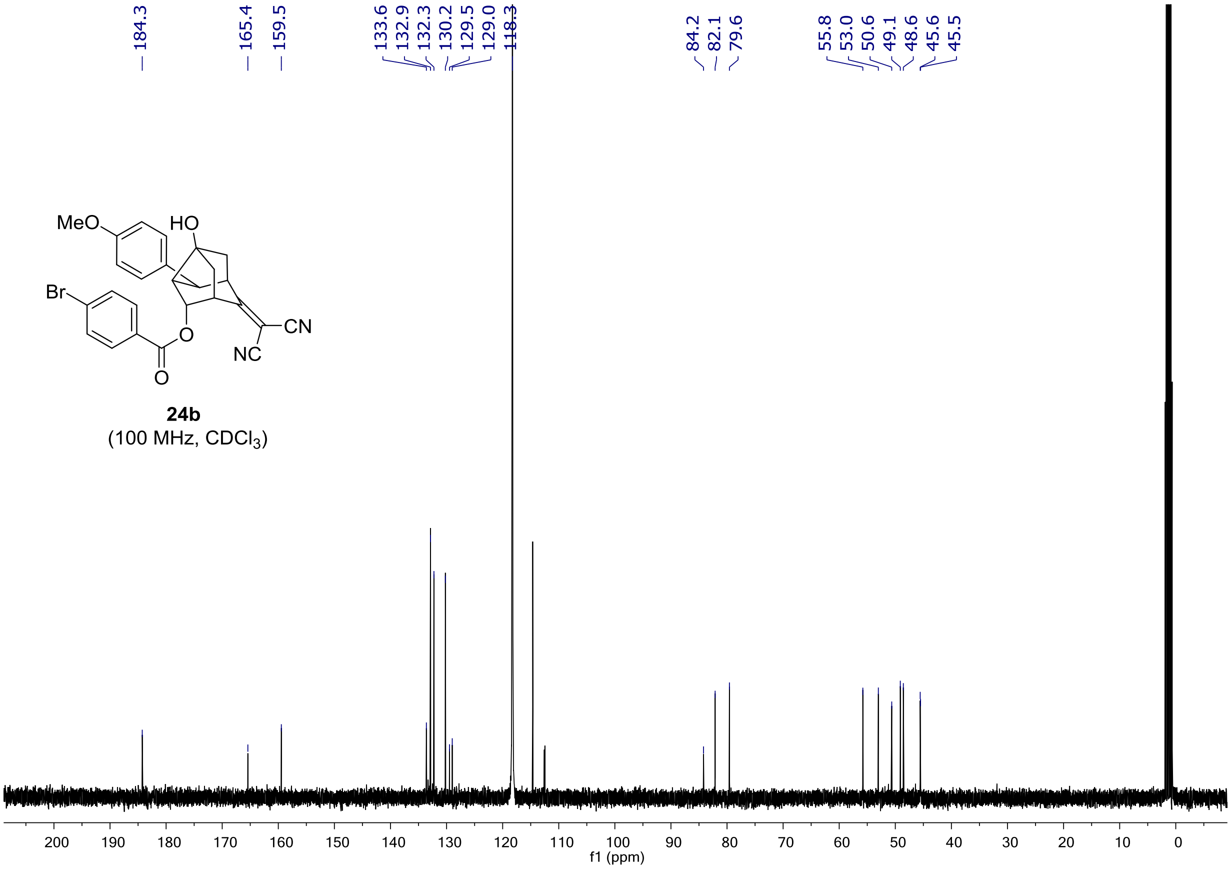

24b
(100 MHz, $CDCl_3$)

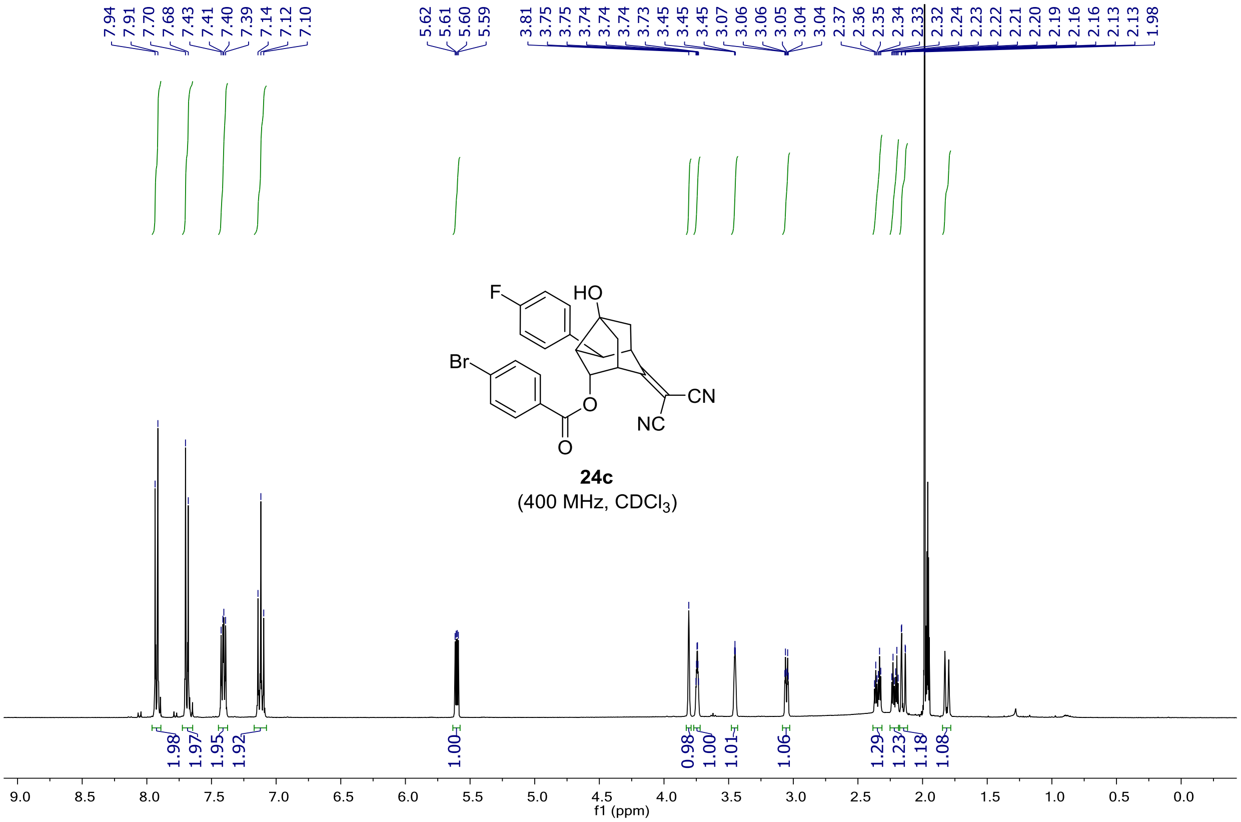
7.94 7.91 7.70 7.68 7.43 7.41 7.40 7.39 7.14 7.12 7.10
5.62 5.61 5.60 5.59
3.81 3.75 3.75 3.74 3.74 3.74 3.73 3.45 3.45 3.45 3.07 3.06 3.06 3.05 3.04 3.04
2.37 2.36 2.35 2.34 2.33 2.32 2.24 2.23 2.22 2.21 2.20 2.19 2.16 2.13 2.13 1.98
F
HO
Br
O
CN
NC
O
24c
(400 MHz, $CDCl_3$)
1.98 1.97 1.95 1.92 1.00 0.98 1.00 1.01 1.06 1.29 1.23 1.18 1.08
9.0 8.5 8.0 7.5 7.0 6.5 6.0 5.5 5.0 4.5 4.0 3.5 3.0 2.5 2.0 1.5 1.0 0.5 0.0
f1 (ppm)

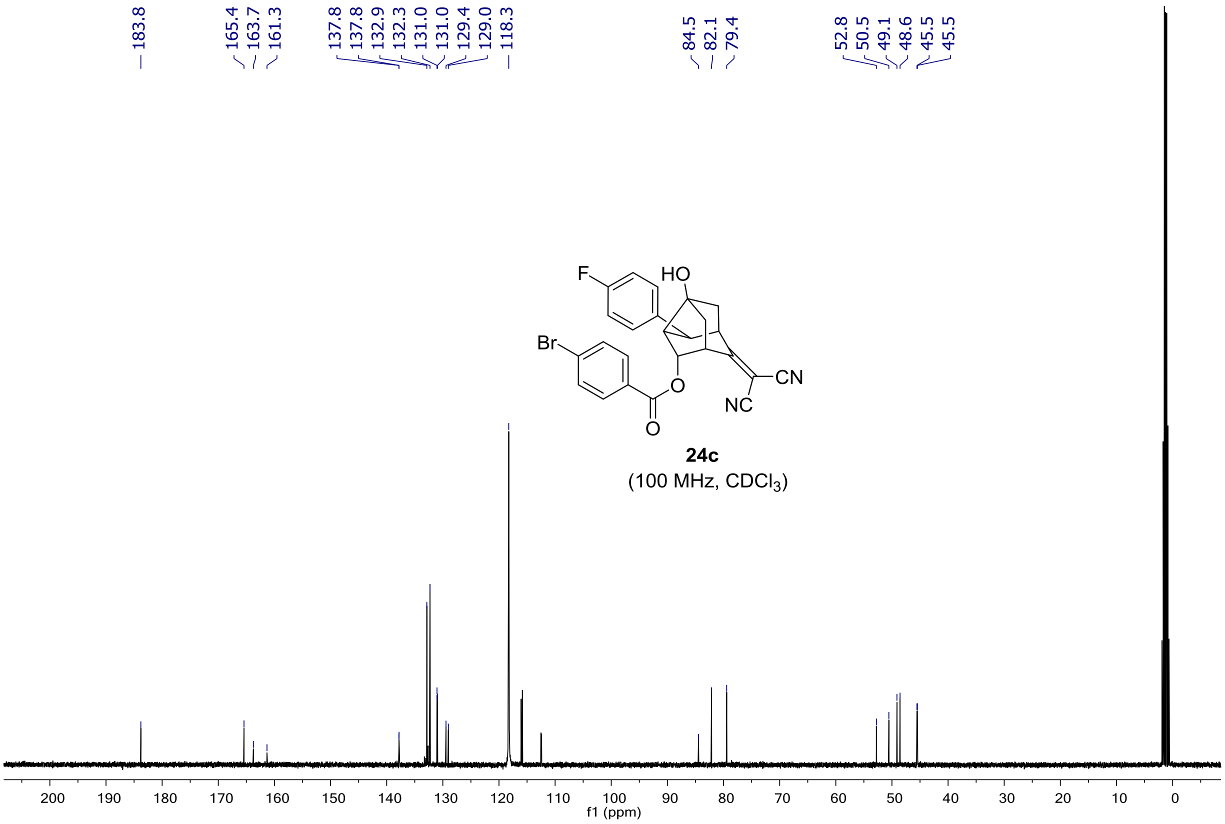
183.8
165.4 163.7 161.3
137.8 137.8 132.9 132.3 131.0 131.0 129.4 129.0 118.3
84.5 82.1 79.4
52.8 50.5 49.1 48.6 45.5 45.5
F
HO
Br
O
CN
NC
O
24c
(100 MHz, $CDCl_3$)
200 190 180 170 160 150 140 130 120 110 100 90 80 70 60 50 40 30 20 10 0
f1 (ppm)

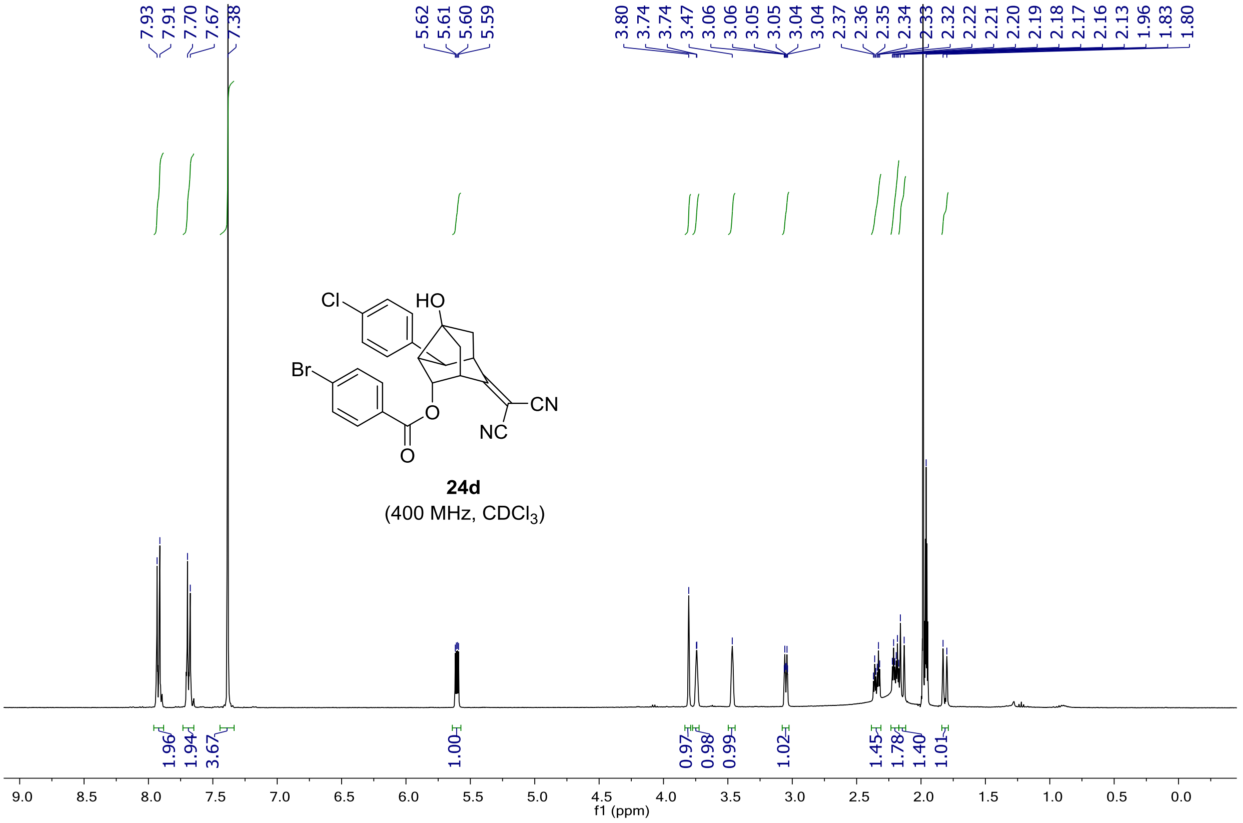

24d
(400 MHz, $CDCl_3$)

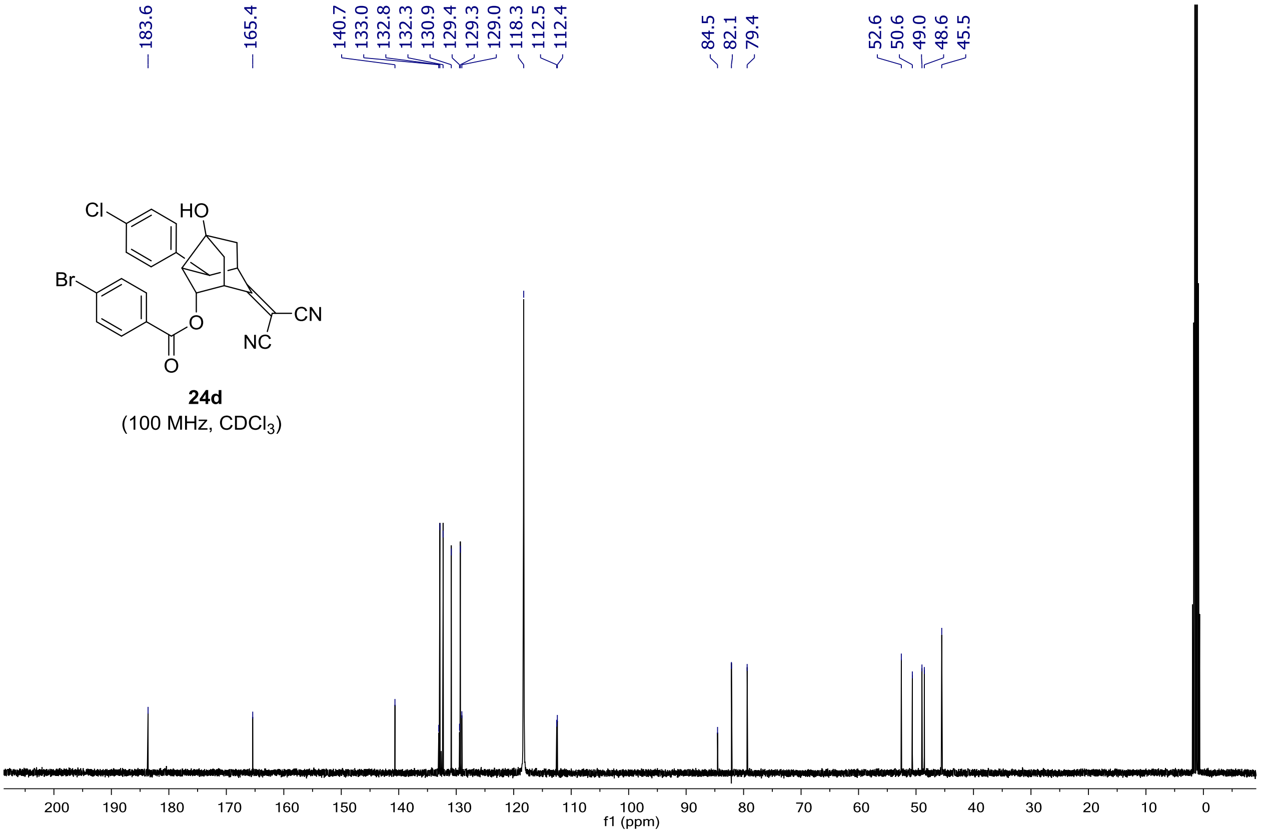

24d
(100 MHz, $CDCl_3$)

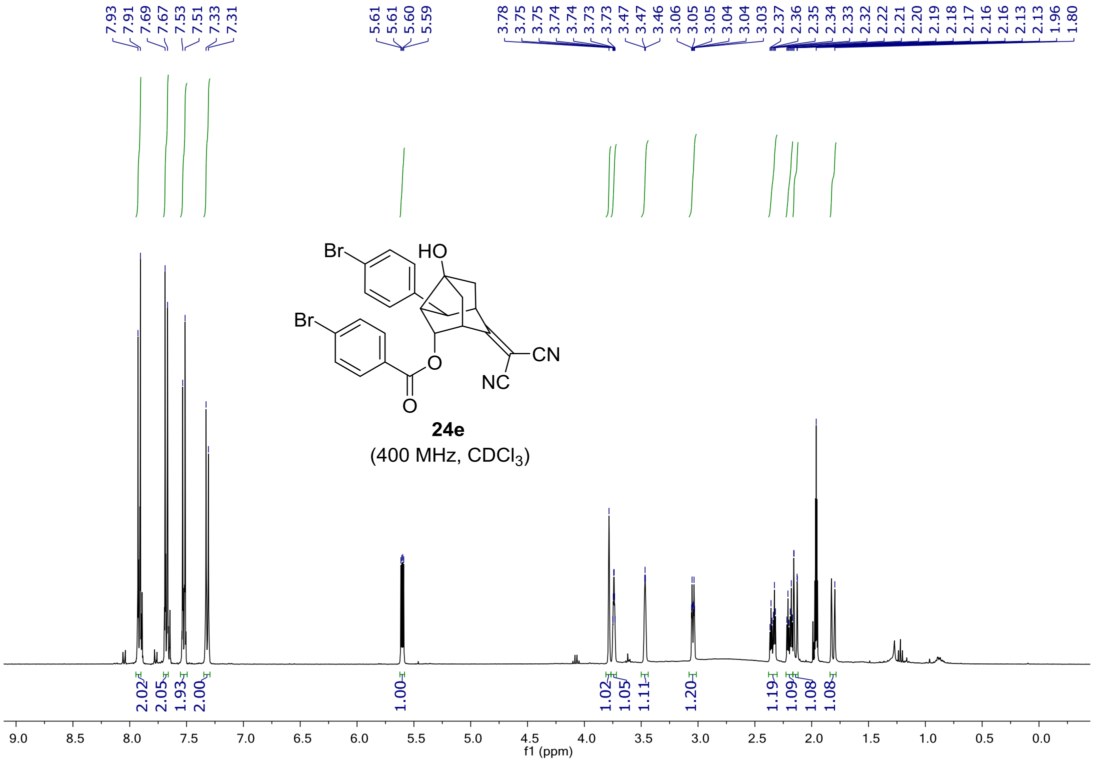
7.93 7.91 7.69 7.67 7.53 7.51 7.33 7.31
5.61 5.61 5.60 5.59
3.78 3.75 3.75 3.74 3.74 3.73 3.73 3.47 3.47 3.46 3.06 3.05 3.05 3.04 3.04 3.03 2.37 2.36 2.35 2.34 2.33 2.32 2.22 2.21 2.20 2.19 2.18 2.17 2.16 2.16 2.13 2.13 1.96 1.80
Br
HO
Br
CN
NC
O
O
24e
(400 MHz, CDCl3)
2.02 2.05 1.93 2.00 1.00 1.02 1.05 1.11 1.20 1.19 1.09 1.08 1.08
f1 (ppm)

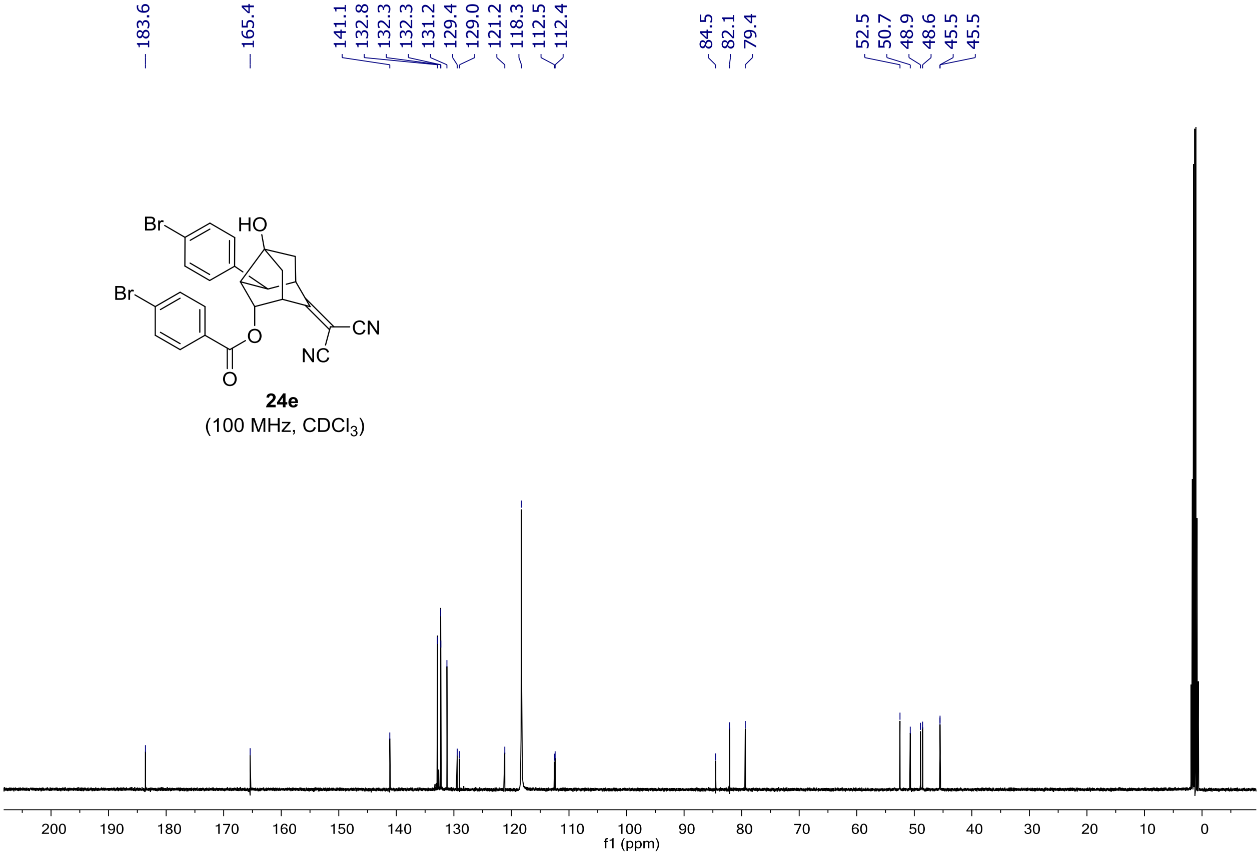
183.6
165.4
141.1 132.8 132.3 132.3 131.2 129.4 129.0 121.2 118.3 112.5 112.4
84.5 82.1 79.4
52.5 50.7 48.9 48.6 45.5 45.5
Br
HO
Br
CN
NC
O
O
24e
(100 MHz, CDCl3)
f1 (ppm)

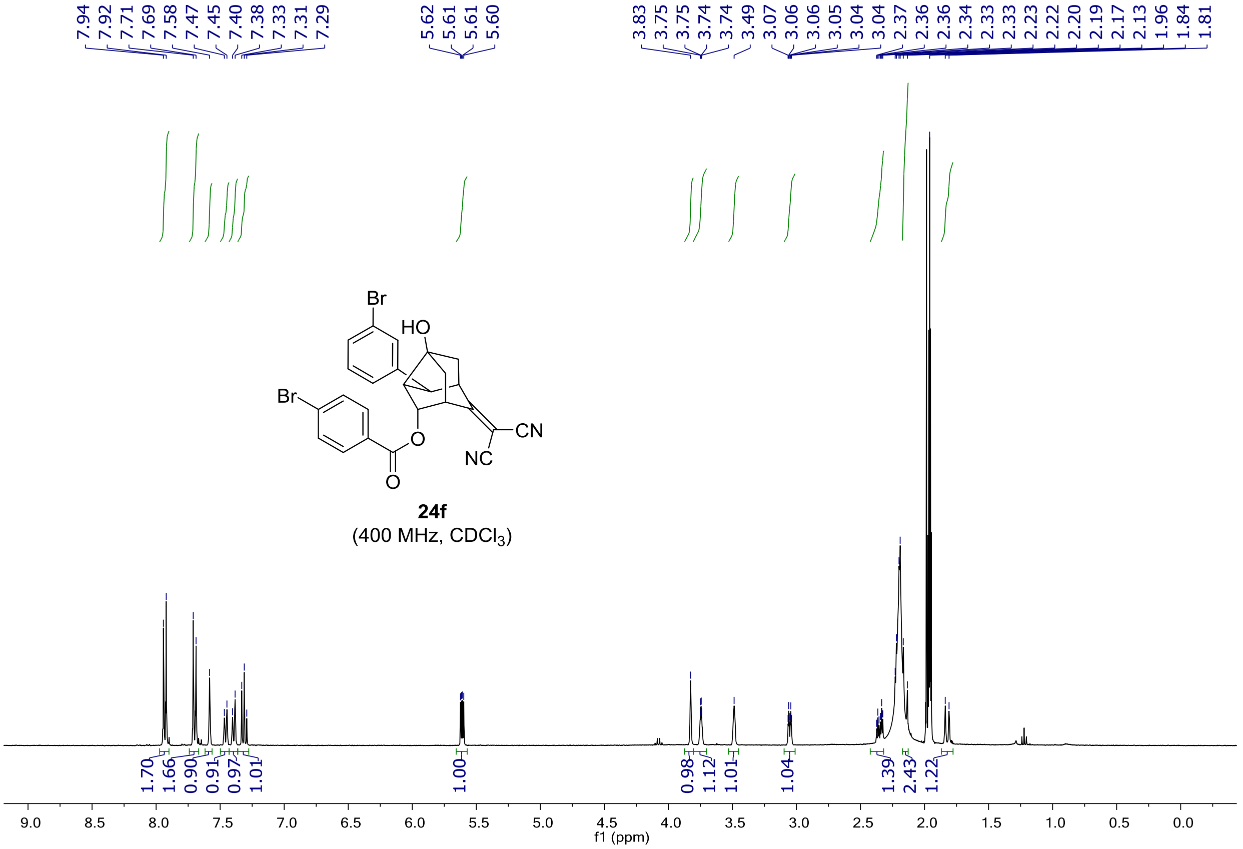
7.94 7.92 7.71 7.69 7.58 7.47 7.45 7.40 7.38 7.33 7.31 7.29
5.62 5.61 5.61 5.60
3.83 3.75 3.75 3.74 3.74 3.49 3.07 3.06 3.05 3.04 3.04 2.37 2.36 2.36 2.34 2.33 2.33 2.23 2.22 2.20 2.19 2.17 2.13 1.96 1.84 1.81
Br
HO
Br
CN
NC
O
O
24f
(400 MHz, CDCl3)
1.70 1.66 0.90 0.91 0.97 1.01 1.00 0.98 1.12 1.01 1.04 1.39 2.43 1.22
f1 (ppm)

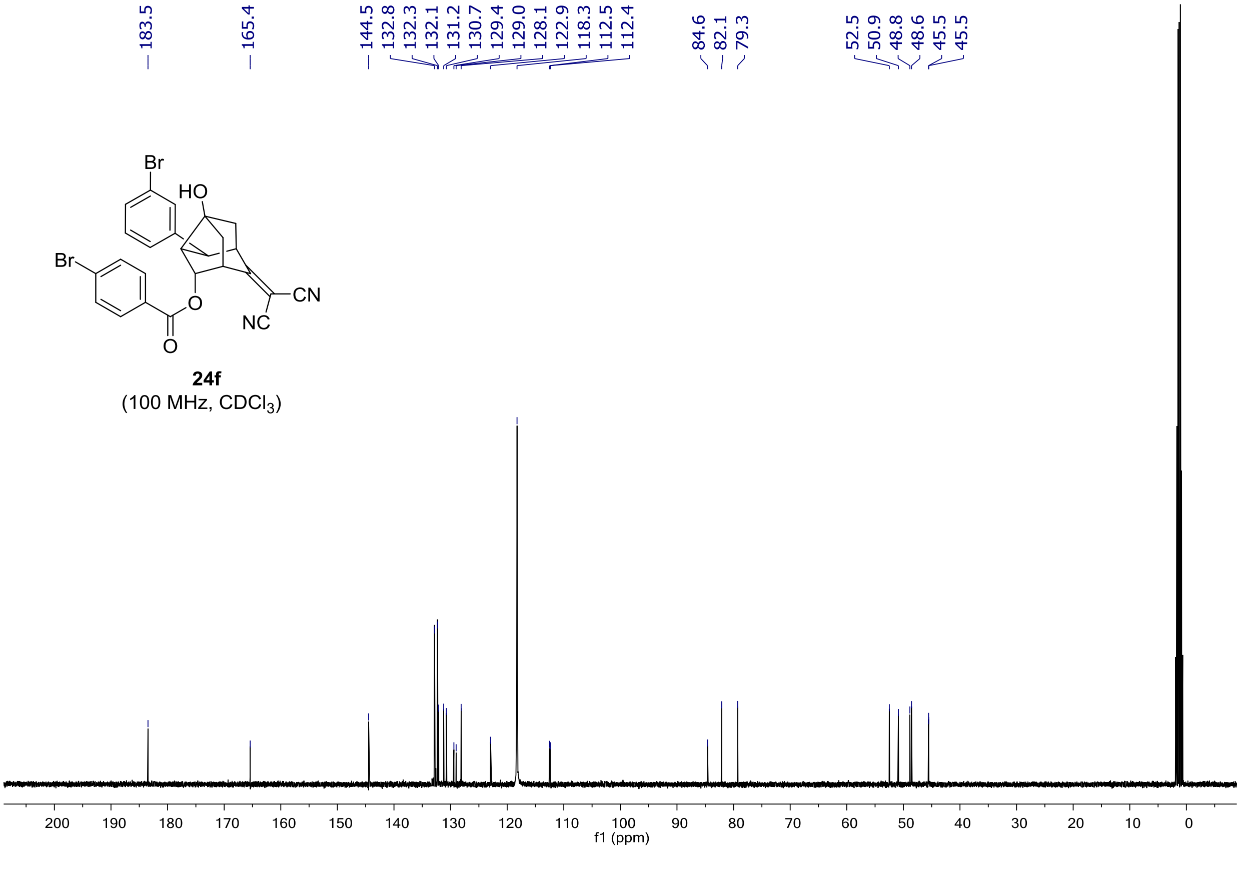
183.5
165.4
144.5 132.8 132.3 132.1 131.2 130.7 129.4 129.0 128.1 122.9 118.3 112.5 112.4
84.6 82.1 79.3
52.5 50.9 48.8 48.6 45.5 45.5
Br
HO
Br
CN
NC
O
O
24f
(100 MHz, CDCl3)
f1 (ppm)

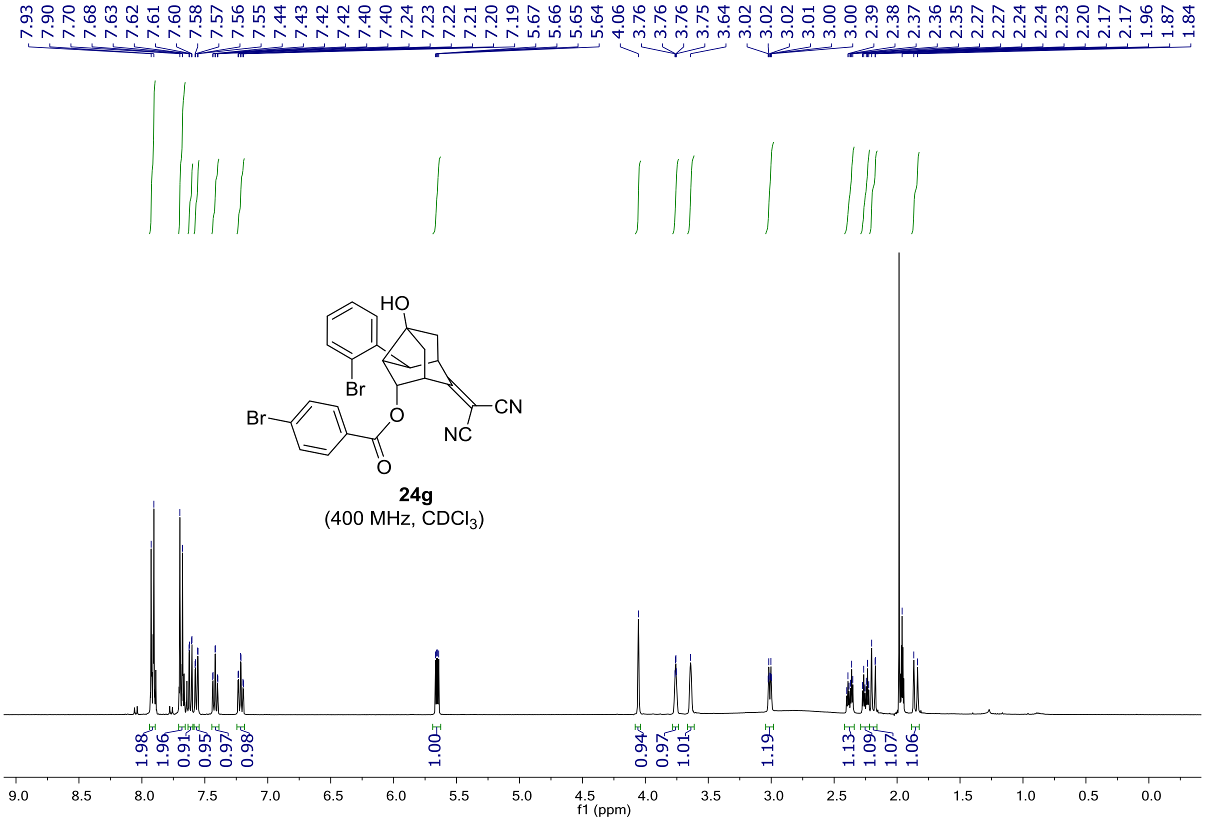
HO
Br
Br
O
CN
NC
O
24g
(400 MHz, CDCl3)
f1 (ppm)

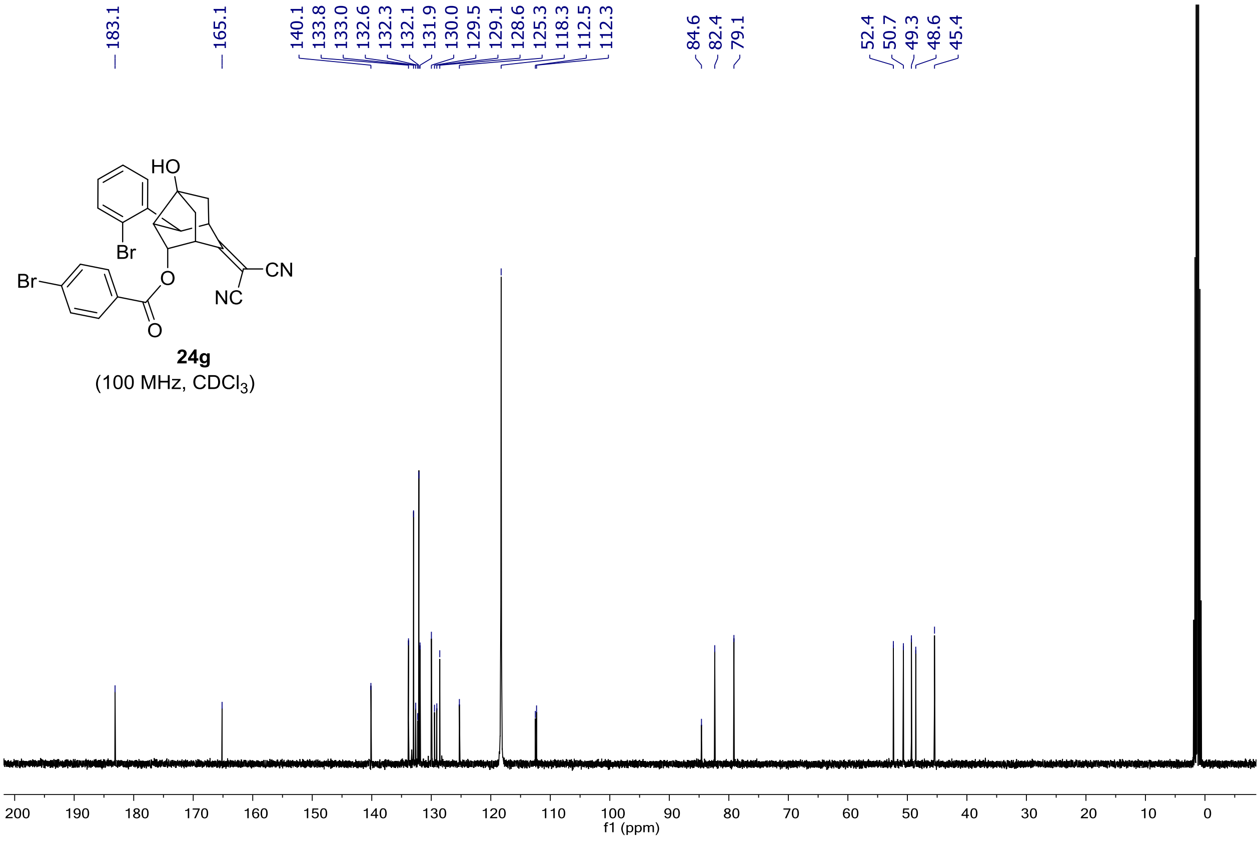
HO
Br
Br
O
CN
NC
O
24g
(100 MHz, CDCl3)
f1 (ppm)

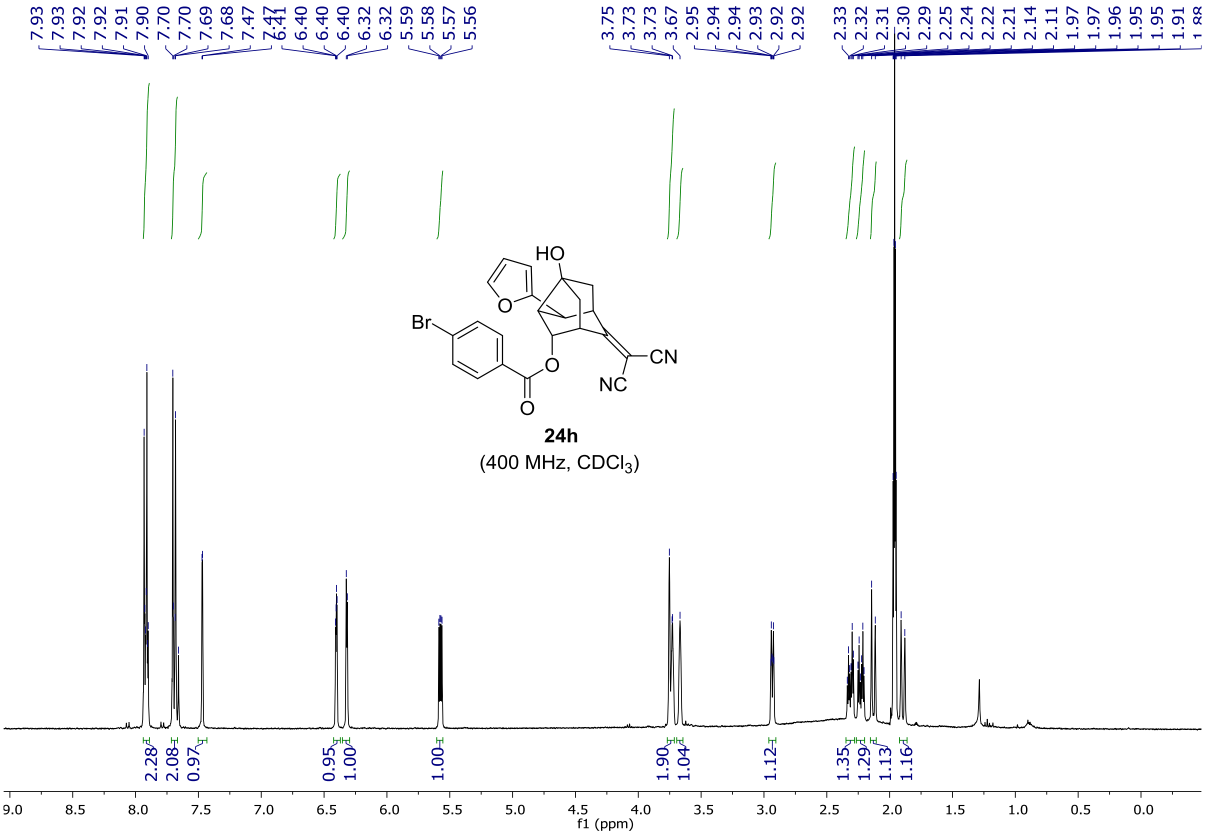
7.93 7.93 7.92 7.92 7.91 7.90 7.70 7.70 7.69 7.68 7.47 7.47 6.41 6.40 6.40 6.40 6.32 6.32 5.59 5.58 5.57 5.56
3.75 3.73 3.73 3.67 2.95 2.94 2.94 2.93 2.92 2.92
2.33 2.32 2.31 2.30 2.29 2.25 2.24 2.22 2.21 2.14 2.11 1.97 1.97 1.96 1.95 1.91
HO
O
Br
O
CN
NC
O
24h
(400 MHz, CDCl$_3$)
2.28 2.08 0.97 0.95 1.00 1.00 1.90 1.04 1.12 1.35 1.29 1.13 1.16
f1 (ppm)

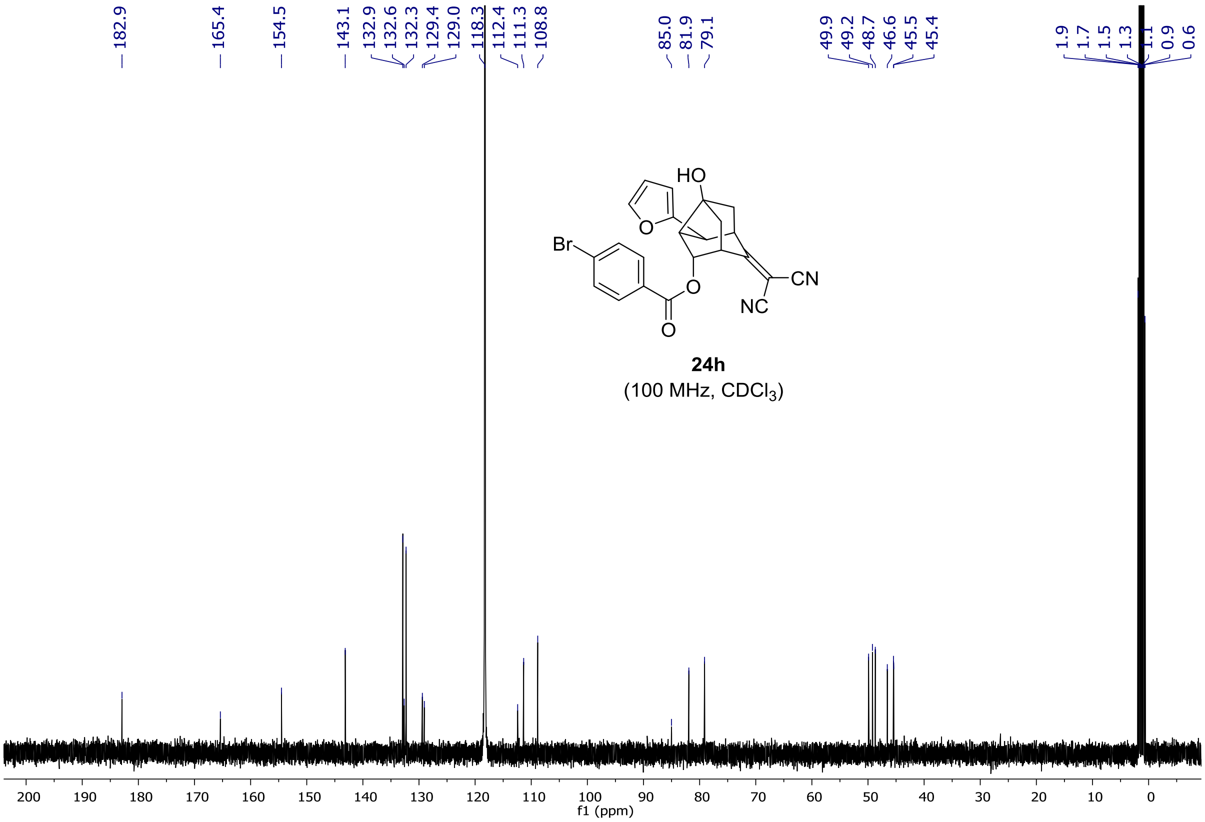
182.9 165.4 154.5 143.1 132.9 132.6 132.3 129.4 129.0 118.3 112.4 111.3 108.8
85.0 81.9 79.1
49.9 49.2 48.7 46.6 45.5 45.4
1.9 1.7 1.5 1.3 1.1 0.9 0.6
HO
O
Br
O
CN
NC
O
24h
(100 MHz, CDCl$_3$)
f1 (ppm)

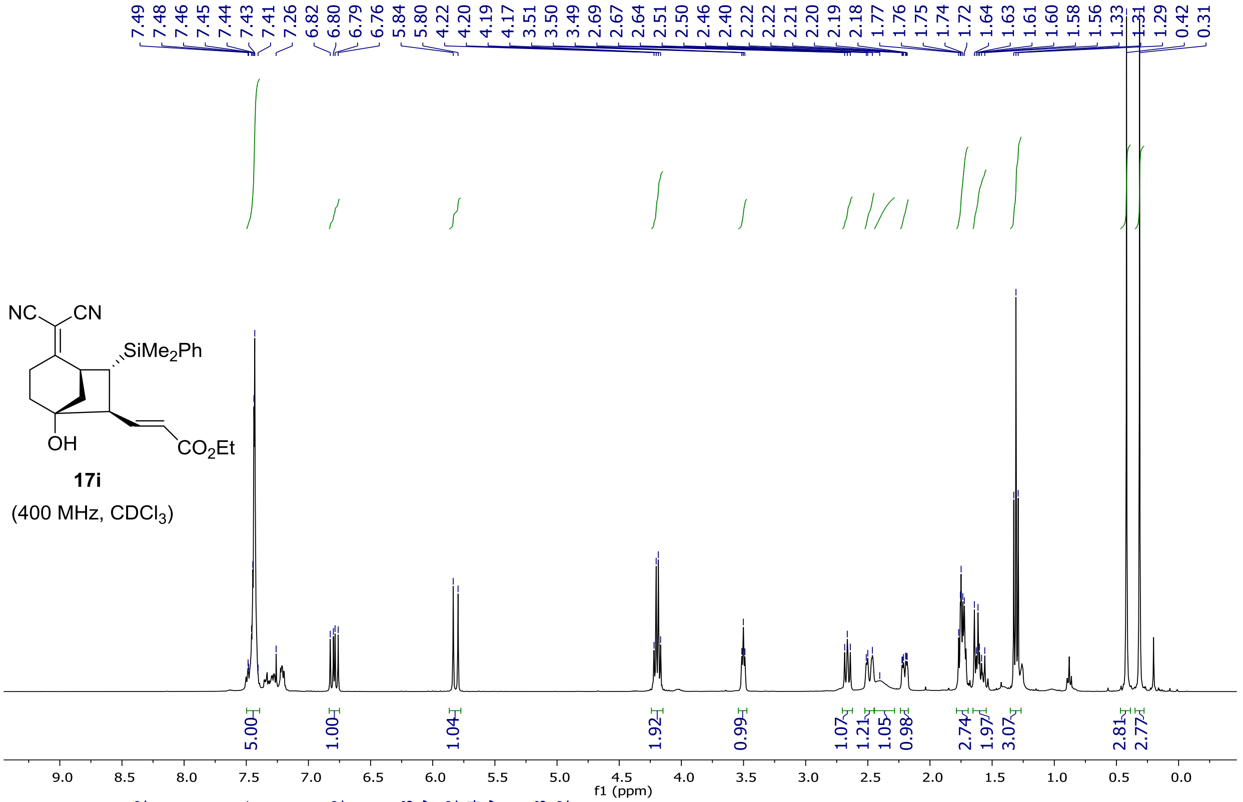

17i

(400 MHz, $CDCl_3$)

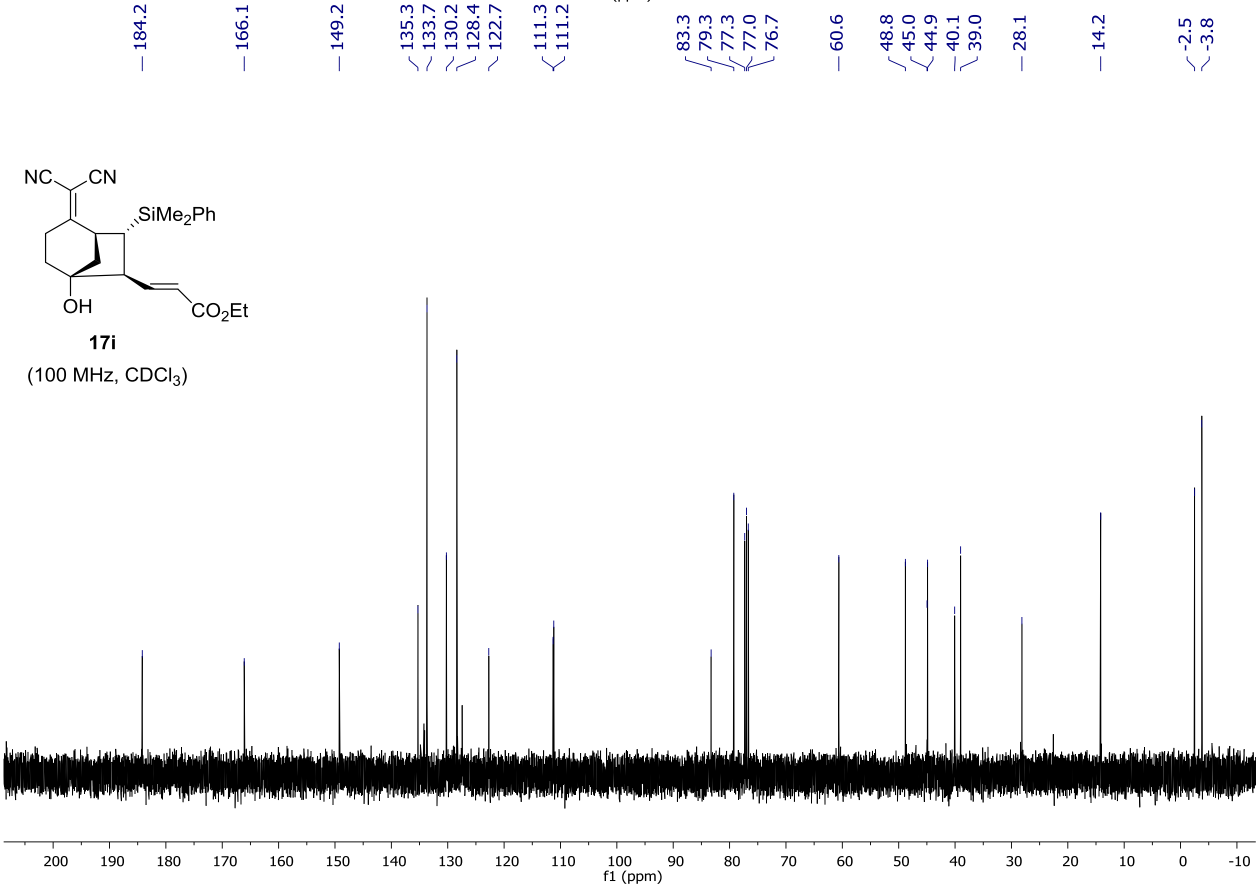

17i

(100 MHz, $CDCl_3$)

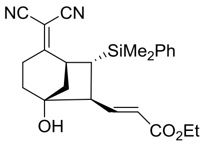

17i
COSY

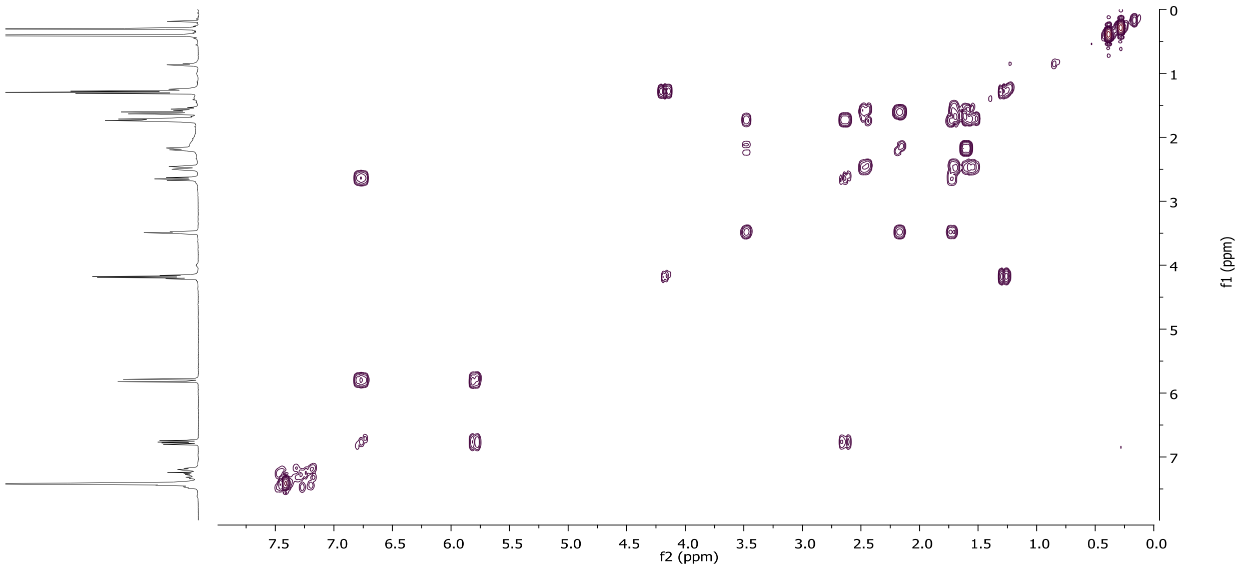

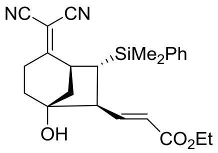

17i
NOESY

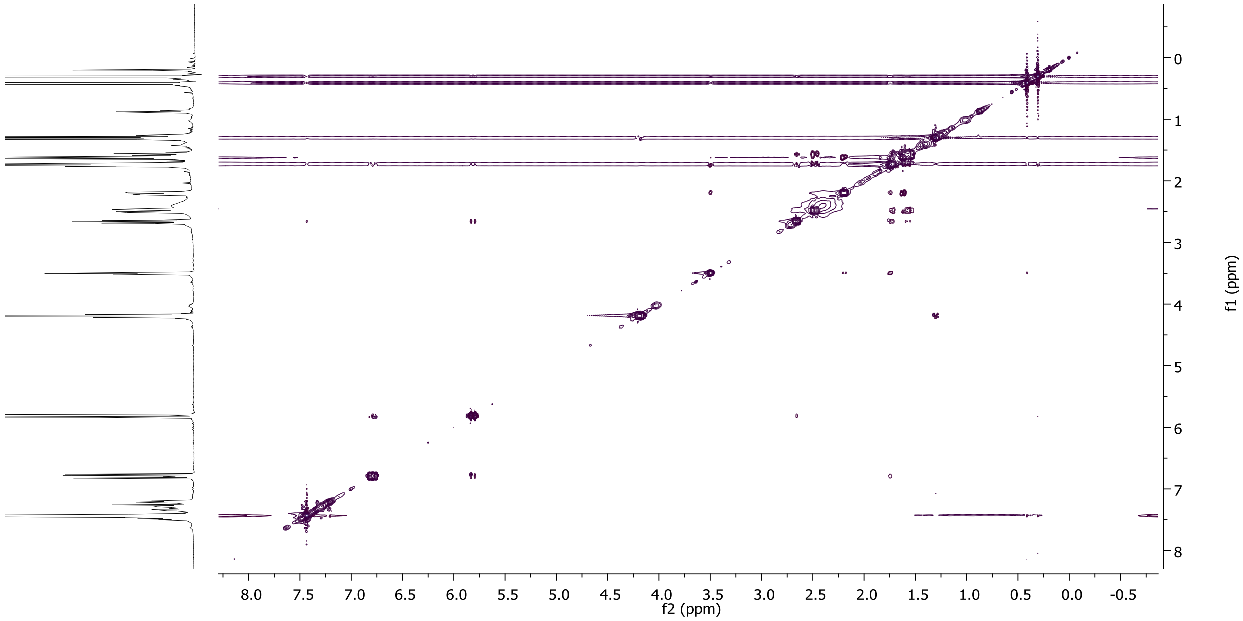

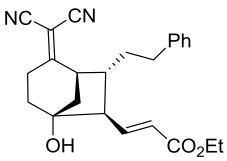

17j
(400 MHz, $CDCl_3$)

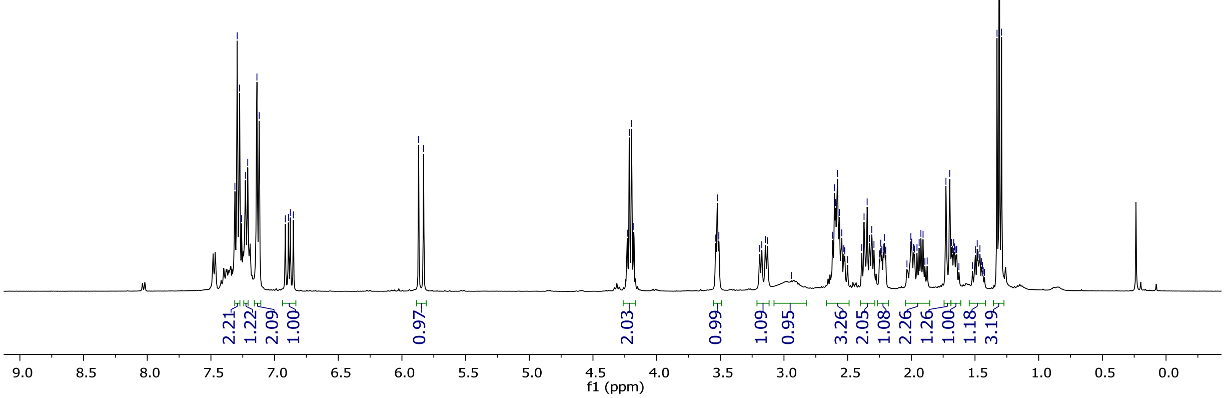

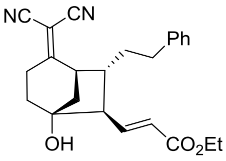

17j
(100 MHz, $CDCl_3$)

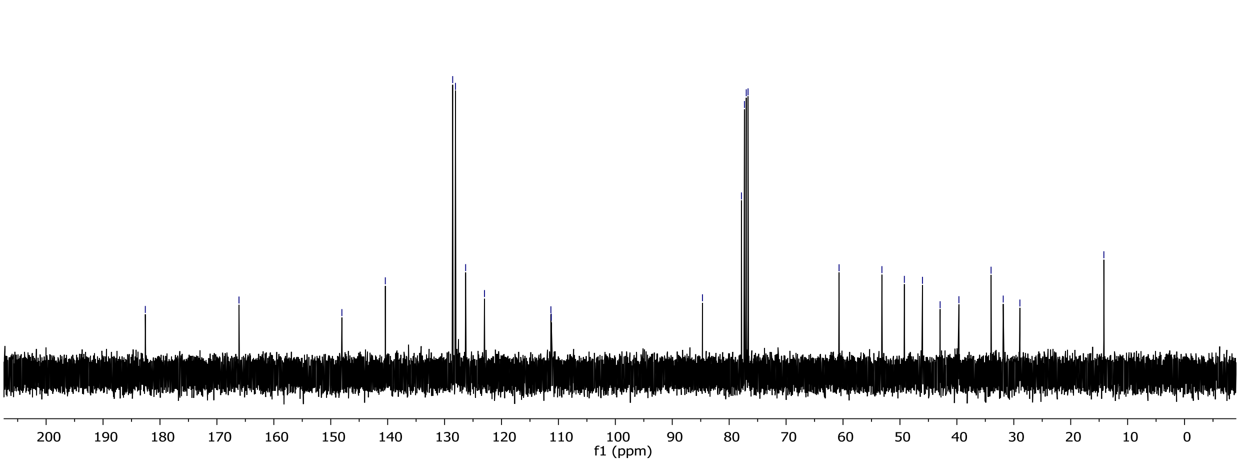

6. IR spectral data

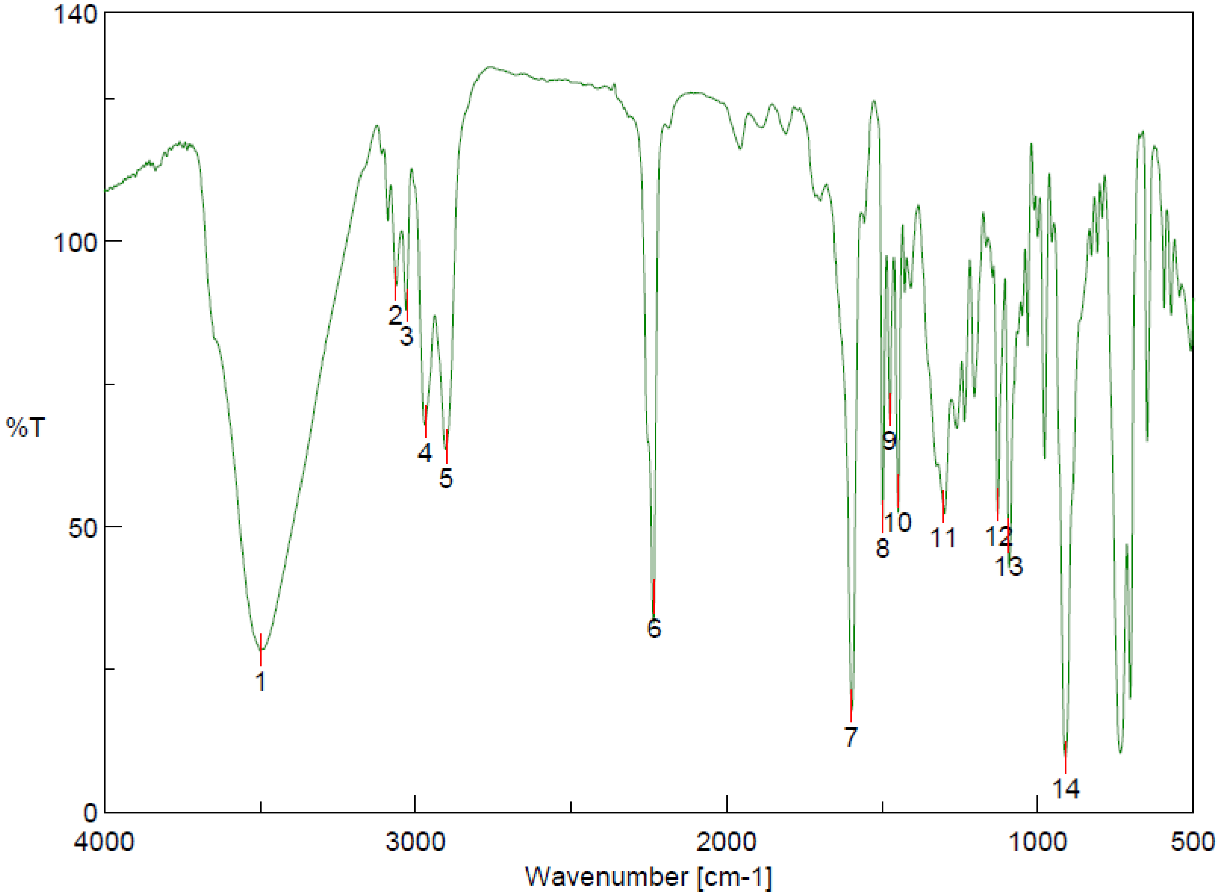

[ピーク検出結果]

No.	位置	強度	No.	位置	強度
1	3498.24	28.2678	2	3063.37	92.3386
3	3027.69	88.8309	4	2967.91	68.3192
5	2900.41	63.9862	6	2232.2	37.7308
7	1599.66	18.5289	8	1497.45	51.6815
9	1476.24	70.435	10	1451.17	56.146
11	1302.68	53.5311	12	1126.22	53.7852
13	1094.4	48.368	14	910.236	9.60326

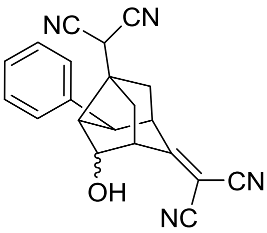

3a, 3a'

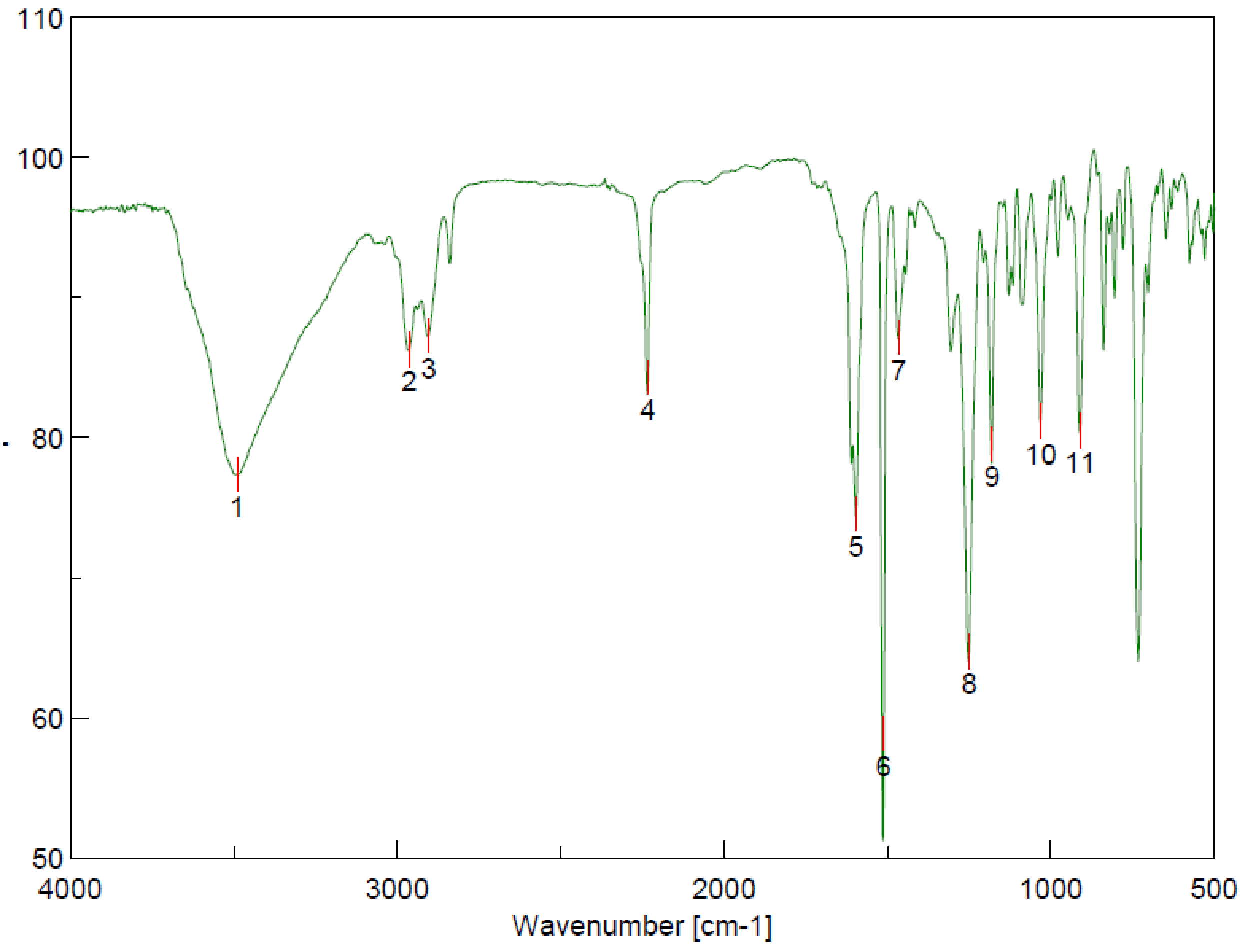

[ピーク検出結果]

No.	位置	強度	No.	位置	強度
1	3490.53	77.3437	2	2964.05	86.2714
3	2904.27	87.2365	4	2232.2	84.3079
5	1595.81	74.5682	6	1510.95	58.8484
7	1465.63	87.0857	8	1249.65	64.7125
9	1179.26	79.5619	10	1030.77	81.1652
11	910.236	80.495			

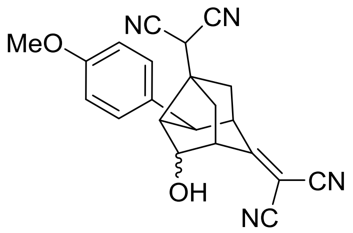

3b, 3b'

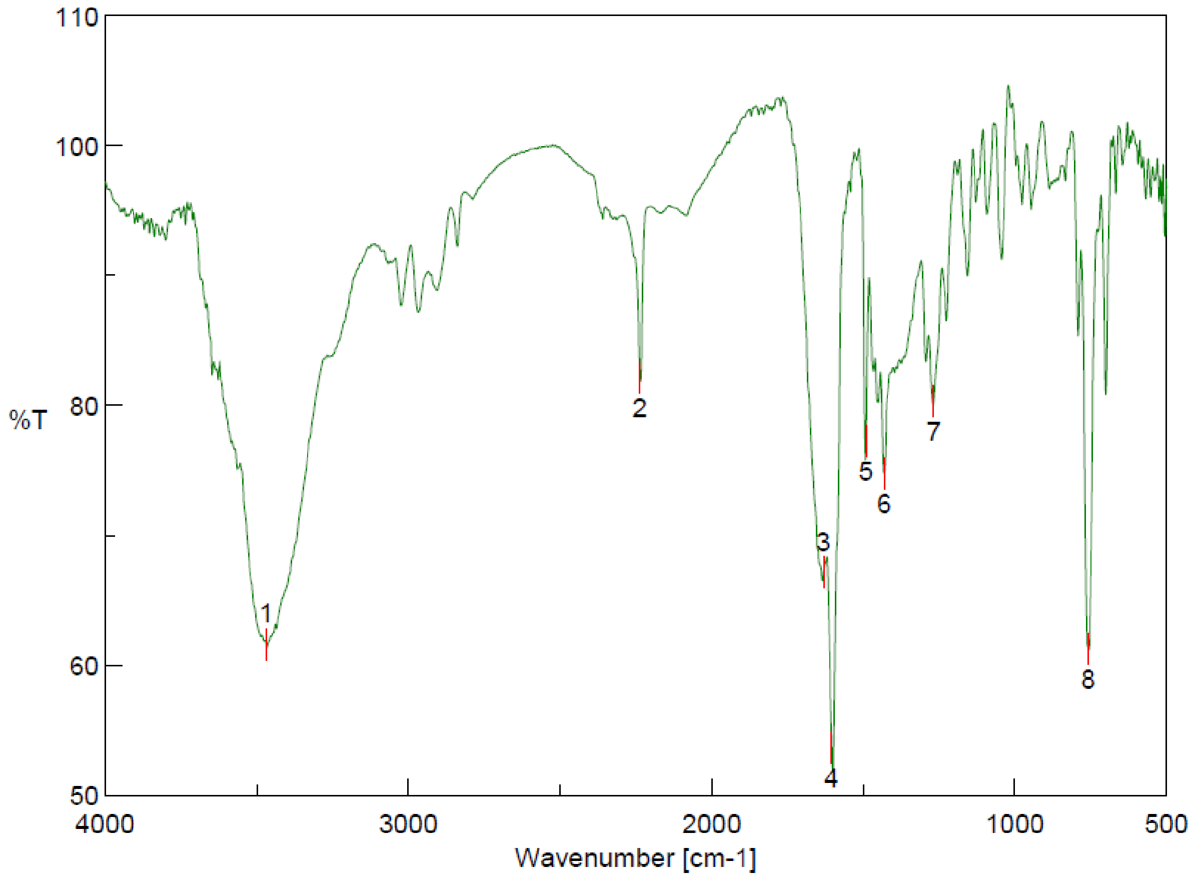

[ピーク検出結果]

No.	位置	強度	No.	位置	強度
1	3469.31	61.6431	2	2236.06	82.1365
3	1631.48	67.1308	4	1603.52	53.6979
5	1489.74	77.2261	6	1429.96	74.7829
7	1267	80.342	8	757.888	61.2965

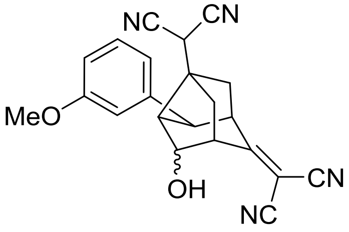

3c, 3c'

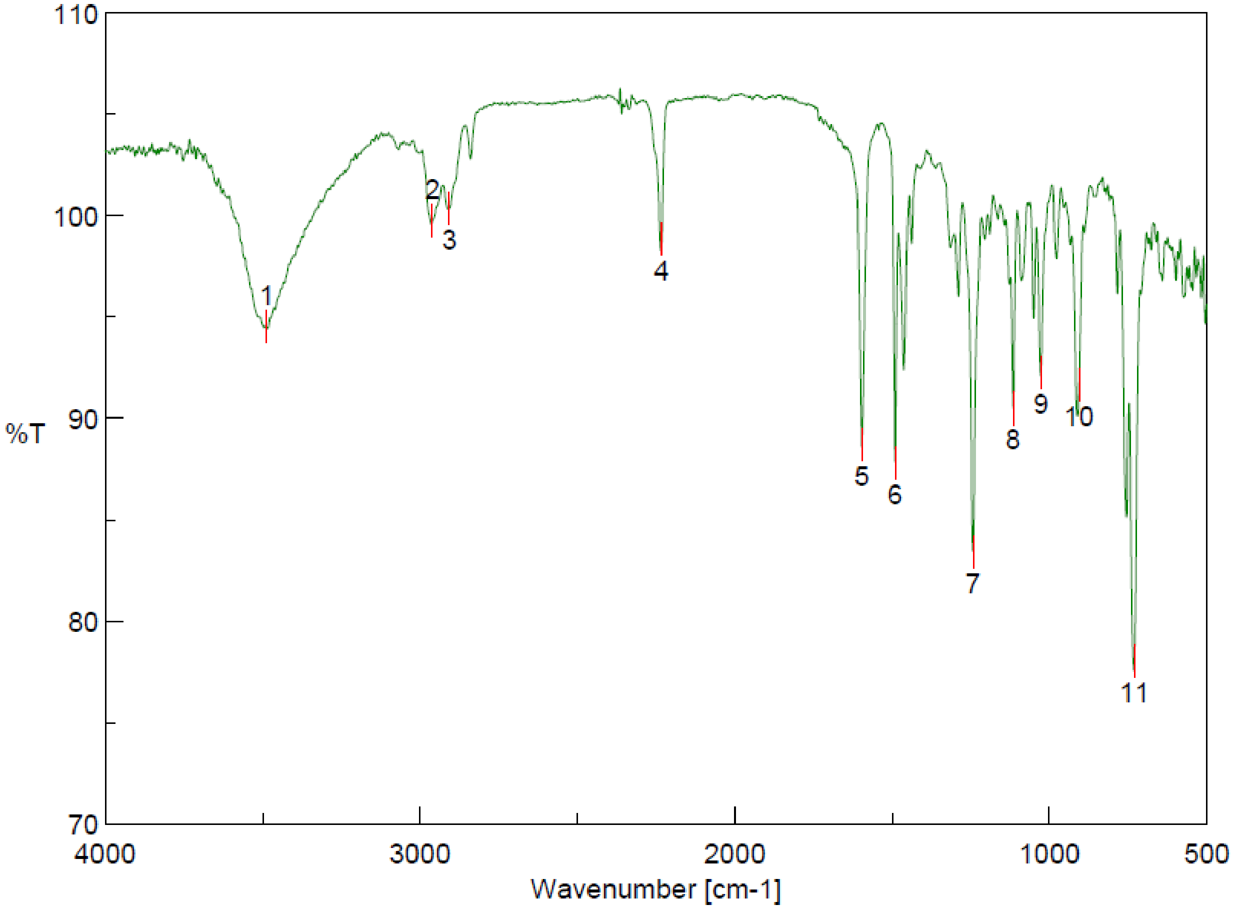

[ピーク検出結果]

No.	位置	強度	No.	位置	強度
1	3487.63	94.5102	2	2960.2	99.732
3	2907.16	100.326	4	2232.2	98.8326
5	1595.81	88.7236	6	1489.74	87.8053
7	1242.9	83.4233	8	1115.62	90.471
9	1026.91	92.2718	10	906.379	91.6341
11	729.925	78.0536			

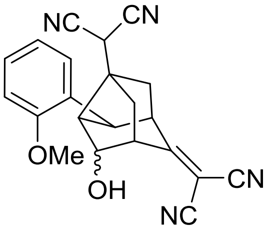

3d, 3d'

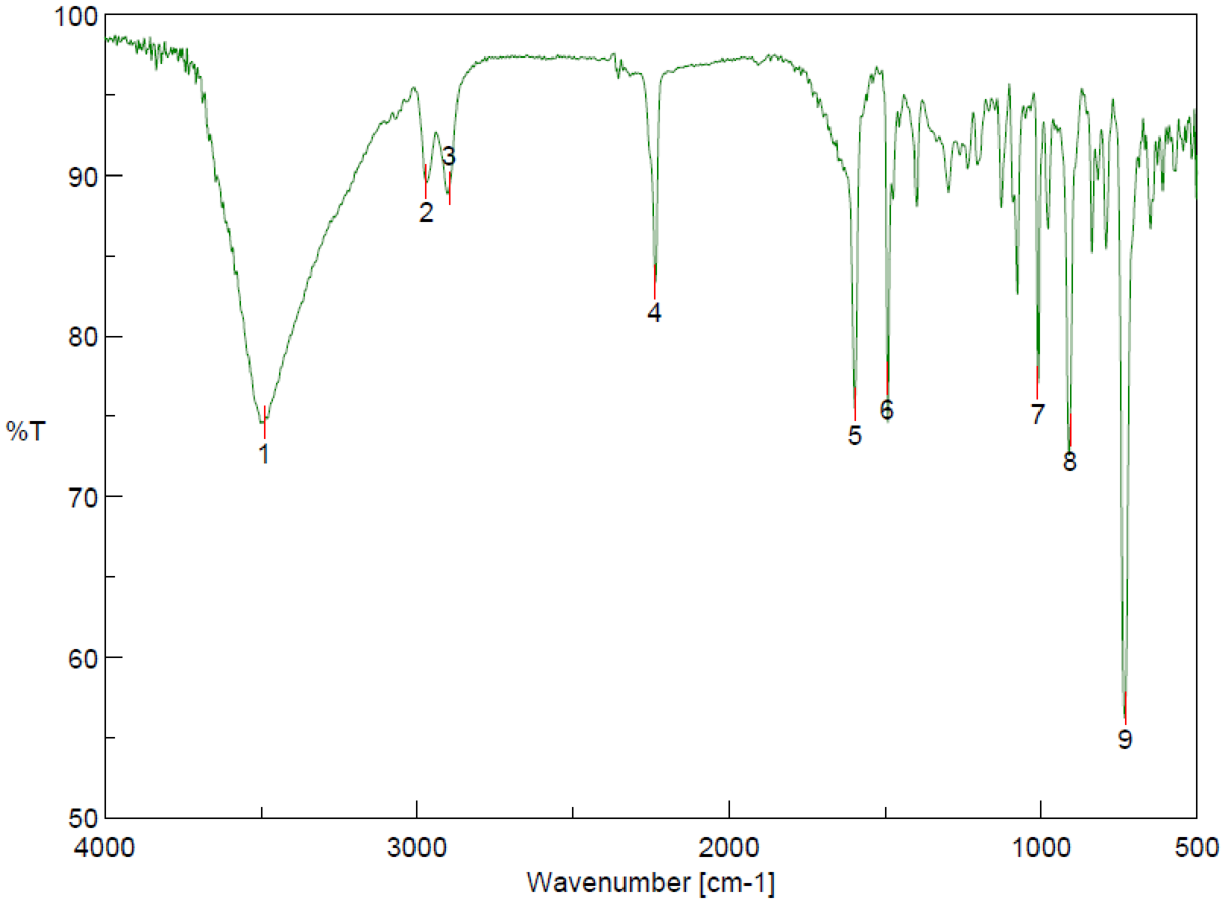

[ピーク検出結果]

No.	位置	強度	No.	位置	強度
1	3490.53	74.5917	2	2970.8	89.5906
3	2896.56	89.2058	4	2236.06	83.3586
5	1595.81	75.723	6	1493.6	77.3678
7	1009.55	77.0503	8	906.379	74.1459
9	729.925	56.7737			

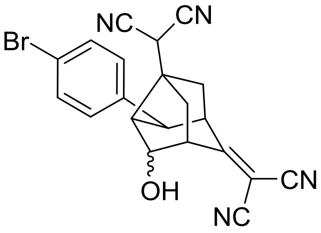

3e, 3e'

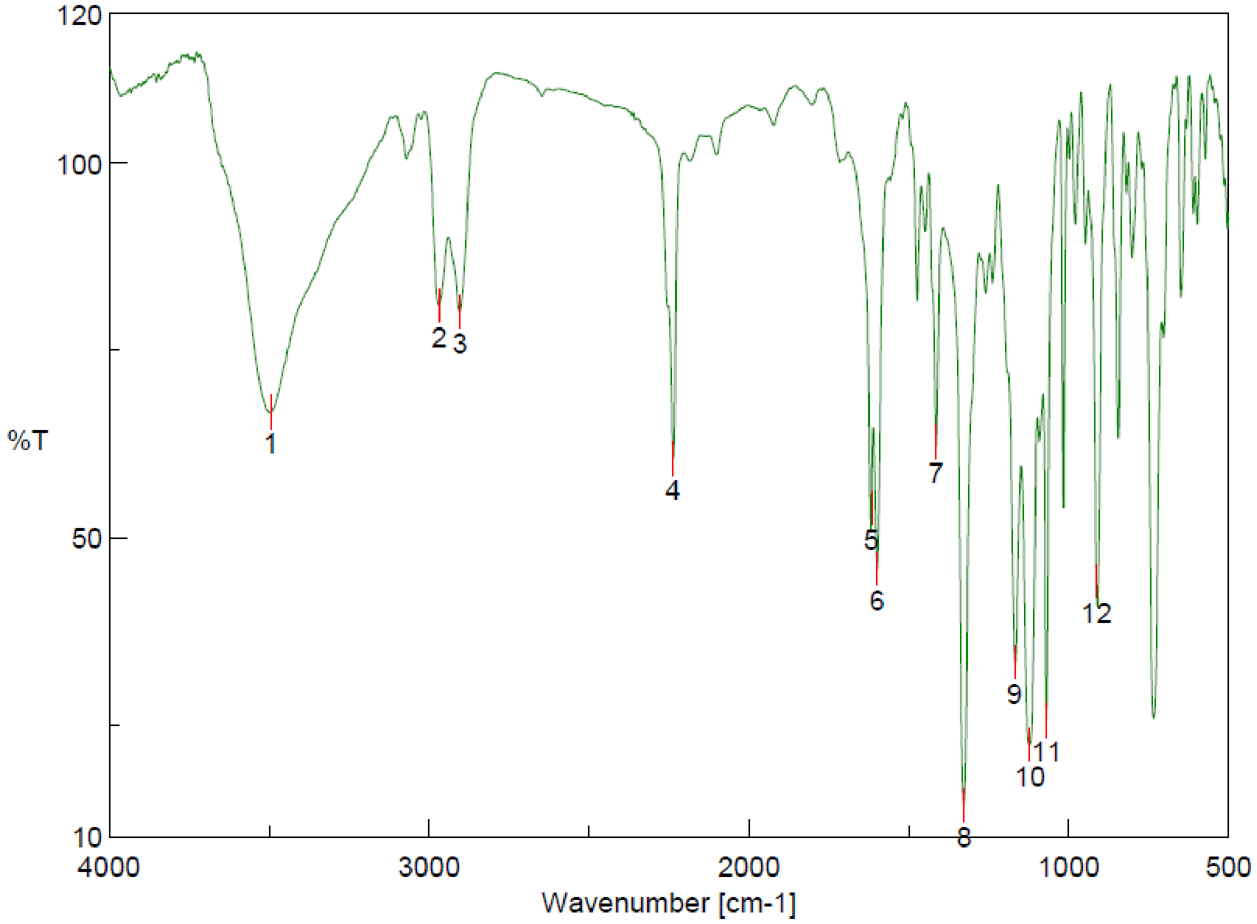

[ピーク検出結果]

No.	位置	強度	No.	位置	強度
1	3494.38	66.7254	2	2967.91	81.0283
3	2904.27	80.1127	4	2236.06	60.4984
5	1617.02	53.995	6	1599.66	45.8865
7	1415.49	62.7907	8	1327.75	14.1339
9	1168.65	33.3841	10	1122.37	22.3068
11	1069.33	25.5295	12	914.093	44.1325

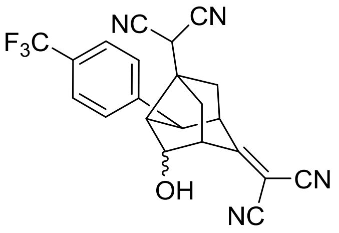

3f, 3f'

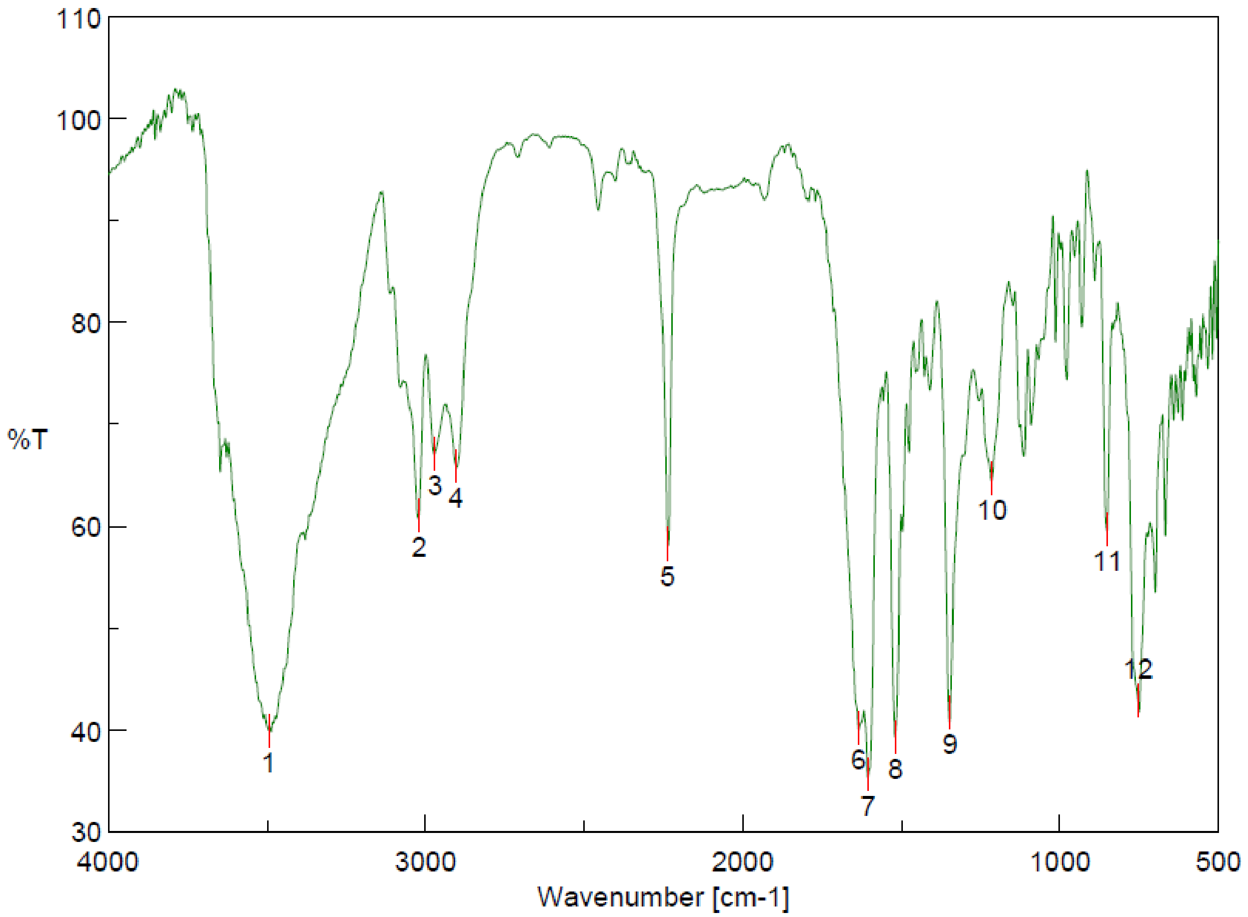

[ピーク検出結果]

No.	位置	強度	No.	位置	強度
1	3494.38	39.9185	2	3020.94	61.0061
3	2970.8	67.1466	4	2904.27	65.8497
5	2236.06	58.2156	6	1635.34	40.1429
7	1603.52	35.6409	8	1518.67	39.3013
9	1345.11	41.7082	10	1213.97	64.6612
11	850.454	59.7284	12	754.995	42.8648

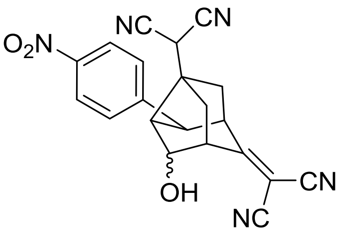

3g, 3g'

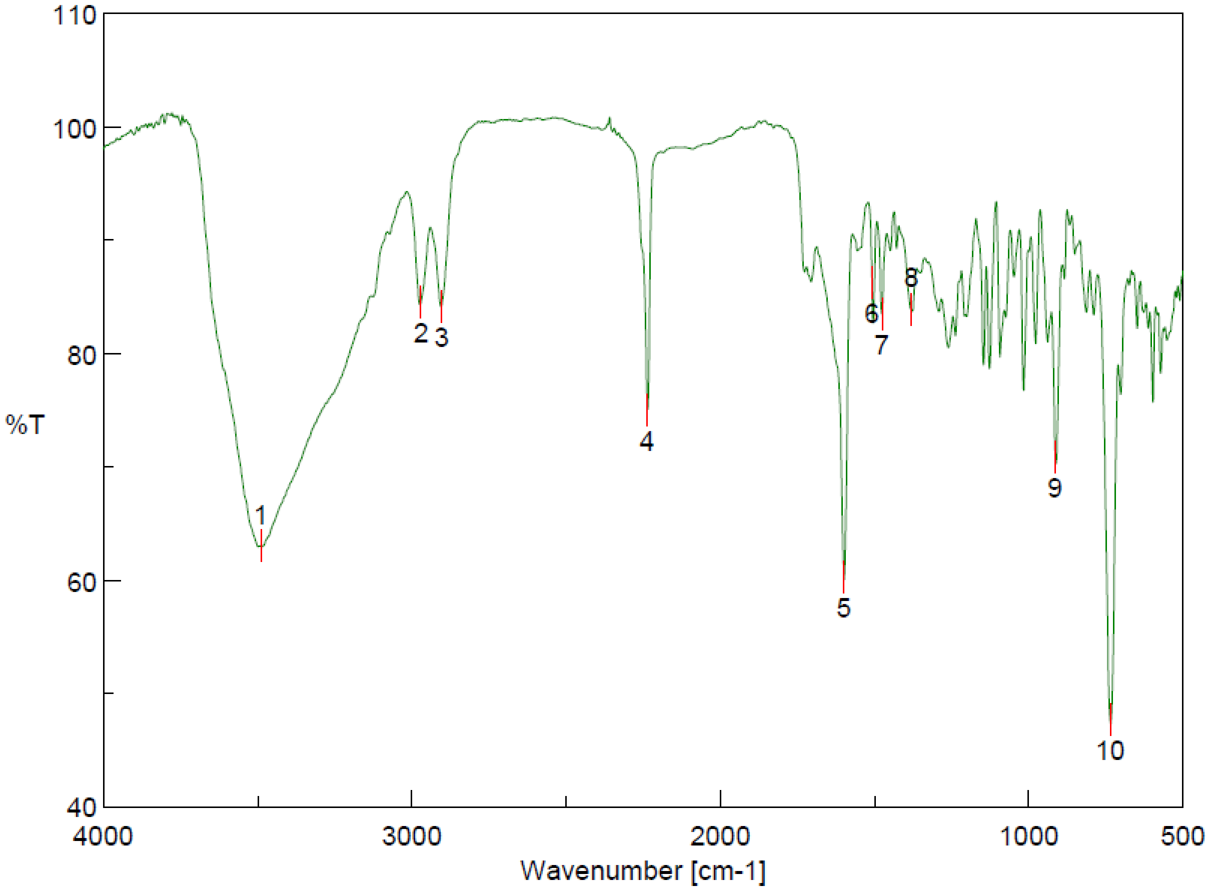

[ピーク検出結果]

No.	位置	強度	No.	位置	強度
1	3490.53	63.0112	2	2970.8	84.4402
3	2904.27	84.139	4	2236.06	75.0272
5	1599.66	60.2629	6	1508.06	86.1732
7	1476.24	83.425	8	1380.78	83.8445
9	914.093	70.8742	10	736.674	47.6923

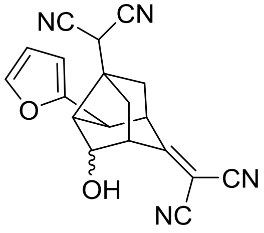

3h, 3h'

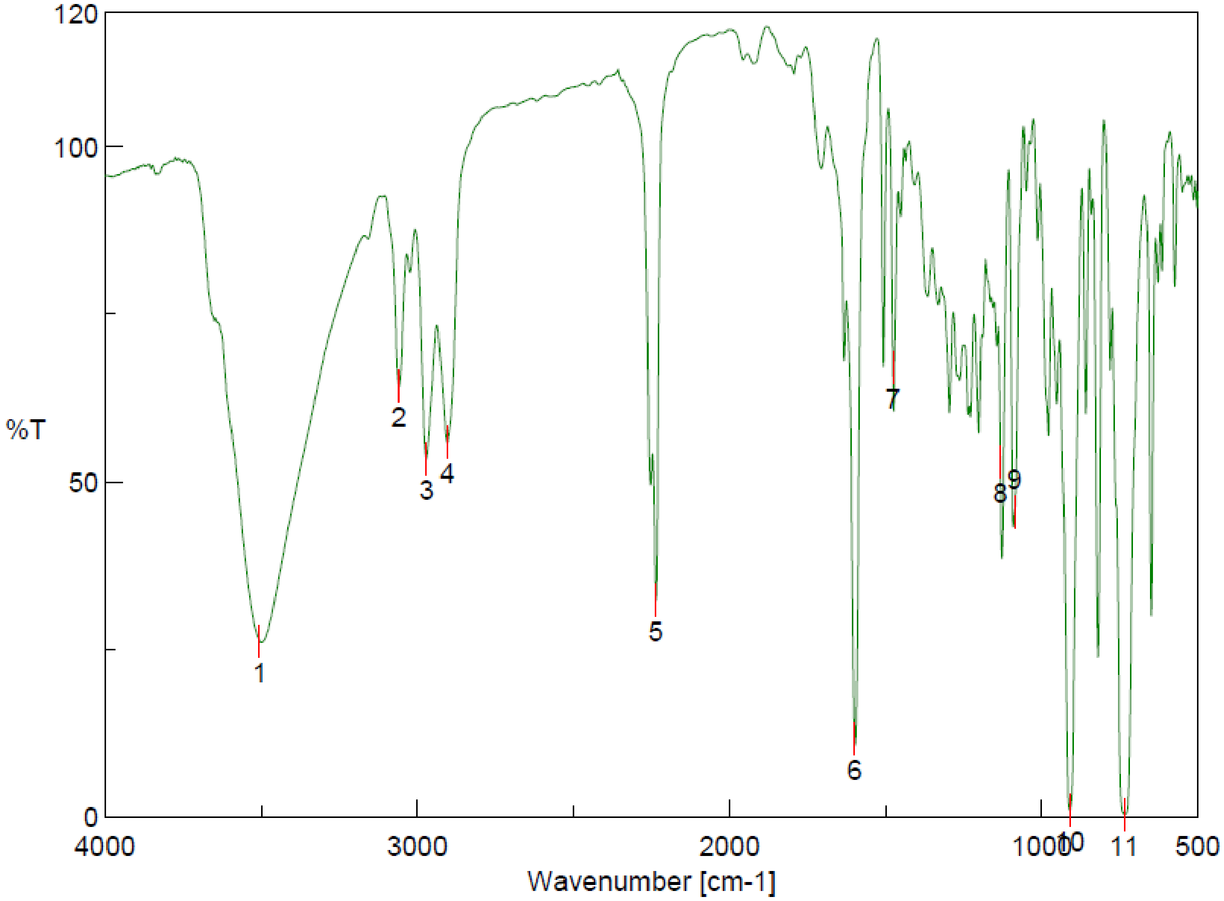

[ピーク検出結果]

No.	位置	強度	No.	位置	強度
1	3504.99	26.2364	2	3059.51	64.2385
3	2970.8	53.3515	4	2904.27	55.78
5	2236.06	32.2307	6	1599.66	11.6305
7	1476.24	66.9353	8	1132.97	52.9961
9	1086.69	45.4547	10	910.236	0.963325
11	736.674	0.35362			

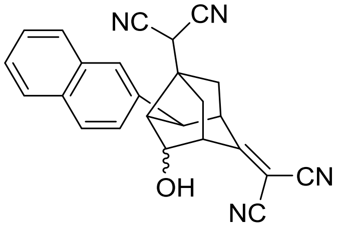

3i, 3i'

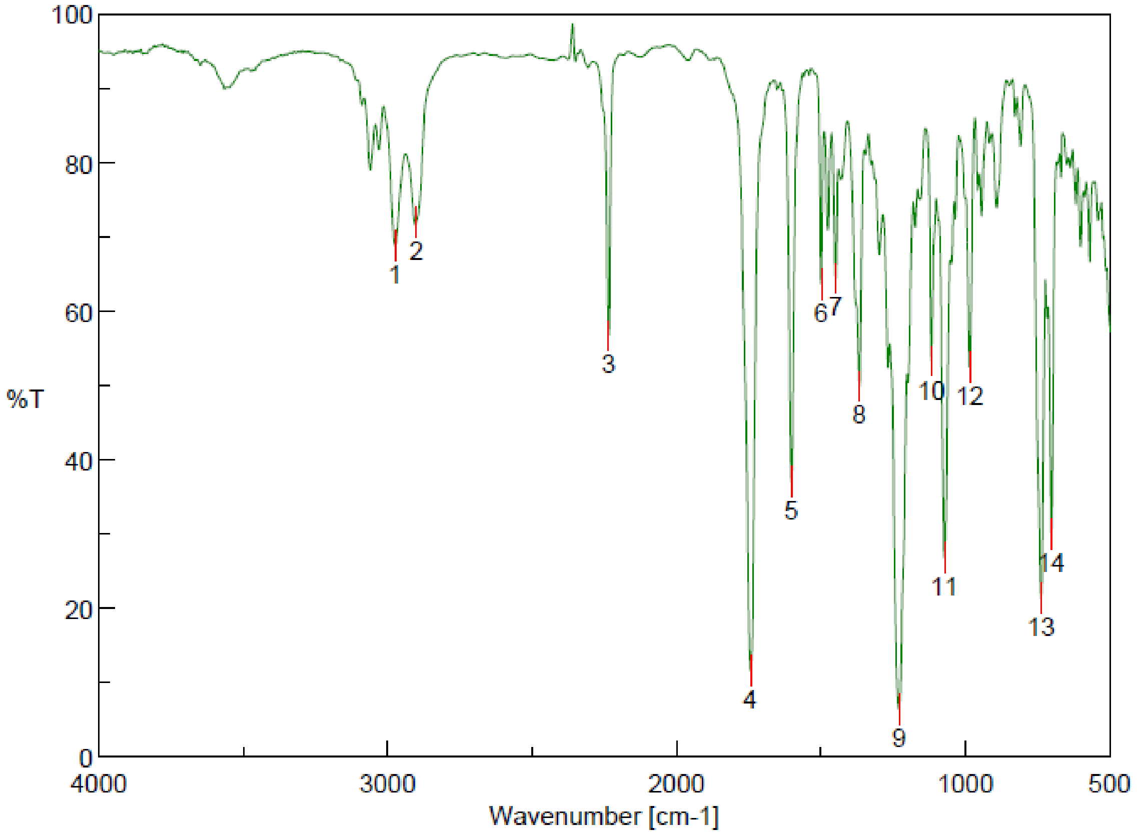

[ピーク検出結果]

No.	位置	強度	No.	位置	強度
1	2974.66	68.7951	2	2900.41	71.8985
3	2235.09	56.6976	4	1743.33	11.6102
5	1602.56	36.9963	6	1498.42	63.6449
7	1449.24	64.363	8	1367.28	49.8635
9	1229.4	6.34712	10	1117.55	53.21
11	1071.26	26.7963	12	984.482	52.4229
13	737.639	21.323	14	701.962	29.9762

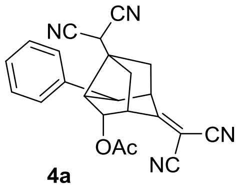

4a

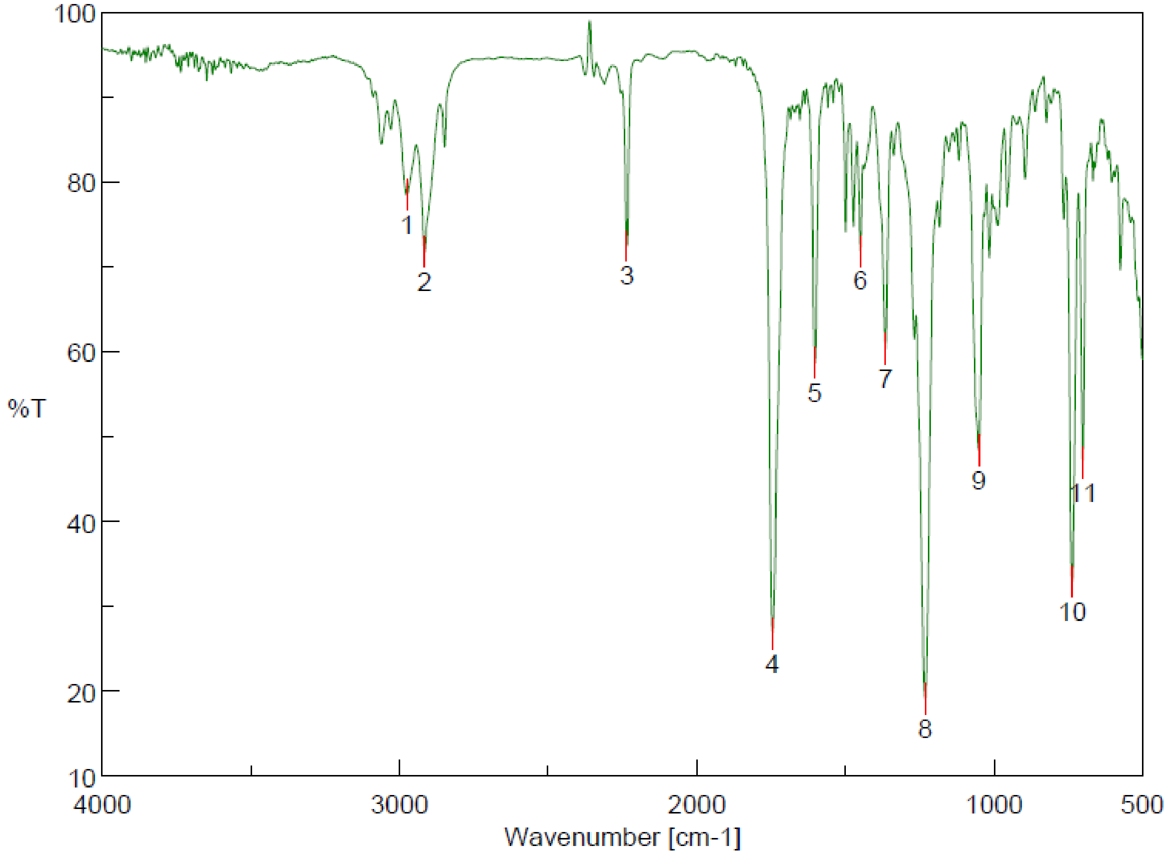

[ピーク検出結果]

No.	位置	強度	No.	位置	強度
1	2974.66	78.4916	2	2915.84	71.7129
3	2235.09	72.4017	4	1744.3	26.7582
5	1602.56	58.6621	6	1449.24	71.8176
7	1365.35	60.2861	8	1231.33	19.0556
9	1051.01	48.3971	10	736.674	32.9249
11	700.998	46.9275			

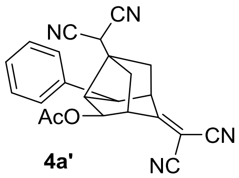

4a'

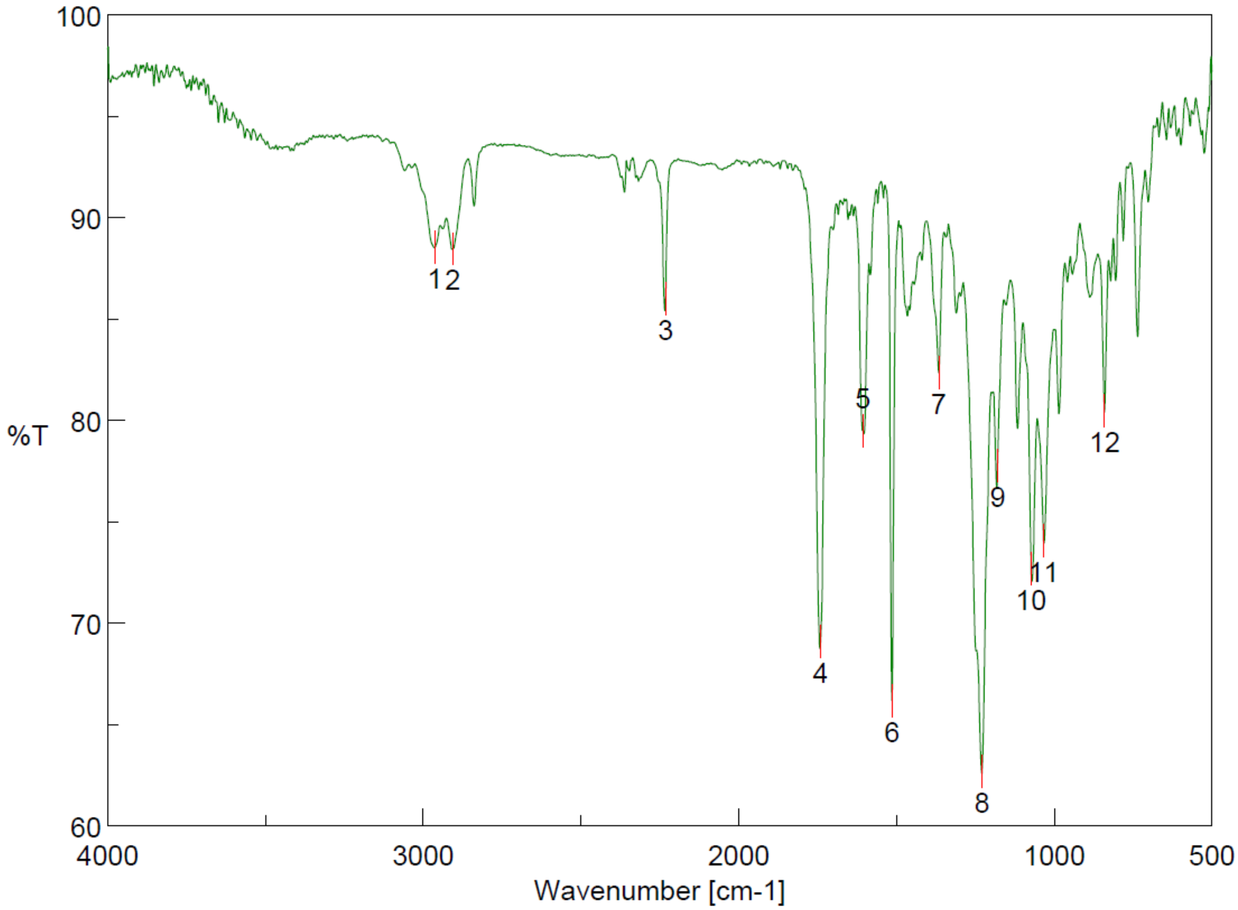

[ピーク検出結果]

No.	位置	強度	No.	位置	強度
1	2964.05	88.5162	2	2907.16	88.4406
3	2232.2	85.9976	4	1740.44	69.0782
5	1606.41	79.4933	6	1514.81	66.18
7	1366.32	82.3381	8	1228.43	62.6827
9	1179.26	77.7555	10	1073.19	72.6759
11	1033.66	74.0739	12	839.847	80.476

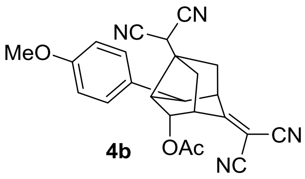

4b

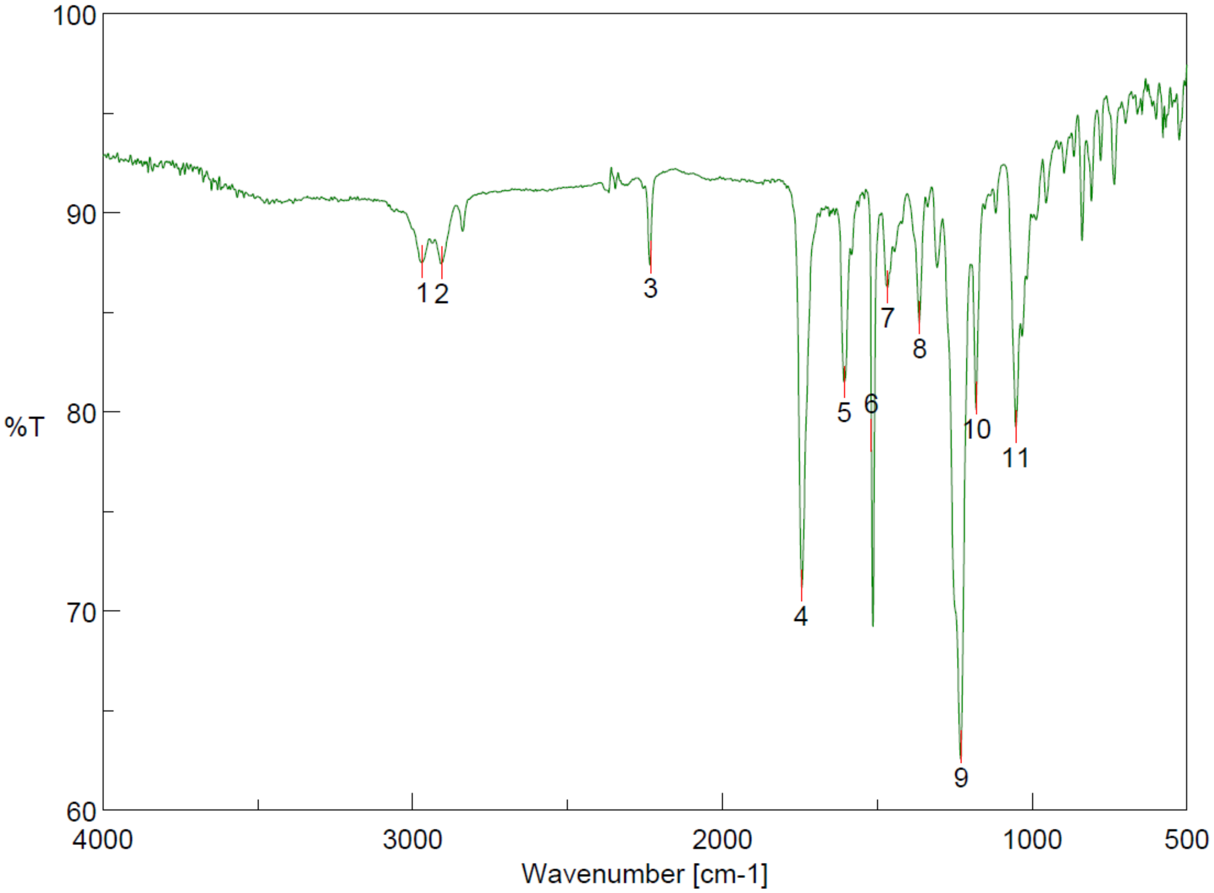

[ピーク検出結果]

No.	位置	強度	No.	位置	強度
1	2967.91	87.5137	2	2907.16	87.4599
3	2232.2	87.7665	4	1744.3	71.2692
5	1606.41	81.4889	6	1518.67	78.7998
7	1465.63	86.2776	8	1362.46	84.7303
9	1228.43	63.1743	10	1179.26	80.7046
11	1051.98	79.2324			

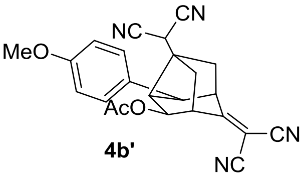

4b'

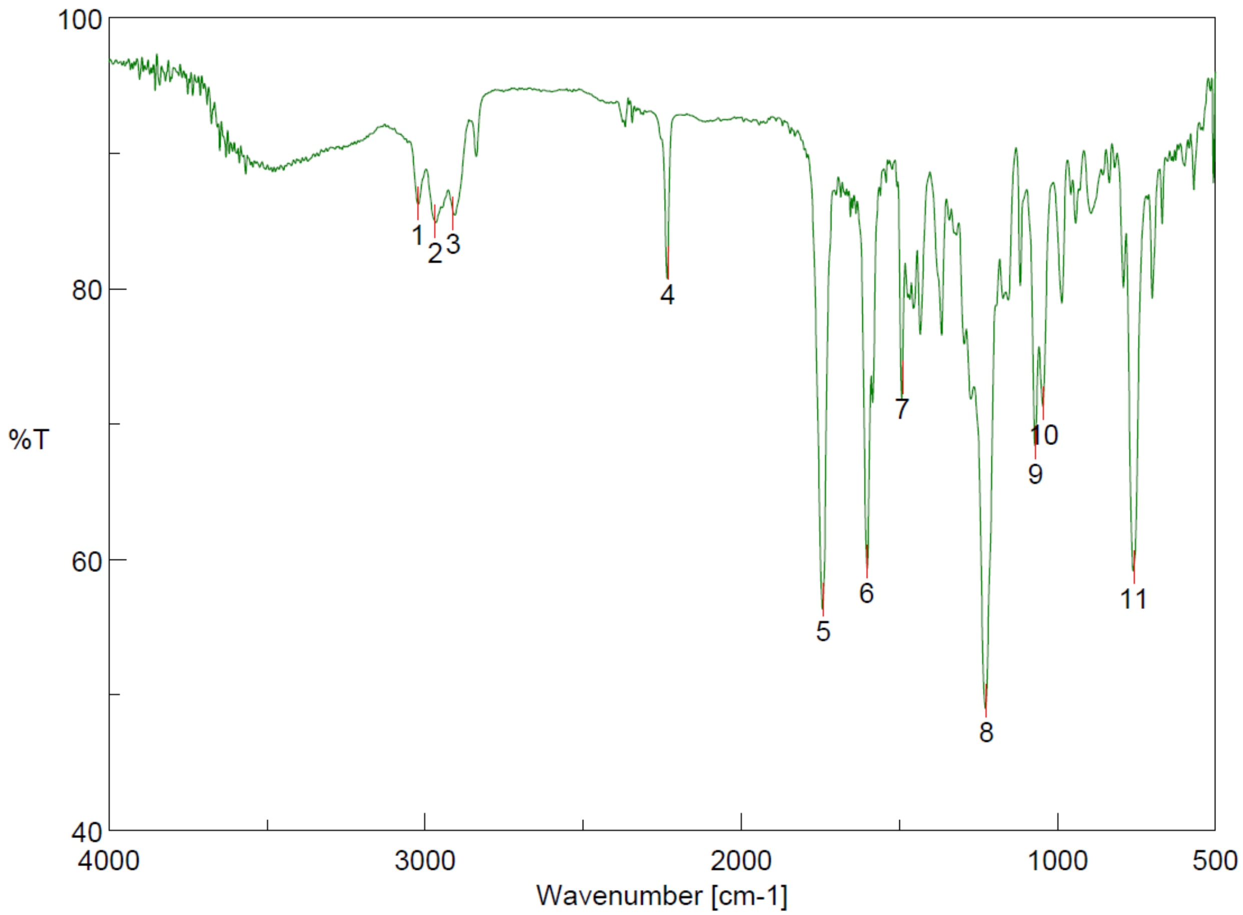

[ピーク検出結果]

No.	位置	強度	No.	位置	強度
1	3023.84	86.2484	2	2967.91	84.9078
3	2911.02	85.5747	4	2232.2	81.8648
5	1740.44	57.0516	6	1603.52	59.8686
7	1489.74	73.42	8	1224.58	49.5328
9	1069.33	68.6398	10	1044.26	71.5133
11	757.888	59.4452			

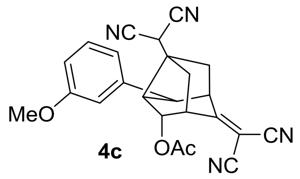

4c

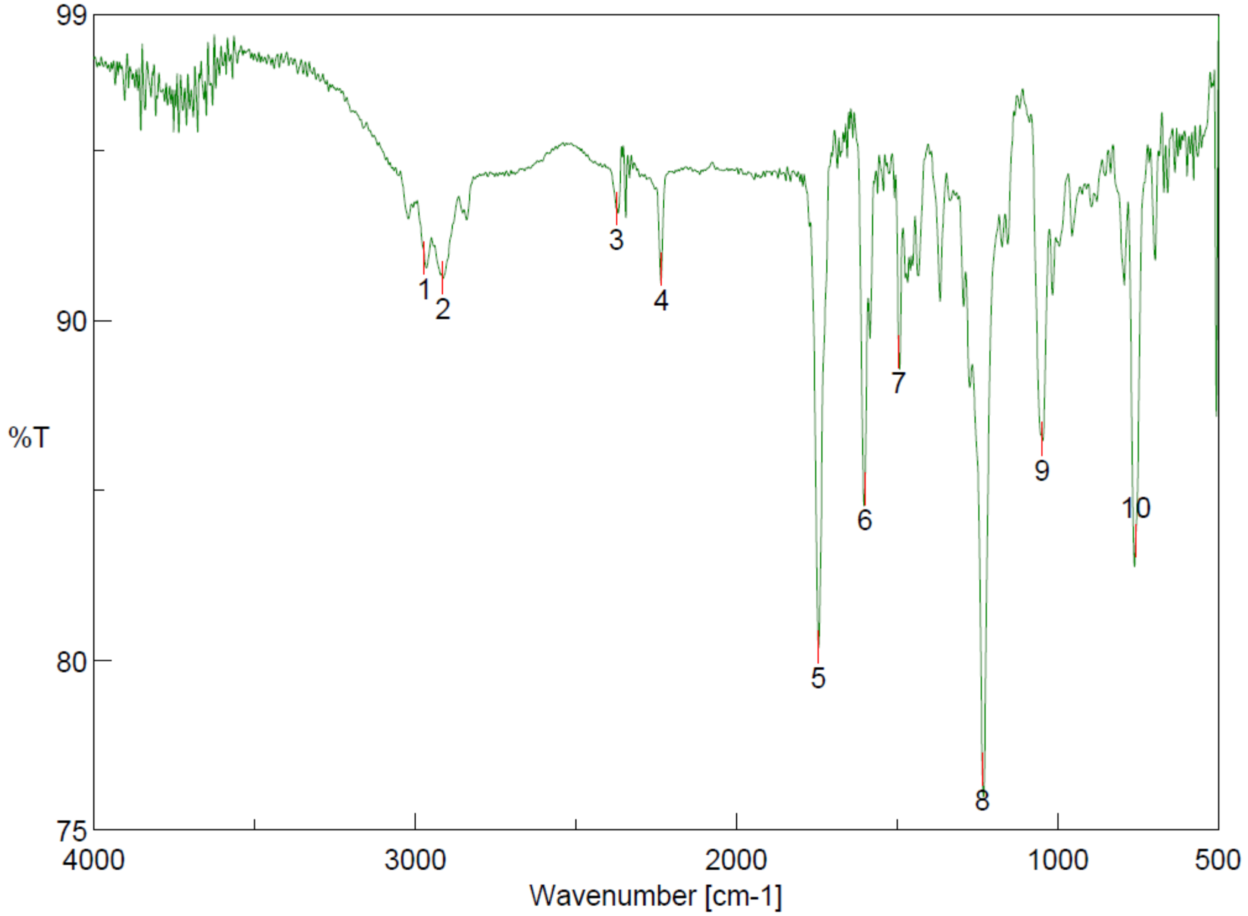

[ピーク検出結果]

No.	位置	強度	No.	位置	強度
1	2970.8	91.822	2	2913.91	91.2421
3	2372.98	93.2907	4	2232.2	91.5067
5	1744.3	80.3971	6	1599.66	85.0397
7	1494.56	89.0761	8	1234.22	76.7931
9	1048.12	86.5181	10	756.923	83.5184

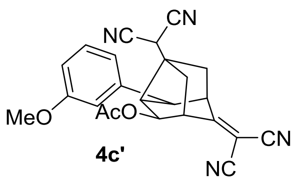

4c'

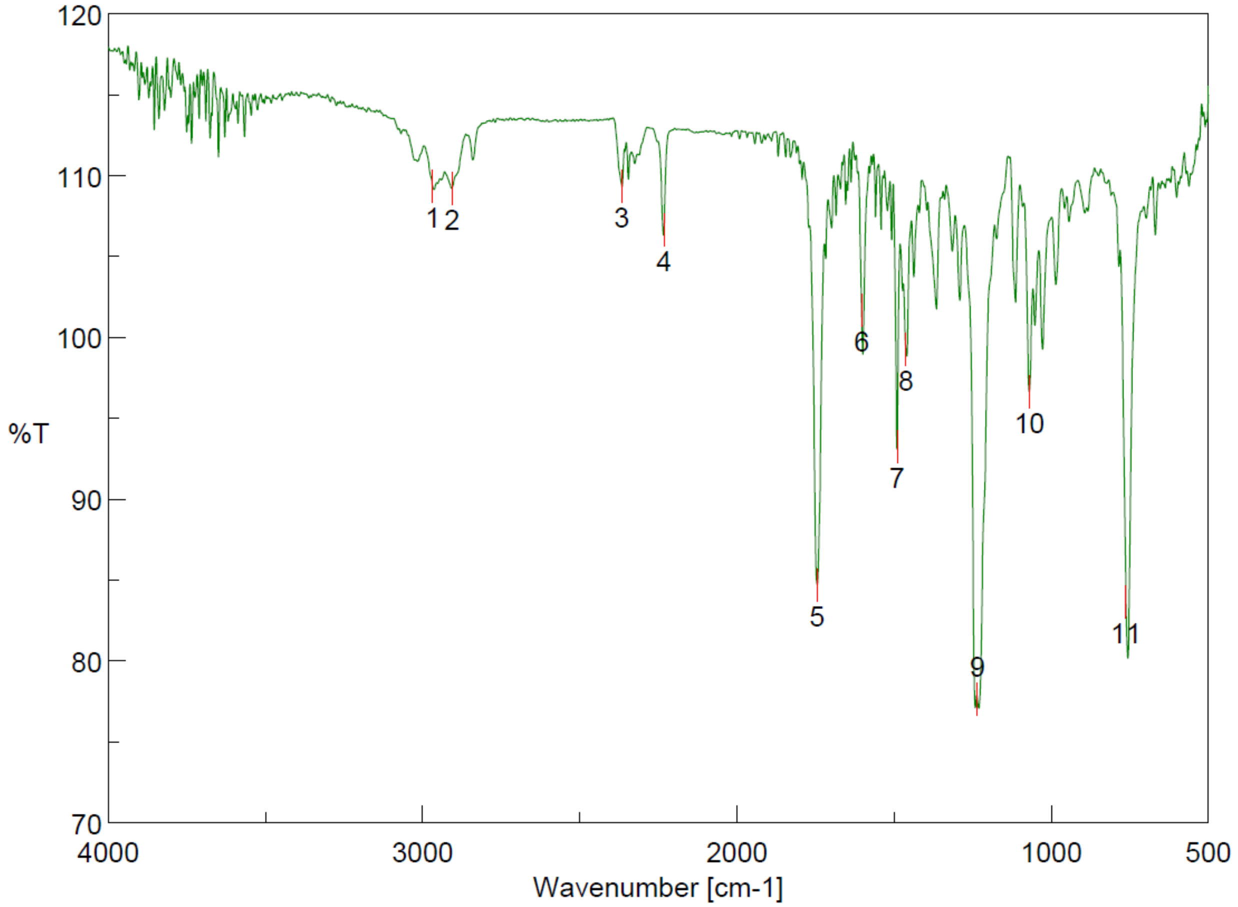

[ピーク検出結果]

No.	位置	強度	No.	位置	強度
1	2967.91	109.304	2	2907.16	109.201
3	2366.23	109.305	4	2232.2	106.642
5	1744.3	84.6854	6	1603.52	101.671
7	1489.74	93.2709	8	1461.78	99.2513
9	1235.18	77.6669	10	1069.33	96.6375
11	761.744	83.6228			

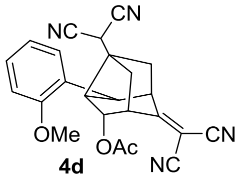

4d

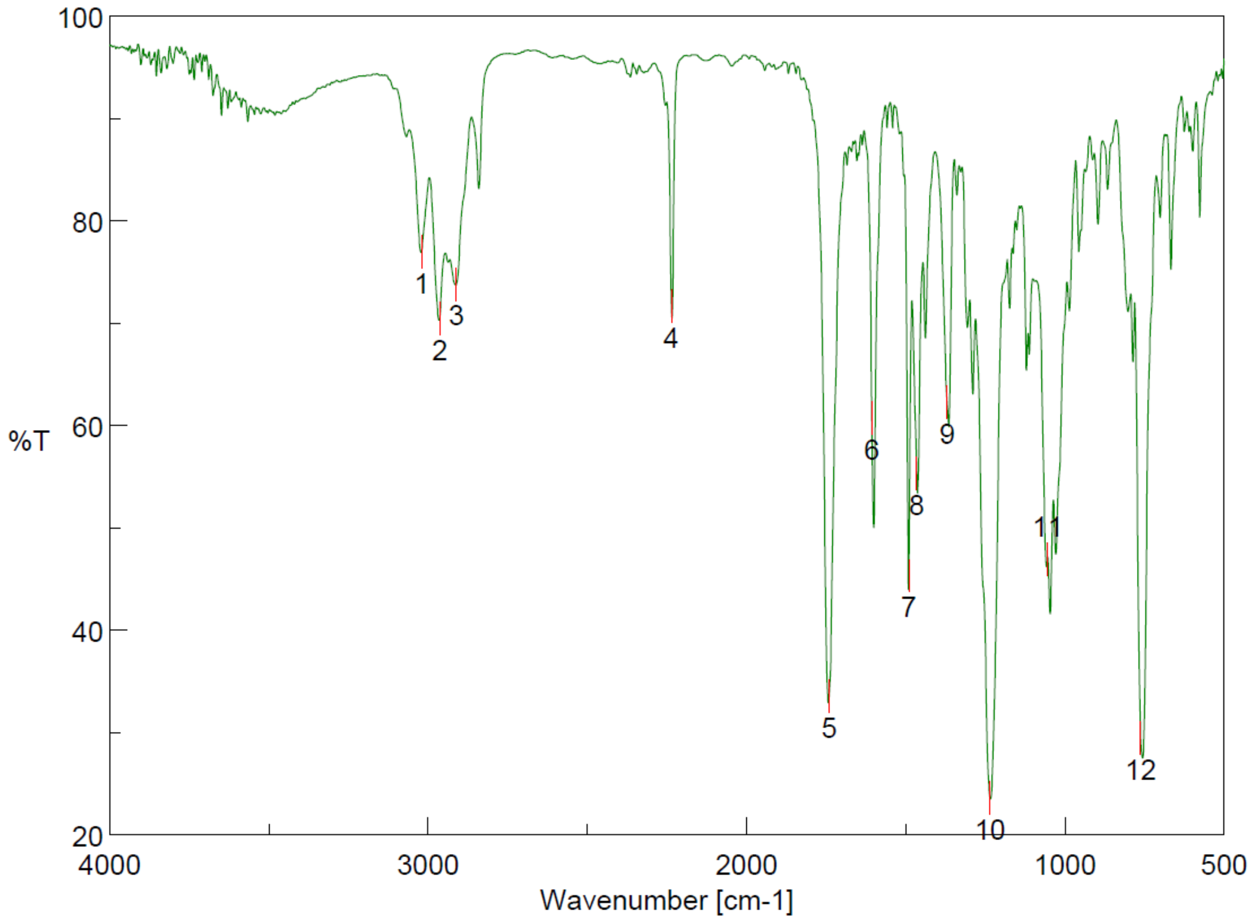

[ピーク検出結果]

No.	位置	強度	No.	位置	強度
1	3020.94	77.0098	2	2964.05	70.4506
3	2911.02	73.795	4	2236.06	71.5961
5	1740.44	33.5637	6	1606.41	60.6881
7	1489.74	45.307	8	1465.63	55.264
9	1370.18	62.2436	10	1235.18	23.5787
11	1054.87	46.9168	12	761.744	29.4201

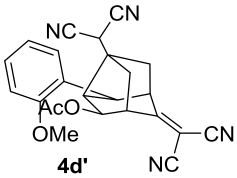

4d'

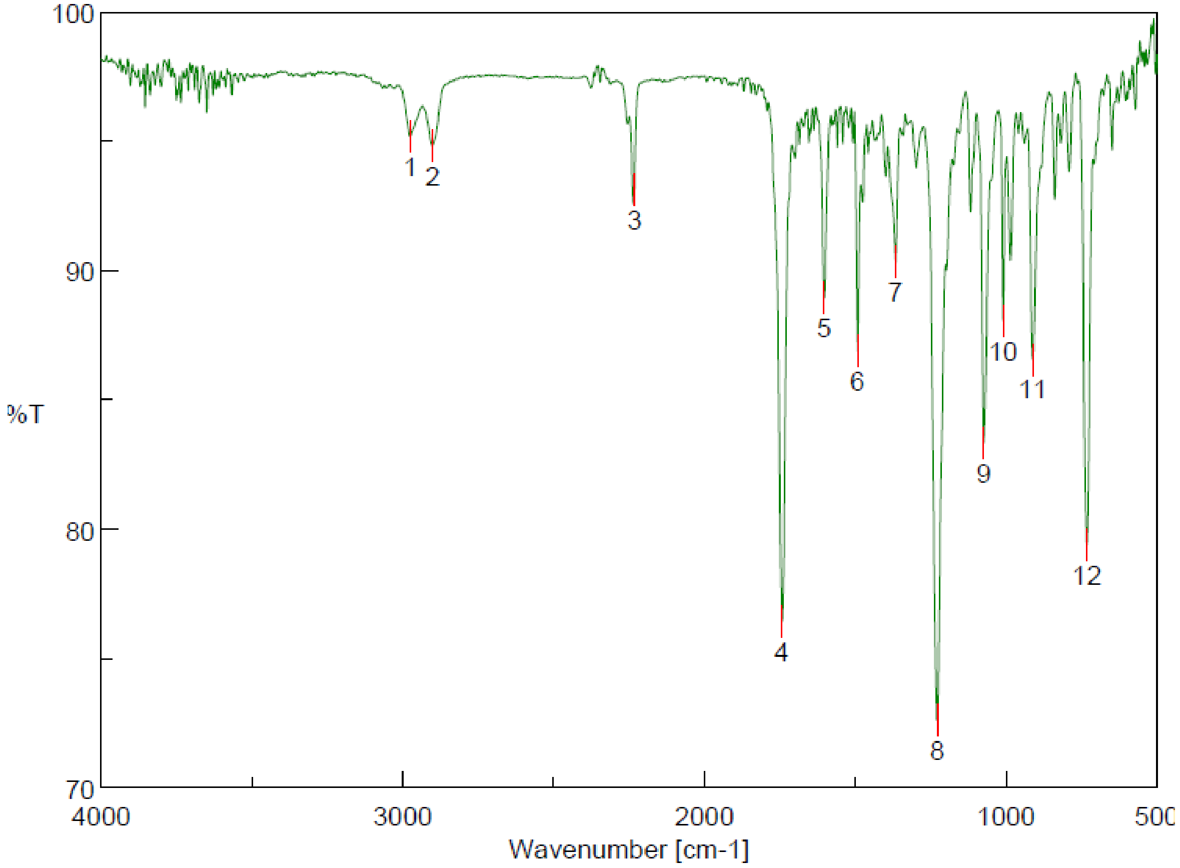

[ピーク検出結果]

No.	位置	強度	No.	位置	強度
1	2974.66	95.1903	2	2900.41	94.8456
3	2232.2	93.1193	4	1742.37	76.4378
5	1602.56	88.939	6	1491.67	86.8898
7	1366.32	90.3471	8	1227.47	72.6066
9	1073.19	83.3038	10	1009.55	88.0467
11	911.201	86.5592	12	731.853	79.3696

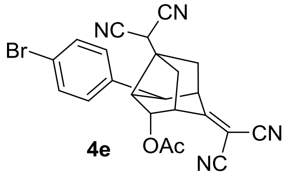

4e

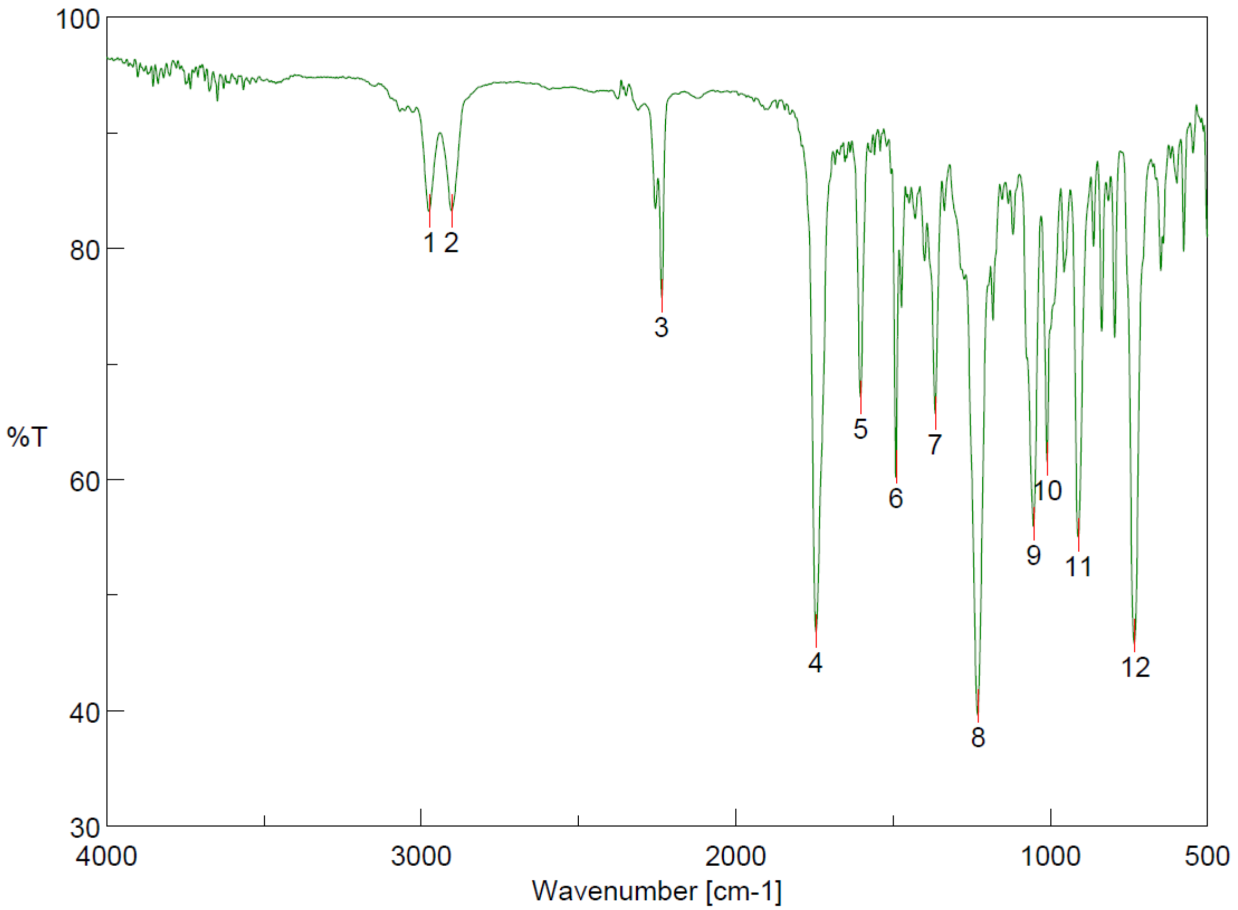

[ピーク検出結果]

No.	位置	強度	No.	位置	強度
1	2974.66	83.2459	2	2904.27	83.2274
3	2236.06	75.8476	4	1744.3	46.823
5	1603.52	67.1153	6	1489.74	61.0755
7	1366.32	65.6774	8	1228.43	40.3769
9	1051.98	56.1852	10	1009.55	61.7419
11	910.236	55.1726	12	729.925	46.4577

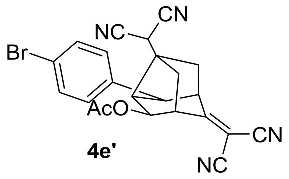

4e'

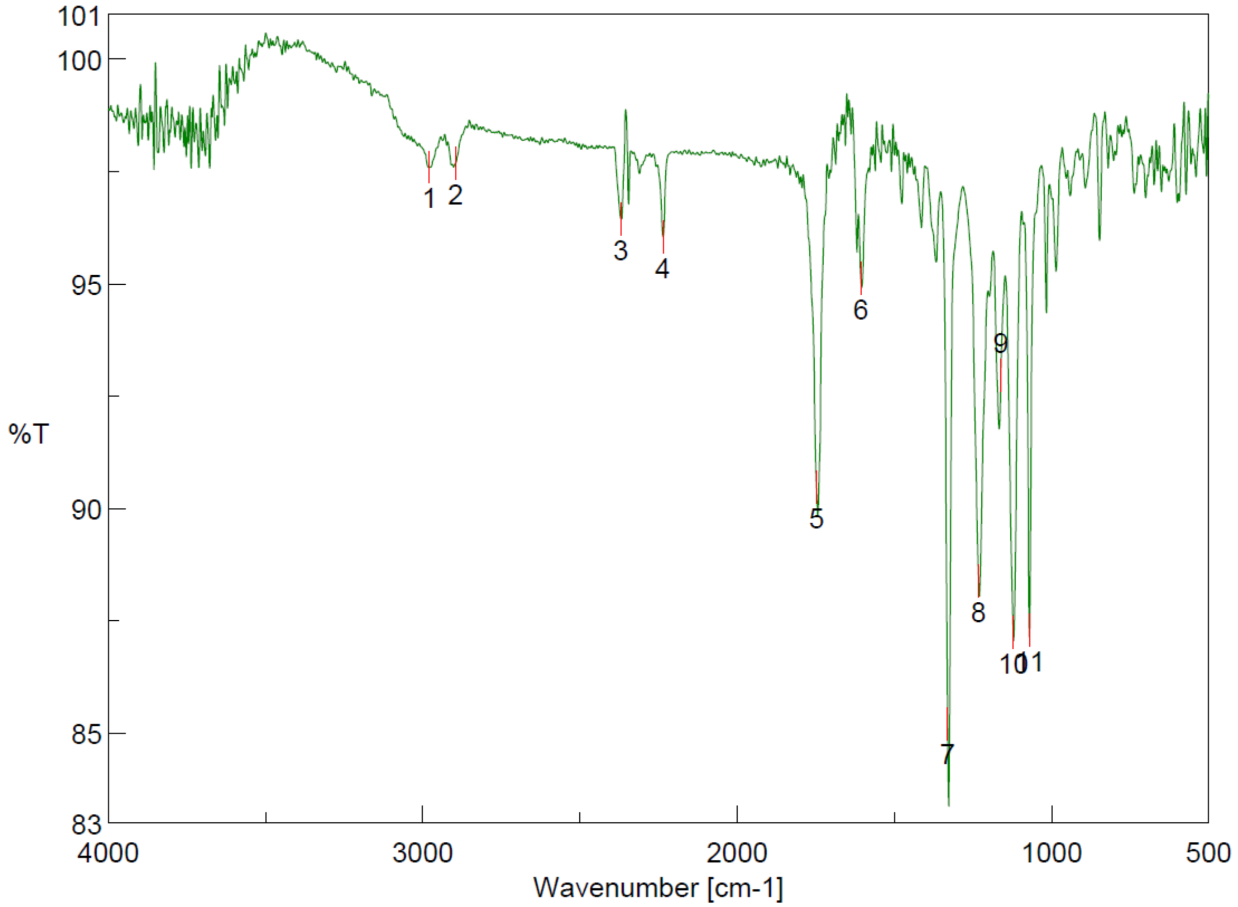

[ピーク検出結果]

No.	位置	強度	No.	位置	強度
1	2978.52	97.5901	2	2896.56	97.6715
3	2370.09	96.4447	4	2236.06	96.0393
5	1748.16	90.4657	6	1606.41	95.1147
7	1330.64	85.212	8	1232.29	88.3748
9	1160.94	92.964	10	1122.37	87.2417
11	1069.33	87.2883			

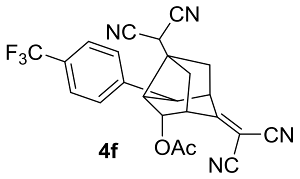

4f

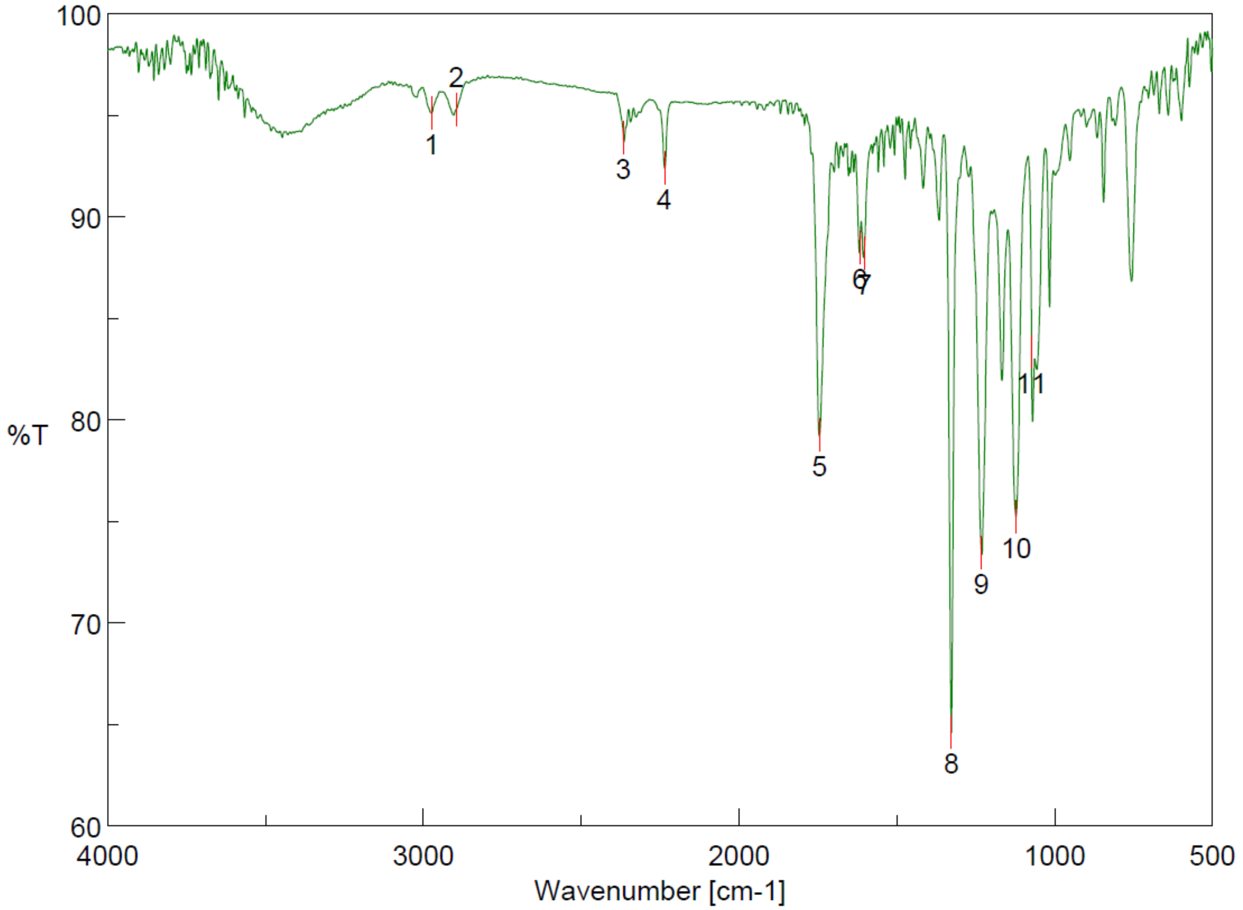

[ピーク検出結果]

No.	位置	強度	No.	位置	強度
1	2974.66	95.1022	2	2896.56	95.2703
3	2366.23	93.8709	4	2236.06	92.3878
5	1744.3	79.2177	6	1617.02	88.4687
7	1603.52	88.1776	8	1327.75	64.6282
9	1232.29	73.4606	10	1122.37	75.2206
11	1073.19	83.3416			

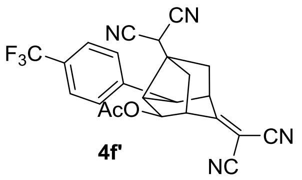

4f'

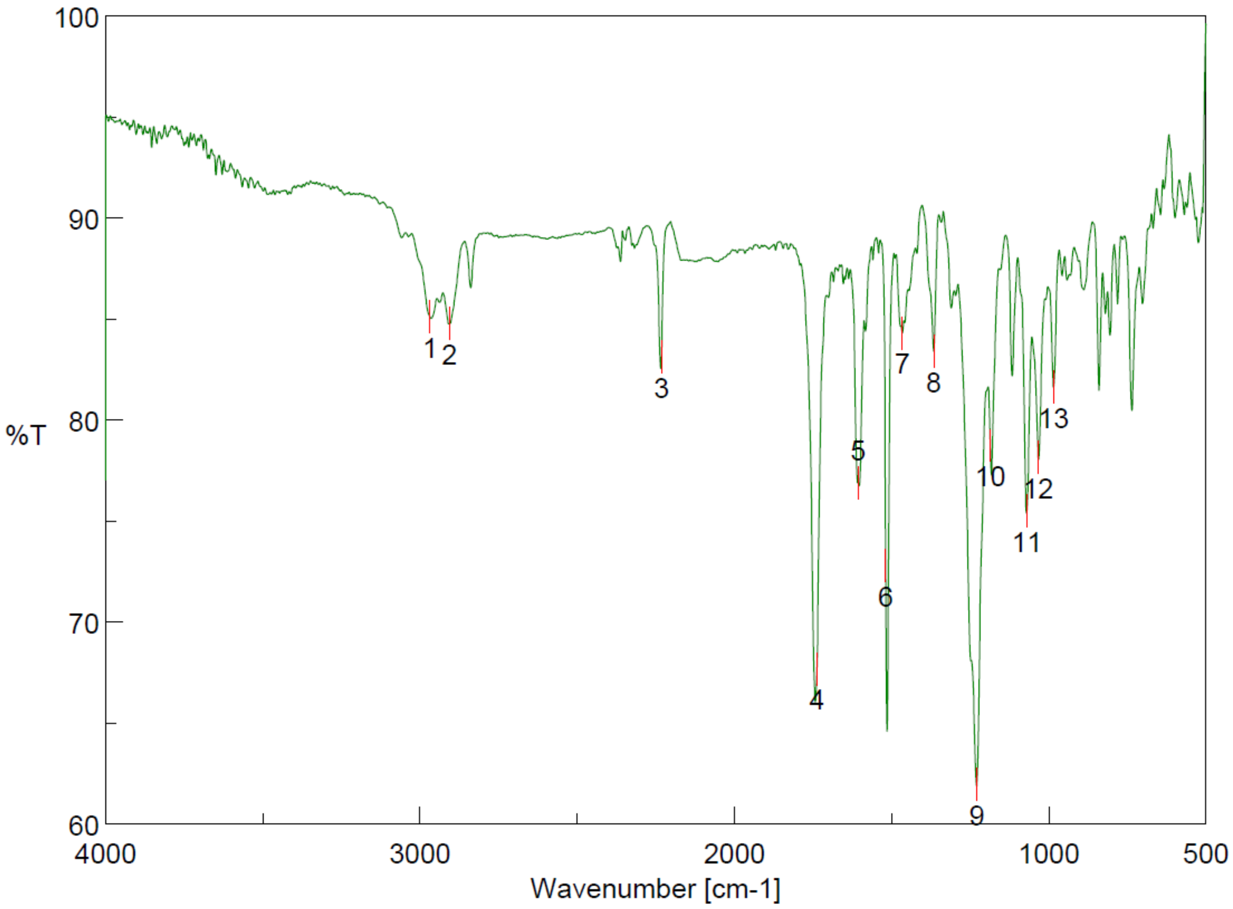

[ピーク検出結果]

No.	位置	強度	No.	位置	強度
1	2967.91	85.0793	2	2907.16	84.7495
3	2232.2	83.1226	4	1737.55	67.6677
5	1606.41	76.8808	6	1518.67	72.7844
7	1465.63	84.3015	8	1366.32	83.3938
9	1228.43	61.9713	10	1186.01	78.7309
11	1069.33	75.5142	12	1033.66	78.1589
13	984.482	81.6048			

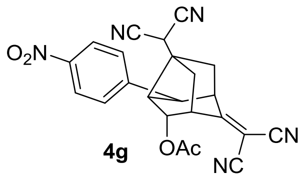

4g

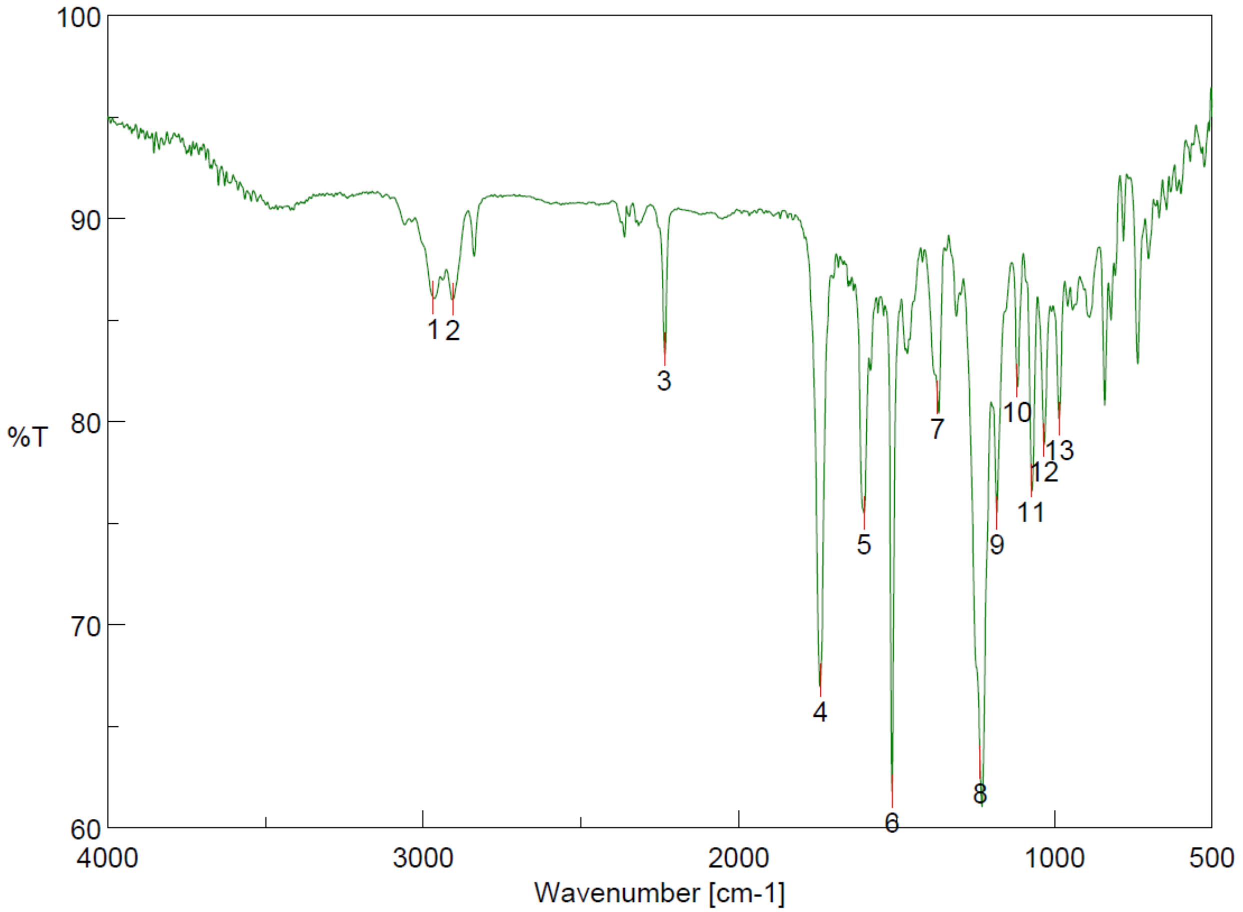

[ピーク検出結果]

No.	位置	強度	No.	位置	強度
1	2967.91	86.0946	2	2907.16	86.0163
3	2236.06	83.5616	4	1740.44	67.2681
5	1603.52	75.4821	6	1514.81	61.7971
7	1370.18	81.1803	8	1235.18	63.2446
9	1182.15	75.5106	10	1118.51	82.0042
11	1073.19	77.0899	12	1033.66	79.0745
13	984.482	80.1177			

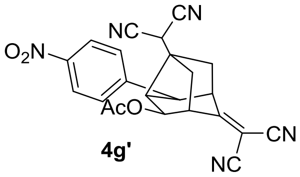

4g'

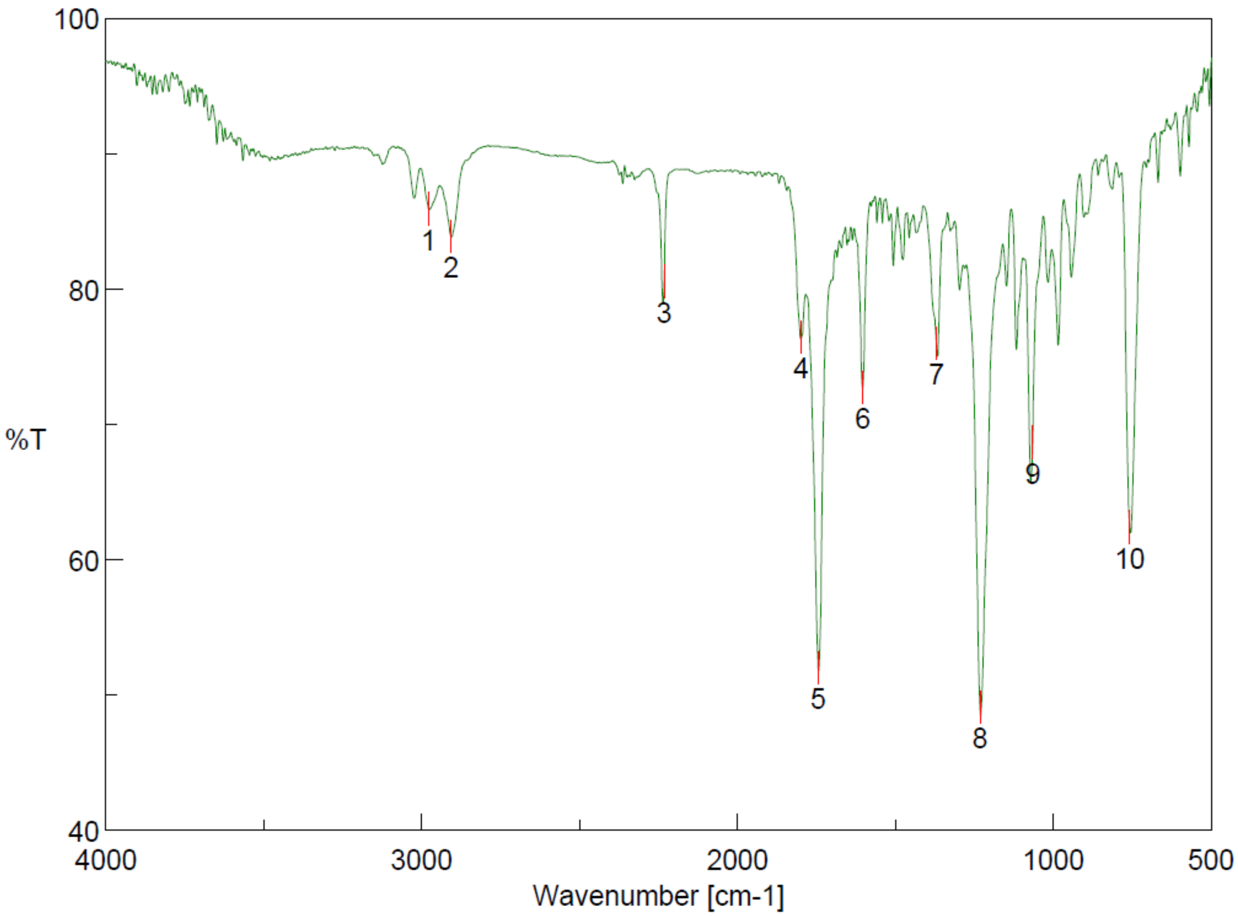

[ピーク検出結果]

No.	位置	強度	No.	位置	強度
1	2978.52	85.9324	2	2907.16	83.8936
3	2232.2	80.5814	4	1797.33	76.4347
5	1744.3	52.0446	6	1603.52	72.7014
7	1370.18	76.0051	8	1232.29	49.0768
9	1065.48	68.6974	10	757.888	62.4203

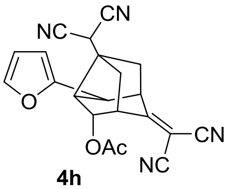

4h

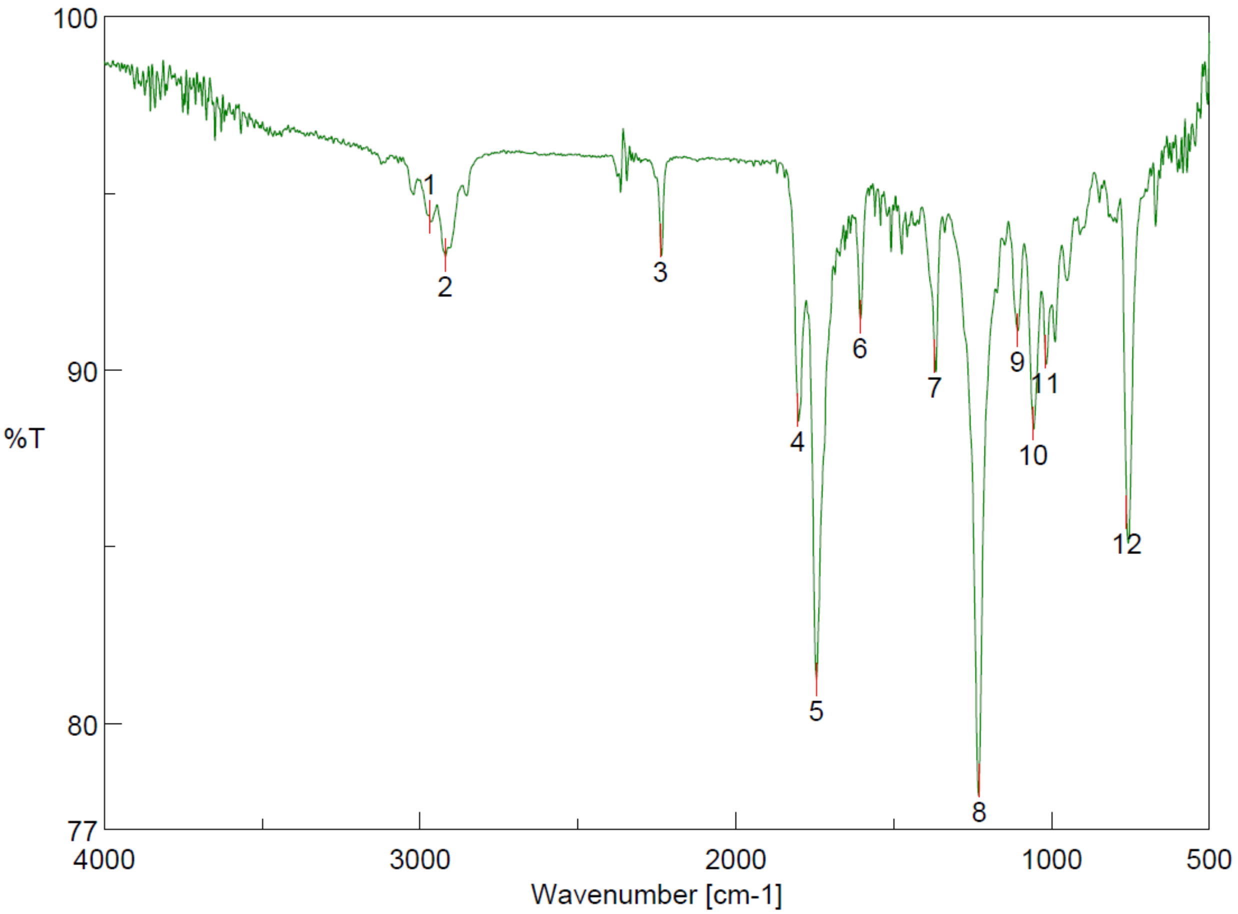

[ピーク検出結果]

No.	位置	強度	No.	位置	強度
1	2970.8	94.3217	2	2921.63	93.2474
3	2238.95	93.6628	4	1804.08	88.872
5	1744.3	81.2322	6	1606.41	91.4975
7	1370.18	90.4023	8	1228.43	78.3815
9	1107.9	91.1266	10	1058.73	88.4863
11	1020.16	90.5036	12	761.744	85.9565

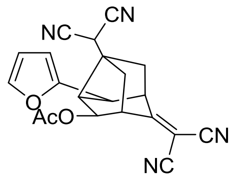

4h'

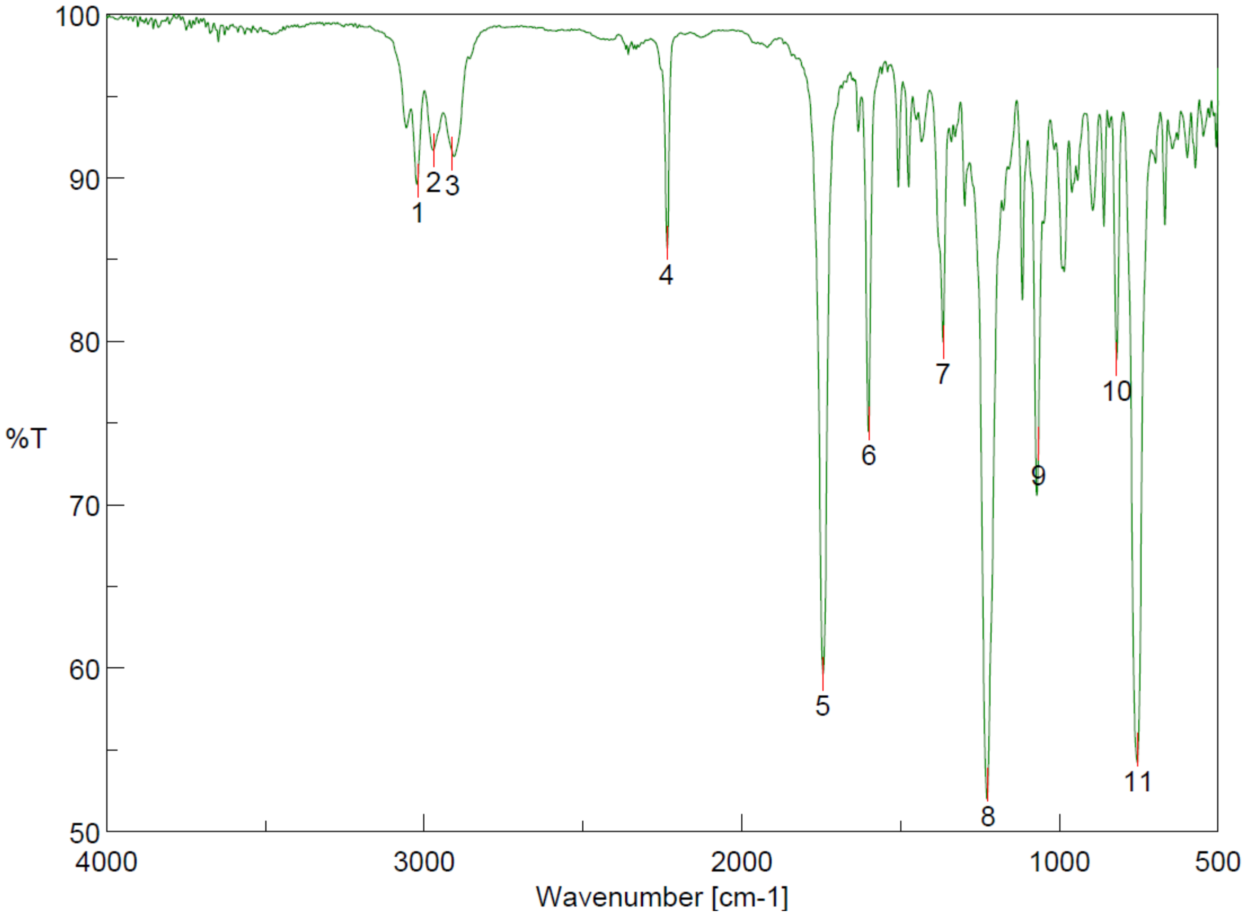

[ピーク検出結果]

No.	位置	強度	No.	位置	強度
1	3020.94	89.8012	2	2970.8	91.6822
3	2911.02	91.4896	4	2236.06	85.9981
5	1744.3	59.6575	6	1599.66	74.9435
7	1366.32	79.9324	8	1224.58	52.8819
9	1065.48	73.7166	10	818.634	78.9217
11	751.138	54.9647			

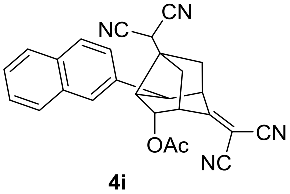

4i

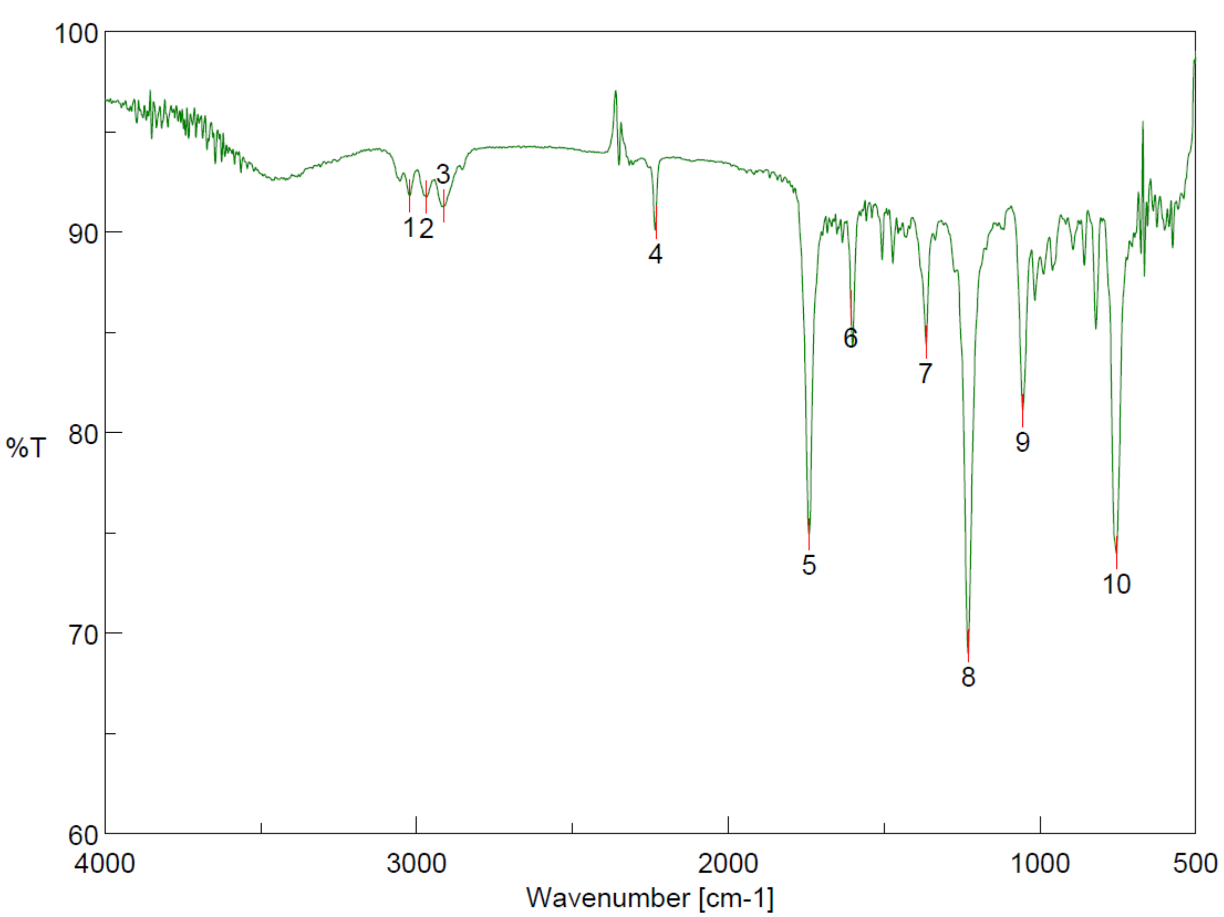

[ピーク検出結果]

No.	位置	強度	No.	位置	強度
1	3023.84	91.8061	2	2967.91	91.7298
3	2914.88	91.2835	4	2232.2	90.4691
5	1740.44	74.9093	6	1606.41	86.2504
7	1366.32	84.5267	8	1228.43	69.3378
9	1054.87	81.0794	10	754.995	73.981

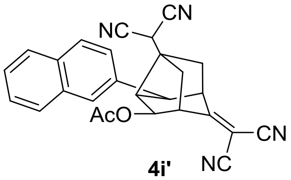

4i'

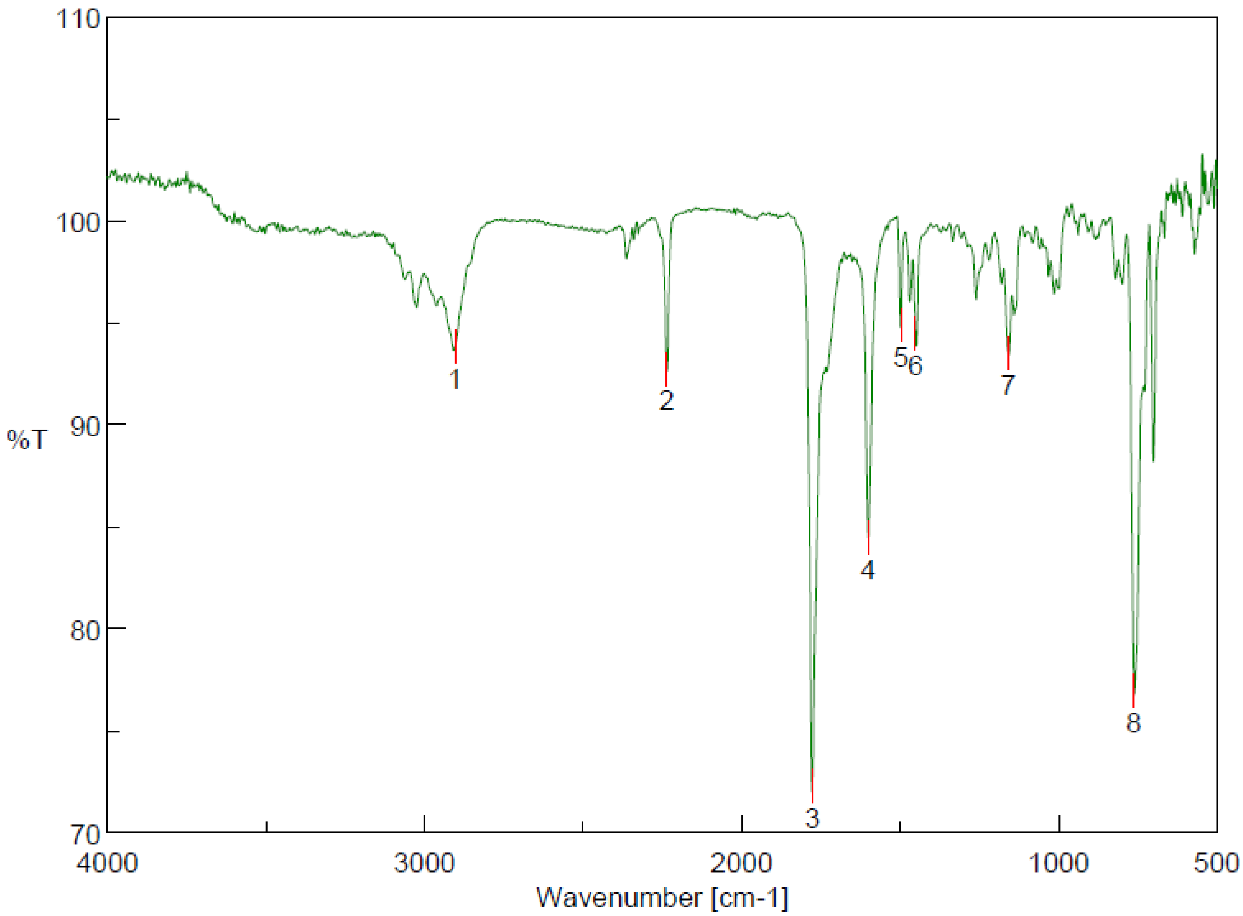

[ピーク検出結果]

No.	位置	強度	No.	位置	強度
1	2904.27	93.8154	2	2236.06	92.7204
3	1776.12	72.2742	4	1599.66	84.4245
5	1497.45	94.8718	6	1451.17	94.4281
7	1158.04	93.4885	8	761.744	76.9304

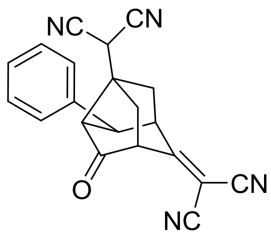

5

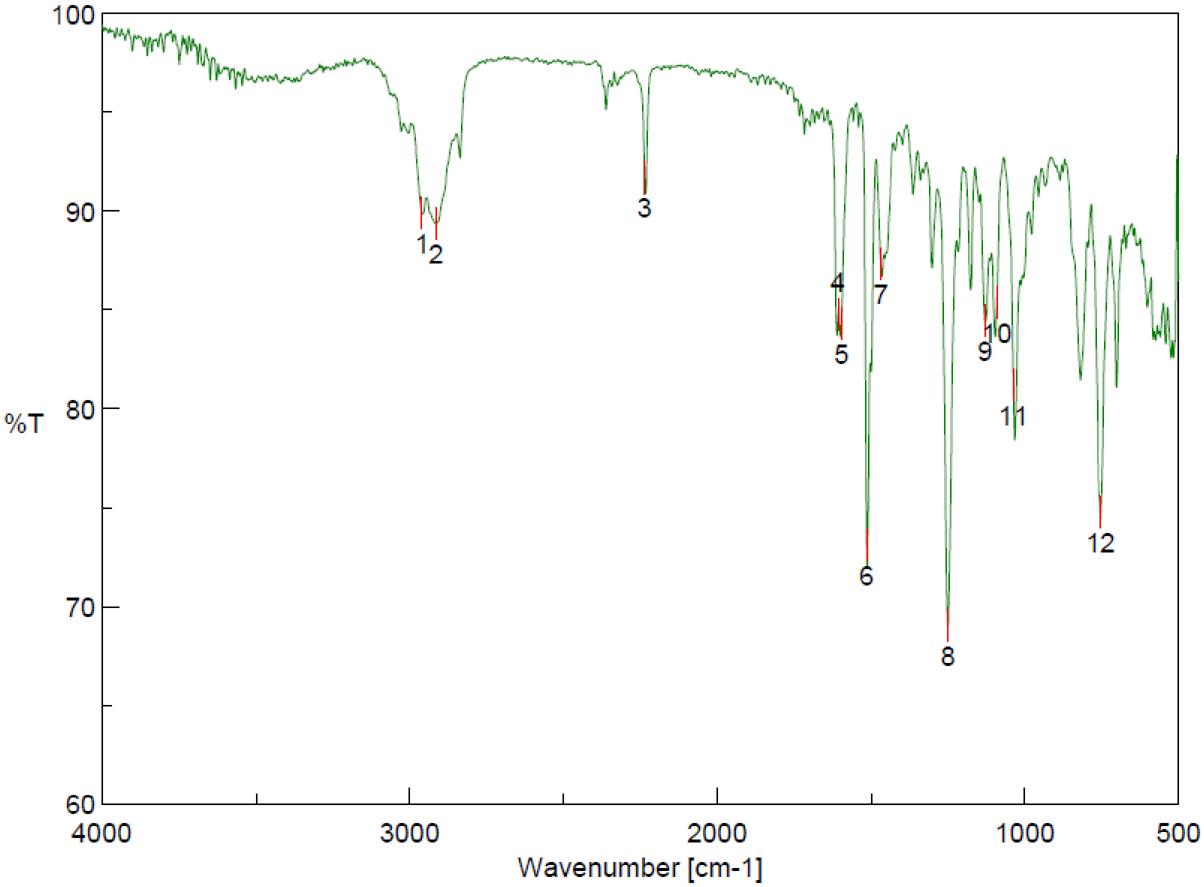

[ピーク検出結果]

No.	位置	強度	No.	位置	強度
1	2960.2	89.8738	2	2914.88	89.3431
3	2236.06	91.6785	4	1606.41	84.7765
5	1595.81	84.3083	6	1514.81	73.086
7	1468.53	87.3024	8	1249.65	68.9916
9	1129.12	84.416	10	1090.55	85.3752
11	1037.52	81.2274	12	754.995	74.7363

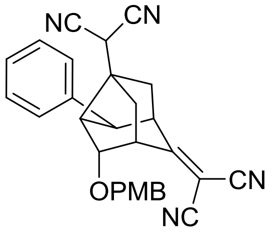

6

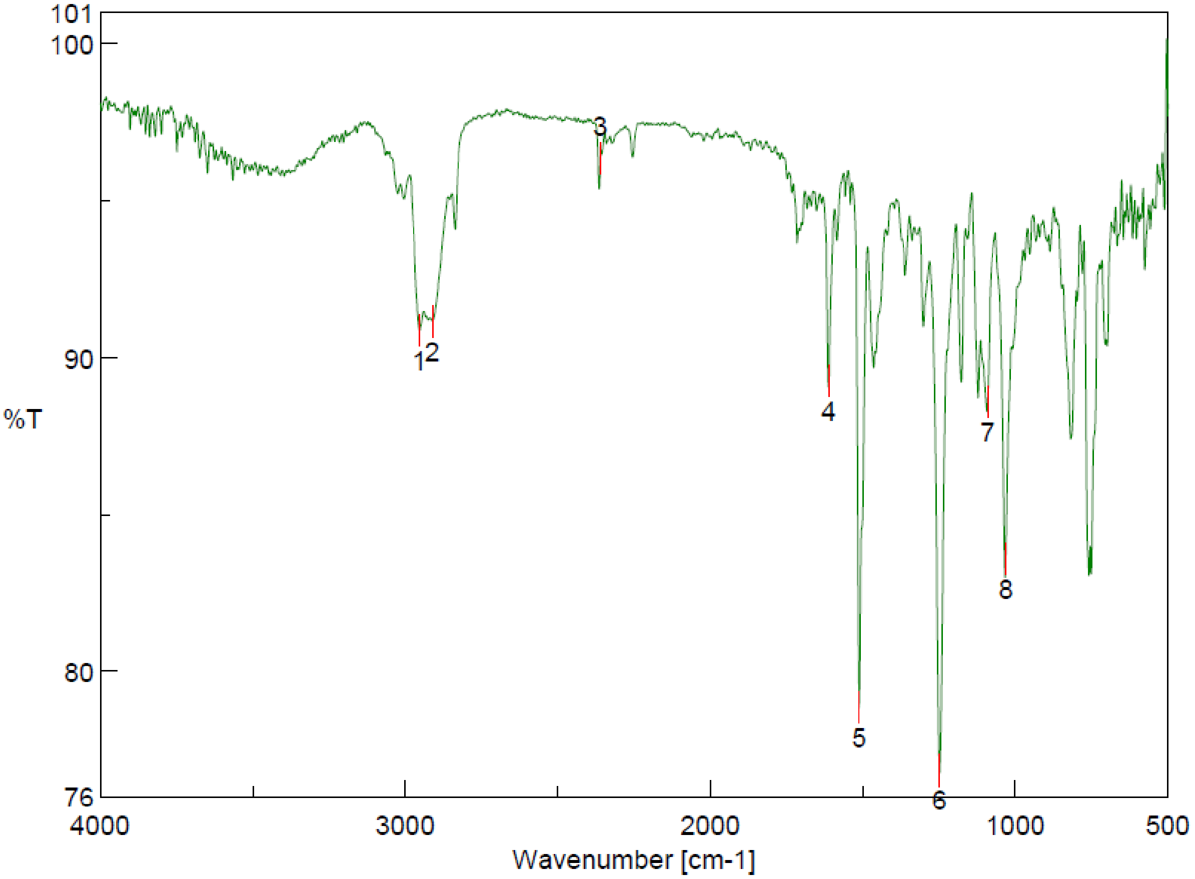

[ピーク検出結果]

No.	位置	強度	No.	位置	強度
1	2953.45	90.8673	2	2911.02	91.1576
3	2359.48	96.3344	4	1610.27	89.2623
5	1510.95	78.8628	6	1249.65	76.8347
7	1090.55	88.5644	8	1030.77	83.5619

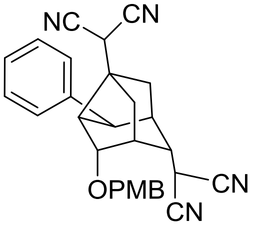

7

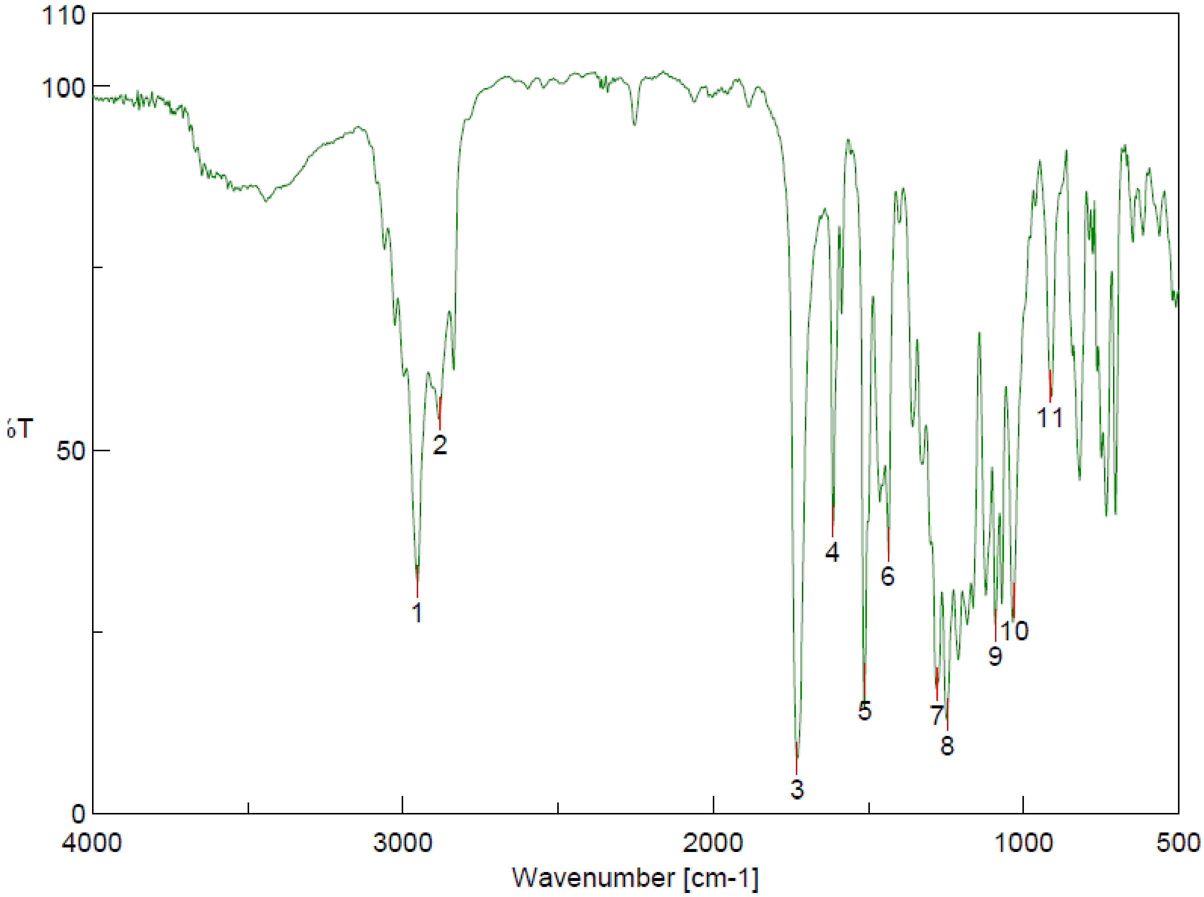

[ピーク検出結果]

No.	位置	強度	No.	位置	強度
1	2953.45	31.8761	2	2879.2	55.0091
3	1729.83	7.45139	4	1614.13	40.1524
5	1510.95	18.2558	6	1436.71	36.916
7	1277.61	17.7083	8	1245.79	13.5034
9	1090.55	25.9188	10	1030.77	29.2586
11	914.093	58.6553			

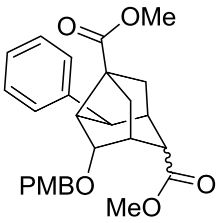

8

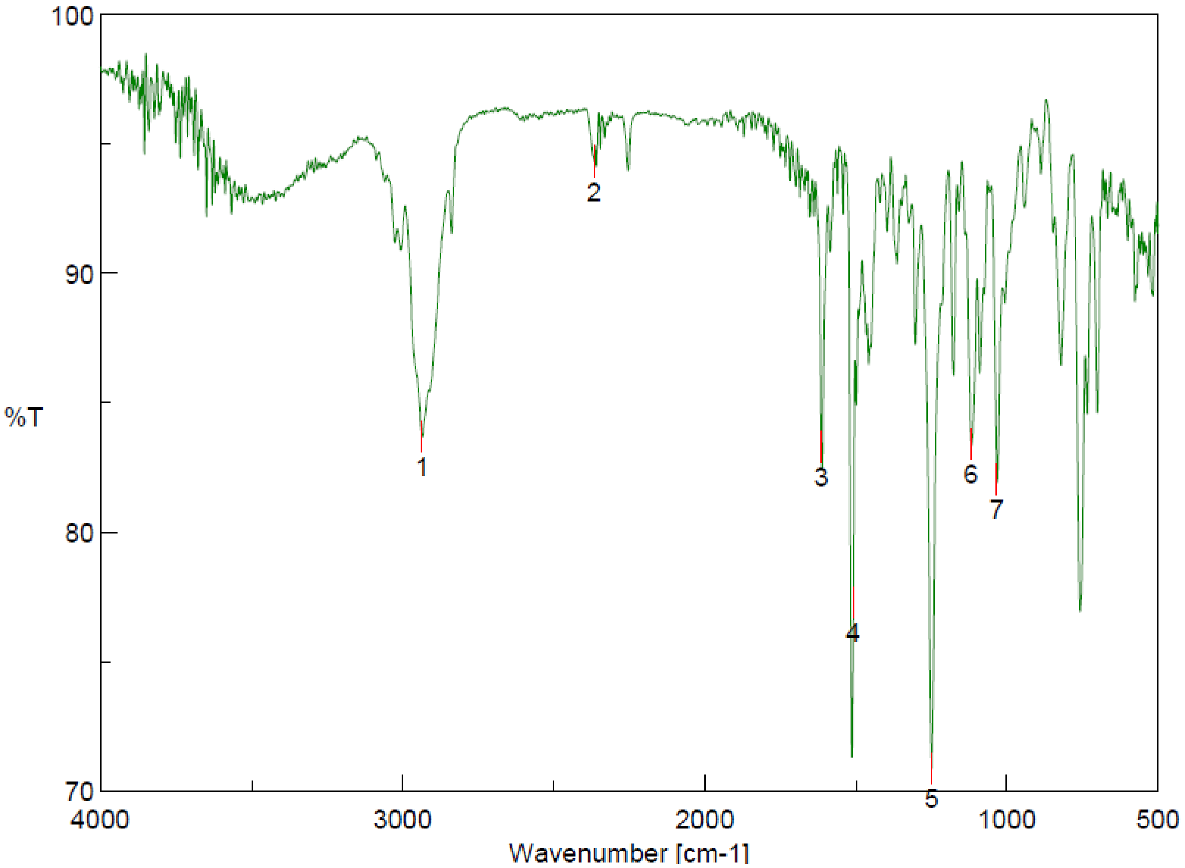

[ピーク検出結果]

No.	位置	強度	No.	位置	強度
1	2936.09	83.6734	2	2366.23	94.3223
3	1614.13	83.2958	4	1508.06	77.2538
5	1249.65	70.8758	6	1118.51	83.3938
7	1033.66	82.0423			

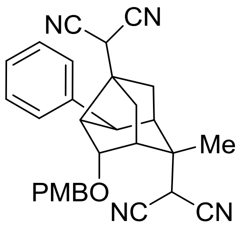

9

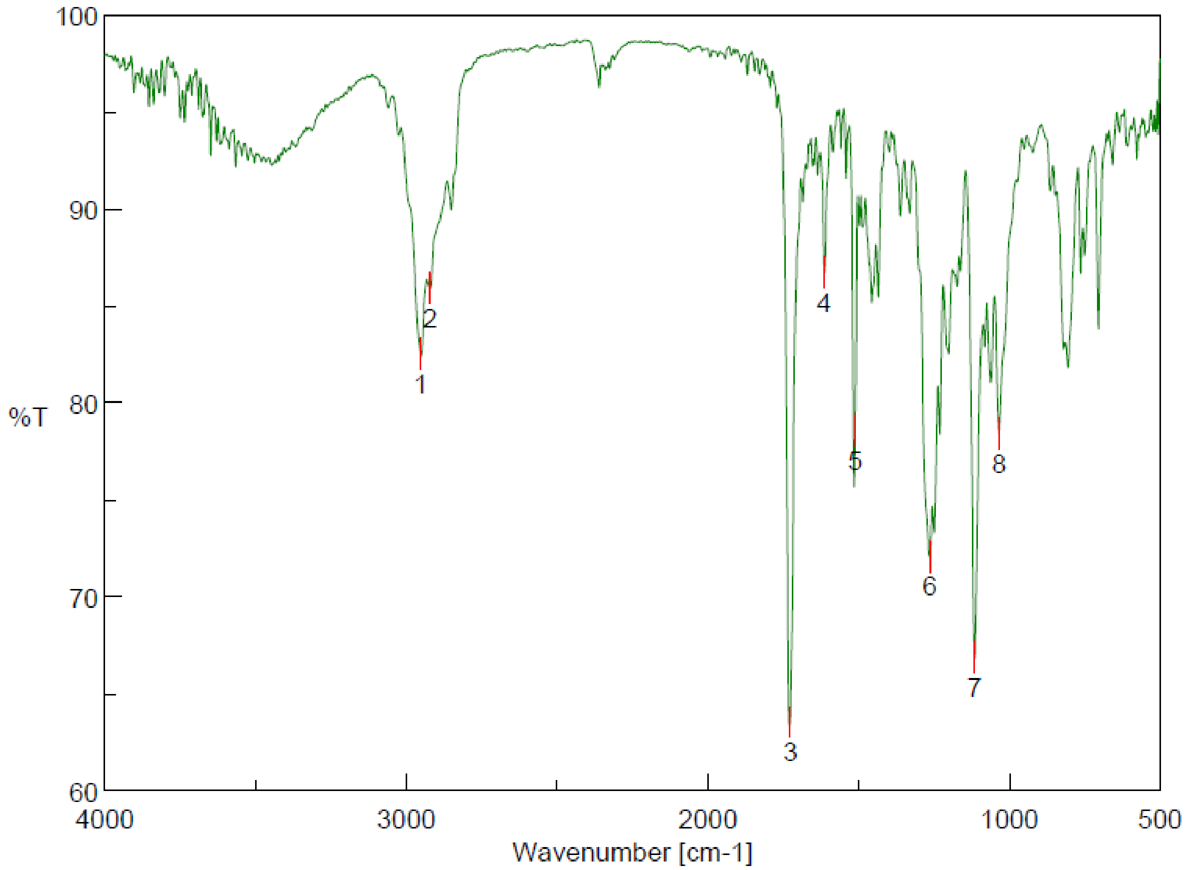

[ピーク検出結果]

No.	位置	強度	No.	位置	強度
1	2953.45	82.4985	2	2921.63	85.9192
3	1726.94	63.5336	4	1614.13	86.7125
5	1510.95	78.5586	6	1264.11	72.0645
7	1115.62	66.8955	8	1033.66	78.4144

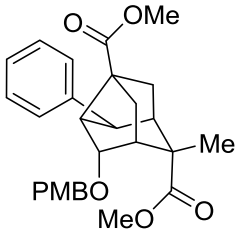

10

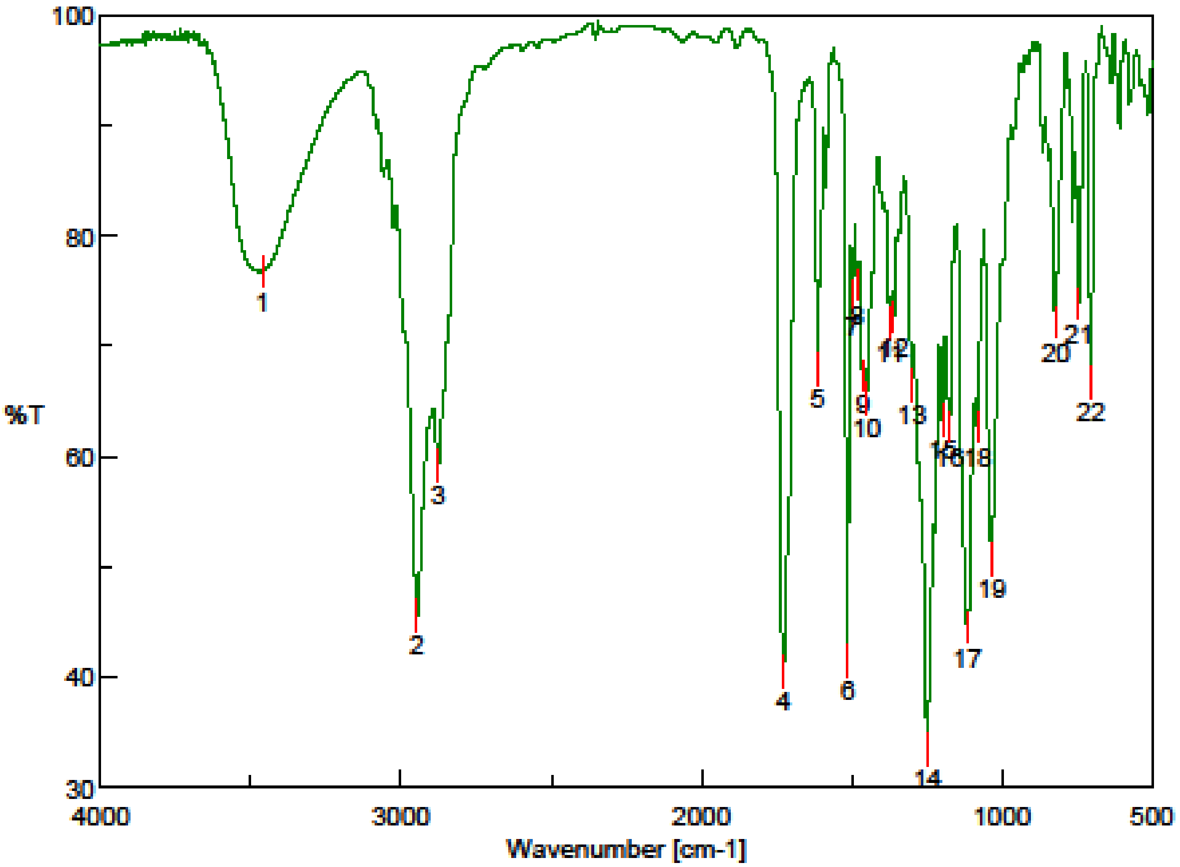

[ピーク検出結果]

No.	位置	強度	No.	位置	強度
1	3462.56	76.7154	2	2947.66	45.5461
3	2877.27	59.1691	4	1726.94	40.3964
5	1613.16	67.9303	6	1513.85	41.3954
7	1497.45	74.3953	8	1481.06	75.5136
9	1463.71	67.3248	10	1450.21	65.1809
11	1373.07	71.9571	12	1359.57	72.5668
13	1300.75	66.408	14	1247.72	33.4244
15	1197.58	63.2226	16	1174.44	62.6901
17	1115.62	44.4041	18	1080.91	62.5973
19	1034.62	50.6061	20	820.563	72.1039
21	744.388	73.8415	22	703.89	66.6393

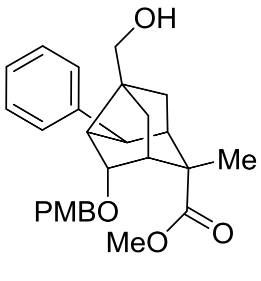

11

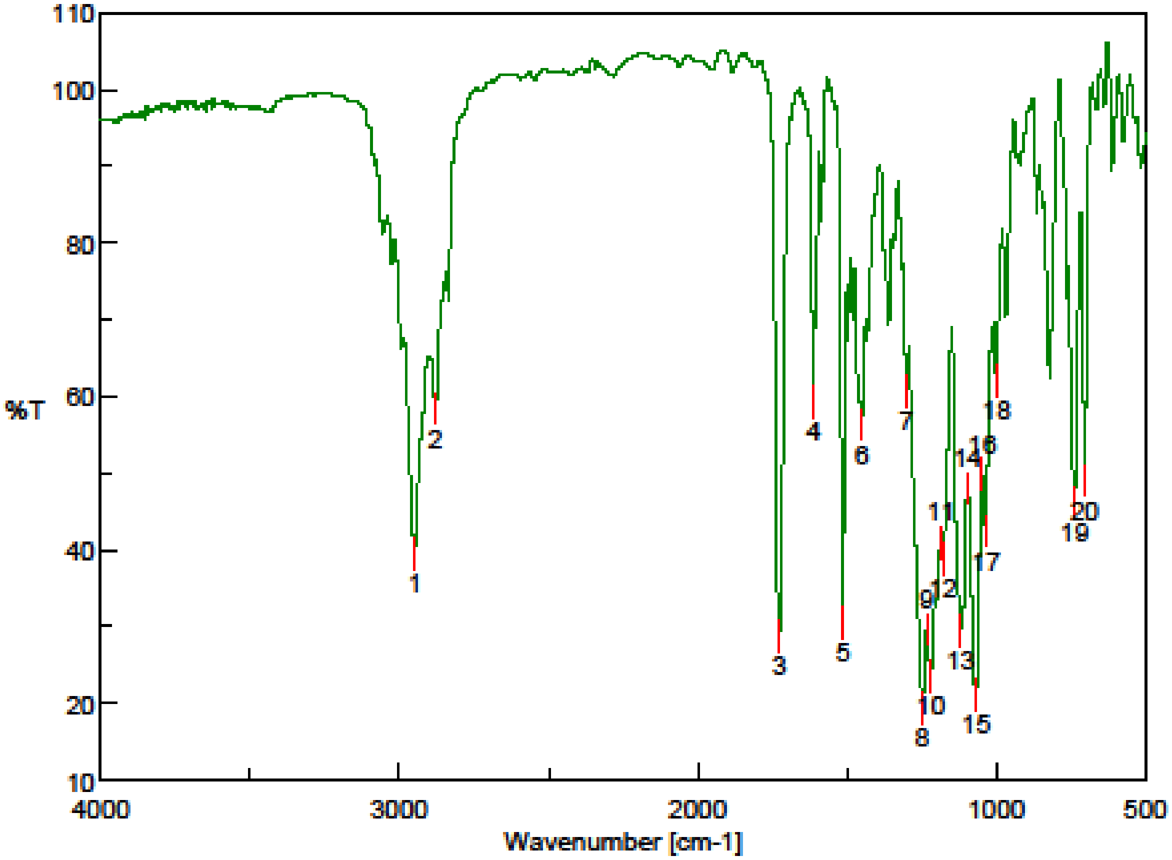

[ピーク検出結果]

No.	位置	強度	No.	位置	強度
1	2949.59	39.5054	2	2879.2	58.3879
3	1726.94	28.7491	4	1612.2	59.2527
5	1513.85	30.535	6	1450.21	56.2574
7	1301.72	60.6916	8	1246.75	19.4626
9	1233.25	29.602	10	1218.79	23.6004
11	1186.01	40.87	12	1179.26	38.7894
13	1119.48	29.4378	14	1096.33	48.0329
15	1068.37	21.0177	16	1048.12	49.8833
17	1035.59	42.4765	18	999.91	61.9822
19	737.639	46.2502	20	703.89	48.9979

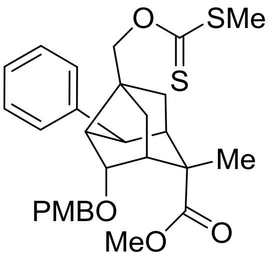

12

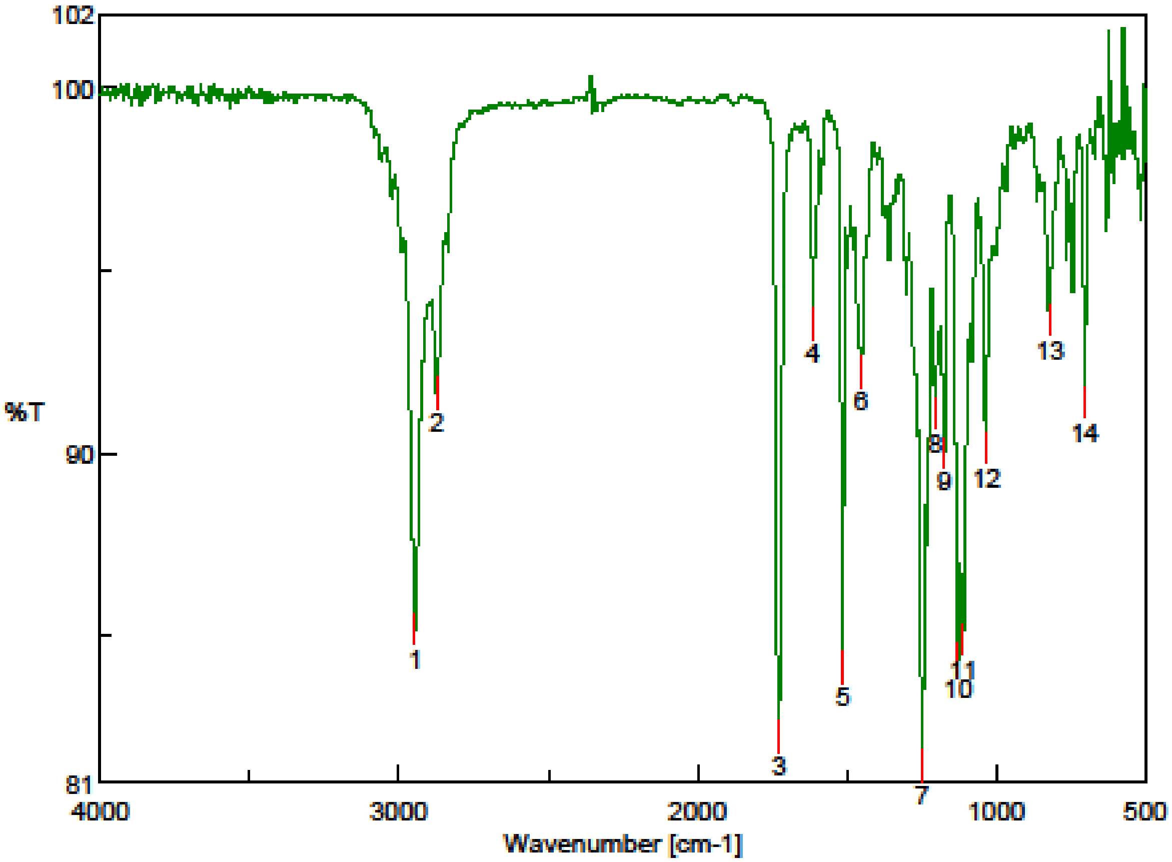

[ピーク検出結果]

No.	位置	強度	No.	位置	強度
1	2946.7	85.1814	2	2874.38	91.6239
3	1727.91	82.2207	4	1613.16	93.5291
5	1513.85	84.1458	6	1452.14	92.2197
7	1247.72	81.4387	8	1203.36	91.0816
9	1172.51	90.0097	10	1127.19	84.3356
11	1110.8	84.91	12	1034.62	90.1411
13	820.563	93.6409	14	702.926	91.3667

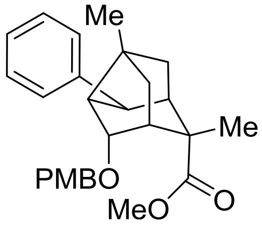

13

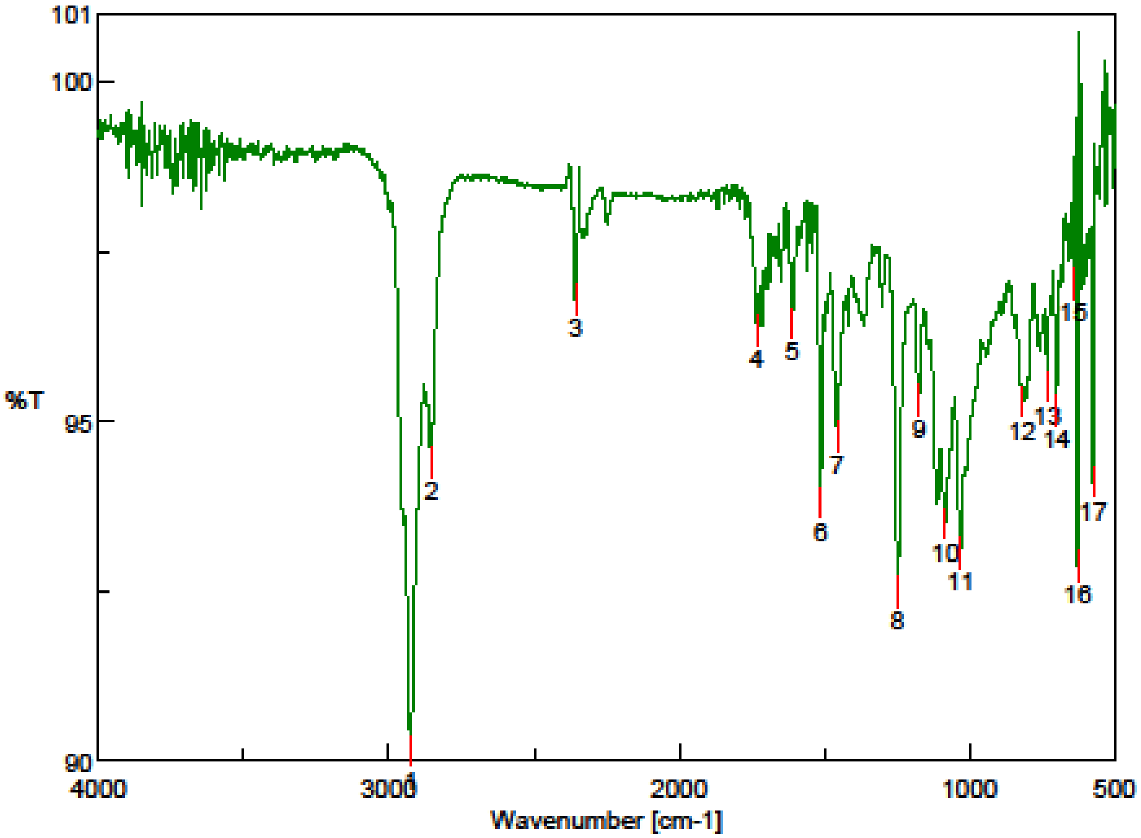

[ピーク検出結果]

No.	位置	強度	No.	位置	強度
1	2924.52	90.1364	2	2854.13	94.3802
3	2357.55	96.7901	4	1731.76	96.3415
5	1612.2	96.4368	6	1512.88	93.7916
7	1455.99	94.7707	8	1246.75	92.4711
9	1174.44	95.2963	10	1082.83	93.499
11	1030.77	93.0479	12	820.563	95.283
13	731.853	95.5083	14	700.998	95.161
15	642.179	97.0126	16	627.716	92.8563
17	573.719	94.0973			

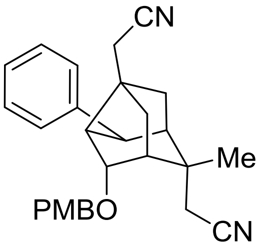

14

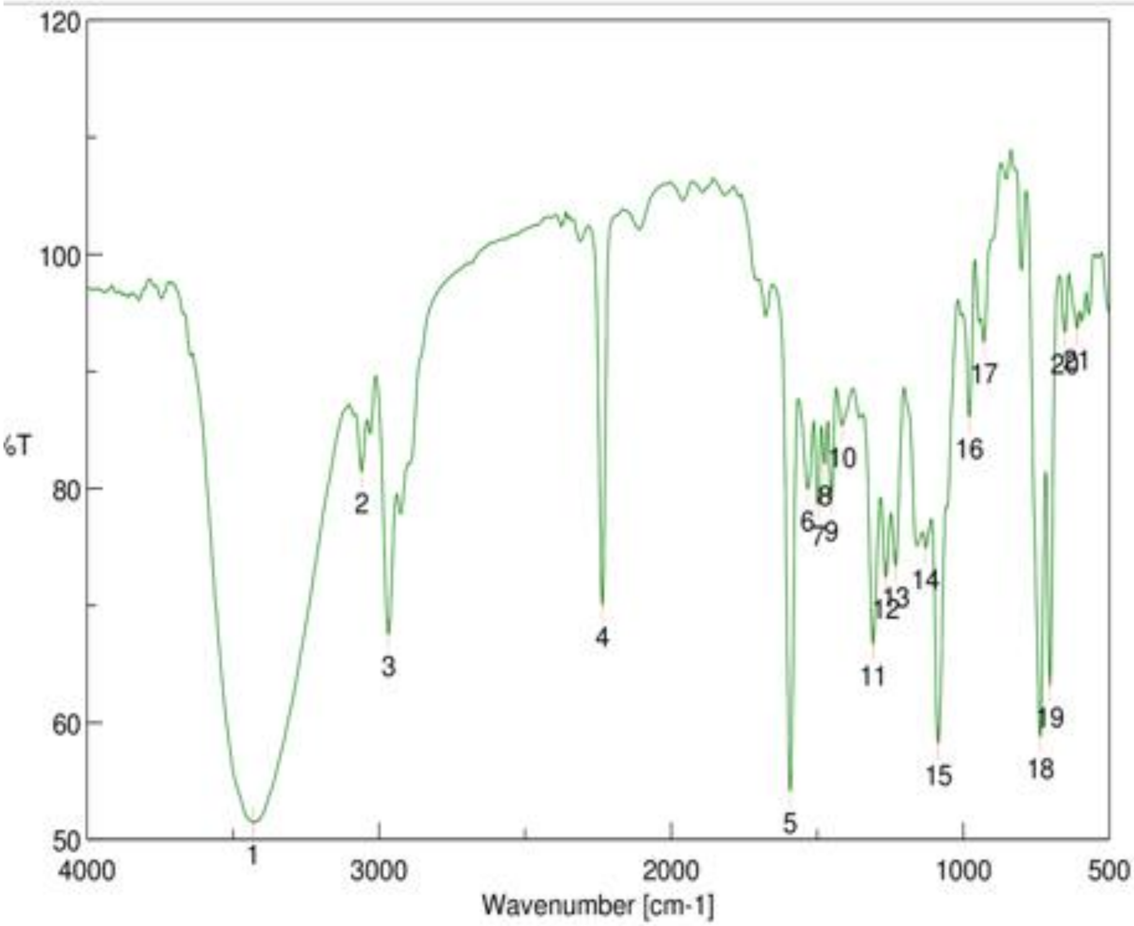

[ピーク検出結果]

No.	位置	強度	No.	位置	強度
1	3431.71	51.449	2	3060.48	81.4409
3	2967.91	67.5255	4	2236.06	70.0209
5	1592.91	54.1249	6	1533.13	79.9543
7	1497.45	78.6294	8	1474.31	82.0666
9	1451.17	79.0596	10	1414.53	85.3547
11	1308.46	66.6997	12	1265.07	72.4376
13	1231.33	73.4111	14	1129.12	74.918
15	1085.73	58.2236	16	979.661	86.0836
17	929.521	92.499	18	736.674	58.7628
19	703.89	63.1563	20	651.822	93.3352
21	610.36	93.6685			

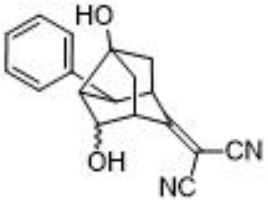

16a, 16a'

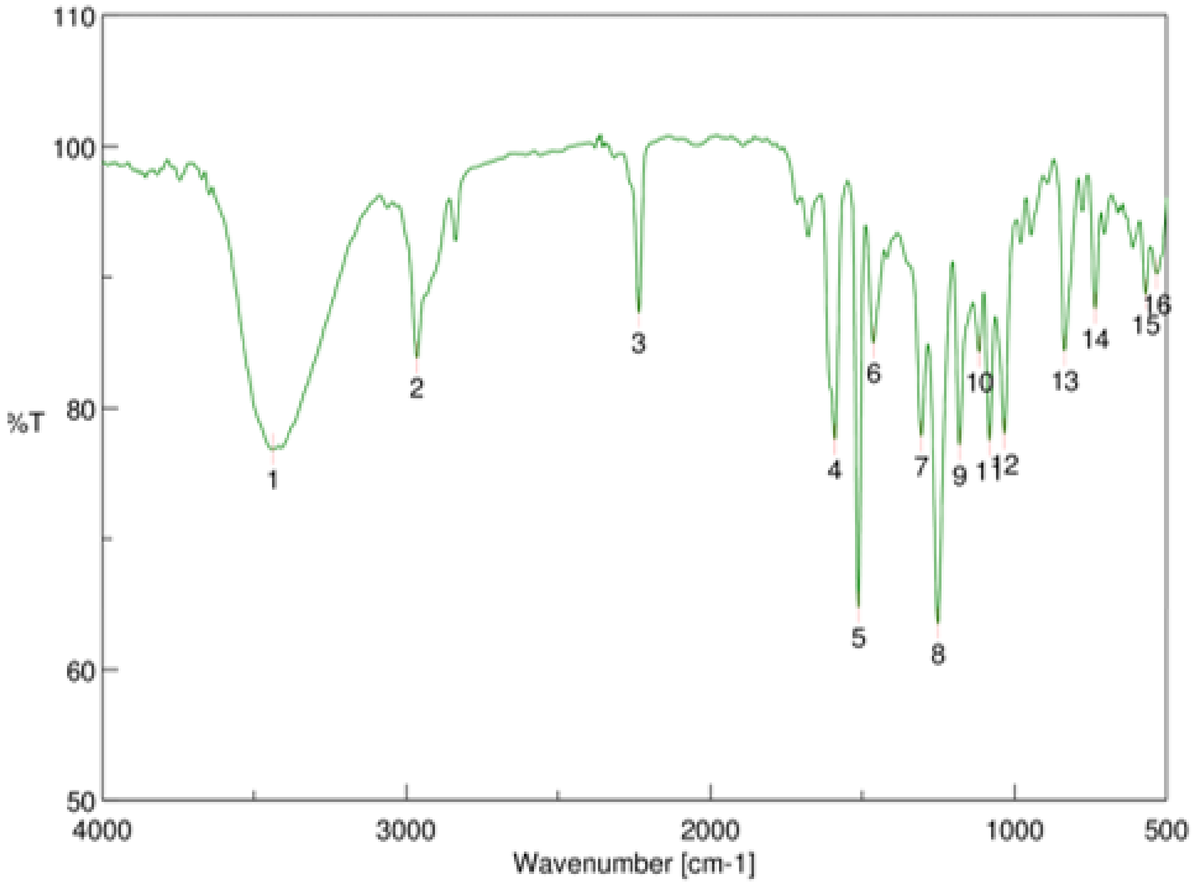

[ピーク検出結果]

No.	位置	強度	No.	位置	強度
1	3439.42	76.8516	2	2965.02	83.8205
3	2235.09	87.3086	4	1590.99	77.5839
5	1512.88	64.7466	6	1463.71	84.9839
7	1306.54	77.8438	8	1251.58	63.5398
9	1181.19	77.1539	10	1115.62	84.3045
11	1081.87	77.5205	12	1033.66	78.0052
13	835.026	84.3744	14	734.746	87.6044
15	568.898	88.6869	16	532.257	90.2389

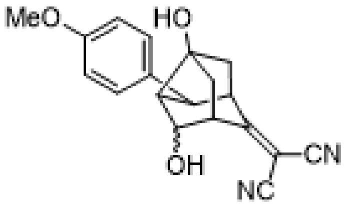

16b, 16b’

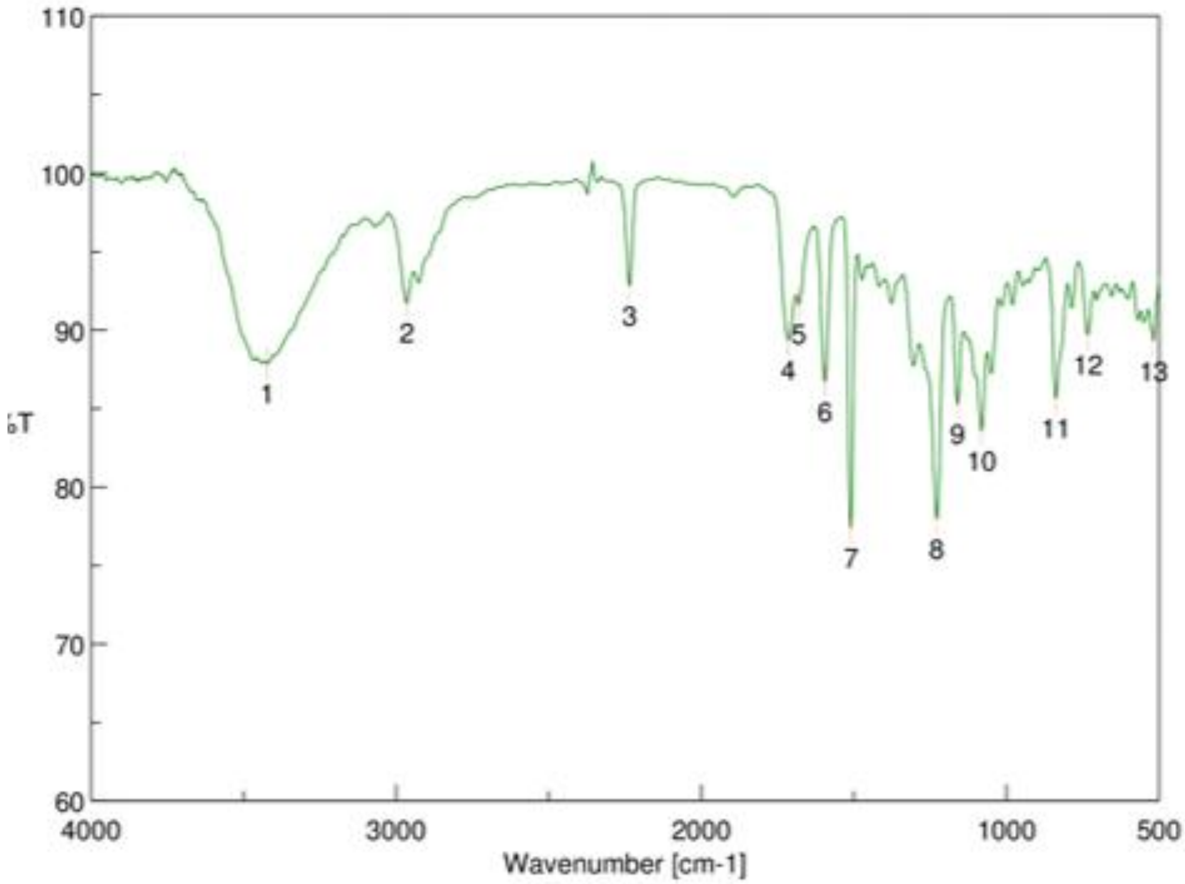

［ピーク検出結果］

No.	位置	強度	No.	位置	強度
1	3423.03	87.8514	2	2965.98	91.721
3	2235.09	92.8158	4	1714.41	89.358
5	1680.66	91.6454	6	1594.84	86.7254
7	1509.99	77.4042	8	1228.43	77.9511
9	1159.97	85.2878	10	1081.87	83.6033
11	837.919	85.6589	12	733.782	89.7186
13	517.793	89.2978			

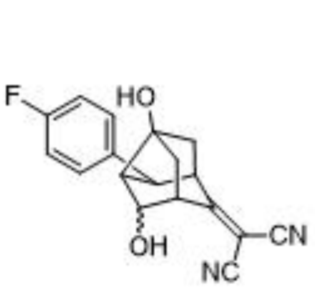

16c, 16c’

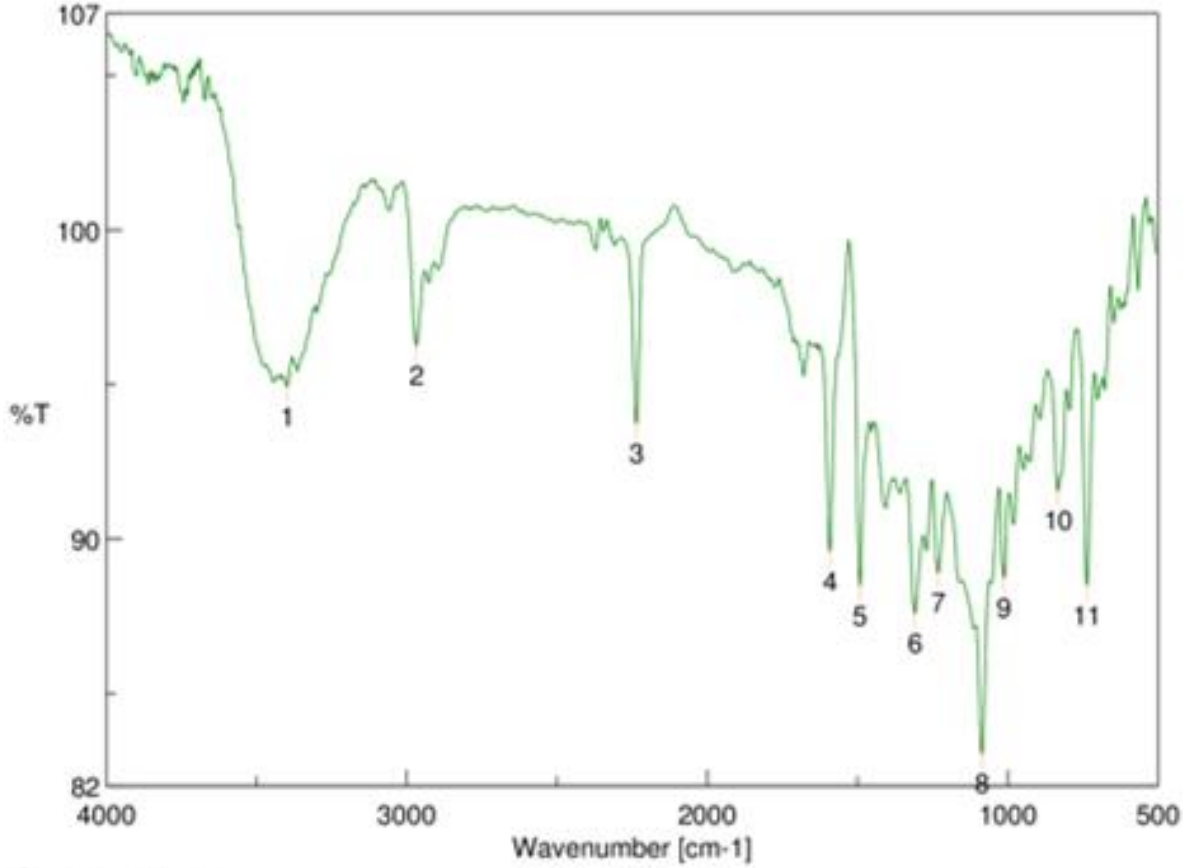

［ピーク検出結果］

No.	位置	強度	No.	位置	強度
1	3398.92	94.9194	2	2967.91	96.2506
3	2236.06	93.7287	4	1591.95	89.5951
5	1491.67	88.4705	6	1309.43	87.5832
7	1232.29	88.9068	8	1085.73	83.051
9	1012.45	88.7404	10	833.098	91.5563
11	736.674	88.4928			

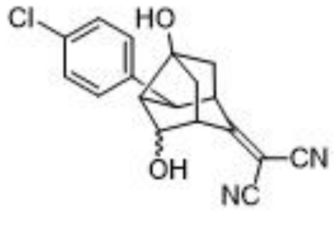

16d, 16d'

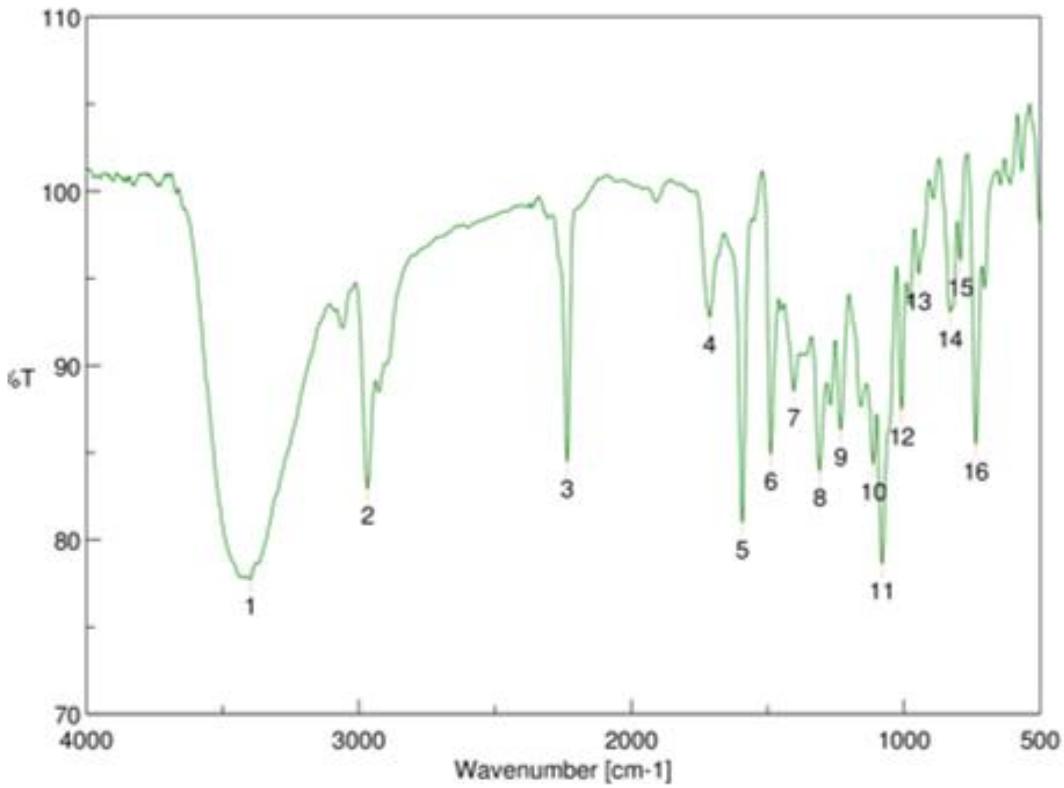

[ピーク検出結果]

No.	位置	強度	No.	位置	強度
1	3397.96	77.7417	2	2967.91	82.9282
3	2235.09	84.4867	4	1712.48	92.7776
5	1592.91	80.9664	6	1488.78	84.8953
7	1403.92	88.5262	8	1309.43	83.9693
9	1232.29	86.3087	10	1112.73	84.3235
11	1079.94	78.6061	12	1008.59	87.4634
13	945.913	95.2301	14	830.205	93.0698
15	792.6	96.0273	16	736.674	85.473

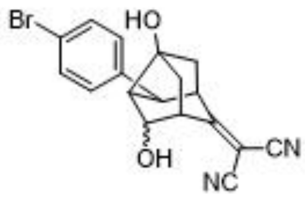

16e, 16e'

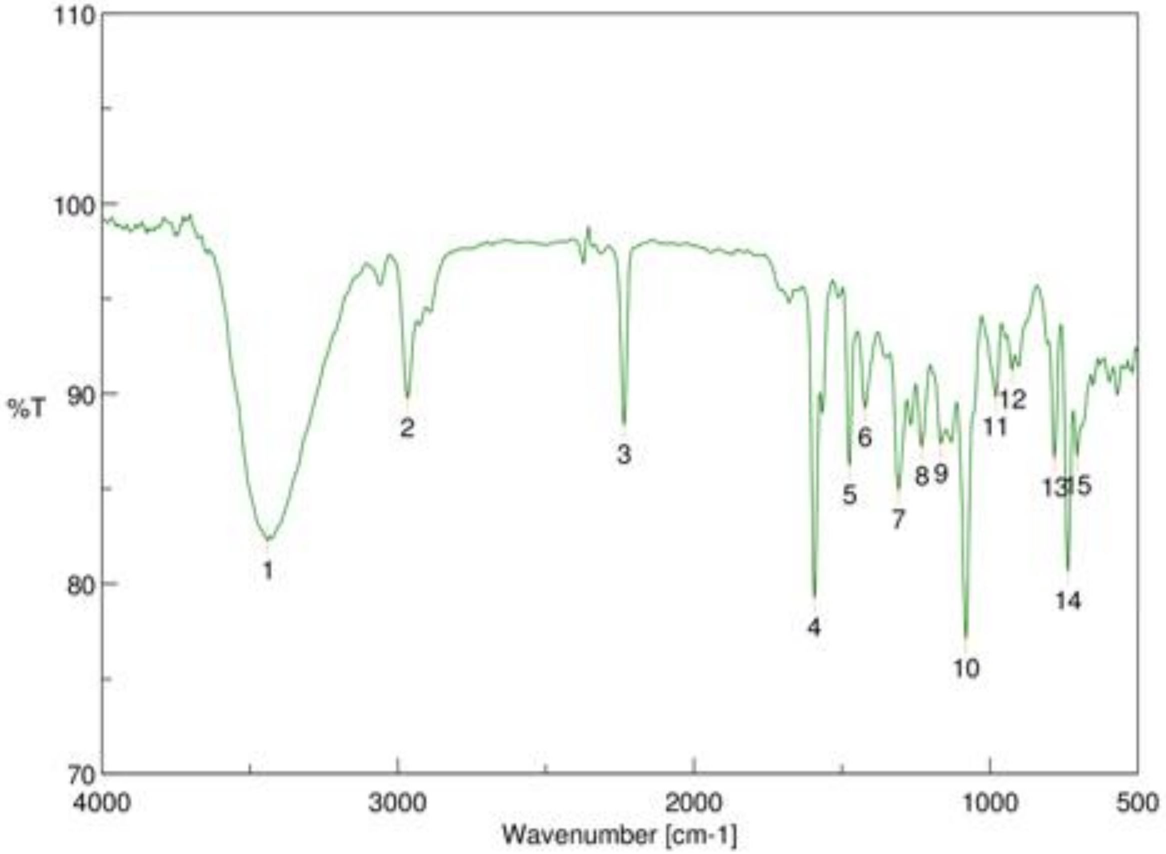

[ピーク検出結果]

No.	位置	強度	No.	位置	強度
1	3439.42	82.2776	2	2966.95	89.7741
3	2236.06	88.3719	4	1592.91	79.2687
5	1474.31	86.2536	6	1422.24	89.2563
7	1308.46	84.9506	8	1230.36	87.2238
9	1165.76	87.3851	10	1080.91	77.1324
11	980.625	89.8499	12	925.664	91.2785
13	781.993	86.6474	14	736.674	80.6878
15	704.855	86.731			

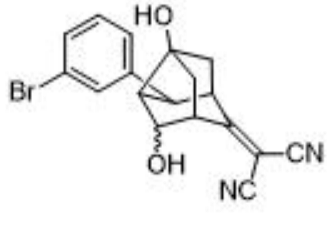

16f, 16f'

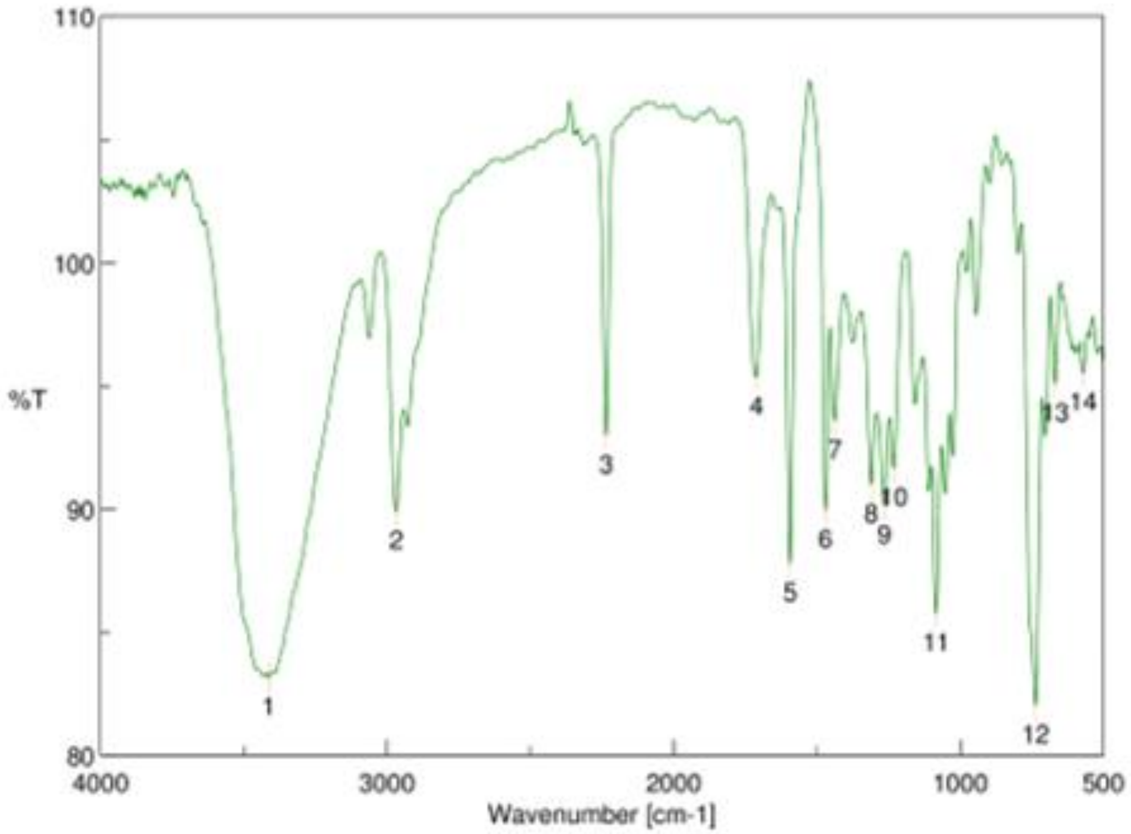

[ピーク検出結果]

No.	位置	強度	No.	位置	強度
1	3414.35	83.1641	2	2967.91	89.8978
3	2236.06	92.9889	4	1711.51	95.3557
5	1593.88	87.7797	6	1470.46	89.9671
7	1437.67	93.5814	8	1310.39	90.9889
9	1266.04	90.1277	10	1232.29	91.6659
11	1083.8	85.767	12	737.639	82.0267
13	669.178	95.0704	14	570.826	95.5145

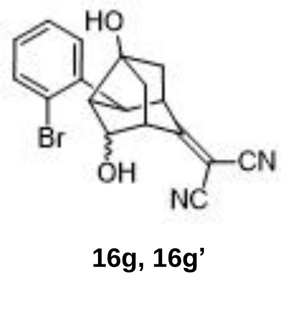

16g, 16g’

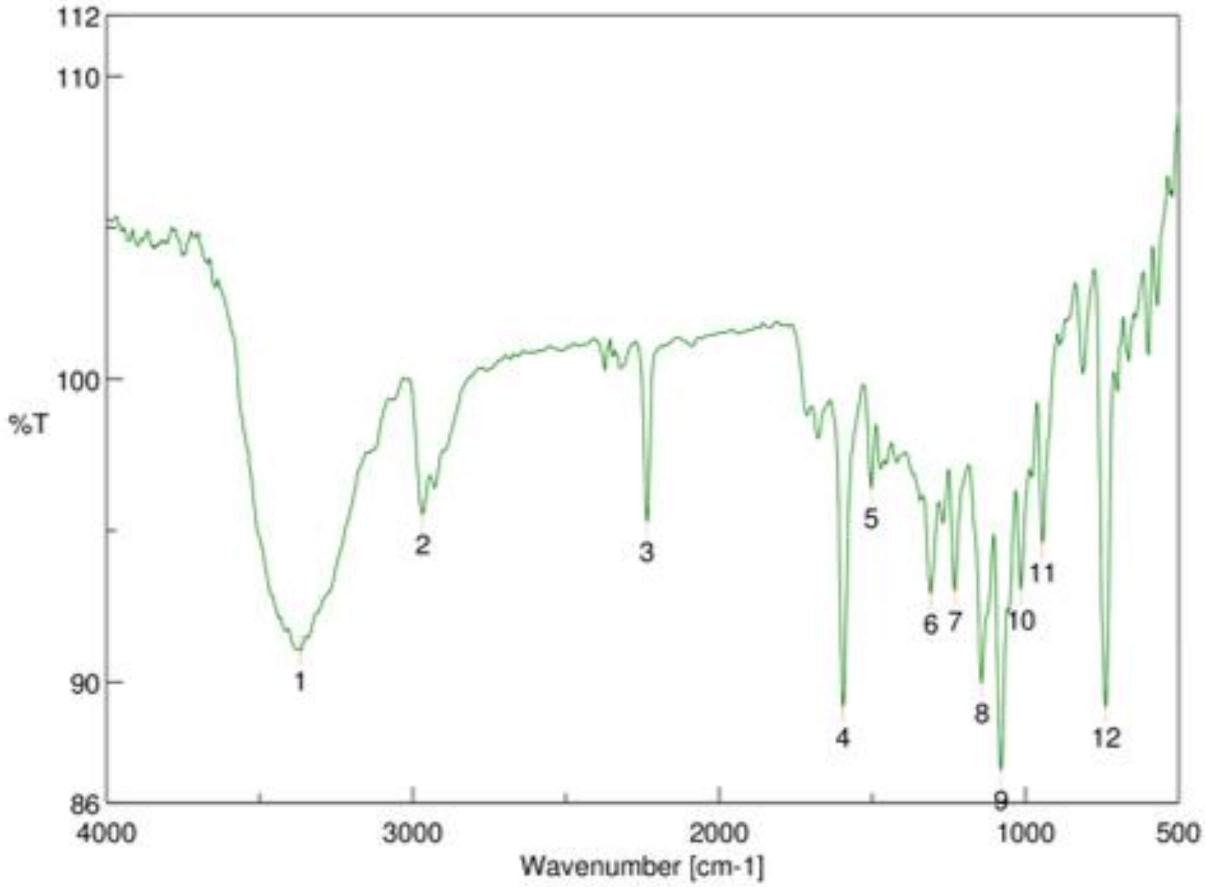

[ピーク検出結果]

No.	位置	強度	No.	位置	強度
1	3367.1	91.0407	2	2967.91	95.5439
3	2235.09	95.2943	4	1594.84	89.1837
5	1503.24	96.3926	6	1308.46	92.9294
7	1230.36	93.0023	8	1142.62	89.9735
9	1079.94	87.0908	10	1015.34	93.0423
11	943.985	94.5979	12	737.639	89.1666

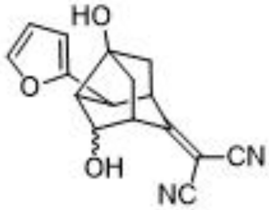

16h, 16h'

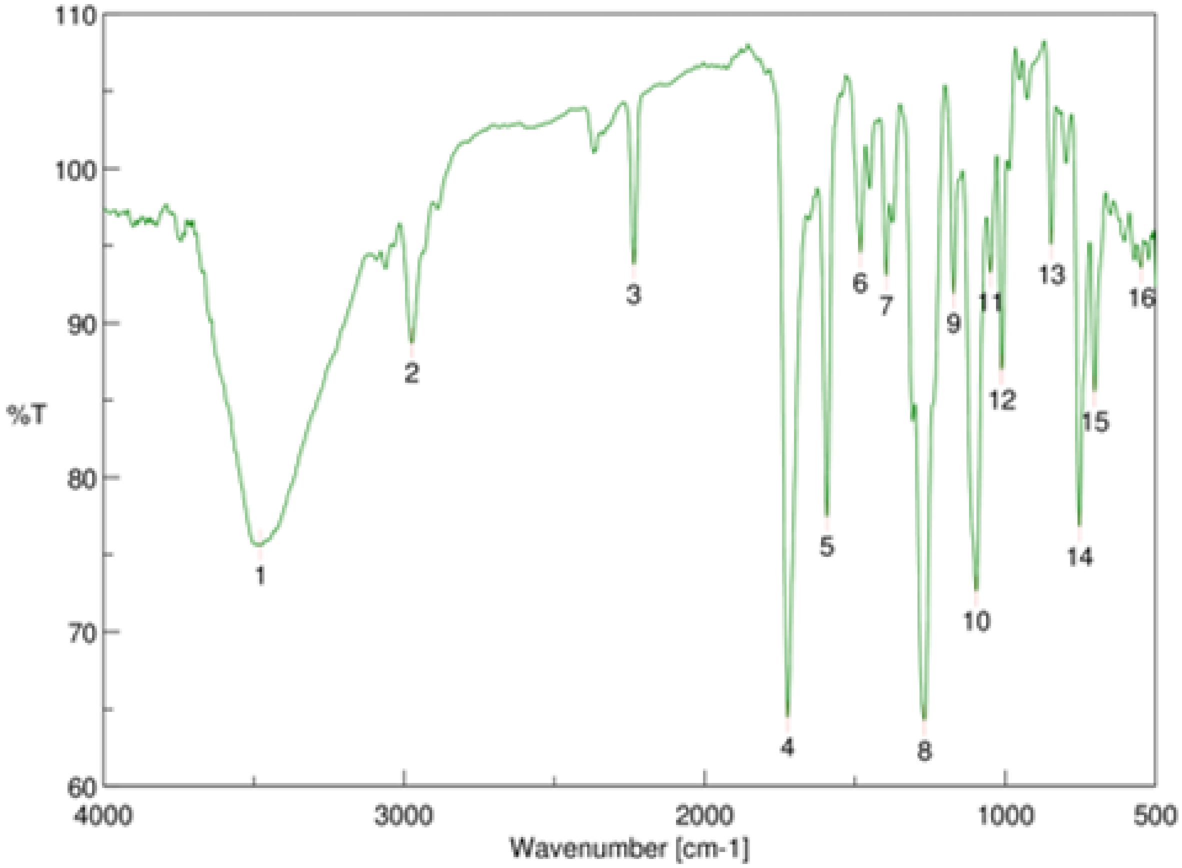

[ピーク検出結果]

No.	位置	強度	No.	位置	強度
1	3477.03	75.5978	2	2974.66	88.689
3	2235.09	93.7828	4	1724.05	64.4638
5	1592.91	77.4568	6	1481.06	94.5437
7	1396.21	93.0331	8	1268.93	64.2537
9	1172.51	91.8894	10	1097.3	72.6232
11	1049.09	93.2708	12	1012.45	86.965
13	847.561	95.0606	14	754.031	76.8189
15	702.926	85.547	16	548.649	93.5519

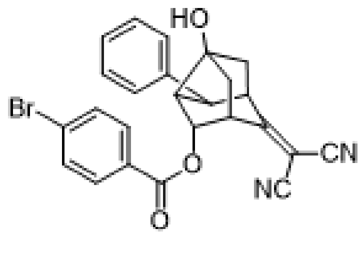

24a

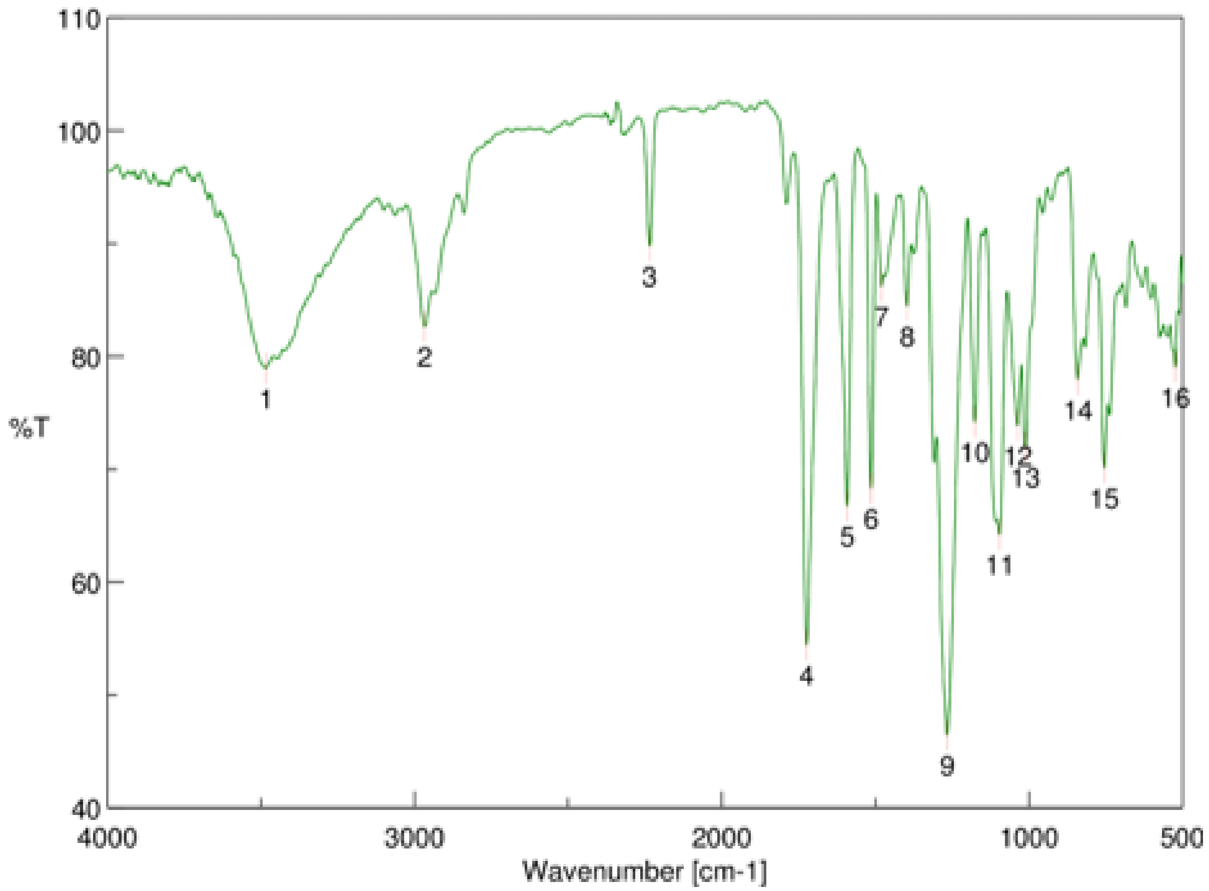

[ピーク検出結果]

No.	位置	強度	No.	位置	強度
1	3483.78	78.9186	2	2967.91	82.6444
3	2235.09	89.7422	4	1724.05	54.4195
5	1591.95	66.7378	6	1513.85	68.3034
7	1480.1	86.1915	8	1396.21	84.458
9	1266.04	46.4242	10	1176.36	74.1831
11	1095.37	64.193	12	1036.55	73.8382
13	1011.48	71.9527	14	840.812	77.9262
15	754.995	70.1077	16	523.579	79.0446

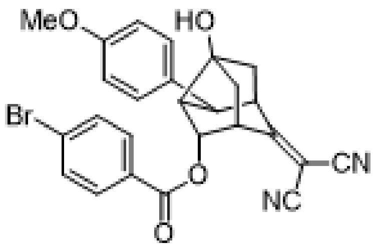

24b

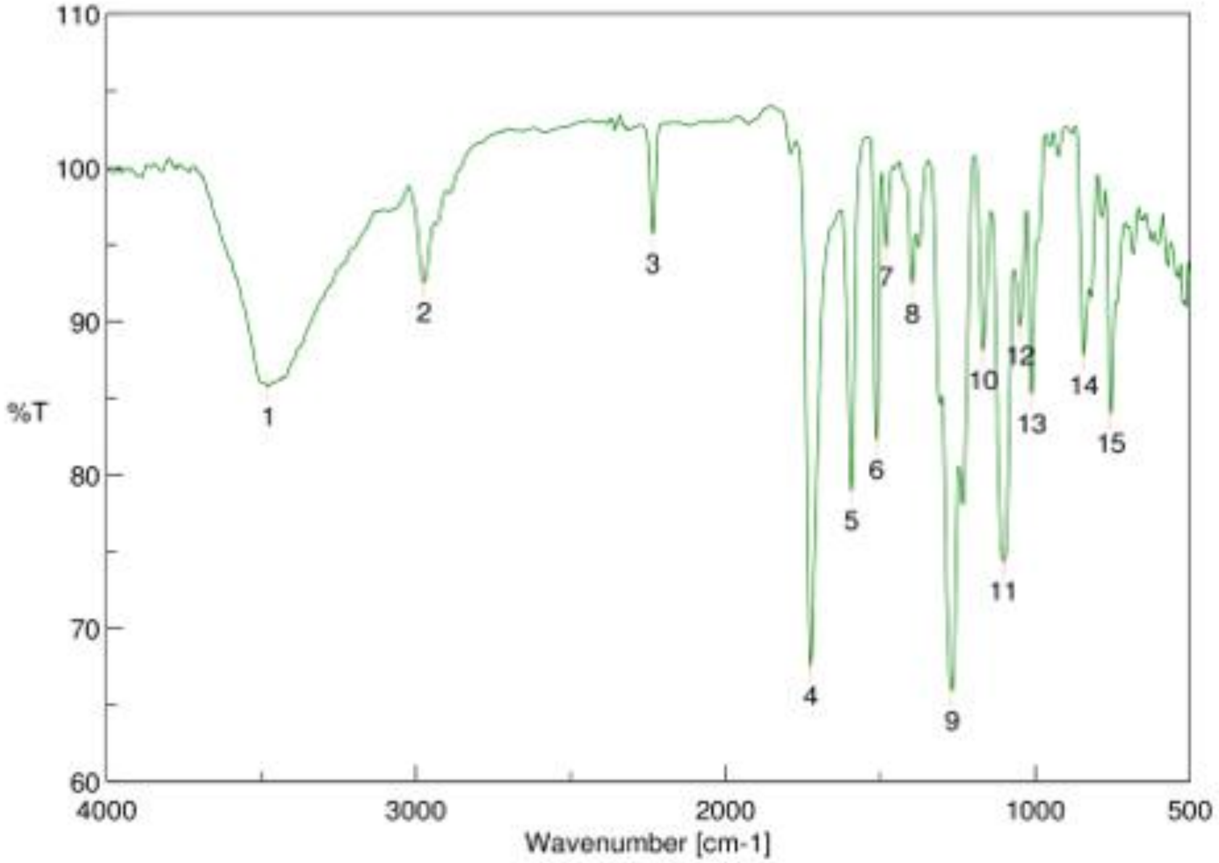

[ピーク検出結果]

No.	位置	強度	No.	位置	強度
1	3477.03	85.7204	2	2974.66	92.4769
3	2235.09	95.6637	4	1724.05	67.5163
5	1592.91	78.9265	6	1511.92	82.2688
7	1481.06	94.7773	8	1396.21	92.4199
9	1267.97	65.8815	10	1167.69	88.0753
11	1102.12	74.3297	12	1049.09	89.6811
13	1011.48	85.2367	14	842.74	87.7662
15	755.959	83.9547			

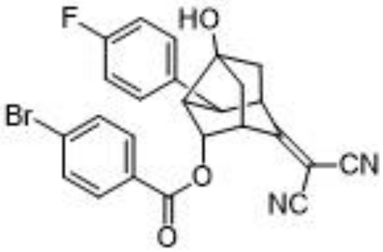

24c

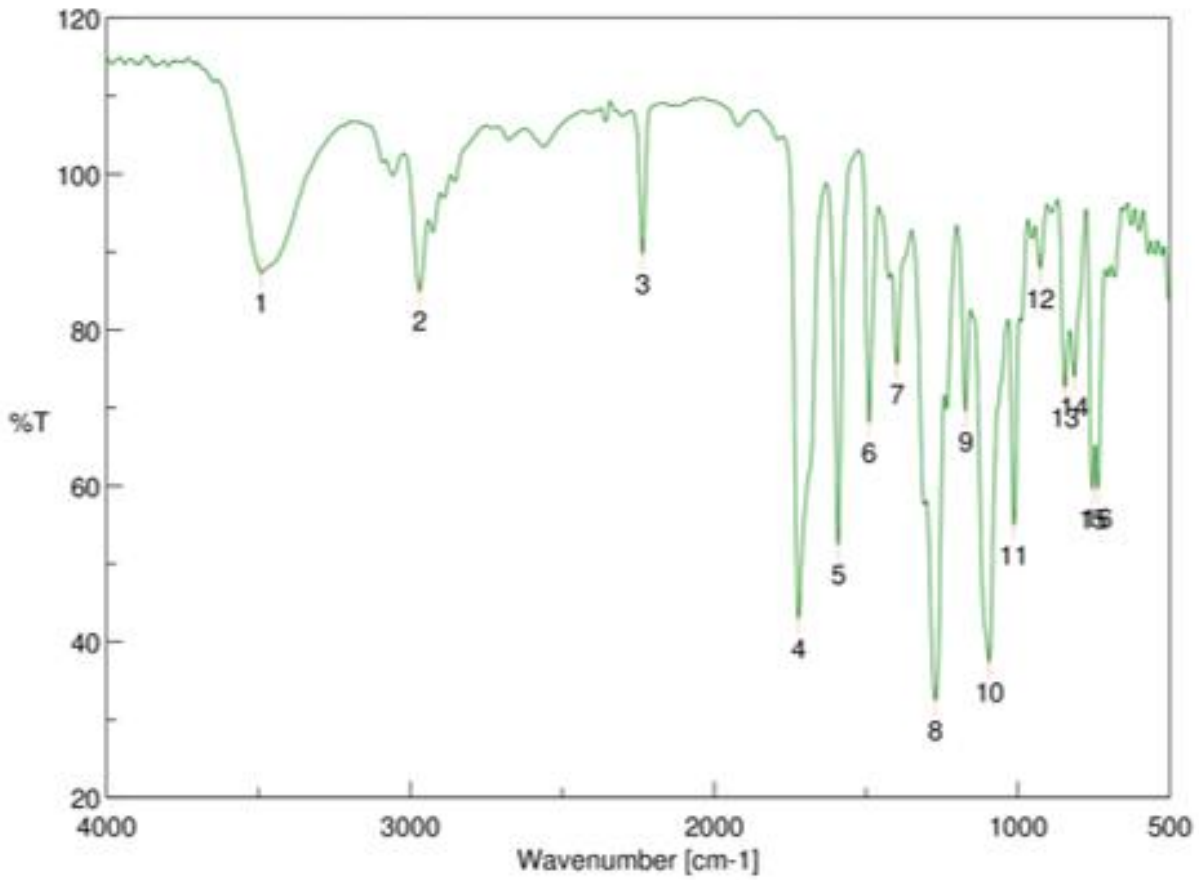

[ピーク検出結果]

No.	位置	強度	No.	位置	強度
1	3491.49	87.2967	2	2969.84	84.965
3	2236.06	89.745	4	1722.12	42.9537
5	1591.95	52.4301	6	1489.74	68.1009
7	1398.14	75.5772	8	1271.82	32.4969
9	1172.51	69.511	10	1094.4	37.4074
11	1013.41	54.9367	12	926.628	87.8369
13	845.633	72.5909	14	814.777	73.971
15	754.031	59.5945	16	737.639	59.6529

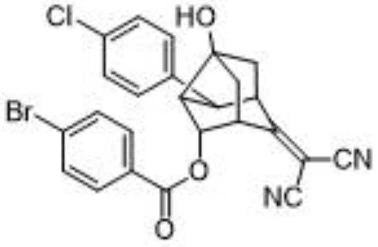

24d

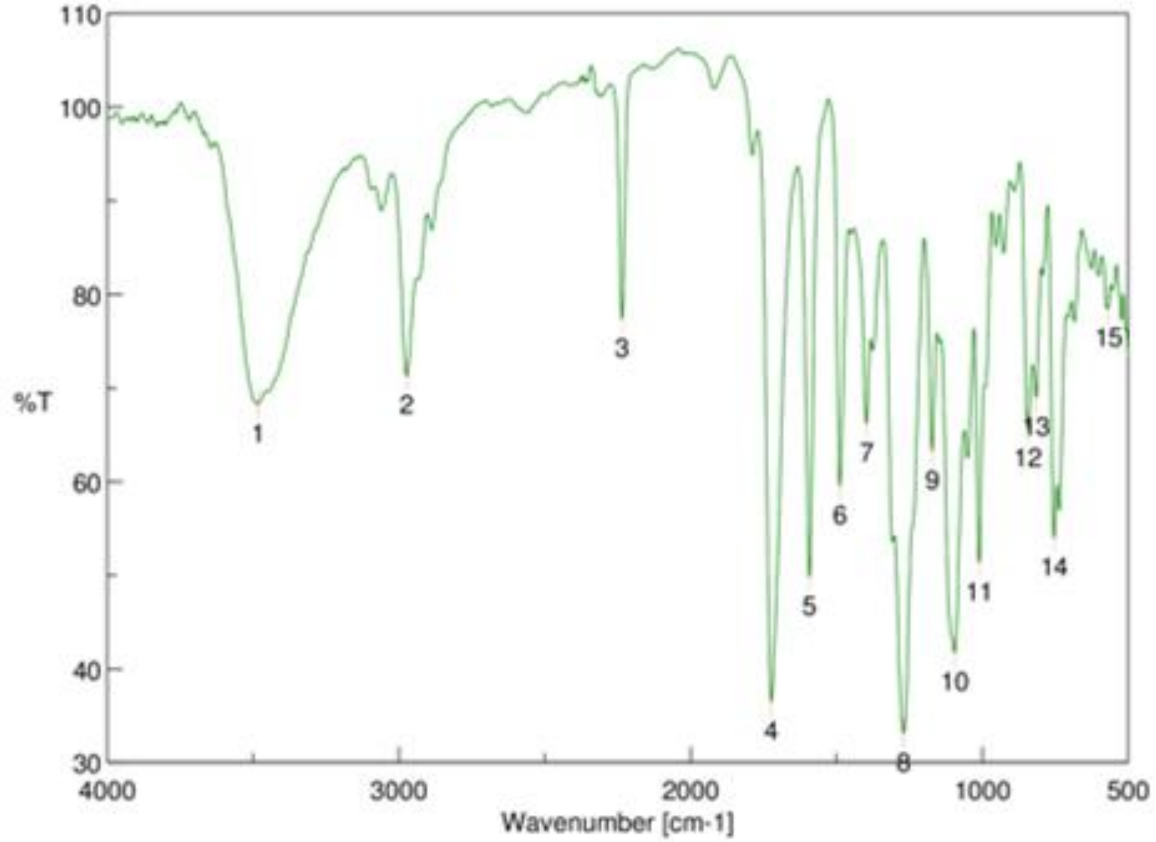

[ピーク検出結果]

No.	位置	強度	No.	位置	強度
1	3482.81	68.2612	2	2973.7	71.3043
3	2235.09	77.3731	4	1724.05	36.5216
5	1592.91	49.8328	6	1488.78	59.5467
7	1397.17	66.2241	8	1269.9	33.1139
9	1172.51	63.2686	10	1095.37	41.7483
11	1010.52	51.3648	12	844.669	65.6798
13	815.742	68.9697	14	754.031	54.0297
15	567.934	78.4947			

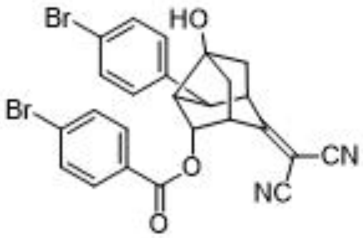

24e

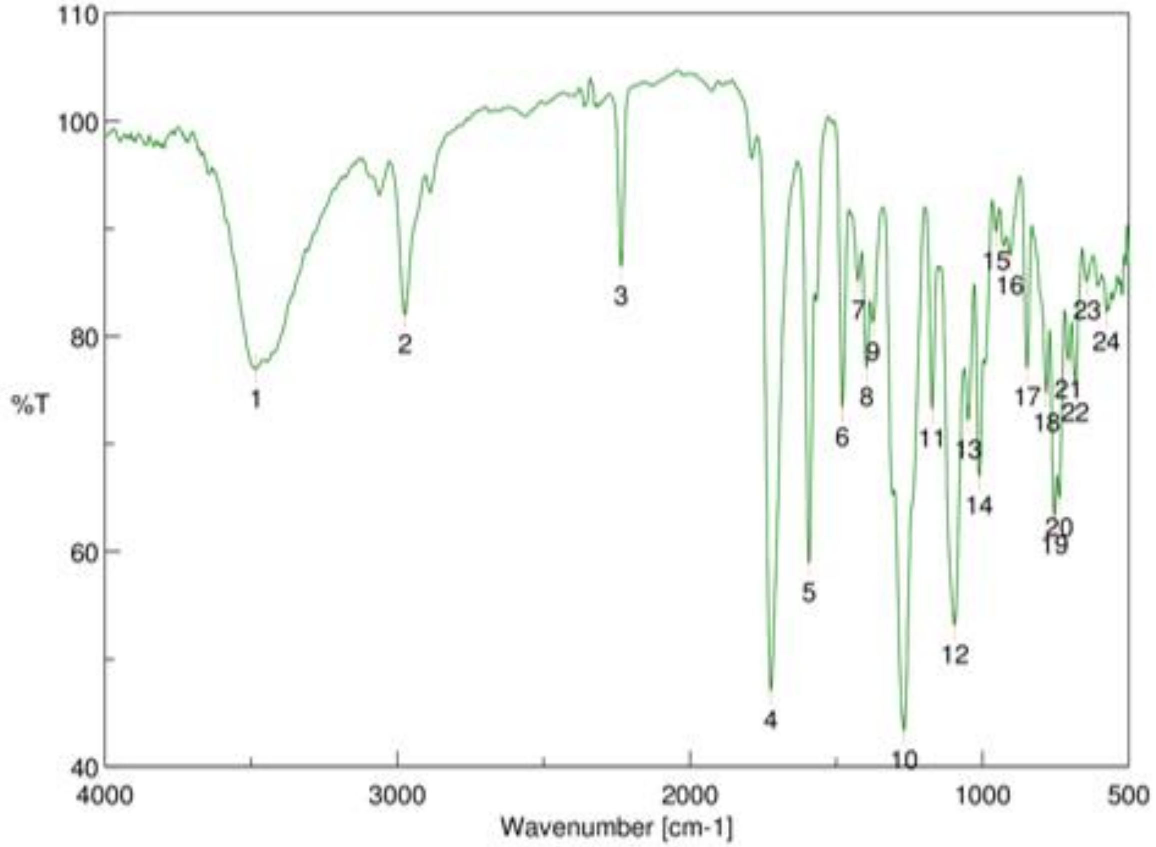

[ピーク検出結果]

No.	位置	強度	No.	位置	強度
1	3483.78	76.9398	2	2974.66	81.9716
3	2235.09	86.4858	4	1724.05	47.0673
5	1592.91	58.9451	6	1479.13	73.3758
7	1425.14	85.1616	8	1396.21	77.1024
9	1374.03	81.2845	10	1267.97	43.3591
11	1172.51	73.2372	12	1095.37	53.1739
13	1049.09	72.2318	14	1011.48	67.0134
15	953.627	89.7221	16	906.379	87.4949
17	847.561	77.0724	18	781.993	74.7045
19	754.995	63.3391	20	737.639	64.9975
21	705.819	77.774	22	682.677	75.6464
23	642.179	85.1236	24	575.647	82.2357

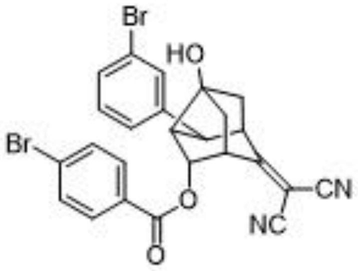

24f

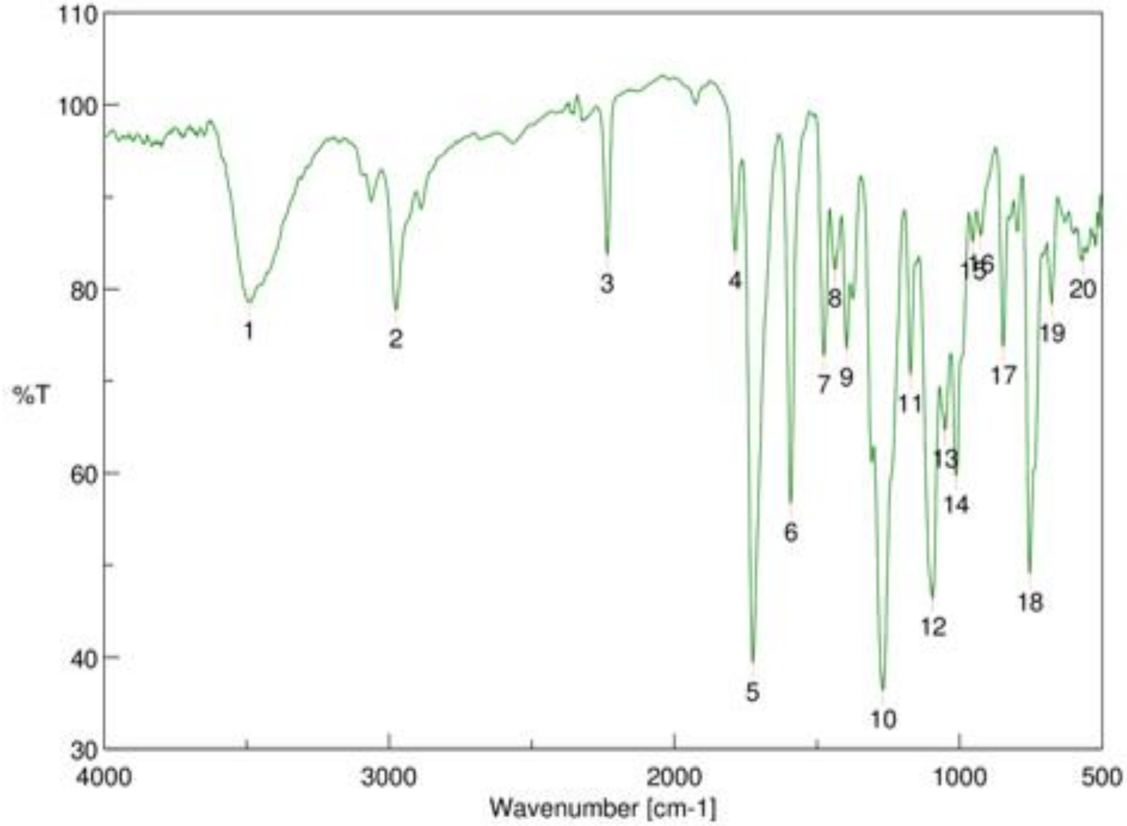

[ピーク検出結果]

No.	位置	強度	No.	位置	強度
1	3492.45	78.6031	2	2975.62	77.7165
3	2236.06	83.7189	4	1786.72	84.0556
5	1725.01	39.3094	6	1591.95	56.7097
7	1476.24	72.7461	8	1436.71	82.1064
9	1396.21	73.5309	10	1268.93	36.3861
11	1172.51	70.6613	12	1094.4	46.4112
13	1051.01	64.6578	14	1012.45	59.6866
15	953.627	85.1196	16	925.664	85.8388
17	846.597	73.7173	18	754.031	49.0629
19	676.892	78.2909	20	567.934	83.1024

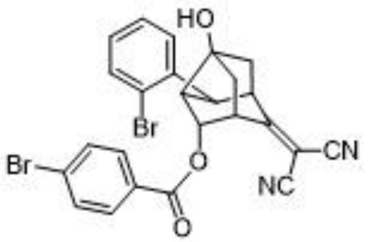

24g

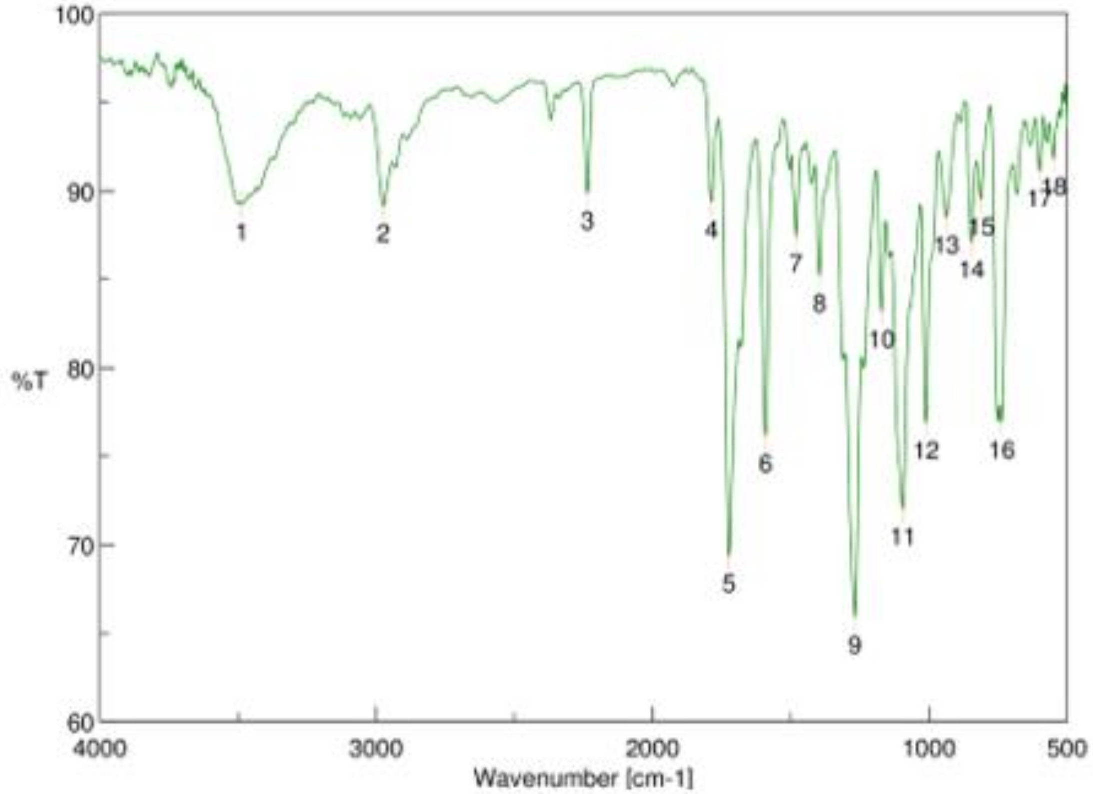

[ピーク検出結果]

No.	位置	強度	No.	位置	強度
1	3488.6	89.2363	2	2973.7	89.1388
3	2235.09	89.8539	4	1786.72	89.3604
5	1723.09	69.3444	6	1591.95	76.1459
7	1481.06	87.4117	8	1396.21	85.1796
9	1267	65.8989	10	1172.51	83.1658
11	1094.4	72.012	12	1010.52	76.8862
13	937.235	88.4801	14	845.633	87.0776
15	810.92	89.5054	16	737.639	76.9139
17	599.753	91.1203	18	548.649	91.784

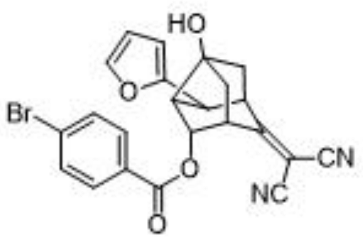

24h

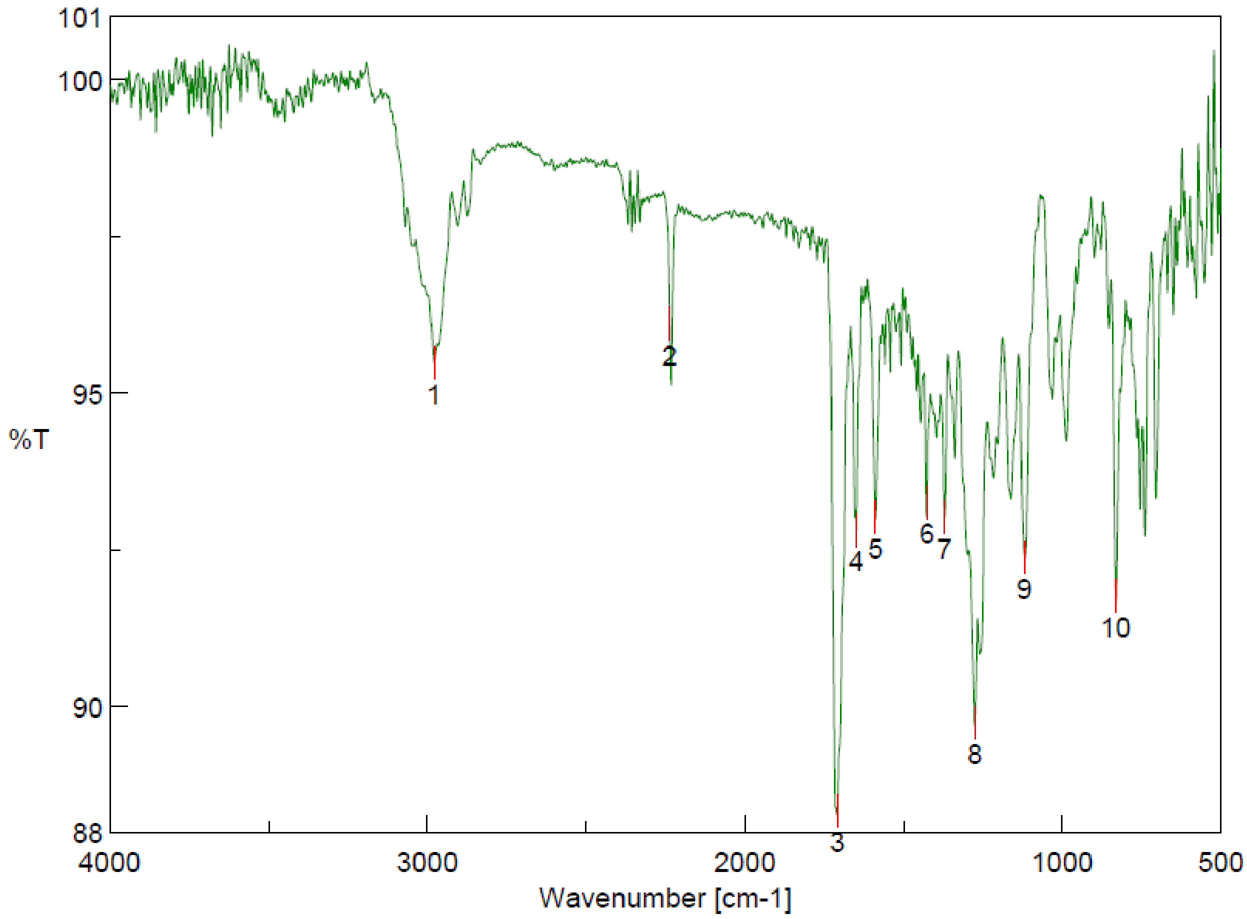

[ピーク検出結果]

No.	位置	強度	No.	位置	強度
1	2978.52	95.483	2	2236.06	96.1044
3	1708.62	88.3464	4	1648.84	92.809
5	1589.06	93.0133	6	1426.1	93.253
7	1370.18	93.0173	8	1274.72	89.7484
9	1118.51	92.3818	10	832.133	91.7642

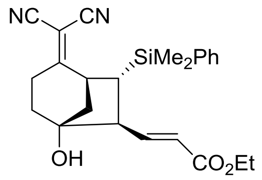

17i

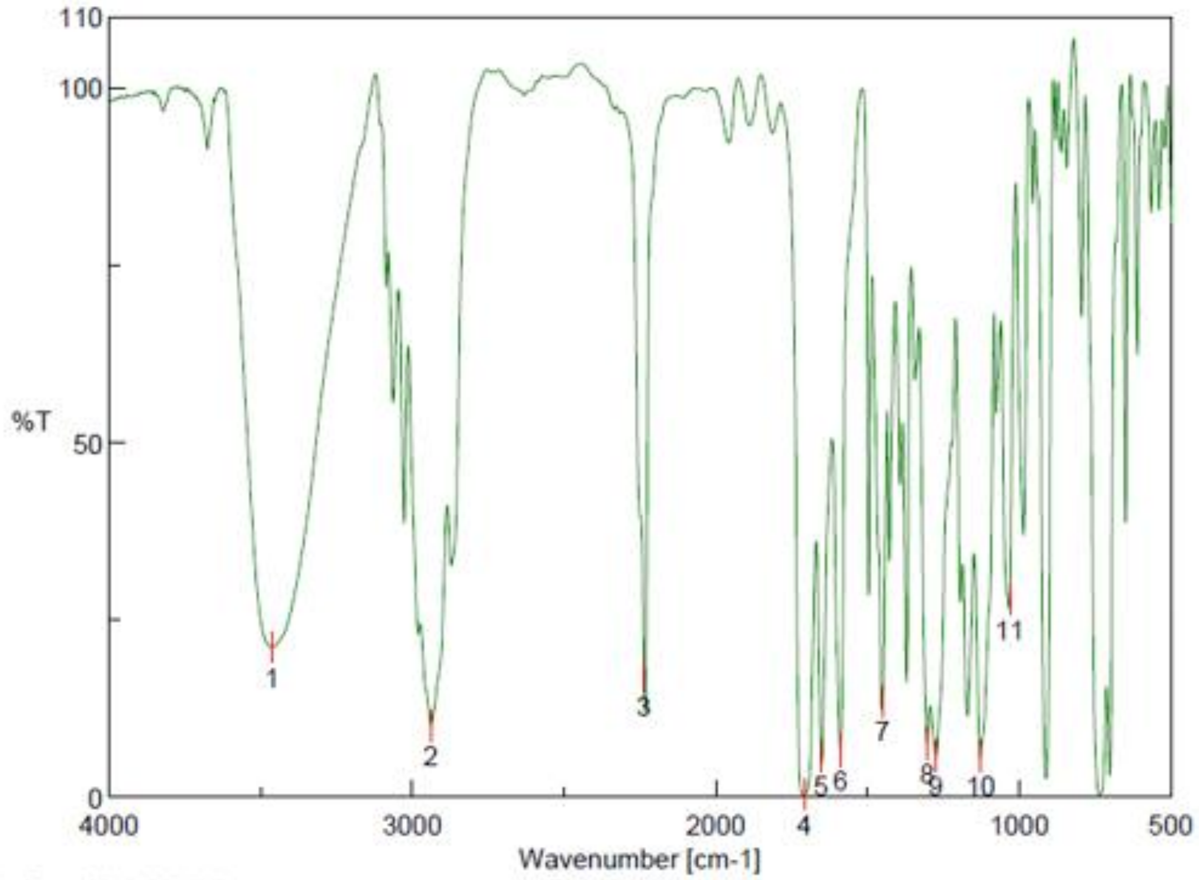

［ピーク検出結果］

No.	位置	強度	No.	位置	強度
1	3462.56	21.1152	2	2938.98	10.0286
3	2236.06	17.001	4	1708.62	0.368164
5	1652.7	5.69139	6	1589.06	6.47535
7	1451.17	13.4461	8	1302.68	7.51137
9	1274.72	5.76774	10	1126.22	5.81212
11	1030.77	27.847			

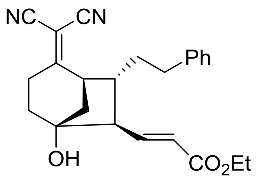

17j

7. HPLC data

A mixture of both enantiomers of **4a**

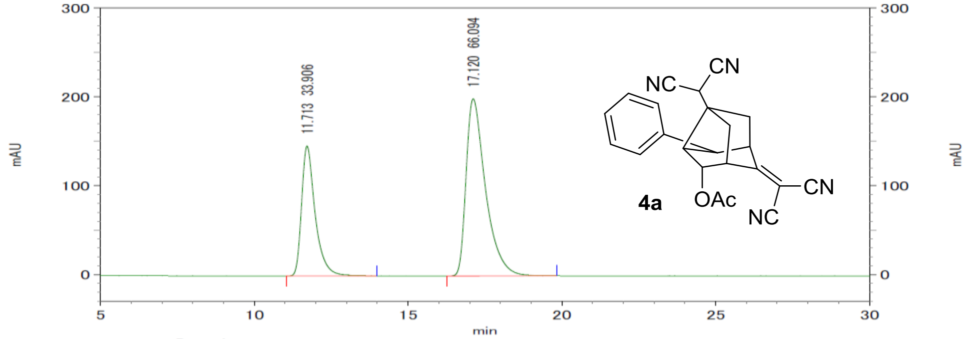

Result
1： 233.0 nm, 4.0 nm 結果

Pk #	Retention time / min	Integration/ %
1	11.713	33.906
2	17.120	66.094
合計 Total		100.000

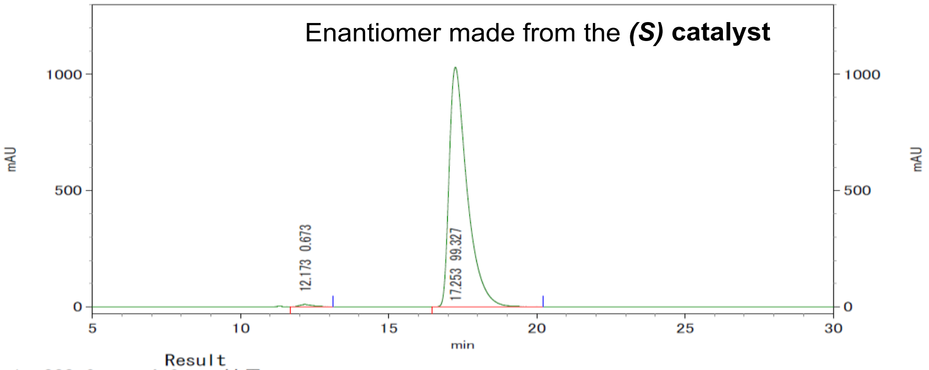

Result
1： 233.0 nm, 4.0 nm 結果

Pk #	Retention time / min	Integration/ %
1	12.173	0.673
2	17.253	99.327
合計 Total		100.000

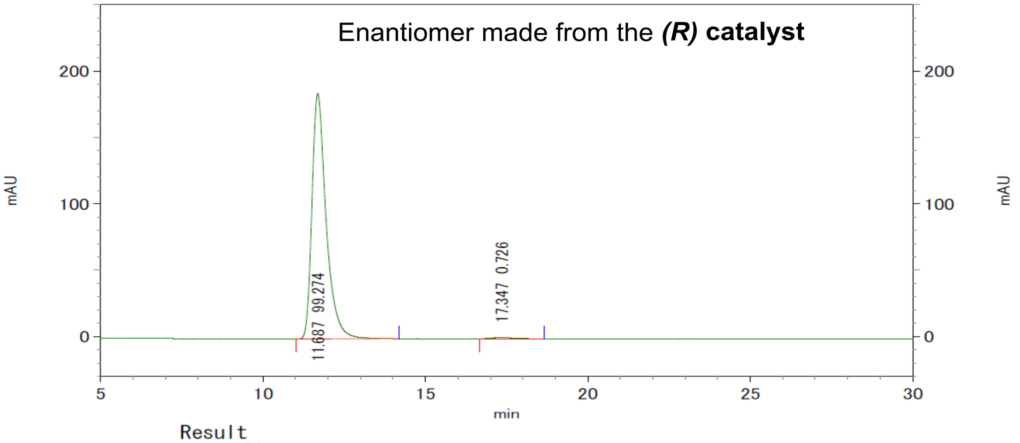

Result
1： 233.0 nm, 4.0 nm 結果

Pk #	Retention time / min	Integration/ %
1	11.687	99.274
2	17.347	0.726
合計 Total		100.000

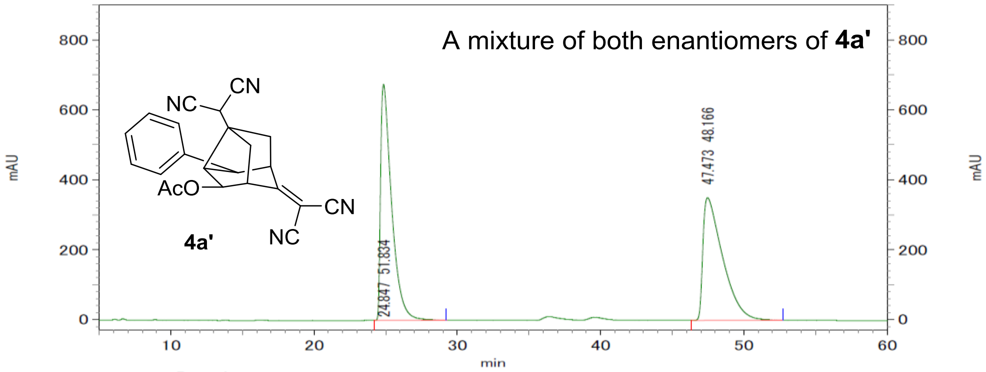

Result
1： 233.0 nm, 4.0 nm 結果

Pk #	Retention time / min	Integration/ %
1	24.847	51.834
2	47.473	48.166
合計 Total		100.000

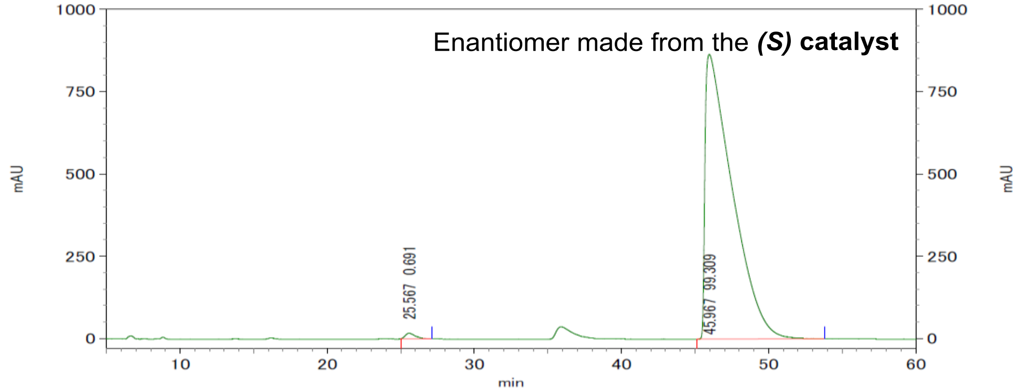

Result
1： 233.0 nm, 4.0 nm 結果

Pk #	Retention time / min	Integration/ %
1	25.567	0.691
2	45.967	99.309
合計 Total		100.000

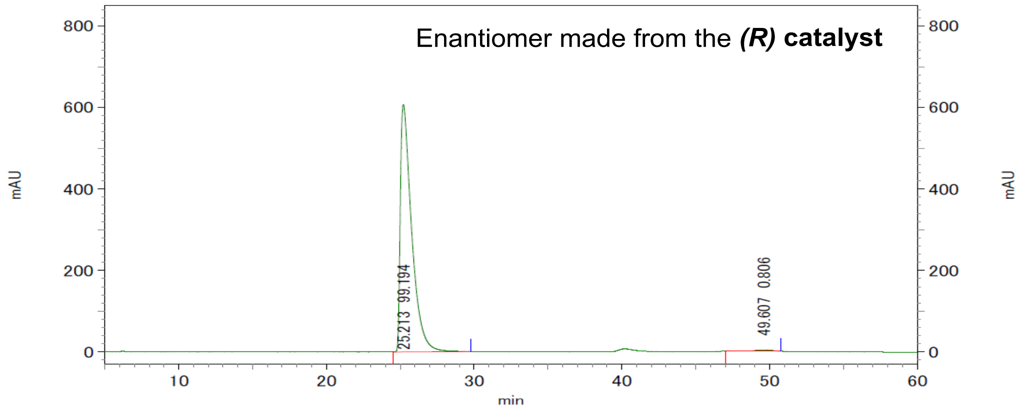

Result
1： 233.0 nm, 4.0 nm 結果

Pk #	Retention time / min	Integration/ %
1	25.213	99.194
2	49.607	0.806
合計 Total		100.000

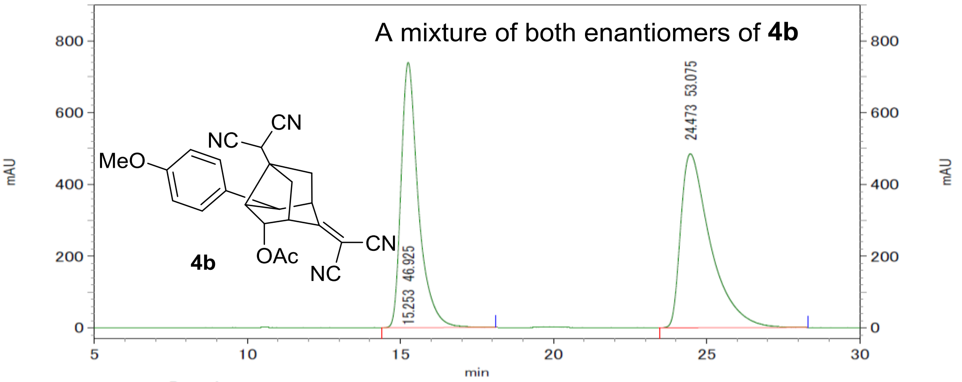

Result
1: 233.0 nm, 4.0 nm 結果

Pk #	Retention time / min	Integration/ %
1	15.253	46.925
2	24.473	53.075
合計 Total		100.000

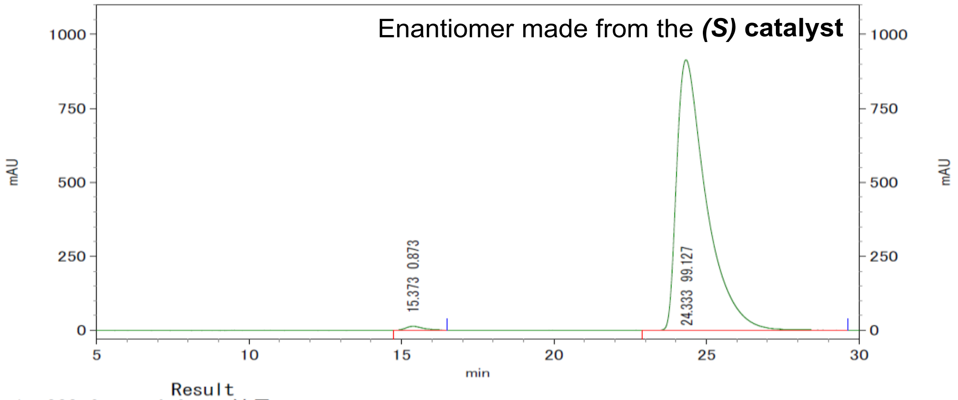

Result
1: 233.0 nm, 4.0 nm 結果

Pk #	Retention time / min	Integration/ %
1	15.373	0.873
2	24.333	99.127
合計 Total		100.000

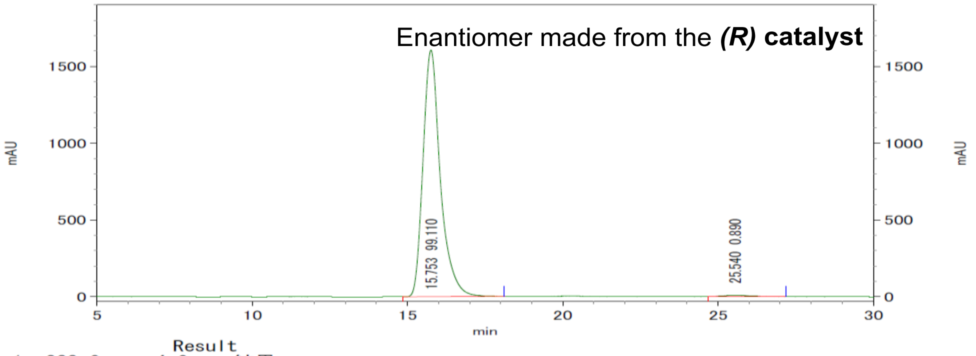

Result
1: 233.0 nm, 4.0 nm 結果

Pk #	Retention time / min	Integration/ %
1	15.753	99.110
2	25.540	0.890
合計 Total		100.000

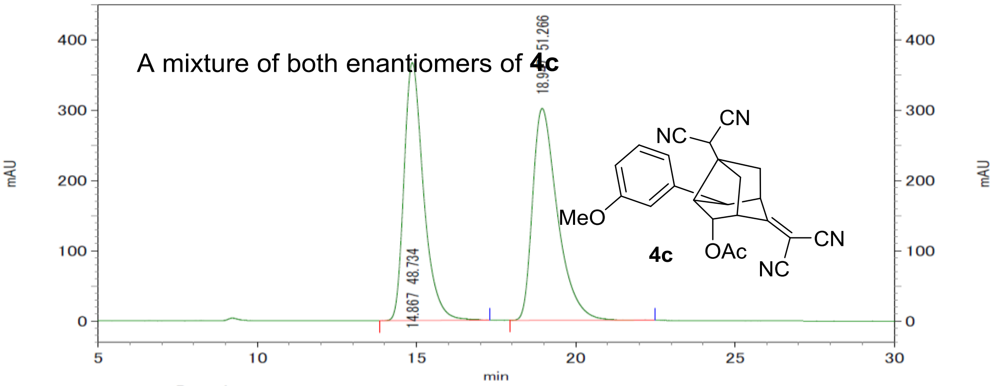

Result
1： 233.0 nm, 4.0 nm 結果

Pk #	Retention time / min	Integration/ %
1	14.867	48.734
2	18.947	51.266
合計 Total		100.000

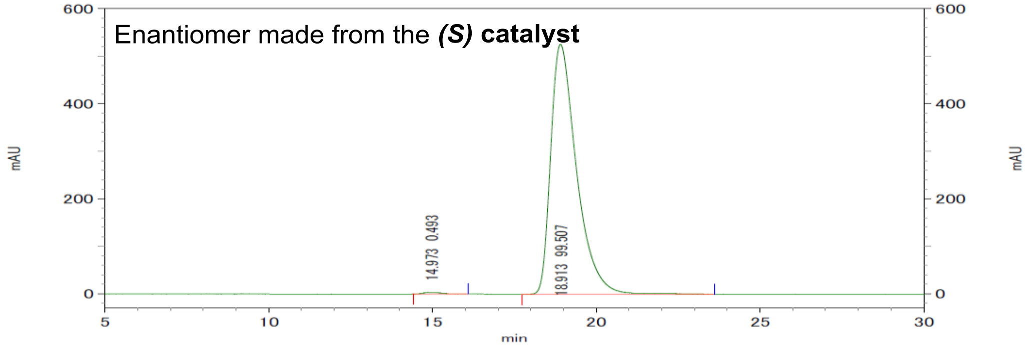

Result
1： 233.0 nm, 4.0 nm 結果

Pk #	Retention time / min	Integration/ %
1	14.973	0.493
2	18.913	99.507
合計 Total		100.000

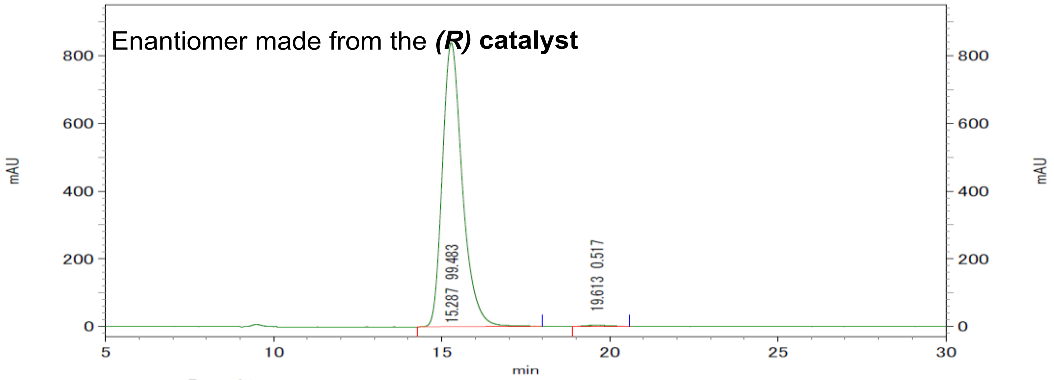

Result
1： 233.0 nm, 4.0 nm 結果

Pk #	Retention time / min	Integration/ %
1	15.287	99.483
2	19.613	0.517
合計 Total		100.000

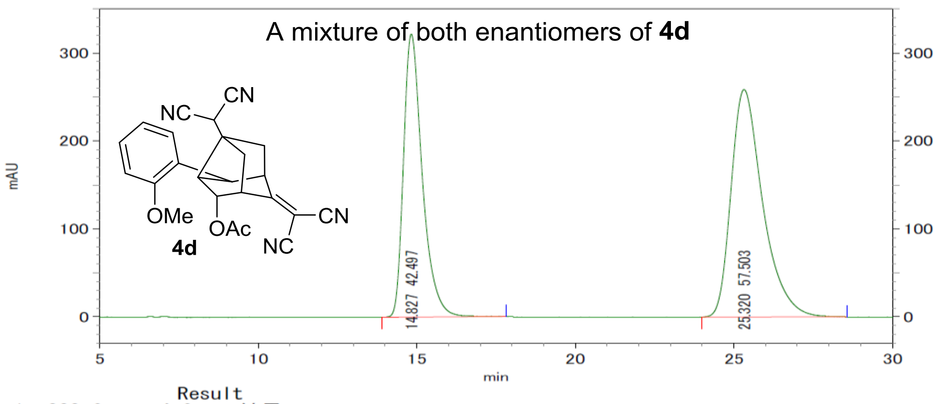

Result
1: 233.0 nm, 4.0 nm 結果

Pk #	Retention time / min	Integration/ %
1	14.827	42.497
2	25.320	57.503
合計 Total		100.000

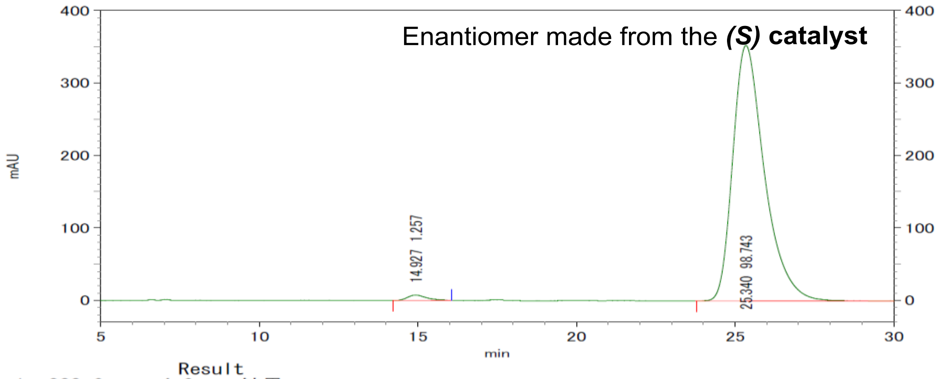

Result
1: 233.0 nm, 4.0 nm 結果

Pk #	Retention time / min	Integration/ %
1	14.927	1.257
2	25.340	98.743
合計 Total		100.000

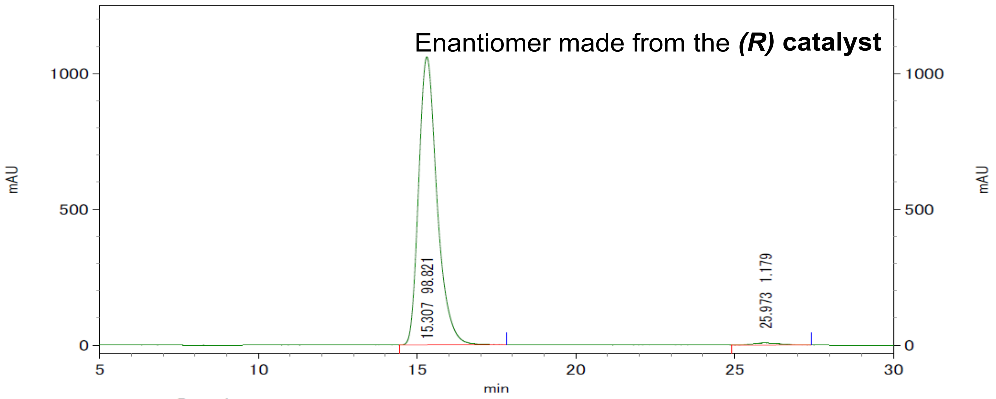

Result
1: 233.0 nm, 4.0 nm 結果

Pk #	Retention time / min	Integration/ %
1	15.307	98.821
2	25.973	1.179
合計 Total		100.000

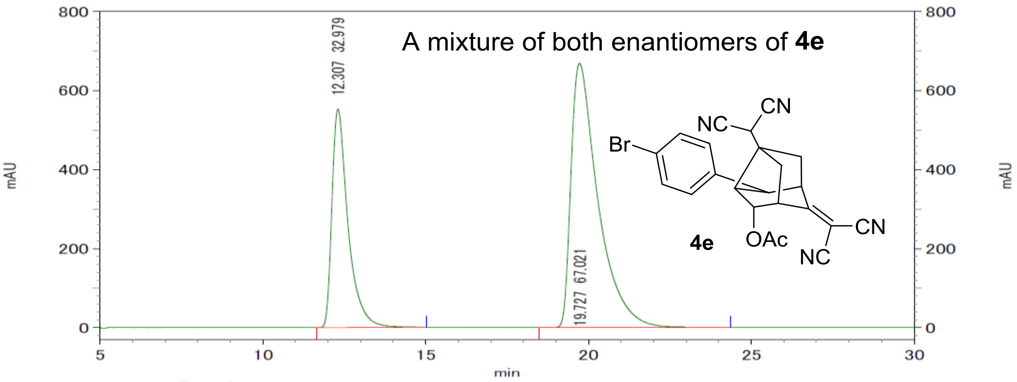

Result
1： 233.0 nm, 4.0 nm 結果

Pk #	Retention time / min	Integration/ %
1	12.307	32.979
2	19.727	67.021
合計 Total		100.000

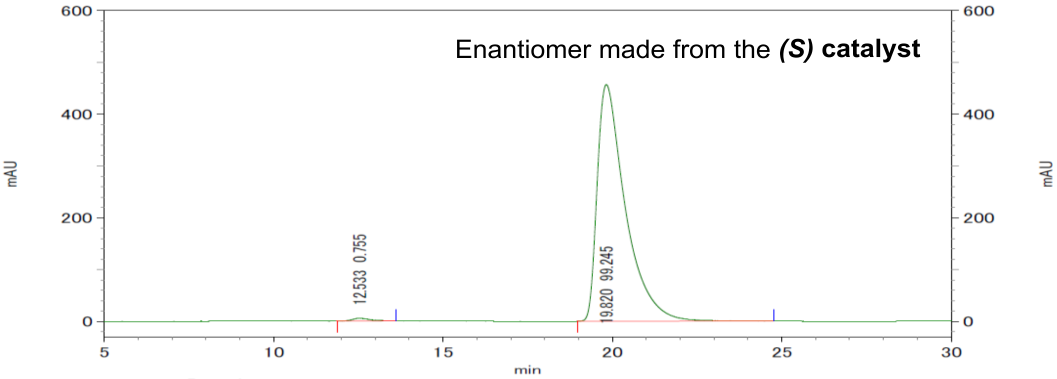

Result
1： 233.0 nm, 4.0 nm 結果

Pk #	Retention time / min	Integration/ %
1	12.533	0.755
2	19.820	99.245
合計 Total		100.000

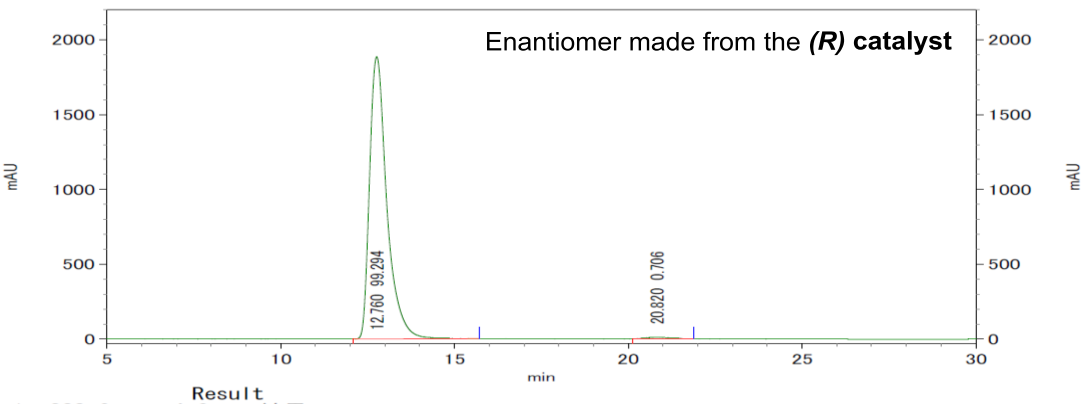

Result
1： 233.0 nm, 4.0 nm 結果

Pk #	Retention time / min	Integration/ %
1	12.760	99.294
2	20.820	0.706
合計 Total		100.000

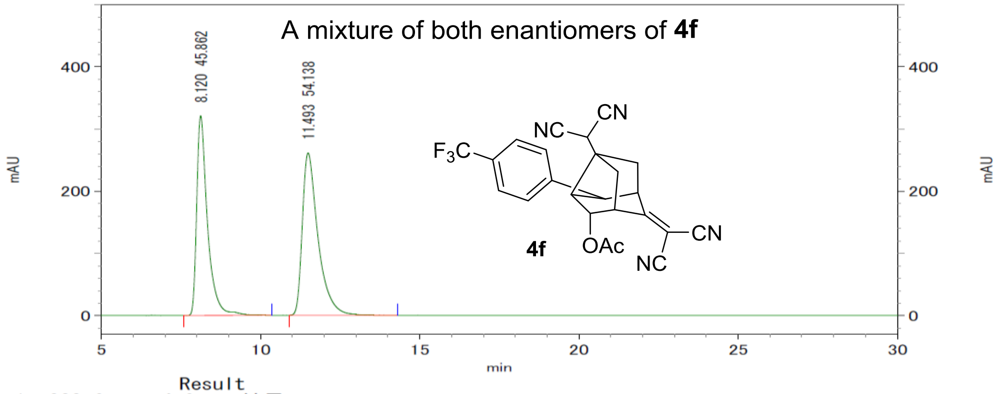

Result
1： 233.0 nm, 4.0 nm 結果

Pk #	Retention time / min	Integration/ %
1	8.120	45.862
2	11.493	54.138
合計 Total		100.000

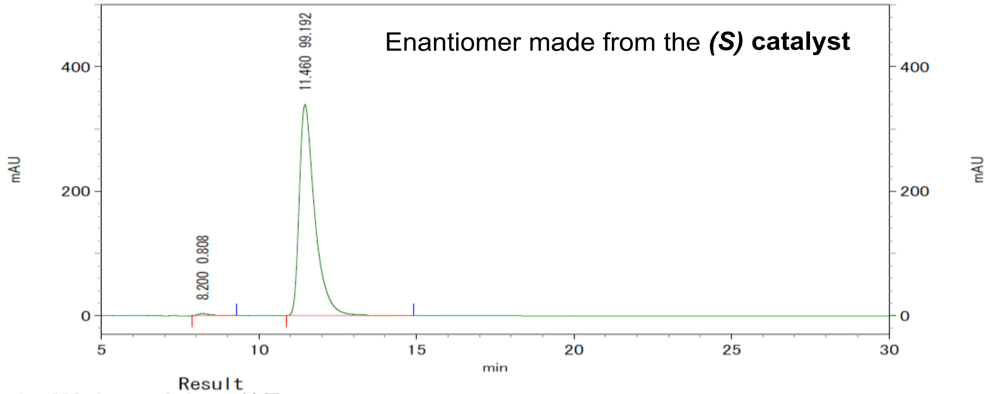

Result
1： 233.0 nm, 4.0 nm 結果

Pk #	Retention time / min	Integration/ %
1	8.200	0.808
2	11.460	99.192
合計 Total		100.000

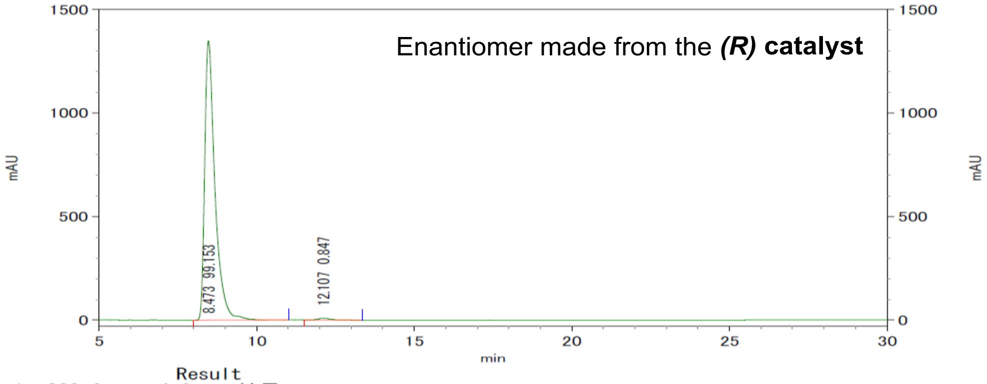

Result
1： 233.0 nm, 4.0 nm 結果

Pk #	Retention time / min	Integration/ %
1	8.473	99.153
2	12.107	0.847
合計 Total		100.000

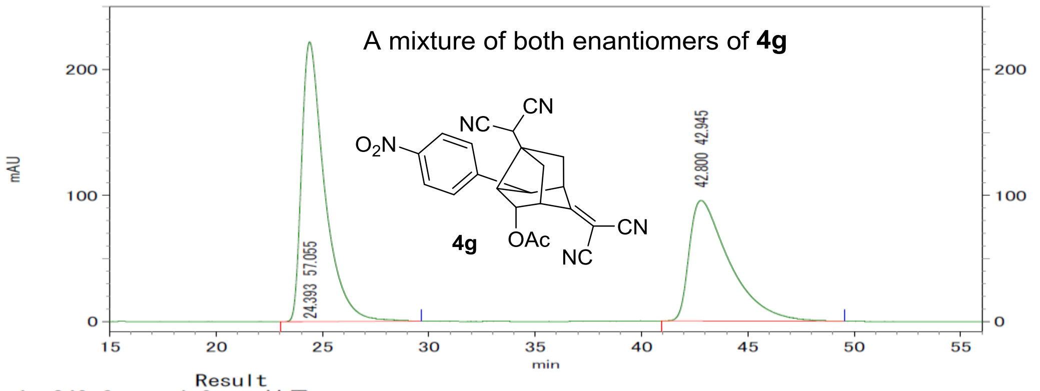

Result
4: 249.0 nm, 4.0 nm 結果

Pk #	Retention time / min	Integration/ %
1	24.393	57.055
2	42.800	42.945
合計 Total		100.000

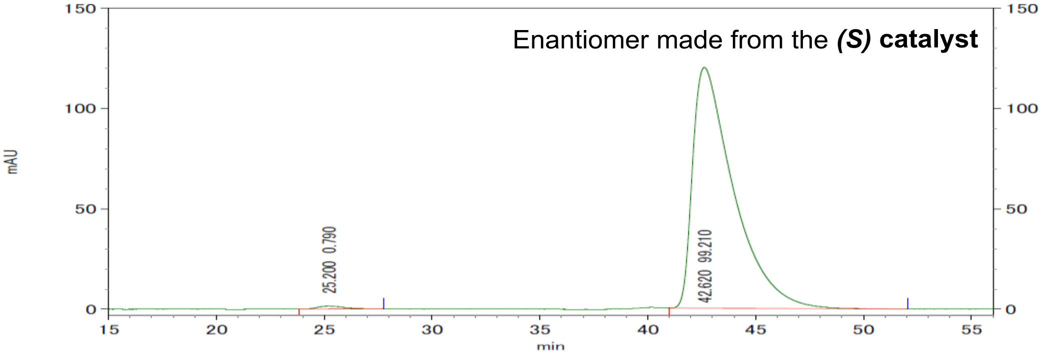

Result
4: 249.0 nm, 4.0 nm 結果

Pk #	Retention time / min	Integration/ %
1	25.200	0.790
2	42.620	99.210
合計 Total		100.000

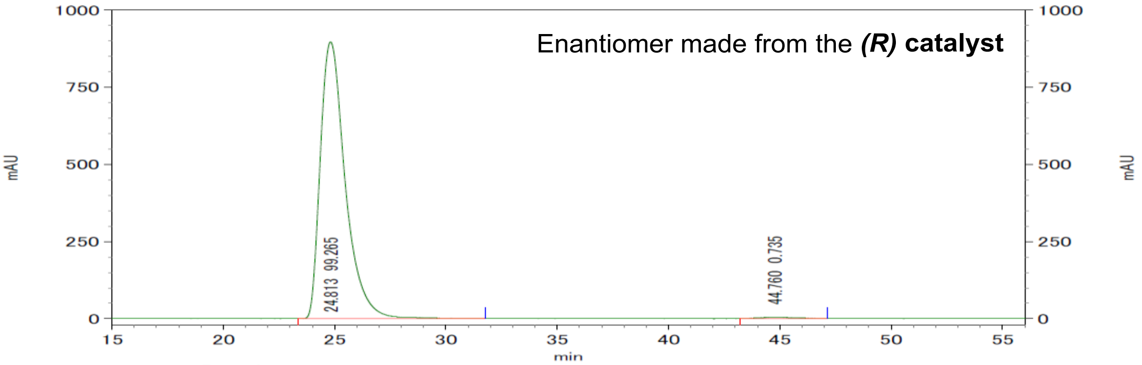

Result
4: 249.0 nm, 4.0 nm 結果

Pk #	Retention time / min	Integration/ %
1	24.813	99.265
2	44.760	0.735
合計 Total		100.000

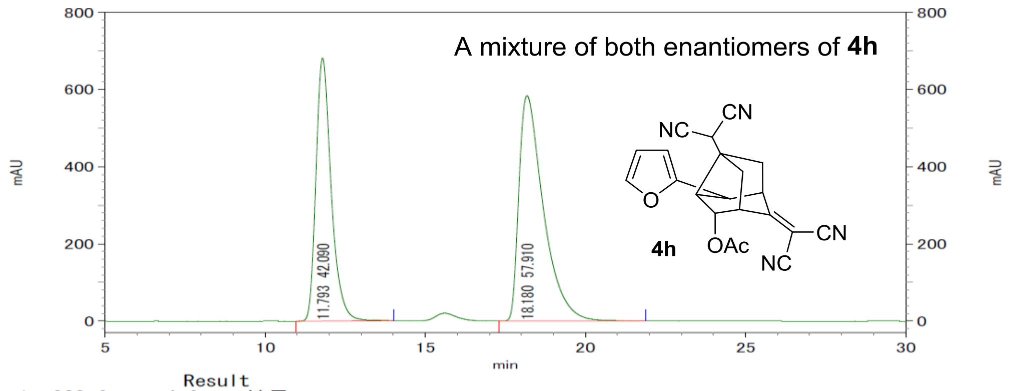

Result
1： 233.0 nm, 4.0 nm 結果

Pk #	Retention time / min	Integration/ %
1	11.793	42.090
2	18.180	57.910
合計 Total		100.000

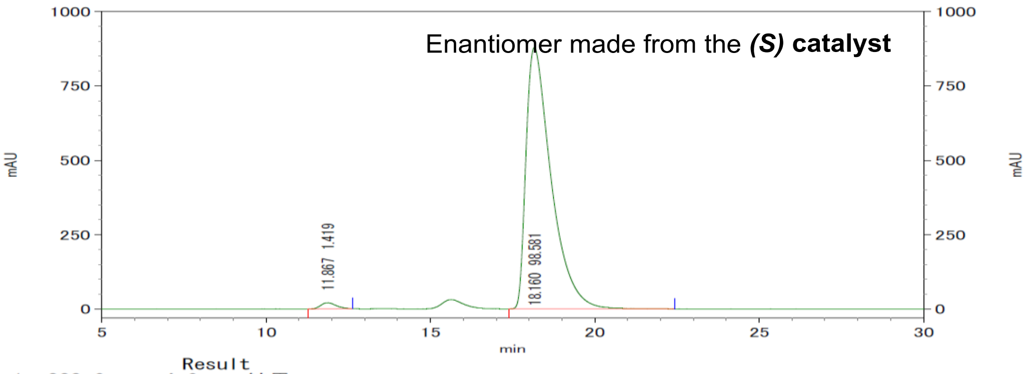

Result
1： 233.0 nm, 4.0 nm 結果

Pk #	Retention time / min	Integration/ %
1	11.867	1.419
2	18.160	98.581
合計 Total		100.000

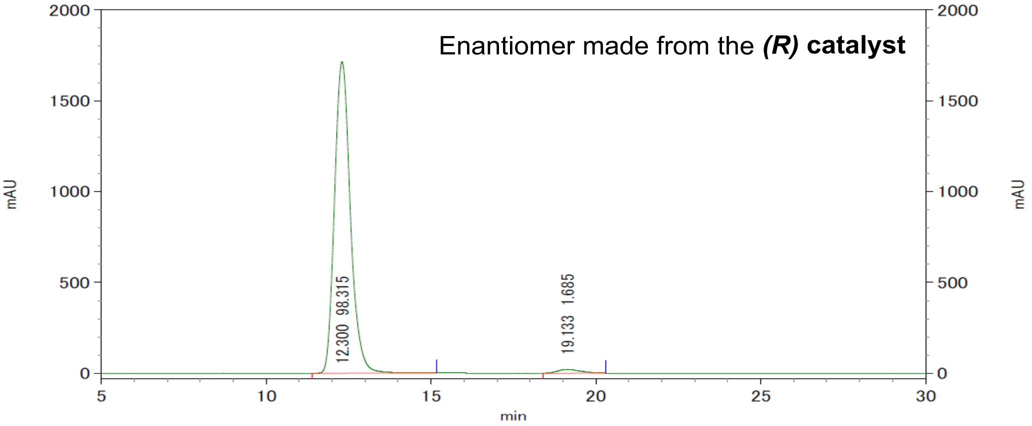

Result
1： 233.0 nm, 4.0 nm 結果

Pk #	Retention time / min	Integration/ %
1	12.300	98.315
2	19.133	1.685
合計 Total		100.000

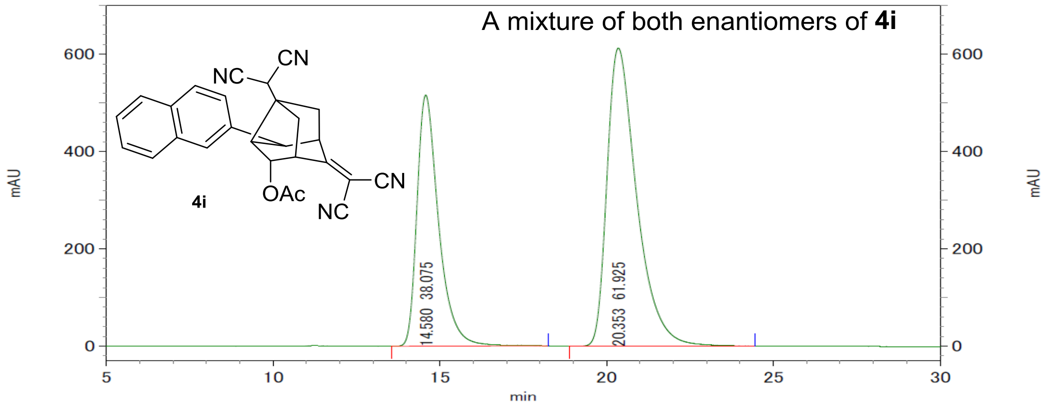

Result
2: 226.0 nm, 4.0 nm 結果

Pk #	Retention time / min	Integration/ %
1	14.580	38.075
2	20.353	61.925
合計 Total		100.000

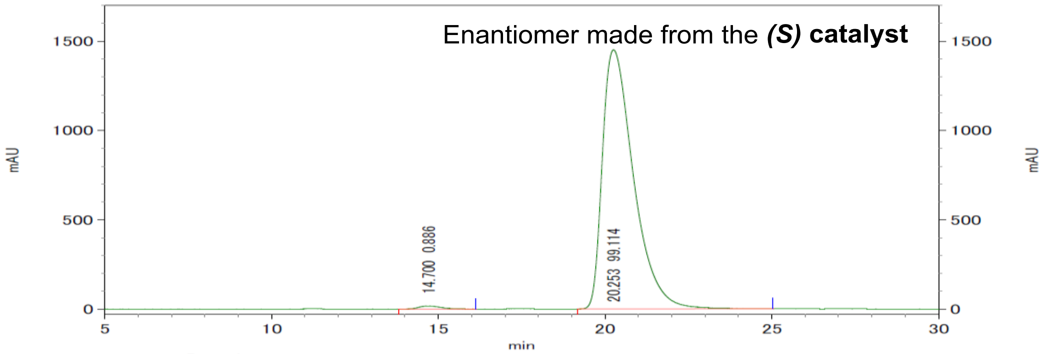

Result
2: 226.0 nm, 4.0 nm 結果

Pk #	Retention time / min	Integration/ %
1	14.700	0.886
2	20.253	99.114
合計 Total		100.000

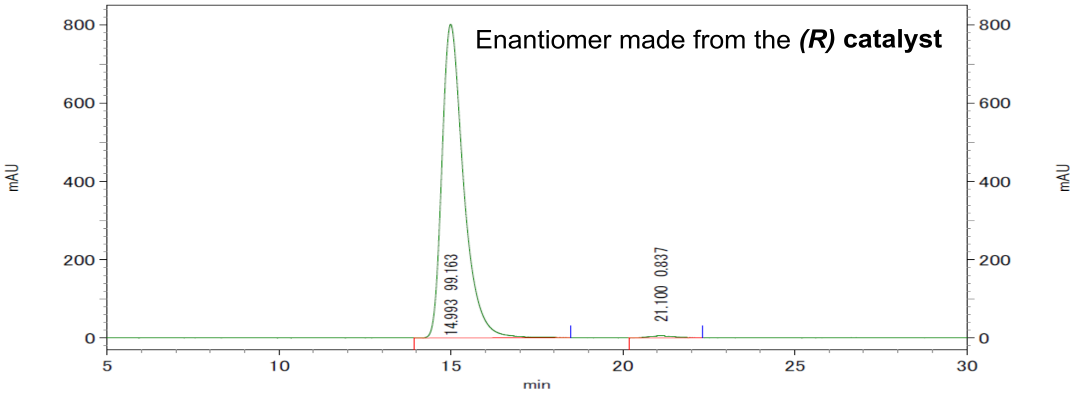

Result
2: 226.0 nm, 4.0 nm 結果

Pk #	Retention time / min	Integration/ %
1	14.993	99.163
2	21.100	0.837
合計 Total		100.000

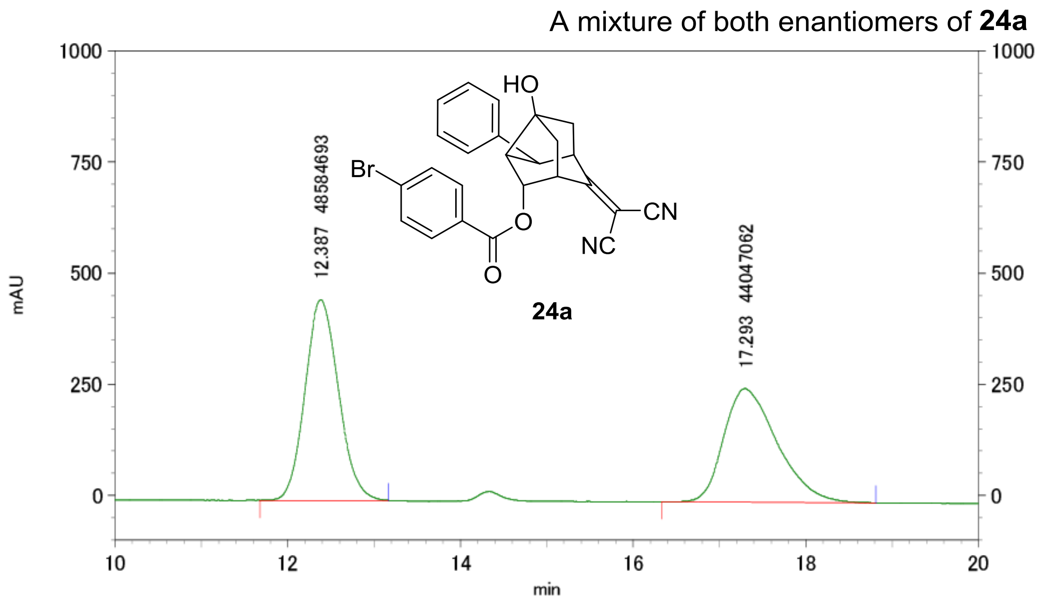

Result
2: 225 nm, 4 nm結果

Pk #	Retention time / min	Integration/ %
1	12.387	52.449
2	17.293	47.551
トータル Total		100.000

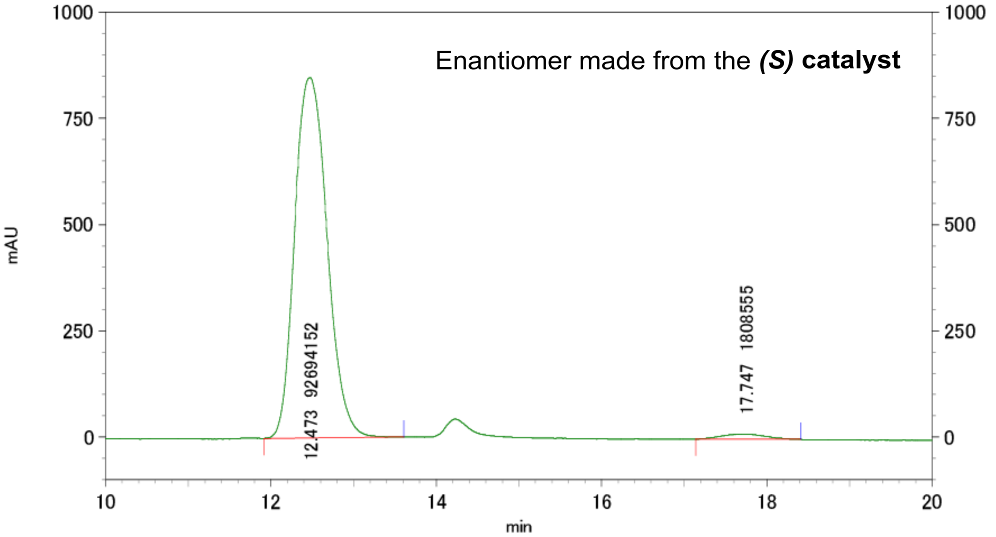

Result
2: 225 nm, 4 nm結果

Pk #	Retention time / min	Integration/ %
1	12.473	98.086
2	17.747	1.914
トータル Total		100.000

A mixture of both enantiomers of **24b**

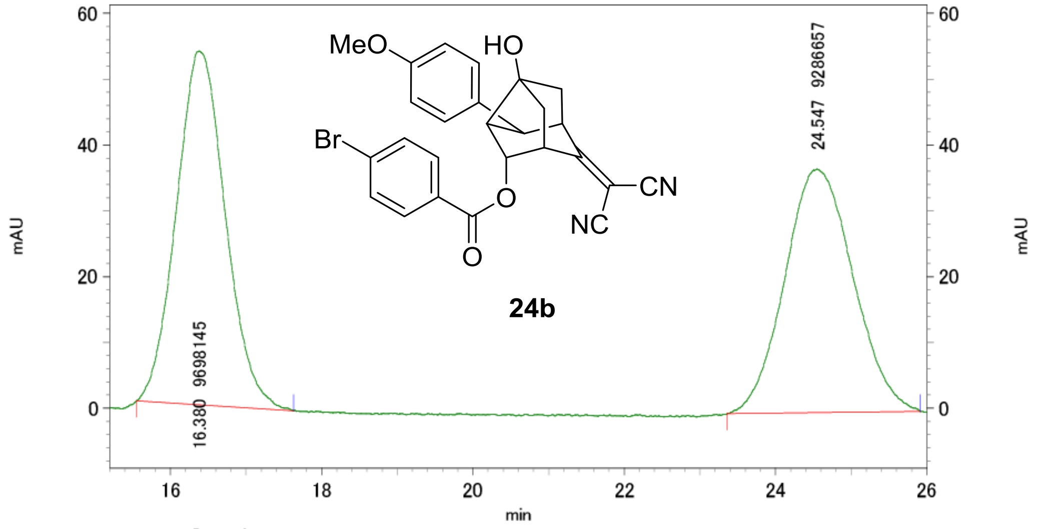

Result
2: 299 nm, 4 nm結果

Pk #	Retention time / min	Integration/ %
1	16.380	51.084
2	24.547	48.916
トータル<br>Total		100.000

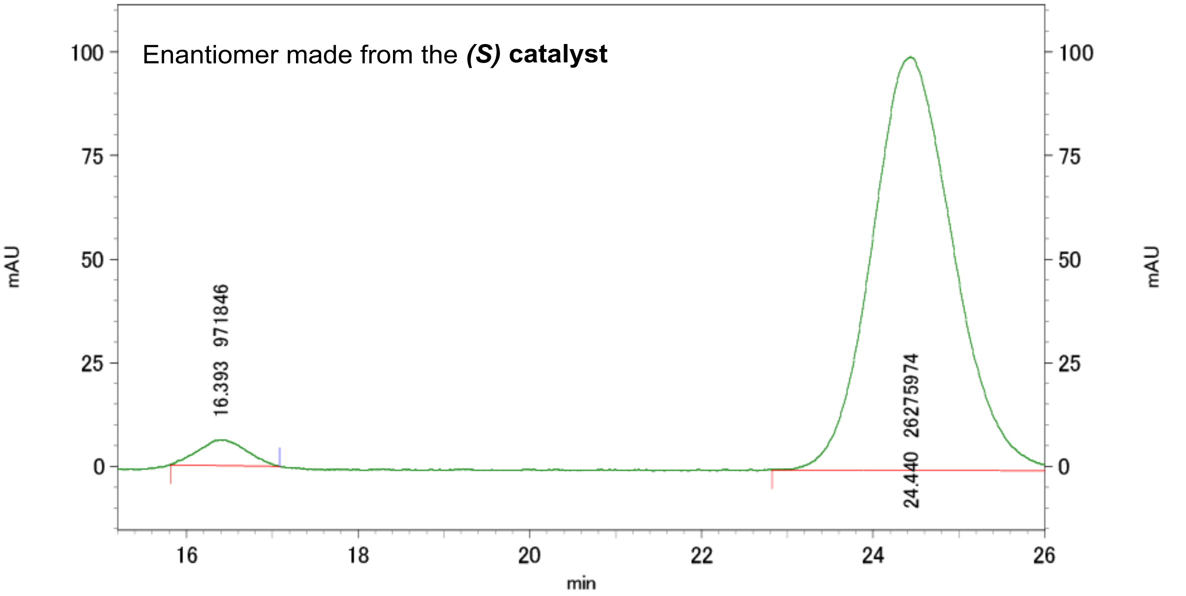

Result
2: 299 nm, 4 nm結果

Pk #	Retention time / min	Integration/ %
1	16.393	3.567
2	24.440	96.433
トータル<br>Total		100.000

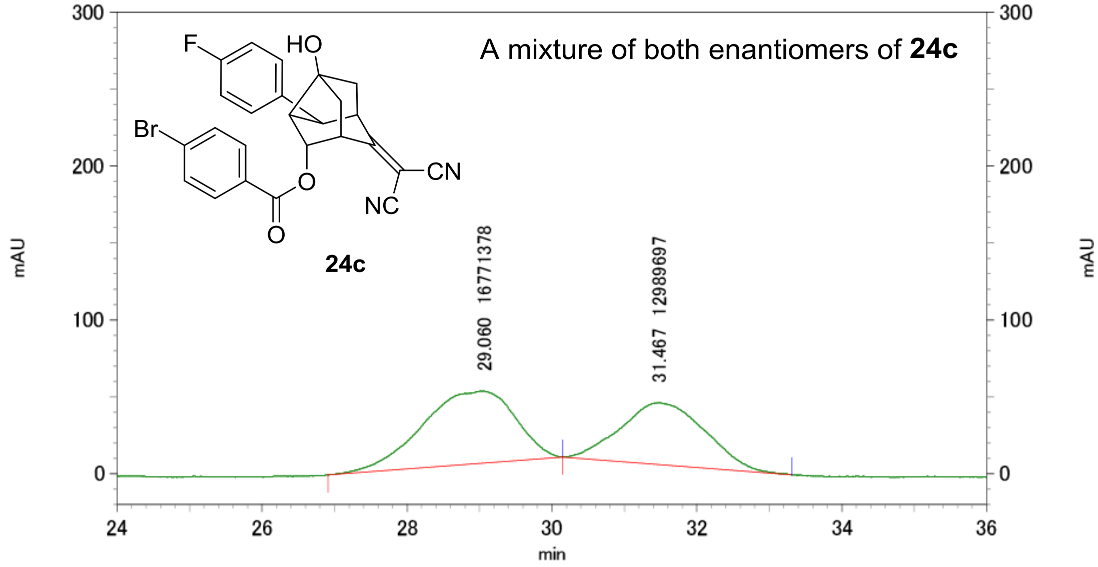

Result
2: 283 nm, 4 nm結果

Pk #	Retention time / min	Integration/ %
1	29.060	56.353
2	31.467	43.647
トータル Total		100.000

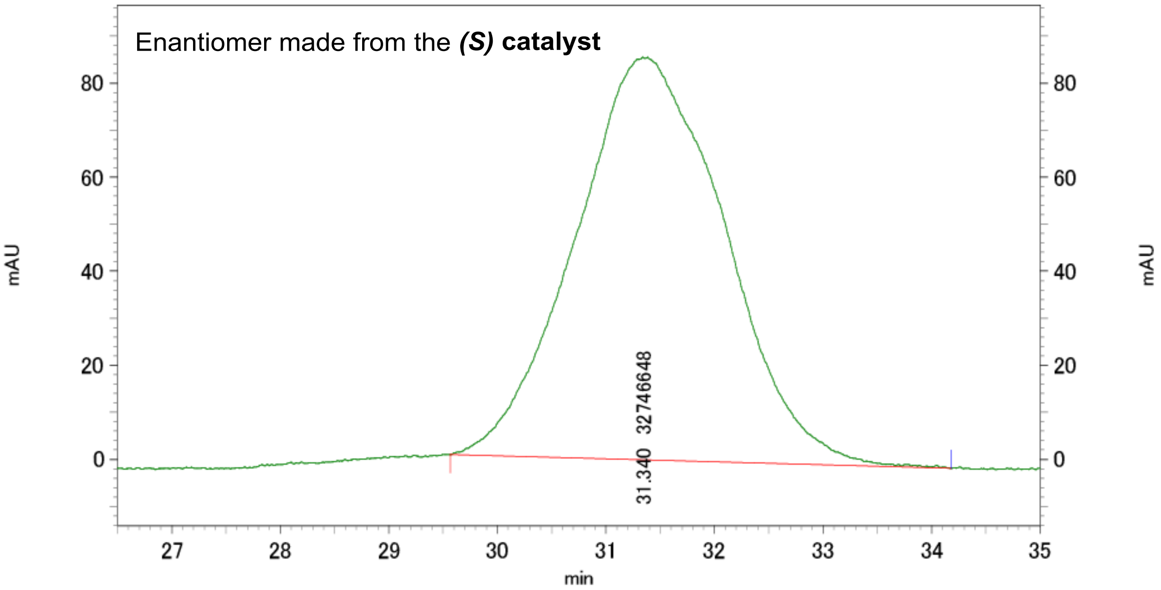

Result
2: 283 nm, 4 nm結果

Pk #	Retention time / min	Integration/ %
1	31.340	100.000
トータル Total		100.000

A mixture of both enantiomers of **24d**

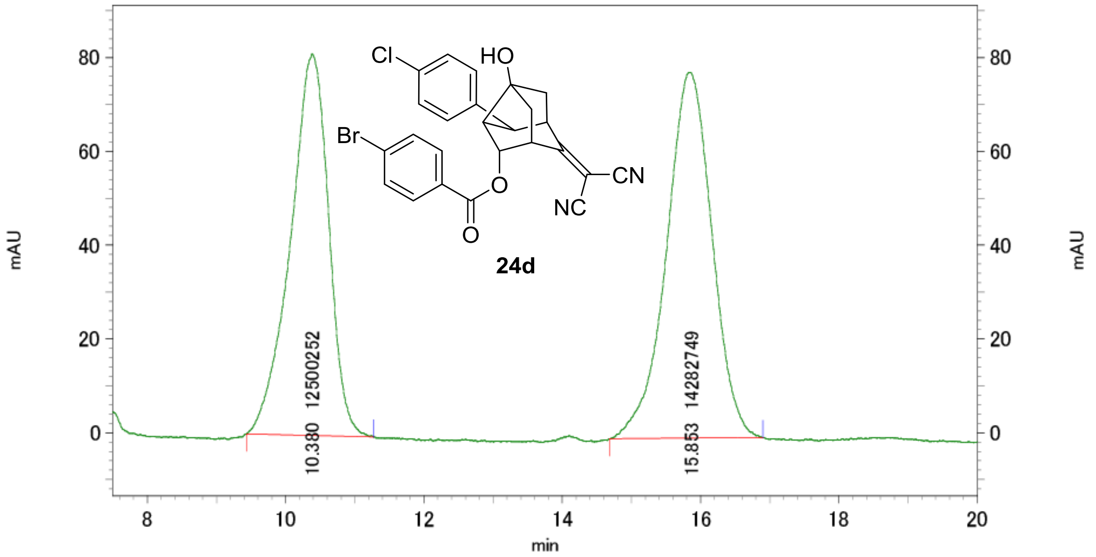

Result
2: 267 nm, 4 nm結果

Pk #	Retention time / min	Integration/ %
1	10.380	46.672
2	15.853	53.328
トータル Total		100.000

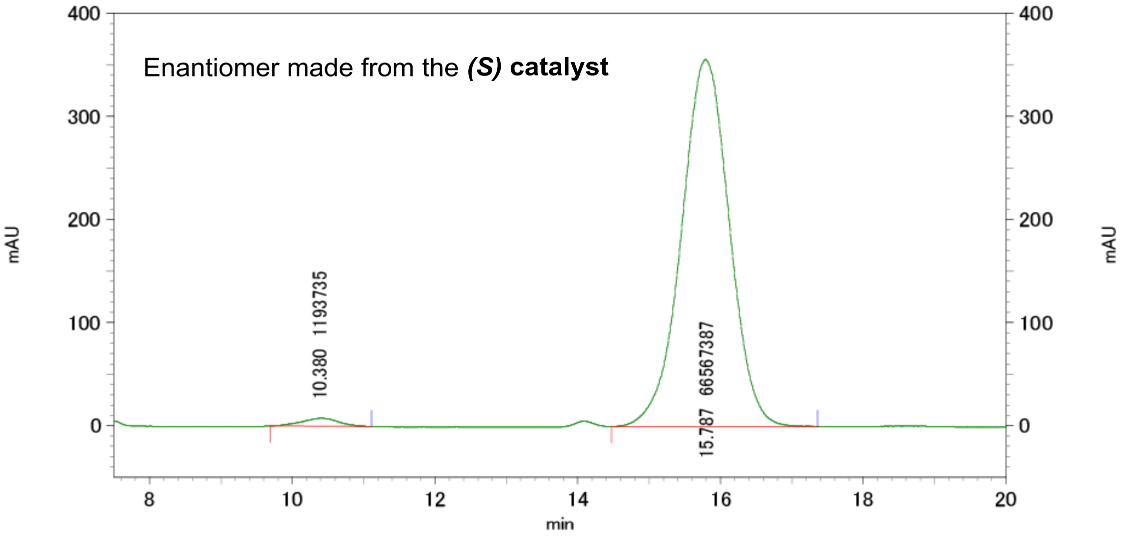

Result
2: 267 nm, 4 nm結果

Pk #	Retention time / min	Integration/ %
1	10.380	1.762
2	15.787	98.238
トータル Total		100.000

A mixture of both enantiomers of **24e**

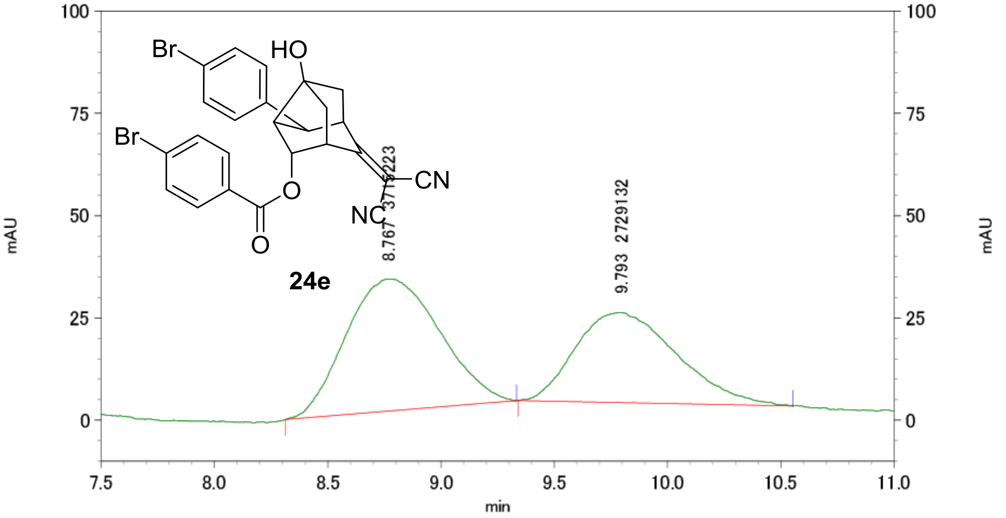

Result
2: 282 nm, 4 nm結果

Pk #	Retention time / min	Integration/ %
1	8.767	57.651
2	9.793	42.349
トータル Total		100.000

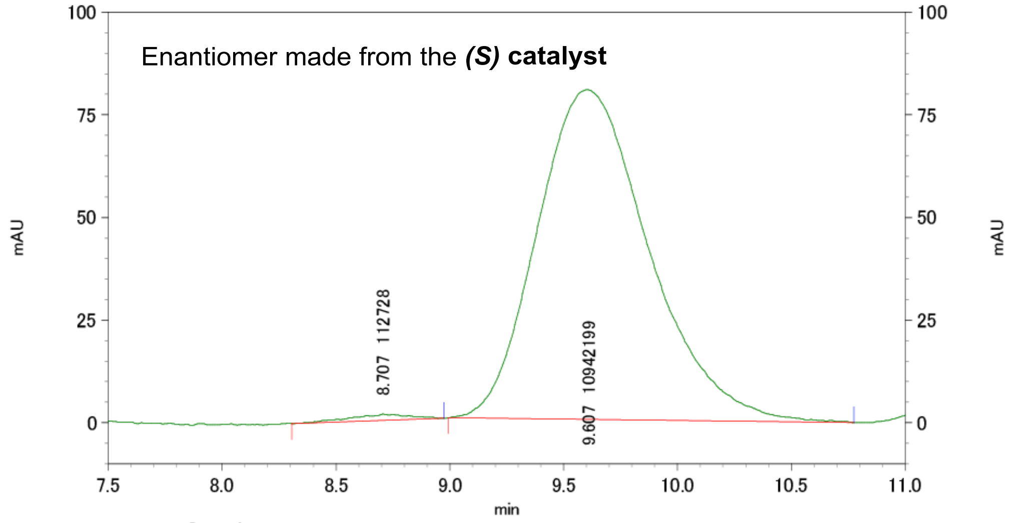

Result
2: 282 nm, 4 nm結果

Pk #	Retention time / min	Integration/ %
1	8.707	1.020
2	9.607	98.980
トータル Total		100.000

A mixture of both enantiomers of **24f**

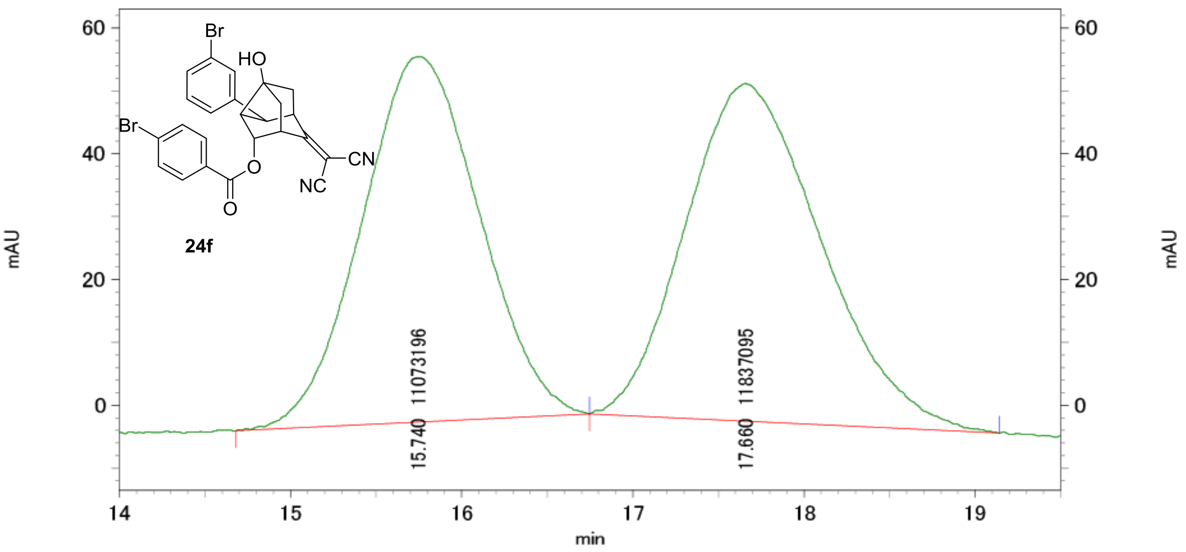

Result
1: 275 nm, 4 nm結果

Pk #	Retention time / min	Integration/ %
1	15.740	48.333
2	17.660	51.667
トータル Total		100.000

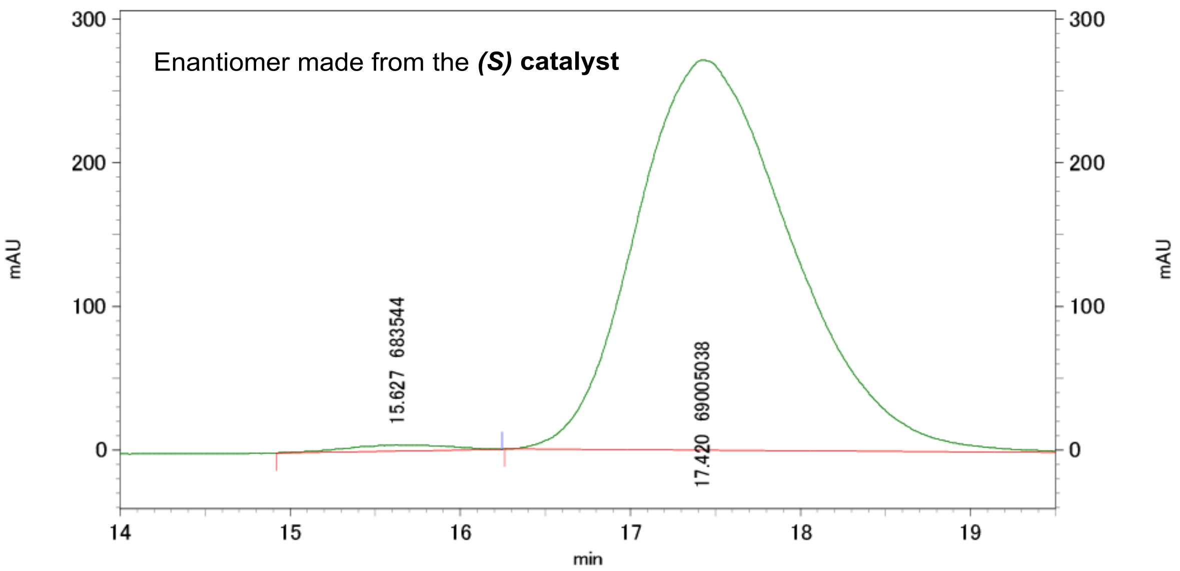

Result
1: 275 nm, 4 nm結果

Pk #	Retention time / min	Integration/ %
1	15.627	0.981
2	17.420	99.019
トータル Total		100.000

A mixture of both enantiomers of **24g**

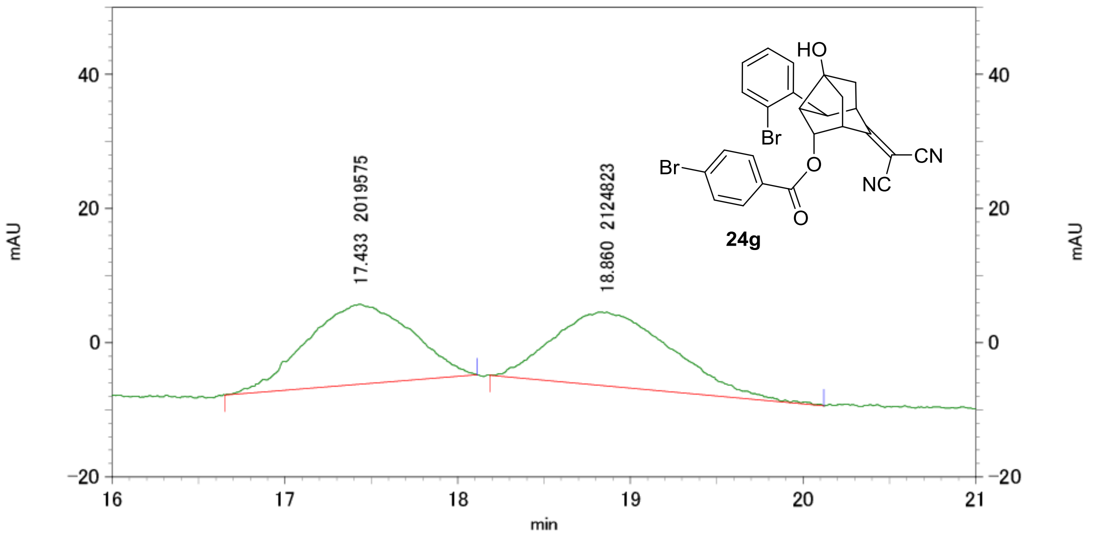

Result
1: 286 nm, 4 nm結果

Pk #	Retention time / min	Integration/ %
1	17.433	48.730
2	18.860	51.270
トータル<br>Total		100.000

Enantiomer made from the ***(S)* catalyst**

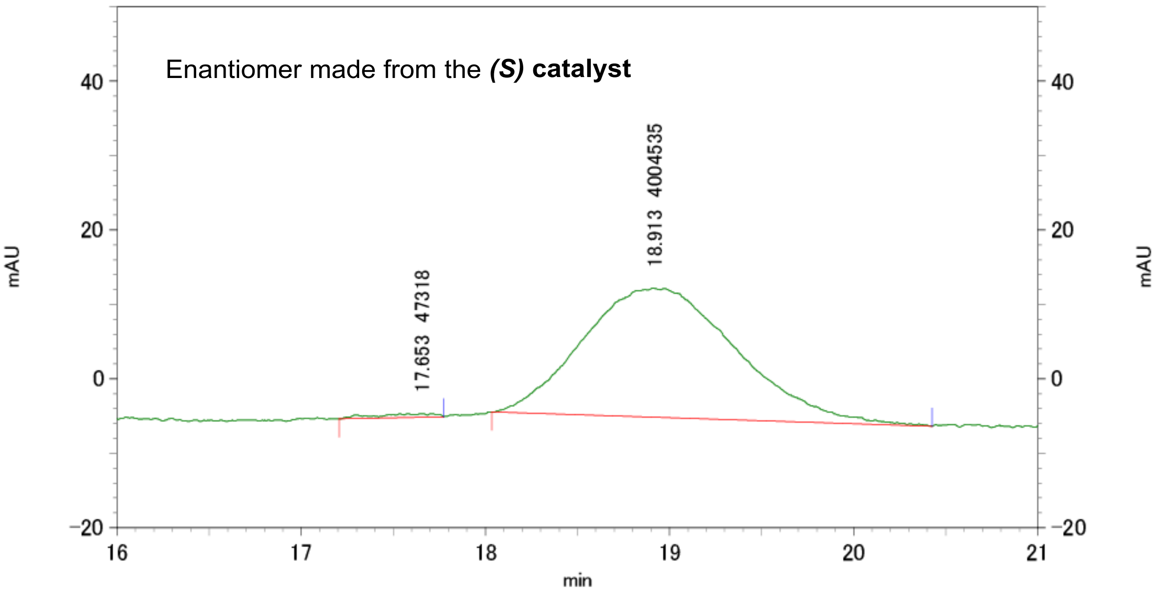

Result
1: 286 nm, 4 nm結果

Pk #	Retention time / min	Integration/ %
1	17.653	1.168
2	18.913	98.832
トータル<br>Total		100.000

A mixture of both enantiomers of **24h**

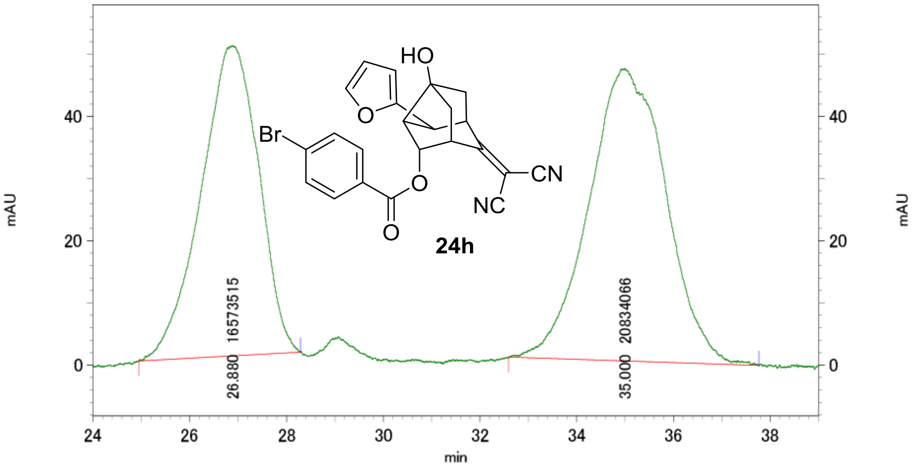

Result
1: 275 nm, 4 nm結果

Pk #	Retention time / min	Integration/ %
1	26.880	44.305
2	35.000	55.695
トータル Total		100.000

Enantiomer made from the ***(S)* catalyst**

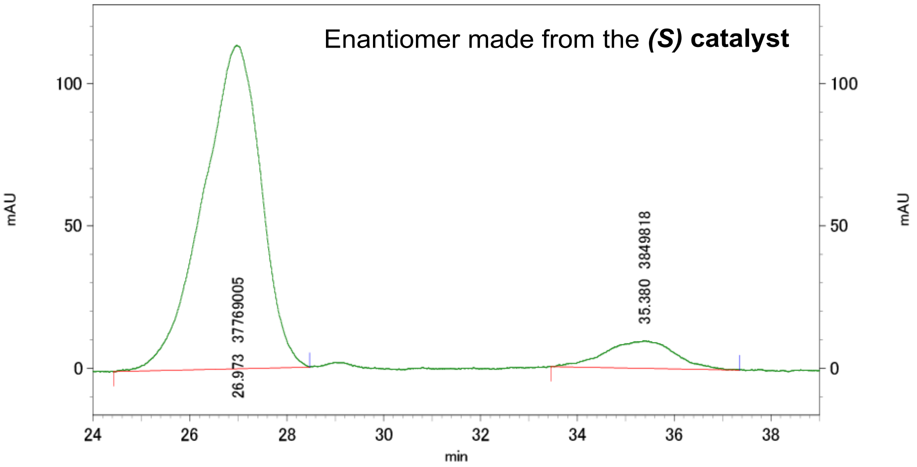

Result
1: 275 nm, 4 nm結果

Pk #	Retention time / min	Integration/ %
1	26.973	90.750
2	35.380	9.250
トータル Total		100.000

A mixture of both enantiomers of **17i**

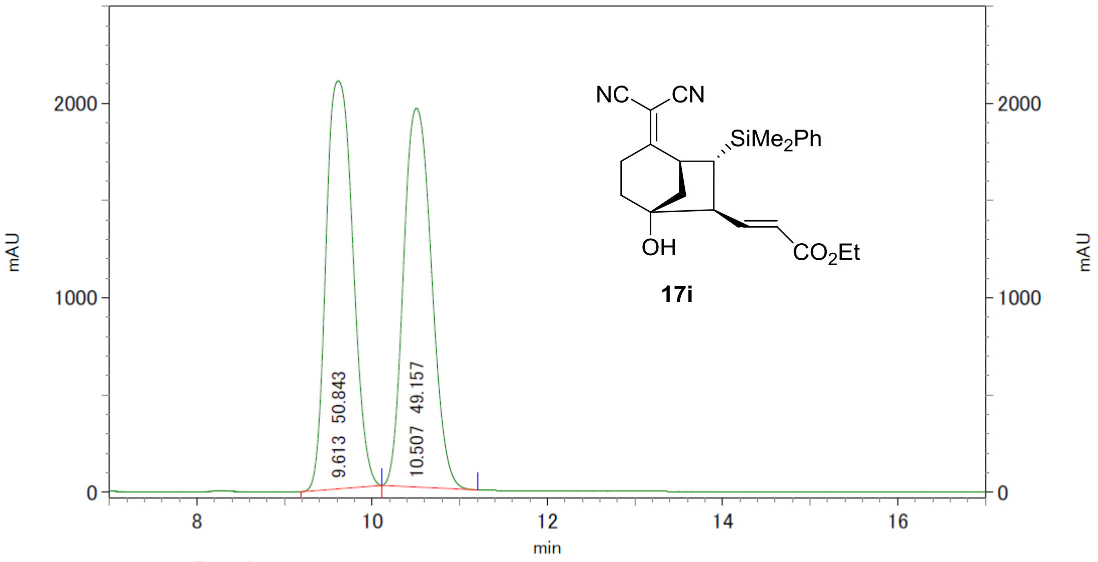

Result
1: 230.0 nm, 4.0 nm 結果

Pk #	Retention time / min	Integration/ %
1	9.613	50.843
2	10.507	49.157
合計 Total		100.000

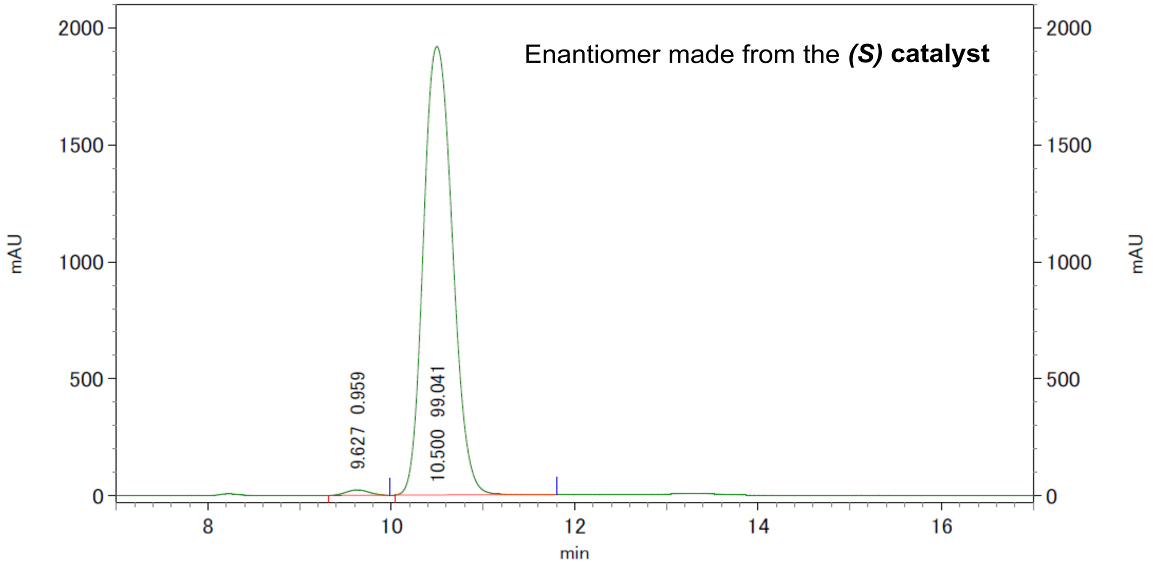

Result
1: 230.0 nm, 4.0 nm 結果

Pk #	Retention time / min	Integration/ %
1	9.627	0.959
2	10.500	99.041
合計 Total		100.000

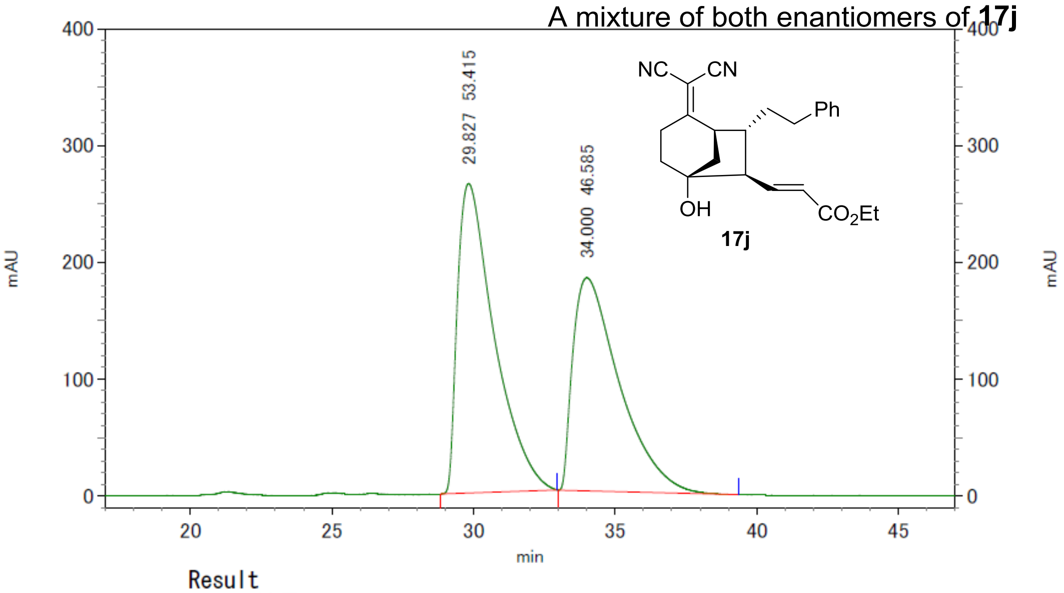

Result
1: 243.0 nm, 4.0 nm 結果

Pk #	Retention time / min	Integration/ %
1	29.827	53.415
2	34.000	46.585
合計 Total		100.000

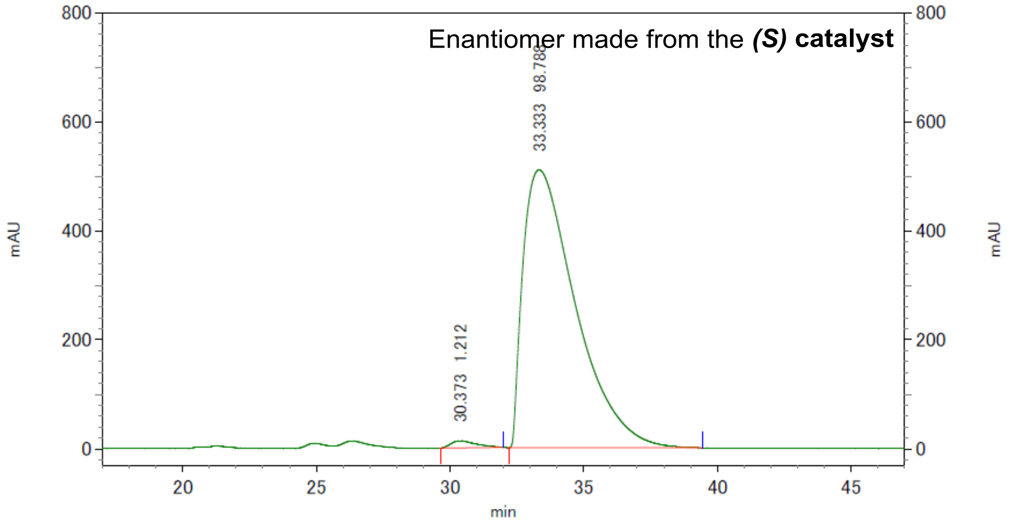

Result
1: 243.0 nm, 4.0 nm 結果

Pk #	Retention time / min	Integration/ %
1	30.373	1.212
2	33.333	98.788
合計 Total		100.000
